# CRISPR-Cas9 Editing in Maize: Systematic Evaluation of Off-target Activity and Its Relevance in Crop Improvement

**DOI:** 10.1038/s41598-019-43141-6

**Published:** 2019-04-30

**Authors:** Joshua Young, Gina Zastrow-Hayes, Stéphane Deschamps, Sergei Svitashev, Mindaugas Zaremba, Ananta Acharya, Sushmitha Paulraj, Brooke Peterson-Burch, Chris Schwartz, Vesna Djukanovic, Brian Lenderts, Lanie Feigenbutz, Lijuan Wang, Clara Alarcon, Virginijus Siksnys, Gregory May, N. Doane Chilcoat, Sandeep Kumar

**Affiliations:** 1 Corteva Agriscience™, Agriculture Division of DowDuPont™, Johnston, IA 50131 USA; 2 Institute of Biotechnology, Vilnius University, Vilnius, LT 10257 Lithuania

**Keywords:** Molecular engineering in plants, Plant molecular biology

## Abstract

CRISPR-Cas9 enabled genome engineering has great potential for improving agriculture productivity, but the possibility of unintended off-target edits has evoked some concerns. Here we employ a three-step strategy to investigate Cas9 nuclease specificity in a complex plant genome. Our approach pairs computational prediction with genome-wide biochemical off-target detection followed by validation in maize plants. Our results reveal high frequency (up to 90%) on-target editing with no evidence of off-target cleavage activity when guide RNAs were bioinformatically predicted to be specific. Predictable off-target edits were observed but only with a promiscuous guide RNA intentionally designed to validate our approach. Off-target editing can be minimized by designing guide RNAs that are different from other genomic locations by at least three mismatches in combination with at least one mismatch occurring in the PAM proximal region. With well-designed guides, genetic variation from Cas9 off-target cleavage in plants is negligible, and much less than inherent variation.

## Introduction

Humans have been practicing crop improvement for many millennia. Since the advent of the Green Revolution in the 1960’s, agriculture has used modern technology to improve the yield and nutritional quality of crops^1^. Conventional breeding, however, is unlikely to keep up with the world’s increasing food demand^2^. Genome editing approaches utilizing site-directed endonucleases (SDNs) capable of making chromosomal double-strand breaks (DSBs)^3–7^ can help overcome the limitations of conventional breeding and accelerate development of improved crops. By harnessing natural cellular DNA repair processes, DSBs can be used to introduce targeted disease resistance, genome edits to improve agronomic traits (e.g., plant grain yield, nutritional content)^8–12^ or speed-up domestication, ultimately, helping to accelerate the development of new beneficial plant varieties for society^13^. These edits include small insertion or deletion (indel) modifications that can knockout the expression of a gene (SDN1), or correction of the DSB with a homologous template DNA resulting in the precise alteration of a sequence (SDN2), or the targeted insertion of a new DNA sequence (SDN3)^14,15^

CRISPR (clustered regularly interspaced short palindromic repeats)-Cas9^6^ has emerged as a robust and versatile DSB tool with broad applications for gene discovery, trait development and expedited breeding in crop species. Cas9 endonuclease and guide RNA can be delivered into plant cells as DNA, RNA or ribonucleoprotein (RNP) to cleave target DNA sequence(s) in the genome. However, in addition to the intended target (on-target) site, Cas9 can potentially cause off-target DSBs at genomic locations with significant sequence similarity to that of the intended target sequence^16,17^ resulting in the possibility of off-target edits. To mitigate the potential for off-target editing, a variety of approaches have been developed. These include protein engineering^18–20^, genome editing using ribonucleoprotein (RNP)^21–24^, biochemical and cellular assays to empirically assess target specificity^25–29^, and computational identification of Cas9 targets with low potential for off-target editing^30–34^.

Here we explored the specificity of *Streptococcus pyogenes* Cas9 (Cas9) endonuclease in the complex crop genome of maize (*Zea mays* L.) using a comprehensive approach that included: (1) *in silico* computational prediction of an off-target site portfolio; (2) biochemical confirmation of off-target cutting activity and (3) surveillance of candidate off-target sites in a cellular context. The effect of off-target editing as a function of Cas9 and guide RNA delivery was assessed by three different methods: DNA-free (using RNPs and particle gun (PG)), and DNA-based delivery using *Agrobacterium* or PG.

Our results show that bioinformatic selection of unique target sites can be used as a reliable tool to mitigate the potential for off-target editing in crop plants with reference genomes. Potential off-target sites were identified with genome-wide biochemical assay, CLEAVE-Seq, that provides increased sensitivity over similar methods^26,28^. The biochemically identified sites were subsequently monitored using molecular inversion probes (MIPs) in maize plants subjected to Cas9 editing. Computationally unique targets demonstrated no evidence of off-target cutting in a cellular context, while up to ~90% of all the observed alleles had on-target activity. At a limited number of genomic sites, we also report that inherent genetic variation in the genotype used in this study far exceeded potential genetic changes generated by CRISPR-Cas9 genome editing techniques. Therefore, without a targeted approach such as one described here, whole genome sequencing may not be a practical way to differentiate CRISPR-Cas9 off-target effects from inherent background variation in plants.

To our knowledge, this is the first comprehensive study of CRISPR-Cas9 specificity in plants highlighting prediction and validation of unintended genome editing, and their relevance in the background of innate genetic variation.

## Results

**Figure 1:**
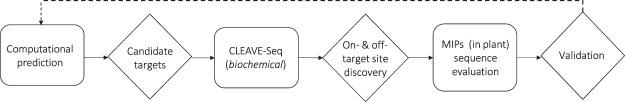
Overview of biochemical off-target site identification and in plant validation workflow.

We designed a three-step approach to evaluate the specificity of CRISPR-Cas9 editing activity. This included the computational prediction of target specificity, the biochemical capture and identification of genomic sequences susceptible to Cas9 induced DSBs, and off-target site validation in plants (Fig. 1). First, Cas-OFFinder^32^ was utilized to predict the specificity of targets. Next, a new biochemical method, CLEAVE-Seq, was used for the biochemical discovery of candidate off-target sites as the method avoids potential complex steps associated with other biochemical methods^25,27,35^ and enhances on- and off-target discovery. Finally, MIPs analysis was performed in plants to examine off-target sites identified computationally and confirmed biochemically^36^. MIPs was chosen due to its scalability for throughput and multiplexable analysis capability. Thus, permitting many genomic loci to be monitored simultaneously for off-target non-homologous end-joining (NHEJ) mutations. A similar two-step strategy, Verification of *In Vivo* Off-targets (VIVO), has recently been used to identify and evaluate off-target cutting in the mouse genome^37^.

### Target sites and computational predictions

**Figure 2:**
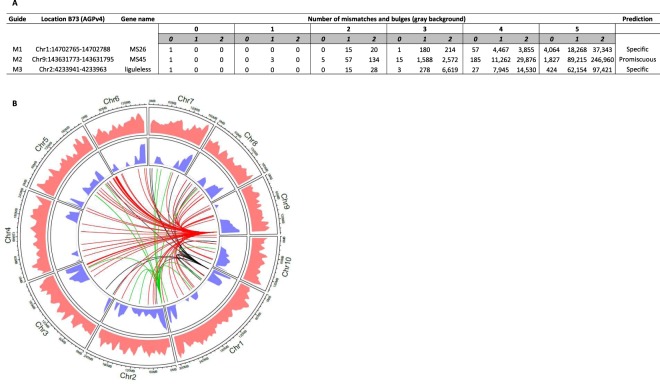
Computational prediction of guide and off-target sites. (**A**) Summary table indicating number of genomic sites with up to 5 mismatches and 1 bulge. (**B**) Circos plot representing the guides used for this study and their potential off targets. Layers outermost to innermost: B73 AGPv4 chromosomes, repeats (pink), genes (blue), guides and their computationally predicted off-sites up to 2 mismatches and 1 bulge. All lines originate from the on-site. Black: M1, Red: M2, Green: M3.

Three targets sites spread across different chromosomes in the maize genome (Fig. 2) were selected for analyses. Each target was chosen to be within a gene non-essential for embryonic cell proliferation and plant regeneration. Guide RNA design and prediction was accomplished using Cas-OFFinder^32^ set to search for all potential off-target sites with up to 5 mismatches and 2 bulges between the guide RNA and DNA target sequence. Guide RNAs M1 and M2 were designed to target the male sterile 26 (*Ms26*)^38,39^ and 45 (*Ms45*) genes^40,41^, respectively. The M3 guide RNA was designed to target the liguleless 1 (*Lig1*) gene^42^. Only targets adjacent to an appropriate protospacer adjacent motif (PAM) for Cas9 (NAG or NGG) were considered. M1 and M3 were designated as specific based on their lack of homology with other sequences in the maize B73 reference genome, AGPv4^43^. For example, the most closely related sequences to M1 and M3 targets were different by at least 2 mismatches and 1 bulge. Additionally, these guides had no predicted off-target site containing a perfect match within the PAM proximal seed region (10 bases 5′ of the PAM)^44,45^ of their respective target site (Supplementary Tables 1 and 2). In contrast to M1 and M3 guides, M2 guide had multiple matching genomic sites (Fig. 2) with 1 or 2 mismatches and at least 1 bulge, without any mismatch or bulge in the PAM proximal seed region (Fig. 2A and Supplementary Table 3). The M2 guide, designated as promiscuous, was intentionally selected for its potential of inducing off-target edits, which would also serve as a positive control for method validation.

### Genome-wide identification of off-target sites

The genomic specificity of Cas9 was examined biochemically using CLEAVE-Seq at 37 °C. Previous experiments in maize demonstrated that the modifications made here (described in the Methods section) increased the number of Illumina reads mapping to various cut sites biochemically, both on-target and off-target, by approximately 10-fold over the original SITE-Seq protocol (Supplementary Fig. 1). Specifically, a phosphatase treatment step prior to RNP cleavage was added to reduce adapter ligation to random ends generated by physical shearing of genomic DNA templates during DNA extraction and pipetting. An additional step of DNA release through NotI cleavage after biotinylated adapter ligation and biotin selection, followed by exonuclease treatment and second strand synthesis with a semi-random primer (see Methods) was added to the protocol. This step further improves specificity of the final PCR amplification step prior to DNA sequencing, replacing PCR amplification performed directly off streptavidin bead-bound DNA in the original protocol. Two independent CLEAVE-Seq replicates were performed for each guide.

Analysis was performed by scanning chromosomally mapped CLEAVE-Seq data for discontinuities in sequence read coverage within a +/−2 bp window of the expected cut site for Cas9 (3 bp 5′ of the PAM) similar to that described in Cameron, *et al*.^28^. Since Cas9 has been reported to generate off-target cutting in eukaryotic cells in sequences with up to 5 mismatches and tolerate bulges between guide RNA and DNA target recognition^25,46–48^, we limited our search for CLEAVE-Seq signatures to protospacer targets within 5 mismatches and 2 bulges (a total of 7 differences) from the on-target protospacer sequence (Fig. 2A). To account for off-targets generated by mismatches in PAM recognition, protospacer targets adjacent to a 3′ prime NAG were also considered in addition to the canonical 3′ NGG PAM. Next, this collection of computationally predicted sites, permitting up to 8 differences from the on-target site, were searched for the presence of cleavage signatures.

CLEAVE-Seq was first performed for the promiscuous M2 guide RNA to optimize the target discovery process for the two-remaining guide RNAs computationally predicted to be specific. As observed previously with other biochemical based methods^25,26,28^, many cleaved genomic target sites were detected (3,052 in total (Supplementary Table 4). This feature of off-target detection methods is perhaps due soley to their biochemical nature. Being devoid of a cellular, nuclear, and chromosomal context, we speculate that Cas9 and guide RNA under high persistent concentrations are capable of cleaving target sites that are less likely to be edited in cellular context^49^. Next, the average number of reads (normalized based on CLEAVE-Seq library read depth) identified at the target cut-site were compared with the number of mismatches and bulges relative to the on-target site. For example, 227 CLEAVE-Seq reads (normalized) were recovered at the on-target site, M2-1 (Table 1). A relatively high read count (>40) was observed for the sites M2-2, M2–4 and M2-6, which all carried 1-2 mismatches and 1-2 bulges (Table 1). None of the mismatches or bulges in these sites were within the PAM-proximal seed region. In comparison, the rest of the sites, all with significantly lower read counts, contained 2 or more mismatches and 1 or more bulge, but with the difference that at least 1 mismatch was located within the seed region (Table 1) or PAM (Supplementary Table 4). This observation confirms that the binding interaction between guide RNA and target sequence is more sensitive to mismatches within the PAM proximal seed sequence^50^. Interestingly, off-target M2-6 resulted in more reads than the on-site. This could be the result of more robust biochemical cleavage efficiency at this target under the digest conditions used or the result of enhanced recovery for this location by CLEAVE-Seq. Finally, low numbers of CLEAVE-Seq reads were observed from two of the computationally predicted sites with 1–2 mismatches and 1 bulge (M2-3, M2-5 in Table 1 & site 4 in Supplementary Table 5). In each of these three cases, each site had either a bulge or a mismatch in the seed region.

**Table 1:**
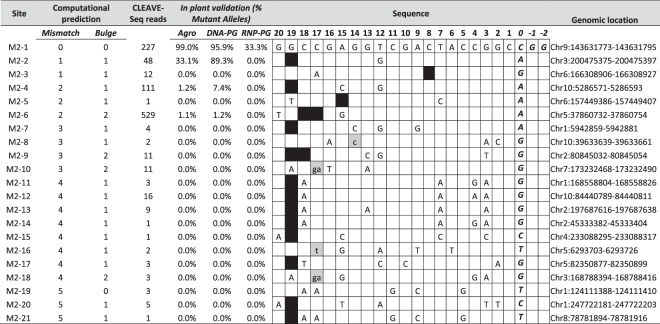
CLEAVE-seq data and validation of M2 sites in plants. On-target site M2-1 is shown on the top. Percent mutant allele is number of alleles with mutation/total number of alleles observed. DNA and RNA bulges are shown as gray and black boxes, respectively. Lower case indicates additional nucleotides in RNA. Mismatch is shown as a letter indicating the nucleotide, absence of a nucleotide indicates no mismatch. PAM is italicized.

Next, CLEAVE-Seq was performed for the two guides computationally predicted to be specific, M1 and M3. Similar to the CLEAVE-Seq data obtained from guide M2, high on-target normalized read counts were detected for both M1 (Table 2, M1-1) and M3 (Table 3, M3-1). No other M1 or M3 sites were detected with more than 19 normalized reads, except for the M1-2 site where 93 CLEAVE-Seq reads were recovered. This target had 2 mismatches and 2 bulges with 1 mismatch being within the PAM proximal seed sequence of the protospacer (Table 2).

**Table 2:**
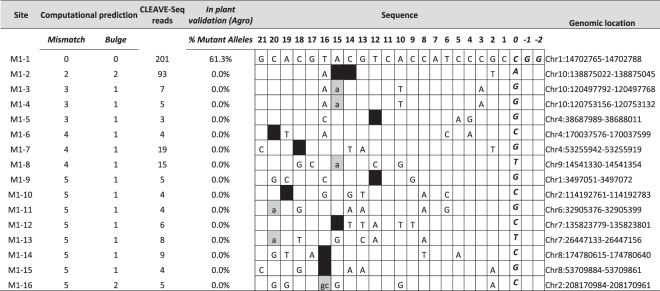
CLEAVE-seq data and validation of M1 sites in plants. On-target site M1-1 is shown on the top. Percent mutant allele is number of alleles with mutation/total number of alleles observed. DNA and RNA bulges are shown as gray and black boxes, respectively. Lower case indicates additional nucleotides in RNA. Mismatch is shown as a letter indicating the nucleotide, absence of a nucleotide indicates no mismatch. PAM is italicized.

**Table 3:**
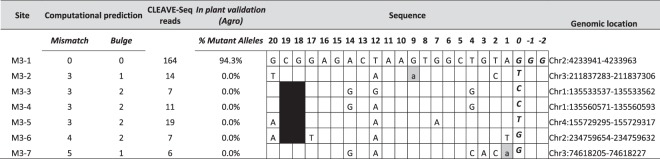
CLEAVE-seq data and validation of M3 sites in plants. On-target site M3-1 is shown on the top. Percent mutant allele is number of alleles with mutation/total number of alleles observed. DNA and RNA bulges are shown as gray and black boxes, respectively. Lower case in gray box indicates additional nucleotide(s) in RNA. Mismatch is shown as a letter indicating the nucleotide, absence of a nucleotide indicates no mismatch. PAM is italicized.

### Validation of potential off-target sites in plants

Following the computational and biochemical identification of potential off-target sites for M2, we validated off-target cleavage in maize using three different plant Cas9-guide RNA delivery methods: DNA-free (using RNP and particle gun (PG), and DNA-based using *Agrobacterium* or PG. First, 21 genomic targets sites, including the on-target site, were selected from a wide range of CLEAVE-Seq read count conditions and examined in a cellular context for evidence of DNA cleavage and repair. Reasoning that genomic targets with a higher CLEAVE-Seq read coverage are more likely to be cut, all sites with greater than or equal to 40 normalized reads originating from the cut-site were selected. Additionally, targets were selected to come from all mismatch (1-5) and bulge (1-2) categories (Supplementary Table 6). To validate our target discovery process, an additional 75 targets not identified by CLEAVE-Seq but computationally predicted with different mismatch classifications, were also selected for characterization in maize plants (Supplementary Table 6). MIPs analysis was then performed on all 96 targets in ~300 T_0_ plants exposed to Cas9 and the M2 guide RNA using three different plant delivery methods, *Agrobacterium*, particle gun (PG)-mediated DNA or RNP (Supplementary Table 7). High efficiency on-target mutagenesis (>95% of the total number of alleles) was observed in the T_0_ maize plants obtained using *Agrobacterium* or PG-mediated DNA delivery (Table 1, M2-1 site). Targeted mutagenesis was also obtained using PG-mediated RNP delivery, although the frequency of alleles mutated using this method was much lower (33% of the total alleles analysed). No off-target activity was observed in any of the T_0_ plants using the RNP delivery.

As expected, M2 off-target sites identified by CLEAVE-Seq were also mutated with *Agrobacterium* and PG-mediated DNA delivery, though at varying frequencies (Table 1). High-frequency off-target activity was observed at site M2-2 with 33% and 89% mutant alleles obtained using *Agrobacterium* and PG-mediated DNA delivery methods, respectively. Low-frequency off-target mutation (1.1–7.4% of total alleles analysed) was also detected at sites M2-4 and M2-6. No activity was observed for the other 17 candidate off-target sites identified with CLEAVE-Seq. Notably, only off-target sites with a normalized CLEAVE-Seq read count greater than 40 were modified in plants (Table 1). Moreover, analysis of the 75 computationally predicted M2 targets not detected by CLEAVE-Seq also did not produce evidence of cutting activity in plants.

Since *Agrobacterium*-mediated DNA delivery provided the highest frequency of on-target site editing with the lowest propensity for off-target cleavage, validation of potential off-targets for M1 and M3 guide RNAs in plants was performed only with this method. Additionally, given that off-targets demonstrated to be cleaved biochemically were modified in plants, MIPs analysis was conducted only for those sites identified by CLEAVE-Seq. Off-target sites for M1 and M3 guide RNAs were selected from a wide range of read counts with a preference being given to those with higher normalized read coverage. Similar to guide RNA M2, MIPs analysis revealed robust on-target activity for M1 and M3 guide RNAs with mutation frequencies of 61% and 94%, respectively, of the total alleles analysed in ~50 T_0_ plants (Tables 2 and 3, respectively; Supplementary Table 7). In contrast to guide RNA M2, M1 and M3 guide RNAs produced no detectable off-target activity.

Next, computational predictions and data from CLEAVE-Seq and MIPs analyses were examined for trends that could be utilized to avoid off-target cleavage in plants. For guide RNAs M1, M2, and M3, CLEAVE-Seq identified targets with a normalized read count of greater than 40 yielded evidence of off-target cleavage and repair in plants (Tables 1–3), which suggests that targets with a higher read count tend to be more prone to modification in plants. Overall, this feature of CLEAVE-Seq translated well, accurately predicting 6 of the 7 targets showing editing in plants. Moreover, none of the targets modified in plants contained mismatches or bulges in the PAM proximal region of the protospacer and fewer than a combination of 5 mismatches and/or bulges in the PAM distal region of the protospacer. Taken together, this indicates that off-target editing can be significantly reduced in plants by computationally selecting unique Cas9 targets that are different from other genomic locations by at least a combination of 5 mismatches and/or bulges in the PAM distal region or at least three differences in the PAM distal region with at least one additional discrepancy in the PAM proximal region (Tables 1–3).

### Natural variation in control plants

To evaluate polymorphism in the Hi-II maize genotype used in this study, we obtained MIPs data for 19 M1, 96 M2, and 8 M3 sites obtained from 500 non-transformed control plants. The MIPs data when compared to B73 AGPv4 revealed single nucleotide variations (SNVs) in 50% of the M2 sites analysed. Similarly, SNVs were observed in 12% and 16% of M3 and M1 MIPS sites, respectively (Table 4). In total, 228 variant nucleotides were observed out of 8033 nucleotides sequenced from 96 M2 sites.

**Table 4:** Inherent variation in Hi-II genotype.

Guide	No of Sites	Nucleotides sequenced	Variant sites	Variant nucleotides
M1	19	1078	3 (16%)	4
M2	96	8033	48 (50%)	228
M3	8	480	1 (12%)	2

Same “number of sites” (Column 2) were MIPS analyzed in T_0_ plants CRISPR/Cas9-treated plants. “Nucleotides sequenced” indicate total sequence coverage calculated by multiplying sequence length of MIPS assay to number of sites analyzed. “Variant sites” show the total number of sites observed with at least one nucleotide variation. “Variant nucleotides” indicate total number of nucleotide variation observed.

## Discussion

CRISPR-Cas based genome editing is very precise^51,52^ compared to other crop improvement technologies such as traditional or mutational breeding. However, unintended off-target cleavage and editing can occur at locations within the genome that share significant sequence similarity to the intended target site^17,50,53^. In this report we assessed CRISPR-Cas9 specificity in maize, a crop plant with a complex genome similar in size to the human genome. Three different genes, *Ms45*, *Ms26*, and *Lig1*, each residing on one of the three different chromosomes, were targeted for cleavage with three different Cas9-guide RNA delivery methods (DNA-free (using RNP and PG), and DNA-based delivery using *Agrobacterium* or, PG). Two specific and one promiscuous guide RNAs were used to develop our methodologies. Biochemically cleavable genomic off-target sites were subsequently identified using a new sensitivity enhanced biochemical method, CLEAVE-Seq. Next, we validated our biochemical cleavage method, first by examining a portfolio of targets not detected by CLEAVE-Seq but computationally predicted as potential off-target sites. Finally, the relevance of biochemically identified targets to cleavage in T_0_ plants was established. In all, none of the computationally predicted targets that were not detected by CLEAVE-Seq yielded sequence alterations in plants, while 6 of the 7 (86%) targets identified by CLEAVE-Seq with a read coverage greater than 40 were modified. RNP delivery with no off-target cutting being observed with the promiscuous M2 guide, provided highest specificity, which is consistent with previous reports^21–24^. This enhancement to specificity, however, came with a lower on-target mutation frequency. Also, DNA-PG due to its propensity to deliver more copies of the expression cassettes resulting in higher cellular concentrations of Cas9-gRNA, could be a better approach to evaluate potential off-target activity. Overall, the delivery of Cas9 and guide RNA by *Agrobacterium* provided the best balance between on-target editing and off-target cleavage for future applications aimed at plant improvement.

The results from the M2-6 site in this report present an intriguing case study. CLEAVE-Seq read coverage for this site far exceeded on-target (M2-1) site. However, unexpectedly low mutation frequency was observed in T_0_ plants. Interestingly, the M2-6 target site was mapped to an intergenic region while all other target sites exhibiting more robust mutation frequencies were located within genic regions (M2-1: *Ms45* and M2-2: uncharacterized gene LOC100217080). This finding is consistent with previous report where indel frequencies were significantly reduced in the absence of gene transcription^54^.

Our data confirms previous reports demonstrating high-specificity of CRISPR-Cas9-mediated genome editing in plants^50,51,55–57^. Expected off-target activity in plants was observed for the promiscuous M2 guide RNA at sites predicted bioinformatically and identified biochemically. All sites showing evidence of cutting in plants were observed to contain mismatches and/or bulges only in the PAM distal region (nucleotides 11–20) outside of the PAM proximal region (nucleotides 1–10) reported to be the seed region necessary for establishing R-loop formation^44,58,59^. This hyper-sensitivity to mismatches in the seed region of the target sequence has previously been reported in plants^50^ and documented in biochemical studies examining Cas9 specificity during R-loop formation^58^. Taken together, our data fully support the notion of computationally predicting specific target sites with at least a 3-nt mismatch, bulge, or mismatch bulge combination with at least one difference being in the seed sequence of the protospacer to ameliorate off-target Cas9 activity^50^. Interestingly, these results are inconsistent with Cas9 specificity reports in mammalian cells^25–27^. One major difference between mammalian and plant cells are the temperature at which tissue cultures are maintained. In the case of human cells, this is 37 °C while for maize the temperature is significantly lower, 28 °C. This difference may have significant effects on Cas9 cleavage activity and specificity^60^.

Inherent genetic diversity in maize is extensive^61^. Maize high type II (Hi-II) line^62^ was generated by crossing two partially inbred lines (Hi-II Parents A and B) selected from a cross between A188 and B73 which demonstrated a greatly improved Type-II tissue culture response. MIPs analysis of 96 M2 targets revealed variation in 50% of the sites among 500 non-treated plants screened in this study. A portion of the genetic variation observed could be attributed to the parental origins of the Hi-II line but also may be the result of *de novo* spontaneous mutations over many generations. Genetic variation due to spontaneous mutations during multiple generations of breeding is well established in plants including major food crops^63,64^. Additionally, all conventional crop improvement methods including crosses between different genotypes, varieties, and species, chemical or irradiation mutagenesis, and plant tissue culture inherently produces random genetic variation^50,63–71^. Taken together, our study indicates that with appropriately designed guide RNAs, genetic changes observed through CRISPR-Cas9 off-target editing are negligible and much less than the naturally occurring diversity in plants.

The work described herein illustrates that CRISPR-Cas9 is remarkably specific and efficient at generating on-target genome edits. Robust bioinformatics tools alleviate the potential for off-target cleavage by identifying targets that are unique within the genome by at least combination of 3 mismatches and/or bulges with at least 1 difference within the PAM proximal seed region. Furthermore, biochemical identification of off-target sites using CLEAVE-Seq followed by MIPs validation in plants complements computational guide RNA predictions, in particular when the intended target site is not unique and has highly similar sequences elsewhere in the genome. While CRISPR-Cas9 has the potential to generate off-target cutting in genomic sites that are substantially similar to the target site, off-target edits are likely to be negligible in the background of existing natural variation and continuous unintended changes being generated during the plant breeding process. Finally, regardless of the breeding method, standard practices of commercial crop development include advancement of candidate lines following extensive agronomic evaluations specific for a given crop. This has proven to be an effective tool to eliminate plants with undesirable characteristics resulting in crops with a history of safe use. Therefore, concerns related to specificity of CRISPR-Cas9 technology in crop improvement have little relevance.

## Methods

### Computational prediction of target sites and guide selection

Target site selection was based on the number of potential off-target sites observed at 0, 1, 2, 3, 4, and 5 nt mismatches from the guide RNA protospacer target permitting up to 2 bulges between guide RNA spacer and protospacer target sequence. Mismatch and bulge assessments were performed using Cas-OFFinder^32^ against the B73 (one of the Hi-II parent line) reference AGPv4^43^ using a NRG protospacer adjacent motif (PAM). We used a command line/custom version of Cas-OFFinder to include reference AGPv4. Two target sites were selected to be specific (have low off-target potential) and one target site was chosen to be promiscuous (have high off-target potential) based on the number of homologous targets identified with up to a combination of 2 mismatches and bulges (Fig. 2A,B).

### *Cas9* protein and guide RNA molecules

Recombinant *Cas9* protein containing a C-terminal 6X His was expressed and purified from *E. coli* as described previously in Karvelis, T. *et al*.^72^. Single guide RNAs (guide RNAs) were generated by T7 *in vitro* transcription using AmpliScribe™ T7-Flash™ kit (Epicentre, USA) according to the manufacturer’s recommendations. Products were purified using NucAway™ Spin Columns (Invitrogen, Life Technologies Inc., USA) followed by ethanol precipitation.

### CLEAVE-Seq

On-site and off-site detection was performed using a new biochemical method called CLEAVE-Seq (Supplementary Fig. 3A). Briefly, 3 µg of high-molecular weight genomic DNA was treated with 1U FastAP Thermosensitive Alkaline Phosphatase (Thermo Fisher Scientific, USA) in 1X FastAP Buffer in final 20 µl volume at 37 °C for 1 hour, followed by 20 min at 80 °C. The treatment of genomic DNA with a phosphatase prior to Cas9 digestion facilitates removal of 5′-phosphates from random fragmented DNA ends to prevent their ligation to the adapter during the adapter ligation step below. After phosphatase treatment, DNA (20 µl) was combined with a previously assembled RNP (20 µl). For RNP assembly, Cas9 (final concentration 2 μM) and guide RNA (final concentration 4 μM) were incubated at 37 °C for 1 hour in Assembly Buffer (10 mM Tris-HCl (pH7.5 at 25 °C), 100 mM NaCl, 1 mM DTT, 1 mM EDTA) and later adding 1 M MgCl2 to the final concentration of 16 mM. After incubation of DNA with RNP for 1 hour at 37 °C, followed by 20 min at 80 °C, Adapters 1 (Upper: 5′-Biotin-AGTTACGCAACCGAGACGCGGCCGCsGsTsGsACTGGAGTTCAGACGTGTGCTCTTCCGATCT-3′, where “s” stands for PTO modification; Lower: 5′ AGATCGGAAGAGCACACGTCTGAACTCCAGTCACGCCCGGGCGTCTCGGTTGCddC-3′) were ligated to the Cas9-digested DNA by adding 40 µl of Ligation Mix (5 mM Tris-HCl (pH8.0 at 25 °C), 10 mM Mg-acetate, 20 mM DTT, 1 mM ATP, 7.5% PEG 4000 (50 w/v) 0.5 U/µl T4 DNA ligase (Thermo Scientific), 2 µM Adapters 1) final 80 µl volume and incubating at 22 °C for 1 hour in, followed by 80 °C for 20 min. After adapter ligation, nick removal was performed by adding 20 µl of Nick Removal Mix (5X Taq Buffer with (NH4)2SO4 (Thermo Scientific), 1 mM dNTPs, 20 mM EDTA, 0.5 U/µl DreamTaq DNA polymerase (Thermo Fisher Scientific) and in final 100 µl volume incubating at 72 °C for 15 min.The reaction was stopped by adding 5 µl 0.5 M EDTA. After shearing the DNA with a M220 Focused-ultrasonicator (Covaris, USA) using microTUBE AFA Fiber Pre-Slit Snap-Cap tubes (Covaris, USA) and followed shearing conditions: 50 W peak incident power, 20% duty factor, 200 cycles per burst, 200 s duration at 20 °C. Residual adapters were removed using a MagJET NGS Cleanup and Size Selection kit (Thermo Fisher Scientific), following the manufacturer’s instructions. DNA was resuspended in 50 µl Elution Buffer (Thermo Fisher Scientific) and purified using 25 µl Dynabeads MyOne Streptavidin C1 beads (Thermo Fisher Scientific), following the manufacturer’s instructions. Bead-bound DNA then was resuspended in 25 µl 1X FastDigest Buffer (Thermo Scientific) and cleaved with 1 µl of FastDigest NotI (Thermo Fisher Scientific) at 37 °C for 30 min. Supernatant was collected using a magnetic stand and digested with Lambda Exonuclease (Thermo Fisher Scientific) by adding 95 µl of Exonuclease Mix (67 mM glycine-KOH (pH9.4 at 25 °C), 0.01% Triton X-100, 0.2 U/µl Lambda Exonuclease) and incubating at 37 °C for 1 hour in final 120 µl volume, followed by 20 min at 80 °C. The resulting single-stranded DNA was purified using an EpiJET Bisulfite Conversion kit column (Thermo Fisher Scientific), following the manufacturer’s instructions, and eluted in a total of 20 µl water. Second strand synthesis then was performed first by adding Second Strand Synthesis primer (5′-CCCTACACGACGCTCTTCCGATCTNNNNNNNNNNNN-3′) to a final concentration of 135 nM in 1X T4 DNA Polymerase buffer (Thermo Fisher Scientific) additionally supplemented with 4 mM Mgacetate and 8.8% (w/v) PEG8000, incubating for 1 min at 98 °C in final 45.5 µl volume, then slowly cooling down to 25 °C by setting the ramp speed to 10% (0.5 °C/second) followed by 30 min at 25 °C before adding 3.5 µl dNTP mix (10 mM each) and 5U T4 DNA polymerase and incubating at 25 °C for 15 min. Double-stranded DNA was purified using the Purification Module with Magnetic Beads (Lexogen, Austria), following manufacturer’s instruction. To avoid under- or overamplification of the DNA library the optimal number of cycles was determined via qPCR as described in QuantSeq/SENSE for Illumina Kit (Lexogen). DNA fragments were amplified and barcoded using the PCR Add-on Kit for Illumina and i7 Index Plate for QuantSeq/SENSE for Illumina (Lexogen, Austria) with the following cycling steps: 98 °C for 2 min, followed by 98 °C for 10 s; 65 °C for 20 s; 72 °C for 30 s (number of cycles determined via qPCR), and hold at 4 °C. The amplified DNA was purified and size-selected using the Purification Module with Magnetic Beads (Lexogen), and quantified using a Qubit fluorometer prior to sequencing on an Illumina HiSeq 2500 sequencer.

### CLEAVE-Seq analysis

Analyses to identify cleaved genomic targets were performed as described in Supplementary Fig. 3B.

#### Physical mapping of CLEAVE-Seq data

Paired end read FASTQ files were pre-processed with Skewer v0.2.2^73^ to remove Illumina universal adapters and enforce a minimum surviving read length of 35 bp. Then, bowtie 2-build v2.3.2^74^ was used to index the reference sequences with default parameters and Bowtie 2 was used to perform non-discordant (for paired input), end to end alignments of the reads to the genomic reference.

#### Generation of normalized read coverage profiles

Normalized read coverage profiles were built for each sample starting with the negative control (a CLEAVE-Seq reaction assembled in the absence of a guide RNA) followed by the treated experimental samples. Coverage counts were calculated for reads initiating within a +/− 2 bp window surrounding the expected site of cleavage (3 bp 5′ of the protospacer adjacent motif (PAM)) of each computationally predicted target site using BEDTools^75^. The total number of reads originating from the cut-site was then adjusted by dividing it by a normalizer (established by dividing the sample’s read depth by the lowest read depth present in the dataset). Normalized read coverage profiles between replicates were averaged.

#### Filtering for biochemically cleaved sites

To identify biochemically cleaved target sites, normalized read coverage was compared with the same location in the negative control and used to ascertain a targets validity. Accounting for potential biases and read duplication generated during PCR construction of CLEAVE-Seq Illumina compatible libraries (Supplementary Fig. 2A step M) and those introduced by Illumina sequencing, genuine biochemically cleaved sites were defined as having a normalized read coverage of at least 5 with at least 5-fold excess coverage over the control.

#### CLEAVE-Seq false negative and positive rates

The rate of false discovery and missed identification of genuinely cleaved target sites were calculated by spiking artificial read coverage into a control dataset. In total, 500 computationally predicted targets were randomly selected and artificial paired end reads (150 bp in length) initiating within the expected window of cleavage were generated using the art_illumina tool^76^. A read coverage of 25 was spiked into the 500 randomly selected targets. Simulated reads were then spiked into the control sample’s FASTQ file and mapped back to the reference genome. After physical mapping, CLEAVE-Seq analysis was performed as described above using an independent control as a comparator and false positive and negative rates were calculated for M1, M2, and M3. Importantly, for all three targets, the false negative rate was 0% illustrating that all targets spiked into the control dataset were recovered. The false positive rates for M1, M2, and M3 were 1.4% (7 additional targets identified (+7 targets)), 19% (+95 targets), and 8.4% (+42 targets), respectively.

### Plant material

Publicly available maize (*Zea mays* L.) hybrid high type II (Hi-II) line was obtained from internal Corteva sources.

### Plasmids and reagents used for plant transformation

Cas9 and guide RNA vector construction was previously described in Svitashev *et al*.^77^. Plasmids containing cell division promoting transcription factors (maize ovule developmental protein 2 (ODP2) and maize Wuschel (WUS)), selectable and visible marker MOPAT-DSRED (a translational fusion of the bialaphos resistance gene, phosphinothricin-N-acetyl-transferase, and the red fluorescent protein DSRED) were previously described in Ananiev, E.V. *et al*.^78^. Ribonucleotprotein (RNP) complex formation was performed as described in Svitashev S. *et al*.^24^. Briefly, Cas9 protein and guide RNA molecules were mixed (in a 1:2 molar ratio, respectively) in 1x NEB Buffer 3 with 1 μl of RNA inhibitor (Ribo Guard^TM^, Epicentre, USA) in a total volume of 20 μl and incubated at room temperature for at least 15 min.

### Maize transformation

Biolistic-mediated delivery of plasmid vectors containing Ubiquitin promoter-regulated Cas9, maize U6 polymerase III promoter-regulated gRNA, Ubiquitin promoter-regulated ODP2, maize IN2 promoter-regulated WUS, and Ubiquitin promoter-regulated selectable and visible marker, *MOPAT-DSRED* fusion, to maize immature embryos was performed as previously described in Svitashev *et al*.^77^. The particle delivery of matrix comprising the RNPs complemented with plasmids containing the Ubiquitin promoter-regulated ODP2, maize IN2 promoter-regulated WUS, and Ubiquitin promoter-regulated selectable and visible marker, *MOPAT-DSRED* fusion, were delivered into maize embryo cells as described in Svitashev, S. *et al*.^24^. Post-bombardment culture, selection, and plant regeneration were performed as previously described in Gordon-Kamm, W. *et al*.^79^. *A. tumefaciens* transformation vectors containing Ubiquitin promoter-regulated Cas9, maize U6 polymerase III promoter-regulated gRNA, and visible marker, *MOPAT-DSRED* fusion, were introduced into maize followed the detailed protocol previously described in Gao, H.R. *et al*.^80^. Regenerated plantlets were moved to soil, where they were sampled (7 mm leaf punch per plant) and grown to maturity in greenhouse conditions. In total 390 plants were analyzed using MIPs and each treatment contained 26–117 plants.

### Target site monitoring in plants with Molecular Inversion Probes

Molecular inversion probes (MIP)^81,82^ assays were designed by analyzing a 100 nt window surrounding the target sites of interest identified via *in silico* and biochemical methods. Targeting arms flanking the region of interest were selected based on the following assay criteria: arm length of 17–28 nt, distance between 5′ and 3′ targeting arms of 1–70 nt and predicted melting temperature of 68–72 °C. Following design, targeting arms for each assay were linked by a common backbone sequence 30–50 nt in length and ordered as individual oligos with a 5′ phosphorylation. The individual 250 µM MIPs oligos were pooled in equal volumes to generate a 250 uM assay pool.

MIPs targeting and sequencing pool creation was accomplished via a four-step process: hybridization, circularization, exonuclease digestion and indexing/amplification. Briefly, hybridization reactions were prepared by combining 250 ng of DNA with 1.25 µl ampligase buffer (Epicentre), 0.5 µl 1 M blocking oligo, a volume of MIPs assay pool that resulted in a DNA:MIPs ratio of 500:1 to 5000:1 depending on panel size, and water to a final reaction volume of 12.5 µl. Reactions were denatured for 10 min at 95 °C followed by 30 min incubation at 60 °C in a thermocycler with heated lid. Following incubation hybridized MIPs were recircularized by addition of 0.2 µl of 10x Ampligase buffer, 1 ul 2 U/µl HF Phusion polymerase (New England Biolabs), 0.25 ul 100 U/µl Ampligase enzyme (Epicentre) and 0.55 µl 0.25 mM dNTP mix (New England Biolabs) to the completed hybridization reaction, while the reaction was maintained at 60 °C. The final circularization reaction was mixed gently, sealed and incubated at 60 °C for 16–18 hours. Following circularization, incubation reactions were collected by centrifugation, incubated for 1 min at 37 °C and stored at 4 °C until exonuclease digestion.

Exonuclease digestion to remove linear genomic DNA and un-circularized probes was performed by adding 1 µl of 20 U/ul Exo I and 1 µl of 100 U/µl Exo III (New England Biolabs, USA) to the circularized MIP reaction from the previous step. Reactions were incubated in a thermocycler for 15 min at 37 °C followed by 2 min inactivation at 95 °C. Following digestion, targeted sequences were indexed and amplified by adding 12.5 µl of 2X iProof Master mix (Biorad), 0.125 µl 100 µM universal backbone forward primer, 0.125 µl 100 µM indexed backbone reverse primer, and 9.8 µl water. Reactions were denatured at 98 °C for 2 min and amplified by 25 cycles of 98 °C for 10 seconds, 60 °C for 30 seconds, 72 °C for 60 seconds. Resulting indexed amplicons were pooled and purified by a 1:1 Ampure XP cleanup according to manufacturer’s recommendations (Beckman Coulter Inc., USA). Purified amplicon pools were sequenced via manufactures recommendations with custom primers to the backbone sequence on Illumina MiSeq sequencers, generating 100 nt paired end reads.

### Analysis of targeted loci sequence

Sequencing reads were deconvoluted into sample bins by index sequence. Per sample reads were analyzed by identifying reads that belong to a specific MIPs assay via the 5′ and 3′ targeting arm. Reads were aligned via Bowtie v2 to the wildtype reference used in design of the assay arms. Differences between the reference sequences were identified by mismatches in alignment and reported via SAM Tools.

## Supplementary information

Supplementary Table 1,2,3,5,6,7

Supplementary Table 4

Supplementary Figure 1, 2

SI

## Data Availability

The data supporting the findings of this study are available within the paper and its Supplementary Information Files. Sequencing data have been deposited in the National Center for Biotechnology Information Sequence Read Archive database under accession code PRJNA526862.
