# Supplementary material for: CRISPR-Cas9 Editing in Maize: Systematic Evaluation of Off-target Activity and Its Relevance in Crop Improvement
Source: Sci Rep. 2019 Apr 30;9:6729. doi: 10.1038/s41598-019-43141-6 (PMC6491584; doi:10.1038/s41598-019-43141-6)
Supplement: Supplementary file 1 — Supplementary Table 1,2,3,5,6,7 [file 41598_2019_43141_MOESM1_ESM.pdf]

Supplementary Table 1 . A shortlist of computationally predicted M1 sites. On-target site is shown on the top. DNA and RNA Bulges are shown as gray and black boxes, respectively. Mismatches are shown in color boxes.

Site	Computational prediction		Sequence																								Genomic Location	Avg CLEAVE-Seq Reads
	Mismatch	*Bulge*	21	20	19	18	17	16	15	14	13	12	11	10	9	8	7	6	5	4	3	2	1	0	-1	-2		
	0	0	G	C	A	C	G	T	A	C	G	T	C	A	C	C	A	T	C	C	C	G	C	C	G	G	Chr1:14702765-14702788	200.78
1	2	1											G							A				C			Chr7:16502824-16502847	0.78
2	2	1											A							G				C			Chr3:12444708-12444731	0.00
3	2	1											G							G				C			Chr1:54992863-54992886	0.00
4	2	1											A				G							C			Chr8:38361070-38361093	1.41
5	2	1											G							G				C			Chr8:73407823-73407846	0.00
6	2	1											G							G				C			Chr10:26835370-26835393	1.46
7	2	1											G							G				C			Chr10:26867320-26867343	0.00
8	2	1											G							G				C			Chr10:142898890-142898913	0.21
9	2	1			T							G												C			Chr7:5753958-5753981	3.02
10	2	1							C						a							A		A			Chr7:156486399-156486422	3.02
11	2	1											G							G				C			Chr5:182622499-182622522	0.00
12	2	1											G							G				C			Chr1:77630220-77630243	0.00
13	2	1											A					G						C			Chr1:84645608-84645631	0.00
14	2	1								t							C					C		G	A		Chr3:56838689-56838712	0.00
15	2	1			T									C										C	A		Chr2:29281983-29282006	0.63

Supplementary Table 2. CLEAVE-seq data and A shortlist of computationally predicted M3 sites. On-target site is shown on the top. DNA and RNA Bulges are shown as gray and black boxes, respectively. Mismatches are shown in color boxes.

Site	Computational prediction		Sequence																							Genomic Location	Avg CLEAVE-Seq Reads
	Mismatch	*Bulge*	20	19	18	17	16	15	14	13	12	11	10	9	8	7	6	5	4	3	2	1	0	-1	-2		
	0	0	G	C	G	G	A	G	A	C	T	A	A	G	T	G	G	C	T	G	T	A	G	G	G	Chr2:4233941-4233964	164.19
1	2	1									A											G	T			Chr2:172786227-172786250	0.00
2	2	1															T	A					A			Chr7:43812401-43812424	2.46
3	2	1							C													G	A			Chr2:120060642-120060665	0.00
4	2	1									G				c						A		G	A		Chr5:93791118-93791140	0.00
5	2	1									G				c						A		G	A		Chr3:196197068-196197090	0.00
6	2	1							G				C										G	A		Chr5:168532712-168532734	0.00
7	2	1									G				c						A		G	A		Chr7:115695254-115695276	0.00
8	2	1									G				c			A					G	A		Chr7:62777273-62777295	0.00
9	2	1								G	C												G	A		Chr1:147217001-147217023	0.00
10	2	1																T				T	G	A		Chr1:286055591-286055613	0.00
11	2	1									G				c						A		G	A		Chr8:86546577-86546599	0.00
12	2	1									G				c						A		G	A		Chr1:76398359-76398381	0.00
13	2	1									G				c		A						G	A		Chr10:64318847-64318869	0.00
14	2	1									G				c						A		G	A		Chr8:86529185-86529207	0.00
15	2	1									G				c						A		G	A		Chr5:93800999-93801021	0.00

Supplementary Table 3. CLEAVE-seq data and a shortlist of computationally predicted M2 sites. On-target site is shown on the top. DNA and RNA Bulges are shown as gray and black boxes, respectively. Mismatches are shown in color boxes.

Site	Computational prediction		Sequence																							Genomic Location	Avg CLEAVE-Seq Reads
	Mismatch	*Bulge*	20	19	18	17	16	15	14	13	12	11	10	9	8	7	6	5	4	3	2	1	0	-1	-2		
1	0	0	G	G	C	C	G	A	G	G	T	C	G	A	C	T	A	C	C	G	G	C	C	G	G	Chr9:143631773-143631796	227.16
2	2	1			c		C		T														A			Chr2:37363776-37363799	14.68
3	1	1		■							G												A			Chr3:200475375-200475398	48.43
4	2	1		■				C			G												A			Chr10:5286571-5286594	111.10
5	2	1			G							G				g							C			Chr5:139807793-139807816	3.50
6	2	1		■							G			G									A			Chr5:196093089-196093112	0.00
7	1	1				A									■								G			Chr6:166308906-166308929	12.21
8	2	1			A						■				T								G			Chr5:219494224-219494247	0.00
9	2	1		T				■								C							A			Chr6:157449386-157449409	1.00
10	1	1											■			C							C			Chr3:214572069-214572092	0.00
11	2	1					■							T		C							G			Chr6:9938751-993874	0.00
12	2	1					■							T		C							G			Chr9:3475286-3475309	0.00
13	2	1						■						T		C							G			Chr3:218090730-218090753	0.00
14	2	1						■						T		C							G			Chr8:80758362-80758385	0.00
15	2	1						■						T		C							G			Chr5:16787335-16787358	0.00
16	2	1						■						T		C							G			Chr5:174188899-174188922	0.00
17	2	1						■						T		C							G			Chr4:17679359-17679382	0.00
18	2	1						■						T		C							G			Chr3:174183068-174183091	0.00
19	2	1						■						T		C							G			Chr9:16372961-16372984	0.00
20	2	1				G								G			■						T			Chr6:35449294-35449317	0.00
21	2	1	C												g		T						C			Chr2:48116260-48116283	0.00
22	2	1	C												g		T						C			Chr10:120428313-120428336	0.00
23	2	1					■							T					T				G			Chr9:78374914-78374937	0.00
24	2	1						■						T					T				G			Chr6:40580933-40580956	0.00
25	2	1						■						T					T				G			Chr1:57503045-57503068	0.00
26	2	1						■						T					T				G			Chr3:216792332-216792355	0.00
27	2	1						■						T					T				G			Chr8:138192658-138192681	0.00
28	2	1						■						T					T				G			Chr10:117146380-117146403	0.00
29	2	1				■										C			G				A			Chr4:184633059-184633082	0.00
30	2	1					■									C			G				G			Chr8:123353866-123353889	0.00
31	2	1						■								C			G				G			Chr4:46962972-46962995	0.00
32	2	1						■								C			G				G			Chr4:46981868-46981891	0.00
33	2	1						■								C			G				G			Chr1:130948589-130948612	0.00
34	2	1						■								C			G				G			Chr8:101460540-101460563	0.00
35	2	1						■								C			G				G			Chr10:38760703-38760726	0.00
36	2	1						■								C			G				G			Chr10:38770324-38770347	0.00
37	2	1						■								C			G				A			Chr5:218532749-218532772	0.00
38	2	1						■								C			G				A			Chr5:218542334-218542357	0.00
39	2	1						■								C			T				G			Chr10:83019302-83019325	0.00
40	2	1												C			■		A				T			Chr3:5743937-5743960	0.00
41	2	1		■														G	G				G			Chr9:63992902-63992925	0.00
42	2	1							T												■		G			Chr9:57340264-57340287	0.00
43	2	1				■								C							C		T			Chr4:146163129-146163152	0.00
44	2	1				■								C							C		T			Chr2:213478153-213478176	0.00
45	2	1				■										C			A				A			Chr5:223273918-223273940	2.38
46	2	1				■						G				A							C	A		Chr1:137725782-137725804	0.00
47	2	1					■							T		C							G	A		Chr1:159151911-159151933	0.00
48	2	1					■							T		C							G	A		Chr1:199634954-199634976	0.00
49	2	1							C		A					■							G	A		Chr1:232879291-232879313	0.00
50	2	0															T		G				G	A		Chr1:304501362-304501384	0.00
51	2	0												C								A	C	A		Chr2:75055786-75055808	0.00
52	2	0															T		G				G	A		Chr3:145198993-145199015	0.00
53	2	1				G							c			C							G	A		Chr4:79878295-79878317	0.00
54	2	1							C										t		■		A	A		Chr5:2689067-2689089	0.00
55	2	1				G							c			C							G	A		Chr5:110101791-110101813	0.00
56	2	1				G							c			C							G	A		Chr5:110111414-110111436	0.00
57	2	1				■										G	G						T	A		Chr5:126305549-126305571	0.00
58	2	0															T		G				G	A		Chr6:125674373-125674395	0.00
59	2	1				■					C		A										G	A		Chr7:124810038-124810060	39.49
60	2	1				G							C		T								G	A		Chr8:15761268-15761290	0.00
61	2	1				G							C		T								G	A		Chr8:15770931-15770953	0.00
62	2	1				■										G	G						T	A		Chr8:50000473-50000495	0.00
63	2	1						■								C	G						A	A		Chr8:139620037-139620059	0.00
64	2	1					C						C	C									A	A		Chr9:22064095-22064117	0.00
65	2	1			T	■										G							T	A		Chr9:59915238-59915260	0.00
66	2	0															T		G				G	A		Chr9:159463178-159463200	0.00

Supplementary Table 5. CLEAVE-seq data and a shortlist of computationally predicted high ranked M2 sites with low number of mismatches and bulges. On-target site is shown on the top. RNA Bulges are shown as boxes, respectively. Mismatches are shown in color boxes.

Site	Computational prediction		CLEAVE-Seq reads	Sequence																							Genomic Location
	Mismatch	***Bulge***	***100nM***	**20**	**19**	**18**	**17**	**16**	**15**	**14**	**13**	**12**	**11**	**10**	**9**	**8**	**7**	**6**	**5**	**4**	**3**	**2**	**1**	**0**	**-1**	**-2**	
1	0	0	227	G	G	C	C	G	A	G	G	T	C	G	A	C	T	A	C	C	G	G	C	C	G	G	Chr9:143631773-143631795
2	1	1	0														C							C			Chr3:214572069-214572090
3	2	1	0												T					T				G			Chr3:216792332-216792353
4	2	1	0									G			G									A			Chr5:196093089-196093111
5	2	1	0			A										T								G			Chr5:219494224-219494245
6	2	2	0		T															T				T			Chr7:116040382-116040402
7	3	0	0		C							G							G					A			Chr4:173200547-173200569

Supplementary Table 6 . CLEAVE-seq data and a shortlist of M2 sites validated using MIPS. On-target site is shown on the top. DNA and RNA Bulges are shown as gray and black boxes, respectively. Mismatches are shown in color boxes.

Site	Computational prediction		Sequence																							Genomic Location	Predicted by Cas-OFFinder	Identified in CLEAVE-Seq	Avg Normalized CLEAVE-Seq Read Count
	Mismatch	*Bulge*	20	19	18	17	16	15	14	13	12	11	10	9	8	7	6	5	4	3	2	1	0	-1	-2				
1	0	0	G	G	C	C	G	A	G	G	T	C	G	A	C	T	A	C	C	G	G	C	C	G	G	Chr9:143631773-143631795	Yes	Yes	227.16
2	1	1		■							G												A			Chr3:200475375-200475397	Yes	Yes	48.43
3	1	1				A									■								G			Chr6:166308906-166308927	Yes	Yes	12.21
4	1	1											■										C			Chr3:214572069-214572090	Yes	No	0.00
5	2	1		■				C			G												A			Chr10:5286571-5286593	Yes	Yes	111.10
6	2	1		T				■								C							A			Chr6:157449386-157449407	Yes	Yes	1.00
7	2	1						■						T					T				G			Chr3:216792332-216792353	Yes	No	0.00
8	2	1		■							G			G									A			Chr5:196093089-196093111	Yes	No	0.00
9	2	1			A						■				T								G			Chr5:219494224-219494245	Yes	No	0.00
10	2	2	T		■	■		G															A			Chr5:37860732-37860754	Yes	Yes	529.17
11	2	2		T				■	■										T				T			Chr7:116040382-116040402	Yes	No	0.00
12	3	0		C							G							G					A			Chr4:173200547-173200569	Yes	No	0.00
13	3	1		■					C		G			G									A			Chr1:5942859-5942881	Yes	Yes	4.41
14	3	1					A				g									A	C		G			Chr10:39633639-39633661	Yes	Yes	1.00
15	3	1				A					A		■						A				A			Chr2:103727824-103727845	Yes	No	0.00
16	3	1						c					A		T					C			G			Chr3:54133373-54133396	Yes	No	0.00
17	3	1		■								A		G							A		A			Chr6:28373069-28373091	Yes	No	0.00
18	3	2		A		g	T			A													G			Chr7:173232468-173232490	Yes	Yes	11.29
19	4	0		C							G		A			C							G			Chr3:54313532-54313554	Yes	No	0.00
20	4	1		■	A											A			G	A			G			Chr1:168558804-168558826	Yes	Yes	3.31
21	4	1		■	A											A			A	A			G			Chr10:84440789-84440811	Yes	Yes	16.42
22	4	1	A	■				C								C				C			C			Chr4:233088295-233088317	Yes	Yes	1.00
23	4	1				t		G			A			T			T						T			Chr5:6293703-6293726	Yes	Yes	1.55
24	4	1		■	T						C		C								A		G			Chr5:82350877-82350899	Yes	Yes	2.93
25	4	1	C		G								A	g				A					C			Chr1:141609726-141609748	Yes	No	0.00
26	4	1	T		G								A	g				A					C			Chr1:141619342-141619364	Yes	No	0.00
27	4	1		■	A											A			G	A			G			Chr1:89052551-89052573	Yes	No	0.00
28	4	1	A	■								A			G					C			T			Chr10:51974012-51974034	Yes	No	0.00
29	4	1		■									A			C				A	C		A			Chr2:19426157-19426179	Yes	No	0.00
30	4	1	A	■				C				T								C			C			Chr2:36174928-36174950	Yes	No	0.00
31	4	1		■	A											A			G	A			G			Chr2:81561460-81561482	Yes	No	0.00
32	4	1		■	A											A			G	A			G			Chr3:199671651-199671673	Yes	No	0.00
33	4	1		■	A			G											G	A			G			Chr3:222638047-222638069	Yes	No	0.00
34	4	1		■		G				C			A				G						G			Chr5:40664611-40664633	Yes	No	0.00
35	4	1	A						A	a									A	T			T			Chr6:114459472-114459495	Yes	No	0.00
36	4	1	C	■							A			C						T			A			Chr7:15742994-15743016	Yes	No	0.00
37	4	1	A		G						a					A				A			A			Chr7:159564951-159564973	Yes	No	0.00
38	4	2		A		g		G											G	A			G			Chr3:168788394-168788416	Yes	Yes	2.48
39	4	2	T				tc				A						T						T			Chr7:79326098-79326121	Yes	No	0.00
40	5	0		C	G					C	G									T			G			Chr2:80845032-80845054	Yes	Yes	10.71
41	5	0	T		A					A						A				A			G			Chr2:197687616-197687638	Yes	Yes	8.58
42	5	0			A	A						G		C				G					T			Chr1:124111388-124111410	Yes	Yes	2.48
43	5	0			A	A						G		C				G					T			Chr0:4325171-4325193	Yes	Yes	2.48
44	5	0		C			A							G		A	T						G			Chr1:101163004-101163026	Yes	No	0.00
45	5	0			A	A						G		C				G					T			Chr1:104009978-104010000	Yes	No	0.00
46	5	0	T		A											A			G	A			T			Chr1:116346261-116346283	Yes	No	0.00
47	5	0			A	A						G		C				G					T			Chr1:203782886-203782908	Yes	No	0.00
48	5	0			A	A						G		C				G					T			Chr1:92500265-92500287	Yes	No	0.00
49	5	0		A							G	G			A					C			T			Chr10:4860335-4860357	Yes	No	0.00
50	5	0			A	A						G		C				G					T			Chr10:88386224-88386246	Yes	No	0.00
51	5	0			A	A						G		C				G					T			Chr2:239879667-239879689	Yes	No	0.00
52	5	0			A	A						G		C				G					T			Chr2:38715655-38715677	Yes	No	0.00
53	5	0			A	A						G		C				G					T			Chr2:75127397-75127419	Yes	No	0.00
54	5	0			A	A						G		C				G					T			Chr3:107792653-107792675	Yes	No	0.00
55	5	0			A	A						G		C				G					T			Chr3:111193547-111193569	Yes	No	0.00
56	5	0		C			A				A					A	T						G			Chr4:131687629-131687651	Yes	No	0.00
57	5	0		C			A	G								A	T						G			Chr4:96932584-96932606	Yes	No	0.00
58	5	0			A	A						G		C				G					T			Chr5:29439883-29439905	Yes	No	0.00
59	5	0			A	A						G		C				G					T			Chr5:39014962-39014984	Yes	No	0.00
60	5	0	C		A			G											G	A			A			Chr5:47203908-47203930	Yes	No	0.00
61	5	0			A	A						G		C				G					T			Chr5:60919606-60919628	Yes	No	0.00
62	5	0			A	A						G		C				G					T			Chr5:75784576-75784598	Yes	No	0.00
63	5	0			A	A						G		C				G					T			Chr7:34966766-34966788	Yes	No	0.00
64	5	0			A	A						G		C				G					T			Chr7:8090624-8090646	Yes	No	0.00
65	5	0			A	A						G		C				G					T			Chr8:160311153-160311175	Yes	No	0.00
66	5	0			A	A						G		C				G					T			Chr8:25573743-25573765	Yes	No	0.00
67	5	0			A	A						G		C				G					T			Chr8:56845265-56845287	Yes	No	0.00
68	5	1	T		A											■		A			A	G	C			Chr2:45333382-45333404	Yes	Yes	0.00
69	5	1	A	■				T			A									T	T		C			Chr1:247722181-247722203	Yes	Yes	4.68
70	5	1		■	A	A						G		C				G					T			Chr8:78781894-78781916	Yes	Yes	1.33
71	5	1	C		G	T					g	A						A					C			Chr1:124001644-124001671	Yes	No	0.00
72	5	1		■	A	A						G		C				G					T			Chr1:175146350-175146372	Yes	No	0.00
73	5	1	T		A			G								G			■			G	C			Chr1:180655201-180655223	Yes	No	0.00
74	5	1		■	A	A						G		C				G					T			Chr1:21083695-21083717	Yes	No	0.00
75	5	1	T		A											■		A			A	G	C			Chr1:5273647-5273669	Yes	No	0.00
76	5	1	C	■			A							G		A	T						G			Chr1:73592041-73592063	Yes	No	0.00
77	5	1				G	A	G				A						A				c	G			Chr10:37395498-37395520	Yes	No	0.00
78	5	1		■	A	A						G		C				G					T			Chr10:75750456-75750478	Yes	No	0.00
79	5	1	A	■			T											T		A	A		C			Chr2:81171011-81171033	Yes	No	0.00
80	5	1		■	A	A						G		C				G					T			Chr2:82344809-82344831	Yes	No	0.00
81	5	1		■	A	A						G		C				G					T			Chr2:84402863-84402885	Yes	No	0.00
82	5	1		■	A	A						G		C				G					T			Chr3:16052720-16052742	Yes	No	0.00
83	5	1	T		A											■		A			A	G	C			Chr4:26448021-26448043	Yes	No	0.00
84	5	1		C							C		A			C			■			G	C			Chr4:35475536-35475558	Yes	No	0.00
85	5	1		■	A	A						G		C				G					T			Chr4:72754228-72754250	Yes	No	0.00
86	5	1				G	A	G				A						A					IcG			Chr5:112910962-112910984	Yes	No	0.00
87	5	1		■	A	A						G		C				G					T			Chr5:141546929-141546951	Yes	No	0.00
88	5	1		■	A	A						G		C				G					T			Chr5:189221660-189221682	Yes	No	0.00
89	5	1	T		A											■		A			A	G	C			Chr5:205487171-205487193	Yes	No	0.00
90	5	1	A	■					A	A	A									A			A			Chr5:41228760-41228782	Yes	No	0.00
91	5	1	C	■							C		A				C		A				T			Chr6:167702031-167702053	Yes	No	0.00
92	5	1		■	A	A						G		C				G					T			Chr6:82650049-82650071	Yes	No	0.00
93	5	1		■	A	A						G		C				G					T			Chr8:146121378-146121400	Yes	No	0.00
94	5	1		■		A	A				G		A								T		T			Chr8:157442665-157442687	Yes	No	0.00
95	5	1		■	A	A						G		C				G					T			Chr8:79394412-79394434	Yes	No	0.00
96	5	2		C		■	■							C	G	G					T		C			Chr7:124810037-124810059	Yes	No	0.00

Supplementary Table 7. MIPS genotyping of 390 T_0 maize plants generated from three different target sites and delivery methods. Any allele, mutant or WT represent >5% of total reads to be called an allele. Percent mutant alleles indicate total mutant alleles/total alleles from plants analyzed. Mosaic plants contain >2 alleles. Homozygous mutant plants contain single mutant allele with >90% reads

Target Site	Transformation Method	Number of Plants Analyzed	Percent Mutant Alleles	Mosaic Plants	Homozygous WT Plants	Homozygous Mutant Plants	Heterozygous Mut/Mut Plants	Heterozygous WT/Mut Plants
M1	Agro	26	61.30%	0.00%	46.20%	34.60%	19.20%	0.00%
M2	DNA-particle gun	112	95.90%	8.10%	5.20%	34.90%	45.90%	5.80%
	Agro	117	99.00%	9.30%	0.00%	49.20%	39.80%	1.70%
	RNP-particle gun	94	33.30%	0.00%	61.50%	15.40%	15.40%	7.70%
M3	Agro	41	94.30%	5.60%	8.30%	55.60%	30.60%	0.00%
