# Supplementary material for: CRISPR-Cas9 Editing in Maize: Systematic Evaluation of Off-target Activity and Its Relevance in Crop Improvement
Source: Sci Rep. 2019 Apr 30;9:6729. doi: 10.1038/s41598-019-43141-6 (PMC6491584; doi:10.1038/s41598-019-43141-6)
Supplement: Supplementary file 2 — Supplementary Table 4 [file 41598_2019_43141_MOESM2_ESM.pdf]

**Supplementary Table 4.** Comprehensive list of cleaved genomic target sites detected from M2 guide RNA using CLEAVE-Seq

| Location | Cut Site | CutSite_WRT_Target | MM_Type | DNA_Sequence | RNA_Sequence | Strand | Mismatches | Bulge Size | Normalized Read Count Rep 1 | Normalized Read Count Rep 2 | Avg CLEAVE-Seq Reads |
|---|---|---|---|---|---|---|---|---|---|---|---|
| Chr5:37860732-37860754 | 37860737 | 3 | RNA | GGCCGAGGTCGACTACCGGCNRG | tG--GgGGTCGACTACCGGCAGG | - | 2 | 2 | 469 | 589.33 | 529.17 |
| Chr9:143631773-143631795 | 143631790 | 3 | X | GGCCGAGGTCGACTACCGGCNRG | GGCCGAGGTCGACTACCGGCCGG | + | 0 | 0 | 234 | 220.31 | 227.16 |
| Chr10:5286571-5286593 | 5286586 | 5 | RNA | GGCCGAGGTCGACTACCGGCNRG | G-CCGcGGgCGACTACCGGCAGG | + | 2 | 1 | 79 | 143.20 | 111.10 |
| Chr3:200475375-200475397 | 200475390 | 5 | RNA | GGCCGAGGTCGACTACCGGCNRG | G-CCGAGGgCGACTACCGGCAGG | + | 1 | 1 | 28 | 68.85 | 48.43 |
| Chr7:124810038-124810060 | 124810053 | 5 | RNA | GGCCGAGGTCGACTACCGGCNRG | GGC-GAGGcCaACTACCGGCGAG | + | 2 | 1 | 25 | 53.98 | 39.49 |
| Chr5:62435814-62435836 | 62435830 | 4 | RNA | GGCCGAGGTCGACTACCGGCNRG | G-aCGAGaTCGACaACaaGCGGG | + | 5 | 1 | 21 | 31.94 | 26.47 |
| Chr5:144051759-144051781 | 144051776 | 3 | DNA | GGCC-GAGGTCGACTACCGGCNRG | GGaCTGAaGggGAaTACCGGCTGG | + | 5 | 1 | 26 | 12.67 | 19.34 |
| Chr7:61544299-61544321 | 61544305 | 4 | DNA | GGC-CGAGGTCGACTACCGGCNRG | GcCTCGAGGaCcACTtCCGGCTGG | - | 4 | 1 | 13 | 24.78 | 18.89 |
| Chr2:152062830-152062852 | 152062846 | 4 | RNA | GGCCGAGGTCGACTACCGGCNRG | GaCaG-GGTCGACaACaaGCGGG | + | 5 | 1 | 1 | 31.94 | 16.47 |
| Chr10:84440789-84440811 | 84440794 | 3 | RNA | GGCCGAGGTCGACTACCGGCNRG | G-aCGAGGTCGACaACaaGCGGG | - | 4 | 1 | 13 | 19.83 | 16.42 |
| Chr10:106127836-106127858 | 106127852 | 4 | RNA | GGCCGAGGTCGACTACCGGCNRG | GaCaG-GGTCGACaACaaGCGGG | + | 5 | 1 | 3 | 29.19 | 16.10 |
| Chr2:37363776-37363798 | 37363794 | 2 | DNA | GGCC-GAGGTCGACTACCGGCNRG | GGCCCcAtGTCGACTACCGGCAGG | + | 2 | 1 | 20 | 9.36 | 14.68 |
| Chr5:69134156-69134178 | 69134163 | 5 | RNA | GGCCGAGGTCGACTACCGGCNRG | GG--GgacaCGcCTACCGGCGGG | - | 5 | 2 | 26 | 0.00 | 13.00 |
| Chr2:6864100-6864122 | 6864116 | 4 | RNA | GGCCGAGGTCGACTACCGGCNRG | GGtCGgGGT-GAgTACCtGCGGG | + | 4 | 1 | 10 | 14.87 | 12.44 |
| Chr6:166308906-166308928 | 166308922 | 4 | RNA | GGCCGAGGTCGACTACCGGCNRG | GGCaGAGGTCGA-TACCGGCGGG | + | 1 | 1 | 20 | 4.41 | 12.21 |
| Chr3:33020604-33020626 | 33020609 | 3 | RNA | GGCCGAGGTCGACTACCGGCNRG | tGaCG-GGTCGACaACaaGCGGG | - | 5 | 1 | 2 | 22.03 | 12.02 |
| Chr1:259957715-259957737 | 259957732 | 3 | X | GGCCGAGGTCGACTACCGGCNRG | GGCCGAGcTCtACcAgCGGCGGG | + | 4 | 0 | 21 | 2.20 | 11.60 |
| Chr7:173232468-173232490 | 173232473 | 3 | RNA | GGCCGAGGTCGACTACCGGCNRG | G-aCtAGaTCGACTACCGGCGGG | - | 3 | 1 | 11 | 11.57 | 11.29 |
| Chr2:80845034-80845056 | 80845049 | 5 | RNA | GGCCGAGGTCGACTACCGGCNRG | G--CGAGcgCGACTACCtGCGGG | + | 3 | 2 | 17 | 4.41 | 10.71 |
| Chr10:101313469-101313491 | 101313487 | 2 | RNA | GGCCGAGGTCGACTACCGGCNRG | GGgCGAG--CGctgACaGGCGGG | + | 5 | 2 | 3 | 18.18 | 10.59 |
| Chr7:119054025-119054047 | 119054032 | 5 | RNA | GGCCGAGGTCGACTACCGGCNRG | GGCgcAGccaGACT-CCGGCAAG | - | 5 | 1 | 7 | 13.77 | 10.39 |
| Chr3:232791371-232791393 | 232791387 | 4 | RNA | GGCCGAGGTCGACTACCGGCNRG | G-aCGgGGTCGACaACgaGCGGG | + | 5 | 1 | 4 | 16.52 | 10.26 |
| Chr5:102819446-102819468 | 102819453 | 5 | RNA | GGCCGAGGTCGACTACCGGCNRG | GGC-GAGtaCGAactCCGGCGAG | - | 5 | 1 | 2 | 17.62 | 9.81 |
| Chr1:17488618-17488640 | 17488625 | 5 | DNA | GGC-CGAGGTCGACTACCGGCNRG | GcCTCGAGGaCGtCTtCCGGCTGG | - | 4 | 1 | 19 | 0.00 | 9.50 |
| Chr4:245455294-245455316 | 245455298 | 2 | DNA | GGCCGAGGTCGACTAC-CGGCNRG | GGCCGAGGggGAaTcCGCGGtCGG | - | 5 | 1 | 0 | 18.73 | 9.37 |
| Chr4:240239220-240239242 | 240239225 | 3 | RNA | GGCCGAGGTCGACTACCGGCNRG | G-aCGgGGTCGACgACgaGCGGG | - | 5 | 1 | 11 | 7.71 | 9.36 |
| Chr2:44981747-44981769 | 44981754 | 5 | RNA | GGCCGAGGTCGACTACCGGCNRG | aGCCGAtaTCaACTACC--gGAG | - | 5 | 2 | 18 | 0.00 | 9.00 |
| Chr1:53187763-53187785 | 53187770 | 5 | RNA | GGCCGAGGTCGACTACCGGCNRG | GGCaGAtcTgGgCTA--GGCAAG | - | 5 | 2 | 18 | 0.00 | 9.00 |
| Chr7:82662153-82662175 | 82662160 | 5 | RNA | GGCCGAGGTCGACTACCGGCNRG | GttCGgGGaCGtCT-CCGGCGAG | - | 5 | 1 | 18 | 0.00 | 9.00 |
| Chr7:102664667-102664689 | 102664684 | 3 | RNA | GGCCGAGGTCGACTACCGGCNRG | GaCCGAGGT-GACgACaaGaAGG | + | 5 | 1 | 18 | 0.00 | 9.00 |
| Chr9:54983896-54983918 | 54983902 | 4 | RNA | GGCCGAGGTCGACTACCGGCNRG | GGC--AGGcCaACctCCGGCCGG | - | 4 | 2 | 0 | 17.62 | 8.81 |
| Chr3:152958354-152958376 | 152958369 | 5 | RNA | GGCCGAGGTCGACTACCGGCNRG | cGgCGAacTtG--TACCGGCCGG | + | 5 | 2 | 0 | 17.62 | 8.81 |
| Chr9:70745680-70745702 | 70745697 | 3 | DNA | GGC-CGAGGTCGACTACCGGCNRG | GcCTCGAGGaCGtCTtCCGGCTGG | + | 4 | 1 | 2 | 15.42 | 8.71 |
| Chr2:197687617-197687639 | 197687633 | 4 | RNA | GGCCGAGGTCGACTACCGGCNRG | G-aCGAGaTCGACaACCaGCGGG | + | 4 | 1 | 10 | 7.16 | 8.58 |
| Chr5:134414881-134414903 | 134414887 | 4 | RNA | GGCCGAGGTCGACTACCGGCNRG | GGaCGAGGTCGACcAaat-CCGG | - | 5 | 1 | 0 | 17.07 | 8.54 |
| Chr1:25488084-25488106 | 25488101 | 3 | DNA | GG-CCGAGGTCGACTACCGGCNRG | GGTCCGAGtTCaAgaACCaGCAGG | + | 5 | 1 | 0 | 17.07 | 8.54 |
| Chr4:100282684-100282706 | 100282690 | 4 | X | GGCCGAGGTCGACTACCGGCNRG | GGaaGAGGTgGcCTAgCGGCTGG | - | 5 | 0 | 0 | 17.07 | 8.54 |
| Chr5:99134781-99134803 | 99134798 | 3 | RNA | GGCCGAGGTCGACTACCGGCNRG | GGCtGAGaTCaA--ACCGtgCAG | + | 5 | 2 | 17 | 0.00 | 8.50 |
| Chr4:220583733-220583755 | 220583749 | 4 | X | GGCCGAGGTCGACTACCGGCNRG | GGCCGAGGTCcAtcACCtGCTGG | + | 4 | 0 | 3 | 13.77 | 8.39 |
| Chr2:56588382-56588404 | 56588398 | 4 | RNA | GGCCGAGGTCGACTACCGGCNRG | G-aCGgGGTCGACgACgaGCGGG | + | 5 | 1 | 0 | 16.52 | 8.26 |
| Chr1:228584553-228584575 | 228584569 | 4 | RNA | GGCCGAGGTCGACTACCGGCNRG | G-aCGgGGTCGACaACgaGCGGG | + | 5 | 1 | 5 | 11.02 | 8.01 |
| Chr2:59843341-59843363 | 59843348 | 5 | DNA | GGC--CGAGGTCGACTACCGGCNRG | GGCGTCGAGGaCctCTACgtGCCGG | - | 5 | 2 | 16 | 0.00 | 8.00 |
| Chr5:90344751-90344773 | 90344768 | 3 | DNA | GGCCGAGGTCGACTAC-CGGCNRG | GGCtGAGGTCGAggcCGaGGCGAG | + | 5 | 1 | 16 | 0.00 | 8.00 |
| Chr2:146608744-146608766 | 146608760 | 4 | RNA | GGCCGAGGTCGACTACCGGCNRG | GGCtaAGcTgGACTA--GGtTGG | + | 5 | 2 | 0 | 15.97 | 7.99 |
| Chr2:52422323-52422345 | 52422330 | 5 | RNA | GGCCGAGGTCGACTACCGGCNRG | GGgCGAGc-CaACctCCGGCCGG | - | 5 | 1 | 0 | 15.97 | 7.99 |
| Chr7:19666173-19666195 | 19666188 | 5 | RNA | GGCCGAGGTCGACTACCGGCNRG | GGC--ttGcgGAtTACCGGCTGG | + | 5 | 2 | 6 | 9.91 | 7.96 |
| Chr2:187376830-187376852 | 187376848 | 2 | RNA | GGCCGAGGTCGACTACCGGCNRG | G--CacGGgCGAgaACCGGCCAG | + | 5 | 2 | 8 | 7.71 | 7.86 |
| Chr6:42796472-42796494 | 42796478 | 4 | RNA | GGCCGAGGTCGACTACCGGCNRG | G-CCGAaGTacACTgCCtGCTGG | - | 5 | 1 | 9 | 6.61 | 7.81 |
| Chr4:132703610-132703632 | 132703628 | 2 | RNA | GGCCGAGGTCGACTACCGGCNRG | cGCgaAGGTgGA--ACgGGCAGG | + | 5 | 2 | 0 | 15.42 | 7.71 |
| Chr6:82325146-82325168 | 82325152 | 4 | RNA | GGCCGAGGTCGACTACCGGCNRG | GGC--tcGcgGACTACCGaCTGG | - | 5 | 2 | 0 | 15.42 | 7.71 |
| Chr2:5765592-5765614 | 5765610 | 2 | RNA | GGCCGAGGTCGACTACCGGCNRG | GGC--AGGgtGAgTgCgGGCGGG | + | 5 | 2 | 0 | 15.42 | 7.71 |
| Chr8:102976801-102976823 | 102976819 | 2 | RNA | GGCCGAGGTCGACTACCGGCNRG | GGtCGtGGTCGAC--CgGaaCAG | + | 5 | 2 | 0 | 15.42 | 7.71 |
| Chr8:90266557-90266579 | 90266572 | 5 | DNA | GGC--CGAGGTCGACTACCGGCNRG | GGCCTCGgGGgCtACacCCGGCGGG | + | 5 | 2 | 0 | 15.42 | 7.71 |
| Chr1:29132869-29132891 | 29132874 | 3 | RNA | GGCCGAGGTCGACTACCGGCNRG | GaCaG-GGTCGACgACaaGCGGG | - | 5 | 1 | 0 | 15.42 | 7.71 |
| Chr3:147799357-147799379 | 147799362 | 3 | RNA | GGCCGAGGTCGACTACCGGCNRG | G-aCGgGGTCGACgACCaGCGGG | - | 4 | 1 | 0 | 15.42 | 7.71 |
| Chr6:133774274-133774296 | 133774291 | 3 | DNA | GGC-CGAGGTCGACTACCGGCNRG | GcCTCGAGGaCGtCTtCCGGCTGG | + | 4 | 1 | 11 | 4.41 | 7.71 |
| Chr7:101947601-101947623 | 101947608 | 5 | DNA | GGC-CGAGGTCGACTACCGGCNRG | GGCGCGgGcTCGACactCGGCGAG | - | 5 | 1 | 15 | 0.00 | 7.50 |
| Chr5:190861491-190861513 | 190861498 | 5 | RNA | GGCCGAGGTCGACTACCGGCNRG | GtCtGtGtTtGACT-CCGGCTGG | - | 5 | 1 | 15 | 0.00 | 7.50 |
| Chr8:137765681-137765703 | 137765688 | 5 | RNA | GGCCGAGGTCGACTACCGGCNRG | GtCCGAaaT--AaTACCGGaAAG | - | 5 | 2 | 15 | 0.00 | 7.50 |
| Chr7:108353924-108353946 | 108353941 | 3 | RNA | GGCCGAGGTCGACTACCGGCNRG | tGCCGAcGaCGACTgCt--CCAG | + | 5 | 2 | 15 | 0.00 | 7.50 |
| Chr3:2742482-2742504 | 2742489 | 5 | DNA | GGCCGAGGTCGACT--ACCGGCNRG | aGCaGAGGTgGACTCGAaCGGCAGG | - | 4 | 2 | 15 | 0.00 | 7.50 |
| Chr2:228111375-228111397 | 228111382 | 5 | RNA | GGCCGAGGTCGACTACCGGCNRG | GaCCGAGGgtGcCT-CgGGCTGG | - | 5 | 1 | 15 | 0.00 | 7.50 |
| Chr2:139725408-139725430 | 139725425 | 3 | RNA | GGCCGAGGTCGACTACCGGCNRG | GGCCatGGTCGACTAttc-CCAG | + | 5 | 1 | 0 | 14.87 | 7.44 |
| Chr7:42529888-42529910 | 42529903 | 5 | RNA | GGCCGAGGTCGACTACCGGCNRG | aGC--ttGTgGACTACCGGCTGG | + | 4 | 2 | 0 | 14.87 | 7.44 |
| Chr5:183185433-183185455 | 183185438 | 3 | RNA | GGCCGAGGTCGACTACCGGCNRG | GGCCGAcGTCG-CTAgCaGCAGG | - | 3 | 1 | 0 | 14.87 | 7.44 |
| Chr4:202796965-202796987 | 202796980 | 5 | RNA | GGCCGAGGTCGACTACCGGCNRG | ttCCc--GTCaACTACCGGCGGG | + | 4 | 2 | 1 | 13.77 | 7.39 |
| Chr4:142294326-142294348 | 142294343 | 3 | DNA | GGC-CGAGGTCGACTACCGGCNRG | GcCTCGAGGaCGtCTtCCGGCTGG | + | 4 | 1 | 4 | 10.46 | 7.23 |
| Chr5:167972398-167972420 | 167972402 | 2 | RNA | GGCCGAGGTCGACTACCGGCNRG | GGgCGA--TCGAgaACtaGCTGG | - | 5 | 2 | 0 | 14.32 | 7.16 |
| Chr2:238855968-238855990 | 238855984 | 4 | RNA | GGCCGAGGTCGACTACCGGCNRG | GGaCGAGGgCGAaT-CCGGCGGG | + | 3 | 1 | 0 | 14.32 | 7.16 |
| Chr8:167939418-167939440 | 167939422 | 2 | RNA | GGCCGAGGTCGACTACCGGCNRG | GG--GAGaTgaACTtCCGGaGAG | - | 5 | 2 | 0 | 14.32 | 7.16 |
| Chr4:178023389-178023411 | 178023407 | 2 | RNA | GGCCGAGGTCGACTACCGGCNRG | t--CGcGGTCGgCTAaCGGtGGG | + | 5 | 2 | 0 | 14.32 | 7.16 |
| Chr2:85683422-85683444 | 85683428 | 4 | RNA | GGCCGAGGTCGACTACCGGCNRG | GcCtcAGG-CGAgTACtGGCAGG | - | 5 | 1 | 0 | 14.32 | 7.16 |
| Chr1:189492830-189492852 | 189492835 | 3 | RNA | GGCCGAGGTCGACTACCGGCNRG | GGCtGgGGTaG--cACCGGgAGG | - | 5 | 2 | 0 | 14.32 | 7.16 |
| Chr5:209743451-209743473 | 209743468 | 3 | RNA | GGCCGAGGTCGACTACCGGCNRG | cGCCtcc--CaACTACCGGCAGG | + | 5 | 2 | 0 | 14.32 | 7.16 |
| Chr10:142955448-142955470 | 142955465 | 3 | RNA | GGCCGAGGTCGACTACCGGCNRG | GGCCGAGGcCGAg--CCctaGGG | + | 5 | 2 | 14 | 0.00 | 7.00 |
| Chr7:165223993-165224015 | 165224010 | 3 | DNA | G--GCCGAGGTCGACTACCGGCNRG | GTAGCCGAGGcCGAgctCgGGCGAG | + | 5 | 2 | 14 | 0.00 | 7.00 |
| Chr4:220064080-220064102 | 220064097 | 3 | RNA | GGCCGAGGTCGACTACCGGCNRG | tGCCGAtGTCtg-TACCtGCAGG | + | 5 | 1 | 14 | 0.00 | 7.00 |
| Chr8:93987706-93987728 | 93987723 | 3 | RNA | GGCCGAGGTCGACTACCGGCNRG | GGCCtcGGaCGgCgACC--CGGG | + | 5 | 2 | 14 | 0.00 | 7.00 |
| Chr7:125135211-125135233 | 125135216 | 3 | DNA | GGCCGAGGTCGAC-TACCGGCNRG | GGCCatGGcCaACGcACCGGCGGG | - | 5 | 1 | 5 | 8.81 | 6.91 |
| Chr6:77861766-77861788 | 77861782 | 4 | RNA | GGCCGAGGTCGACTACCGGCNRG | GGC--tcGcaGACTACCGcCTGG | + | 5 | 2 | 0 | 13.77 | 6.89 |
| Chr2:18015029-18015051 | 18015033 | 2 | RNA | GGCCGAGGTCGACTACCGGCNRG | GGCCGAGcTgGAgTAt-GGCCGG | - | 4 | 1 | 0 | 13.77 | 6.89 |
| Chr2:215736339-215736361 | 215736355 | 4 | RNA | GGCCGAGGTCGACTACCGGCNRG | GGC--tcGcgGACTACCGcCTGG | + | 5 | 2 | 0 | 13.77 | 6.89 |
| Chr10:148195714-148195736 | 148195720 | 4 | RNA | GGCCGAGGTCGACTACCGGCNRG | GGCgGAGGTaGAtT--CGagGAG | - | 5 | 2 | 0 | 13.77 | 6.89 |
| Chr10:77277596-77277618 | 77277613 | 3 | RNA | GGCCGAGGTCGACTACCGGCNRG | aGgCaAGtTCGAgTA--GGCAGG | + | 5 | 2 | 0 | 13.77 | 6.89 |
| Chr4:168992271-168992293 | 168992276 | 3 | RNA | GGCCGAGGTCGACTACCGGCNRG | aGC--AGGcCaACTACCGctTGG | - | 5 | 2 | 0 | 13.77 | 6.89 |
| Chr4:45623225-45623247 | 45623241 | 4 | RNA | GGCCGAGGTCGACTACCGGCNRG | G-aCGAGaTCGACaACgaGCGGG | + | 5 | 1 | 0 | 13.77 | 6.89 |
| Chr9:9413650-9413672 | 9413665 | 5 | RNA | GGCCGAGGTCGACTACCGGCNRG | aGCCaAGtTg--CTcCCGGCGGG | + | 5 | 2 | 0 | 13.77 | 6.89 |
| Chr9:49772148-49772170 | 49772155 | 5 | RNA | GGCCGAGGTCGACTACCGGCNRG | GGt-GAGGTCGgCcggCGGCTGG | - | 5 | 1 | 7 | 6.61 | 6.81 |
| Chr10:32474621-32474643 | 32474626 | 3 | RNA | GGCCGAGGTCGACTACCGGCNRG | tGCCGA--TacACcgCCGGCCGG | - | 5 | 2 | 0 | 13.22 | 6.61 |
| Chr4:170766307-170766329 | 170766324 | 3 | DNA | GGCCGAGGTCGACTACCG--GCNRG | GGCgGAGGTCGcCggCtGATGCCGG | + | 5 | 2 | 0 | 13.22 | 6.61 |
| Chr4:159977272-159977294 | 159977288 | 4 | RNA | GGCCGAGGTCGACTACCGGCNRG | GGCaGtGGcgGACTAC-GGtCAG | + | 5 | 1 | 0 | 13.22 | 6.61 |
| Chr1:94016475-94016497 | 94016480 | 3 | RNA | GGCCGAGGTCGACTACCGGCNRG | GcCCGAGccCGAgT--CGGtAGG | - | 5 | 2 | 0 | 13.22 | 6.61 |
| Chr2:10567713-10567735 | 10567719 | 4 | RNA | GGCCGAGGTCGACTACCGGCNRG | GcggcAGGTgG--TACCGGCGAG | - | 5 | 2 | 0 | 13.22 | 6.61 |
| Chr2:178371913-178371935 | 178371928 | 5 | RNA | GGCCGAGGTCGACTACCGGCNRG | GGC--tcGcgGACTACCGtCTGG | + | 5 | 2 | 0 | 13.22 | 6.61 |
| Chr3:25383214-25383236 | 25383231 | 3 | DNA | GGC-CGAGGTCGACTACCGGCNRG | GcCTCGAGGaCGtCTtCCGGCTGG | + | 4 | 1 | 1 | 12.12 | 6.56 |
| Chr4:108817238-108817260 | 108817253 | 5 | RNA | GGCCGAGGTCGACTACCGGCNRG | cGC--AGGgCtACTgCCGGCCGG | + | 4 | 2 | 7 | 6.06 | 6.53 |
| Chr3:110757482-110757504 | 110757489 | 5 | RNA | GGCCGAGGTCGACTACCGGCNRG | G-aCGAGGTCGACgACgaGaTAG | - | 5 | 1 | 13 | 0.00 | 6.50 |
| Chr1:120789643-120789665 | 120789650 | 5 | RNA | GGCCGAGGTCGACTACCGGCNRG | aGagcAG--CGgCTACCGGCTGG | - | 5 | 2 | 13 | 0.00 | 6.50 |
| Chr6:29254942-29254964 | 29254959 | 3 | RNA | GGCCGAGGTCGACTACCGGCNRG | tGCaGAGcTCGgCTA--GGtTGG | + | 5 | 2 | 13 | 0.00 | 6.50 |
| Chr2:38124656-38124678 | 38124673 | 3 | RNA | GGCCGAGGTCGACTACCGGCNRG | tGgCt-cGTgGACTACCGGCTGG | + | 5 | 1 | 13 | 0.00 | 6.50 |
| Chr5:26296327-26296349 | 26296344 | 3 | RNA | GGCCGAGGTCGACTACCGGCNRG | ccCCGAaGTCGAtTAtC--CCGG | + | 5 | 2 | 13 | 0.00 | 6.50 |
| Chr8:12282731-12282753 | 12282748 | 3 | DNA | GG-CCGAGGTCGACTACCGGCNRG | GGTCCGAGtTCaAgaACCtGCAGG | + | 5 | 1 | 13 | 0.00 | 6.50 |
| Chr7:129636568-129636590 | 129636585 | 3 | RNA | GGCCGAGGTCGACTACCGGCNRG | GGCCGAGtaCG--TtgCcGCAGG | + | 5 | 2 | 13 | 0.00 | 6.50 |
| Chr5:30544853-30544875 | 30544860 | 5 | RNA | GGCCGAGGTCGACTACCGGCNRG | GcCaGtGGaCGAC--CCGGCTAG | - | 4 | 2 | 13 | 0.00 | 6.50 |
| Chr4:79823897-79823919 | 79823914 | 3 | RNA | GGCCGAGGTCGACTACCGGCNRG | GcCgGAGccCGAC--CCGtCAAG | + | 5 | 2 | 13 | 0.00 | 6.50 |
| Chr1:10571571-10571593 | 10571578 | 5 | RNA | GGCCGAGGTCGACTACCGGCNRG | GGtaGctGTCGACT-CtGGCGGG | - | 5 | 1 | 13 | 0.00 | 6.50 |
| Chr4:218341908-218341930 | 218341925 | 3 | RNA | GGCCGAGGTCGACTACCGGCNRG | GGCCGcGGcCG--TgCCGGaGAG | + | 4 | 2 | 13 | 0.00 | 6.50 |
| Chr7:3885860-3885882 | 3885877 | 3 | RNA | GGCCGAGGTCGACTACCGGCNRG | aGCaGgGcTCGACTAC-GGaCGG | + | 5 | 1 | 13 | 0.00 | 6.50 |
| Chr5:5053680-5053702 | 5053697 | 3 | RNA | GGCCGAGGTCGACTACCGGCNRG | GGCgGcGGgCGgCTt--GGCAGG | + | 5 | 2 | 13 | 0.00 | 6.50 |
| Chr6:644841-644863 | 644858 | 3 | RNA | GGCCGAGGTCGACTACCGGCNRG | GGCCtAGtTgcACTACt--CAGG | + | 5 | 2 | 4 | 8.81 | 6.41 |
| Chr6:153932019-153932041 | 153932035 | 4 | RNA | GGCCGAGGTCGACTACCGGCNRG | GGCaGAGGTtGgCgAg-GGCCGG | + | 5 | 1 | 5 | 7.71 | 6.36 |
| Chr7:105603645-105603667 | 105603652 | 5 | RNA | GGCCGAGGTCGACTACCGGCNRG | aGttGAG--aGACTACCGGgGGG | - | 5 | 2 | 0 | 12.67 | 6.34 |
| Chr5:176778964-176778986 | 176778982 | 2 | RNA | GGCCGAGGTCGACTACCGGCNRG | GGCtcAG--CGACTgaCGGgAAG | + | 5 | 2 | 0 | 12.67 | 6.34 |
| Chr5:34923149-34923171 | 34923154 | 3 | DNA | GGC--CGAGGTCGACTACCGGCNRG | GGCCTCGAGGgCtACacCCGGtGGG | - | 5 | 2 | 0 | 12.67 | 6.34 |
| Chr4:163611678-163611700 | 163611696 | 2 | RNA | GGCCGAGGTCGACTACCGGCNRG | GGC--AGGcCGAgcACtaGCCAG | + | 5 | 2 | 0 | 12.67 | 6.34 |
| Chr2:82089788-82089810 | 82089792 | 2 | RNA | GGCCGAGGTCGACTACCGGCNRG | tGCaGAGcTCGgCTA--GGCTGG | - | 4 | 2 | 0 | 12.67 | 6.34 |
| Chr6:117209554-117209576 | 117209572 | 2 | RNA | GGCCGAGGTCGACTACCGGCNRG | GaCCGAGG-CGACgACaaGaAGG | + | 5 | 1 | 0 | 12.67 | 6.34 |
| Chr8:70932227-70932249 | 70932245 | 2 | RNA | GGCCGAGGTCGACTACCGGCNRG | GGgCGAGtTtGggTA--GGCAGG | + | 5 | 2 | 0 | 12.67 | 6.34 |
| Chr4:176320826-176320848 | 176320842 | 4 | RNA | GGCCGAGGTCGACTACCGGCNRG | G-aCGgGGTCGACaACgaGCGGG | + | 5 | 1 | 0 | 12.67 | 6.34 |
| Chr2:78095139-78095161 | 78095144 | 3 | RNA | GGCCGAGGTCGACTACCGGCNRG | GGCCGAGcTCccCTggCG-CAGG | - | 5 | 1 | 0 | 12.67 | 6.34 |
| Chr10:71971177-71971199 | 71971193 | 4 | RNA | GGCCGAGGTCGACTACCGGCNRG | GGC-GtGGTCGAggACtGGCGGG | + | 4 | 1 | 0 | 12.67 | 6.34 |
| Chr1:96437551-96437573 | 96437557 | 4 | DNA | GGC-CGAGGTCGACTACCGGCNRG | GcCTCGAGGaCGtCTtCCGGCTGG | - | 4 | 1 | 7 | 5.51 | 6.26 |
| Chr3:85941004-85941026 | 85941010 | 4 | RNA | GGCCGAGGTCGACTACCGGCNRG | tGaaGAGGc--ACTACCtGCGGG | - | 5 | 2 | 0 | 12.12 | 6.06 |
| Chr1:147535889-147535911 | 147535904 | 5 | RNA | GGCCGAGGTCGACTACCGGCNRG | t--CGcGtTgaACTACCGGCTGG | + | 5 | 2 | 0 | 12.12 | 6.06 |

| | | | | | | | | | | | | |
|---|---|---|---|---|---|---|---|---|---|---|---|---|
| Chr6:47560230-47560252 | 47560246 | 4 | RNA | GGCCGAGGTCGACTACCGGCNRG | G-aCGAGaTCGACaACgaGCGGG | + | 5 | 1 | 0 | 12.12 | 6.06 |
| Chr1:113110081-113110103 | 113110088 | 5 | DNA | GGCCGAGGTCGACT-ACCGGCNRG | GGCtGgGGaCGACTGACaGGtGGG | - | 5 | 1 | 0 | 12.12 | 6.06 |
| Chr8:56668510-56668532 | 56668528 | 2 | RNA | GGCCGAGGTCGACTACCGGCNRG | aGCCGAcG--GACTcgCtGCTGG | + | 5 | 2 | 0 | 12.12 | 6.06 |
| Chr3:158346111-158346133 | 158346128 | 3 | RNA | GGCCGAGGTCGACTACCGGCNRG | GGCCGgGtcgGAC--CgGGCCGG | + | 5 | 2 | 0 | 12.12 | 6.06 |
| Chr6:160184621-160184643 | 160184639 | 2 | RNA | GGCCGAGGTCGACTACCGGCNRG | GaCC-AGGTCttCTtCCaGCAGG | + | 5 | 1 | 0 | 12.12 | 6.06 |
| Chr9:109053816-109053838 | 109053831 | 5 | RNA | GGCCGAGGTCGACTACCGGCNRG | G--tGAGGaCGACTgCaGGCGGG | + | 4 | 2 | 0 | 12.12 | 6.06 |
| Chr5:17487623-17487645 | 17487628 | 3 | RNA | GGCCGAGGTCGACTACCGGCNRG | G-aCGAGGTCGACaACgaGCGGG | - | 4 | 1 | 0 | 12.12 | 6.06 |
| Chr6:101731284-101731306 | 101731290 | 4 | DNA | GGC-CGAGGTCGACTACCGGCNRG | GcCTCGAGGgCatCTtCCGGCGGG | - | 5 | 1 | 0 | 12.12 | 6.06 |
| Chr8:153226687-153226709 | 153226694 | 5 | RNA | GGCCGAGGTCGACTACCGGCNRG | ttCCGAtGTCGAtTg--GGCGGG | - | 5 | 2 | 0 | 12.12 | 6.06 |
| Chr3:42113853-42113875 | 42113869 | 4 | RNA | GGCCGAGGTCGACTACCGGCNRG | GGCCGAcGcCGAtgA--GaCGAG | + | 5 | 2 | 0 | 12.12 | 6.06 |
| Chr1:164563816-164563838 | 164563831 | 5 | RNA | GGCCGAGGTCGACTACCGGCNRG | GGCaGAGGT-GACggCCGGaGAG | + | 4 | 1 | 0 | 12.12 | 6.06 |
| Chr6:67380621-67380643 | 67380638 | 3 | RNA | GGCCGAGGTCGACTACCGGCNRG | GGCCGcGGaCGACT-CtGGCGAG | + | 3 | 1 | 12 | 0.00 | 6.00 |
| Chr9:79804613-79804635 | 79804630 | 3 | RNA | GGCCGAGGTCGACTACCGGCNRG | cGaCGAGcTCa--TACCGGCCGG | + | 4 | 2 | 12 | 0.00 | 6.00 |
| Chr2:57971352-57971374 | 57971369 | 3 | RNA | GGCCGAGGTCGACTACCGGCNRG | GGaCGAG--CGctgACaGGCGGG | + | 5 | 2 | 12 | 0.00 | 6.00 |
| Chr6:75926787-75926809 | 75926804 | 3 | DNA | G-GCCGAGGTCGACTACCGGCNRG | GTGCtGAGaTCGcCTAgCtGCGGG | + | 5 | 1 | 12 | 0.00 | 6.00 |
| Chr9:105659733-105659755 | 105659740 | 5 | DNA | GGCCGAGGT--CGACTACCGGCNRG | GGCCGAGGTCGCGACTcatGGgCGG | - | 4 | 2 | 12 | 0.00 | 6.00 |
| Chr5:24959542-24959564 | 24959549 | 5 | RNA | GGCCGAGGTCGACTACCGGCNRG | GG--GAGGggGgCTtCCGGCGGG | - | 4 | 2 | 12 | 0.00 | 6.00 |
| Chr1:163300941-163300963 | 163300958 | 3 | RNA | GGCCGAGGTCGACTACCGGCNRG | GtgCGcGGaCG-CTACtGGCAGG | + | 5 | 1 | 12 | 0.00 | 6.00 |
| Chr7:152918617-152918639 | 152918624 | 5 | DNA | GGCCGAGGTCGACTAC-CGGCNRG | cGCCaAGaTCGACaACGCGcCCGG | - | 5 | 1 | 12 | 0.00 | 6.00 |
| Chr9:133680153-133680175 | 133680170 | 3 | RNA | GGCCGAGGTCGACTACCGGCNRG | GGaCcAGGTCG--TcCCaGtTGG | + | 5 | 2 | 12 | 0.00 | 6.00 |
| Chr6:66171617-66171639 | 66171634 | 3 | RNA | GGCCGAGGTCGACTACCGGCNRG | G-aCGgGGTCGACaACgaGCGGG | + | 5 | 1 | 12 | 0.00 | 6.00 |
| Chr6:93690437-93690459 | 93690444 | 5 | RNA | GGCCGAGGTCGACTACCGGCNRG | tGCCGAGGaC-ACTgCCtGgGAG | - | 5 | 1 | 12 | 0.00 | 6.00 |
| Chr1:122673697-122673719 | 122673704 | 5 | RNA | GGCCGAGGTCGACTACCGGCNRG | GGCCGAaGc--ACTgCCaGaTAG | - | 5 | 2 | 12 | 0.00 | 6.00 |
| Chr4:80762391-80762413 | 80762408 | 3 | RNA | GGCCGAGGTCGACTACCGGCNRG | G-aCGgGGTCGACaACgaGCGGG | + | 5 | 1 | 12 | 0.00 | 6.00 |
| Chr3:4545233-4545255 | 4545240 | 5 | RNA | GGCCGAGGTCGACTACCGGCNRG | G-aCGgGGTCGACaACgaGCGGG | - | 5 | 1 | 12 | 0.00 | 6.00 |
| Chr4:108794616-108794638 | 108794633 | 3 | RNA | GGCCGAGGTCGACTACCGGCNRG | tGgCt-cGTgGACTACCGGCTGG | + | 5 | 1 | 12 | 0.00 | 6.00 |
| Chr7:128072344-128072366 | 128072361 | 3 | RNA | GGCCGAGGTCGACTACCGGCNRG | G-aCGAGGTCGAtaACgaGCGGG | + | 5 | 1 | 12 | 0.00 | 6.00 |
| Chr2:51154691-51154713 | 51154698 | 5 | RNA | GGCCGAGGTCGACTACCGGCNRG | GGCCG-GGTCGtCcACtGGtGGG | - | 4 | 1 | 12 | 0.00 | 6.00 |
| Chr4:116151160-116151182 | 116151177 | 3 | RNA | GGCCGAGGTCGACTACCGGCNRG | GGggGAGGTaGcCgA--GGCTGG | + | 5 | 2 | 12 | 0.00 | 6.00 |
| Chr2:6101136-6101158 | 6101143 | 5 | RNA | GGCCGAGGTCGACTACCGGCNRG | cGCCaAGGTCGAggA--GGtCAG | - | 5 | 2 | 12 | 0.00 | 6.00 |
| Chr4:114880759-114880781 | 114880776 | 3 | RNA | GGCCGAGGTCGACTACCGGCNRG | GGC-aAGGTCGACgACgtGaTGG | + | 5 | 1 | 12 | 0.00 | 6.00 |
| Chr8:27801455-27801477 | 27801462 | 5 | RNA | GGCCGAGGTCGACTACCGGCNRG | G-aCGAGGTCGAtgACaaGCGGG | - | 5 | 1 | 12 | 0.00 | 6.00 |
| Chr5:120860746-120860768 | 120860764 | 2 | DNA | GGCCGAGGTCGACTAC-CGGCNRG | GGtCGAGGgCGcgTcCACGGCGGG | + | 5 | 1 | 5 | 6.61 | 5.81 |
| Chr2:154881381-154881403 | 154881397 | 4 | RNA | GGCCGAGGTCGACTACCGGCNRG | aGCaGgGcTCGACTAC-GGCCGG | + | 4 | 1 | 0 | 11.57 | 5.79 |
| Chr3:199019683-199019705 | 199019688 | 3 | RNA | GGCCGAGGTCGACTACCGGCNRG | GGC--tcGgCGACaACaGGCTGG | - | 5 | 2 | 0 | 11.57 | 5.79 |
| Chr4:113372151-113372173 | 113372158 | 5 | RNA | GGCCGAGGTCGACTACCGGCNRG | GGC-GtGGaCGAactCCGGCGAG | - | 5 | 1 | 0 | 11.57 | 5.79 |
| Chr6:154812669-154812691 | 154812687 | 2 | RNA | GGCCGAGGTCGACTACCGGCNRG | GGCtcAGGctGACaAC--GCGGG | + | 5 | 2 | 0 | 11.57 | 5.79 |
| Chr4:159335495-159335517 | 159335500 | 3 | RNA | GGCCGAGGTCGACTACCGGCNRG | GGCtcAcG--GACTACtGaCTGG | - | 5 | 2 | 0 | 11.57 | 5.79 |
| Chr8:93012665-93012687 | 93012681 | 4 | RNA | GGCCGAGGTCGACTACCGGCNRG | G-aCGgGGTCGACgACgaGCGGG | + | 5 | 1 | 0 | 11.57 | 5.79 |
| Chr4:140692052-140692074 | 140692067 | 5 | RNA | GGCCGAGGTCGACTACCGGCNRG | GGC-GAcGTCGACTACtacgAGG | + | 5 | 1 | 0 | 11.57 | 5.79 |
| Chr3:232121528-232121550 | 232121546 | 2 | RNA | GGCCGAGGTCGACTACCGGCNRG | tG--GAGGagGAgcACCGGCTAG | + | 5 | 2 | 0 | 11.57 | 5.79 |
| Chr1:180423414-180423436 | 180423429 | 5 | RNA | GGCCGAGGTCGACTACCGGCNRG | GGC--ttGcgGAtTACCGGCTGG | + | 5 | 2 | 0 | 11.57 | 5.79 |
| Chr5:208880944-208880966 | 208880960 | 4 | RNA | GGCCGAGGTCGACTACCGGCNRG | GGCgGcGaTaGcCT--CGGCAAG | + | 5 | 2 | 0 | 11.57 | 5.79 |
| Chr7:2295613-2295635 | 2295620 | 5 | DNA | GGCCGAGGTCGACTA--CCGGCNRG | GGCgGcGcTaGcCTACTCCGGCGAG | - | 5 | 2 | 11 | 0.55 | 5.78 |
| Chr8:161124161-161124183 | 161124167 | 4 | DNA | GGC-CGAGGTCGACTACCGGCNRG | GcCTCGAGGaCGtCTtCCGGCTGG | - | 4 | 1 | 6 | 5.51 | 5.76 |
| Chr4:93494700-93494722 | 93494718 | 2 | DNA | GGCCGAGGTC-GACTACCGGCNRG | GGCCGAGGcCTGAggtCgGGCGAG | + | 5 | 1 | 6 | 5.51 | 5.76 |
| Chr2:145249710-145249732 | 145249716 | 4 | RNA | GGCCGAGGTCGACTACCGGCNRG | GGgCGAcGaCGgC-AgCGGCAAG | - | 5 | 1 | 2 | 9.36 | 5.68 |
| Chr4:154376219-154376241 | 154376223 | 2 | RNA | GGCCGAGGTCGACTACCGGCNRG | GGCCGAtcTCG--cAgCaGCAAG | - | 5 | 2 | 8 | 3.30 | 5.65 |
| Chr6:144813984-144814006 | 144813991 | 5 | RNA | GGCCGAGGTCGACTACCGGCNRG | GctCGAGaTCGACTg--GGaTGG | - | 5 | 2 | 10 | 1.10 | 5.55 |
| Chr2:169106015-169106037 | 169106030 | 5 | RNA | GGCCGAGGTCGACTACCGGCNRG | G--tGAGGTaatCaACCGGCAGG | + | 5 | 2 | 5 | 6.06 | 5.53 |
| Chr1:80665235-80665257 | 80665239 | 2 | RNA | GGCCGAGGTCGACTACCGGCNRG | GGCCa--GctGACgACCaGCTGG | - | 5 | 2 | 0 | 11.02 | 5.51 |
| Chr4:130428771-130428793 | 130428788 | 3 | DNA | GGC-CGAGGTCGACTACCGGCNRG | GcCTCGAGGaCGtCTtCCGGCTGG | + | 4 | 1 | 0 | 11.02 | 5.51 |
| Chr5:197998205-197998227 | 197998222 | 3 | RNA | GGCCGAGGTCGACTACCGGCNRG | GGCgGtGGTCGAtgAC--GaAGG | + | 5 | 2 | 0 | 11.02 | 5.51 |
| Chr7:105179774-105179796 | 105179791 | 3 | RNA | GGCCGAGGTCGACTACCGGCNRG | GGCCGAGcgCGA--AggGGgAAG | + | 5 | 2 | 0 | 11.02 | 5.51 |
| Chr10:121776696-121776718 | 121776714 | 2 | RNA | GGCCGAGGTCGACTACCGGCNRG | GGCCGgcGgCGAgg--CGGCGAG | + | 5 | 2 | 0 | 11.02 | 5.51 |
| Chr9:10406323-10406345 | 10406341 | 2 | RNA | GGCCGAGGTCGACTACCGGCNRG | GGt--AGGcCaACctCCGGCCGG | + | 5 | 2 | 0 | 11.02 | 5.51 |
| Chr8:127021243-127021265 | 127021248 | 3 | RNA | GGCCGAGGTCGACTACCGGCNRG | G-aCGgGGTCGACaACgaGCGGG | - | 5 | 1 | 0 | 11.02 | 5.51 |
| Chr4:177107054-177107076 | 177107070 | 4 | DNA | GG-CCGAGGTCGACTACCGGCNRG | GGTCCGAGGTCGAggtCaGGCGAG | + | 4 | 1 | 0 | 11.02 | 5.51 |
| Chr9:82008064-82008086 | 82008081 | 3 | RNA | GGCCGAGGTCGACTACCGGCNRG | GGaCGAGGgCG--cAggGGCAGG | + | 5 | 2 | 0 | 11.02 | 5.51 |
| Chr2:118622125-118622147 | 118622129 | 2 | RNA | GGCCGAGGTCGACTACCGGCNRG | GGCCGAtcTCG--cAtCaGCAAG | - | 5 | 2 | 0 | 11.02 | 5.51 |
| Chr10:150131590-150131612 | 150131596 | 4 | RNA | GGCCGAGGTCGACTACCGGCNRG | GGC--AGacCGAgcACCaGCCGG | - | 5 | 2 | 0 | 11.02 | 5.51 |
| Chr10:27044129-27044151 | 27044135 | 4 | RNA | GGCCGAGGTCGACTACCGGCNRG | GGCCGccGTCGcCgtC--GCCAG | - | 5 | 2 | 0 | 11.02 | 5.51 |
| Chr5:75886405-75886427 | 75886420 | 5 | RNA | GGCCGAGGTCGACTACCGGCNRG | GGC--tcGcgGACTACCGtCTGG | + | 5 | 2 | 0 | 11.02 | 5.51 |
| Chr4:215783270-215783292 | 215783274 | 2 | RNA | GGCCGAGGTCGACTACCGGCNRG | GGCCGgGcTgGAC--CgGGCCGG | - | 4 | 2 | 0 | 11.02 | 5.51 |
| Chr10:93909137-93909159 | 93909154 | 3 | RNA | GGCCGAGGTCGACTACCGGCNRG | GaCC-AGcTCGAgTACCtGgAGG | + | 5 | 1 | 0 | 11.02 | 5.51 |
| Chr10:70309314-70309336 | 70309331 | 3 | RNA | GGCCGAGGTCGACTACCGGCNRG | ccCCcAG--CGAacACCGGCGAG | + | 5 | 2 | 0 | 11.02 | 5.51 |
| Chr1:30966896-30966918 | 30966912 | 4 | DNA | GGCCGA-GGTCGACTACCGGCNRG | GGaCGATGGcCGACgAaCGGaAAG | + | 5 | 1 | 0 | 11.02 | 5.51 |
| Chr8:154260387-154260409 | 154260394 | 5 | RNA | GGCCGAGGTCGACTACCGGCNRG | aGCCGtGtTCGACTACC--CGGG | - | 3 | 2 | 11 | 0.00 | 5.50 |
| Chr4:210393287-210393309 | 210393294 | 5 | RNA | GGCCGAGGTCGACTACCGGCNRG | GGaCGAGGgC-ACTAtCGGCTGG | - | 3 | 1 | 11 | 0.00 | 5.50 |
| Chr4:82363661-82363683 | 82363668 | 5 | RNA | GGCCGAGGTCGACTACCGGCNRG | cGaCGAcGTCGA-TcCCGGtGAG | - | 5 | 1 | 11 | 0.00 | 5.50 |
| Chr8:152619574-152619596 | 152619581 | 5 | RNA | GGCCGAGGTCGACTACCGGCNRG | GaCCGAGG-CGACgACaaGaAGG | - | 5 | 1 | 11 | 0.00 | 5.50 |
| Chr10:40287830-40287852 | 40287837 | 5 | RNA | GGCCGAGGTCGACTACCGGCNRG | caCCGAGtTgGAgTA--GGCAGG | - | 5 | 2 | 11 | 0.00 | 5.50 |
| Chr4:46003613-46003635 | 46003620 | 5 | RNA | GGCCGAGGTCGACTACCGGCNRG | GagCGAG-TCGtCaACCGtCCAG | - | 5 | 1 | 11 | 0.00 | 5.50 |
| Chr5:49117147-49117169 | 49117164 | 3 | RNA | GGCCGAGGTCGACTACCGGCNRG | GGCCGAGGaCcAggACC--CAAG | + | 4 | 2 | 11 | 0.00 | 5.50 |
| Chr1:121497457-121497479 | 121497474 | 3 | RNA | GGCCGAGGTCGACTACCGGCNRG | GGaCG--GTCGcggACCGtCCGG | + | 5 | 2 | 11 | 0.00 | 5.50 |
| Chr2:224654169-224654191 | 224654186 | 3 | DNA | GGCCGAGGTCGAC-TACCGGCNRG | aGCgGAGGgCGACGaACtGGCGGG | + | 5 | 1 | 11 | 0.00 | 5.50 |
| Chr8:162435245-162435267 | 162435252 | 5 | RNA | GGCCGAGGTCGACTACCGGCNRG | cGCCGAc--CGAggACCtGCAGG | - | 5 | 2 | 11 | 0.00 | 5.50 |
| Chr6:161132228-161132250 | 161132235 | 5 | RNA | GGCCGAGGTCGACTACCGGCNRG | tGgCGtGGTCctCTACC--CAGG | - | 5 | 2 | 11 | 0.00 | 5.50 |
| Chr5:178967961-178967983 | 178967978 | 3 | DNA | GGCCG--AGGTCGACTACCGGCNRG | tGCCGGAAGGTaGgtTtCCGGCTAG | + | 5 | 2 | 11 | 0.00 | 5.50 |
| Chr1:71210030-71210052 | 71210037 | 5 | RNA | GGCCGAGGTCGACTACCGGCNRG | GGCaGcGGTCGAC-AgCGGgCGG | - | 4 | 1 | 11 | 0.00 | 5.50 |
| Chr9:65874133-65874155 | 65874140 | 5 | RNA | GGCCGAGGTCGACTACCGGCNRG | GGC--AGGTCGggcACtcGCCGG | - | 5 | 2 | 11 | 0.00 | 5.50 |
| Chr8:52557746-52557768 | 52557763 | 3 | RNA | GGCCGAGGTCGACTACCGGCNRG | cGCCGAGG--GAgTtCCaGCAGG | + | 4 | 2 | 11 | 0.00 | 5.50 |
| Chr7:145657742-145657764 | 145657749 | 5 | RNA | GGCCGAGGTCGACTACCGGCNRG | GtCttgGGT-GACcACCGGCGGG | - | 5 | 1 | 11 | 0.00 | 5.50 |
| Chr6:71767056-71767078 | 71767073 | 3 | RNA | GGCCGAGGTCGACTACCGGCNRG | GGCaGtGa-CGACTACCaGCAGG | + | 4 | 1 | 11 | 0.00 | 5.50 |
| Chr6:97576679-97576701 | 97576696 | 3 | RNA | GGCCGAGGTCGACTACCGGCNRG | GGC--tcGcgGACTACCGtCTGG | + | 5 | 2 | 11 | 0.00 | 5.50 |
| Chr1:273888216-273888238 | 273888233 | 3 | RNA | GGCCGAGGTCGACTACCGGCNRG | GcCCGAGGaCaACcACC--aCGG | + | 5 | 2 | 11 | 0.00 | 5.50 |
| Chr1:85262125-85262147 | 85262130 | 3 | RNA | GGCCGAGGTCGACTACCGGCNRG | GcCCGAGGcCGA--ACtcGgTGG | - | 5 | 2 | 7 | 3.86 | 5.43 |
| Chr7:77522872-77522894 | 77522877 | 3 | RNA | GGCCGAGGTCGACTACCGGCNRG | GGaCct--TCGAaTAgCGGCAGG | - | 5 | 2 | 3 | 7.71 | 5.36 |
| Chr6:49789848-49789870 | 49789853 | 3 | RNA | GGCCGAGGTCGACTACCGGCNRG | GcCCGAGGgCaACcACC--gTGG | - | 5 | 2 | 4 | 6.61 | 5.31 |
| Chr9:58610543-58610565 | 58610559 | 4 | RNA | GGCCGAGGTCGACTACCGGCNRG | GGCCGAGGgCGcCT--CaGgCGG | + | 4 | 2 | 10 | 0.55 | 5.28 |
| Chr10:64036763-64036785 | 64036768 | 3 | RNA | GGCCGAGGTCGACTACCGGCNRG | GGC--AGGcCGAgcACtcGCCGG | - | 5 | 2 | 0 | 10.46 | 5.23 |
| Chr8:11633727-11633749 | 11633732 | 3 | RNA | GGCCGAGGTCGACTACCGGCNRG | GGC--tcGcgGACTACCGtCTGG | - | 5 | 2 | 0 | 10.46 | 5.23 |
| Chr2:194946823-194946845 | 194946830 | 5 | RNA | GGCCGAGGTCGACTACCGGCNRG | GGC--AGGcCGAgcACtcGCCGG | - | 5 | 2 | 0 | 10.46 | 5.23 |
| Chr10:5403108-5403130 | 5403113 | 3 | RNA | GGCCGAGGTCGACTACCGGCNRG | GGC-GgGGTCGAaTcCCGcCTGG | - | 4 | 1 | 0 | 10.46 | 5.23 |
| Chr9:52767017-52767039 | 52767023 | 4 | RNA | GGCCGAGGTCGACTACCGGCNRG | GcCCGAGtTCGtCatC--GCCGG | - | 5 | 2 | 0 | 10.46 | 5.23 |
| Chr10:99687435-99687457 | 99687453 | 2 | RNA | GGCCGAGGTCGACTACCGGCNRG | G--CGAGGTgGAgctCaGGCAGG | + | 5 | 2 | 0 | 10.46 | 5.23 |
| Chr4:35078794-35078816 | 35078799 | 3 | RNA | GGCCGAGGTCGACTACCGGCNRG | GGgCG-GGcCaACctCCGGCCGG | - | 5 | 1 | 0 | 10.46 | 5.23 |
| Chr4:42312088-42312110 | 42312104 | 4 | RNA | GGCCGAGGTCGACTACCGGCNRG | GGCtaAGG-CtACTAgCaGCAGG | + | 5 | 1 | 0 | 10.46 | 5.23 |
| Chr9:156369685-156369707 | 156369701 | 4 | RNA | GGCCGAGGTCGACTACCGGCNRG | GGgCG-GGcCaACctCCGGCCGG | + | 5 | 1 | 0 | 10.46 | 5.23 |
| Chr2:152782016-152782038 | 152782032 | 4 | RNA | GGCCGAGGTCGACTACCGGCNRG | a--CGAcGaCGACaACCGGCAAG | + | 4 | 2 | 0 | 10.46 | 5.23 |
| Chr6:125744945-125744967 | 125744952 | 5 | RNA | GGCCGAGGTCGACTACCGGCNRG | caCCaAGtTCGcCT-CCGGCCAG | - | 5 | 1 | 0 | 10.46 | 5.23 |
| Chr4:230595515-230595537 | 230595520 | 3 | RNA | GGCCGAGGTCGACTACCGGCNRG | GGC--tcGcgGACTACtGGCTGG | - | 5 | 2 | 0 | 10.46 | 5.23 |
| Chr8:78696806-78696828 | 78696813 | 5 | DNA | GGC--CGAGGTCGACTACCGGCNRG | GGCCTCGgGGgCtACacCCGGCGGG | - | 5 | 2 | 0 | 10.46 | 5.23 |
| Chr10:58359901-58359923 | 58359916 | 5 | RNA | GGCCGAGGTCGACTACCGGCNRG | GGCgGAaGTCG--TcCgGcCGAG | + | 5 | 2 | 0 | 10.46 | 5.23 |
| Chr4:158275718-158275740 | 158275735 | 3 | RNA | GGCCGAGGTCGACTACCGGCNRG | GGC-GAGGTCGAggACCttgGGG | + | 5 | 1 | 0 | 10.46 | 5.23 |
| Chr3:481745-481767 | 481760 | 5 | RNA | GGCCGAGGTCGACTACCGGCNRG | GGCCGgGGTgGAtgACCt-CGAG | + | 5 | 1 | 0 | 10.46 | 5.23 |
| Chr1:122869678-122869700 | 122869693 | 5 | RNA | GGCCGAGGTCGACTACCGGCNRG | G--CGgGGaCatCTtCCGGCTGG | + | 5 | 2 | 0 | 10.46 | 5.23 |
| Chr8:70699892-70699914 | 70699897 | 3 | RNA | GGCCGAGGTCGACTACCGGCNRG | GGC-GAGGTCGACgACgaGaTAG | - | 4 | 1 | 0 | 10.46 | 5.23 |
| Chr8:83477111-83477133 | 83477116 | 3 | RNA | GGCCGAGGTCGACTACCGGCNRG | GGgCGAGcTCGgCcAC-GGCAAG | - | 4 | 1 | 6 | 4.41 | 5.21 |
| Chr1:228759322-228759344 | 228759339 | 3 | RNA | GGCCGAGGTCGACTACCGGCNRG | tGCCtA--TCacCTACCaGCAGG | + | 5 | 2 | 9 | 1.10 | 5.05 |
| Chr2:184130574-184130596 | 184130581 | 5 | RNA | GGCCGAGGTCGACTACCGGCNRG | G-aCGgGGTCGACaACgaGCGGG | - | 5 | 1 | 10 | 0.00 | 5.00 |
| Chr9:129865023-129865045 | 129865030 | 5 | RNA | GGCCGAGGTCGACTACCGGCNRG | GGCCGAcGTCaAggAC-GGCGGG | - | 4 | 1 | 10 | 0.00 | 5.00 |
| Chr2:121388382-121388404 | 121388389 | 5 | RNA | GGCCGAGGTCGACTACCGGCNRG | tGaCcAGGTCG--TcCCaGCTGG | - | 5 | 2 | 10 | 0.00 | 5.00 |
| Chr1:152646417-152646439 | 152646434 | 3 | RNA | GGCCGAGGTCGACTACCGGCNRG | GGC-GAcGTCGACTACtacaAGG | + | 5 | 1 | 10 | 0.00 | 5.00 |
| Chr9:158630407-158630429 | 158630414 | 5 | RNA | GGCCGAGGTCGACTACCGGCNRG | tGCCGAG--CGgaTAgCGGaGAG | - | 5 | 2 | 10 | 0.00 | 5.00 |
| Chr5:125101643-125101665 | 125101660 | 3 | RNA | GGCCGAGGTCGACTACCGGCNRG | G-aCGgGGTCGACaACgaGCGGG | + | 5 | 1 | 10 | 0.00 | 5.00 |
| Chr4:100906350-100906372 | 100906357 | 5 | DNA | GGCCGAGGTCGACTACC--GGCNRG | GcCCaAGGgCaACcACCGTGGCCAG | - | 5 | 2 | 10 | 0.00 | 5.00 |
| Chr6:167289319-167289341 | 167289336 | 3 | RNA | GGCCGAGGTCGACTACCGGCNRG | tGCCtcGGTgGtC-ACCGGCCGG | + | 5 | 1 | 10 | 0.00 | 5.00 |
| Chr1:125171914-125171936 | 125171931 | 3 | RNA | GGCCGAGGTCGACTACCGGCNRG | a--CGgGGTCGACgACgaGCGGG | + | 5 | 2 | 10 | 0.00 | 5.00 |
| Chr4:167248420-167248442 | 167248427 | 5 | RNA | GGCCGAGGTCGACTACCGGCNRG | GGCgGAGG--GACagCtGtCGGG | - | 5 | 2 | 10 | 0.00 | 5.00 |
| Chr3:154208857-154208879 | 154208864 | 5 | RNA | GGCCGAGGTCGACTACCGGCNRG | GGC--AGGcCaACctCCGGCCGG | - | 4 | 2 | 10 | 0.00 | 5.00 |
| Chr2:223655119-223655141 | 223655126 | 5 | RNA | GGCCGAGGTCGACTACCGGCNRG | GGCacAGGaC-ACTACaGGCAGG | - | 4 | 1 | 10 | 0.00 | 5.00 |
| Chr9:141174795-141174817 | 141174802 | 5 | RNA | GGCCGAGGTCGACTACCGGCNRG | GGCCc--GaCtACTACtGGtGAG | - | 5 | 2 | 10 | 0.00 | 5.00 |
| Chr3:176135769-176135791 | 176135776 | 5 | RNA | GGCCGAGGTCGACTACCGGCNRG | G-aCGgGGTCGACgACgaGCGGG | - | 5 | 1 | 10 | 0.00 | 5.00 |
| Chr3:200728717-200728739 | 200728724 | 5 | RNA | GGCCGAGGTCGACTACCGGCNRG | GGC--tcGcgGACTACCGcCTGG | - | 5 | 2 | 10 | 0.00 | 5.00 |
| Chr2:20386778-20386800 | 20386795 | 3 | RNA | GGCCGAGGTCGACTACCGGCNRG | GGCaaAGG-CaACaACaGGCAGG | + | 5 | 1 | 10 | 0.00 | 5.00 |

| | | | | | | | | | | | |
|---|---|---|---|---|---|---|---|---|---|---|---|
| Chr1:117864051-117864073 | 117864068 | 3 | RNA | GGCCGAGGTCGACTACCGGCNRG | GGCCGAGcTaGgC--CgGGCCGG | + | 4 | 2 | 10 | 0.00 | 5.00 |
| Chr1:106937550-106937572 | 106937567 | 3 | RNA | GGCCGAGGTCGACTACCGGCNRG | GcCCGAGGgCaACcACC--gTGG | + | 5 | 2 | 10 | 0.00 | 5.00 |
| Chr7:178519715-178519737 | 178519722 | 5 | RNA | GGCCGAGGTCGACTACCGGCNRG | GtCCG--GaaGAaTACCGGaAAG | - | 5 | 2 | 10 | 0.00 | 5.00 |
| Chr3:98731345-98731367 | 98731352 | 5 | RNA | GGCCGAGGTCGACTACCGGCNRG | GtCCG--GTgcACcACCGGaCAG | - | 5 | 2 | 10 | 0.00 | 5.00 |
| Chr1:286311129-286311151 | 286311146 | 3 | RNA | GGCCGAGGTCGACTACCGGCNRG | GGCgaAGGTtGAtaA--GGCGAG | + | 5 | 2 | 10 | 0.00 | 5.00 |
| Chr8:157911346-157911368 | 157911353 | 5 | RNA | GGCCGAGGTCGACTACCGGCNRG | G-CCtcGGgCGAgTACtGGCAGG | - | 5 | 1 | 10 | 0.00 | 5.00 |
| Chr6:53635479-53635501 | 53635496 | 3 | RNA | GGCCGAGGTCGACTACCGGCNRG | G--CGAGGgCGACgACgGctTGG | + | 5 | 2 | 10 | 0.00 | 5.00 |
| Chr6:123490784-123490806 | 123490791 | 5 | RNA | GGCCGAGGTCGACTACCGGCNRG | aGCaGAG--CGAacgCCGGCTGG | - | 5 | 2 | 10 | 0.00 | 5.00 |
| Chr10:13540353-13540375 | 13540370 | 3 | RNA | GGCCGAGGTCGACTACCGGCNRG | GttCGgGGT-GAgTACCtGCGGG | + | 5 | 1 | 10 | 0.00 | 5.00 |
| Chr2:195438829-195438851 | 195438846 | 3 | RNA | GGCCGAGGTCGACTACCGGCNRG | aaCCGAGGcaGA--ACCGcCTAG | + | 5 | 2 | 10 | 0.00 | 5.00 |
| Chr10:39102347-39102369 | 39102354 | 5 | RNA | GGCCGAGGTCGACTACCGGCNRG | GGtCGtGcTCGAC-ACgaGCTGG | - | 5 | 1 | 10 | 0.00 | 5.00 |
| Chr2:164611581-164611603 | 164611588 | 5 | RNA | GGCCGAGGTCGACTACCGGCNRG | atCCtgGGT-GACcACCGGCGGG | - | 5 | 1 | 10 | 0.00 | 5.00 |
| Chr4:45652247-45652269 | 45652254 | 5 | RNA | GGCCGAGGTCGACTACCGGCNRG | GGC--AGGcCGAgcACCGGCCGG | - | 3 | 2 | 10 | 0.00 | 5.00 |
| Chr6:97520676-97520698 | 97520693 | 3 | RNA | GGCCGAGGTCGACTACCGGCNRG | GGCCG-GGcCGtCcACtGGCGGG | + | 4 | 1 | 10 | 0.00 | 5.00 |
| Chr7:147283183-147283205 | 147283200 | 3 | RNA | GGCCGAGGTCGACTACCGGCNRG | GGC--tcGcgGAtTACCGGCTGG | + | 5 | 2 | 10 | 0.00 | 5.00 |
| Chr1:439322-439344 | 439329 | 5 | DNA | GGCCGAGGTCGACTA-CCGGCNRG | GGCCGAGGcaGcCTcGCtGGCAGG | - | 5 | 1 | 10 | 0.00 | 5.00 |
| Chr1:103542857-103542879 | 103542874 | 3 | DNA | GGC--CGAGGTCGACTACCGGCNRG | GGCGGCGAGtaCGAactCCGGCGAG | + | 5 | 2 | 10 | 0.00 | 5.00 |
| Chr6:140378297-140378319 | 140378303 | 4 | RNA | GGCCGAGGTCGACTACCGGCNRG | caCCGAtGcCGACTg--GGCGGG | - | 5 | 2 | 0 | 9.91 | 4.96 |
| Chr4:138289321-138289343 | 138289336 | 5 | DNA | GGCCGAGGTCGACTACC--GGCNRG | GGCCGAGaTCtcCTctCGAGGCCGG | + | 5 | 2 | 0 | 9.91 | 4.96 |
| Chr4:158402256-158402278 | 158402260 | 2 | RNA | GGCCGAGGTCGACTACCGGCNRG | GGaCG--GTCcACgACCtGgGAG | - | 5 | 2 | 0 | 9.91 | 4.96 |
| Chr2:204784597-204784619 | 204784612 | 5 | RNA | GGCCGAGGTCGACTACCGGCNRG | GcCgGAGccCGAC--CCGtCAAG | + | 5 | 2 | 0 | 9.91 | 4.96 |
| Chr9:124699320-124699342 | 124699326 | 4 | DNA | GGCCGAGGTCGACT--ACCGGCNRG | GGCtcAGGctGACTGGtCCGGCTGG | - | 5 | 2 | 0 | 9.91 | 4.96 |
| Chr4:73619090-73619112 | 73619106 | 4 | RNA | GGCCGAGGTCGACTACCGGCNRG | G-aCGgGGTCGACaACgaGCGGG | + | 5 | 1 | 0 | 9.91 | 4.96 |
| Chr3:174099641-174099663 | 174099657 | 4 | DNA | GGCCGAGGTCGACTACCGG-CNRG | GcCgGAGGTgGACTACaGGCtCAG | + | 5 | 1 | 0 | 9.91 | 4.96 |
| Chr3:96583646-96583668 | 96583650 | 2 | RNA | GGCCGAGGTCGACTACCGGCNRG | GGCCGAGG--GgtcAgCGaCTGG | - | 5 | 2 | 0 | 9.91 | 4.96 |
| Chr4:130192593-130192615 | 130192608 | 5 | RNA | GGCCGAGGTCGACTACCGGCNRG | GGC-GtGtTCaACTACaGGgAAG | + | 5 | 1 | 0 | 9.91 | 4.96 |
| Chr5:93537567-93537589 | 93537572 | 3 | RNA | GGCCGAGGTCGACTACCGGCNRG | GGCCGAGGcCtcCT-CaGGCTGG | - | 4 | 1 | 0 | 9.91 | 4.96 |
| Chr9:128563722-128563744 | 128563727 | 3 | DNA | GGCCGAGGT--CGACTACCGGCNRG | GGgCGAGGTTGCGACTcCtccCGAG | - | 5 | 2 | 0 | 9.91 | 4.96 |
| Chr3:170087571-170087593 | 170087577 | 4 | RNA | GGCCGAGGTCGACTACCGGCNRG | GaCCGAGG-CGACgACaaGaAGG | - | 5 | 1 | 0 | 9.91 | 4.96 |
| Chr4:144380215-144380237 | 144380233 | 2 | RNA | GGCCGAGGTCGACTACCGGCNRG | GGCCGAGGTgGgtTgCC--tGAG | + | 5 | 2 | 0 | 9.91 | 4.96 |
| Chr10:150293314-150293336 | 150293331 | 3 | DNA | GGCCGAGGT--CGACTACCGGCNRG | aGaCGAGGTTCCGACcAagGGCAGG | + | 5 | 2 | 0 | 9.91 | 4.96 |
| Chr7:152524654-152524676 | 152524669 | 5 | RNA | GGCCGAGGTCGACTACCGGCNRG | GGCaGcGGTCG--TtCCcGaGGG | + | 5 | 2 | 0 | 9.91 | 4.96 |
| Chr5:115554458-115554480 | 115554464 | 4 | RNA | GGCCGAGGTCGACTACCGGCNRG | GGCtGAGtTCGA--ACCctgGGG | - | 5 | 2 | 0 | 9.91 | 4.96 |
| Chr6:150089663-150089685 | 150089669 | 4 | RNA | GGCCGAGGTCGACTACCGGCNRG | GaCC-AGGTCGAggctCGGCGGG | - | 5 | 1 | 0 | 9.91 | 4.96 |
| Chr5:60029242-60029264 | 60029259 | 3 | RNA | GGCCGAGGTCGACTACCGGCNRG | GGCCatGGctGA--ACCcGCTAG | + | 5 | 2 | 0 | 9.91 | 4.96 |
| Chr7:67938984-67939006 | 67939002 | 2 | RNA | GGCCGAGGTCGACTACCGGCNRG | GGCaGAGGa--ACTcCgGGCCGG | + | 4 | 2 | 0 | 9.91 | 4.96 |
| Chr5:205219580-205219602 | 205219584 | 2 | RNA | GGCCGAGGTCGACTACCGGCNRG | GGC-GAaGTCGAagACCtcCCAG | - | 5 | 1 | 0 | 9.91 | 4.96 |
| Chr4:27992760-27992782 | 27992776 | 4 | RNA | GGCCGAGGTCGACTACCGGCNRG | G-aCGgGGTCGACaACgaGCGGG | + | 5 | 1 | 0 | 9.91 | 4.96 |
| Chr1:216532936-216532958 | 216532954 | 2 | RNA | GGCCGAGGTCGACTACCGGCNRG | GGCCGAacTgGgtTA--GGCTGG | + | 5 | 2 | 0 | 9.91 | 4.96 |
| Chr9:36909819-36909841 | 36909837 | 2 | RNA | GGCCGAGGTCGACTACCGGCNRG | GGgaGAGGTCaAC-ACCctCAGG | + | 5 | 1 | 0 | 9.91 | 4.96 |
| Chr5:67199808-67199830 | 67199825 | 3 | DNA | GGCCGAGGTCGACTACC--GGCNRG | GGCgGAGGTCGtCggCaGAGGCCGG | + | 5 | 2 | 1 | 8.81 | 4.91 |
| Chr4:68033576-68033598 | 68033581 | 3 | RNA | GGCCGAGGTCGACTACCGGCNRG | tGgCtAGGa-GACTACCGGCTGG | - | 4 | 1 | 7 | 2.75 | 4.88 |
| Chr1:247722181-247722203 | 247722187 | 4 | RNA | GGCCGAGGTCGACTACCGGCNRG | a-CCGtGGaCGACTACCttCCGG | - | 5 | 1 | 0 | 9.36 | 4.68 |
| Chr10:88759429-88759451 | 88759447 | 2 | DNA | GGCCGAGGTCG-ACTACCGGCNRG | GGCCGAGcaCGAACcgCCGGCTGG | + | 4 | 1 | 0 | 9.36 | 4.68 |
| Chr5:213831551-213831573 | 213831567 | 4 | RNA | GGCCGAGGTCGACTACCGGCNRG | GGC--AGGggGAgTtgCGGCCGG | + | 5 | 2 | 0 | 9.36 | 4.68 |
| Chr1:52136970-52136992 | 52136986 | 4 | RNA | GGCCGAGGTCGACTACCGGCNRG | GG--GAGcTCGgCTggCGtCCAG | + | 5 | 2 | 0 | 9.36 | 4.68 |
| Chr2:184496767-184496789 | 184496782 | 5 | RNA | GGCCGAGGTCGACTACCGGCNRG | GtCCG--GTgcACcACCGGaCAG | + | 5 | 2 | 0 | 9.36 | 4.68 |
| Chr1:228514745-228514767 | 228514751 | 4 | RNA | GGCCGAGGTCGACTACCGGCNRG | GGCCGAGGcgGA-TctCGtCGAG | - | 5 | 1 | 0 | 9.36 | 4.68 |
| Chr1:2293685-2293707 | 2293702 | 3 | RNA | GGCCGAGGTCGACTACCGGCNRG | G-CCcAGGTgGACgAtCtGCTGG | + | 5 | 1 | 0 | 9.36 | 4.68 |
| Chr10:16554047-16554069 | 16554063 | 4 | RNA | GGCCGAGGTCGACTACCGGCNRG | G-aCGgGGTCGACaACgaGCGGG | + | 5 | 1 | 0 | 9.36 | 4.68 |
| Chr9:143499986-143500008 | 143500004 | 2 | RNA | GGCCGAGGTCGACTACCGGCNRG | GG--GAcGgCGAgTgCCGaCGAG | + | 5 | 2 | 0 | 9.36 | 4.68 |
| Chr1:158090635-158090657 | 158090653 | 2 | RNA | GGCCGAGGTCGACTACCGGCNRG | GGgCGAGGgCatgTAC-GGCTGG | + | 5 | 1 | 0 | 9.36 | 4.68 |
| Chr1:154432586-154432608 | 154432604 | 2 | RNA | GGCCGAGGTCGACTACCGGCNRG | GGCCGAG--CGctgACaGGCGGG | + | 4 | 2 | 0 | 9.36 | 4.68 |
| Chr2:161607900-161607922 | 161607905 | 3 | RNA | GGCCGAGGTCGACTACCGGCNRG | GGCCGAGGctGAaTtC--GgGGG | - | 5 | 2 | 0 | 9.36 | 4.68 |
| Chr4:64043402-64043424 | 64043408 | 4 | RNA | GGCCGAGGTCGACTACCGGCNRG | GGgCGgGGTtGgCT--CGGgGAG | - | 5 | 2 | 0 | 9.36 | 4.68 |
| Chr1:146058732-146058754 | 146058748 | 4 | RNA | GGCCGAGGTCGACTACCGGCNRG | G-aCGtGGTCGACaACaaGCGGG | + | 5 | 1 | 0 | 9.36 | 4.68 |
| Chr8:22695348-22695370 | 22695364 | 4 | RNA | GGCCGAGGTCGACTACCGGCNRG | GGCCaAGGaaGACcAaC--CAAG | + | 5 | 2 | 0 | 9.36 | 4.68 |
| Chr1:3849157-3849179 | 3849172 | 5 | RNA | GGCCGAGGTCGACTACCGGCNRG | GtaCGtGtTC--CTACCGGCCGG | + | 4 | 2 | 0 | 9.36 | 4.68 |
| Chr4:238876534-238876556 | 238876539 | 3 | RNA | GGCCGAGGTCGACTACCGGCNRG | GGCCGAGtaCG--TtgCcGCAGG | - | 5 | 2 | 0 | 9.36 | 4.68 |
| Chr8:78722620-78722642 | 78722638 | 2 | RNA | GGCCGAGGTCGACTACCGGCNRG | tcCCGAG--CagCTAaCGGCGAG | + | 5 | 2 | 0 | 9.36 | 4.68 |
| Chr4:144606847-144606869 | 144606865 | 2 | RNA | GGCCGAGGTCGACTACCGGCNRG | GaCCGAGG-CGACgACaaGaAGG | + | 5 | 1 | 0 | 9.36 | 4.68 |
| Chr5:78742960-78742982 | 78742978 | 2 | RNA | GGCCGAGGTCGACTACCGGCNRG | c--CGAGcTgGACTtCgGGCGAG | + | 5 | 2 | 0 | 9.36 | 4.68 |
| Chr8:96590069-96590091 | 96590074 | 3 | X | GGCCGAGGTCGACTACCGGCNRG | GcCCaAGGaCGACatCCGGCGGG | - | 5 | 0 | 0 | 9.36 | 4.68 |
| Chr1:163321856-163321878 | 163321874 | 2 | RNA | GGCCGAGGTCGACTACCGGCNRG | tGCtGAGGTtGACTgCC--tGAG | + | 5 | 2 | 0 | 9.36 | 4.68 |
| Chr2:99680837-99680859 | 99680854 | 3 | RNA | GGCCGAGGTCGACTACCGGCNRG | GGCCaAG-TCcACgAaCGaCCAG | + | 5 | 1 | 0 | 9.36 | 4.68 |
| Chr2:231931085-231931107 | 231931091 | 4 | RNA | GGCCGAGGTCGACTACCGGCNRG | ttCCGAtGTCGAtTg--GGCAGG | - | 5 | 2 | 0 | 9.36 | 4.68 |
| Chr5:61784087-61784109 | 61784093 | 4 | RNA | GGCCGAGGTCGACTACCGGCNRG | G-aCGgGGTCGACcACgaGCGGG | - | 5 | 1 | 0 | 9.36 | 4.68 |
| Chr1:246411365-246411387 | 246411370 | 3 | RNA | GGCCGAGGTCGACTACCGGCNRG | GGCCaAttTCGcCTtC--GCCGG | - | 5 | 2 | 0 | 9.36 | 4.68 |
| Chr9:87557604-87557626 | 87557608 | 2 | DNA | GGCCGAG-GTCGACTACCGGCNRG | GttCGAGCGTCaACgAtCGGCAAG | - | 5 | 1 | 0 | 9.36 | 4.68 |
| Chr7:138229160-138229182 | 138229176 | 4 | RNA | GGCCGAGGTCGACTACCGGCNRG | cGCCGtGaTCG--cACCaGCCAG | + | 5 | 2 | 0 | 9.36 | 4.68 |
| Chr4:139245577-139245599 | 139245582 | 3 | RNA | GGCCGAGGTCGACTACCGGCNRG | GagCGAG--aGACaACCGGCTGG | - | 4 | 2 | 0 | 9.36 | 4.68 |
| Chr3:98763891-98763913 | 98763908 | 3 | RNA | GGCCGAGGTCGACTACCGGCNRG | cGaCGgGGTCGgCT--CGGCTGG | + | 4 | 2 | 0 | 9.36 | 4.68 |
| Chr8:69628163-69628185 | 69628181 | 2 | DNA | GGCCGAGGTCGA--CTACCGGCNRG | GcgCcAtcTCGATTCTACCGGCGAG | + | 5 | 2 | 0 | 9.36 | 4.68 |
| Chr7:11948366-11948388 | 11948372 | 4 | RNA | GGCCGAGGTCGACTACCGGCNRG | GtCCG--GTgcACcACCGGaTAG | - | 5 | 2 | 0 | 9.36 | 4.68 |
| Chr7:180826397-180826419 | 180826403 | 4 | DNA | GGCCGAGGTCGACTACC-GGCNRG | GGCCaAGGgCctCaACCAGGCCAG | - | 5 | 1 | 0 | 9.36 | 4.68 |
| Chr7:141948844-141948866 | 141948848 | 2 | RNA | GGCCGAGGTCGACTACCGGCNRG | GtCCGAGGTC--CgAgCGGaGAG | - | 4 | 2 | 0 | 9.36 | 4.68 |
| Chr1:7417887-7417909 | 7417891 | 2 | RNA | GGCCGAGGTCGACTACCGGCNRG | GGC-GcGGTCGAggACtGGtGGG | - | 5 | 1 | 0 | 9.36 | 4.68 |
| Chr6:118300599-118300621 | 118300605 | 4 | RNA | GGCCGAGGTCGACTACCGGCNRG | GGC--tcGcgGACTACCGGtTGG | - | 5 | 2 | 0 | 9.36 | 4.68 |
| Chr3:77262867-77262889 | 77262883 | 4 | RNA | GGCCGAGGTCGACTACCGGCNRG | G-aCGgGGTCGACaACgaGCGGG | + | 5 | 1 | 0 | 9.36 | 4.68 |
| Chr9:27097749-27097771 | 27097766 | 3 | X | GGCCGAGGTCGACTACCGGCNRG | GGaCGAcGaCGACcACCaGCAGG | + | 5 | 0 | 0 | 9.36 | 4.68 |
| Chr4:151090887-151090909 | 151090891 | 2 | RNA | GGCCGAGGTCGACTACCGGCNRG | GGCCGAGcaCcACgAC--GCCAG | - | 4 | 2 | 0 | 9.36 | 4.68 |
| Chr4:141042986-141043008 | 141043002 | 4 | RNA | GGCCGAGGTCGACTACCGGCNRG | G-aCGgGGTCGACaACgaGCGGG | + | 5 | 1 | 1 | 8.26 | 4.63 |
| Chr2:40890230-40890252 | 40890248 | 2 | DNA | GGCC-GAGGTCGACTACCGGCNRG | GcCCCGAGcTatACTACCGGCGGG | + | 4 | 1 | 3 | 6.06 | 4.53 |
| Chr3:227789873-227789895 | 227789878 | 3 | RNA | GGCCGAGGTCGACTACCGGCNRG | GGCacAGGTCGtCgAa-GGCAAG | - | 5 | 1 | 3 | 6.06 | 4.53 |
| Chr1:20944079-20944101 | 20944096 | 3 | RNA | GGCCGAGGTCGACTACCGGCNRG | GGC--gGGcCaACctCCGGCCGG | + | 5 | 2 | 9 | 0.00 | 4.50 |
| Chr10:53678961-53678983 | 53678978 | 3 | RNA | GGCCGAGGTCGACTACCGGCNRG | GGC--tcGcgGACTACCGGCTGG | + | 4 | 2 | 9 | 0.00 | 4.50 |
| Chr7:69146911-69146933 | 69146918 | 5 | RNA | GGCCGAGGTCGACTACCGGCNRG | GGCtGgGcTgGgCTA--GGCTGG | - | 5 | 2 | 9 | 0.00 | 4.50 |
| Chr3:57766932-57766954 | 57766949 | 3 | RNA | GGCCGAGGTCGACTACCGGCNRG | cGCatAGGaC-ACTACaGGCAGG | + | 5 | 1 | 9 | 0.00 | 4.50 |
| Chr7:58504461-58504483 | 58504478 | 3 | RNA | GGCCGAGGTCGACTACCGGCNRG | GGCCGAtcTCG--cAtCaGCAAG | + | 5 | 2 | 9 | 0.00 | 4.50 |
| Chr7:113839294-113839316 | 113839311 | 3 | RNA | GGCCGAGGTCGACTACCGGCNRG | aaCCGAGGTtGgCTA--GGtCAG | + | 5 | 2 | 9 | 0.00 | 4.50 |
| Chr2:79782349-79782371 | 79782366 | 3 | RNA | GGCCGAGGTCGACTACCGGCNRG | GGgCG-GGcCaACccCCGGCCGG | + | 5 | 1 | 9 | 0.00 | 4.50 |
| Chr9:149956927-149956949 | 149956944 | 3 | RNA | GGCCGAGGTCGACTACCGGCNRG | GGCtGgGGTaG--cACCGGgAGG | + | 5 | 2 | 9 | 0.00 | 4.50 |
| Chr6:40897005-40897027 | 40897012 | 5 | RNA | GGCCGAGGTCGACTACCGGCNRG | t--CGAGGTCaACctCaGGCGGG | - | 5 | 2 | 9 | 0.00 | 4.50 |
| Chr6:98810749-98810771 | 98810756 | 5 | RNA | GGCCGAGGTCGACTACCGGCNRG | GGgCGAGcTCGACTAg-GGCTGG | - | 3 | 1 | 9 | 0.00 | 4.50 |
| Chr10:73879415-73879437 | 73879432 | 3 | RNA | GGCCGAGGTCGACTACCGGCNRG | GGCCGtttTC--CcACtGGCCGG | + | 5 | 2 | 9 | 0.00 | 4.50 |
| Chr4:65946711-65946733 | 65946718 | 5 | RNA | GGCCGAGGTCGACTACCGGCNRG | GGCttgGGa--ACTACCGGaAGG | - | 5 | 2 | 9 | 0.00 | 4.50 |
| Chr8:132764657-132764679 | 132764664 | 5 | RNA | GGCCGAGGTCGACTACCGGCNRG | GGC--AGGcgGAgcACCGGCCGG | - | 4 | 2 | 9 | 0.00 | 4.50 |
| Chr5:160726065-160726087 | 160726082 | 3 | RNA | GGCCGAGGTCGACTACCGGCNRG | tGC--AaGTacACcACCGGCCGG | + | 5 | 2 | 9 | 0.00 | 4.50 |
| Chr2:216933376-216933398 | 216933393 | 3 | RNA | GGCCGAGGTCGACTACCGGCNRG | GGCCGAGGTgcAtgAC--GaAGG | + | 5 | 2 | 9 | 0.00 | 4.50 |
| Chr1:3030226-3030248 | 3030233 | 5 | RNA | GGCCGAGGTCGACTACCGGCNRG | GGaCcAGtTCGACTA--aGtTAG | - | 5 | 2 | 9 | 0.00 | 4.50 |
| Chr3:162654656-162654678 | 162654673 | 3 | DNA | GGCCGAGG-TCGACTACCGGCNRG | tGCCGAGGGTtGgCcAgCGGCCGG | + | 5 | 1 | 9 | 0.00 | 4.50 |
| Chr2:183134698-183134720 | 183134705 | 5 | RNA | GGCCGAGGTCGACTACCGGCNRG | GcCCGAGcTtGtCT--CGGaCAG | - | 5 | 2 | 9 | 0.00 | 4.50 |
| Chr7:59259610-59259632 | 59259627 | 3 | DNA | GGCC-GAGGTCGACTACCGGCNRG | GGCCCGAGGTgGAagACgGcCAGG | + | 5 | 1 | 9 | 0.00 | 4.50 |
| Chr1:59065802-59065824 | 59065809 | 5 | RNA | GGCCGAGGTCGACTACCGGCNRG | GGC--tcGcgGACTAtCGGCTGG | - | 5 | 2 | 9 | 0.00 | 4.50 |
| Chr3:32260847-32260869 | 32260854 | 5 | RNA | GGCCGAGGTCGACTACCGGCNRG | GGgCGAG--CGctgACaGGCAGG | - | 5 | 2 | 9 | 0.00 | 4.50 |
| Chr1:228816797-228816819 | 228816814 | 3 | RNA | GGCCGAGGTCGACTACCGGCNRG | GGCCGAGtaCG--TtgCcGCAGG | + | 5 | 2 | 9 | 0.00 | 4.50 |
| Chr2:213671633-213671655 | 213671650 | 3 | RNA | GGCCGAGGTCGACTACCGGCNRG | GGgCG-GGcCaACctCCGGCCGG | + | 5 | 1 | 9 | 0.00 | 4.50 |
| Chr8:129460902-129460924 | 129460919 | 3 | RNA | GGCCGAGGTCGACTACCGGCNRG | GcCCGAGGgCaACcACC--aTGG | + | 5 | 2 | 9 | 0.00 | 4.50 |
| Chr8:53789705-53789727 | 53789722 | 3 | RNA | GGCCGAGGTCGACTACCGGCNRG | GGCCGAGGTCGgC--CCaGgGAG | + | 3 | 2 | 9 | 0.00 | 4.50 |
| Chr5:7822135-7822157 | 7822142 | 5 | DNA | GGCCGAGGTCGACTACCGG--CNRG | GGtCGAGGTCGAgTcCaaGCCCCAG | - | 5 | 2 | 9 | 0.00 | 4.50 |
| Chr5:42726464-42726486 | 42726481 | 3 | RNA | GGCCGAGGTCGACTACCGGCNRG | G-aCGgGGTCGACaACgaGCGGG | + | 5 | 1 | 9 | 0.00 | 4.50 |
| Chr3:158374613-158374635 | 158374620 | 5 | RNA | GGCCGAGGTCGACTACCGGCNRG | GGggGAGGTgG--TAgaGGCTGG | - | 5 | 2 | 9 | 0.00 | 4.50 |
| Chr2:159474844-159474866 | 159474861 | 3 | RNA | GGCCGAGGTCGACTACCGGCNRG | cGgCGAGGTCGcCTg--GGtCGG | + | 5 | 2 | 9 | 0.00 | 4.50 |
| Chr10:126724882-126724904 | 126724899 | 3 | RNA | GGCCGAGGTCGACTACCGGCNRG | GGC--tcGcgGAtTACCGGCTGG | + | 5 | 2 | 9 | 0.00 | 4.50 |
| Chr3:13873822-13873844 | 13873839 | 3 | RNA | GGCCGAGGTCGACTACCGGCNRG | GGCgGAccTC--CTAgCGaCGGG | + | 5 | 2 | 9 | 0.00 | 4.50 |
| Chr4:199352157-199352179 | 199352164 | 5 | RNA | GGCCGAGGTCGACTACCGGCNRG | GaCCGAGtgCtACTACCt-CCGG | - | 5 | 1 | 9 | 0.00 | 4.50 |
| Chr9:146062728-146062750 | 146062735 | 5 | DNA | GGCCGAGGTCGAC--TACCGGCNRG | cGCCGAGaTCGACGCTgCgGtCGGG | - | 5 | 2 | 9 | 0.00 | 4.50 |
| Chr7:135866544-135866566 | 135866561 | 3 | RNA | GGCCGAGGTCGACTACCGGCNRG | GGCCGgGGgCGgCTg--GGCCGG | + | 4 | 2 | 9 | 0.00 | 4.50 |
| Chr1:129947164-129947186 | 129947171 | 5 | RNA | GGCCGAGGTCGACTACCGGCNRG | GGaatAGGTC-ACcACCGaCGAG | - | 5 | 1 | 9 | 0.00 | 4.50 |
| Chr10:148670436-148670458 | 148670453 | 3 | RNA | GGCCGAGGTCGACTACCGGCNRG | cG--GAGGcCGgCgACCGGaAGG | + | 5 | 2 | 9 | 0.00 | 4.50 |
| Chr3:202339574-202339596 | 202339581 | 5 | RNA | GGCCGAGGTCGACTACCGGCNRG | GGCtcAGG-CagCTACtGGCTGG | - | 5 | 1 | 9 | 0.00 | 4.50 |
| Chr2:41347030-41347052 | 41347047 | 3 | RNA | GGCCGAGGTCGACTACCGGCNRG | GGaCtAGGTCG--TcCCaGtTGG | + | 5 | 2 | 9 | 0.00 | 4.50 |
| Chr8:150902144-150902166 | 150902151 | 5 | RNA | GGCCGAGGTCGACTACCGGCNRG | GtCttgGGT-GACcACCGGCGGG | - | 5 | 1 | 9 | 0.00 | 4.50 |
| Chr2:57503831-57503853 | 57503848 | 3 | RNA | GGCCGAGGTCGACTACCGGCNRG | GGttGAGaT--AaTACCGtCTGG | + | 5 | 2 | 9 | 0.00 | 4.50 |
| Chr4:50364334-50364356 | 50364351 | 3 | RNA | GGCCGAGGTCGACTACCGGCNRG | G-CCGAGGTCGAgcACatGgTGG | + | 5 | 1 | 9 | 0.00 | 4.50 |
| Chr5:200279639-200279661 | 200279646 | 5 | RNA | GGCCGAGGTCGACTACCGGCNRG | G-CCGAaGTgcACTgCCtGCTGG | - | 5 | 1 | 9 | 0.00 | 4.50 |

| | | | | | | | | | | |
|---|---|---|---|---|---|---|---|---|---|---|
| Chr2:202024331-202024353 | 202024348 | 3 | RNA | GGCCGAGGTCGACTACCGGCNRG | GGC--tcGcgGACTACCGtCTGG | + | 5 | 2 | 9 | 0.00 | 4.50 |
| Chr5:209656316-209656338 | 209656322 | 4 | RNA | GGCCGAGGTCGACTACCGGCNRG | GGCCtcGaTCGtCTt--GGCCAG | - | 5 | 2 | 0 | 8.81 | 4.41 |
| Chr7:73014299-73014321 | 73014304 | 3 | RNA | GGCCGAGGTCGACTACCGGCNRG | aGCCGAGGg-GACgACCtGCTGG | - | 4 | 1 | 0 | 8.81 | 4.41 |
| Chr10:83133264-83133286 | 83133279 | 5 | RNA | GGCCGAGGTCGACTACCGGCNRG | G--CtAGGTCGcggACCGtCCGG | + | 5 | 2 | 0 | 8.81 | 4.41 |
| Chr5:188279279-188279301 | 188279284 | 3 | DNA | GGCCGAGGTCG-ACTACCGGCNRG | cGaCGtGGgCGCACcACCGGCCGG | - | 5 | 1 | 0 | 8.81 | 4.41 |
| Chr10:11351215-11351237 | 11351220 | 3 | RNA | GGCCGAGGTCGACTACCGGCNRG | GGCtG-cGTCGcCggCCGGCGGG | - | 5 | 1 | 0 | 8.81 | 4.41 |
| Chr8:107236058-107236080 | 107236063 | 3 | RNA | GGCCGAGGTCGACTACCGGCNRG | GGC--tcGcgGACTACCGGCTGG | - | 4 | 2 | 0 | 8.81 | 4.41 |
| Chr7:92808129-92808151 | 92808147 | 2 | RNA | GGCCGAGGTCGACTACCGGCNRG | GcCCaAtGcCaACTACC--CAGG | + | 5 | 2 | 0 | 8.81 | 4.41 |
| Chr6:112250306-112250328 | 112250324 | 2 | RNA | GGCCGAGGTCGACTACCGGCNRG | GtCCG--GTgcACcACCGGaCAG | + | 5 | 2 | 0 | 8.81 | 4.41 |
| Chr5:147641274-147641296 | 147641291 | 3 | X | GGCCGAGGTCGACTACCGGCNRG | GGaCGAGGTaGACcAgCaGCAGG | + | 5 | 0 | 0 | 8.81 | 4.41 |
| Chr4:176279491-176279513 | 176279498 | 5 | RNA | GGCCGAGGTCGACTACCGGCNRG | GtCCG--GTgcACcACCGGaCAG | - | 5 | 2 | 0 | 8.81 | 4.41 |
| Chr8:78639353-78639375 | 78639358 | 3 | RNA | GGCCGAGGTCGACTACCGGCNRG | G-aCGgGGTCGACgACgaGCGGG | - | 5 | 1 | 0 | 8.81 | 4.41 |
| Chr1:5942859-5942881 | 5942865 | 4 | RNA | GGCCGAGGTCGACTACCGGCNRG | G-CCGAcGgCGgCTACCGGCAGG | - | 3 | 1 | 0 | 8.81 | 4.41 |
| Chr1:231826861-231826883 | 231826877 | 4 | RNA | GGCCGAGGTCGACTACCGGCNRG | ttCCGActTCGACTA--tGCTGG | + | 5 | 2 | 0 | 8.81 | 4.41 |
| Chr1:160764689-160764711 | 160764704 | 5 | RNA | GGCCGAGGTCGACTACCGGCNRG | cGCCGccGTCGgCTA--GtCTGG | + | 5 | 2 | 0 | 8.81 | 4.41 |
| Chr5:85731455-85731477 | 85731471 | 4 | RNA | GGCCGAGGTCGACTACCGGCNRG | G-aCGgGGTCGACaACgaGCGGG | + | 5 | 1 | 0 | 8.81 | 4.41 |
| Chr2:193924808-193924830 | 193924814 | 4 | RNA | GGCCGAGGTCGACTACCGGCNRG | GGCCG-GGcCatCcACtGGCGGG | - | 5 | 1 | 0 | 8.81 | 4.41 |
| Chr8:134545370-134545392 | 134545377 | 5 | RNA | GGCCGAGGTCGACTACCGGCNRG | GGCCGAGGatGcCT-CgGGCTGG | - | 4 | 1 | 0 | 8.81 | 4.41 |
| Chr4:109407677-109407699 | 109407684 | 5 | RNA | GGCCGAGGTCGACTACCGGCNRG | GGCCaAtcTCG--TAgCaGCAAG | - | 5 | 2 | 0 | 8.81 | 4.41 |
| Chr7:54629060-54629082 | 54629076 | 4 | RNA | GGCCGAGGTCGACTACCGGCNRG | GGC-GAGGTCGgCcACCaGgGAG | + | 4 | 1 | 0 | 8.81 | 4.41 |
| Chr4:101066696-101066718 | 101066713 | 3 | RNA | GGCCGAGGTCGACTACCGGCNRG | GcCCGAGGcCGA--ACtcGtGGG | + | 5 | 2 | 0 | 8.81 | 4.41 |
| Chr7:100980934-100980956 | 100980951 | 3 | RNA | GGCCGAGGTCGACTACCGGCNRG | aaCaGAGGT--ACTACtGGaGAG | + | 5 | 2 | 0 | 8.81 | 4.41 |
| Chr2:205897453-205897475 | 205897471 | 2 | RNA | GGCCGAGGTCGACTACCGGCNRG | GaCCGAGG-CGACgACaaGaAGG | + | 5 | 1 | 0 | 8.81 | 4.41 |
| Chr3:83773525-83773547 | 83773532 | 5 | RNA | GGCCGAGGTCGACTACCGGCNRG | tGCgGAGG--GAgTACgGcCGAG | - | 5 | 2 | 0 | 8.81 | 4.41 |
| Chr7:5414793-5414815 | 5414808 | 5 | RNA | GGCCGAGGTCGACTACCGGCNRG | GaC--tGGctaACTACCGGCAGG | + | 5 | 2 | 0 | 8.81 | 4.41 |
| Chr4:182848845-182848867 | 182848852 | 5 | RNA | GGCCGAGGTCGACTACCGGCNRG | caCCaAGtTCGcCT-CCGGCCAG | - | 5 | 1 | 0 | 8.81 | 4.41 |
| Chr1:266828847-266828869 | 266828864 | 3 | RNA | GGCCGAGGTCGACTACCGGCNRG | GGgCGAGGTaGcCgA--GGCTGG | + | 4 | 2 | 0 | 8.81 | 4.41 |
| Chr8:165249561-165249583 | 165249568 | 5 | RNA | GGCCGAGGTCGACTACCGGCNRG | GGCCa--GTCGACTAtgGGgTGG | - | 4 | 2 | 0 | 8.81 | 4.41 |
| Chr2:143611350-143611372 | 143611357 | 5 | RNA | GGCCGAGGTCGACTACCGGCNRG | GtCCG--GTgcACcACCGGaCAG | - | 5 | 2 | 0 | 8.81 | 4.41 |
| Chr1:17167918-17167940 | 17167933 | 5 | DNA | G-GCCGAGGTCGACTACCGGCNRG | GCGCCcAGGTCGcggACCGtCCGG | + | 5 | 1 | 0 | 8.81 | 4.41 |
| Chr3:141127837-141127859 | 141127842 | 3 | RNA | GGCCGAGGTCGACTACCGGCNRG | GGCCGAGtaCG--TtgCcGCAGG | - | 5 | 2 | 0 | 8.81 | 4.41 |
| Chr1:121275645-121275667 | 121275652 | 5 | RNA | GGCCGAGGTCGACTACCGGCNRG | GG--GAGcaCGAactCCGGCGAG | - | 5 | 2 | 7 | 1.65 | 4.33 |
| Chr1:27905964-27905986 | 27905980 | 4 | RNA | GGCCGAGGTCGACTACCGGCNRG | G-aCGgGGTCGACgACgaGCGGG | + | 5 | 1 | 8 | 0.55 | 4.28 |
| Chr4:67266523-67266545 | 67266541 | 2 | DNA | GGCCGAGGT-CGACTACCGGCNRG | cGgCGAGGTACGAactCCGGCGAG | + | 5 | 1 | 8 | 0.55 | 4.28 |
| Chr8:96868831-96868853 | 96868848 | 3 | RNA | GGCCGAGGTCGACTACCGGCNRG | cGCCtAGGTCGgCTgC--GCGAG | + | 4 | 2 | 3 | 5.51 | 4.26 |
| Chr2:91712420-91712442 | 91712425 | 3 | DNA | GGCCGAGGTCGACTA--CCGGCNRG | ctCCaAGGcCGACcAATCCGGCAGG | - | 5 | 2 | 3 | 5.51 | 4.26 |
| Chr3:176910066-176910088 | 176910072 | 4 | RNA | GGCCGAGGTCGACTACCGGCNRG | GGgCGAGcTCGACTAg-GGCTGG | - | 3 | 1 | 5 | 3.30 | 4.15 |
| Chr10:96088780-96088802 | 96088786 | 4 | X | GGCCGAGGTCGACTACCGGCNRG | GaCCGAGGggGAaTACCcGCTGG | - | 5 | 0 | 5 | 3.30 | 4.15 |
| Chr6:83243418-83243440 | 83243425 | 5 | DNA | GGCCGAGGTCGACTAC--CGGCNRG | GGCgGgtGTtGAaTACTACGGCTAG | - | 5 | 2 | 0 | 8.26 | 4.13 |
| Chr8:126721138-126721160 | 126721144 | 4 | DNA | GGCCGAGGTCGAC-TACCGGCNRG | GGCCGAtGTCGtCGaAgCaGCAGG | - | 5 | 1 | 0 | 8.26 | 4.13 |
| Chr8:78342334-78342356 | 78342339 | 3 | RNA | GGCCGAGGTCGACTACCGGCNRG | G-aCGgGGTCGACaACgaGCGGG | - | 5 | 1 | 0 | 8.26 | 4.13 |
| Chr10:23777922-23777944 | 23777928 | 4 | RNA | GGCCGAGGTCGACTACCGGCNRG | GGaCcAGGTCG--TcCCaGtTGG | - | 5 | 2 | 0 | 8.26 | 4.13 |
| Chr8:84425660-84425682 | 84425675 | 5 | RNA | GGCCGAGGTCGACTACCGGCNRG | G--aGcGGgaGACgACCGGCTGG | + | 5 | 2 | 0 | 8.26 | 4.13 |
| Chr5:121247559-121247581 | 121247574 | 5 | RNA | GGCCGAGGTCGACTACCGGCNRG | tGaCGAGGctG--TAgCGGCAGG | + | 5 | 2 | 0 | 8.26 | 4.13 |
| Chr10:56056617-56056639 | 56056634 | 3 | RNA | GGCCGAGGTCGACTACCGGCNRG | GGaCGgGcTCGACa--CGGtCGG | + | 5 | 2 | 0 | 8.26 | 4.13 |
| Chr10:92262059-92262081 | 92262064 | 3 | RNA | GGCCGAGGTCGACTACCGGCNRG | GGC--AGGcCGAgcACCGGCCGG | - | 3 | 2 | 0 | 8.26 | 4.13 |
| Chr7:160511260-160511282 | 160511277 | 3 | RNA | GGCCGAGGTCGACTACCGGCNRG | GaCCGAGGgaGAgTACt--CAGG | + | 5 | 2 | 0 | 8.26 | 4.13 |
| Chr1:126987078-126987100 | 126987096 | 2 | RNA | GGCCGAGGTCGACTACCGGCNRG | GGC--AGGgCtACcACCcGgGGG | + | 5 | 2 | 0 | 8.26 | 4.13 |
| Chr2:3657200-3657222 | 3657216 | 4 | RNA | GGCCGAGGTCGACTACCGGCNRG | G-CCGAGGcCaACctCgGGCGGG | + | 5 | 1 | 0 | 8.26 | 4.13 |
| Chr4:81670489-81670511 | 81670507 | 2 | RNA | GGCCGAGGTCGACTACCGGCNRG | GGCaGAGGT-GAgcACCaGgGAG | + | 5 | 1 | 0 | 8.26 | 4.13 |
| Chr7:83863920-83863942 | 83863924 | 2 | RNA | GGCCGAGGTCGACTACCGGCNRG | GcCCc--GTCGAgctCCGGCGAG | - | 5 | 2 | 0 | 8.26 | 4.13 |
| Chr1:8847411-8847433 | 8847417 | 4 | RNA | GGCCGAGGTCGACTACCGGCNRG | GGaCGAGGaCGAtgAC--GaCGG | - | 5 | 2 | 0 | 8.26 | 4.13 |
| Chr1:168799184-168799206 | 168799201 | 3 | DNA | GGC-CGAGGTCGACTACCGGCNRG | GcCTCGAGGaCGtCTtCCGGCTGG | + | 4 | 1 | 0 | 8.26 | 4.13 |
| Chr3:28133551-28133573 | 28133567 | 4 | RNA | GGCCGAGGTCGACTACCGGCNRG | GGaCGgGGTCa--gACCGGCCGG | + | 4 | 2 | 0 | 8.26 | 4.13 |
| Chr10:85106046-85106068 | 85106053 | 5 | RNA | GGCCGAGGTCGACTACCGGCNRG | GGC--AGGcCaACctCCGGCCGG | - | 4 | 2 | 0 | 8.26 | 4.13 |
| Chr1:93460470-93460492 | 93460477 | 5 | RNA | GGCCGAGGTCGACTACCGGCNRG | GGgCGAc--CGAagtCCGGCGAG | - | 5 | 2 | 0 | 8.26 | 4.13 |
| Chr10:87882332-87882354 | 87882347 | 5 | RNA | GGCCGAGGTCGACTACCGGCNRG | GGCtcAcG--GACTACCGGtTGG | + | 4 | 2 | 0 | 8.26 | 4.13 |
| Chr4:228199996-228200018 | 228200011 | 5 | RNA | GGCCGAGGTCGACTACCGGCNRG | GGCgGAGaTCGtCTtCC--gTGG | + | 5 | 2 | 0 | 8.26 | 4.13 |
| Chr5:148957471-148957493 | 148957477 | 4 | RNA | GGCCGAGGTCGACTACCGGCNRG | GGtgGAGGcCGcCTgCC--CCGG | - | 5 | 2 | 0 | 8.26 | 4.13 |
| Chr6:96825790-96825812 | 96825797 | 5 | DNA | GGC-CGAGGTCGACTACCGGCNRG | GcCTCGgGGaCGtCTtCCGGCTGG | - | 5 | 1 | 0 | 8.26 | 4.13 |
| Chr9:25819071-25819093 | 25819086 | 5 | RNA | GGCCGAGGTCGACTACCGGCNRG | GGC--ttGcgGACTAtCGGCTGG | + | 5 | 2 | 0 | 8.26 | 4.13 |
| Chr1:286677288-286677310 | 286677304 | 4 | RNA | GGCCGAGGTCGACTACCGGCNRG | cGCCGAGG-CaACTAgtGcCCAG | + | 5 | 1 | 0 | 8.26 | 4.13 |
| Chr5:190521127-190521149 | 190521134 | 5 | RNA | GGCCGAGGTCGACTACCGGCNRG | GcCgGAGccCGAC--CCGtCAGG | - | 5 | 2 | 0 | 8.26 | 4.13 |
| Chr5:86294324-86294346 | 86294339 | 5 | RNA | GGCCGAGGTCGACTACCGGCNRG | tGgCt-cGTgGACTACCGGCTGG | + | 5 | 1 | 0 | 8.26 | 4.13 |
| Chr6:130743476-130743498 | 130743494 | 2 | RNA | GGCCGAGGTCGACTACCGGCNRG | G-CCGgGtTCGACTcCgGGtCAG | + | 5 | 1 | 0 | 8.26 | 4.13 |
| Chr4:128461665-128461687 | 128461670 | 3 | RNA | GGCCGAGGTCGACTACCGGCNRG | GGaCGAGGag--CaACgGGCGAG | - | 5 | 2 | 0 | 8.26 | 4.13 |
| Chr5:129459716-129459738 | 129459732 | 4 | RNA | GGCCGAGGTCGACTACCGGCNRG | G-aCGgGGTCGACaACgaGCGGG | + | 5 | 1 | 0 | 8.26 | 4.13 |
| Chr1:274397528-274397550 | 274397532 | 2 | RNA | GGCCGAGGTCGACTACCGGCNRG | GGCC-AGGcCGcCggCCGGCCGG | - | 4 | 1 | 0 | 8.26 | 4.13 |
| Chr10:37323842-37323864 | 37323857 | 5 | RNA | GGCCGAGGTCGACTACCGGCNRG | GGCgGccGcCG--cACCGGCTGG | + | 5 | 2 | 0 | 8.26 | 4.13 |
| Chr9:12599199-12599221 | 12599206 | 5 | RNA | GGCCGAGGTCGACTACCGGCNRG | GGCCGccGT-GAgTACaGGtGAG | - | 5 | 1 | 0 | 8.26 | 4.13 |
| Chr7:80448651-80448673 | 80448669 | 2 | DNA | GGC--CGAGGTCGACTACCGGCNRG | GaCAACGgGGTCGACgACgaGCGGG | + | 5 | 2 | 0 | 8.26 | 4.13 |
| Chr8:95012880-95012902 | 95012886 | 4 | RNA | GGCCGAGGTCGACTACCGGCNRG | GtCCtcGG--GAtaACCGGCAGG | - | 5 | 2 | 0 | 8.26 | 4.13 |
| Chr8:142802791-142802813 | 142802796 | 3 | RNA | GGCCGAGGTCGACTACCGGCNRG | G-aCGAGGTCaACaACgaGCGGG | - | 5 | 1 | 0 | 8.26 | 4.13 |
| Chr3:28930689-28930711 | 28930707 | 2 | RNA | GGCCGAGGTCGACTACCGGCNRG | GtCCG--GTgcACcACCGGaTAG | + | 5 | 2 | 0 | 8.26 | 4.13 |
| Chr3:89804446-89804468 | 89804452 | 4 | RNA | GGCCGAGGTCGACTACCGGCNRG | GGC--ttGTgGACTACCGGCTGG | - | 3 | 2 | 0 | 8.26 | 4.13 |
| Chr2:60165521-60165543 | 60165538 | 3 | DNA | G--GCCGAGGTCGACTACCGGCNRG | GATGaCGgGGTCGACaACgaGCGGG | + | 5 | 2 | 0 | 8.26 | 4.13 |
| Chr4:237728625-237728647 | 237728643 | 2 | RNA | GGCCGAGGTCGACTACCGGCNRG | GGgCGAGGgCt--TgCCGGtGAG | + | 5 | 2 | 0 | 8.26 | 4.13 |
| Chr6:162934695-162934717 | 162934701 | 4 | DNA | GGC-CGAGGTCGACTACCGGCNRG | GcCTCGAGGaCatCTtCCGGCTGG | - | 5 | 1 | 0 | 8.26 | 4.13 |
| Chr1:223867058-223867080 | 223867073 | 5 | DNA | GGC--CGAGGTCGACTACCGGCNRG | GGCCTCGgGGgCtACacCCGGCGGG | + | 5 | 2 | 0 | 8.26 | 4.13 |
| Chr9:147054948-147054970 | 147054964 | 4 | RNA | GGCCGAGGTCGACTACCGGCNRG | G-aCGgGGTCGACgACaaGCGGG | + | 5 | 1 | 0 | 8.26 | 4.13 |
| Chr7:111060700-111060722 | 111060717 | 3 | RNA | GGCCGAGGTCGACTACCGGCNRG | GtCCG--GTgcACcACCGGaCAG | + | 5 | 2 | 0 | 8.26 | 4.13 |
| Chr6:163883708-163883730 | 163883726 | 2 | RNA | GGCCGAGGTCGACTACCGGCNRG | GaCaGAGGgCG--cACCGGaCAG | + | 5 | 2 | 0 | 8.26 | 4.13 |
| Chr8:104410705-104410727 | 104410711 | 4 | RNA | GGCCGAGGTCGACTACCGGCNRG | GcCgGAGccCGAC--CCGtCAAG | - | 5 | 2 | 0 | 8.26 | 4.13 |
| Chr5:205212791-205212813 | 205212807 | 4 | RNA | GGCCGAGGTCGACTACCGGCNRG | G-CCGAaGTacACcACCtGCTGG | + | 5 | 1 | 0 | 8.26 | 4.13 |
| Chr10:15875069-15875091 | 15875074 | 3 | RNA | GGCCGAGGTCGACTACCGGCNRG | GaCaGAGGTCGAC-gtCaGCAGG | - | 5 | 1 | 0 | 8.26 | 4.13 |
| Chr7:43839142-43839164 | 43839147 | 3 | RNA | GGCCGAGGTCGACTACCGGCNRG | GcCCGAGGcCGA--ACttGgGGG | - | 5 | 2 | 1 | 7.16 | 4.08 |
| Chr6:138881986-138882008 | 138881993 | 5 | DNA | GGCCGAGGTCGAC-TACCGGCNRG | GGCgGAGGgCGcCTTAggGGCGGG | - | 5 | 1 | 8 | 0.00 | 4.00 |
| Chr1:137743674-137743696 | 137743691 | 3 | RNA | GGCCGAGGTCGACTACCGGCNRG | GGCCGAGG--GgagtCCGGCGGG | + | 4 | 2 | 8 | 0.00 | 4.00 |
| Chr5:54424582-54424604 | 54424589 | 5 | RNA | GGCCGAGGTCGACTACCGGCNRG | cGCCGAtGa--ACcACgGGCGGG | - | 5 | 2 | 8 | 0.00 | 4.00 |
| Chr7:25947108-25947130 | 25947115 | 5 | RNA | GGCCGAGGTCGACTACCGGCNRG | GcCCGAGGTgGcCT-CgGcCTAG | - | 5 | 1 | 8 | 0.00 | 4.00 |
| Chr2:205493225-205493247 | 205493242 | 3 | RNA | GGCCGAGGTCGACTACCGGCNRG | GtCttgGGT-GACcACCGGCGAG | + | 5 | 1 | 8 | 0.00 | 4.00 |
| Chr6:135951500-135951522 | 135951517 | 3 | RNA | GGCCGAGGTCGACTACCGGCNRG | GGaCaAGG--GACTAgaGaCTAG | + | 5 | 2 | 8 | 0.00 | 4.00 |
| Chr10:52256073-52256095 | 52256080 | 5 | RNA | GGCCGAGGTCGACTACCGGCNRG | GGCCGgG--CcACTggCGGaAGG | - | 5 | 2 | 8 | 0.00 | 4.00 |
| Chr8:20984843-20984865 | 20984850 | 5 | RNA | GGCCGAGGTCGACTACCGGCNRG | GtCttgGGT-GACcACCGGCGGG | - | 5 | 1 | 8 | 0.00 | 4.00 |
| Chr10:122462295-122462317 | 122462312 | 3 | RNA | GGCCGAGGTCGACTACCGGCNRG | GG--GAcGcCGAggACCGGgGAG | + | 5 | 2 | 8 | 0.00 | 4.00 |
| Chr3:126204066-126204088 | 126204083 | 3 | RNA | GGCCGAGGTCGACTACCGGCNRG | a--CGgGGTCGACaACgaGCGGG | + | 5 | 2 | 8 | 0.00 | 4.00 |
| Chr3:76958152-76958174 | 76958159 | 5 | RNA | GGCCGAGGTCGACTACCGGCNRG | GGCCGAGGaCG--TgCgGctGGG | - | 5 | 2 | 8 | 0.00 | 4.00 |
| Chr9:65249312-65249334 | 65249329 | 3 | RNA | GGCCGAGGTCGACTACCGGCNRG | G--CGAGGTgGACggCgGGgTGG | + | 5 | 2 | 8 | 0.00 | 4.00 |
| Chr1:205586244-205586266 | 205586251 | 5 | RNA | GGCCGAGGTCGACTACCGGCNRG | GGC-GgGGTCGACaACgaGCGGG | - | 4 | 1 | 8 | 0.00 | 4.00 |
| Chr1:270302293-270302315 | 270302310 | 3 | RNA | GGCCGAGGTCGACTACCGGCNRG | G--CGAGGaCGAggACgGGaCAG | + | 5 | 2 | 8 | 0.00 | 4.00 |
| Chr4:179369305-179369327 | 179369312 | 5 | DNA | GGCCGAGGTCGAC--TACCGGCNRG | aGCCGAGGTCGAgCTTAgCatCGGG | - | 5 | 2 | 8 | 0.00 | 4.00 |
| Chr2:97653979-97654001 | 97653996 | 3 | DNA | GGCCGAGGTCGACT--ACCGGCNRG | GcCCGAcaTCGcCTACACCGtCCAG | + | 5 | 2 | 8 | 0.00 | 4.00 |
| Chr1:254800155-254800177 | 254800162 | 5 | RNA | GGCCGAGGTCGACTACCGGCNRG | G--CGtGGcCGACTgCCtGaTGG | - | 5 | 2 | 8 | 0.00 | 4.00 |
| Chr8:74268261-74268283 | 74268268 | 5 | DNA | GGCCGAGGTCGACTA--CCGGCNRG | ctCCaAGGcCGACcAATCCGGCAGG | - | 5 | 2 | 8 | 0.00 | 4.00 |
| Chr2:12385408-12385430 | 12385415 | 5 | RNA | GGCCGAGGTCGACTACCGGCNRG | G-aCGgGGTCGACaACgaGCGGG | - | 5 | 1 | 8 | 0.00 | 4.00 |
| Chr1:61400776-61400798 | 61400783 | 5 | RNA | GGCCGAGGTCGACTACCGGCNRG | GtCCG--GTgcACcACCGGaTAG | - | 5 | 2 | 8 | 0.00 | 4.00 |
| Chr7:60861832-60861854 | 60861839 | 5 | DNA | GGCCGAGGTCGACTACCG-GCNRG | cGCCaccGTCGtCTACCGCGCCGG | - | 5 | 1 | 8 | 0.00 | 4.00 |
| Chr1:94763135-94763157 | 94763152 | 3 | RNA | GGCCGAGGTCGACTACCGGCNRG | cGgtGAGcTtG--TACCGGCCAG | + | 5 | 2 | 8 | 0.00 | 4.00 |
| Chr9:128187526-128187548 | 128187533 | 5 | RNA | GGCCGAGGTCGACTACCGGCNRG | GGgCGAGG-CGcaTctCGGCAAG | - | 5 | 1 | 8 | 0.00 | 4.00 |
| Chr1:158127319-158127341 | 158127326 | 5 | DNA | GGC-CGAGGTCGACTACCGGCNRG | GcCTCGAGGaCGtCTtCCGGCTGG | - | 4 | 1 | 8 | 0.00 | 4.00 |
| Chr3:145198993-145199015 | 145199010 | 3 | X | GGCCGAGGTCGACTACCGGCNRG | GGCCGAGGTCGACTtCgGGCGAG | + | 2 | 0 | 8 | 0.00 | 4.00 |
| Chr4:120988901-120988923 | 120988918 | 3 | RNA | GGCCGAGGTCGACTACCGGCNRG | G--CGAcGaCGACgACgGGgGGG | + | 5 | 2 | 8 | 0.00 | 4.00 |
| Chr2:12705817-12705839 | 12705834 | 3 | RNA | GGCCGAGGTCGACTACCGGCNRG | GGCCaAGcgCG--gACCGtCCGG | + | 5 | 2 | 8 | 0.00 | 4.00 |
| Chr4:221567798-221567820 | 221567805 | 5 | RNA | GGCCGAGGTCGACTACCGGCNRG | GGCCGAGGgtGcCT-CgGGCTGG | - | 4 | 1 | 8 | 0.00 | 4.00 |
| Chr3:84288772-84288794 | 84288789 | 3 | RNA | GGCCGAGGTCGACTACCGGCNRG | GGCCGtGtTCatCTtCC--CGAG | + | 5 | 2 | 8 | 0.00 | 4.00 |
| Chr4:103530190-103530212 | 103530207 | 3 | DNA | GGCCGAGGTCGACTACC--GGCNRG | GGCgGAGGTCGtCagCaGAGGCCGG | + | 5 | 2 | 8 | 0.00 | 4.00 |
| Chr3:214293275-214293297 | 214293292 | 3 | RNA | GGCCGAGGTCGACTACCGGCNRG | GGCCGAGtaCG--TtgCcGCAGG | + | 5 | 2 | 8 | 0.00 | 4.00 |
| Chr5:82998323-82998345 | 82998330 | 5 | RNA | GGCCGAGGTCGACTACCGGCNRG | GaagGAGGTCGtCgA--GGCCGG | - | 5 | 2 | 8 | 0.00 | 4.00 |
| Chr8:130151606-130151628 | 130151623 | 3 | RNA | GGCCGAGGTCGACTACCGGCNRG | GGaCGAGGaCGAggA--GGgGAG | + | 5 | 2 | 8 | 0.00 | 4.00 |
| Chr6:125230154-125230176 | 125230161 | 5 | RNA | GGCCGAGGTCGACTACCGGCNRG | GctCGAGcTCGACT--CtGaTGG | - | 5 | 2 | 8 | 0.00 | 4.00 |
| Chr10:91548707-91548729 | 91548714 | 5 | RNA | GGCCGAGGTCGACTACCGGCNRG | GGaCcAGGTCG--TcCCaGtTGG | - | 5 | 2 | 8 | 0.00 | 4.00 |
| Chr5:63299889-63299911 | 63299906 | 3 | RNA | GGCCGAGGTCGACTACCGGCNRG | cGCCGcGGaCGACTcaC--CGAG | + | 5 | 2 | 8 | 0.00 | 4.00 |
| Chr1:276234159-276234181 | 276234166 | 5 | RNA | GGCCGAGGTCGACTACCGGCNRG | aGCgaAGG--GACTAatGGCGAG | - | 5 | 2 | 8 | 0.00 | 4.00 |
| Chr8:21667646-21667668 | 21667663 | 3 | RNA | GGCCGAGGTCGACTACCGGCNRG | GGgCG-GGcCaACctCCGGCCGG | + | 5 | 1 | 8 | 0.00 | 4.00 |
| Chr5:65294307-65294329 | 65294324 | 3 | RNA | GGCCGAGGTCGACTACCGGCNRG | GGCtGgGcTgGgCTA--GGCTGG | + | 5 | 2 | 8 | 0.00 | 4.00 |
| Chr2:171195536-171195558 | 171195543 | 5 | RNA | GGCCGAGGTCGACTACCGGCNRG | GGCCGgGGcgGgC--CgGGCCGG | - | 5 | 2 | 8 | 0.00 | 4.00 |

| | | | | | | | | | | | |
|---|---|---|---|---|---|---|---|---|---|---|---|
| Chr8:18781622-18781644 | 18781629 | 5 | RNA | GGCCGAGGTCGACTACCGGCNRG | GGCCaAGcgCG--gACCGtCCGG | - | 5 | 2 | 8 | 0.00 | 4.00 |
| Chr10:110702323-110702345 | 110702340 | 3 | DNA | GGC-CGAGGTCGACTACCGGCNRG | GcCTCGAGGgCtACacCCGGCGGG | + | 5 | 1 | 8 | 0.00 | 4.00 |
| Chr5:161945647-161945669 | 161945654 | 5 | DNA | GGCCGAGGT-CGACTACCGGCNRG | GGCCGAGGTGCctCTtCCaGtCAG | - | 5 | 1 | 8 | 0.00 | 4.00 |
| Chr1:194552141-194552163 | 194552158 | 3 | RNA | GGCCGAGGTCGACTACCGGCNRG | GG--GAGGagGtaTACgGGCAAG | + | 5 | 2 | 8 | 0.00 | 4.00 |
| Chr5:4113674-4113696 | 4113681 | 5 | RNA | GGCCGAGGTCGACTACCGGCNRG | GGCCGAGaTgtAtcAC--GCAGG | - | 5 | 2 | 8 | 0.00 | 4.00 |
| Chr8:41479679-41479701 | 41479686 | 5 | RNA | GGCCGAGGTCGACTACCGGCNRG | GGCCtAcaTCtACT-CCGGCGAG | - | 4 | 1 | 8 | 0.00 | 4.00 |
| Chr10:91548575-91548597 | 91548592 | 3 | RNA | GGCCGAGGTCGACTACCGGCNRG | GGC--tcGcgGACTACCGGCTGG | + | 4 | 2 | 8 | 0.00 | 4.00 |
| Chr5:11905700-11905722 | 11905707 | 5 | DNA | GGCCGAGGTCGACT-ACCGGCNRG | cGCCGcGGatGACTCACCGGaCAG | - | 5 | 1 | 8 | 0.00 | 4.00 |
| Chr6:3324436-3324458 | 3324443 | 5 | DNA | GGCCGAGGTCGA-CTACCGGCNRG | GGCCGAGGTCGAGCTcgaGGCTGG | - | 3 | 1 | 8 | 0.00 | 4.00 |
| Chr5:102936921-102936943 | 102936938 | 3 | RNA | GGCCGAGGTCGACTACCGGCNRG | GGCCGgGcTgaACT-CCGaCTAG | + | 5 | 1 | 0 | 7.71 | 3.86 |
| Chr10:59019062-59019084 | 59019067 | 3 | DNA | GGC--CGAGGTCGACTACCGGCNRG | GaCAACGgGGTCGACgACgaGCGGG | - | 5 | 2 | 0 | 7.71 | 3.86 |
| Chr5:22797751-22797773 | 22797758 | 5 | DNA | GGCCGAGGTCGACTA-CCGGCNRG | GGtgGAGGTtGtCTcTCCGGCGAG | - | 5 | 1 | 0 | 7.71 | 3.86 |
| Chr2:209209610-209209632 | 209209628 | 2 | RNA | GGCCGAGGTCGACTACCGGCNRG | aGCCGgGG--GAaTcCCtGCGGG | + | 5 | 2 | 0 | 7.71 | 3.86 |
| Chr2:42120543-42120565 | 42120561 | 2 | RNA | GGCCGAGGTCGACTACCGGCNRG | GGaCcAGGTgG--TtCgGGCGAG | + | 5 | 2 | 0 | 7.71 | 3.86 |
| Chr10:150122156-150122178 | 150122160 | 2 | RNA | GGCCGAGGTCGACTACCGGCNRG | aGCCGAGGTCGACTgg-GGgCGG | - | 4 | 1 | 0 | 7.71 | 3.86 |
| Chr10:63152603-63152625 | 63152609 | 4 | RNA | GGCCGAGGTCGACTACCGGCNRG | GGC--tcGcgGACTACCGGCTGG | - | 4 | 2 | 0 | 7.71 | 3.86 |
| Chr3:188615147-188615169 | 188615151 | 2 | RNA | GGCCGAGGTCGACTACCGGCNRG | aGCCGAcGTaG-CcACtGGCGAG | - | 5 | 1 | 0 | 7.71 | 3.86 |
| Chr5:111129257-111129279 | 111129262 | 3 | DNA | GGC--CGAGGTCGACTACCGGCNRG | GGCCTCGgGGgCtACacCCGGCGGG | - | 5 | 2 | 0 | 7.71 | 3.86 |
| Chr3:147343929-147343951 | 147343946 | 3 | RNA | GGCCGAGGTCGACTACCGGCNRG | cGaCGgGGTCGACgAC--GaGAG | + | 5 | 2 | 0 | 7.71 | 3.86 |
| Chr5:205698867-205698889 | 205698872 | 3 | RNA | GGCCGAGGTCGACTACCGGCNRG | GaCaG-GGTCGACgACgaGCGGG | - | 5 | 1 | 0 | 7.71 | 3.86 |
| Chr4:171574757-171574779 | 171574762 | 3 | RNA | GGCCGAGGTCGACTACCGGCNRG | G-aCGgGGTCGACaACgaGCGGG | - | 5 | 1 | 0 | 7.71 | 3.86 |
| Chr10:97282160-97282182 | 97282178 | 2 | RNA | GGCCGAGGTCGACTACCGGCNRG | GG--GAGGTCGggTtgCGGgAGG | + | 5 | 2 | 0 | 7.71 | 3.86 |
| Chr7:10258808-10258830 | 10258823 | 5 | RNA | GGCCGAGGTCGACTACCGGCNRG | tGCtGAGGTgGAtTACC--tGAG | + | 5 | 2 | 0 | 7.71 | 3.86 |
| Chr10:129800836-129800858 | 129800841 | 3 | RNA | GGCCGAGGTCGACTACCGGCNRG | GGC--tcGcgGACTACCGGCTGG | - | 4 | 2 | 0 | 7.71 | 3.86 |
| Chr10:147635855-147635877 | 147635870 | 5 | RNA | GGCCGAGGTCGACTACCGGCNRG | GGC--tcGcgGACTACCGcCTGG | + | 5 | 2 | 0 | 7.71 | 3.86 |
| Chr1:100983773-100983795 | 100983777 | 2 | RNA | GGCCGAGGTCGACTACCGGCNRG | tGCCGAGGTCG--TAtgGctGGG | - | 5 | 2 | 0 | 7.71 | 3.86 |
| Chr10:139168803-139168825 | 139168807 | 2 | DNA | GGCCGAGGTCGACTACCGG--CNRG | GGCCGAGGTCGAggcCgaGCCCTGG | - | 5 | 2 | 0 | 7.71 | 3.86 |
| Chr2:66966821-66966843 | 66966825 | 2 | RNA | GGCCGAGGTCGACTACCGGCNRG | GGC--AGcTCGAggACgaGCTGG | - | 5 | 2 | 0 | 7.71 | 3.86 |
| Chr3:212883751-212883773 | 212883766 | 5 | RNA | GGCCGAGGTCGACTACCGGCNRG | GGCtcAcG--GACTACCGGCTGG | + | 3 | 2 | 0 | 7.71 | 3.86 |
| Chr5:93592105-93592127 | 93592110 | 3 | RNA | GGCCGAGGTCGACTACCGGCNRG | GGCCGtcGgC--CTgCCcGCCGG | - | 5 | 2 | 0 | 7.71 | 3.86 |
| Chr5:148721181-148721203 | 148721186 | 3 | RNA | GGCCGAGGTCGACTACCGGCNRG | GGCCGtGGTaGA--AaCcGtCGG | - | 5 | 2 | 0 | 7.71 | 3.86 |
| Chr2:35713287-35713309 | 35713302 | 5 | RNA | GGCCGAGGTCGACTACCGGCNRG | GGCttgGGa--ACTACCGGaAGG | + | 5 | 2 | 0 | 7.71 | 3.86 |
| Chr5:123207231-123207253 | 123207235 | 2 | RNA | GGCCGAGGTCGACTACCGGCNRG | GGCtatGGTCaA--ACCGaCGGG | - | 5 | 2 | 0 | 7.71 | 3.86 |
| Chr2:78413225-78413247 | 78413231 | 4 | DNA | GGC--CGAGGTCGACTACCGGCNRG | GGCCTCGgGGgCtACacCCGGCGGG | - | 5 | 2 | 0 | 7.71 | 3.86 |
| Chr3:39499815-39499837 | 39499820 | 3 | RNA | GGCCGAGGTCGACTACCGGCNRG | GGCaGtGGT--ACTcCgGtCGAG | - | 5 | 2 | 0 | 7.71 | 3.86 |
| Chr1:171522793-171522815 | 171522810 | 3 | DNA | GGC-CGAGGTCGACTACCGGCNRG | GcCTCGgGGaCGtCTtCCGGCTGG | + | 5 | 1 | 0 | 7.71 | 3.86 |
| Chr10:128767734-128767756 | 128767741 | 5 | RNA | GGCCGAGGTCGACTACCGGCNRG | GGgCG-GGcCaACctCCGGCCGG | - | 5 | 1 | 0 | 7.71 | 3.86 |
| Chr2:182828126-182828148 | 182828131 | 3 | RNA | GGCCGAGGTCGACTACCGGCNRG | GtCCG--GTgcACcACCGGaCAG | - | 5 | 2 | 0 | 7.71 | 3.86 |
| Chr2:126913098-126913120 | 126913103 | 3 | RNA | GGCCGAGGTCGACTACCGGCNRG | cGCCGAaG-CtACcACCGGaTGG | - | 5 | 1 | 0 | 7.71 | 3.86 |
| Chr3:209871649-209871671 | 209871655 | 4 | DNA | GGCCGAGGTCGACTA--CCGGCNRG | GGgCGgGGTCGgCgAAGCaGGCCAG | - | 5 | 2 | 0 | 7.71 | 3.86 |
| Chr3:57466224-57466246 | 57466241 | 3 | RNA | GGCCGAGGTCGACTACCGGCNRG | GaCCcAaGTCaACTACt--CAGG | + | 5 | 2 | 0 | 7.71 | 3.86 |
| Chr1:102297391-102297413 | 102297397 | 4 | RNA | GGCCGAGGTCGACTACCGGCNRG | GaCCGAGGaCttCTACC--aTGG | - | 5 | 2 | 0 | 7.71 | 3.86 |
| Chr1:129682246-129682268 | 129682251 | 3 | RNA | GGCCGAGGTCGACTACCGGCNRG | GGCCGgGGgatACT-CtGGCGAG | - | 5 | 1 | 0 | 7.71 | 3.86 |
| Chr7:52124860-52124882 | 52124878 | 2 | RNA | GGCCGAGGTCGACTACCGGCNRG | GGCgGcGGTgG--TgCtGGCGGG | + | 5 | 2 | 0 | 7.71 | 3.86 |
| Chr10:53136815-53136837 | 53136822 | 5 | RNA | GGCCGAGGTCGACTACCGGCNRG | caCCaAGtTCGcCT-CCGGCTAG | - | 5 | 1 | 0 | 7.71 | 3.86 |
| Chr3:83227638-83227660 | 83227644 | 4 | RNA | GGCCGAGGTCGACTACCGGCNRG | GGCCGAtcTCG--cAtCaGCAAG | - | 5 | 2 | 0 | 7.71 | 3.86 |
| Chr2:145347124-145347146 | 145347139 | 5 | RNA | GGCCGAGGTCGACTACCGGCNRG | GtCgGActTCGACaAC--GCGGG | + | 5 | 2 | 0 | 7.71 | 3.86 |
| Chr7:167250194-167250216 | 167250199 | 3 | RNA | GGCCGAGGTCGACTACCGGCNRG | GGCgGAGG--GACagCtGtCGGG | - | 5 | 2 | 0 | 7.71 | 3.86 |
| Chr5:201556778-201556800 | 201556785 | 5 | RNA | GGCCGAGGTCGACTACCGGCNRG | GGgCaAGGgCGACgtCC--CCGG | - | 5 | 2 | 0 | 7.71 | 3.86 |
| Chr6:159401411-159401433 | 159401416 | 3 | RNA | GGCCGAGGTCGACTACCGGCNRG | G-aCGgGGTCGACgACgaGCGGG | - | 5 | 1 | 0 | 7.71 | 3.86 |
| Chr10:41545147-41545169 | 41545152 | 3 | DNA | GGCCGAGGTCGACTACC--GGCNRG | GcCCGAGagCaACcACCGTGGCCAG | - | 5 | 2 | 0 | 7.71 | 3.86 |
| Chr8:21977857-21977879 | 21977875 | 2 | DNA | GGCCGAGG-TCGACTACCGGCNRG | GaCCcgGGTTCGAtTcCCGGCTGG | + | 5 | 1 | 0 | 7.71 | 3.86 |
| Chr7:41944476-41944498 | 41944481 | 3 | RNA | GGCCGAGGTCGACTACCGGCNRG | GtaCG--GTtGAaTACtGGCCGG | - | 5 | 2 | 0 | 7.71 | 3.86 |
| Chr4:79739608-79739630 | 79739623 | 5 | RNA | GGCCGAGGTCGACTACCGGCNRG | GGgCGgGGTtGgCT--CGGgGAG | + | 5 | 2 | 0 | 7.71 | 3.86 |
| Chr7:20017349-20017371 | 20017365 | 4 | RNA | GGCCGAGGTCGACTACCGGCNRG | GtCaGActTCGACaAC--GCAGG | + | 5 | 2 | 0 | 7.71 | 3.86 |
| Chr5:132284412-132284434 | 132284430 | 2 | RNA | GGCCGAGGTCGACTACCGGCNRG | tGCaGAGcTCGgCTA--GGCTGG | + | 4 | 2 | 0 | 7.71 | 3.86 |
| Chr6:83985168-83985190 | 83985172 | 2 | RNA | GGCCGAGGTCGACTACCGGCNRG | GGaCGAGG--GtCcgCCGGgAAG | - | 5 | 2 | 0 | 7.71 | 3.86 |
| Chr6:86858920-86858942 | 86858937 | 3 | DNA | GGC-CGAGGTCGACTACCGGCNRG | GGCTCGAatTCtACcACCGGCTGG | + | 4 | 1 | 0 | 7.71 | 3.86 |
| Chr10:28362556-28362578 | 28362562 | 4 | RNA | GGCCGAGGTCGACTACCGGCNRG | aGCCtcGGgCGACTACt--CTAG | - | 5 | 2 | 0 | 7.71 | 3.86 |
| Chr2:39860358-39860380 | 39860363 | 3 | RNA | GGCCGAGGTCGACTACCGGCNRG | GGtCcAGGgC--CTtCCtGCGGG | - | 5 | 2 | 0 | 7.71 | 3.86 |
| Chr4:244201160-244201182 | 244201165 | 3 | RNA | GGCCGAGGTCGACTACCGGCNRG | G-aCGgGGTCGACcACgaGCGGG | - | 5 | 1 | 0 | 7.71 | 3.86 |
| Chr1:259160861-259160883 | 259160868 | 5 | RNA | GGCCGAGGTCGACTACCGGCNRG | GGC--tcGcaGACTACtGGCTGG | - | 5 | 2 | 0 | 7.71 | 3.86 |
| Chr9:156236554-156236576 | 156236570 | 4 | RNA | GGCCGAGGTCGACTACCGGCNRG | aGCCGAGGgtG-CcACaGGCTGG | + | 5 | 1 | 0 | 7.71 | 3.86 |
| Chr10:132199051-132199073 | 132199058 | 5 | RNA | GGCCGAGGTCGACTACCGGCNRG | G--CGAGGTCGgCggCCGcgAAG | - | 5 | 2 | 0 | 7.71 | 3.86 |
| Chr6:146032127-146032149 | 146032132 | 3 | RNA | GGCCGAGGTCGACTACCGGCNRG | G-aCGgGGTCGACgACgaGCGGG | - | 5 | 1 | 0 | 7.71 | 3.86 |
| Chr3:117491968-117491990 | 117491986 | 2 | DNA | GGC--CGAGGTCGACTACCGGCNRG | GGCCTCGAGGgCtACacCCGGCGGG | + | 4 | 2 | 0 | 7.71 | 3.86 |
| Chr9:119038444-119038466 | 119038448 | 2 | DNA | GGCCGAGGTCGACTACC--GGCNRG | cGCCGccGTCtACTACCGCGcCGGG | - | 5 | 2 | 0 | 7.71 | 3.86 |
| Chr4:21100458-21100480 | 21100462 | 2 | RNA | GGCCGAGGTCGACTACCGGCNRG | GGCCcAGGTgtAC-AatGGCGGG | - | 5 | 1 | 0 | 7.71 | 3.86 |
| Chr6:165163533-165163555 | 165163551 | 2 | DNA | G-GCCGAGGTCGACTACCGGCNRG | GCGCCtcGGgCGAgTACtGGCAGG | + | 5 | 1 | 0 | 7.71 | 3.86 |
| Chr9:133600039-133600061 | 133600054 | 5 | RNA | GGCCGAGGTCGACTACCGGCNRG | GGC--ttGcgGACTACCGGCTGG | + | 4 | 2 | 6 | 1.65 | 3.83 |
| Chr3:216843243-216843265 | 216843261 | 2 | RNA | GGCCGAGGTCGACTACCGGCNRG | GGC--gGGTaGACgAgCaGCAAG | + | 5 | 2 | 1 | 6.61 | 3.81 |
| Chr4:142295394-142295416 | 142295400 | 4 | RNA | GGCCGAGGTCGACTACCGGCNRG | GGC--AGGcCGAgcACCGGCCGG | - | 3 | 2 | 7 | 0.55 | 3.78 |
| Chr6:71670360-71670382 | 71670365 | 3 | RNA | GGCCGAGGTCGACTACCGGCNRG | G-CCGgaGTCGACTgCCGGtGAG | - | 4 | 1 | 7 | 0.55 | 3.78 |
| Chr10:123030488-123030510 | 123030494 | 4 | RNA | GGCCGAGGTCGACTACCGGCNRG | tG--GAGGTtGcCaACCGGaTAG | - | 5 | 2 | 2 | 5.51 | 3.76 |
| Chr3:129365996-129366018 | 129366011 | 5 | RNA | GGCCGAGGTCGACTACCGGCNRG | GGgC-AGGcCGAgcACCGGCCGG | + | 4 | 1 | 0 | 7.16 | 3.58 |
| Chr7:22380372-22380394 | 22380387 | 5 | RNA | GGCCGAGGTCGACTACCGGCNRG | a--CGgGGTCGACgACgaGCGGG | + | 5 | 2 | 0 | 7.16 | 3.58 |
| Chr9:91636078-91636100 | 91636094 | 4 | RNA | GGCCGAGGTCGACTACCGGCNRG | G-aCGgGGTCGACaACgaGCGGG | + | 5 | 1 | 0 | 7.16 | 3.58 |
| Chr2:8288552-8288574 | 8288570 | 2 | RNA | GGCCGAGGTCGACTACCGGCNRG | GaCCGAGG-CGACgACaaGaAGG | + | 5 | 1 | 0 | 7.16 | 3.58 |
| Chr6:349520-349542 | 349527 | 5 | RNA | GGCCGAGGTCGACTACCGGCNRG | GcCCGAGGgCaACcACC--gTGG | - | 5 | 2 | 0 | 7.16 | 3.58 |
| Chr7:70339169-70339191 | 70339174 | 3 | RNA | GGCCGAGGTCGACTACCGGCNRG | G-aCGgGGTCGACaACgaGCGGG | - | 5 | 1 | 0 | 7.16 | 3.58 |
| Chr6:144446673-144446695 | 144446690 | 3 | RNA | GGCCGAGGTCGACTACCGGCNRG | aGt--tGGTgGAaTACCGGCAAG | + | 5 | 2 | 0 | 7.16 | 3.58 |
| Chr7:133222422-133222444 | 133222428 | 4 | RNA | GGCCGAGGTCGACTACCGGCNRG | GGgCG-GGcCaACcACCGGCCGG | - | 4 | 1 | 0 | 7.16 | 3.58 |
| Chr2:216374341-216374363 | 216374347 | 4 | RNA | GGCCGAGGTCGACTACCGGCNRG | GGCgGcGGTCGAC-AtCtcCCGG | - | 5 | 1 | 0 | 7.16 | 3.58 |
| Chr3:163017842-163017864 | 163017858 | 4 | RNA | GGCCGAGGTCGACTACCGGCNRG | G-aCGgGGTCGACaACgaGCAGG | + | 5 | 1 | 0 | 7.16 | 3.58 |
| Chr4:28897450-28897472 | 28897455 | 3 | RNA | GGCCGAGGTCGACTACCGGCNRG | tGCCGAGGTCG--TAtgGctGGG | - | 5 | 2 | 0 | 7.16 | 3.58 |
| Chr7:171206166-171206188 | 171206184 | 2 | RNA | GGCCGAGGTCGACTACCGGCNRG | GGCCGAGcgCGA--AggGGgAAG | + | 5 | 2 | 0 | 7.16 | 3.58 |
| Chr4:70715583-70715605 | 70715601 | 2 | RNA | GGCCGAGGTCGACTACCGGCNRG | GGCgGAGGTCGttTggC--CGAG | + | 5 | 2 | 0 | 7.16 | 3.58 |
| Chr10:9074274-9074296 | 9074290 | 4 | RNA | GGCCGAGGTCGACTACCGGCNRG | GaCaG-GGTCGACgACaaGCGGG | + | 5 | 1 | 0 | 7.16 | 3.58 |
| Chr1:87264724-87264746 | 87264741 | 3 | RNA | GGCCGAGGTCGACTACCGGCNRG | GaCCGAGGagGgCT-CtGGCGAG | + | 5 | 1 | 0 | 7.16 | 3.58 |
| Chr7:149461770-149461792 | 149461775 | 3 | RNA | GGCCGAGGTCGACTACCGGCNRG | cGCCGA--TCGAtTtCCGGCAGG | - | 3 | 2 | 0 | 7.16 | 3.58 |
| Chr10:67516012-67516034 | 67516018 | 4 | RNA | GGCCGAGGTCGACTACCGGCNRG | GtCCG--GTgcACcACCGGaCAG | - | 5 | 2 | 0 | 7.16 | 3.58 |
| Chr5:172175136-172175158 | 172175141 | 3 | RNA | GGCCGAGGTCGACTACCGGCNRG | GGC--tcGcgGACTACCGaCTGG | - | 5 | 2 | 0 | 7.16 | 3.58 |
| Chr7:30880933-30880955 | 30880938 | 3 | RNA | GGCCGAGGTCGACTACCGGCNRG | GaCaG-GGTCGACgACaaGCGGG | - | 5 | 1 | 0 | 7.16 | 3.58 |
| Chr6:158971260-158971282 | 158971276 | 4 | RNA | GGCCGAGGTCGACTACCGGCNRG | GGa--AaGTCGACTAtCtcCAAG | + | 5 | 2 | 0 | 7.16 | 3.58 |
| Chr4:182081075-182081097 | 182081080 | 3 | RNA | GGCCGAGGTCGACTACCGGCNRG | GaCgGgGGgCGA--ACCGGCCGG | - | 4 | 2 | 0 | 7.16 | 3.58 |
| Chr7:34243873-34243895 | 34243879 | 4 | RNA | GGCCGAGGTCGACTACCGGCNRG | GGCatAGGaC-ACTACaGGCAGG | - | 4 | 1 | 0 | 7.16 | 3.58 |
| Chr7:50901975-50901997 | 50901993 | 2 | RNA | GGCCGAGGTCGACTACCGGCNRG | GGC-GAcGTtGACTACCacgAGG | + | 5 | 1 | 0 | 7.16 | 3.58 |
| Chr5:153506362-153506384 | 153506378 | 4 | RNA | GGCCGAGGTCGACTACCGGCNRG | GGCaGcaGT-GACTACtcGCTGG | + | 5 | 1 | 0 | 7.16 | 3.58 |
| Chr4:123030529-123030551 | 123030545 | 4 | RNA | GGCCGAGGTCGACTACCGGCNRG | tG--GAcGTCGAggACaGGCGGG | + | 5 | 2 | 0 | 7.16 | 3.58 |
| Chr7:67571569-67571591 | 67571585 | 4 | DNA | GGCCGAGGT--CGACTACCGGCNRG | GGCtGAGGgGCttACTAaCGGCGAG | + | 5 | 2 | 0 | 7.16 | 3.58 |
| Chr10:33397048-33397070 | 33397064 | 4 | RNA | GGCCGAGGTCGACTACCGGCNRG | G-aCGAGaTCGACaACgaGCGGG | + | 5 | 1 | 0 | 7.16 | 3.58 |
| Chr4:6330673-6330695 | 6330689 | 4 | RNA | GGCCGAGGTCGACTACCGGCNRG | GGCgGAGGg-GACgACtGGtTAG | + | 5 | 1 | 0 | 7.16 | 3.58 |
| Chr4:226502511-226502533 | 226502518 | 5 | RNA | GGCCGAGGTCGACTACCGGCNRG | GcCCGAGGgCaACcACC--aTGG | - | 5 | 2 | 0 | 7.16 | 3.58 |
| Chr4:59004096-59004118 | 59004111 | 5 | RNA | GGCCGAGGTCGACTACCGGCNRG | GGCCGAGcgCGA--AggGGgGAG | + | 5 | 2 | 0 | 7.16 | 3.58 |
| Chr5:17433511-17433533 | 17433527 | 4 | RNA | GGCCGAGGTCGACTACCGGCNRG | GcCgGgGGTCGACTt--GGaGGG | + | 5 | 2 | 0 | 7.16 | 3.58 |
| Chr6:73639609-73639631 | 73639614 | 3 | RNA | GGCCGAGGTCGACTACCGGCNRG | GcCgGAGccCGAC--CCGtCAAG | - | 5 | 2 | 0 | 7.16 | 3.58 |
| Chr5:115200351-115200373 | 115200367 | 4 | DNA | GGCCGAGGTCGACTACCG--GCNRG | GGtCGAGGgCGAaTgaCGATGCGAG | + | 5 | 2 | 0 | 7.16 | 3.58 |
| Chr1:78790585-78790607 | 78790603 | 2 | RNA | GGCCGAGGTCGACTACCGGCNRG | tGCaGAGcTCGACTA--GGtTGG | + | 4 | 2 | 0 | 7.16 | 3.58 |
| Chr3:402259-402281 | 402265 | 4 | RNA | GGCCGAGGTCGACTACCGGCNRG | tGgCGAGGTCGAggACC--tTGG | - | 5 | 2 | 0 | 7.16 | 3.58 |
| Chr1:93474325-93474347 | 93474329 | 2 | RNA | GGCCGAGGTCGACTACCGGCNRG | GGCCGAGGTCGgCT-CaGGgAGG | - | 3 | 1 | 0 | 7.16 | 3.58 |
| Chr2:197224569-197224591 | 197224574 | 3 | RNA | GGCCGAGGTCGACTACCGGCNRG | GGtCGAc--CGACggCCGGgGGG | - | 5 | 2 | 0 | 7.16 | 3.58 |
| Chr10:16220929-16220951 | 16220947 | 2 | RNA | GGCCGAGGTCGACTACCGGCNRG | GGgCG-GGcCaACctCCGGCTGG | + | 5 | 1 | 0 | 7.16 | 3.58 |
| Chr7:22342053-22342075 | 22342060 | 5 | RNA | GGCCGAGGTCGACTACCGGCNRG | cGaCGAGcTCGACT-CtGGtTGG | - | 5 | 1 | 0 | 7.16 | 3.58 |
| Chr6:9863390-9863412 | 9863406 | 4 | RNA | GGCCGAGGTCGACTACCGGCNRG | G-aCGgGGTCGACaACgaGCGGG | + | 5 | 1 | 0 | 7.16 | 3.58 |
| Chr4:243994844-243994866 | 243994859 | 5 | RNA | GGCCGAGGTCGACTACCGGCNRG | GGgCG-GGcCaACatCCGGCCGG | + | 5 | 1 | 0 | 7.16 | 3.58 |
| Chr3:210835976-210835998 | 210835982 | 4 | RNA | GGCCGAGGTCGACTACCGGCNRG | GGCCGAaGcCG--gACCaGaTGG | - | 5 | 2 | 0 | 7.16 | 3.58 |
| Chr5:119332567-119332589 | 119332573 | 4 | RNA | GGCCGAGGTCGACTACCGGCNRG | GGCCGAaGcgGA--ACCaGaTGG | - | 5 | 2 | 0 | 7.16 | 3.58 |
| Chr1:111870679-111870701 | 111870695 | 4 | DNA | GGCCGAGGTCGACTACC--GGCNRG | GGCCGActTgGgCTAaCTGGGCTAG | + | 5 | 2 | 0 | 7.16 | 3.58 |
| Chr6:91323980-91324002 | 91323985 | 3 | RNA | GGCCGAGGTCGACTACCGGCNRG | GGgCGAGagCGAC--CtGGCGGG | - | 4 | 2 | 0 | 7.16 | 3.58 |
| Chr7:77885290-77885312 | 77885306 | 4 | RNA | GGCCGAGGTCGACTACCGGCNRG | G-aCGgGGTCGACgACgaGCGGG | + | 5 | 1 | 0 | 7.16 | 3.58 |
| Chr6:79443078-79443100 | 79443083 | 3 | RNA | GGCCGAGGTCGACTACCGGCNRG | G-aCGgGGTCGACgACgaGCGGG | - | 5 | 1 | 0 | 7.16 | 3.58 |
| Chr2:52246652-52246674 | 52246667 | 5 | RNA | GGCCGAGGTCGACTACCGGCNRG | GGtCacGGTCGAgTgC--GCTGG | + | 5 | 2 | 0 | 7.16 | 3.58 |
| Chr10:58332207-58332229 | 58332224 | 3 | DNA | G--GCCGAGGTCGACTACCGGCNRG | GATGaCGgGGTCGACgACgaGCGGG | + | 5 | 2 | 0 | 7.16 | 3.58 |
| Chr5:123649676-123649698 | 123649681 | 3 | RNA | GGCCGAGGTCGACTACCGGCNRG | GGC--ttGcgGACTACCGGCTGG | - | 4 | 2 | 0 | 7.16 | 3.58 |
| Chr4:44343443-44343465 | 44343448 | 3 | RNA | GGCCGAGGTCGACTACCGGCNRG | GG--GAGGgCGggTgCgGGCGAG | - | 5 | 2 | 0 | 7.16 | 3.58 |
| Chr8:27381695-27381717 | 27381710 | 5 | RNA | GGCCGAGGTCGACTACCGGCNRG | GGCCGgGccgGACTA--GGtTGG | + | 5 | 2 | 0 | 7.16 | 3.58 |

| | | | | | | | | | | | |
|---|---|---|---|---|---|---|---|---|---|---|---|
| Chr7:22832794-22832816 | 22832799 | 3 | RNA | GGCCGAGGTCGACTACCGGCNRG | GGCttAcG--GACTACCGGtTGG | - | 4 | 2 | 0 | 7.16 | 3.58 |
| Chr10:148732123-148732145 | 148732138 | 5 | RNA | GGCCGAGGTCGACTACCGGCNRG | GGCCGAGGT--AaaAggGGaTGG | + | 5 | 2 | 0 | 7.16 | 3.58 |
| Chr7:5877505-5877527 | 5877510 | 3 | DNA | GGCCGAGGTCG-ACTACCGGCNRG | GGCgGAGtcCGCACgAaCGGCGAG | - | 5 | 1 | 0 | 7.16 | 3.58 |
| Chr2:177707135-177707157 | 177707141 | 4 | RNA | GGCCGAGGTCGACTACCGGCNRG | caCCaAGtTCGcCT-CCGGCCAG | - | 5 | 1 | 0 | 7.16 | 3.58 |
| Chr2:55203621-55203643 | 55203628 | 5 | RNA | GGCCGAGGTCGACTACCGGCNRG | GGatGAGGagGACT--CGGaCGG | - | 5 | 2 | 0 | 7.16 | 3.58 |
| Chr7:29338454-29338476 | 29338470 | 4 | RNA | GGCCGAGGTCGACTACCGGCNRG | G-aCGgGGTCGACcACgaGCGGG | + | 5 | 1 | 0 | 7.16 | 3.58 |
| Chr5:203929589-203929611 | 203929607 | 2 | RNA | GGCCGAGGTCGACTACCGGCNRG | cGCCGtcGcCG--TACCGGCCGG | + | 4 | 2 | 0 | 7.16 | 3.58 |
| Chr4:220932677-220932699 | 220932695 | 2 | DNA | GGCCGAGGTCG-ACTACCGGCNRG | GGgCtAGGaaGCgCTACCGGCTGG | + | 5 | 1 | 0 | 7.16 | 3.58 |
| Chr6:96275518-96275540 | 96275534 | 4 | RNA | GGCCGAGGTCGACTACCGGCNRG | GGC-tAGGcCGAggACCtGCCGG | + | 5 | 1 | 0 | 7.16 | 3.58 |
| Chr1:298705082-298705104 | 298705087 | 3 | RNA | GGCCGAGGTCGACTACCGGCNRG | GaCCGtGGaCGgCaAC--GCAAG | - | 5 | 2 | 0 | 7.16 | 3.58 |
| Chr4:11090905-11090927 | 11090922 | 3 | RNA | GGCCGAGGTCGACTACCGGCNRG | GGCtGgGaTCGcCTt--GGCAGG | + | 5 | 2 | 0 | 7.16 | 3.58 |
| Chr3:106976798-106976820 | 106976816 | 2 | DNA | GGCCGAGGTCGAC-TACCGGCNRG | cGCgGgcGTCGACGTACaGGCGGG | + | 5 | 1 | 0 | 7.16 | 3.58 |
| Chr10:128414405-128414427 | 128414423 | 2 | DNA | GGCCGAGGTCGACT--ACCGGCNRG | GGgtGAGGTtaAtTGGACCGGCGGG | + | 5 | 2 | 0 | 7.16 | 3.58 |
| Chr1:129829565-129829587 | 129829582 | 3 | RNA | GGCCGAGGTCGACTACCGGCNRG | GGgCGAGcTCGgCTg--GGCGAG | + | 4 | 2 | 0 | 7.16 | 3.58 |
| Chr3:59076867-59076889 | 59076872 | 3 | RNA | GGCCGAGGTCGACTACCGGCNRG | GG--GtGGcgGACTtCgGGCGGG | - | 5 | 2 | 0 | 7.16 | 3.58 |
| Chr4:151252361-151252383 | 151252377 | 4 | RNA | GGCCGAGGTCGACTACCGGCNRG | G-aCGgGGTCGACgACaaGCGGG | + | 5 | 1 | 0 | 7.16 | 3.58 |
| Chr8:79028944-79028966 | 79028949 | 3 | RNA | GGCCGAGGTCGACTACCGGCNRG | G-CCGAaGTCGAagACCttCGGG | - | 5 | 1 | 0 | 7.16 | 3.58 |
| Chr4:22645704-22645726 | 22645708 | 2 | RNA | GGCCGAGGTCGACTACCGGCNRG | GGaaGAGGTCGAC-AaCGGgCGG | - | 4 | 1 | 6 | 1.10 | 3.55 |
| Chr6:153135198-153135220 | 153135215 | 3 | RNA | GGCCGAGGTCGACTACCGGCNRG | GttCGAaGgCGA--ACtGGCGGG | + | 5 | 2 | 7 | 0.00 | 3.50 |
| Chr2:203927648-203927670 | 203927665 | 3 | DNA | G--GCCGAGGTCGACTACCGGCNRG | GATGaCGgGGTCGACaACgaGCGGG | + | 5 | 2 | 7 | 0.00 | 3.50 |
| Chr5:43118194-43118216 | 43118211 | 3 | RNA | GGCCGAGGTCGACTACCGGCNRG | GtCCG--GTgcACcACCGGgCAG | + | 5 | 2 | 7 | 0.00 | 3.50 |
| Chr5:34213261-34213283 | 34213278 | 3 | RNA | GGCCGAGGTCGACTACCGGCNRG | G-aCGgGGTCGACgACgaGCGGG | + | 5 | 1 | 7 | 0.00 | 3.50 |
| Chr1:118890949-118890971 | 118890956 | 5 | RNA | GGCCGAGGTCGACTACCGGCNRG | GGCCGgGccgGACTg--GGCTGG | - | 5 | 2 | 7 | 0.00 | 3.50 |
| Chr10:50507707-50507729 | 50507714 | 5 | RNA | GGCCGAGGTCGACTACCGGCNRG | GtCCG--GTgcACcACCGGaCAG | - | 5 | 2 | 7 | 0.00 | 3.50 |
| Chr5:184500071-184500093 | 184500078 | 5 | RNA | GGCCGAGGTCGACTACCGGCNRG | GGCtGgGcTgGgCTA--GGCTGG | - | 5 | 2 | 7 | 0.00 | 3.50 |
| Chr8:101357451-101357473 | 101357458 | 5 | RNA | GGCCGAGGTCGACTACCGGCNRG | G-aCGgGGTCGACgACgaGCGGG | - | 5 | 1 | 7 | 0.00 | 3.50 |
| Chr3:122575340-122575362 | 122575357 | 3 | RNA | GGCCGAGGTCGACTACCGGCNRG | GGCCGgGcTCtA--AaCGcCCAG | + | 5 | 2 | 7 | 0.00 | 3.50 |
| Chr5:139807793-139807815 | 139807810 | 3 | DNA | GGCCGAGGTCGACT-ACCGGCNRG | GGgCGAGGTgGACTGACCGGCCGG | + | 2 | 1 | 7 | 0.00 | 3.50 |
| Chr1:132397497-132397519 | 132397514 | 3 | RNA | GGCCGAGGTCGACTACCGGCNRG | GGCatAGGaC-ACTACaGGCAGG | + | 4 | 1 | 7 | 0.00 | 3.50 |
| Chr10:75544386-75544408 | 75544403 | 3 | X | GGCCGAGGTCGACTACCGGCNRG | GGCCGAGGTCGAgcttgGGCGAG | + | 5 | 0 | 7 | 0.00 | 3.50 |
| Chr1:82565696-82565718 | 82565713 | 3 | RNA | GGCCGAGGTCGACTACCGGCNRG | ctCCGtcGTCaA--ACCGGCAAG | + | 5 | 2 | 7 | 0.00 | 3.50 |
| Chr4:94360303-94360325 | 94360310 | 5 | DNA | GGCCGAGGT--CGACTACCGGCNRG | GtaCGAGGgAACttCTACCGGCAGG | - | 5 | 2 | 7 | 0.00 | 3.50 |
| Chr6:6243148-6243170 | 6243155 | 5 | RNA | GGCCGAGGTCGACTACCGGCNRG | GGCCGAGcTgGgCTg--GGtCGG | - | 5 | 2 | 7 | 0.00 | 3.50 |
| Chr3:147242959-147242981 | 147242976 | 3 | RNA | GGCCGAGGTCGACTACCGGCNRG | GGC--ttGTgGAtTACCGGCTGG | + | 4 | 2 | 7 | 0.00 | 3.50 |
| Chr4:79771225-79771247 | 79771242 | 3 | RNA | GGCCGAGGTCGACTACCGGCNRG | cGCCc--GTCGtggACCGGCGAG | + | 5 | 2 | 7 | 0.00 | 3.50 |
| Chr2:210440077-210440099 | 210440084 | 5 | RNA | GGCCGAGGTCGACTACCGGCNRG | aGCaGAG--CGAacgCCGGCGGG | - | 5 | 2 | 7 | 0.00 | 3.50 |
| Chr8:2789127-2789149 | 2789144 | 3 | RNA | GGCCGAGGTCGACTACCGGCNRG | GGC--tcGcgGACTACCGtCTGG | + | 5 | 2 | 7 | 0.00 | 3.50 |
| Chr9:114322401-114322423 | 114322418 | 3 | RNA | GGCCGAGGTCGACTACCGGCNRG | GGC--AtGTCcAaTACCttCAAG | + | 5 | 2 | 7 | 0.00 | 3.50 |
| Chr1:96423243-96423265 | 96423250 | 5 | RNA | GGCCGAGGTCGACTACCGGCNRG | GGgCG-GGcCaACctCCGGCCGG | - | 5 | 1 | 7 | 0.00 | 3.50 |
| Chr6:126437749-126437771 | 126437766 | 3 | RNA | GGCCGAGGTCGACTACCGGCNRG | GGCCGAGtaCG--TtgCcGCAGG | + | 5 | 2 | 7 | 0.00 | 3.50 |
| Chr4:82973383-82973405 | 82973390 | 5 | RNA | GGCCGAGGTCGACTACCGGCNRG | tG--GAGGaaGACgACCtGCAAG | - | 5 | 2 | 7 | 0.00 | 3.50 |
| Chr1:83586315-83586337 | 83586322 | 5 | RNA | GGCCGAGGTCGACTACCGGCNRG | GaCaG-GGTCGACaACgaGCGGG | - | 5 | 1 | 7 | 0.00 | 3.50 |
| Chr9:81332157-81332179 | 81332174 | 3 | RNA | GGCCGAGGTCGACTACCGGCNRG | G-aCGgGGTCGACaACgaGCGGG | + | 5 | 1 | 7 | 0.00 | 3.50 |
| Chr2:123904469-123904491 | 123904486 | 3 | RNA | GGCCGAGGTCGACTACCGGCNRG | G-CCGAGGaCGcCgAgCaGCTGG | + | 5 | 1 | 7 | 0.00 | 3.50 |
| Chr7:72631503-72631525 | 72631510 | 5 | RNA | GGCCGAGGTCGACTACCGGCNRG | GGC-GAGtTCGACgACgaGaTAG | - | 5 | 1 | 7 | 0.00 | 3.50 |
| Chr7:89813178-89813200 | 89813195 | 3 | RNA | GGCCGAGGTCGACTACCGGCNRG | acgCGAGGgC--CcACCGGCCGG | + | 5 | 2 | 7 | 0.00 | 3.50 |
| Chr9:18535881-18535903 | 18535888 | 5 | DNA | GGCCGAGGT--CGACTACCGGCNRG | GGCtcAGGTGAaGAgcACCGGCCGG | - | 5 | 2 | 7 | 0.00 | 3.50 |
| Chr5:98526228-98526250 | 98526245 | 3 | RNA | GGCCGAGGTCGACTACCGGCNRG | GGgCGAG--CGACTAagatCAGG | + | 5 | 2 | 7 | 0.00 | 3.50 |
| Chr4:44065607-44065629 | 44065614 | 5 | DNA | GGC-CGAGGTCGACTACCGGCNRG | GcCTCGAGGaCGtCTtCCGGCTGG | - | 4 | 1 | 7 | 0.00 | 3.50 |
| Chr2:14961540-14961562 | 14961557 | 3 | RNA | GGCCGAGGTCGACTACCGGCNRG | GtCgGActTCGACaAC--GCGGG | + | 5 | 2 | 7 | 0.00 | 3.50 |
| Chr7:129103879-129103901 | 129103886 | 5 | RNA | GGCCGAGGTCGACTACCGGCNRG | GGC--tcGcgGACTACCGGCTGG | - | 4 | 2 | 7 | 0.00 | 3.50 |
| Chr6:154784775-154784797 | 154784782 | 5 | RNA | GGCCGAGGTCGACTACCGGCNRG | G-CaGtGGagcACTACCGGCCGG | - | 5 | 1 | 7 | 0.00 | 3.50 |
| Chr3:171376425-171376447 | 171376442 | 3 | RNA | GGCCGAGGTCGACTACCGGCNRG | GGCgGAGGTCG--TtCaGcaGAG | + | 5 | 2 | 7 | 0.00 | 3.50 |
| Chr7:73073809-73073831 | 73073816 | 5 | RNA | GGCCGAGGTCGACTACCGGCNRG | cGCCG--GTCctCatCCGGCCGG | - | 5 | 2 | 7 | 0.00 | 3.50 |
| Chr4:67786921-67786943 | 67786938 | 3 | DNA | GGC-CGAGGTCGACTACCGGCNRG | GcCACGgGGaCtACTtCCGGCTGG | + | 5 | 1 | 7 | 0.00 | 3.50 |
| Chr4:170665740-170665762 | 170665747 | 5 | RNA | GGCCGAGGTCGACTACCGGCNRG | GGCCGAGG-CGAggcCgGGtAGG | - | 5 | 1 | 7 | 0.00 | 3.50 |
| Chr2:78577394-78577416 | 78577401 | 5 | DNA | GGCCGAGGTCGACTACC--GGCNRG | GcCCaAGcaCGACcACCGTGGCCAG | - | 5 | 2 | 7 | 0.00 | 3.50 |
| Chr8:46329914-46329936 | 46329931 | 3 | RNA | GGCCGAGGTCGACTACCGGCNRG | GGCCGtctTCGAgTA--GaCCGG | + | 5 | 2 | 7 | 0.00 | 3.50 |
| Chr8:162864120-162864142 | 162864137 | 3 | DNA | GGC-CGAGGTCGACTACCGGCNRG | GtCTCGAGGaCGtCTtCCGGCTGG | + | 4 | 1 | 7 | 0.00 | 3.50 |
| Chr5:136819775-136819797 | 136819782 | 5 | RNA | GGCCGAGGTCGACTACCGGCNRG | GGCCG--GcaaACTACCaGtAAG | - | 5 | 2 | 7 | 0.00 | 3.50 |
| Chr5:17629262-17629284 | 17629269 | 5 | RNA | GGCCGAGGTCGACTACCGGCNRG | GaCCGAGGT-GACgACaaGaAGG | - | 5 | 1 | 7 | 0.00 | 3.50 |
| Chr1:208079284-208079306 | 208079291 | 5 | RNA | GGCCGAGGTCGACTACCGGCNRG | GttCaAGGTCt--TACCGGaGAG | - | 5 | 2 | 7 | 0.00 | 3.50 |
| Chr10:87020568-87020590 | 87020585 | 3 | RNA | GGCCGAGGTCGACTACCGGCNRG | G-aCGgGGTCGACaACgaGCGGG | + | 5 | 1 | 7 | 0.00 | 3.50 |
| Chr2:132714464-132714486 | 132714481 | 3 | RNA | GGCCGAGGTCGACTACCGGCNRG | a--CGgGGTCGACgACgaGCGGG | + | 5 | 2 | 7 | 0.00 | 3.50 |
| Chr2:196222822-196222844 | 196222839 | 3 | DNA | GGCCGAG-GTCGACTACCGGCNRG | GttCGAGCGTCaACgAtCGGCAGG | + | 5 | 1 | 7 | 0.00 | 3.50 |
| Chr8:115703111-115703133 | 115703128 | 3 | RNA | GGCCGAGGTCGACTACCGGCNRG | GGgC-AGGcCGAgcACCaGCCGG | + | 5 | 1 | 7 | 0.00 | 3.50 |
| Chr1:182096085-182096107 | 182096092 | 5 | RNA | GGCCGAGGTCGACTACCGGCNRG | G--CGAGGTCGtCcgtCtGCGAG | - | 5 | 2 | 7 | 0.00 | 3.50 |
| Chr1:58868607-58868629 | 58868624 | 3 | RNA | GGCCGAGGTCGACTACCGGCNRG | G-CtGAGGcCGACTtCaGaCGAG | + | 5 | 1 | 7 | 0.00 | 3.50 |
| Chr10:73019873-73019895 | 73019880 | 5 | RNA | GGCCGAGGTCGACTACCGGCNRG | G-CtGAGaTCGcCTAgCtGCGGG | - | 5 | 1 | 7 | 0.00 | 3.50 |
| Chr2:188582221-188582243 | 188582238 | 3 | RNA | GGCCGAGGTCGACTACCGGCNRG | GGC--tcGcaGACTACCGcCTGG | + | 5 | 2 | 7 | 0.00 | 3.50 |
| Chr10:39089907-39089929 | 39089914 | 5 | RNA | GGCCGAGGTCGACTACCGGCNRG | GG--GAGGagGtaTACgGGCAAG | - | 5 | 2 | 7 | 0.00 | 3.50 |
| Chr1:101069060-101069082 | 101069067 | 5 | DNA | GGCCGAGGTCGACTACCGG--CNRG | GGCtGAGGgCGAtTgCCtGCTCCAG | - | 5 | 2 | 7 | 0.00 | 3.50 |
| Chr8:155249330-155249352 | 155249337 | 5 | RNA | GGCCGAGGTCGACTACCGGCNRG | tGCCGAtGT-GACTgtCGGaGAG | - | 5 | 1 | 7 | 0.00 | 3.50 |
| Chr4:60783612-60783634 | 60783619 | 5 | DNA | GGC--CGAGGTCGACTACCGGCNRG | GGCCTCGgGGgCtACacCCGGCGGG | - | 5 | 2 | 7 | 0.00 | 3.50 |
| Chr2:183742862-183742884 | 183742869 | 5 | RNA | GGCCGAGGTCGACTACCGGCNRG | GGC--ttGcaGACTACCGGtTGG | - | 5 | 2 | 7 | 0.00 | 3.50 |
| Chr4:194036935-194036957 | 194036942 | 5 | RNA | GGCCGAGGTCGACTACCGGCNRG | GGtCGAGGcgcACTAC-GGgTAG | - | 5 | 1 | 7 | 0.00 | 3.50 |
| Chr10:20678206-20678228 | 20678223 | 3 | RNA | GGCCGAGGTCGACTACCGGCNRG | GGC-GAtGaCGAactCCGGCGAG | + | 5 | 1 | 7 | 0.00 | 3.50 |
| Chr4:176549488-176549510 | 176549495 | 5 | RNA | GGCCGAGGTCGACTACCGGCNRG | caCC-AGGTCGcCTACCtGgAGG | - | 5 | 1 | 7 | 0.00 | 3.50 |
| Chr8:70163945-70163967 | 70163952 | 5 | RNA | GGCCGAGGTCGACTACCGGCNRG | GGCttgGGa--ACTACCGGaAGG | - | 5 | 2 | 7 | 0.00 | 3.50 |
| Chr5:218107646-218107668 | 218107653 | 5 | DNA | GGCCGAGGTCGACTA-CCGGCNRG | aGCCaAGGTCaACTAGCCGcaTGG | - | 5 | 1 | 7 | 0.00 | 3.50 |
| Chr5:128710966-128710988 | 128710983 | 3 | RNA | GGCCGAGGTCGACTACCGGCNRG | GaCaG-GGTCGACgACaaGCGGG | + | 5 | 1 | 7 | 0.00 | 3.50 |
| Chr3:78246029-78246051 | 78246036 | 5 | RNA | GGCCGAGGTCGACTACCGGCNRG | GGCCGAGcTgGAg--CtGGgAAG | - | 5 | 2 | 7 | 0.00 | 3.50 |
| Chr1:69482345-69482367 | 69482362 | 3 | RNA | GGCCGAGGTCGACTACCGGCNRG | GGC-GAGcaCctgTACCGGCCGG | + | 5 | 1 | 7 | 0.00 | 3.50 |
| Chr7:104890395-104890417 | 104890402 | 5 | RNA | GGCCGAGGTCGACTACCGGCNRG | GGCCGgGcTgGgCTg--GGCTGG | - | 5 | 2 | 7 | 0.00 | 3.50 |
| Chr1:290345962-290345984 | 290345979 | 3 | RNA | GGCCGAGGTCGACTACCGGCNRG | GGaCGAGG-CGACggaCtGCTGG | + | 5 | 1 | 7 | 0.00 | 3.50 |
| Chr3:59473846-59473868 | 59473853 | 5 | DNA | GGCCGAGGTCGACTA--CCGGCNRG | GGtCaAGGTCGACcAAGCCacCAAG | - | 5 | 2 | 7 | 0.00 | 3.50 |
| Chr8:111280383-111280405 | 111280390 | 5 | RNA | GGCCGAGGTCGACTACCGGCNRG | GGgCG-GGcCaACctCCGGCCGG | - | 5 | 1 | 7 | 0.00 | 3.50 |
| Chr5:32392655-32392677 | 32392672 | 3 | RNA | GGCCGAGGTCGACTACCGGCNRG | GttCGAGaT--ACTtCgGGCAAG | + | 5 | 2 | 7 | 0.00 | 3.50 |
| Chr6:119742892-119742914 | 119742909 | 3 | RNA | GGCCGAGGTCGACTACCGGCNRG | GtCttgGGT-GACcACCGGCGGG | + | 5 | 1 | 7 | 0.00 | 3.50 |
| Chr1:252608099-252608121 | 252608106 | 5 | RNA | GGCCGAGGTCGACTACCGGCNRG | caCCaAGtTCGcCT-CCGGCCAG | - | 5 | 1 | 7 | 0.00 | 3.50 |
| Chr6:142107425-142107447 | 142107442 | 3 | RNA | GGCCGAGGTCGACTACCGGCNRG | GGC--AcactGACcACCGGCGGG | + | 5 | 2 | 7 | 0.00 | 3.50 |
| Chr2:83285025-83285047 | 83285042 | 3 | RNA | GGCCGAGGTCGACTACCGGCNRG | GGCCcAG--CGAagACCcGgGGG | + | 5 | 2 | 7 | 0.00 | 3.50 |
| Chr7:69146935-69146957 | 69146942 | 5 | RNA | GGCCGAGGTCGACTACCGGCNRG | GGCCGgGtcgGAC--CgGGCCGG | - | 5 | 2 | 7 | 0.00 | 3.50 |
| Chr10:128566492-128566514 | 128566499 | 5 | RNA | GGCCGAGGTCGACTACCGGCNRG | GGgCGAaGgCaACT-CgGGCGGG | - | 5 | 1 | 7 | 0.00 | 3.50 |
| Chr4:82725659-82725681 | 82725666 | 5 | RNA | GGCCGAGGTCGACTACCGGCNRG | GGCgtgGGgCaACT-CCGGCGAG | - | 5 | 1 | 7 | 0.00 | 3.50 |
| Chr8:155273821-155273843 | 155273828 | 5 | RNA | GGCCGAGGTCGACTACCGGCNRG | aaaCGAG--aGACaACCGGCTGG | - | 5 | 2 | 7 | 0.00 | 3.50 |
| Chr3:152917112-152917134 | 152917129 | 3 | RNA | GGCCGAGGTCGACTACCGGCNRG | GGgCGgGGTtGgCT--CGGgGAG | + | 5 | 2 | 7 | 0.00 | 3.50 |
| Chr7:48025358-48025380 | 48025375 | 3 | DNA | GGCCGAGGT--CGACTACCGGCNRG | aGaCGAGGTTCCGACcAagGGCAGG | + | 5 | 2 | 7 | 0.00 | 3.50 |
| Chr8:123372560-123372582 | 123372577 | 3 | RNA | GGCCGAGGTCGACTACCGGCNRG | GGCgGAGG--GACagCtGtCGGG | + | 5 | 2 | 7 | 0.00 | 3.50 |
| Chr3:224266456-224266478 | 224266473 | 3 | RNA | GGCCGAGGTCGACTACCGGCNRG | GGC--tcGcgGACTACCGcCTGG | + | 5 | 2 | 7 | 0.00 | 3.50 |
| Chr4:145494802-145494824 | 145494809 | 5 | RNA | GGCCGAGGTCGACTACCGGCNRG | tGCCGAaGgaGACTACtG-CTGG | - | 5 | 1 | 7 | 0.00 | 3.50 |
| Chr8:124423370-124423392 | 124423377 | 5 | RNA | GGCCGAGGTCGACTACCGGCNRG | aGgaGAGGTgGACTACa--CCAG | - | 5 | 2 | 7 | 0.00 | 3.50 |
| Chr7:56578178-56578200 | 56578195 | 3 | RNA | GGCCGAGGTCGACTACCGGCNRG | GGCCGAtcTCG--cAtCaGCAAG | + | 5 | 2 | 7 | 0.00 | 3.50 |
| Chr7:151409140-151409162 | 151409147 | 5 | RNA | GGCCGAGGTCGACTACCGGCNRG | G-aCGgGGTCGACaACgaGCGGG | - | 5 | 1 | 7 | 0.00 | 3.50 |
| Chr5:114748179-114748201 | 114748186 | 5 | RNA | GGCCGAGGTCGACTACCGGCNRG | tagCGAGcTCG--TACtGGCCAG | - | 5 | 2 | 7 | 0.00 | 3.50 |
| Chr5:68831643-68831665 | 68831650 | 5 | RNA | GGCCGAGGTCGACTACCGGCNRG | GGa-GcGGgaGACaACCGGCTGG | - | 5 | 1 | 7 | 0.00 | 3.50 |
| Chr5:117397363-117397385 | 117397380 | 3 | RNA | GGCCGAGGTCGACTACCGGCNRG | GGC--tcGcgGACTACCGGCTGG | + | 4 | 2 | 7 | 0.00 | 3.50 |
| Chr8:59232310-59232332 | 59232317 | 5 | RNA | GGCCGAGGTCGACTACCGGCNRG | GGgCacGGTCGgCTACC--CTGG | - | 4 | 2 | 7 | 0.00 | 3.50 |
| Chr4:73384049-73384071 | 73384066 | 3 | RNA | GGCCGAGGTCGACTACCGGCNRG | GGC--AGGcCGAgcACCGGCCGG | + | 3 | 2 | 7 | 0.00 | 3.50 |
| Chr8:115764999-115765021 | 115765006 | 5 | DNA | GGCCGAGGTC-GACTACCGGCNRG | GGCCGAGGcCTGAggtCgGGCGAG | - | 5 | 1 | 7 | 0.00 | 3.50 |
| Chr7:74380977-74380999 | 74380994 | 3 | RNA | GGCCGAGGTCGACTACCGGCNRG | cGCCGAGGagGACcAg-GGCCGG | + | 5 | 1 | 7 | 0.00 | 3.50 |
| Chr10:67921131-67921153 | 67921148 | 3 | RNA | GGCCGAGGTCGACTACCGGCNRG | aGCaGgGcTCGACTAC-GGaCGG | + | 5 | 1 | 7 | 0.00 | 3.50 |
| Chr8:87053422-87053444 | 87053439 | 3 | DNA | GGCCGAGGTCGACTA-CCGGCNRG | cGtCGAGGTCcACcATCaGGCGGG | + | 5 | 1 | 7 | 0.00 | 3.50 |
| Chr5:82849175-82849197 | 82849182 | 5 | RNA | GGCCGAGGTCGACTACCGGCNRG | G-aCGgGGTCGACgACgaGCGGG | - | 5 | 1 | 7 | 0.00 | 3.50 |
| Chr2:170258467-170258489 | 170258484 | 3 | RNA | GGCCGAGGTCGACTACCGGCNRG | GGtCcAGGgC--CTtCCtGCGAG | + | 5 | 2 | 7 | 0.00 | 3.50 |
| Chr1:214812603-214812625 | 214812610 | 5 | DNA | GGCCGAGGTCGACTACCGG--CNRG | GGtCGAGGcCGAgTcCgGGGTCGGG | - | 5 | 2 | 7 | 0.00 | 3.50 |
| Chr1:275415405-275415427 | 275415422 | 3 | RNA | GGCCGAGGTCGACTACCGGCNRG | ttCCGAtGTCGAtTA--tGCCGG | + | 5 | 2 | 7 | 0.00 | 3.50 |
| Chr2:174401136-174401158 | 174401153 | 3 | RNA | GGCCGAGGTCGACTACCGGCNRG | G-aCGgGGTCGACaACgaGCGGG | + | 5 | 1 | 7 | 0.00 | 3.50 |
| Chr2:121584147-121584169 | 121584154 | 5 | RNA | GGCCGAGGTCGACTACCGGCNRG | G-CtGAGaTCGcCTAgCtGCGGG | - | 5 | 1 | 7 | 0.00 | 3.50 |
| Chr8:53101329-53101351 | 53101336 | 5 | RNA | GGCCGAGGTCGACTACCGGCNRG | GGC--AGGcCGAactCCGGCCGG | - | 4 | 2 | 7 | 0.00 | 3.50 |
| Chr10:43246327-43246349 | 43246334 | 5 | DNA | GGCCGAGGTCGACTACC--GGCNRG | GcCCGAGagCaACcACCGTGGCCAG | - | 5 | 2 | 7 | 0.00 | 3.50 |
| Chr5:128592874-128592896 | 128592891 | 3 | RNA | GGCCGAGGTCGACTACCGGCNRG | cGgCGAGcctG--TACCGGCTGG | + | 5 | 2 | 7 | 0.00 | 3.50 |
| Chr4:210515291-210515313 | 210515308 | 3 | RNA | GGCCGAGGTCGACTACCGGCNRG | GaCCGcG--CGtCTAgCGaCCAG | + | 5 | 2 | 7 | 0.00 | 3.50 |
| Chr1:88901509-88901531 | 88901526 | 3 | RNA | GGCCGAGGTCGACTACCGGCNRG | GGCCGAGcTC--CggCgaGCTGG | + | 5 | 2 | 7 | 0.00 | 3.50 |
| Chr4:29844905-29844927 | 29844912 | 5 | DNA | GGCCGAGGTCGACTA--CCGGCNRG | ctCCtAGGcCGACcAATCCGGCAGG | - | 5 | 2 | 7 | 0.00 | 3.50 |
| Chr9:37132733-37132755 | 37132740 | 5 | RNA | GGCCGAGGTCGACTACCGGCNRG | cGCCGgGGcCGAC-ACCGaCGGG | - | 4 | 1 | 7 | 0.00 | 3.50 |

| | | | | | | | | | | | |
|---|---|---|---|---|---|---|---|---|---|---|---|
| Chr10:101058734-101058756 | 101058741 | 5 | RNA | GGCCGAGGTCGACTACCGGCNRG | GtCCGAcGTaGcCT--CGGaCGG | - | 5 | 2 | 7 | 0.00 | 3.50 |
| Chr3:76087062-76087084 | 76087079 | 3 | RNA | GGCCGAGGTCGACTACCGGCNRG | GGgCGcGGTCGgCg--CGGgGAG | + | 5 | 2 | 7 | 0.00 | 3.50 |
| Chr2:109839679-109839701 | 109839696 | 3 | RNA | GGCCGAGGTCGACTACCGGCNRG | GGCCacGGgCGgCT-CtGGCGGG | + | 5 | 1 | 7 | 0.00 | 3.50 |
| Chr9:17097128-17097150 | 17097133 | 3 | RNA | GGCCGAGGTCGACTACCGGCNRG | GGC--tcGcgGACTACCGGCTGG | - | 4 | 2 | 2 | 4.96 | 3.48 |
| Chr2:118375013-118375035 | 118375018 | 3 | RNA | GGCCGAGGTCGACTACCGGCNRG | G--aGcGGgaGACgACCGGCTGG | - | 5 | 2 | 3 | 3.86 | 3.43 |
| Chr7:122722114-122722136 | 122722132 | 2 | RNA | GGCCGAGGTCGACTACCGGCNRG | GcCCGgGGa--AaTACCcGCTAG | + | 5 | 2 | 4 | 2.75 | 3.38 |
| Chr2:220603800-220603822 | 220603817 | 3 | RNA | GGCCGAGGTCGACTACCGGCNRG | tG--GAGGTCGACTgCgGtaCGG | + | 5 | 2 | 4 | 2.75 | 3.38 |
| Chr5:50331645-50331667 | 50331663 | 2 | RNA | GGCCGAGGTCGACTACCGGCNRG | GcCCatGGTCGAgTtC--GCCAG | + | 5 | 2 | 5 | 1.65 | 3.33 |
| Chr7:132883954-132883976 | 132883959 | 3 | RNA | GGCCGAGGTCGACTACCGGCNRG | G-aCGgGGTCGACaACgaGCGGG | - | 5 | 1 | 5 | 1.65 | 3.33 |
| Chr5:132656602-132656624 | 132656617 | 5 | RNA | GGCCGAGGTCGACTACCGGCNRG | GGCtGgGcTgGACTA--GGtTGG | + | 5 | 2 | 0 | 6.61 | 3.31 |
| Chr2:191357245-191357267 | 191357250 | 3 | RNA | GGCCGAGGTCGACTACCGGCNRG | GGC--AGGcCGggcACtGGCCGG | - | 5 | 2 | 0 | 6.61 | 3.31 |
| Chr7:112767413-112767435 | 112767420 | 5 | RNA | GGCCGAGGTCGACTACCGGCNRG | GtCCGgtGT--ACcACCGGaCAG | - | 5 | 2 | 0 | 6.61 | 3.31 |
| Chr4:209877700-209877722 | 209877705 | 3 | RNA | GGCCGAGGTCGACTACCGGCNRG | GGCCGAGGagGAaggC--GCGGG | - | 5 | 2 | 0 | 6.61 | 3.31 |
| Chr1:149914618-149914640 | 149914633 | 5 | RNA | GGCCGAGGTCGACTACCGGCNRG | aG--GAcGaCGACTAgCaGCAGG | + | 5 | 2 | 0 | 6.61 | 3.31 |
| Chr5:141994705-141994727 | 141994721 | 4 | RNA | GGCCGAGGTCGACTACCGGCNRG | cGgCGAcGTCGA--AgCtGCTGG | + | 5 | 2 | 0 | 6.61 | 3.31 |
| Chr1:62812157-62812179 | 62812175 | 2 | RNA | GGCCGAGGTCGACTACCGGCNRG | GcCgGAGccCGAC--CCGtCAAG | + | 5 | 2 | 0 | 6.61 | 3.31 |
| Chr7:150943166-150943188 | 150943171 | 3 | RNA | GGCCGAGGTCGACTACCGGCNRG | G-aCGgGGTCGACaACgaGCGGG | - | 5 | 1 | 0 | 6.61 | 3.31 |
| Chr8:436501-436523 | 436508 | 5 | RNA | GGCCGAGGTCGACTACCGGCNRG | cG--GtGGTCGACgAgCtGCTGG | - | 5 | 2 | 0 | 6.61 | 3.31 |
| Chr9:62104788-62104810 | 62104792 | 2 | DNA | GGCCGAGGTCGACTACCGG--CNRG | GGCaGAtaTCGcgTACCGGTTCAGG | - | 5 | 2 | 0 | 6.61 | 3.31 |
| Chr8:126611744-126611766 | 126611762 | 2 | RNA | GGCCGAGGTCGACTACCGGCNRG | GGCCctGGTC--CcACCtGCAGG | + | 4 | 2 | 0 | 6.61 | 3.31 |
| Chr7:154322906-154322928 | 154322913 | 5 | RNA | GGCCGAGGTCGACTACCGGCNRG | GGCCGAGGgtGcCT-CgGGCTGG | - | 4 | 1 | 0 | 6.61 | 3.31 |
| Chr8:104798108-104798130 | 104798125 | 3 | RNA | GGCCGAGGTCGACTACCGGCNRG | caCCaAGtTCGcCT-CCGGCCAG | + | 5 | 1 | 0 | 6.61 | 3.31 |
| Chr3:210542609-210542631 | 210542614 | 3 | DNA | GGCCGAGGTCGACTA--CCGGCNRG | ctCCtAGGcCGACcAATCCGGCAGG | - | 5 | 2 | 0 | 6.61 | 3.31 |
| Chr2:13460991-13461013 | 13460996 | 3 | RNA | GGCCGAGGTCGACTACCGGCNRG | GGgCG-GGcCaACctCCGGCCGG | - | 5 | 1 | 0 | 6.61 | 3.31 |
| Chr2:118665339-118665361 | 118665343 | 2 | RNA | GGCCGAGGTCGACTACCGGCNRG | GGtCcAGGagGA--ACaGGCAAG | - | 5 | 2 | 0 | 6.61 | 3.31 |
| Chr1:706593-706615 | 706597 | 2 | DNA | GGCCGAGGTCGACTA--CCGGCNRG | GaCCGgaGTCGACgAAGCCGGtGGG | - | 5 | 2 | 0 | 6.61 | 3.31 |
| Chr2:170420095-170420117 | 170420100 | 3 | DNA | GGCCGAGGTCGACTA--CCGGCNRG | ctCCtAGGcCGACcAATCCGGCAGG | - | 5 | 2 | 0 | 6.61 | 3.31 |
| Chr8:154675293-154675315 | 154675310 | 3 | DNA | G-GCCGAGGTCGACTACCGGCNRG | GCGCCtcGGgCGAgTACtGGCAGG | + | 5 | 1 | 0 | 6.61 | 3.31 |
| Chr4:140230920-140230942 | 140230938 | 2 | RNA | GGCCGAGGTCGACTACCGGCNRG | a--CGgGGTCGACaACgaGCGGG | + | 5 | 2 | 0 | 6.61 | 3.31 |
| Chr4:93762624-93762646 | 93762639 | 5 | RNA | GGCCGAGGTCGACTACCGGCNRG | cGgCGAGcctGA--ACCGGCCGG | + | 5 | 2 | 0 | 6.61 | 3.31 |
| Chr7:110131344-110131366 | 110131350 | 4 | RNA | GGCCGAGGTCGACTACCGGCNRG | cG--GAGGggGAaTACCtGCTGG | - | 5 | 2 | 0 | 6.61 | 3.31 |
| Chr7:117228090-117228112 | 117228094 | 2 | RNA | GGCCGAGGTCGACTACCGGCNRG | cGaCGAtGTCGACcACC--tGAG | - | 5 | 2 | 0 | 6.61 | 3.31 |
| Chr5:72879057-72879079 | 72879072 | 5 | RNA | GGCCGAGGTCGACTACCGGCNRG | GGCCGAaaTCccCTcCC--CTGG | + | 5 | 2 | 0 | 6.61 | 3.31 |
| Chr7:113449336-113449358 | 113449351 | 5 | RNA | GGCCGAGGTCGACTACCGGCNRG | GGC--tcGTgGACTACCGGCTGG | + | 3 | 2 | 0 | 6.61 | 3.31 |
| Chr2:132941984-132942006 | 132941990 | 4 | RNA | GGCCGAGGTCGACTACCGGCNRG | tGCCGAGGT-GACaAagGaCAGG | - | 5 | 1 | 0 | 6.61 | 3.31 |
| Chr1:286460736-286460758 | 286460742 | 4 | RNA | GGCCGAGGTCGACTACCGGCNRG | GGaCcAGGTCG--TcCCaGtTGG | - | 5 | 2 | 0 | 6.61 | 3.31 |
| Chr1:36204131-36204153 | 36204136 | 3 | RNA | GGCCGAGGTCGACTACCGGCNRG | GGgCG-GGcCaACccCCGGCCGG | - | 5 | 1 | 0 | 6.61 | 3.31 |
| Chr3:134841581-134841603 | 134841596 | 5 | RNA | GGCCGAGGTCGACTACCGGCNRG | GGgCG-GGcCaACccCCGGCCGG | + | 5 | 1 | 0 | 6.61 | 3.31 |
| Chr7:107967449-107967471 | 107967464 | 5 | RNA | GGCCGAGGTCGACTACCGGCNRG | GGCCGAGcgCGA--AggGGgGAG | + | 5 | 2 | 0 | 6.61 | 3.31 |
| Chr1:246890071-246890093 | 246890076 | 3 | RNA | GGCCGAGGTCGACTACCGGCNRG | G-aCGgGGTCGACaACaaGCGGG | - | 5 | 1 | 0 | 6.61 | 3.31 |
| Chr1:134587246-134587268 | 134587251 | 3 | RNA | GGCCGAGGTCGACTACCGGCNRG | acCCGAGcTCGAgTtC--GCCGG | - | 5 | 2 | 0 | 6.61 | 3.31 |
| Chr8:76911959-76911981 | 76911965 | 4 | DNA | GGCCGAGGTCGACTAC--CGGCNRG | tGaCGAGGgCGACgACGGCGaCAAG | - | 5 | 2 | 0 | 6.61 | 3.31 |
| Chr5:223334173-223334195 | 223334188 | 5 | RNA | GGCCGAGGTCGACTACCGGCNRG | GGa-GAGGaaGACgAgCGGCGGG | + | 5 | 1 | 0 | 6.61 | 3.31 |
| Chr5:31582421-31582443 | 31582436 | 5 | DNA | GGC--CGAGGTCGACTACCGGCNRG | GGCCTCGgGGgCtACacCCGGCGGG | + | 5 | 2 | 0 | 6.61 | 3.31 |
| Chr1:62811430-62811452 | 62811435 | 3 | RNA | GGCCGAGGTCGACTACCGGCNRG | G-aCGgGGTCGACgACgaGCGGG | - | 5 | 1 | 0 | 6.61 | 3.31 |
| Chr8:26953026-26953048 | 26953042 | 4 | RNA | GGCCGAGGTCGACTACCGGCNRG | G-aCGgGGTCGACaACgaGCGGG | + | 5 | 1 | 0 | 6.61 | 3.31 |
| Chr2:158280105-158280127 | 158280109 | 2 | RNA | GGCCGAGGTCGACTACCGGCNRG | GGCCcAc--CGACTgCCGcgGGG | - | 5 | 2 | 0 | 6.61 | 3.31 |
| Chr2:167850650-167850672 | 167850657 | 5 | RNA | GGCCGAGGTCGACTACCGGCNRG | GttCGAGcTCG--TACtGGaCAG | - | 5 | 2 | 0 | 6.61 | 3.31 |
| Chr4:222910238-222910260 | 222910256 | 2 | RNA | GGCCGAGGTCGACTACCGGCNRG | GtCCG--GaaGAgTACCGGaAAG | + | 5 | 2 | 0 | 6.61 | 3.31 |
| Chr10:42821618-42821640 | 42821624 | 4 | RNA | GGCCGAGGTCGACTACCGGCNRG | G--CGtGGatGACTACCGcgTGG | - | 5 | 2 | 0 | 6.61 | 3.31 |
| Chr2:26710193-26710215 | 26710197 | 2 | RNA | GGCCGAGGTCGACTACCGGCNRG | GGgCGgGGTCGACgAC-GcgAGG | - | 5 | 1 | 0 | 6.61 | 3.31 |
| Chr4:39844606-39844628 | 39844621 | 5 | RNA | GGCCGAGGTCGACTACCGGCNRG | GGC--tcGagGACTACCGGCTGG | + | 4 | 2 | 0 | 6.61 | 3.31 |
| Chr1:242800982-242801004 | 242800999 | 3 | RNA | GGCCGAGGTCGACTACCGGCNRG | G--tGAGGctGAgcACCGGCCAG | + | 5 | 2 | 0 | 6.61 | 3.31 |
| Chr1:119652003-119652025 | 119652009 | 4 | DNA | GGC-CGAGGTCGACTACCGGCNRG | GcCTCGAGGaCGtCTtCCaGCTGG | - | 5 | 1 | 0 | 6.61 | 3.31 |
| Chr4:195261284-195261306 | 195261289 | 3 | RNA | GGCCGAGGTCGACTACCGGCNRG | GGCtcAc--aGACTACCGGCTGG | - | 4 | 2 | 0 | 6.61 | 3.31 |
| Chr10:36951947-36951969 | 36951963 | 4 | RNA | GGCCGAGGTCGACTACCGGCNRG | GcCgGAGccCGAC--CCGtCAAG | + | 5 | 2 | 0 | 6.61 | 3.31 |
| Chr3:26127355-26127377 | 26127371 | 4 | RNA | GGCCGAGGTCGACTACCGGCNRG | aaCaGgGGTCGACTA--GGgGAG | + | 5 | 2 | 0 | 6.61 | 3.31 |
| Chr1:29483704-29483726 | 29483708 | 2 | DNA | GGC-CGAGGTCGACTACCGGCNRG | cGCTCGAGGTCaACctCgGGCGAG | - | 5 | 1 | 0 | 6.61 | 3.31 |
| Chr9:58043514-58043536 | 58043521 | 5 | RNA | GGCCGAGGTCGACTACCGGCNRG | GGCCGAGcTC-ACccCCtGCCAG | - | 4 | 1 | 0 | 6.61 | 3.31 |
| Chr3:232572372-232572394 | 232572390 | 2 | RNA | GGCCGAGGTCGACTACCGGCNRG | tGCgGAGGTgGACa--CcGCGAG | + | 5 | 2 | 0 | 6.61 | 3.31 |
| Chr2:4237640-4237662 | 4237644 | 2 | DNA | GGCC-GAGGTCGACTACCGGCNRG | GtCCAGAGGaCGACacaCGGCCGG | - | 5 | 1 | 0 | 6.61 | 3.31 |
| Chr3:226146250-226146272 | 226146254 | 2 | RNA | GGCCGAGGTCGACTACCGGCNRG | GcCCGAGGcCGA--ACtcGgTGG | - | 5 | 2 | 0 | 6.61 | 3.31 |
| Chr2:228863194-228863216 | 228863212 | 2 | RNA | GGCCGAGGTCGACTACCGGCNRG | GcCgGAGccCGAC--CCGtCAGG | + | 5 | 2 | 0 | 6.61 | 3.31 |
| Chr8:160556769-160556791 | 160556774 | 3 | RNA | GGCCGAGGTCGACTACCGGCNRG | G-aCGgGGTCGACaACaaGCGGG | - | 5 | 1 | 0 | 6.61 | 3.31 |
| Chr9:78187110-78187132 | 78187116 | 4 | RNA | GGCCGAGGTCGACTACCGGCNRG | cGCCGtGGTCGcCg--CGGCAGG | - | 4 | 2 | 0 | 6.61 | 3.31 |
| Chr5:179994095-179994117 | 179994099 | 2 | RNA | GGCCGAGGTCGACTACCGGCNRG | GtCttgGGT-GACcACCGGCGGG | - | 5 | 1 | 0 | 6.61 | 3.31 |
| Chr1:262657980-262658002 | 262657997 | 3 | RNA | GGCCGAGGTCGACTACCGGCNRG | GGCCGAGGgCtA-gACCcGtGGG | + | 5 | 1 | 0 | 6.61 | 3.31 |
| Chr6:48834209-48834231 | 48834213 | 2 | DNA | GGCCGAGGTCGACTACCGG-CNRG | GGCCGAGtTgGcCTggCGGCCTAG | - | 5 | 1 | 0 | 6.61 | 3.31 |
| Chr1:173662124-173662146 | 173662141 | 3 | RNA | GGCCGAGGTCGACTACCGGCNRG | GGC--AGGcCaACctCCGGCTGG | + | 4 | 2 | 0 | 6.61 | 3.31 |
| Chr2:129399458-129399480 | 129399474 | 4 | DNA | GGCCGAGGTCGAC--TACCGGCNRG | GcgCcAtcTCGACTCTACCGGCGAG | + | 5 | 2 | 0 | 6.61 | 3.31 |
| Chr9:76346098-76346120 | 76346113 | 5 | RNA | GGCCGAGGTCGACTACCGGCNRG | GaCgaAGGTgGACTA--GtCAGG | + | 5 | 2 | 0 | 6.61 | 3.31 |
| Chr7:56579233-56579255 | 56579239 | 4 | RNA | GGCCGAGGTCGACTACCGGCNRG | GGCCGAtcTCG--cAtCaGCAAG | - | 5 | 2 | 0 | 6.61 | 3.31 |
| Chr8:59924109-59924131 | 59924124 | 5 | DNA | GGCCGAG--GTCGACTACCGGCNRG | GctCGAGCGGgaGACgACCGGCTGG | + | 5 | 2 | 0 | 6.61 | 3.31 |
| Chr8:126646991-126647013 | 126646995 | 2 | RNA | GGCCGAGGTCGACTACCGGCNRG | GGCCGAGGTCG--ggCgaGgCGG | - | 5 | 2 | 0 | 6.61 | 3.31 |
| Chr3:152155382-152155404 | 152155388 | 4 | DNA | GGC--CGAGGTCGACTACCGGCNRG | GGCCTCagGGaCGtCTtCCGGCAAG | - | 5 | 2 | 0 | 6.61 | 3.31 |
| Chr1:32038881-32038903 | 32038897 | 4 | RNA | GGCCGAGGTCGACTACCGGCNRG | G-aCGgGGTCGACaACgaGCGGG | + | 5 | 1 | 0 | 6.61 | 3.31 |
| Chr5:92606775-92606797 | 92606790 | 5 | RNA | GGCCGAGGTCGACTACCGGCNRG | GGCCatGGcCGA--ACCcGCCAG | + | 4 | 2 | 0 | 6.61 | 3.31 |
| Chr6:84303473-84303495 | 84303477 | 2 | RNA | GGCCGAGGTCGACTACCGGCNRG | GcCCGAGccCGAgT--CGGtAGG | - | 5 | 2 | 0 | 6.61 | 3.31 |
| Chr2:22314149-22314171 | 22314154 | 3 | RNA | GGCCGAGGTCGACTACCGGCNRG | cGtCGAGGTCcA-gACtGGCAAG | - | 5 | 1 | 0 | 6.61 | 3.31 |
| Chr1:20374787-20374809 | 20374802 | 5 | RNA | GGCCGAGGTCGACTACCGGCNRG | GGC--AGGcCGAgcACtcGCCGG | + | 5 | 2 | 0 | 6.61 | 3.31 |
| Chr2:190325651-190325673 | 190325657 | 4 | RNA | GGCCGAGGTCGACTACCGGCNRG | tGtaGAGtT-GAaTACCGGCTGG | - | 5 | 1 | 0 | 6.61 | 3.31 |
| Chr4:62678695-62678717 | 62678701 | 4 | DNA | GGC-CGAGGTCGACTACCGGCNRG | GcCTCGgGGaCGtCTtCCGGCTGG | - | 5 | 1 | 0 | 6.61 | 3.31 |
| Chr1:116873093-116873115 | 116873111 | 2 | RNA | GGCCGAGGTCGACTACCGGCNRG | GG--GAtGgCGAgcACCGGtGAG | + | 5 | 2 | 0 | 6.61 | 3.31 |
| Chr3:26264873-26264895 | 26264878 | 3 | RNA | GGCCGAGGTCGACTACCGGCNRG | G-aCGgGGTCGACaACgaGCGGG | - | 5 | 1 | 0 | 6.61 | 3.31 |
| Chr2:149630338-149630360 | 149630356 | 2 | RNA | GGCCGAGGTCGACTACCGGCNRG | GGC--AGGcCaACctCCGGCCGG | + | 4 | 2 | 0 | 6.61 | 3.31 |
| Chr1:117372166-117372188 | 117372182 | 4 | RNA | GGCCGAGGTCGACTACCGGCNRG | GGC-GAGGTCGgCcACCaGgGAG | + | 4 | 1 | 0 | 6.61 | 3.31 |
| Chr2:217698302-217698324 | 217698319 | 3 | RNA | GGCCGAGGTCGACTACCGGCNRG | GGgCGccGgCGA--ACCaGCAGG | + | 5 | 2 | 0 | 6.61 | 3.31 |
| Chr3:160982243-160982265 | 160982250 | 5 | RNA | GGCCGAGGTCGACTACCGGCNRG | GGC--tcGcaGACTACCGcCTGG | - | 5 | 2 | 0 | 6.61 | 3.31 |
| Chr2:162923118-162923140 | 162923133 | 5 | RNA | GGCCGAGGTCGACTACCGGCNRG | GGCCGAGccCGA-gAgtGGCCAG | + | 5 | 1 | 0 | 6.61 | 3.31 |
| Chr1:101470370-101470392 | 101470375 | 3 | DNA | GGCCGAG-GTCGACTACCGGCNRG | GttCGAGCGTCaACgAtCGGCAGG | - | 5 | 1 | 0 | 6.61 | 3.31 |
| Chr9:15776841-15776863 | 15776859 | 2 | DNA | GGCCGAGGTCGACTACC--GGCNRG | GGCgGAGGTCGtCTgCaGAGGCCAG | + | 4 | 2 | 0 | 6.61 | 3.31 |
| Chr4:200141171-200141193 | 200141176 | 3 | DNA | GGCCGAGGTCGACTA--CCGGCNRG | cGtaaAGGTCGACcAATCCGGCAGG | - | 5 | 2 | 0 | 6.61 | 3.31 |
| Chr1:168558804-168558826 | 168558809 | 3 | RNA | GGCCGAGGTCGACTACCGGCNRG | G-aCGAGGTCGACaACgaGCGGG | - | 4 | 1 | 0 | 6.61 | 3.31 |
| Chr2:206435290-206435312 | 206435296 | 4 | RNA | GGCCGAGGTCGACTACCGGCNRG | G-CCtcGGgCGAaTACtGGCAGG | - | 5 | 1 | 0 | 6.61 | 3.31 |
| Chr1:68850688-68850710 | 68850695 | 5 | RNA | GGCCGAGGTCGACTACCGGCNRG | GGC--AGGcCaACctCCGGCCGG | - | 4 | 2 | 0 | 6.61 | 3.31 |
| Chr5:28838900-28838922 | 28838915 | 5 | RNA | GGCCGAGGTCGACTACCGGCNRG | GGtCGcGGaCGAgT--CGGCGGG | + | 4 | 2 | 0 | 6.61 | 3.31 |
| Chr3:73329873-73329895 | 73329878 | 3 | DNA | GGCCGAGGTCGACTA--CCGGCNRG | GGgCGgGaTgGACaACTCCGGCGAG | - | 5 | 2 | 6 | 0.55 | 3.28 |
| Chr1:129429467-129429489 | 129429483 | 4 | DNA | GGCCGAGGTCGACTACC--GGCNRG | GcCCGAGagCaACcACCATGGCCAG | + | 5 | 2 | 6 | 0.55 | 3.28 |
| Chr4:83512534-83512556 | 83512552 | 2 | DNA | GGCCGAGGTCGA-CTACCGGCNRG | GaCtcAGGTCGATCTggCGGCGGG | + | 5 | 1 | 6 | 0.55 | 3.28 |
| Chr4:183244851-183244873 | 183244856 | 3 | RNA | GGCCGAGGTCGACTACCGGCNRG | G-aCGgGGTCGACaACgaGCGGG | - | 5 | 1 | 6 | 0.55 | 3.28 |
| Chr6:113403735-113403757 | 113403740 | 3 | RNA | GGCCGAGGTCGACTACCGGCNRG | GGC--ttGcgGACTACCGGCTGG | - | 4 | 2 | 6 | 0.55 | 3.28 |
| Chr5:65009067-65009089 | 65009083 | 4 | RNA | GGCCGAGGTCGACTACCGGCNRG | G-aCGgGaTCGACaACCaGCGGG | + | 5 | 1 | 2 | 4.41 | 3.21 |
| Chr7:43329720-43329742 | 43329725 | 3 | RNA | GGCCGAGGTCGACTACCGGCNRG | t--CttcGTCGACTACCGGCGGG | - | 4 | 2 | 2 | 4.41 | 3.21 |
| Chr4:44208712-44208734 | 44208727 | 5 | RNA | GGCCGAGGTCGACTACCGGCNRG | GtC--tccTtGACTACCGGCCGG | + | 5 | 2 | 3 | 3.30 | 3.15 |
| Chr8:130821435-130821457 | 130821440 | 3 | RNA | GGCCGAGGTCGACTACCGGCNRG | GGC--AGGcCGAgcACCGGCCGG | - | 3 | 2 | 4 | 2.20 | 3.10 |
| Chr5:81287185-81287207 | 81287191 | 4 | RNA | GGCCGAGGTCGACTACCGGCNRG | GGC--ttGcgGACTACCGGCTGG | - | 4 | 2 | 4 | 2.20 | 3.10 |
| Chr4:226970908-226970930 | 226970914 | 4 | RNA | GGCCGAGGTCGACTACCGGCNRG | GGgCGtGGTgGACcACC--aCGG | - | 5 | 2 | 4 | 2.20 | 3.10 |
| Chr10:31757182-31757204 | 31757189 | 5 | RNA | GGCCGAGGTCGACTACCGGCNRG | aGCaGgGcTCGACTAC-GGaCGG | - | 5 | 1 | 5 | 1.10 | 3.05 |
| Chr2:216234751-216234773 | 216234756 | 3 | RNA | GGCCGAGGTCGACTACCGGCNRG | G-aCGgGGTCGACaACgaGCGGG | - | 5 | 1 | 5 | 1.10 | 3.05 |
| Chr1:245881204-245881226 | 245881209 | 3 | RNA | GGCCGAGGTCGACTACCGGCNRG | G-aCGgGGTCGACaACgaGCGGG | - | 5 | 1 | 0 | 6.06 | 3.03 |
| Chr2:66950351-66950373 | 66950367 | 4 | RNA | GGCCGAGGTCGACTACCGGCNRG | GaCaG-GGTCGACgACaaGCGGG | + | 5 | 1 | 0 | 6.06 | 3.03 |
| Chr4:154841632-154841654 | 154841648 | 4 | RNA | GGCCGAGGTCGACTACCGGCNRG | G-aCGgGGTCGACaACgaGCGGG | + | 5 | 1 | 0 | 6.06 | 3.03 |
| Chr4:39033658-39033680 | 39033664 | 4 | RNA | GGCCGAGGTCGACTACCGGCNRG | aGt--AGGaCGACTACgaGCAGG | - | 5 | 2 | 0 | 6.06 | 3.03 |
| Chr5:33134336-33134358 | 33134351 | 5 | RNA | GGCCGAGGTCGACTACCGGCNRG | GGCgGAGG--GACaACtGtCGGG | + | 4 | 2 | 0 | 6.06 | 3.03 |
| Chr7:6227987-6228009 | 6228005 | 2 | RNA | GGCCGAGGTCGACTACCGGCNRG | GGCtGgGcTgGACTA--GGtTGG | + | 5 | 2 | 0 | 6.06 | 3.03 |
| Chr4:65506444-65506466 | 65506462 | 2 | RNA | GGCCGAGGTCGACTACCGGCNRG | GGCCG--GTCcACTttCGaaAAG | + | 5 | 2 | 0 | 6.06 | 3.03 |
| Chr2:40662144-40662166 | 40662160 | 4 | RNA | GGCCGAGGTCGACTACCGGCNRG | G-aCGgGGTCGACaACgaGCGGG | + | 5 | 1 | 0 | 6.06 | 3.03 |
| Chr9:93885367-93885389 | 93885383 | 4 | RNA | GGCCGAGGTCGACTACCGGCNRG | tGCCGAtGTgG--cACCGGaGAG | + | 5 | 2 | 0 | 6.06 | 3.03 |
| Chr1:269001932-269001954 | 269001938 | 4 | RNA | GGCCGAGGTCGACTACCGGCNRG | cGCgGcGG-CGACTACtGGCCAG | - | 4 | 1 | 0 | 6.06 | 3.03 |
| Chr2:7791386-7791408 | 7791403 | 3 | RNA | GGCCGAGGTCGACTACCGGCNRG | GGaCcAGGTCG--TcCCaGtTGG | + | 5 | 2 | 0 | 6.06 | 3.03 |
| Chr9:89937215-89937237 | 89937230 | 5 | RNA | GGCCGAGGTCGACTACCGGCNRG | GGC--tcGcgGACTACCGGCTGG | + | 4 | 2 | 0 | 6.06 | 3.03 |
| Chr1:142432589-142432611 | 142432593 | 2 | RNA | GGCCGAGGTCGACTACCGGCNRG | GGCCaAGcTCaAaTg--GGCCAG | - | 5 | 2 | 0 | 6.06 | 3.03 |
| Chr7:166764771-166764793 | 166764775 | 2 | RNA | GGCCGAGGTCGACTACCGGCNRG | GcCCGAGGcCGA--ACtcGgTGG | - | 5 | 2 | 0 | 6.06 | 3.03 |
| Chr6:55724835-55724857 | 55724841 | 4 | X | GGCCGAGGTCGACTACCGGCNRG | GGCaGAGGaCGAacAaCGGCGAG | - | 5 | 0 | 0 | 6.06 | 3.03 |
| Chr1:174719672-174719694 | 174719677 | 3 | RNA | GGCCGAGGTCGACTACCGGCNRG | GGCgGAGGTC--CaAaCctCTGG | - | 5 | 2 | 0 | 6.06 | 3.03 |
| Chr2:80020140-80020162 | 80020158 | 2 | RNA | GGCCGAGGTCGACTACCGGCNRG | cG--GAGGTgaAtcACCGGCCAG | + | 5 | 2 | 0 | 6.06 | 3.03 |

| | | | | | | | | | | | |
|---|---|---|---|---|---|---|---|---|---|---|---|
| Chr5:47727559-47727581 | 47727566 | 5 | DNA | GGCCGAGGTCGACT--ACCGGCNRG | GGCCtAGGTacAtTGAAaCGGCCAG | - | 5 | 2 | 0 | 6.06 | 3.03 |
| Chr8:94051057-94051079 | 94051073 | 4 | RNA | GGCCGAGGTCGACTACCGGCNRG | GGCCGAGGTCG--ggCgaGgCGG | + | 5 | 2 | 0 | 6.06 | 3.03 |
| Chr1:290316383-290316405 | 290316387 | 2 | RNA | GGCCGAGGTCGACTACCGGCNRG | GGCCGAGGTCG--ggCgaGgCGG | - | 5 | 2 | 0 | 6.06 | 3.03 |
| Chr4:180668802-180668824 | 180668818 | 4 | RNA | GGCCGAGGTCGACTACCGGCNRG | G-aCGgGGTCGACgACaaGCGGG | + | 5 | 1 | 0 | 6.06 | 3.03 |
| Chr1:39102091-39102113 | 39102109 | 2 | RNA | GGCCGAGGTCGACTACCGGCNRG | c--CGAGGTCaACcACCacCAAG | + | 5 | 2 | 0 | 6.06 | 3.03 |
| Chr2:164201552-164201574 | 164201558 | 4 | DNA | GGC-CGAGGTCGACTACCGGCNRG | GcCTCGAGGaCGtCTtCCGGCTGG | - | 4 | 1 | 0 | 6.06 | 3.03 |
| Chr10:49463482-49463504 | 49463489 | 5 | RNA | GGCCGAGGTCGACTACCGGCNRG | caCCaAGG--GACTgCCGtCGAG | - | 5 | 2 | 0 | 6.06 | 3.03 |
| Chr5:119153448-119153470 | 119153466 | 2 | RNA | GGCCGAGGTCGACTACCGGCNRG | GGCCGAGGTgGgtTgCC--tGAG | + | 5 | 2 | 0 | 6.06 | 3.03 |
| Chr10:57989987-57990009 | 57990002 | 5 | RNA | GGCCGAGGTCGACTACCGGCNRG | GGC--tcGcgGACTACCGtCTGG | + | 5 | 2 | 0 | 6.06 | 3.03 |
| Chr1:257619477-257619499 | 257619494 | 3 | RNA | GGCCGAGGTCGACTACCGGCNRG | GGCCGAcGagt--gACCGGCTGG | + | 5 | 2 | 0 | 6.06 | 3.03 |
| Chr9:59711973-59711995 | 59711988 | 5 | RNA | GGCCGAGGTCGACTACCGGCNRG | GtC--tccTtGACTACCGGCCGG | + | 5 | 2 | 0 | 6.06 | 3.03 |
| Chr5:735500-735522 | 735516 | 4 | RNA | GGCCGAGGTCGACTACCGGCNRG | GGC-GAGGTCGAggACCttgGGG | + | 5 | 1 | 0 | 6.06 | 3.03 |
| Chr3:79080994-79081016 | 79081010 | 4 | RNA | GGCCGAGGTCGACTACCGGCNRG | GGtCGtGGTCGAC--CgGaaCGG | + | 5 | 2 | 0 | 6.06 | 3.03 |
| Chr5:182735082-182735104 | 182735086 | 2 | RNA | GGCCGAGGTCGACTACCGGCNRG | GG--GAGGaaGgCgACCGGgAAG | - | 5 | 2 | 0 | 6.06 | 3.03 |
| Chr9:132573667-132573689 | 132573672 | 3 | RNA | GGCCGAGGTCGACTACCGGCNRG | G-aCGgGGTCGACaACgaGCGGG | - | 5 | 1 | 0 | 6.06 | 3.03 |
| Chr5:61844852-61844874 | 61844856 | 2 | RNA | GGCCGAGGTCGACTACCGGCNRG | GGCgGAGaTCGtCTtCC--gGGG | - | 5 | 2 | 0 | 6.06 | 3.03 |
| Chr3:199670664-199670686 | 199670679 | 5 | RNA | GGCCGAGGTCGACTACCGGCNRG | cGC--tcGTgGACTACCGGCTGG | + | 4 | 2 | 0 | 6.06 | 3.03 |
| Chr10:73463374-73463396 | 73463381 | 5 | RNA | GGCCGAGGTCGACTACCGGCNRG | GGt-GtGGTCGAggACtGGCGAG | - | 5 | 1 | 0 | 6.06 | 3.03 |
| Chr3:45464703-45464725 | 45464710 | 5 | DNA | GGCCGAGGTCGACTACCGG--CNRG | GGtCGAGGTCGAgTcCgaGCCCTGG | - | 5 | 2 | 0 | 6.06 | 3.03 |
| Chr1:109199431-109199453 | 109199448 | 3 | RNA | GGCCGAGGTCGACTACCGGCNRG | G--CGAcGTCaAtTACCGcaTGG | + | 5 | 2 | 0 | 6.06 | 3.03 |
| Chr2:106623030-106623052 | 106623034 | 2 | RNA | GGCCGAGGTCGACTACCGGCNRG | GGCCGAGG-CGcgTgggGGCGGG | - | 5 | 1 | 0 | 6.06 | 3.03 |
| Chr5:55733352-55733374 | 55733357 | 3 | RNA | GGCCGAGGTCGACTACCGGCNRG | GGgtGgGGgCGA--ACCGGCCGG | - | 4 | 2 | 0 | 6.06 | 3.03 |
| Chr7:52762499-52762521 | 52762517 | 2 | DNA | GGCC-GAGGTCGACTACCGGCNRG | GGCCAGgGGagGACTACtGGCGGG | + | 4 | 1 | 0 | 6.06 | 3.03 |
| Chr2:40021028-40021050 | 40021034 | 4 | RNA | GGCCGAGGTCGACTACCGGCNRG | t--CGAGGcCtACacCCGGCGGG | - | 5 | 2 | 0 | 6.06 | 3.03 |
| Chr8:33512836-33512858 | 33512853 | 3 | DNA | GGCCGAGGTCGACT--ACCGGCNRG | GGtgGAGGTgGgCTGGAgCGGCCGG | + | 5 | 2 | 0 | 6.06 | 3.03 |
| Chr5:180616727-180616749 | 180616734 | 5 | RNA | GGCCGAGGTCGACTACCGGCNRG | caCCaAGtTCGcCT-CCGGCCAG | - | 5 | 1 | 0 | 6.06 | 3.03 |
| Chr8:45366222-45366244 | 45366237 | 5 | RNA | GGCCGAGGTCGACTACCGGCNRG | GGgC-AGGcCaACctCCGGCCGG | + | 5 | 1 | 0 | 6.06 | 3.03 |
| Chr9:97776243-97776265 | 97776250 | 5 | RNA | GGCCGAGGTCGACTACCGGCNRG | GGCCGAatTCaACT-CCtGgAGG | - | 5 | 1 | 0 | 6.06 | 3.03 |
| Chr2:204428188-204428210 | 204428195 | 5 | RNA | GGCCGAGGTCGACTACCGGCNRG | GGC--AGGcCagCctCCGGCCGG | - | 5 | 2 | 0 | 6.06 | 3.03 |
| Chr9:4324450-4324472 | 4324455 | 3 | DNA | GG--CCGAGGTCGACTACCGGCNRG | GGATCCaAGaTCcACTtCaGGCAAG | - | 5 | 2 | 0 | 6.06 | 3.03 |
| Chr6:61842309-61842331 | 61842314 | 3 | RNA | GGCCGAGGTCGACTACCGGCNRG | GGtCGtGcTCGAC-ACgaGCTGG | - | 5 | 1 | 0 | 6.06 | 3.03 |
| Chr8:51395291-51395313 | 51395309 | 2 | RNA | GGCCGAGGTCGACTACCGGCNRG | GGC--AGcTCGgCTcCCtGCTGG | + | 4 | 2 | 0 | 6.06 | 3.03 |
| Chr2:192273889-192273911 | 192273893 | 2 | RNA | GGCCGAGGTCGACTACCGGCNRG | GG--GAGcTacACTggCGGCGGG | - | 5 | 2 | 0 | 6.06 | 3.03 |
| Chr2:31312277-31312299 | 31312295 | 2 | RNA | GGCCGAGGTCGACTACCGGCNRG | GGC--tcGTgGACTACCGaCTGG | + | 4 | 2 | 0 | 6.06 | 3.03 |
| Chr10:21375920-21375942 | 21375924 | 2 | RNA | GGCCGAGGTCGACTACCGGCNRG | GagtGAGGTCtACTACC--aGAG | - | 5 | 2 | 0 | 6.06 | 3.03 |
| Chr6:77225701-77225723 | 77225716 | 5 | DNA | GGCCGAGGTCGACTACCGG--CNRG | cGCCGAGGcCGAaagCCGGGCCGAG | + | 5 | 2 | 0 | 6.06 | 3.03 |
| Chr8:59936404-59936426 | 59936421 | 3 | DNA | GGCCGAGGTCGACTA--CCGGCNRG | GGCgGAGcTtatCTACTCCGGCGAG | + | 5 | 2 | 0 | 6.06 | 3.03 |
| Chr2:84193865-84193887 | 84193881 | 4 | RNA | GGCCGAGGTCGACTACCGGCNRG | tGCCcAGGTC-ACgACacGCAGG | + | 5 | 1 | 0 | 6.06 | 3.03 |
| Chr4:151080712-151080734 | 151080727 | 5 | RNA | GGCCGAGGTCGACTACCGGCNRG | tGCgcAGGTCGACgtC--GCGGG | + | 5 | 2 | 0 | 6.06 | 3.03 |
| Chr2:20305841-20305863 | 20305847 | 4 | RNA | GGCCGAGGTCGACTACCGGCNRG | GGCtcAGG--GgCTACtGtCGGG | - | 5 | 2 | 0 | 6.06 | 3.03 |
| Chr2:151877121-151877143 | 151877138 | 3 | DNA | G--GCCGAGGTCGACTACCGGCNRG | GATGaCGgGGTCGACcACgaGCGGG | + | 5 | 2 | 0 | 6.06 | 3.03 |
| Chr4:33372328-33372350 | 33372344 | 4 | RNA | GGCCGAGGTCGACTACCGGCNRG | aGaCGAGGgCGAC--CCtGgCAG | + | 5 | 2 | 0 | 6.06 | 3.03 |
| Chr8:100193135-100193157 | 100193140 | 3 | RNA | GGCCGAGGTCGACTACCGGCNRG | G-aCGgGGTCGACgACgaGCGGG | - | 5 | 1 | 0 | 6.06 | 3.03 |
| Chr4:161446845-161446867 | 161446861 | 4 | RNA | GGCCGAGGTCGACTACCGGCNRG | tG--GAGGaaGACgACCtGCAAG | + | 5 | 2 | 0 | 6.06 | 3.03 |
| Chr4:181076698-181076720 | 181076704 | 4 | RNA | GGCCGAGGTCGACTACCGGCNRG | aGaCGAcGaCGACTAC--GaTAG | - | 5 | 2 | 0 | 6.06 | 3.03 |
| Chr1:170076236-170076258 | 170076254 | 2 | DNA | GGC-CGAGGTCGACTACCGGCNRG | GcCTCGgGGaCGtCTtCCGGCTGG | + | 5 | 1 | 0 | 6.06 | 3.03 |
| Chr5:166179889-166179911 | 166179894 | 3 | RNA | GGCCGAGGTCGACTACCGGCNRG | GGC--AGGcCaACctCCGGCCGG | - | 4 | 2 | 0 | 6.06 | 3.03 |
| Chr3:68259948-68259970 | 68259964 | 4 | RNA | GGCCGAGGTCGACTACCGGCNRG | G-aCGgGGTCGACgACaaGCGGG | + | 5 | 1 | 0 | 6.06 | 3.03 |
| Chr5:20291371-20291393 | 20291387 | 4 | RNA | GGCCGAGGTCGACTACCGGCNRG | GGgCGAGGgCtA--ACCGatCGG | + | 5 | 2 | 0 | 6.06 | 3.03 |
| Chr1:52972018-52972040 | 52972034 | 4 | RNA | GGCCGAGGTCGACTACCGGCNRG | GtCCG--GTgcACcACCGGaCAG | + | 5 | 2 | 0 | 6.06 | 3.03 |
| Chr4:234537134-234537156 | 234537150 | 4 | DNA | GGCCGAGGTCGACTACC--GGCNRG | GGCaGAGGTCGtCggCaGAGGCCAG | + | 5 | 2 | 0 | 6.06 | 3.03 |
| Chr3:106063514-106063536 | 106063521 | 5 | RNA | GGCCGAGGTCGACTACCGGCNRG | GtCCG--GTgcACcACCGGaCAG | - | 5 | 2 | 0 | 6.06 | 3.03 |
| Chr1:117362439-117362461 | 117362443 | 2 | RNA | GGCCGAGGTCGACTACCGGCNRG | a--CGAGcTCGACcACCaGaGAG | - | 5 | 2 | 0 | 6.06 | 3.03 |
| Chr7:95664010-95664032 | 95664028 | 2 | RNA | GGCCGAGGTCGACTACCGGCNRG | GGtCGtGGaCGAgT--CGGCGGG | + | 4 | 2 | 0 | 6.06 | 3.03 |
| Chr8:78627447-78627469 | 78627462 | 5 | RNA | GGCCGAGGTCGACTACCGGCNRG | G--CaAGGcCGAgctCCGGCAAG | + | 5 | 2 | 0 | 6.06 | 3.03 |
| Chr1:271077305-271077327 | 271077309 | 2 | RNA | GGCCGAGGTCGACTACCGGCNRG | GGCgGAGGTCG--TtCgGcaGAG | - | 5 | 2 | 0 | 6.06 | 3.03 |
| Chr8:163063070-163063092 | 163063086 | 4 | RNA | GGCCGAGGTCGACTACCGGCNRG | GGCCGAGG-CtACTcttGGgTGG | + | 5 | 1 | 0 | 6.06 | 3.03 |
| Chr1:230928743-230928765 | 230928759 | 4 | RNA | GGCCGAGGTCGACTACCGGCNRG | caCCaAGtTCGcCT-CCGGCCAG | + | 5 | 1 | 0 | 6.06 | 3.03 |
| Chr1:162962298-162962320 | 162962303 | 3 | RNA | GGCCGAGGTCGACTACCGGCNRG | GGCCtAtcTgGACTA--GGtTGG | - | 5 | 2 | 0 | 6.06 | 3.03 |
| Chr8:130470592-130470614 | 130470608 | 4 | RNA | GGCCGAGGTCGACTACCGGCNRG | G-aCGgGGTCGACaACgaGCGGG | + | 5 | 1 | 0 | 6.06 | 3.03 |
| Chr5:141155973-141155995 | 141155988 | 5 | RNA | GGCCGAGGTCGACTACCGGCNRG | GaCCGAGtTCagCcACCG-CCAG | + | 5 | 1 | 0 | 6.06 | 3.03 |
| Chr4:155949350-155949372 | 155949354 | 2 | RNA | GGCCGAGGTCGACTACCGGCNRG | GGCCaAtGTCGACaA--tGCAAG | - | 4 | 2 | 0 | 6.06 | 3.03 |
| Chr5:15533161-15533183 | 15533179 | 2 | RNA | GGCCGAGGTCGACTACCGGCNRG | GGt-GAGGTCGAgTttCGGgTGG | + | 5 | 1 | 0 | 6.06 | 3.03 |
| Chr1:268746054-268746076 | 268746072 | 2 | RNA | GGCCGAGGTCGACTACCGGCNRG | GtCCG--GTgcACcACCGGaCAG | + | 5 | 2 | 0 | 6.06 | 3.03 |
| Chr5:106271711-106271733 | 106271717 | 4 | RNA | GGCCGAGGTCGACTACCGGCNRG | tGaaGAGGc--ACTACCtGCGGG | - | 5 | 2 | 0 | 6.06 | 3.03 |
| Chr3:234332595-234332617 | 234332599 | 2 | RNA | GGCCGAGGTCGACTACCGGCNRG | GGCCG-GGcCGtCcACtaGCGGG | - | 5 | 1 | 0 | 6.06 | 3.03 |
| Chr4:49010447-49010469 | 49010463 | 4 | RNA | GGCCGAGGTCGACTACCGGCNRG | G--CGAGGTCGcCcgtCtGCGAG | + | 5 | 2 | 0 | 6.06 | 3.03 |
| Chr10:29619551-29619573 | 29619566 | 5 | RNA | GGCCGAGGTCGACTACCGGCNRG | GG--GcaGTCGtggACCGGCGAG | + | 5 | 2 | 0 | 6.06 | 3.03 |
| Chr3:211952508-211952530 | 211952524 | 4 | RNA | GGCCGAGGTCGACTACCGGCNRG | G-aCGgGGTCGACgACgaGCGGG | + | 5 | 1 | 0 | 6.06 | 3.03 |
| Chr8:39542492-39542514 | 39542496 | 2 | RNA | GGCCGAGGTCGACTACCGGCNRG | GtCgcAGGTCGA-gACCGaCCGG | - | 5 | 1 | 0 | 6.06 | 3.03 |
| Chr10:6663614-6663636 | 6663631 | 3 | DNA | GGC-CGAGGTCGACTACCGGCNRG | GcCTCGgGGaCGtCTtCCGGCTGG | + | 5 | 1 | 0 | 6.06 | 3.03 |
| Chr6:81702532-81702554 | 81702536 | 2 | RNA | GGCCGAGGTCGACTACCGGCNRG | GtCCGAa--aGAgTACCGGaAAG | - | 5 | 2 | 0 | 6.06 | 3.03 |
| Chr4:40538516-40538538 | 40538532 | 4 | DNA | GGCCGAGGTCGACTA--CCGGCNRG | GcCCGAcaTCGcCTACGCCGtCCAG | + | 5 | 2 | 0 | 6.06 | 3.03 |
| Chr6:83379081-83379103 | 83379088 | 5 | RNA | GGCCGAGGTCGACTACCGGCNRG | G-aCtAGGcgGACcACCGGCGGG | - | 5 | 1 | 6 | 0.00 | 3.00 |
| Chr9:143900795-143900817 | 143900812 | 3 | RNA | GGCCGAGGTCGACTACCGGCNRG | GcCCGAGGcCGA--ACttGgTGG | + | 5 | 2 | 6 | 0.00 | 3.00 |
| Chr2:62452759-62452781 | 62452776 | 3 | RNA | GGCCGAGGTCGACTACCGGCNRG | GaatGAGaTCGACTACt--CCAG | + | 5 | 2 | 6 | 0.00 | 3.00 |
| Chr1:239824175-239824197 | 239824192 | 3 | RNA | GGCCGAGGTCGACTACCGGCNRG | GcCCGAGGcCGA--ACtcGgGGG | + | 5 | 2 | 6 | 0.00 | 3.00 |
| Chr2:183812566-183812588 | 183812573 | 5 | DNA | GGCCGAGGTCGA-CTACCGGCNRG | GcCCGAGGgaGAGCgACCGaCCGG | - | 5 | 1 | 6 | 0.00 | 3.00 |
| Chr4:44956370-44956392 | 44956387 | 3 | RNA | GGCCGAGGTCGACTACCGGCNRG | tGC--AGGTgcACggCCGGCAGG | + | 5 | 2 | 6 | 0.00 | 3.00 |
| Chr4:201718685-201718707 | 201718702 | 3 | RNA | GGCCGAGGTCGACTACCGGCNRG | GctCGAGGTCG-CcgCCGGtGAG | + | 5 | 1 | 6 | 0.00 | 3.00 |
| Chr3:13342931-13342953 | 13342948 | 3 | RNA | GGCCGAGGTCGACTACCGGCNRG | GGC--AGGcCGAgcACCcGCCGG | + | 4 | 2 | 6 | 0.00 | 3.00 |
| Chr1:267072992-267073014 | 267072999 | 5 | DNA | GGC--CGAGGTCGACTACCGGCNRG | GGCCTCGgGGgCtACacCCGGCGGG | - | 5 | 2 | 6 | 0.00 | 3.00 |
| Chr2:117524235-117524257 | 117524252 | 3 | RNA | GGCCGAGGTCGACTACCGGCNRG | cGC--AcaaCGACTACCGcCCGG | + | 5 | 2 | 6 | 0.00 | 3.00 |
| Chr5:138036569-138036591 | 138036586 | 3 | RNA | GGCCGAGGTCGACTACCGGCNRG | GGC--AGGcCaACcACCGGCCGG | + | 3 | 2 | 6 | 0.00 | 3.00 |
| Chr9:17768554-17768576 | 17768571 | 3 | RNA | GGCCGAGGTCGACTACCGGCNRG | GGC--AGGcCGggcACCGaCCGG | + | 5 | 2 | 6 | 0.00 | 3.00 |
| Chr2:20891575-20891597 | 20891582 | 5 | RNA | GGCCGAGGTCGACTACCGGCNRG | cGaCGAGcTta--TACCGGCCGG | - | 5 | 2 | 6 | 0.00 | 3.00 |
| Chr6:159625937-159625959 | 159625954 | 3 | DNA | G--GCCGAGGTCGACTACCGGCNRG | GAGGCCGAGGcCGAggtCgGGCGAG | + | 5 | 2 | 6 | 0.00 | 3.00 |
| Chr5:24489734-24489756 | 24489751 | 3 | RNA | GGCCGAGGTCGACTACCGGCNRG | GtCCGAGGaC--CTAaCtGCGAG | + | 4 | 2 | 6 | 0.00 | 3.00 |
| Chr4:927905-927927 | 927922 | 3 | RNA | GGCCGAGGTCGACTACCGGCNRG | GGCCGcGG-CGACcAaCtGaAAG | + | 5 | 1 | 6 | 0.00 | 3.00 |
| Chr5:18605364-18605386 | 18605381 | 3 | RNA | GGCCGAGGTCGACTACCGGCNRG | GGCCGAGtaCG--TtgCcGCAGG | + | 5 | 2 | 6 | 0.00 | 3.00 |
| Chr6:100600754-100600776 | 100600761 | 5 | RNA | GGCCGAGGTCGACTACCGGCNRG | GaCCGAGGTCctCgAC--GaCGG | - | 5 | 2 | 6 | 0.00 | 3.00 |
| Chr3:179281155-179281177 | 179281162 | 5 | RNA | GGCCGAGGTCGACTACCGGCNRG | GGCCGAGGTgGcCTtC--GCGGG | - | 3 | 2 | 6 | 0.00 | 3.00 |
| Chr1:89413185-89413207 | 89413192 | 5 | RNA | GGCCGAGGTCGACTACCGGCNRG | aGCCGAGGgC-ACctCgGGCAGG | - | 5 | 1 | 6 | 0.00 | 3.00 |
| Chr8:37497992-37498014 | 37498009 | 3 | RNA | GGCCGAGGTCGACTACCGGCNRG | GGgCGAGGgCGA-gAatGGCAGG | + | 5 | 1 | 6 | 0.00 | 3.00 |
| Chr8:83380376-83380398 | 83380393 | 3 | RNA | GGCCGAGGTCGACTACCGGCNRG | GGCCG-GGTCGAgaAgCatCAAG | + | 5 | 1 | 6 | 0.00 | 3.00 |
| Chr4:85007047-85007069 | 85007054 | 5 | DNA | GGCCGAGGTCG--ACTACCGGCNRG | GcCCGAGGTCGCTtgTACCctCGGG | - | 5 | 2 | 6 | 0.00 | 3.00 |
| Chr4:91487901-91487923 | 91487918 | 3 | RNA | GGCCGAGGTCGACTACCGGCNRG | GGCCGAGcTtGcCTtC-GcCCAG | + | 5 | 1 | 6 | 0.00 | 3.00 |
| Chr8:134314717-134314739 | 134314724 | 5 | DNA | GGCCGAGGTCGACTACCG-GCNRG | GGCCGAGGTCGcCTcagGCtCGGG | - | 5 | 1 | 6 | 0.00 | 3.00 |
| Chr1:17752534-17752556 | 17752551 | 3 | DNA | GGCCGAGGTCGACTAC-CGGCNRG | GaCCaAGGTCaACTgCTCGGaTGG | + | 5 | 1 | 6 | 0.00 | 3.00 |
| Chr1:248317774-248317796 | 248317781 | 5 | RNA | GGCCGAGGTCGACTACCGGCNRG | GGttGAG--CcACTAaCGtCAGG | - | 5 | 2 | 6 | 0.00 | 3.00 |
| Chr2:33213866-33213888 | 33213883 | 3 | RNA | GGCCGAGGTCGACTACCGGCNRG | GGCgcAaGTCGAgg--CGGCCAG | + | 5 | 2 | 6 | 0.00 | 3.00 |
| Chr8:75568215-75568237 | 75568232 | 3 | RNA | GGCCGAGGTCGACTACCGGCNRG | GagCGAG-TCGtCaACCGtCCAG | + | 5 | 1 | 6 | 0.00 | 3.00 |
| Chr8:58903585-58903607 | 58903592 | 5 | X | GGCCGAGGTCGACTACCGGCNRG | GGCCGAcGgCGACcAaCaGCCAG | - | 5 | 0 | 6 | 0.00 | 3.00 |
| Chr2:147395006-147395028 | 147395023 | 3 | DNA | GGCCGAGGTC-GACTACCGGCNRG | GGCCGAGGcCTGAggtCgGGCGAG | + | 5 | 1 | 6 | 0.00 | 3.00 |
| Chr2:104049539-104049561 | 104049546 | 5 | RNA | GGCCGAGGTCGACTACCGGCNRG | tttCGAGGgCGA--ACCGGgCAG | - | 5 | 2 | 6 | 0.00 | 3.00 |
| Chr8:120988300-120988322 | 120988317 | 3 | RNA | GGCCGAGGTCGACTACCGGCNRG | GGtCGAGa-CGtCcACCaGCCGG | + | 5 | 1 | 6 | 0.00 | 3.00 |
| Chr3:54316439-54316461 | 54316456 | 3 | DNA | GGCC-GAGGTCGACTACCGGCNRG | tGCCTGAGGTCatCcACaGGCGGG | + | 5 | 1 | 6 | 0.00 | 3.00 |
| Chr3:3896525-3896547 | 3896532 | 5 | RNA | GGCCGAGGTCGACTACCGGCNRG | aGCCGAGGgtGcCT-CgGGCAGG | - | 5 | 1 | 6 | 0.00 | 3.00 |
| Chr2:20807348-20807370 | 20807365 | 3 | RNA | GGCCGAGGTCGACTACCGGCNRG | G-CCGgGGTCGgCTcCgGGtCAG | + | 5 | 1 | 6 | 0.00 | 3.00 |
| Chr1:214809013-214809035 | 214809030 | 3 | RNA | GGCCGAGGTCGACTACCGGCNRG | GGCCGcG--CGtgcACgGGCCAG | + | 5 | 2 | 6 | 0.00 | 3.00 |
| Chr8:34046458-34046480 | 34046475 | 3 | RNA | GGCCGAGGTCGACTACCGGCNRG | GGCCGgGaT--AtcAtCGGCAGG | + | 5 | 2 | 6 | 0.00 | 3.00 |
| Chr5:165138320-165138342 | 165138327 | 5 | RNA | GGCCGAGGTCGACTACCGGCNRG | tGCgcAGGTCGACatCC--CTGG | - | 5 | 2 | 6 | 0.00 | 3.00 |
| Chr8:88896833-88896855 | 88896840 | 5 | RNA | GGCCGAGGTCGACTACCGGCNRG | G-aCGgGGTCGACaACgaGCGGG | - | 5 | 1 | 6 | 0.00 | 3.00 |
| Chr7:65079196-65079218 | 65079213 | 3 | RNA | GGCCGAGGTCGACTACCGGCNRG | cGgCGgGGaCGA-TAgCGGCAAG | + | 5 | 1 | 6 | 0.00 | 3.00 |
| Chr3:61761079-61761101 | 61761096 | 3 | RNA | GGCCGAGGTCGACTACCGGCNRG | G--CGAGGTCGAtgAatGGgTAG | + | 5 | 2 | 6 | 0.00 | 3.00 |
| Chr2:187105452-187105474 | 187105459 | 5 | DNA | GGCCGAGGTCGACTACCGG-CNRG | GaCCGAGGTCGAggcCaGGTCCGG | - | 5 | 1 | 6 | 0.00 | 3.00 |
| Chr1:45735557-45735579 | 45735574 | 3 | RNA | GGCCGAGGTCGACTACCGGCNRG | GGC--tcGTgGACTACCGGCTGG | + | 3 | 2 | 6 | 0.00 | 3.00 |
| Chr2:8554078-8554100 | 8554095 | 3 | RNA | GGCCGAGGTCGACTACCGGCNRG | GG--GAGtTCGgCTAggGGCAAG | + | 4 | 2 | 6 | 0.00 | 3.00 |
| Chr4:57838553-57838575 | 57838560 | 5 | RNA | GGCCGAGGTCGACTACCGGCNRG | GGCCGcG--tGACTACCccaAGG | - | 5 | 2 | 6 | 0.00 | 3.00 |
| Chr10:76813792-76813814 | 76813809 | 3 | DNA | GGCCGAGGTCGACT-ACCGGCNRG | cGCCGcGGatGACTCACCGGaCAG | + | 5 | 1 | 6 | 0.00 | 3.00 |
| Chr9:21642208-21642230 | 21642215 | 5 | RNA | GGCCGAGGTCGACTACCGGCNRG | GG--GAGGgCGcgggCCGGCCGG | - | 5 | 2 | 6 | 0.00 | 3.00 |
| Chr2:219367667-219367689 | 219367684 | 3 | RNA | GGCCGAGGTCGACTACCGGCNRG | GGC--AGGcCGAgcACCaGCCGG | + | 4 | 2 | 6 | 0.00 | 3.00 |
| Chr2:27010006-27010028 | 27010023 | 3 | RNA | GGCCGAGGTCGACTACCGGCNRG | GcCCGAG-TCGgCTcgCGGtAGG | + | 5 | 1 | 6 | 0.00 | 3.00 |
| Chr4:14797194-14797216 | 14797201 | 5 | RNA | GGCCGAGGTCGACTACCGGCNRG | GtCCG--GaaGAgTACCGGaAAG | - | 5 | 2 | 6 | 0.00 | 3.00 |
| Chr1:142633277-142633299 | 142633284 | 5 | RNA | GGCCGAGGTCGACTACCGGCNRG | GGCCGcGGaCagCgAC--GCGGG | - | 5 | 2 | 6 | 0.00 | 3.00 |
| Chr5:78069463-78069485 | 78069480 | 3 | DNA | GGCCG--AGGTCGACTACCGGCNRG | GGCCGCAAGGTCagtgACCaGCGAG | + | 5 | 2 | 6 | 0.00 | 3.00 |

| | | | | | | | | | | | |
|---|---|---|---|---|---|---|---|---|---|---|---|
| Chr1:67539943-67539965 | 67539950 | 5 | RNA | GGCCGAGGTCGACTACCGGCNRG | GcCCGAGccCGAgT--CGGtAGG | - | 5 | 2 | 6 | 0.00 | 3.00 |
| Chr5:216890104-216890126 | 216890121 | 3 | RNA | GGCCGAGGTCGACTACCGGCNRG | GG--GAaGTCGACTAtCtcCAAG | + | 4 | 2 | 6 | 0.00 | 3.00 |
| Chr4:65705658-65705680 | 65705675 | 3 | RNA | GGCCGAGGTCGACTACCGGCNRG | GGCgGAGGT-GctTACCaGgAGG | + | 5 | 1 | 6 | 0.00 | 3.00 |
| Chr8:95257752-95257774 | 95257769 | 3 | RNA | GGCCGAGGTCGACTACCGGCNRG | GaCCGAGG-CGACgACaaGaAGG | + | 5 | 1 | 6 | 0.00 | 3.00 |
| Chr6:159611040-159611062 | 159611057 | 3 | RNA | GGCCGAGGTCGACTACCGGCNRG | cGtCGAGGTC--CTcCCaGgTAG | + | 5 | 2 | 6 | 0.00 | 3.00 |
| Chr7:106612436-106612458 | 106612443 | 5 | RNA | GGCCGAGGTCGACTACCGGCNRG | GGC-GtGGTCGAggACtGGCGGG | - | 4 | 1 | 6 | 0.00 | 3.00 |
| Chr2:131651307-131651329 | 131651314 | 5 | RNA | GGCCGAGGTCGACTACCGGCNRG | GGgCGAG--CGctgACaGGCGGG | - | 5 | 2 | 6 | 0.00 | 3.00 |
| Chr2:63726311-63726333 | 63726318 | 5 | RNA | GGCCGAGGTCGACTACCGGCNRG | aGCCtAtGTCGACTACC--tCGG | - | 4 | 2 | 6 | 0.00 | 3.00 |
| Chr3:132856517-132856539 | 132856534 | 3 | DNA | GGC--CGAGGTCGACTACCGGCNRG | GGCGTCGAGGTCaACggCCGGgCGG | + | 4 | 2 | 6 | 0.00 | 3.00 |
| Chr5:211208289-211208311 | 211208296 | 5 | RNA | GGCCGAGGTCGACTACCGGCNRG | GcCCGAcGTC--CTcCCaGgTGG | - | 5 | 2 | 6 | 0.00 | 3.00 |
| Chr5:99721071-99721093 | 99721078 | 5 | RNA | GGCCGAGGTCGACTACCGGCNRG | GGgCGgGG-CGACcACacGCGGG | - | 5 | 1 | 6 | 0.00 | 3.00 |
| Chr2:191038543-191038565 | 191038550 | 5 | RNA | GGCCGAGGTCGACTACCGGCNRG | cGCCGccGTCGAC--CtGGtTAG | - | 5 | 2 | 6 | 0.00 | 3.00 |
| Chr9:20794407-20794429 | 20794414 | 5 | RNA | GGCCGAGGTCGACTACCGGCNRG | G-CCtcGGgCGAgTACtGGCAGG | - | 5 | 1 | 6 | 0.00 | 3.00 |
| Chr3:12643969-12643991 | 12643976 | 5 | DNA | GGCCGAGGTCGACTACCG-GCNRG | GGaCGgaGTCGtCTACCGGGgTGG | - | 5 | 1 | 6 | 0.00 | 3.00 |
| Chr1:146155367-146155389 | 146155384 | 3 | RNA | GGCCGAGGTCGACTACCGGCNRG | G-CCtcGGgCGAgTACtGGCAGG | + | 5 | 1 | 6 | 0.00 | 3.00 |
| Chr2:72470284-72470306 | 72470301 | 3 | DNA | GGCCGA--GGTCGACTACCGGCNRG | GGgtGACCGaaCGACgACCGGCAAG | + | 5 | 2 | 6 | 0.00 | 3.00 |
| Chr1:190346228-190346250 | 190346245 | 3 | RNA | GGCCGAGGTCGACTACCGGCNRG | GGaCcAGGTCG--TcCCaGtTGG | + | 5 | 2 | 6 | 0.00 | 3.00 |
| Chr3:70899041-70899063 | 70899048 | 5 | RNA | GGCCGAGGTCGACTACCGGCNRG | GaCCGAGGaCttCTACCG-CGGG | - | 4 | 1 | 6 | 0.00 | 3.00 |
| Chr9:77411136-77411158 | 77411143 | 5 | RNA | GGCCGAGGTCGACTACCGGCNRG | G-CCGAGGTtGgCctCgGGCAAG | - | 5 | 1 | 6 | 0.00 | 3.00 |
| Chr1:108825688-108825710 | 108825705 | 3 | RNA | GGCCGAGGTCGACTACCGGCNRG | G-CCGAaGTacACcACCtGCTGG | + | 5 | 1 | 6 | 0.00 | 3.00 |
| Chr2:225361920-225361942 | 225361927 | 5 | RNA | GGCCGAGGTCGACTACCGGCNRG | actgGcGGTCGACT-CCGGCCGG | - | 5 | 1 | 6 | 0.00 | 3.00 |
| Chr5:71895882-71895904 | 71895889 | 5 | RNA | GGCCGAGGTCGACTACCGGCNRG | GGCCGAaGTCGgCaACC--tGG | - | 4 | 2 | 6 | 0.00 | 3.00 |
| Chr10:93666644-93666666 | 93666651 | 5 | RNA | GGCCGAGGTCGACTACCGGCNRG | GctCGAaGcC--CTAaCGGCAGG | - | 5 | 2 | 6 | 0.00 | 3.00 |
| Chr3:34385661-34385683 | 34385678 | 3 | RNA | GGCCGAGGTCGACTACCGGCNRG | G--CGAGcTCatCggCCGGCCGG | + | 5 | 2 | 6 | 0.00 | 3.00 |
| Chr6:113142918-113142940 | 113142935 | 3 | RNA | GGCCGAGGTCGACTACCGGCNRG | atCCtgGGT-GACcACCGGCGGG | + | 5 | 1 | 6 | 0.00 | 3.00 |
| Chr4:141664652-141664674 | 141664669 | 3 | RNA | GGCCGAGGTCGACTACCGGCNRG | G-aCGgGGTCGACaACgaGCGGG | + | 5 | 1 | 6 | 0.00 | 3.00 |
| Chr1:132746271-132746293 | 132746288 | 3 | RNA | GGCCGAGGTCGACTACCGGCNRG | GGaCGtcGTCGACcgCCG-CGGG | + | 5 | 1 | 6 | 0.00 | 3.00 |
| Chr3:59560648-59560670 | 59560665 | 3 | RNA | GGCCGAGGTCGACTACCGGCNRG | GGaCGgtcTCGACT-CCGGtTGG | + | 5 | 1 | 6 | 0.00 | 3.00 |
| Chr9:118995348-118995370 | 118995365 | 3 | DNA | GGCCGAGGTCGACTAC-CGGCNRG | GGCCGAGGctGACacCGaGGCCGG | + | 5 | 1 | 6 | 0.00 | 3.00 |
| Chr1:35071769-35071791 | 35071776 | 5 | X | GGCCGAGGTCGACTACCGGCNRG | GGCgGtGGTCGgCgAgCGGCGGG | - | 5 | 0 | 6 | 0.00 | 3.00 |
| Chr10:99023149-99023171 | 99023156 | 5 | RNA | GGCCGAGGTCGACTACCGGCNRG | GGC--tcGcaGACTACCGGCTGG | - | 4 | 2 | 6 | 0.00 | 3.00 |
| Chr6:43494395-43494417 | 43494402 | 5 | DNA | GGCCGAGGTCGACT-ACCGGCNRG | GGgCGcGGctGACTCACCGGaTAG | - | 5 | 1 | 6 | 0.00 | 3.00 |
| Chr5:185200067-185200089 | 185200084 | 3 | RNA | GGCCGAGGTCGACTACCGGCNRG | cGCgcAcGTCGACTgCC--CCGG | + | 5 | 2 | 6 | 0.00 | 3.00 |
| Chr10:142691979-142692001 | 142691986 | 5 | DNA | GGC--CGAGGTCGACTACCGGCNRG | GGCCTCGgGGgCtACacCCGGCGGG | - | 5 | 2 | 6 | 0.00 | 3.00 |
| Chr4:225409632-225409654 | 225409649 | 3 | RNA | GGCCGAGGTCGACTACCGGCNRG | GGCCGAGtcCG-CgACCaGaTAG | + | 5 | 1 | 6 | 0.00 | 3.00 |
| Chr1:123925346-123925368 | 123925353 | 5 | RNA | GGCCGAGGTCGACTACCGGCNRG | GGCCttGtTCGctTA--GGCGGG | - | 5 | 2 | 6 | 0.00 | 3.00 |
| Chr2:124766213-124766235 | 124766230 | 3 | DNA | GGC--CGAGGTCGACTACCGGCNRG | GGCGGCGtGGTCGACggCgaGCAAG | + | 5 | 2 | 6 | 0.00 | 3.00 |
| Chr5:178180723-178180745 | 178180740 | 3 | RNA | GGCCGAGGTCGACTACCGGCNRG | t--CGgGGaCGtCTtCCGGCTGG | + | 5 | 2 | 6 | 0.00 | 3.00 |
| Chr8:106889847-106889869 | 106889864 | 3 | RNA | GGCCGAGGTCGACTACCGGCNRG | G-aCGgGGTCGACaACgaGCGGG | + | 5 | 1 | 6 | 0.00 | 3.00 |
| Chr7:46237825-46237847 | 46237842 | 3 | RNA | GGCCGAGGTCGACTACCGGCNRG | GG--GAGtTCcACTcaaGGCAGG | + | 5 | 2 | 6 | 0.00 | 3.00 |
| Chr2:157494426-157494448 | 157494433 | 5 | RNA | GGCCGAGGTCGACTACCGGCNRG | GGgCGAtGaCGACgAC--GgAGG | - | 5 | 2 | 6 | 0.00 | 3.00 |
| Chr9:14443523-14443545 | 14443540 | 3 | DNA | GGCCGAG--GTCGACTACCGGCNRG | GttCGAGATGTCGAtTAtgGGCGAG | + | 5 | 2 | 6 | 0.00 | 3.00 |
| Chr8:94676777-94676799 | 94676784 | 5 | RNA | GGCCGAGGTCGACTACCGGCNRG | GGC--tcGagGACTACCGGCTGG | - | 4 | 2 | 6 | 0.00 | 3.00 |
| Chr2:118140559-118140581 | 118140566 | 5 | RNA | GGCCGAGGTCGACTACCGGCNRG | cGCCaAaGaCGACTgC--GCGGG | - | 5 | 2 | 6 | 0.00 | 3.00 |
| Chr9:81776195-81776217 | 81776212 | 3 | RNA | GGCCGAGGTCGACTACCGGCNRG | GGgCG-GGcCaACccCCGGCCGG | + | 5 | 1 | 6 | 0.00 | 3.00 |
| Chr4:155102967-155102989 | 155102984 | 3 | RNA | GGCCGAGGTCGACTACCGGCNRG | ttCCGAtGTCGAtTg--GGCAGG | + | 5 | 2 | 6 | 0.00 | 3.00 |
| Chr2:40746979-40747001 | 40746986 | 5 | RNA | GGCCGAGGTCGACTACCGGCNRG | GcCCGAGGcCGA--ACtcGgTGG | - | 5 | 2 | 6 | 0.00 | 3.00 |
| Chr5:38850092-38850114 | 38850099 | 5 | RNA | GGCCGAGGTCGACTACCGGCNRG | tG--GAGGTtGcCaACCGGaTAG | - | 5 | 2 | 6 | 0.00 | 3.00 |
| Chr2:199881248-199881270 | 199881255 | 5 | RNA | GGCCGAGGTCGACTACCGGCNRG | t--CGgGGaCGtCTtCCGGCTGG | - | 5 | 2 | 6 | 0.00 | 3.00 |
| Chr9:52723075-52723097 | 52723092 | 3 | RNA | GGCCGAGGTCGACTACCGGCNRG | GGCaGAG--CGAtTAgaaGCTAG | + | 5 | 2 | 6 | 0.00 | 3.00 |
| Chr6:135676726-135676748 | 135676743 | 3 | RNA | GGCCGAGGTCGACTACCGGCNRG | aaCCGAGcTCG--gAaCGGCGGG | + | 5 | 2 | 6 | 0.00 | 3.00 |
| Chr2:149630337-149630359 | 149630354 | 3 | RNA | GGCCGAGGTCGACTACCGGCNRG | GGgC-AGGcCaACctCCGGCCGG | + | 5 | 1 | 6 | 0.00 | 3.00 |
| Chr8:120335079-120335101 | 120335086 | 5 | RNA | GGCCGAGGTCGACTACCGGCNRG | GGC--ttGcgGACTACCGGCTGG | - | 4 | 2 | 6 | 0.00 | 3.00 |
| Chr5:88785285-88785307 | 88785302 | 3 | RNA | GGCCGAGGTCGACTACCGGCNRG | GGaCcAGtTCGACTA--aGtCAG | + | 5 | 2 | 6 | 0.00 | 3.00 |
| Chr5:211083589-211083611 | 211083606 | 3 | RNA | GGCCGAGGTCGACTACCGGCNRG | GcCgGAGccCGAC--CCGtCAAG | + | 5 | 2 | 6 | 0.00 | 3.00 |
| Chr8:60853116-60853138 | 60853133 | 3 | RNA | GGCCGAGGTCGACTACCGGCNRG | GGC--tcGcgGACTAtCGGCTGG | + | 5 | 2 | 6 | 0.00 | 3.00 |
| Chr4:38855777-38855799 | 38855794 | 3 | RNA | GGCCGAGGTCGACTACCGGCNRG | GGC--AGGcCGAgcACCGGCCGG | + | 3 | 2 | 6 | 0.00 | 3.00 |
| Chr2:103752146-103752168 | 103752163 | 3 | DNA | GGCCGAGGTCGACTACC--GGCNRG | GGCCGcGGTaGACTAaCCTcGaCGG | + | 5 | 2 | 6 | 0.00 | 3.00 |
| Chr4:22135726-22135748 | 22135743 | 3 | RNA | GGCCGAGGTCGACTACCGGCNRG | cGtCGAGtTCGcCT-CCGcCGGG | + | 5 | 1 | 6 | 0.00 | 3.00 |
| Chr1:281412146-281412168 | 281412153 | 5 | DNA | GGC--CGAGGTCGACTACCGGCNRG | GGCCTCGgGGgCtACacCCGGCGGG | - | 5 | 2 | 6 | 0.00 | 3.00 |
| Chr9:55197101-55197123 | 55197108 | 5 | RNA | GGCCGAGGTCGACTACCGGCNRG | GGC-GAccTCGgCTtCCtGCTGG | - | 5 | 1 | 6 | 0.00 | 3.00 |
| Chr2:119960514-119960536 | 119960531 | 3 | DNA | G-GCCGAGGTCGACTACCGGCNRG | GAGCtGAGcaCGgCcACCGGCGGG | + | 5 | 1 | 6 | 0.00 | 3.00 |
| Chr10:76266404-76266426 | 76266411 | 5 | RNA | GGCCGAGGTCGACTACCGGCNRG | GcCgGAGccCGAC--CCGtCAAG | - | 5 | 2 | 6 | 0.00 | 3.00 |
| Chr4:69757015-69757037 | 69757022 | 5 | RNA | GGCCGAGGTCGACTACCGGCNRG | G-aCGAGGTCGACaACgaGCGGG | - | 4 | 1 | 6 | 0.00 | 3.00 |
| Chr2:29891318-29891340 | 29891325 | 5 | RNA | GGCCGAGGTCGACTACCGGCNRG | GG--GAGcTCcACTACtGGgAAG | - | 4 | 2 | 6 | 0.00 | 3.00 |
| Chr8:38341080-38341102 | 38341087 | 5 | DNA | GGCCGAGGTCGACT-ACCGGCNRG | GGgCGtGGgCGACTGACaGGgGGG | - | 5 | 1 | 6 | 0.00 | 3.00 |
| Chr3:231393788-231393810 | 231393795 | 5 | RNA | GGCCGAGGTCGACTACCGGCNRG | G-tCGccGTCGtCgACCGGCGGG | - | 5 | 1 | 6 | 0.00 | 3.00 |
| Chr2:116690125-116690147 | 116690132 | 5 | RNA | GGCCGAGGTCGACTACCGGCNRG | GGC-atGGTCGAggACtGGCGGG | - | 5 | 1 | 6 | 0.00 | 3.00 |
| Chr2:5869429-5869451 | 5869436 | 5 | RNA | GGCCGAGGTCGACTACCGGCNRG | GGCCGAGGgCtA-gACatGCCGG | - | 5 | 1 | 6 | 0.00 | 3.00 |
| Chr6:97775764-97775786 | 97775781 | 3 | RNA | GGCCGAGGTCGACTACCGGCNRG | aGCCGAGcTC--CTtCCGcgGAG | + | 5 | 2 | 6 | 0.00 | 3.00 |
| Chr9:61102069-61102091 | 61102076 | 5 | RNA | GGCCGAGGTCGACTACCGGCNRG | tG--GAGGagGACgACCtGCTGG | - | 5 | 2 | 6 | 0.00 | 3.00 |
| Chr1:276604348-276604370 | 276604355 | 5 | RNA | GGCCGAGGTCGACTACCGGCNRG | GaagGAGGTC-AtgACCGGCAAG | - | 5 | 1 | 6 | 0.00 | 3.00 |
| Chr5:65822139-65822161 | 65822146 | 5 | RNA | GGCCGAGGTCGACTACCGGCNRG | GGC--tcGcgGACTACCGtCTGG | - | 5 | 2 | 6 | 0.00 | 3.00 |
| Chr10:50542966-50542988 | 50542983 | 3 | RNA | GGCCGAGGTCGACTACCGGCNRG | GGC--AGGcCGAgcACtaGCCGG | + | 5 | 2 | 6 | 0.00 | 3.00 |
| Chr7:1563558-1563580 | 1563565 | 5 | RNA | GGCCGAGGTCGACTACCGGCNRG | aGCCGAGGatGACcAC-GtCCAG | - | 5 | 1 | 6 | 0.00 | 3.00 |
| Chr5:218328664-218328686 | 218328671 | 5 | RNA | GGCCGAGGTCGACTACCGGCNRG | GaCC-AGGTCGtCTcCCaGtAGG | - | 5 | 1 | 6 | 0.00 | 3.00 |
| Chr3:210170747-210170769 | 210170764 | 3 | RNA | GGCCGAGGTCGACTACCGGCNRG | GGCCGtGcTtG--TcCCGGaGGG | + | 5 | 2 | 6 | 0.00 | 3.00 |
| Chr9:65079073-65079095 | 65079090 | 3 | RNA | GGCCGAGGTCGACTACCGGCNRG | GGC--AGGcCaACctCCGGCCGG | + | 4 | 2 | 6 | 0.00 | 3.00 |
| Chr6:72911352-72911374 | 72911368 | 4 | RNA | GGCCGAGGTCGACTACCGGCNRG | G-aCGgGGTCGACaACgaGCGGG | + | 5 | 1 | 1 | 4.96 | 2.98 |
| Chr3:62192763-62192785 | 62192779 | 4 | RNA | GGCCGAGGTCGACTACCGGCNRG | cGCCGAGtcCGAaTAtC--CTAG | + | 5 | 2 | 1 | 4.96 | 2.98 |
| Chr2:33514086-33514108 | 33514092 | 4 | RNA | GGCCGAGGTCGACTACCGGCNRG | GGC--AGGctaACctCCGGCCGG | - | 5 | 2 | 2 | 3.86 | 2.93 |
| Chr4:191426183-191426205 | 191426189 | 4 | RNA | GGCCGAGGTCGACTACCGGCNRG | caCCaAGtTCGcCT-CCGGCCAG | - | 5 | 1 | 2 | 3.86 | 2.93 |
| Chr5:82350877-82350899 | 82350882 | 3 | RNA | GGCCGAGGTCGACTACCGGCNRG | G-tCGAGGcCcACTACCGaCGGG | - | 4 | 1 | 2 | 3.86 | 2.93 |
| Chr1:73313951-73313973 | 73313957 | 4 | RNA | GGCCGAGGTCGACTACCGGCNRG | GGC-GAGGTCGACggCgaGaTGG | - | 5 | 1 | 3 | 2.75 | 2.88 |
| Chr10:60613212-60613234 | 60613219 | 5 | RNA | GGCCGAGGTCGACTACCGGCNRG | GGCgcAGcTCaACT-CCGGCGAG | - | 4 | 1 | 4 | 1.65 | 2.83 |
| Chr10:103260982-103261004 | 103261000 | 2 | RNA | GGCCGAGGTCGACTACCGGCNRG | GG--GAcGaCGACgAtCtGCTAG | + | 5 | 2 | 4 | 1.65 | 2.83 |
| Chr3:123060771-123060793 | 123060776 | 3 | RNA | GGCCGAGGTCGACTACCGGCNRG | atCCGAGaTCGAt--CgGGCGAG | - | 5 | 2 | 5 | 0.55 | 2.78 |
| Chr3:27939723-27939745 | 27939728 | 3 | DNA | GGCCGAGGTCGACTAC-CGGCNRG | cGaCGAGGTCGACcACGCGGtCGG | - | 4 | 1 | 0 | 5.51 | 2.76 |
| Chr4:32708440-32708462 | 32708458 | 2 | RNA | GGCCGAGGTCGACTACCGGCNRG | cGgCGAGGaCGA--ACaGGgGAG | + | 5 | 2 | 0 | 5.51 | 2.76 |
| Chr10:24810560-24810582 | 24810577 | 3 | RNA | GGCCGAGGTCGACTACCGGCNRG | GG--GcGGTCaACTgCtGaCAGG | + | 5 | 2 | 0 | 5.51 | 2.76 |
| Chr2:161671483-161671505 | 161671500 | 3 | RNA | GGCCGAGGTCGACTACCGGCNRG | GGCCGAGGTaGgCaAta--CTGG | + | 5 | 2 | 0 | 5.51 | 2.76 |
| Chr8:131056168-131056190 | 131056184 | 4 | RNA | GGCCGAGGTCGACTACCGGCNRG | GGC--tcGcgGACTACCGGCTGG | + | 4 | 2 | 0 | 5.51 | 2.76 |
| Chr5:39204058-39204080 | 39204074 | 4 | RNA | GGCCGAGGTCGACTACCGGCNRG | GGaCcAGGTCG--TcCCaGtTGG | + | 5 | 2 | 0 | 5.51 | 2.76 |
| Chr5:114002789-114002811 | 114002794 | 3 | DNA | GGC--CGAGGTCGACTACCGGCNRG | GaCAACGgGGTCGACgACgaGCGGG | - | 5 | 2 | 0 | 5.51 | 2.76 |
| Chr4:12783698-12783720 | 12783716 | 2 | DNA | GGCCGAGGTCGACT-ACCGGCNRG | GGgCGAGcgCGtCTGACaGGCGAG | + | 5 | 1 | 0 | 5.51 | 2.76 |
| Chr8:94701654-94701676 | 94701659 | 3 | RNA | GGCCGAGGTCGACTACCGGCNRG | GaCCGAGG-CGACgACaaGaAGG | - | 5 | 1 | 0 | 5.51 | 2.76 |
| Chr4:160175695-160175717 | 160175710 | 5 | RNA | GGCCGAGGTCGACTACCGGCNRG | GGC--tcGcaGACTACCGGCTGG | + | 4 | 2 | 0 | 5.51 | 2.76 |
| Chr9:51977210-51977232 | 51977228 | 2 | DNA | GGCCGAGGTCGACTACC--GGCNRG | GcCCGAGcaCGACcACCGTGGCCAG | + | 4 | 2 | 0 | 5.51 | 2.76 |
| Chr2:24626185-24626207 | 24626202 | 3 | RNA | GGCCGAGGTCGACTACCGGCNRG | GGC--tcGcgGACTACCGGCTGG | + | 4 | 2 | 0 | 5.51 | 2.76 |
| Chr8:136445037-136445059 | 136445055 | 2 | RNA | GGCCGAGGTCGACTACCGGCNRG | GGCCG--GTCtgCcAgCGGgAGG | + | 5 | 2 | 0 | 5.51 | 2.76 |
| Chr8:124912135-124912157 | 124912142 | 5 | DNA | GGCCGAGGTCGACTACCGG-CNRG | GGCCGAGGTCGgCactCGGCaAAG | - | 5 | 1 | 0 | 5.51 | 2.76 |
| Chr6:91836601-91836623 | 91836605 | 2 | RNA | GGCCGAGGTCGACTACCGGCNRG | GGC-GAGcTCcACcAtCtGCCGG | - | 5 | 1 | 0 | 5.51 | 2.76 |
| Chr8:143752936-143752958 | 143752952 | 4 | RNA | GGCCGAGGTCGACTACCGGCNRG | GG--GAGGctGtCTgCCGaCGGG | + | 5 | 2 | 0 | 5.51 | 2.76 |
| Chr5:20481950-20481972 | 20481966 | 4 | DNA | GGC-CGAGGTCGACTACCGGCNRG | GcCTCGgGGaCGtCTtCCGGCTGG | + | 5 | 1 | 0 | 5.51 | 2.76 |
| Chr1:70047759-70047781 | 70047764 | 3 | RNA | GGCCGAGGTCGACTACCGGCNRG | GGtCG--GTCGgCTACCcGaCGG | - | 4 | 2 | 0 | 5.51 | 2.76 |
| Chr3:44982986-44983008 | 44982992 | 4 | RNA | GGCCGAGGTCGACTACCGGCNRG | GaCCGgGGTCGcCcA--GaCGGG | - | 5 | 2 | 0 | 5.51 | 2.76 |
| Chr5:102052323-102052345 | 102052330 | 5 | RNA | GGCCGAGGTCGACTACCGGCNRG | cGaCGAtGgCGAC--CtGGCCAG | - | 5 | 2 | 0 | 5.51 | 2.76 |
| Chr9:72567266-72567288 | 72567273 | 5 | RNA | GGCCGAGGTCGACTACCGGCNRG | GGtCGgGGT--AactCCGGCGAG | - | 5 | 2 | 0 | 5.51 | 2.76 |
| Chr7:139865706-139865728 | 139865713 | 5 | RNA | GGCCGAGGTCGACTACCGGCNRG | GGCtGAacTCG-CTACCttCGGG | - | 5 | 1 | 0 | 5.51 | 2.76 |
| Chr2:136391856-136391878 | 136391873 | 3 | RNA | GGCCGAGGTCGACTACCGGCNRG | GGaCtAGGTCG--TcCCaGtTGG | + | 5 | 2 | 0 | 5.51 | 2.76 |
| Chr5:34201656-34201678 | 34201661 | 3 | RNA | GGCCGAGGTCGACTACCGGCNRG | GGtCGAGGTCG--TggCcGaGGG | - | 5 | 2 | 0 | 5.51 | 2.76 |
| Chr1:205218340-205218362 | 205218358 | 2 | RNA | GGCCGAGGTCGACTACCGGCNRG | GGt--AGGcCGAgcACCaGCCGG | + | 5 | 2 | 0 | 5.51 | 2.76 |
| Chr2:63390237-63390259 | 63390242 | 3 | RNA | GGCCGAGGTCGACTACCGGCNRG | GGC--AcGTCGACggCgGGaTGG | - | 5 | 2 | 0 | 5.51 | 2.76 |
| Chr1:102169381-102169403 | 102169386 | 3 | RNA | GGCCGAGGTCGACTACCGGCNRG | tGCCGA--TacACcgCCGGCCGG | - | 5 | 2 | 0 | 5.51 | 2.76 |
| Chr7:126216859-126216881 | 126216866 | 5 | DNA | GGCCGAGGT-CGACTACCGGCNRG | GGCgGAGGTCCGAtcACCGGCGAG | - | 3 | 1 | 0 | 5.51 | 2.76 |
| Chr1:253412138-253412160 | 253412143 | 3 | RNA | GGCCGAGGTCGACTACCGGCNRG | G-aCGgGGTCGACaACgaGCGGG | - | 5 | 1 | 0 | 5.51 | 2.76 |
| Chr6:155049463-155049485 | 155049479 | 4 | RNA | GGCCGAGGTCGACTACCGGCNRG | caCCaAGtTCGcCT-CCGGCCAG | + | 5 | 1 | 0 | 5.51 | 2.76 |
| Chr10:34498204-34498226 | 34498221 | 3 | RNA | GGCCGAGGTCGACTACCGGCNRG | cGCCGcGGTCGcCa--CGGtAGG | + | 5 | 2 | 0 | 5.51 | 2.76 |
| Chr3:127034085-127034107 | 127034103 | 2 | RNA | GGCCGAGGTCGACTACCGGCNRG | GGC--AGGagccCTACCGcCAAG | + | 5 | 2 | 0 | 5.51 | 2.76 |
| Chr4:91166567-91166589 | 91166583 | 4 | DNA | GGCCGAGGTC--GACTACCGGCNRG | GGCCGAGaTCTGGtCTAggGaCGAG | + | 5 | 2 | 0 | 5.51 | 2.76 |
| Chr7:121933023-121933045 | 121933030 | 5 | RNA | GGCCGAGGTCGACTACCGGCNRG | GGgCGAc--CGAagtCCGGCGAG | - | 5 | 2 | 0 | 5.51 | 2.76 |
| Chr10:24222972-24222994 | 24222978 | 4 | RNA | GGCCGAGGTCGACTACCGGCNRG | GGC--ttGcgGACTACCGGCTGG | - | 4 | 2 | 0 | 5.51 | 2.76 |
| Chr4:146867394-146867416 | 146867412 | 2 | RNA | GGCCGAGGTCGACTACCGGCNRG | cGCCcAGGTgcACg--CGGCAAG | + | 5 | 2 | 0 | 5.51 | 2.76 |
| Chr9:52196348-52196370 | 52196364 | 4 | RNA | GGCCGAGGTCGACTACCGGCNRG | G-aCGgGGTCGACaACgaGCGGG | + | 5 | 1 | 0 | 5.51 | 2.76 |
| Chr1:49000424-49000446 | 49000441 | 3 | DNA | GGC--CGAGGTCGACTACCGGCNRG | GGCCTCGgGGgCtACacCCGGCGGG | + | 5 | 2 | 0 | 5.51 | 2.76 |
| Chr5:30550117-30550139 | 30550134 | 3 | DNA | GGC-CGAGGTCGACTACCGGCNRG | GcCTCGAGGaCGtCTtCCGGCTGG | + | 4 | 1 | 0 | 5.51 | 2.76 |

| | | | | | | | | | | | |
|---|---|---|---|---|---|---|---|---|---|---|---|
| Chr5:76511711-76511733 | 76511729 | 2 | RNA | GGCCGAGGTCGACTACCGGCNRG | GaCCGAGG-CGACgACaaGaAGG | + | 5 | 1 | 0 | 5.51 | 2.76 |
| Chr8:65327322-65327344 | 65327326 | 2 | RNA | GGCCGAGGTCGACTACCGGCNRG | cGCCGAcGTCGAaTcCCG-aGAG | - | 5 | 1 | 0 | 5.51 | 2.76 |
| Chr10:64853316-64853338 | 64853334 | 2 | RNA | GGCCGAGGTCGACTACCGGCNRG | GGCgGAGcagGACT--CGcCAAG | + | 5 | 2 | 0 | 5.51 | 2.76 |
| Chr4:50998463-50998485 | 50998467 | 2 | RNA | GGCCGAGGTCGACTACCGGCNRG | GagaGgGGTCGgCT--CGGCCGG | - | 5 | 2 | 0 | 5.51 | 2.76 |
| Chr5:138899230-138899252 | 138899246 | 4 | RNA | GGCCGAGGTCGACTACCGGCNRG | ttCCGAtGTCGAtTA--tGCAGG | + | 5 | 2 | 0 | 5.51 | 2.76 |
| Chr2:18898307-18898329 | 18898311 | 2 | RNA | GGCCGAGGTCGACTACCGGCNRG | cGaCGcGGTCG--cACaGGCTGG | - | 5 | 2 | 0 | 5.51 | 2.76 |
| Chr4:45282082-45282104 | 45282098 | 4 | RNA | GGCCGAGGTCGACTACCGGCNRG | G-aCGgGGTCGACaACgaGCGGG | + | 5 | 1 | 0 | 5.51 | 2.76 |
| Chr7:10962590-10962612 | 10962597 | 5 | RNA | GGCCGAGGTCGACTACCGGCNRG | GGaCGAcccCGACT-CCGGtTGG | - | 5 | 1 | 0 | 5.51 | 2.76 |
| Chr4:204318800-204318822 | 204318804 | 2 | RNA | GGCCGAGGTCGACTACCGGCNRG | G--CGtGGTCGAtgACtGaCGGG | - | 5 | 2 | 0 | 5.51 | 2.76 |
| Chr4:20023417-20023439 | 20023435 | 2 | RNA | GGCCGAGGTCGACTACCGGCNRG | GGCCaAGGTCGAgg--CGttGGG | + | 5 | 2 | 0 | 5.51 | 2.76 |
| Chr3:152334774-152334796 | 152334780 | 4 | RNA | GGCCGAGGTCGACTACCGGCNRG | GGC--AGGcCGAgcACtaGCCGG | - | 5 | 2 | 0 | 5.51 | 2.76 |
| Chr1:111975106-111975128 | 111975111 | 3 | DNA | GGC--CGAGGTCGACTACCGGCNRG | GaCAACGgGGTCGACgACgaGCGGG | - | 5 | 2 | 0 | 5.51 | 2.76 |
| Chr4:173332208-173332230 | 173332226 | 2 | RNA | GGCCGAGGTCGACTACCGGCNRG | GGCgGAGGTgGAgg--CGGtGGG | + | 5 | 2 | 0 | 5.51 | 2.76 |
| Chr2:194479906-194479928 | 194479910 | 2 | RNA | GGCCGAGGTCGACTACCGGCNRG | tGtaGAGtT-GAaTACCGGCTGG | - | 5 | 1 | 0 | 5.51 | 2.76 |
| Chr5:57230321-57230343 | 57230339 | 2 | RNA | GGCCGAGGTCGACTACCGGCNRG | GGCCGccGcCGtCT--CtGCTGG | + | 5 | 2 | 0 | 5.51 | 2.76 |
| Chr5:112241586-112241608 | 112241602 | 4 | RNA | GGCCGAGGTCGACTACCGGCNRG | GGCCctGGTCGAC-ggCGGCGGG | + | 4 | 1 | 0 | 5.51 | 2.76 |
| Chr2:122542398-122542420 | 122542405 | 5 | RNA | GGCCGAGGTCGACTACCGGCNRG | GGCgcAacTCtACT-CCGGCGAG | - | 5 | 1 | 0 | 5.51 | 2.76 |
| Chr8:75250363-75250385 | 75250369 | 4 | RNA | GGCCGAGGTCGACTACCGGCNRG | GGCCGAGGgtGcCTtg-GGCTGG | - | 5 | 1 | 0 | 5.51 | 2.76 |
| Chr4:8562958-8562980 | 8562964 | 4 | DNA | GGCC-GAGGTCGACTACCGGCNRG | aGCCGGAccTCGACTACCGcCAAG | - | 4 | 1 | 0 | 5.51 | 2.76 |
| Chr5:145171136-145171158 | 145171151 | 5 | RNA | GGCCGAGGTCGACTACCGGCNRG | G-CCGAaGTacACTgCCtGCTGG | + | 5 | 1 | 0 | 5.51 | 2.76 |
| Chr2:108353742-108353764 | 108353758 | 4 | RNA | GGCCGAGGTCGACTACCGGCNRG | GaC--AGGTCGACcAaCtGaGAG | + | 5 | 2 | 0 | 5.51 | 2.76 |
| Chr6:139324076-139324098 | 139324093 | 3 | RNA | GGCCGAGGTCGACTACCGGCNRG | GcCgGAGccCGAC--CCGtCAAG | + | 5 | 2 | 0 | 5.51 | 2.76 |
| Chr1:95966370-95966392 | 95966375 | 3 | DNA | GGCCGAGGTCGACTACCG-GCNRG | GGCCGAGGcCGACcAtgGAGtAGG | - | 5 | 1 | 0 | 5.51 | 2.76 |
| Chr7:116767343-116767365 | 116767347 | 2 | DNA | GGCCGAGGTCGACTAC-CGGCNRG | GGCCGgGGcCGAgggCTCGGCGGG | - | 5 | 1 | 0 | 5.51 | 2.76 |
| Chr8:102046492-102046514 | 102046510 | 2 | RNA | GGCCGAGGTCGACTACCGGCNRG | GGCC-AGGTCGtCcACtGGCGGG | + | 3 | 1 | 0 | 5.51 | 2.76 |
| Chr10:105155678-105155700 | 105155683 | 3 | RNA | GGCCGAGGTCGACTACCGGCNRG | GcCCGgGGaaG--TACCcGCTGG | - | 5 | 2 | 0 | 5.51 | 2.76 |
| Chr4:85296426-85296448 | 85296441 | 5 | DNA | GGCC--GAGGTCGACTACCGGCNRG | GaCCTGGAGGTacACTACCacCCAG | + | 5 | 2 | 0 | 5.51 | 2.76 |
| Chr7:96700804-96700826 | 96700820 | 4 | RNA | GGCCGAGGTCGACTACCGGCNRG | GGaCcAGGTCGAggAC-GGCGGG | + | 4 | 1 | 0 | 5.51 | 2.76 |
| Chr8:106926614-106926636 | 106926631 | 3 | DNA | GGCC--GAGGTCGACTACCGGCNRG | GGCCCGGcGcTCGACcttCGGCAGG | + | 5 | 2 | 0 | 5.51 | 2.76 |
| Chr8:22618385-22618407 | 22618389 | 2 | DNA | GGCCGAGGTCGACTA-CCGGCNRG | GGCCaAGGTCGACgAGgaaGCAGG | - | 5 | 1 | 0 | 5.51 | 2.76 |
| Chr9:100306060-100306082 | 100306075 | 5 | RNA | GGCCGAGGTCGACTACCGGCNRG | GGagGAGcgCGAC--CCcGCGGG | + | 5 | 2 | 0 | 5.51 | 2.76 |
| Chr1:218649619-218649641 | 218649625 | 4 | RNA | GGCCGAGGTCGACTACCGGCNRG | tcCCGAG--CagCTAaCGGCGAG | - | 5 | 2 | 0 | 5.51 | 2.76 |
| Chr4:35292644-35292666 | 35292662 | 2 | RNA | GGCCGAGGTCGACTACCGGCNRG | aGaCaAGGTC--CTACaGGCCAG | + | 4 | 2 | 0 | 5.51 | 2.76 |
| Chr2:214053615-214053637 | 214053620 | 3 | RNA | GGCCGAGGTCGACTACCGGCNRG | G-aCGgGGTCGACaACgaGCGGG | - | 5 | 1 | 0 | 5.51 | 2.76 |
| Chr8:47814225-47814247 | 47814231 | 4 | RNA | GGCCGAGGTCGACTACCGGCNRG | GG--GcGGcCGtggACCGGCGAG | - | 5 | 2 | 0 | 5.51 | 2.76 |
| Chr3:158011792-158011814 | 158011808 | 4 | RNA | GGCCGAGGTCGACTACCGGCNRG | G-aCGgGGTCGACaACgaGCGGG | + | 5 | 1 | 0 | 5.51 | 2.76 |
| Chr9:76069600-76069622 | 76069604 | 2 | DNA | GGCCGAGGTCGACTACCGG--CNRG | GGCCGctGTgGgaTACCGGTCCCGG | - | 5 | 2 | 0 | 5.51 | 2.76 |
| Chr5:50499058-50499080 | 50499063 | 3 | RNA | GGCCGAGGTCGACTACCGGCNRG | tGCtGgGGTCccCTACC--CTGG | - | 5 | 2 | 0 | 5.51 | 2.76 |
| Chr4:117007892-117007914 | 117007908 | 4 | RNA | GGCCGAGGTCGACTACCGGCNRG | GtCCGcGGTC-ACcACCGGCCGG | + | 3 | 1 | 0 | 5.51 | 2.76 |
| Chr8:118315427-118315449 | 118315443 | 4 | X | GGCCGAGGTCGACTACCGGCNRG | GGaCGAcGaCGACTAgCaGCAGG | + | 5 | 0 | 0 | 5.51 | 2.76 |
| Chr3:64552259-64552281 | 64552277 | 2 | DNA | GGCCGAGGTCGACT-ACCGGCNRG | GGggGAGGagaACTCACCGGCGGG | + | 5 | 1 | 0 | 5.51 | 2.76 |
| Chr5:111781100-111781122 | 111781107 | 5 | RNA | GGCCGAGGTCGACTACCGGCNRG | GGCacAacTCaACT-CCGGCGAG | - | 5 | 1 | 0 | 5.51 | 2.76 |
| Chr4:11473236-11473258 | 11473240 | 2 | RNA | GGCCGAGGTCGACTACCGGCNRG | aGgttAGGTCGACaA--GGCCGG | - | 5 | 2 | 0 | 5.51 | 2.76 |
| Chr7:129386860-129386882 | 129386865 | 3 | RNA | GGCCGAGGTCGACTACCGGCNRG | G-aCGAGGTCGACaACgaGCGGG | - | 4 | 1 | 1 | 4.41 | 2.71 |
| Chr9:130431026-130431048 | 130431031 | 3 | RNA | GGCCGAGGTCGACTACCGGCNRG | G-aCGgGGTCGACaACgaGCGGG | - | 5 | 1 | 2 | 3.30 | 2.65 |
| Chr2:74528155-74528177 | 74528172 | 3 | DNA | GGC-CGAGGTCGACTACCGGCNRG | GcCTCGAGGaCGtCTtCCGGCTGG | + | 4 | 1 | 2 | 3.30 | 2.65 |
| Chr1:183300743-183300765 | 183300749 | 4 | RNA | GGCCGAGGTCGACTACCGGCNRG | G-CCtcGGgCGAaTACtGGCAGG | - | 5 | 1 | 3 | 2.20 | 2.60 |
| Chr2:151640455-151640477 | 151640460 | 3 | RNA | GGCCGAGGTCGACTACCGGCNRG | G-aCGgGGTCGACgACgaGCGGG | - | 5 | 1 | 4 | 1.10 | 2.55 |
| Chr5:218406195-218406217 | 218406212 | 3 | RNA | GGCCGAGGTCGACTACCGGCNRG | cG--GAGGggGAaTACCcGCTGG | + | 5 | 2 | 4 | 1.10 | 2.55 |
| Chr7:172676964-172676986 | 172676981 | 3 | RNA | GGCCGAGGTCGACTACCGGCNRG | tGCCGAGtTgG--TcCCtGCTGG | + | 5 | 2 | 5 | 0.00 | 2.50 |
| Chr9:7785646-7785668 | 7785653 | 5 | DNA | GGCCGAGG--TCGACTACCGGCNRG | cGgCGAGGCATCGACcACCtGaGAG | - | 5 | 2 | 5 | 0.00 | 2.50 |
| Chr3:41459758-41459780 | 41459775 | 3 | RNA | GGCCGAGGTCGACTACCGGCNRG | caCCGAGGTtGAt--CCGGtGAG | + | 5 | 2 | 5 | 0.00 | 2.50 |
| Chr2:127323580-127323602 | 127323587 | 5 | RNA | GGCCGAGGTCGACTACCGGCNRG | aGttGAGcTCGACTACt--CGAG | - | 5 | 2 | 5 | 0.00 | 2.50 |
| Chr8:58388506-58388528 | 58388513 | 5 | RNA | GGCCGAGGTCGACTACCGGCNRG | GGtCGAG-TCtACTACactCAAG | - | 5 | 1 | 5 | 0.00 | 2.50 |
| Chr7:55023024-55023046 | 55023031 | 5 | RNA | GGCCGAGGTCGACTACCGGCNRG | aGCCGAGGgtGcCT-CgGGCTGG | - | 5 | 1 | 5 | 0.00 | 2.50 |
| Chr3:120787484-120787506 | 120787501 | 3 | RNA | GGCCGAGGTCGACTACCGGCNRG | GG--GAGGTCGgCTtCgtGtGAG | + | 5 | 2 | 5 | 0.00 | 2.50 |
| Chr5:45619841-45619863 | 45619858 | 3 | RNA | GGCCGAGGTCGACTACCGGCNRG | G--CGAGGTgGACTgCgGGCAAG | + | 3 | 2 | 5 | 0.00 | 2.50 |
| Chr3:177913195-177913217 | 177913202 | 5 | RNA | GGCCGAGGTCGACTACCGGCNRG | cGaCaAcGTCGACTACCa-CGAG | - | 5 | 1 | 5 | 0.00 | 2.50 |
| Chr4:133565679-133565701 | 133565686 | 5 | RNA | GGCCGAGGTCGACTACCGGCNRG | GGgCtcGGTC--CTgCgGGCAGG | - | 5 | 2 | 5 | 0.00 | 2.50 |
| Chr9:154291337-154291359 | 154291344 | 5 | RNA | GGCCGAGGTCGACTACCGGCNRG | aGCaGAG--CaACaACCGaCAAG | - | 5 | 2 | 5 | 0.00 | 2.50 |
| Chr10:65936308-65936330 | 65936315 | 5 | RNA | GGCCGAGGTCGACTACCGGCNRG | GtCCG--GTgcACcACCGGaCAG | - | 5 | 2 | 5 | 0.00 | 2.50 |
| Chr2:231454238-231454260 | 231454255 | 3 | RNA | GGCCGAGGTCGACTACCGGCNRG | GGCCaAcGaCaACgACC--CGAG | + | 5 | 2 | 5 | 0.00 | 2.50 |
| Chr7:120068997-120069019 | 120069014 | 3 | RNA | GGCCGAGGTCGACTACCGGCNRG | GGCtaAGGTtGcCTtC-GGCAGG | + | 5 | 1 | 5 | 0.00 | 2.50 |
| Chr1:192787286-192787308 | 192787303 | 3 | DNA | GGCCGAGGTCG-ACTACCGGCNRG | GGgCGAGGTCGGACTtCgGcgAGG | + | 5 | 1 | 5 | 0.00 | 2.50 |
| Chr5:103391091-103391113 | 103391098 | 5 | RNA | GGCCGAGGTCGACTACCGGCNRG | GG--GAaGcCGAaatCCGGCGAG | - | 5 | 2 | 5 | 0.00 | 2.50 |
| Chr10:38036968-38036990 | 38036975 | 5 | RNA | GGCCGAGGTCGACTACCGGCNRG | GaagGAGcTCGACTg--GGCGAG | - | 5 | 2 | 5 | 0.00 | 2.50 |
| Chr8:171311548-171311570 | 171311555 | 5 | DNA | GGCC-GAGGTCGACTACCGGCNRG | cGCCTGAGGTCaACctCgGGCAGG | - | 5 | 1 | 5 | 0.00 | 2.50 |
| Chr1:97695165-97695187 | 97695182 | 3 | RNA | GGCCGAGGTCGACTACCGGCNRG | GG--GAGaTgGACgcCCGGaGAG | + | 5 | 2 | 5 | 0.00 | 2.50 |
| Chr8:144061144-144061166 | 144061151 | 5 | RNA | GGCCGAGGTCGACTACCGGCNRG | GGgCG-GGcCaACctCCGGCTGG | - | 5 | 1 | 5 | 0.00 | 2.50 |
| Chr5:223785019-223785041 | 223785026 | 5 | RNA | GGCCGAGGTCGACTACCGGCNRG | GGCCGgGccaGACcA--GGCTGG | - | 5 | 2 | 5 | 0.00 | 2.50 |
| Chr1:217765429-217765451 | 217765436 | 5 | DNA | GGCCGAGGTCGACT--ACCGGCNRG | GaCCtAGtcCGACTCAACCGGaTGG | - | 5 | 2 | 5 | 0.00 | 2.50 |
| Chr8:85459239-85459261 | 85459256 | 3 | RNA | GGCCGAGGTCGACTACCGGCNRG | GGaCGAGGTtG--ggCCGGCCGG | + | 4 | 2 | 5 | 0.00 | 2.50 |
| Chr7:1512817-1512839 | 1512824 | 5 | RNA | GGCCGAGGTCGACTACCGGCNRG | GGtCGAGGcCGAgT--CtGgGGG | - | 5 | 2 | 5 | 0.00 | 2.50 |
| Chr1:211205132-211205154 | 211205139 | 5 | RNA | GGCCGAGGTCGACTACCGGCNRG | G-aCGgGGTCGACaACgaGCGGG | - | 5 | 1 | 5 | 0.00 | 2.50 |
| Chr6:159078741-159078763 | 159078758 | 3 | RNA | GGCCGAGGTCGACTACCGGCNRG | GcCgGAGccCGAC--CCGtCAGG | + | 5 | 2 | 5 | 0.00 | 2.50 |
| Chr1:130411725-130411747 | 130411742 | 3 | RNA | GGCCGAGGTCGACTACCGGCNRG | GGtCGtGGTCGAC--CgGaaCGG | + | 5 | 2 | 5 | 0.00 | 2.50 |
| Chr1:162784612-162784634 | 162784619 | 5 | RNA | GGCCGAGGTCGACTACCGGCNRG | GGCCGAtcTCG--cAtCaGCAAG | - | 5 | 2 | 5 | 0.00 | 2.50 |
| Chr6:144539158-144539180 | 144539175 | 3 | DNA | GGC--CGAGGTCGACTACCGGCNRG | GaCAACGgGGTCGACgACgaGCGGG | + | 5 | 2 | 5 | 0.00 | 2.50 |
| Chr2:65855349-65855371 | 65855356 | 5 | DNA | GGCCGAGGTCGACTACCGG--CNRG | GGtCGAGGTCGAgTcCaaGCCCCGG | - | 5 | 2 | 5 | 0.00 | 2.50 |
| Chr10:86321315-86321337 | 86321322 | 5 | RNA | GGCCGAGGTCGACTACCGGCNRG | aGCCGAGGgtGcCT-CgGGCTGG | - | 5 | 1 | 5 | 0.00 | 2.50 |
| Chr4:46369098-46369120 | 46369115 | 3 | RNA | GGCCGAGGTCGACTACCGGCNRG | GaCCGAtGgCGAggAC--GCGGG | + | 5 | 2 | 5 | 0.00 | 2.50 |
| Chr7:66541582-66541604 | 66541599 | 3 | RNA | GGCCGAGGTCGACTACCGGCNRG | GGCaGAGGgaGcCaA--GGCGAG | + | 5 | 2 | 5 | 0.00 | 2.50 |
| Chr10:136500128-136500150 | 136500135 | 5 | RNA | GGCCGAGGTCGACTACCGGCNRG | GGgCGAcGT--ACTAtCGGgGGG | - | 4 | 2 | 5 | 0.00 | 2.50 |
| Chr10:97117459-97117481 | 97117476 | 3 | RNA | GGCCGAGGTCGACTACCGGCNRG | GGgaGAGGgC--CTACCttCAGG | + | 5 | 2 | 5 | 0.00 | 2.50 |
| Chr10:77052172-77052194 | 77052179 | 5 | RNA | GGCCGAGGTCGACTACCGGCNRG | GGC--AGGcCaACctCCGGCTGG | - | 4 | 2 | 5 | 0.00 | 2.50 |
| Chr9:60479550-60479572 | 60479567 | 3 | RNA | GGCCGAGGTCGACTACCGGCNRG | tGCCGAG--aGgCTtCgGGCAGG | + | 5 | 2 | 5 | 0.00 | 2.50 |
| Chr10:135176533-135176555 | 135176540 | 5 | DNA | GGCCGAGGTCGACTACCG--GCNRG | GGCCGgGGTCGgCccaCGGCGCGGG | - | 5 | 2 | 5 | 0.00 | 2.50 |
| Chr1:208246274-208246296 | 208246291 | 3 | RNA | GGCCGAGGTCGACTACCGGCNRG | GtCaGAGacCGAt--CCGGCCAG | + | 5 | 2 | 5 | 0.00 | 2.50 |
| Chr6:33387550-33387572 | 33387567 | 3 | RNA | GGCCGAGGTCGACTACCGGCNRG | cGCCGAGGTtG--TcCaGGgAAG | + | 5 | 2 | 5 | 0.00 | 2.50 |
| Chr3:211293273-211293295 | 211293280 | 5 | RNA | GGCCGAGGTCGACTACCGGCNRG | aGC--AGGgCGgCatCCGGCAGG | - | 5 | 2 | 5 | 0.00 | 2.50 |
| Chr7:66242738-66242760 | 66242745 | 5 | RNA | GGCCGAGGTCGACTACCGGCNRG | GGCCG--GTCcACctgCGGaAGG | - | 5 | 2 | 5 | 0.00 | 2.50 |
| Chr10:111236500-111236522 | 111236507 | 5 | RNA | GGCCGAGGTCGACTACCGGCNRG | cGaCt--GTCGACgACCtGCAGG | - | 5 | 2 | 5 | 0.00 | 2.50 |
| Chr10:124294629-124294651 | 124294646 | 3 | RNA | GGCCGAGGTCGACTACCGGCNRG | G-aCGgGGTCGACgACgaGCGGG | + | 5 | 1 | 5 | 0.00 | 2.50 |
| Chr4:123728515-123728537 | 123728532 | 3 | DNA | GGCCGAGGTCGACTACCG--GCNRG | GcCCGAGGgCaACcACCGTAGCCAG | + | 4 | 2 | 5 | 0.00 | 2.50 |
| Chr1:292730640-292730662 | 292730647 | 5 | DNA | GGCCGAGGTC--GACTACCGGCNRG | GGCgGAGGTCTGGACTtaCatCCGG | - | 5 | 2 | 5 | 0.00 | 2.50 |
| Chr4:180225805-180225827 | 180225822 | 3 | RNA | GGCCGAGGTCGACTACCGGCNRG | G-tCGgGGTCGACaACaaGCGGG | + | 5 | 1 | 5 | 0.00 | 2.50 |
| Chr2:159572671-159572693 | 159572688 | 3 | DNA | GGCCGAGGTCG--ACTACCGGCNRG | GGCacAGGgaGAAACTAtCGGCGAG | + | 5 | 2 | 5 | 0.00 | 2.50 |
| Chr10:98651813-98651835 | 98651820 | 5 | DNA | GGCCGA-GGTCGACTACCGGCNRG | GGCgGACGGTCcgCgACCGGCCAG | - | 4 | 1 | 5 | 0.00 | 2.50 |
| Chr5:109297039-109297061 | 109297046 | 5 | RNA | GGCCGAGGTCGACTACCGGCNRG | GGtCGAtcTCG--TAtCaGCAAG | - | 5 | 2 | 5 | 0.00 | 2.50 |
| Chr2:16319893-16319915 | 16319900 | 5 | RNA | GGCCGAGGTCGACTACCGGCNRG | GtCCtAGG-CaACaACaGGCCGG | - | 5 | 1 | 5 | 0.00 | 2.50 |
| Chr3:223007823-223007845 | 223007830 | 5 | RNA | GGCCGAGGTCGACTACCGGCNRG | GaCCGAGaaacACT-CCGGCGAG | - | 5 | 1 | 5 | 0.00 | 2.50 |
| Chr5:176728105-176728127 | 176728112 | 5 | RNA | GGCCGAGGTCGACTACCGGCNRG | GGC-GAGGTgGAtggaCGGCGAG | - | 5 | 1 | 5 | 0.00 | 2.50 |
| Chr5:21270867-21270889 | 21270874 | 5 | RNA | GGCCGAGGTCGACTACCGGCNRG | GcCCGAGGgCaACcACC--gTGG | - | 5 | 2 | 5 | 0.00 | 2.50 |
| Chr1:299750699-299750721 | 299750716 | 3 | RNA | GGCCGAGGTCGACTACCGGCNRG | GtCCG--GTgcACcACCGGaCAG | + | 5 | 2 | 5 | 0.00 | 2.50 |
| Chr8:134959558-134959580 | 134959565 | 5 | RNA | GGCCGAGGTCGACTACCGGCNRG | GGCCG-GGTCGtCcACtGGgGGG | - | 4 | 1 | 5 | 0.00 | 2.50 |
| Chr2:201473809-201473831 | 201473826 | 3 | RNA | GGCCGAGGTCGACTACCGGCNRG | GGC--AGGcCGAgcACCaGCCGG | + | 4 | 2 | 5 | 0.00 | 2.50 |
| Chr8:110600319-110600341 | 110600326 | 5 | RNA | GGCCGAGGTCGACTACCGGCNRG | GGC--AGGcCGAgcACCGGCCGG | - | 3 | 2 | 5 | 0.00 | 2.50 |
| Chr1:114523409-114523431 | 114523426 | 3 | RNA | GGCCGAGGTCGACTACCGGCNRG | GaCaGAGG--GAtTcCaGGCTAG | + | 5 | 2 | 5 | 0.00 | 2.50 |
| Chr9:89546799-89546821 | 89546806 | 5 | RNA | GGCCGAGGTCGACTACCGGCNRG | cGCaGAGaT-GgCcACCGGCGAG | - | 5 | 1 | 5 | 0.00 | 2.50 |
| Chr10:29992070-29992092 | 29992087 | 3 | RNA | GGCCGAGGTCGACTACCGGCNRG | tGgCGAGcctG--TACCGGCCAG | + | 5 | 2 | 5 | 0.00 | 2.50 |
| Chr7:27340483-27340505 | 27340490 | 5 | RNA | GGCCGAGGTCGACTACCGGCNRG | GaCCGAGcTgGtCT-CCGtCCAG | - | 5 | 1 | 5 | 0.00 | 2.50 |
| Chr2:151303439-151303461 | 151303456 | 3 | DNA | GGCCGAGGTCGACTACC--GGCNRG | GGCgGAGGTCGtCggCaGAGGCCAG | + | 5 | 2 | 5 | 0.00 | 2.50 |
| Chr1:207676943-207676965 | 207676960 | 3 | RNA | GGCCGAGGTCGACTACCGGCNRG | GGCaGAGGcaGcCgACC--CAGG | + | 5 | 2 | 5 | 0.00 | 2.50 |
| Chr4:130241005-130241027 | 130241012 | 5 | RNA | GGCCGAGGTCGACTACCGGCNRG | G--CGAGGTCcACggCaaGCCGG | - | 5 | 2 | 5 | 0.00 | 2.50 |
| Chr5:216916984-216917006 | 216917001 | 3 | RNA | GGCCGAGGTCGACTACCGGCNRG | GGaCtAGGTCG--TcCCaGtTGG | + | 5 | 2 | 5 | 0.00 | 2.50 |
| Chr8:52567473-52567495 | 52567480 | 5 | RNA | GGCCGAGGTCGACTACCGGCNRG | GGCCGAtcTCG--cAtCaGCAAG | - | 5 | 2 | 5 | 0.00 | 2.50 |
| Chr7:57909967-57909989 | 57909984 | 3 | RNA | GGCCGAGGTCGACTACCGGCNRG | G-CCGAGcaCGACgACttGCTGG | + | 5 | 1 | 5 | 0.00 | 2.50 |
| Chr5:193317877-193317899 | 193317884 | 5 | DNA | GGC--CGAGGTCGACTACCGGCNRG | GGCCTCGgGGgCtACacCCGGCGGG | - | 5 | 2 | 5 | 0.00 | 2.50 |
| Chr4:194333577-194333599 | 194333584 | 5 | RNA | GGCCGAGGTCGACTACCGGCNRG | GGCCGAGGgCGcgaAC--GCGGG | - | 4 | 2 | 5 | 0.00 | 2.50 |
| Chr4:214388654-214388676 | 214388671 | 3 | RNA | GGCCGAGGTCGACTACCGGCNRG | GGC-GcGGTCGACggCgaGCAAG | + | 5 | 1 | 5 | 0.00 | 2.50 |
| Chr3:17115923-17115945 | 17115930 | 5 | RNA | GGCCGAGGTCGACTACCGGCNRG | GGtCGAGGTtGACTgC--GgTGG | - | 4 | 2 | 5 | 0.00 | 2.50 |
| Chr4:95457064-95457086 | 95457081 | 3 | RNA | GGCCGAGGTCGACTACCGGCNRG | GcCCatGGTCGAgTtC--GCCAG | + | 5 | 2 | 5 | 0.00 | 2.50 |
| Chr7:123821724-123821746 | 123821731 | 5 | DNA | GGC--CGAGGTCGACTACCGGCNRG | GGCCTCGgGGgCtACacCCGGCGGG | - | 5 | 2 | 5 | 0.00 | 2.50 |
| Chr4:230157569-230157591 | 230157576 | 5 | RNA | GGCCGAGGTCGACTACCGGCNRG | aGCCGAGGgCGcCT-CgGGCAGG | - | 4 | 1 | 5 | 0.00 | 2.50 |
| Chr5:146175700-146175722 | 146175707 | 5 | DNA | GGCCGAGGTCGAC-TACCGGCNRG | GGCCGgcGgCGAgGaACCGGCCGG | - | 5 | 1 | 5 | 0.00 | 2.50 |
| Chr3:217269250-217269272 | 217269267 | 3 | RNA | GGCCGAGGTCGACTACCGGCNRG | GGCCtAGcT--ACTACtGatTGG | + | 5 | 2 | 5 | 0.00 | 2.50 |

| | | | | | | | | | | | |
|---|---|---|---|---|---|---|---|---|---|---|---|
| Chr4:70087974-70087996 | 70087991 | 3 | RNA | GGCCGAGGTCGACTACCGGCNRG | GcCCGAGGaCcACcACC--tTGG | + | 5 | 2 | 5 | 0.00 | 2.50 |
| Chr6:61773294-61773316 | 61773311 | 3 | DNA | GGCCGAGGTCG-ACTACCGGCNRG | GGCCGAGaTCGGACggCCcaCGGG | + | 5 | 1 | 5 | 0.00 | 2.50 |
| Chr3:9295106-9295128 | 9295123 | 3 | RNA | GGCCGAGGTCGACTACCGGCNRG | GGgCG-GGcCaACctCCGGCCGG | + | 5 | 1 | 5 | 0.00 | 2.50 |
| Chr7:101933327-101933349 | 101933334 | 5 | RNA | GGCCGAGGTCGACTACCGGCNRG | GGCCcAG--aGAtTgCaGGCCGG | - | 5 | 2 | 5 | 0.00 | 2.50 |
| Chr3:129442814-129442836 | 129442821 | 5 | RNA | GGCCGAGGTCGACTACCGGCNRG | GaCaG-GGTCGACaACgaGCGGG | - | 5 | 1 | 5 | 0.00 | 2.50 |
| Chr3:145021398-145021420 | 145021415 | 3 | RNA | GGCCGAGGTCGACTACCGGCNRG | GGCtGgGGgCGA-TACtGGgTGG | + | 5 | 1 | 5 | 0.00 | 2.50 |
| Chr1:239838730-239838752 | 239838737 | 5 | RNA | GGCCGAGGTCGACTACCGGCNRG | GGC--ttGcgGACTACCGGCTGG | - | 4 | 2 | 5 | 0.00 | 2.50 |
| Chr1:198971584-198971606 | 198971601 | 3 | DNA | GGCCGAGGT--CGACTACCGGCNRG | GcCCGAcGTGGCGACTtCCaGCAAG | + | 4 | 2 | 5 | 0.00 | 2.50 |
| Chr3:97830725-97830747 | 97830732 | 5 | DNA | GGCCGAGGTCGA--CTACCGGCNRG | GGCgGAGGTgGAGGCTgtgGGCAAG | - | 5 | 2 | 5 | 0.00 | 2.50 |
| Chr1:194591431-194591453 | 194591438 | 5 | RNA | GGCCGAGGTCGACTACCGGCNRG | GGaCGAcccCGACT-CCGGtTAG | - | 5 | 1 | 5 | 0.00 | 2.50 |
| Chr10:81885111-81885133 | 81885128 | 3 | RNA | GGCCGAGGTCGACTACCGGCNRG | GtCCG--GTgcACcACCGGaCAG | + | 5 | 2 | 5 | 0.00 | 2.50 |
| Chr2:161400844-161400866 | 161400861 | 3 | RNA | GGCCGAGGTCGACTACCGGCNRG | GGCCGAGGgtGtCT-CgGGCTGG | + | 4 | 1 | 5 | 0.00 | 2.50 |
| Chr4:23415534-23415556 | 23415551 | 3 | RNA | GGCCGAGGTCGACTACCGGCNRG | tGCCGAaGaCGAC-AgCaGCAGG | + | 5 | 1 | 5 | 0.00 | 2.50 |
| Chr3:156398865-156398887 | 156398872 | 5 | RNA | GGCCGAGGTCGACTACCGGCNRG | GGC--ttGcgGACTACtGGCTGG | - | 5 | 2 | 5 | 0.00 | 2.50 |
| Chr2:44925552-44925574 | 44925569 | 3 | RNA | GGCCGAGGTCGACTACCGGCNRG | G-tCGAGcaCGAgTACCcGCAGG | + | 5 | 1 | 5 | 0.00 | 2.50 |
| Chr2:199451763-199451785 | 199451780 | 3 | RNA | GGCCGAGGTCGACTACCGGCNRG | GGCCGAGtTCGAg--CCctgGGG | + | 5 | 2 | 5 | 0.00 | 2.50 |
| Chr1:182580711-182580733 | 182580718 | 5 | RNA | GGCCGAGGTCGACTACCGGCNRG | GGCCGAccTCGgCaA--GGaCAG | - | 5 | 2 | 5 | 0.00 | 2.50 |
| Chr4:206598736-206598758 | 206598743 | 5 | RNA | GGCCGAGGTCGACTACCGGCNRG | GGaCaAGcT-GACgACgGGCAGG | - | 5 | 1 | 5 | 0.00 | 2.50 |
| Chr3:100225721-100225743 | 100225738 | 3 | RNA | GGCCGAGGTCGACTACCGGCNRG | GaCCaAGGTCaACTgCt--CGGG | + | 5 | 2 | 5 | 0.00 | 2.50 |
| Chr2:59873070-59873092 | 59873087 | 3 | RNA | GGCCGAGGTCGACTACCGGCNRG | GGC--tGGcaGACTACCGGCTGG | + | 3 | 2 | 5 | 0.00 | 2.50 |
| Chr1:137635972-137635994 | 137635989 | 3 | RNA | GGCCGAGGTCGACTACCGGCNRG | GGCCGAtcTCG--cAtCaGCAAG | + | 5 | 2 | 5 | 0.00 | 2.50 |
| Chr2:34031210-34031232 | 34031227 | 3 | RNA | GGCCGAGGTCGACTACCGGCNRG | G--CGAcGaCGACccCgGGCGAG | + | 5 | 2 | 5 | 0.00 | 2.50 |
| Chr1:218040835-218040857 | 218040852 | 3 | RNA | GGCCGAGGTCGACTACCGGCNRG | GGCgGAGGTCG--TtCgGtaGAG | + | 5 | 2 | 5 | 0.00 | 2.50 |
| Chr1:118689618-118689640 | 118689625 | 5 | RNA | GGCCGAGGTCGACTACCGGCNRG | tagCGAGGTgGgCTAC--GCGGG | - | 5 | 2 | 5 | 0.00 | 2.50 |
| Chr10:137336849-137336871 | 137336866 | 3 | RNA | GGCCGAGGTCGACTACCGGCNRG | GGtCGAGGT--ACTtCaaGCCGG | + | 4 | 2 | 5 | 0.00 | 2.50 |
| Chr7:132378711-132378733 | 132378718 | 5 | RNA | GGCCGAGGTCGACTACCGGCNRG | GGCCGAGGcCGAgccCC--gGGG | - | 5 | 2 | 5 | 0.00 | 2.50 |
| Chr8:93717635-93717657 | 93717642 | 5 | RNA | GGCCGAGGTCGACTACCGGCNRG | GGCCGAG--CaAggAgCGcCAAG | - | 5 | 2 | 5 | 0.00 | 2.50 |
| Chr2:30605462-30605484 | 30605479 | 3 | RNA | GGCCGAGGTCGACTACCGGCNRG | GGgaGAGGTCcACgAC--GgCGG | + | 5 | 2 | 5 | 0.00 | 2.50 |
| Chr4:211794527-211794549 | 211794534 | 5 | X | GGCCGAGGTCGACTACCGGCNRG | GGCCGAGGcCGAgctCgGGCGAG | - | 5 | 0 | 5 | 0.00 | 2.50 |
| Chr9:52942755-52942777 | 52942762 | 5 | RNA | GGCCGAGGTCGACTACCGGCNRG | G-aCGgGGTCGACgACgaGCGGG | - | 5 | 1 | 5 | 0.00 | 2.50 |
| Chr9:88453297-88453319 | 88453314 | 3 | RNA | GGCCGAGGTCGACTACCGGCNRG | GG--GAGGatGACTAgCGtgAGG | + | 5 | 2 | 5 | 0.00 | 2.50 |
| Chr5:3147718-3147740 | 3147725 | 5 | RNA | GGCCGAGGTCGACTACCGGCNRG | GGCCaAa--CGACaAtCtGCTGG | - | 5 | 2 | 5 | 0.00 | 2.50 |
| Chr4:239488684-239488706 | 239488701 | 3 | RNA | GGCCGAGGTCGACTACCGGCNRG | GGCCGcGGcCGAtT--CGGCCGG | + | 3 | 2 | 5 | 0.00 | 2.50 |
| Chr2:72470288-72470310 | 72470305 | 3 | RNA | GGCCGAGGTCGACTACCGGCNRG | GaCCGAa--CGACgACCGGCAAG | + | 3 | 2 | 5 | 0.00 | 2.50 |
| Chr1:262569085-262569107 | 262569102 | 3 | RNA | GGCCGAGGTCGACTACCGGCNRG | GGCCGctGcCG-CcgCCGGCGAG | + | 5 | 1 | 5 | 0.00 | 2.50 |
| Chr10:67750513-67750535 | 67750520 | 5 | RNA | GGCCGAGGTCGACTACCGGCNRG | GGtaGAGGagGAC-gCCGGCTGG | - | 5 | 1 | 5 | 0.00 | 2.50 |
| Chr2:53062851-53062873 | 53062858 | 5 | DNA | GGCCGAGGTCGACTA--CCGGCNRG | ctCCaAGGTCGACcAATCCGGCAGG | - | 4 | 2 | 5 | 0.00 | 2.50 |
| Chr9:7013318-7013340 | 7013325 | 5 | DNA | GGC-CGAGGTCGACTACCGGCNRG | GcCTCGgGGaCGtCTtCCGGCTGG | - | 5 | 1 | 5 | 0.00 | 2.50 |
| Chr9:82081647-82081669 | 82081654 | 5 | DNA | GGCCGAGGT-CGACTACCGGCNRG | cGgCGAGGTACGAactCCGGCGAG | - | 5 | 1 | 5 | 0.00 | 2.50 |
| Chr5:151398762-151398784 | 151398779 | 3 | RNA | GGCCGAGGTCGACTACCGGCNRG | GG--GAGaTgaACTtCCGGaGGG | + | 5 | 2 | 5 | 0.00 | 2.50 |
| Chr1:304742668-304742690 | 304742685 | 3 | RNA | GGCCGAGGTCGACTACCGGCNRG | aGCCGgG--aGAaTACCtGCGGG | + | 5 | 2 | 5 | 0.00 | 2.50 |
| Chr2:17907161-17907183 | 17907178 | 3 | DNA | GGCCGAGGTCGACTA--CCGGCNRG | ctCCtAGGcCGACcAATCCGGCAGG | + | 5 | 2 | 5 | 0.00 | 2.50 |
| Chr1:92062580-92062602 | 92062587 | 5 | RNA | GGCCGAGGTCGACTACCGGCNRG | GtCttgGGT-GACcACCGGCGAG | - | 5 | 1 | 5 | 0.00 | 2.50 |
| Chr2:133229184-133229206 | 133229201 | 3 | RNA | GGCCGAGGTCGACTACCGGCNRG | G--CGAGGgCtgCTACCGGCCGG | + | 3 | 2 | 5 | 0.00 | 2.50 |
| Chr4:44510781-44510803 | 44510798 | 3 | RNA | GGCCGAGGTCGACTACCGGCNRG | cGCCGAGtTaGAgTA--GGCAGG | + | 4 | 2 | 5 | 0.00 | 2.50 |
| Chr8:114282077-114282099 | 114282084 | 5 | DNA | GGCCGAGGTCG-ACTACCGGCNRG | tcCCGAGGTCGCACggCtGGCCAG | - | 5 | 1 | 5 | 0.00 | 2.50 |
| Chr1:282902439-282902461 | 282902456 | 3 | RNA | GGCCGAGGTCGACTACCGGCNRG | GGCCaAGcgCG--gACCGtCCGG | + | 5 | 2 | 5 | 0.00 | 2.50 |
| Chr7:182231025-182231047 | 182231042 | 3 | RNA | GGCCGAGGTCGACTACCGGCNRG | GGC--tcGcgGACTACCGGCTGG | + | 4 | 2 | 5 | 0.00 | 2.50 |
| Chr2:58693796-58693818 | 58693803 | 5 | RNA | GGCCGAGGTCGACTACCGGCNRG | GG--GAGaTgaACTtCCGGaGGG | - | 5 | 2 | 5 | 0.00 | 2.50 |
| Chr8:25629545-25629567 | 25629562 | 3 | RNA | GGCCGAGGTCGACTACCGGCNRG | G-aCGgGGTCGACaACgaGCGGG | + | 5 | 1 | 5 | 0.00 | 2.50 |
| Chr7:146414653-146414675 | 146414670 | 3 | RNA | GGCCGAGGTCGACTACCGGCNRG | GGgaGAGaTCGACTtC--GgTGG | + | 5 | 2 | 5 | 0.00 | 2.50 |
| Chr3:138783219-138783241 | 138783236 | 3 | RNA | GGCCGAGGTCGACTACCGGCNRG | GGC--tcGcgGACTACCGGtTGG | + | 5 | 2 | 5 | 0.00 | 2.50 |
| Chr3:128477406-128477428 | 128477413 | 5 | RNA | GGCCGAGGTCGACTACCGGCNRG | tGC--AGGcCGACcAaCaGCTAG | - | 5 | 2 | 5 | 0.00 | 2.50 |
| Chr5:165674116-165674138 | 165674123 | 5 | RNA | GGCCGAGGTCGACTACCGGCNRG | G-aCGgGGTCGACgACgaGCGGG | - | 5 | 1 | 5 | 0.00 | 2.50 |
| Chr4:221870198-221870220 | 221870215 | 3 | RNA | GGCCGAGGTCGACTACCGGCNRG | GGCgGAGGTCGACgACa--aCGG | + | 4 | 2 | 5 | 0.00 | 2.50 |
| Chr1:151859796-151859818 | 151859813 | 3 | RNA | GGCCGAGGTCGACTACCGGCNRG | GGgCcAcGTCGACT--CaaCCAG | + | 5 | 2 | 5 | 0.00 | 2.50 |
| Chr1:139517907-139517929 | 139517924 | 3 | RNA | GGCCGAGGTCGACTACCGGCNRG | GG--GtGtTCGACaACaaGCAAG | + | 5 | 2 | 5 | 0.00 | 2.50 |
| Chr8:171457896-171457918 | 171457903 | 5 | RNA | GGCCGAGGTCGACTACCGGCNRG | GGCgGAGGTCG--TtCgGcaGAG | - | 5 | 2 | 5 | 0.00 | 2.50 |
| Chr2:131249967-131249989 | 131249974 | 5 | DNA | GGCCGAGGTCGACTA--CCGGCNRG | cGCaaAGGcCGACcAATCCGGCAGG | - | 5 | 2 | 5 | 0.00 | 2.50 |
| Chr5:77159066-77159088 | 77159083 | 3 | RNA | GGCCGAGGTCGACTACCGGCNRG | G-aCGgGGTCGACaACgaGCGGG | + | 5 | 1 | 5 | 0.00 | 2.50 |
| Chr10:266540-266562 | 266557 | 3 | DNA | GGC-CGAGGTCGACTACCGGCNRG | cGCTCGAGGTtGgCcACaGGCGAG | + | 5 | 1 | 5 | 0.00 | 2.50 |
| Chr7:43887934-43887956 | 43887941 | 5 | RNA | GGCCGAGGTCGACTACCGGCNRG | G-aCGgGGTCGACaACgaGCGGG | - | 5 | 1 | 5 | 0.00 | 2.50 |
| Chr2:88096193-88096215 | 88096200 | 5 | X | GGCCGAGGTCGACTACCGGCNRG | GGaaGAGGTgGcCTAgCGGCTGG | - | 5 | 0 | 5 | 0.00 | 2.50 |
| Chr6:19224183-19224205 | 19224190 | 5 | RNA | GGCCGAGGTCGACTACCGGCNRG | GGC--ttGcgGACTACCGGCTGG | - | 4 | 2 | 5 | 0.00 | 2.50 |
| Chr3:15524289-15524311 | 15524296 | 5 | RNA | GGCCGAGGTCGACTACCGGCNRG | GcCaGAGGcCGACTt--GGaGGG | - | 5 | 2 | 5 | 0.00 | 2.50 |
| Chr2:28078546-28078568 | 28078563 | 3 | RNA | GGCCGAGGTCGACTACCGGCNRG | tGCgGtGGTCG--TAggGGCGGG | + | 5 | 2 | 5 | 0.00 | 2.50 |
| Chr7:79915664-79915686 | 79915671 | 5 | RNA | GGCCGAGGTCGACTACCGGCNRG | GGCacAGG-CGACTgCtGtCCGG | - | 5 | 1 | 5 | 0.00 | 2.50 |
| Chr7:74928865-74928887 | 74928872 | 5 | RNA | GGCCGAGGTCGACTACCGGCNRG | GcCgGAGcTCtACTgCCG-CCGG | - | 5 | 1 | 5 | 0.00 | 2.50 |
| Chr8:170611572-170611594 | 170611589 | 3 | RNA | GGCCGAGGTCGACTACCGGCNRG | GGCCGAGGgtGtCT-CgGGCTGG | + | 4 | 1 | 5 | 0.00 | 2.50 |
| Chr7:163533997-163534019 | 163534014 | 3 | RNA | GGCCGAGGTCGACTACCGGCNRG | GGCCcAG--CGACcgCgaGCAGG | + | 5 | 2 | 5 | 0.00 | 2.50 |
| Chr7:119842350-119842372 | 119842367 | 3 | DNA | GGCCGAGGTCGACTACCG-GCNRG | GGgCGAGcTCGACcAtgGAGCAGG | + | 5 | 1 | 5 | 0.00 | 2.50 |
| Chr2:66148672-66148694 | 66148689 | 3 | RNA | GGCCGAGGTCGACTACCGGCNRG | GGCCcA--TCGgtcACgGGCCAG | + | 5 | 2 | 5 | 0.00 | 2.50 |
| Chr2:22445536-22445558 | 22445543 | 5 | RNA | GGCCGAGGTCGACTACCGGCNRG | G-CCtcGGgCGAgTACtGGCAGG | - | 5 | 1 | 5 | 0.00 | 2.50 |
| Chr4:53984293-53984315 | 53984310 | 3 | RNA | GGCCGAGGTCGACTACCGGCNRG | GGC-GAGGTCGAgcgCCaGCGGG | + | 4 | 1 | 5 | 0.00 | 2.50 |
| Chr4:12529706-12529728 | 12529723 | 3 | DNA | GGC--CGAGGTCGACTACCGGCNRG | GGCCTCGgGGgCtACacCCGGCGGG | + | 5 | 2 | 5 | 0.00 | 2.50 |
| Chr9:36447980-36448002 | 36447997 | 3 | RNA | GGCCGAGGTCGACTACCGGCNRG | GGtCtAGGTCttC-ACCGGaTGG | + | 5 | 1 | 5 | 0.00 | 2.50 |
| Chr8:7139077-7139099 | 7139084 | 5 | RNA | GGCCGAGGTCGACTACCGGCNRG | GtCCG--GTgGtgTACCGGaCAG | - | 5 | 2 | 5 | 0.00 | 2.50 |
| Chr6:45157195-45157217 | 45157212 | 3 | RNA | GGCCGAGGTCGACTACCGGCNRG | GaCCaAaGTCaACTACt--CAGG | + | 5 | 2 | 5 | 0.00 | 2.50 |
| Chr7:106275519-106275541 | 106275526 | 5 | RNA | GGCCGAGGTCGACTACCGGCNRG | cGCCaAGcTCGA-TACCtGgTGG | - | 5 | 1 | 5 | 0.00 | 2.50 |
| Chr5:136248185-136248207 | 136248192 | 5 | RNA | GGCCGAGGTCGACTACCGGCNRG | GGCgctaaTCGACT-CCGGCGAG | - | 5 | 1 | 5 | 0.00 | 2.50 |
| Chr4:160494645-160494667 | 160494652 | 5 | DNA | GGCCGAGGTCGACTA--CCGGCNRG | ctCCaAGGcCGACcAATCCGGCAGG | - | 5 | 2 | 5 | 0.00 | 2.50 |
| Chr4:156124844-156124866 | 156124851 | 5 | RNA | GGCCGAGGTCGACTACCGGCNRG | GGatGAGcTCGAC--CCGagGAG | - | 5 | 2 | 5 | 0.00 | 2.50 |
| Chr10:36509790-36509812 | 36509797 | 5 | RNA | GGCCGAGGTCGACTACCGGCNRG | GGC-GAGGcCGgCcACCaGgCGG | - | 5 | 1 | 5 | 0.00 | 2.50 |
| Chr4:107995573-107995595 | 107995580 | 5 | RNA | GGCCGAGGTCGACTACCGGCNRG | GGC-GAGGTCGACgACgaGaTAG | - | 4 | 1 | 5 | 0.00 | 2.50 |
| Chr5:36540877-36540899 | 36540894 | 3 | RNA | GGCCGAGGTCGACTACCGGCNRG | tG--GAGGagGACgACCtGCTGG | + | 5 | 2 | 5 | 0.00 | 2.50 |
| Chr6:47196181-47196203 | 47196198 | 3 | RNA | GGCCGAGGTCGACTACCGGCNRG | GGCCaAttTCGgCTtC--GCCAG | + | 5 | 2 | 5 | 0.00 | 2.50 |
| Chr7:77796091-77796113 | 77796108 | 3 | DNA | G-GCCGAGGTCGACTACCGGCNRG | GCGCCtcGGgCGAgTACtGGCAGG | + | 5 | 1 | 5 | 0.00 | 2.50 |
| Chr3:96386434-96386456 | 96386451 | 3 | RNA | GGCCGAGGTCGACTACCGGCNRG | GGtCGAGGTCacC--CCGGgTAG | + | 4 | 2 | 5 | 0.00 | 2.50 |
| Chr4:67320914-67320936 | 67320921 | 5 | RNA | GGCCGAGGTCGACTACCGGCNRG | GGCCGgGcTaGgCTg--GGCTAG | - | 5 | 2 | 5 | 0.00 | 2.50 |
| Chr1:109314742-109314764 | 109314749 | 5 | RNA | GGCCGAGGTCGACTACCGGCNRG | GGCCGAGGatGcCT-CgGGCTGG | - | 4 | 1 | 5 | 0.00 | 2.50 |
| Chr8:54564954-54564976 | 54564971 | 3 | DNA | GGCC-GAGGTCGACTACCGGCNRG | GaCaTGAGGTtGACgACCaGCGGG | + | 5 | 1 | 5 | 0.00 | 2.50 |
| Chr8:127697101-127697123 | 127697118 | 3 | RNA | GGCCGAGGTCGACTACCGGCNRG | G-aCGgGGTCGACaACgaGCGGG | + | 5 | 1 | 5 | 0.00 | 2.50 |
| Chr5:192640023-192640045 | 192640030 | 5 | RNA | GGCCGAGGTCGACTACCGGCNRG | aGC--tcGcgGACTACCGGCTGG | - | 5 | 2 | 5 | 0.00 | 2.50 |
| Chr4:93186487-93186509 | 93186494 | 5 | RNA | GGCCGAGGTCGACTACCGGCNRG | aGCCGcGGTgGcCgA--GGCTAG | - | 5 | 2 | 5 | 0.00 | 2.50 |
| Chr8:65248729-65248751 | 65248746 | 3 | RNA | GGCCGAGGTCGACTACCGGCNRG | GGaCGAGaTtG--TttCGGCCGG | + | 5 | 2 | 5 | 0.00 | 2.50 |
| Chr7:68428927-68428949 | 68428934 | 5 | RNA | GGCCGAGGTCGACTACCGGCNRG | GG--GAGcTCGACgAgCtGgAGG | - | 5 | 2 | 5 | 0.00 | 2.50 |
| Chr2:118505663-118505685 | 118505680 | 3 | RNA | GGCCGAGGTCGACTACCGGCNRG | GGggGAGGTgG--TAgaGGCTGG | + | 5 | 2 | 5 | 0.00 | 2.50 |
| Chr8:5110933-5110955 | 5110940 | 5 | DNA | GGCCGAGGTCGACTA--CCGGCNRG | ctCCtAGGcCGACcAATCCGGCAGG | - | 5 | 2 | 5 | 0.00 | 2.50 |
| Chr10:48124588-48124610 | 48124605 | 3 | DNA | GGC--CGAGGTCGACTACCGGCNRG | GGCCTCGgGGgCtACacCCGGCGGG | + | 5 | 2 | 5 | 0.00 | 2.50 |
| Chr5:158087904-158087926 | 158087911 | 5 | RNA | GGCCGAGGTCGACTACCGGCNRG | GaCgagGGgCGACT-CCGGCGAG | - | 5 | 1 | 5 | 0.00 | 2.50 |
| Chr3:44342876-44342898 | 44342883 | 5 | RNA | GGCCGAGGTCGACTACCGGCNRG | GGgCGccGT-GACTgCtGGCCGG | - | 5 | 1 | 5 | 0.00 | 2.50 |
| Chr3:31201635-31201657 | 31201652 | 3 | RNA | GGCCGAGGTCGACTACCGGCNRG | tGCCcAGG--GACgAtaGGCAAG | + | 5 | 2 | 5 | 0.00 | 2.50 |
| Chr1:15490356-15490378 | 15490373 | 3 | RNA | GGCCGAGGTCGACTACCGGCNRG | GatCG-GGTCGACgACgaGCGGG | + | 5 | 1 | 5 | 0.00 | 2.50 |
| Chr10:72899570-72899592 | 72899587 | 3 | RNA | GGCCGAGGTCGACTACCGGCNRG | GcCCGAGaTCGACctCa--CCGG | + | 5 | 2 | 5 | 0.00 | 2.50 |
| Chr1:18004391-18004413 | 18004398 | 5 | RNA | GGCCGAGGTCGACTACCGGCNRG | GG--GAtaTCGAgTACCaaCAGG | - | 5 | 2 | 5 | 0.00 | 2.50 |
| Chr7:153162134-153162156 | 153162141 | 5 | DNA | GGCCGAGGT--CGACTACCGGCNRG | aGaCGAGGTTCCGACcAagGGCAGG | - | 5 | 2 | 5 | 0.00 | 2.50 |
| Chr5:19559432-19559454 | 19559438 | 4 | RNA | GGCCGAGGTCGACTACCGGCNRG | GcCaGtGGaCGgC--CCGGCTAG | - | 5 | 2 | 0 | 4.96 | 2.48 |
| Chr2:98913861-98913883 | 98913878 | 3 | DNA | GGCCGAG--GTCGACTACCGGCNRG | GGCCGAGCAGagGAactCCGGCCGG | + | 5 | 2 | 0 | 4.96 | 2.48 |
| Chr5:195931969-195931991 | 195931975 | 4 | RNA | GGCCGAGGTCGACTACCGGCNRG | GGC--tcGTgGACTACCGcCTGG | - | 4 | 2 | 0 | 4.96 | 2.48 |
| Chr6:128822413-128822435 | 128822418 | 3 | RNA | GGCCGAGGTCGACTACCGGCNRG | GaCaG-GGTCGACaACgaGCGGG | - | 5 | 1 | 0 | 4.96 | 2.48 |
| Chr1:124111388-124111410 | 124111404 | 4 | X | GGCCGAGGTCGACTACCGGCNRG | GGaaGAGGTgGcCTAgCGGCTGG | + | 5 | 0 | 0 | 4.96 | 2.48 |
| Chr1:109940088-109940110 | 109940094 | 4 | RNA | GGCCGAGGTCGACTACCGGCNRG | GGaCG--GTCGACacttGGCAGG | - | 5 | 2 | 0 | 4.96 | 2.48 |
| Chr6:164441173-164441195 | 164441188 | 5 | RNA | GGCCGAGGTCGACTACCGGCNRG | GcCaGAGccCGAC--CCGtCAGG | + | 5 | 2 | 0 | 4.96 | 2.48 |
| Chr4:8755605-8755627 | 8755620 | 5 | DNA | GGCCGAGGT--CGACTACCGGCNRG | GGgCGAGGcGGaGAtTgCCGGCTGG | + | 5 | 2 | 0 | 4.96 | 2.48 |
| Chr3:168788394-168788416 | 168788400 | 4 | RNA | GGCCGAGGTCGACTACCGGCNRG | G-aCGgGGTCGACTACgaGCGGG | - | 4 | 1 | 0 | 4.96 | 2.48 |
| Chr4:199315961-199315983 | 199315976 | 5 | RNA | GGCCGAGGTCGACTACCGGCNRG | GcaCGgcGTCGACTgC-GGCGAG | + | 5 | 1 | 0 | 4.96 | 2.48 |
| Chr9:133783471-133783493 | 133783475 | 2 | RNA | GGCCGAGGTCGACTACCGGCNRG | tGCaGtGGTCGtCTgC--GCCAG | - | 5 | 2 | 0 | 4.96 | 2.48 |
| Chr5:194108057-194108079 | 194108073 | 4 | RNA | GGCCGAGGTCGACTACCGGCNRG | cGCCG-GcTacACTAaCGGCGGG | + | 5 | 1 | 0 | 4.96 | 2.48 |
| Chr10:86070399-86070421 | 86070416 | 3 | RNA | GGCCGAGGTCGACTACCGGCNRG | GGC--ttGTgGAaTACCGGCTGG | + | 4 | 2 | 0 | 4.96 | 2.48 |
| Chr4:187534235-187534257 | 187534251 | 4 | RNA | GGCCGAGGTCGACTACCGGCNRG | t--CGgGGaCGtCTtCCGGCTGG | + | 5 | 2 | 0 | 4.96 | 2.48 |
| Chr2:37800422-37800444 | 37800427 | 3 | RNA | GGCCGAGGTCGACTACCGGCNRG | G-aCGgGGTCGACaACgaGCGGG | - | 5 | 1 | 0 | 4.96 | 2.48 |
| Chr4:125456219-125456241 | 125456223 | 2 | RNA | GGCCGAGGTCGACTACCGGCNRG | GtCgGAGGTaaACT-CCaGCCAG | - | 5 | 1 | 0 | 4.96 | 2.48 |
| Chr1:241548677-241548699 | 241548693 | 4 | RNA | GGCCGAGGTCGACTACCGGCNRG | tGCaGAGcTCGgCTA--GGCTGG | + | 4 | 2 | 0 | 4.96 | 2.48 |
| Chr8:31049932-31049954 | 31049938 | 4 | RNA | GGCCGAGGTCGACTACCGGCNRG | GcCCGAGGgCaACcACC--gTGG | - | 5 | 2 | 0 | 4.96 | 2.48 |
| Chr7:1323940-1323962 | 1323955 | 5 | DNA | GGCCGAGGTCGACTACC--GGCNRG | GGCgGAGGTCGtCggCaGAGGCCAG | + | 5 | 2 | 0 | 4.96 | 2.48 |
| Chr5:92060275-92060297 | 92060293 | 2 | RNA | GGCCGAGGTCGACTACCGGCNRG | GttCGAGcTCG--TACtGGaCAG | + | 5 | 2 | 0 | 4.96 | 2.48 |

| | | | | | | | | | | |
|---|---|---|---|---|---|---|---|---|---|---|
| Chr9:104833430-104833452 | 104833436 | 4 | RNA | GGCCGAGGTCGACTACCGGCNRG | aGCCG--GcCGACaACCaGaGAG | - | 5 | 2 | 0 | 4.96 | 2.48 |
| Chr10:78339100-78339122 | 78339104 | 2 | RNA | GGCCGAGGTCGACTACCGGCNRG | GcCgcAGGTgG--TgCCGGCAAG | - | 5 | 2 | 0 | 4.96 | 2.48 |
| Chr3:67721785-67721807 | 67721790 | 3 | RNA | GGCCGAGGTCGACTACCGGCNRG | GGCCGAcGTCGACcACt--CGAG | - | 3 | 2 | 0 | 4.96 | 2.48 |
| Chr1:179482810-179482832 | 179482827 | 3 | DNA | GGCCGAGGTCGACTAC--CGGCNRG | GGaCGAGcTCGcCggCGGCGGCTGG | + | 5 | 2 | 0 | 4.96 | 2.48 |
| Chr5:155563529-155563551 | 155563547 | 2 | RNA | GGCCGAGGTCGACTACCGGCNRG | GGaCcAGGTCG--TcCCaGtTGG | + | 5 | 2 | 0 | 4.96 | 2.48 |
| Chr2:109941218-109941240 | 109941224 | 4 | RNA | GGCCGAGGTCGACTACCGGCNRG | cG--GAGGgaGAaTACCcGCTGG | - | 5 | 2 | 0 | 4.96 | 2.48 |
| Chr4:70664764-70664786 | 70664770 | 4 | RNA | GGCCGAGGTCGACTACCGGCNRG | GGaCcAGGTCG--TcCCaGtTGG | - | 5 | 2 | 0 | 4.96 | 2.48 |
| Chr7:26041820-26041842 | 26041838 | 2 | RNA | GGCCGAGGTCGACTACCGGCNRG | GGCgGAGGTCG--TtCgGcaGAG | + | 5 | 2 | 0 | 4.96 | 2.48 |
| Chr9:145402222-145402244 | 145402227 | 3 | RNA | GGCCGAGGTCGACTACCGGCNRG | GcgCGtGGTaGcC--CCGGCGAG | - | 5 | 2 | 0 | 4.96 | 2.48 |
| Chr1:167309177-167309199 | 167309192 | 5 | RNA | GGCCGAGGTCGACTACCGGCNRG | acCCGAtGTCG--TACgaGCTAG | + | 5 | 2 | 0 | 4.96 | 2.48 |
| Chr5:193726588-193726610 | 193726595 | 5 | RNA | GGCCGAGGTCGACTACCGGCNRG | GGCCaAGGTCGg-TACgtGCAAG | - | 4 | 1 | 0 | 4.96 | 2.48 |
| Chr1:147651919-147651941 | 147651925 | 4 | DNA | GGCCGAGGTCGACTACC--GGCNRG | GGCgGAGGTCGtCTgCaGAGGCCAG | - | 4 | 2 | 0 | 4.96 | 2.48 |
| Chr4:239022854-239022876 | 239022861 | 5 | RNA | GGCCGAGGTCGACTACCGGCNRG | GcCgGAGccCGAC--CCGtCAAG | - | 5 | 2 | 0 | 4.96 | 2.48 |
| Chr4:206793171-206793193 | 206793186 | 5 | RNA | GGCCGAGGTCGACTACCGGCNRG | GGCCc--GcCaACTtCCGGgCAG | + | 5 | 2 | 0 | 4.96 | 2.48 |
| Chr2:162983071-162983093 | 162983076 | 3 | RNA | GGCCGAGGTCGACTACCGGCNRG | GGtCGAGGTC-ACcAtaGaCAGG | - | 5 | 1 | 0 | 4.96 | 2.48 |
| Chr8:96802599-96802621 | 96802615 | 4 | RNA | GGCCGAGGTCGACTACCGGCNRG | GGaCGAG-TCGACgACgGGCGGG | + | 3 | 1 | 0 | 4.96 | 2.48 |
| Chr2:134103104-134103126 | 134103109 | 3 | RNA | GGCCGAGGTCGACTACCGGCNRG | GGgCGAGGgCGAagAt-GGCGGG | - | 5 | 1 | 0 | 4.96 | 2.48 |
| Chr6:65982269-65982291 | 65982284 | 5 | RNA | GGCCGAGGTCGACTACCGGCNRG | aGC--AGGTCGACgtaaGGCCGG | + | 5 | 2 | 0 | 4.96 | 2.48 |
| Chr6:108385885-108385907 | 108385903 | 2 | RNA | GGCCGAGGTCGACTACCGGCNRG | GGCgGAG-TCcACcACCcaCTAG | + | 5 | 1 | 0 | 4.96 | 2.48 |
| Chr10:71156174-71156196 | 71156181 | 5 | RNA | GGCCGAGGTCGACTACCGGCNRG | GGCCGAaaTCccCTcCC--CTGG | - | 5 | 2 | 0 | 4.96 | 2.48 |
| Chr4:107995249-107995271 | 107995254 | 3 | RNA | GGCCGAGGTCGACTACCGGCNRG | GGC-GAGcTgGACTgCtGGCCAG | - | 4 | 1 | 0 | 4.96 | 2.48 |
| Chr2:49221166-49221188 | 49221183 | 3 | DNA | GGC-CGAGGTCGACTACCGGCNRG | GcCTCGAGGaCGtCTtCCGGCTGG | + | 4 | 1 | 0 | 4.96 | 2.48 |
| Chr9:16620561-16620583 | 16620578 | 3 | RNA | GGCCGAGGTCGACTACCGGCNRG | G--CtAGGTCGcggACCGtCCGG | + | 5 | 2 | 0 | 4.96 | 2.48 |
| Chr5:96941919-96941941 | 96941935 | 4 | RNA | GGCCGAGGTCGACTACCGGCNRG | G-aCGgGGTCGACaACgaGCGGG | + | 5 | 1 | 0 | 4.96 | 2.48 |
| Chr6:145848412-145848434 | 145848418 | 4 | RNA | GGCCGAGGTCGACTACCGGCNRG | GGCCGAGtcCG-CgACCaGaTAG | - | 5 | 1 | 0 | 4.96 | 2.48 |
| Chr7:133210223-133210245 | 133210241 | 2 | RNA | GGCCGAGGTCGACTACCGGCNRG | cGgCGAGG--GAggtCCGGCGAG | + | 5 | 2 | 0 | 4.96 | 2.48 |
| Chr4:142823192-142823214 | 142823208 | 4 | X | GGCCGAGGTCGACTACCGGCNRG | GGCgGAGcTCGAgcACgGGCGGG | + | 5 | 0 | 0 | 4.96 | 2.48 |
| Chr8:22412025-22412047 | 22412041 | 4 | RNA | GGCCGAGGTCGACTACCGGCNRG | GGgCG-GGcCaACccCCGGCCGG | + | 5 | 1 | 0 | 4.96 | 2.48 |
| Chr4:208161430-208161452 | 208161436 | 4 | RNA | GGCCGAGGTCGACTACCGGCNRG | GGagGAGGaCGACT--CatCAAG | - | 5 | 2 | 0 | 4.96 | 2.48 |
| Chr6:62578280-62578302 | 62578298 | 2 | RNA | GGCCGAGGTCGACTACCGGCNRG | aGC--AGaTCGACTtaaGGCCGG | + | 5 | 2 | 0 | 4.96 | 2.48 |
| Chr3:83892362-83892384 | 83892377 | 5 | RNA | GGCCGAGGTCGACTACCGGCNRG | GG--GAcaTCataTACCGGCTAG | + | 5 | 2 | 0 | 4.96 | 2.48 |
| Chr10:99222241-99222263 | 99222246 | 3 | RNA | GGCCGAGGTCGACTACCGGCNRG | GGCatAGGat-ACTACaGGCAGG | - | 5 | 1 | 0 | 4.96 | 2.48 |
| Chr2:75002590-75002612 | 75002596 | 4 | DNA | GGCCGAG-GTCGACTACCGGCNRG | aGCtGAGCGgCGgCaACCGGCGAG | - | 5 | 1 | 0 | 4.96 | 2.48 |
| Chr7:43788995-43789017 | 43789012 | 3 | RNA | GGCCGAGGTCGACTACCGGCNRG | tGCCGcGcTCGgCT--CGGgCGG | + | 5 | 2 | 0 | 4.96 | 2.48 |
| Chr2:111869271-111869293 | 111869289 | 2 | RNA | GGCCGAGGTCGACTACCGGCNRG | GGCCGccGT-GAgTACaGGtGAG | + | 5 | 1 | 0 | 4.96 | 2.48 |
| Chr2:216239561-216239583 | 216239567 | 4 | RNA | GGCCGAGGTCGACTACCGGCNRG | GGCCG-GGcCGtCcACtGGCGGG | - | 4 | 1 | 0 | 4.96 | 2.48 |
| Chr1:118097494-118097516 | 118097510 | 4 | RNA | GGCCGAGGTCGACTACCGGCNRG | GGtCGtGcTCGAC-ACgaGCTGG | + | 5 | 1 | 0 | 4.96 | 2.48 |
| Chr2:182693714-182693736 | 182693730 | 4 | DNA | GGCCGAGGTCGACTACC--GGCNRG | aGCCGAcGgCGACTgCaAGGGCGGG | + | 5 | 2 | 0 | 4.96 | 2.48 |
| Chr4:18892791-18892813 | 18892796 | 3 | RNA | GGCCGAGGTCGACTACCGGCNRG | G-CCGttaTCcACcACCGGCAGG | - | 5 | 1 | 0 | 4.96 | 2.48 |
| Chr8:14811737-14811759 | 14811753 | 4 | RNA | GGCCGAGGTCGACTACCGGCNRG | G-CCGAaGTacACcACCtGCTGG | + | 5 | 1 | 0 | 4.96 | 2.48 |
| Chr8:34557825-34557847 | 34557842 | 3 | RNA | GGCCGAGGTCGACTACCGGCNRG | cGCCGccGTC--CTcCCGtCAAG | + | 5 | 2 | 0 | 4.96 | 2.48 |
| Chr2:1399700-1399722 | 1399716 | 4 | DNA | GGCCGAGGTCG--ACTACCGGCNRG | GcCCGAGGTCGCTcgTACCctCGGG | + | 5 | 2 | 0 | 4.96 | 2.48 |
| Chr7:134261506-134261528 | 134261522 | 4 | RNA | GGCCGAGGTCGACTACCGGCNRG | GGCgGAGa-CatCTtCCGGCTGG | + | 5 | 1 | 0 | 4.96 | 2.48 |
| Chr10:18704782-18704804 | 18704789 | 5 | RNA | GGCCGAGGTCGACTACCGGCNRG | GctCGAGGTgcACTAC-GGgTAG | - | 5 | 1 | 0 | 4.96 | 2.48 |
| Chr10:89301823-89301845 | 89301827 | 2 | RNA | GGCCGAGGTCGACTACCGGCNRG | GGagGAGGagGACT--CGGaCGG | - | 5 | 2 | 0 | 4.96 | 2.48 |
| Chr8:42999715-42999737 | 42999721 | 4 | RNA | GGCCGAGGTCGACTACCGGCNRG | GGaCGAGG-CGAgaAgtGGCTGG | - | 5 | 1 | 0 | 4.96 | 2.48 |
| Chr8:73523361-73523383 | 73523378 | 3 | RNA | GGCCGAGGTCGACTACCGGCNRG | tGCCGtGGTCGcCg--CGGCGGG | + | 4 | 2 | 0 | 4.96 | 2.48 |
| Chr2:6666036-6666058 | 6666052 | 4 | RNA | GGCCGAGGTCGACTACCGGCNRG | cGaCGAcGTCcACT--CGGgAAG | + | 5 | 2 | 0 | 4.96 | 2.48 |
| Chr9:117044286-117044308 | 117044291 | 3 | RNA | GGCCGAGGTCGACTACCGGCNRG | GcCCGAGccCGAgT--CGGtGGG | - | 5 | 2 | 0 | 4.96 | 2.48 |
| Chr2:98435223-98435245 | 98435240 | 3 | DNA | GGCCGAGGTCGACTACC--GGCNRG | GcCCGAGcaCaACcACCATGGCCAG | + | 5 | 2 | 0 | 4.96 | 2.48 |
| Chr2:103423915-103423937 | 103423933 | 2 | RNA | GGCCGAGGTCGACTACCGGCNRG | GGCaGAaG--GACTcCgGGCCAG | + | 4 | 2 | 0 | 4.96 | 2.48 |
| Chr3:131973043-131973065 | 131973058 | 5 | RNA | GGCCGAGGTCGACTACCGGCNRG | GGgaGAaGTgGACgACCG-CTGG | + | 5 | 1 | 0 | 4.96 | 2.48 |
| Chr6:152415481-152415503 | 152415499 | 2 | RNA | GGCCGAGGTCGACTACCGGCNRG | GGatGAG--aGACaACtGGCGAG | + | 5 | 2 | 0 | 4.96 | 2.48 |
| Chr8:109840741-109840763 | 109840756 | 5 | RNA | GGCCGAGGTCGACTACCGGCNRG | GGtCG-GGcCGtCcACtGGCGGG | + | 5 | 1 | 0 | 4.96 | 2.48 |
| Chr6:11350926-11350948 | 11350930 | 2 | RNA | GGCCGAGGTCGACTACCGGCNRG | GGCCaAGGgCGcCT-CgGGCAGG | - | 4 | 1 | 0 | 4.96 | 2.48 |
| Chr4:65515622-65515644 | 65515637 | 5 | RNA | GGCCGAGGTCGACTACCGGCNRG | GGC--ttGcgGACTACCGGCTGG | + | 4 | 2 | 0 | 4.96 | 2.48 |
| Chr1:153168105-153168127 | 153168121 | 4 | RNA | GGCCGAGGTCGACTACCGGCNRG | GGgCG-GGcCaACccCCGGCCGG | + | 5 | 1 | 0 | 4.96 | 2.48 |
| Chr3:179124793-179124815 | 179124810 | 3 | RNA | GGCCGAGGTCGACTACCGGCNRG | GGC--tcGcgGACTACCGGCTGG | + | 4 | 2 | 0 | 4.96 | 2.48 |
| Chr1:304148669-304148691 | 304148684 | 5 | RNA | GGCCGAGGTCGACTACCGGCNRG | GtCttgGGT-GACcACCGGCGAG | + | 5 | 1 | 0 | 4.96 | 2.48 |
| Chr2:149089809-149089831 | 149089825 | 4 | RNA | GGCCGAGGTCGACTACCGGCNRG | GGC--AGGcCaACctCCGGCCGG | + | 4 | 2 | 0 | 4.96 | 2.48 |
| Chr8:74798064-74798086 | 74798081 | 3 | RNA | GGCCGAGGTCGACTACCGGCNRG | GGCtGgGGTaG--cACCGGgAGG | + | 5 | 2 | 0 | 4.96 | 2.48 |
| Chr8:70236156-70236178 | 70236172 | 4 | RNA | GGCCGAGGTCGACTACCGGCNRG | a-CCGAGGTCGAgctCgGGCGAG | + | 5 | 1 | 0 | 4.96 | 2.48 |
| Chr2:85574295-85574317 | 85574310 | 5 | RNA | GGCCGAGGTCGACTACCGGCNRG | G-aCGAGGTCGACgACgaGCGGG | + | 4 | 1 | 0 | 4.96 | 2.48 |
| Chr7:34662937-34662959 | 34662942 | 3 | RNA | GGCCGAGGTCGACTACCGGCNRG | GGC--ttGcaGACTACCGGCTGG | - | 4 | 2 | 0 | 4.96 | 2.48 |
| Chr4:106462938-106462960 | 106462954 | 4 | DNA | GGCCGAGGTCG-ACTACCGGCNRG | aGCCtAGGTCGCAgaACaGGCTAG | + | 5 | 1 | 0 | 4.96 | 2.48 |
| Chr1:53396316-53396338 | 53396322 | 4 | RNA | GGCCGAGGTCGACTACCGGCNRG | GcCCGAGtcCGA-gAgCGGCTGG | - | 5 | 1 | 0 | 4.96 | 2.48 |
| Chr7:180147340-180147362 | 180147357 | 3 | RNA | GGCCGAGGTCGACTACCGGCNRG | G-CCGAGGagGACgACCGGgTGG | + | 4 | 1 | 0 | 4.96 | 2.48 |
| Chr4:145616709-145616731 | 145616724 | 5 | RNA | GGCCGAGGTCGACTACCGGCNRG | GGgCGAGGgCG--gACCtGgAGG | + | 5 | 2 | 0 | 4.96 | 2.48 |
| Chr5:116752231-116752253 | 116752236 | 3 | RNA | GGCCGAGGTCGACTACCGGCNRG | GcCCGAGGgCaACcACC--gTGG | - | 5 | 2 | 0 | 4.96 | 2.48 |
| Chr2:63483043-63483065 | 63483049 | 4 | RNA | GGCCGAGGTCGACTACCGGCNRG | GtgCtcGG--GACaACCGGCAGG | - | 5 | 2 | 0 | 4.96 | 2.48 |
| Chr9:143141983-143142005 | 143141988 | 3 | RNA | GGCCGAGGTCGACTACCGGCNRG | GGCacAGGTtGAgg--CGGCCGG | - | 5 | 2 | 0 | 4.96 | 2.48 |
| Chr5:95635826-95635848 | 95635843 | 3 | RNA | GGCCGAGGTCGACTACCGGCNRG | GGCCt-GGTCcACgtgCGGCTGG | + | 5 | 1 | 0 | 4.96 | 2.48 |
| Chr1:205664183-205664205 | 205664200 | 3 | DNA | GGC-CGAGGTCGACTACCGGCNRG | GcCTCGAGGaCGtCTtCCGGCTGG | + | 4 | 1 | 0 | 4.96 | 2.48 |
| Chr3:16510324-16510346 | 16510342 | 2 | RNA | GGCCGAGGTCGACTACCGGCNRG | GGC-GAGGTCctCctCCtGCCAG | + | 5 | 1 | 0 | 4.96 | 2.48 |
| Chr8:110049406-110049428 | 110049410 | 2 | RNA | GGCCGAGGTCGACTACCGGCNRG | tGaaGAGaTCGACTACaG-CAGG | - | 5 | 1 | 0 | 4.96 | 2.48 |
| Chr7:124425503-124425525 | 124425510 | 5 | RNA | GGCCGAGGTCGACTACCGGCNRG | tGCgGgGcTCGACTAC-GGaCGG | - | 5 | 1 | 0 | 4.96 | 2.48 |
| Chr6:45977536-45977558 | 45977541 | 3 | RNA | GGCCGAGGTCGACTACCGGCNRG | aGCaGAG--CGAgatCCGGCCAG | - | 5 | 2 | 0 | 4.96 | 2.48 |
| Chr5:206529952-206529974 | 206529968 | 4 | RNA | GGCCGAGGTCGACTACCGGCNRG | GcgCGtGGTaGcC--CCGGCGAG | + | 5 | 2 | 0 | 4.96 | 2.48 |
| Chr1:94666673-94666695 | 94666678 | 3 | RNA | GGCCGAGGTCGACTACCGGCNRG | GGC--AGGcCGtgcACCGGCCGG | - | 4 | 2 | 0 | 4.96 | 2.48 |
| Chr6:127441781-127441803 | 127441796 | 5 | RNA | GGCCGAGGTCGACTACCGGCNRG | GGCaGAGG--GACaACtGtCGGG | + | 4 | 2 | 0 | 4.96 | 2.48 |
| Chr2:164236196-164236218 | 164236211 | 5 | RNA | GGCCGAGGTCGACTACCGGCNRG | GaCCG-GGTCGtCcACtGGCGGG | + | 4 | 1 | 0 | 4.96 | 2.48 |
| Chr1:149384055-149384077 | 149384062 | 5 | RNA | GGCCGAGGTCGACTACCGGCNRG | GGgCGAGGgCG--gACCtGgAGG | - | 5 | 2 | 0 | 4.96 | 2.48 |
| Chr1:137801723-137801745 | 137801738 | 5 | RNA | GGCCGAGGTCGACTACCGGCNRG | GtCCGgtGT--ACcACCGGaCAG | + | 5 | 2 | 0 | 4.96 | 2.48 |
| Chr8:151879708-151879730 | 151879726 | 2 | RNA | GGCCGAGGTCGACTACCGGCNRG | GG--GAGGagGtaTACgGGCAAG | + | 5 | 2 | 0 | 4.96 | 2.48 |
| Chr4:15127855-15127877 | 15127871 | 4 | RNA | GGCCGAGGTCGACTACCGGCNRG | tG--GAGGagGACgACCtGCTGG | + | 5 | 2 | 0 | 4.96 | 2.48 |
| Chr1:263810281-263810303 | 263810299 | 2 | RNA | GGCCGAGGTCGACTACCGGCNRG | GaCCGAGG-CGACgACaaGaAGG | + | 5 | 1 | 0 | 4.96 | 2.48 |
| Chr9:21156229-21156251 | 21156245 | 4 | RNA | GGCCGAGGTCGACTACCGGCNRG | G-aCGgGGTCGACaACgaGCGGG | + | 5 | 1 | 0 | 4.96 | 2.48 |
| Chr7:31825811-31825833 | 31825818 | 5 | RNA | GGCCGAGGTCGACTACCGGCNRG | GGatGgGGTCGgC-gCCGGCGGG | - | 5 | 1 | 0 | 4.96 | 2.48 |
| Chr7:165795458-165795480 | 165795476 | 2 | RNA | GGCCGAGGTCGACTACCGGCNRG | GGCgGtGG--GgCTgtCGGCGAG | + | 5 | 2 | 0 | 4.96 | 2.48 |
| Chr5:33545793-33545815 | 33545808 | 5 | RNA | GGCCGAGGTCGACTACCGGCNRG | tGgCt-cGTgGACTACCGGCTGG | + | 5 | 1 | 0 | 4.96 | 2.48 |
| Chr9:74121535-74121557 | 74121539 | 2 | RNA | GGCCGAGGTCGACTACCGGCNRG | GGCaGtGGT--ACTcCgGtCGAG | - | 5 | 2 | 0 | 4.96 | 2.48 |
| Chr2:159436251-159436273 | 159436258 | 5 | RNA | GGCCGAGGTCGACTACCGGCNRG | GGCCcAc--CGACcACCGtgGAG | - | 5 | 2 | 0 | 4.96 | 2.48 |
| Chr4:165980880-165980902 | 165980896 | 4 | RNA | GGCCGAGGTCGACTACCGGCNRG | G-aCGgGGTCGACgACgaGCGGG | + | 5 | 1 | 0 | 4.96 | 2.48 |
| Chr3:214260258-214260280 | 214260273 | 5 | RNA | GGCCGAGGTCGACTACCGGCNRG | GGC--ttGcgGACTACCGGCTGG | + | 4 | 2 | 0 | 4.96 | 2.48 |
| Chr2:121783603-121783625 | 121783608 | 3 | RNA | GGCCGAGGTCGACTACCGGCNRG | GaC--AGGcCGAgctCCGGCGAG | - | 5 | 2 | 0 | 4.96 | 2.48 |
| Chr3:214892996-214893018 | 214893002 | 4 | RNA | GGCCGAGGTCGACTACCGGCNRG | G-CCtcGGgCGAgTACtGGCAGG | - | 5 | 1 | 2 | 2.75 | 2.38 |
| Chr5:223273918-223273940 | 223273923 | 3 | RNA | GGCCGAGGTCGACTACCGGCNRG | GGC-GAGGTCGACcACaGGCAGG | - | 2 | 1 | 2 | 2.75 | 2.38 |
| Chr5:176274493-176274515 | 176274500 | 5 | RNA | GGCCGAGGTCGACTACCGGCNRG | GGCCGAGGgtGcCT-CgGGaTGG | - | 5 | 1 | 2 | 2.75 | 2.38 |
| Chr5:173848653-173848675 | 173848669 | 4 | RNA | GGCCGAGGTCGACTACCGGCNRG | G-CCGAaGTCcACTgCCtGCTGG | + | 4 | 1 | 3 | 1.65 | 2.33 |
| Chr2:179678271-179678293 | 179678288 | 3 | X | GGCCGAGGTCGACTACCGGCNRG | GGCacAGGcCGAgcACCGGCCGG | + | 5 | 0 | 3 | 1.65 | 2.33 |
| Chr4:121416807-121416829 | 121416812 | 3 | DNA | GGCCGAGGTCGACTACC--GGCNRG | GaCaGAGGaCGACgtCCACGGCTGG | - | 5 | 2 | 0 | 4.41 | 2.21 |
| Chr4:156821274-156821296 | 156821290 | 4 | RNA | GGCCGAGGTCGACTACCGGCNRG | G-aCGgGGTCGACgACgaGCGGG | + | 5 | 1 | 0 | 4.41 | 2.21 |
| Chr5:160510373-160510395 | 160510378 | 3 | RNA | GGCCGAGGTCGACTACCGGCNRG | G-CCGAaGTacACcACCtGCTGG | - | 5 | 1 | 0 | 4.41 | 2.21 |
| Chr2:120563297-120563319 | 120563315 | 2 | RNA | GGCCGAGGTCGACTACCGGCNRG | GGCCGAG--CGAtgcCCaGCCAG | + | 4 | 2 | 0 | 4.41 | 2.21 |
| Chr1:178319218-178319240 | 178319236 | 2 | RNA | GGCCGAGGTCGACTACCGGCNRG | a--CGgGGTCGACgACgaGCGGG | + | 5 | 2 | 0 | 4.41 | 2.21 |
| Chr7:72811777-72811799 | 72811793 | 4 | RNA | GGCCGAGGTCGACTACCGGCNRG | G-aCGgGGTCGACaACgaGCGGG | + | 5 | 1 | 0 | 4.41 | 2.21 |
| Chr1:151987562-151987584 | 151987568 | 4 | DNA | GGC--CGAGGTCGACTACCGGCNRG | GGCAAtGAaGTCtgCTAaCGGCTAG | - | 5 | 2 | 0 | 4.41 | 2.21 |
| Chr2:109649192-109649214 | 109649210 | 2 | RNA | GGCCGAGGTCGACTACCGGCNRG | GatCaAcGTCGACaA--GGCAAG | + | 5 | 2 | 0 | 4.41 | 2.21 |
| Chr9:90809908-90809930 | 90809913 | 3 | DNA | GGCCGAGGTCGACT--ACCGGCNRG | GGCtGtGtTtaACTCAACCGGCTAG | - | 5 | 2 | 0 | 4.41 | 2.21 |
| Chr2:11791465-11791487 | 11791483 | 2 | RNA | GGCCGAGGTCGACTACCGGCNRG | GttCGAGaT--ACTtCgGGCAAG | + | 5 | 2 | 0 | 4.41 | 2.21 |
| Chr5:66670790-66670812 | 66670808 | 2 | RNA | GGCCGAGGTCGACTACCGGCNRG | GGC--tcGcgGACTACCGaCTGG | + | 5 | 2 | 0 | 4.41 | 2.21 |
| Chr2:134228263-134228285 | 134228270 | 5 | RNA | GGCCGAGGTCGACTACCGGCNRG | GGgCtAtG-CGACTAtCGGaTGG | - | 5 | 1 | 0 | 4.41 | 2.21 |
| Chr4:84440416-84440438 | 84440433 | 3 | RNA | GGCCGAGGTCGACTACCGGCNRG | GGCCtAGG--GAgaAaCGaCTAG | + | 5 | 2 | 0 | 4.41 | 2.21 |
| Chr8:9283741-9283763 | 9283757 | 4 | RNA | GGCCGAGGTCGACTACCGGCNRG | GGaCagccTCGAC-ACCGGCGAG | + | 5 | 1 | 0 | 4.41 | 2.21 |
| Chr3:119452257-119452279 | 119452274 | 3 | X | GGCCGAGGTCGACTACCGGCNRG | GGCCGAGGTtGAgctCgGGCGAG | + | 5 | 0 | 0 | 4.41 | 2.21 |
| Chr5:125789930-125789952 | 125789945 | 5 | RNA | GGCCGAGGTCGACTACCGGCNRG | GGC--AGcTCGAggACgaGCTGG | + | 5 | 2 | 0 | 4.41 | 2.21 |
| Chr3:213544370-213544392 | 213544377 | 5 | RNA | GGCCGAGGTCGACTACCGGCNRG | G-CCtcGGgCGAgTACtGGCAGG | - | 5 | 1 | 0 | 4.41 | 2.21 |
| Chr8:106891427-106891449 | 106891434 | 5 | RNA | GGCCGAGGTCGACTACCGGCNRG | GGgCG-GGcCaACctCCGGCCGG | - | 5 | 1 | 0 | 4.41 | 2.21 |
| Chr3:57148199-57148221 | 57148203 | 2 | RNA | GGCCGAGGTCGACTACCGGCNRG | GGC--tcGcgGACTACCGGCTAG | - | 4 | 2 | 0 | 4.41 | 2.21 |
| Chr3:145244821-145244843 | 145244839 | 2 | RNA | GGCCGAGGTCGACTACCGGCNRG | GGCCGgGGTCGg-TcgaGGCGAG | + | 5 | 1 | 0 | 4.41 | 2.21 |
| Chr3:231991398-231991420 | 231991413 | 5 | RNA | GGCCGAGGTCGACTACCGGCNRG | GGC--tGGTCcAtTACatGCTGG | + | 5 | 2 | 0 | 4.41 | 2.21 |
| Chr4:116217396-116217418 | 116217403 | 5 | RNA | GGCCGAGGTCGACTACCGGCNRG | GGC-GtGGTCGAggACtaGCGGG | - | 5 | 1 | 0 | 4.41 | 2.21 |
| Chr3:64476367-64476389 | 64476383 | 4 | RNA | GGCCGAGGTCGACTACCGGCNRG | GGaCGAGccCGACT-CtGGtTGG | + | 5 | 1 | 0 | 4.41 | 2.21 |
| Chr2:92306997-92307019 | 92307015 | 2 | RNA | GGCCGAGGTCGACTACCGGCNRG | G-aCGAaGaCGACTcCCGGaGAG | + | 5 | 1 | 0 | 4.41 | 2.21 |
| Chr3:132432660-132432682 | 132432665 | 3 | RNA | GGCCGAGGTCGACTACCGGCNRG | cGCCGtGGTCGcCg--CGGCAGG | - | 4 | 2 | 0 | 4.41 | 2.21 |
| Chr4:41834984-41835006 | 41834990 | 4 | DNA | GGC-CGAGGTCGACTACCGGCNRG | GcCACGgGGaCGtCTtCCGGCTGG | - | 5 | 1 | 0 | 4.41 | 2.21 |

| | | | | | | | | | | | |
|---|---|---|---|---|---|---|---|---|---|---|---|
| Chr1:26437689-26437711 | 26437695 | 4 | RNA | GGCCGAGGTCGACTACCGGCNRG | GtCgaAGGgCG--TACCGGaCAG | - | 5 | 2 | 0 | 4.41 | 2.21 |
| Chr6:169692089-169692111 | 169692104 | 5 | RNA | GGCCGAGGTCGACTACCGGCNRG | GGCaG-GcTacACTACCaGCTAG | + | 5 | 1 | 0 | 4.41 | 2.21 |
| Chr8:20913523-20913545 | 20913540 | 3 | DNA | GGCCGAGGTCGACTACCGG-CNRG | GctgGAGGTCGACTtCCtGCCCGG | + | 5 | 1 | 0 | 4.41 | 2.21 |
| Chr10:23768318-23768340 | 23768324 | 4 | RNA | GGCCGAGGTCGACTACCGGCNRG | GGCCGAcGaCa--TACCtGgAGG | - | 5 | 2 | 0 | 4.41 | 2.21 |
| Chr2:90292371-90292393 | 90292376 | 3 | RNA | GGCCGAGGTCGACTACCGGCNRG | GGCCG-GGTCGtCcACtGGCGGG | - | 3 | 1 | 0 | 4.41 | 2.21 |
| Chr6:71306205-71306227 | 71306221 | 4 | DNA | GGCCGAGGTCGA--CTACCGGCNRG | GcgCcAtcTCGATTCTACCGGCGAG | + | 5 | 2 | 0 | 4.41 | 2.21 |
| Chr2:16285307-16285329 | 16285325 | 2 | RNA | GGCCGAGGTCGACTACCGGCNRG | GcCCGAGccCGAgT--CGGtAGG | + | 5 | 2 | 0 | 4.41 | 2.21 |
| Chr5:36749334-36749356 | 36749338 | 2 | RNA | GGCCGAGGTCGACTACCGGCNRG | GaCgGgGGTCGtCTAC-GGgTAG | - | 5 | 1 | 0 | 4.41 | 2.21 |
| Chr2:232550878-232550900 | 232550885 | 5 | RNA | GGCCGAGGTCGACTACCGGCNRG | caCCaAGtTCGcCT-CCGGCCAG | - | 5 | 1 | 0 | 4.41 | 2.21 |
| Chr3:104176262-104176284 | 104176269 | 5 | RNA | GGCCGAGGTCGACTACCGGCNRG | GGCCGAGGcCtcCT-CgGGCTGG | - | 4 | 1 | 0 | 4.41 | 2.21 |
| Chr2:210153486-210153508 | 210153493 | 5 | RNA | GGCCGAGGTCGACTACCGGCNRG | GGCaaActTCGgCTAC--GCGGG | - | 5 | 2 | 0 | 4.41 | 2.21 |
| Chr7:93462714-93462736 | 93462729 | 5 | RNA | GGCCGAGGTCGACTACCGGCNRG | GGC-GAaGTCGAagACCtcCCAG | + | 5 | 1 | 0 | 4.41 | 2.21 |
| Chr2:188670649-188670671 | 188670667 | 2 | RNA | GGCCGAGGTCGACTACCGGCNRG | GtCCG--GTgcACcACCGGaCAG | + | 5 | 2 | 0 | 4.41 | 2.21 |
| Chr5:119886116-119886138 | 119886134 | 2 | DNA | GGC-CGAGGTCGACTACCGGCNRG | GcCTCGgGGaCGtCTtCCGGCTGG | + | 5 | 1 | 0 | 4.41 | 2.21 |
| Chr7:65064182-65064204 | 65064200 | 2 | RNA | GGCCGAGGTCGACTACCGGCNRG | cGCCGtGcTC--CgACCGGtGGG | + | 5 | 2 | 0 | 4.41 | 2.21 |
| Chr7:84651792-84651814 | 84651810 | 2 | RNA | GGCCGAGGTCGACTACCGGCNRG | GGCCGtctTCGAgTA--GaCCGG | + | 5 | 2 | 0 | 4.41 | 2.21 |
| Chr7:101820957-101820979 | 101820963 | 4 | DNA | GGC-CGAGGTCGACTACCGGCNRG | GcCTCGgGGaCGtCTtCCGGCTGG | - | 5 | 1 | 0 | 4.41 | 2.21 |
| Chr7:80303198-80303220 | 80303202 | 2 | RNA | GGCCGAGGTCGACTACCGGCNRG | tGaCGAGGTCGAC-AaCGagCGG | - | 5 | 1 | 0 | 4.41 | 2.21 |
| Chr4:158415050-158415072 | 158415057 | 5 | RNA | GGCCGAGGTCGACTACCGGCNRG | GaC--AGGTgGgCcACCGGaCAG | - | 5 | 2 | 0 | 4.41 | 2.21 |
| Chr10:26799035-26799057 | 26799042 | 5 | RNA | GGCCGAGGTCGACTACCGGCNRG | cGCCGtGGataACT-CCGGCCAG | - | 5 | 1 | 0 | 4.41 | 2.21 |
| Chr4:222763126-222763148 | 222763131 | 3 | RNA | GGCCGAGGTCGACTACCGGCNRG | G-aCGgGGTCGACgACgaGCGGG | - | 5 | 1 | 0 | 4.41 | 2.21 |
| Chr1:3169837-3169859 | 3169855 | 2 | RNA | GGCCGAGGTCGACTACCGGCNRG | GGa--AGGcCtACTACCaGCAAG | + | 4 | 2 | 0 | 4.41 | 2.21 |
| Chr7:5224528-5224550 | 5224533 | 3 | X | GGCCGAGGTCGACTACCGGCNRG | GaCCGtGGTCGACcgtCGGCTGG | - | 5 | 0 | 0 | 4.41 | 2.21 |
| Chr8:13338032-13338054 | 13338037 | 3 | RNA | GGCCGAGGTCGACTACCGGCNRG | cGtCGAaGTCGACgACCt-CGAG | - | 5 | 1 | 0 | 4.41 | 2.21 |
| Chr2:154847211-154847233 | 154847228 | 3 | RNA | GGCCGAGGTCGACTACCGGCNRG | GGCCaAGG--GACacCCacCTGG | + | 5 | 2 | 0 | 4.41 | 2.21 |
| Chr5:117804758-117804780 | 117804762 | 2 | DNA | GGCCGAGG--TCGACTACCGGCNRG | GGCCGAGGCCTCGACgcgCGcgGAG | - | 5 | 2 | 0 | 4.41 | 2.21 |
| Chr10:24024196-24024218 | 24024200 | 2 | RNA | GGCCGAGGTCGACTACCGGCNRG | G-aCGgGGTCGACgACgtGCGGG | - | 5 | 1 | 0 | 4.41 | 2.21 |
| Chr8:45808111-45808133 | 45808127 | 4 | RNA | GGCCGAGGTCGACTACCGGCNRG | GGCgGAaGTCG--TtCgGcCGAG | + | 5 | 2 | 0 | 4.41 | 2.21 |
| Chr6:160577612-160577634 | 160577630 | 2 | RNA | GGCCGAGGTCGACTACCGGCNRG | aGC--tGtTCGtCaACCGGCAAG | + | 5 | 2 | 0 | 4.41 | 2.21 |
| Chr1:28144461-28144483 | 28144477 | 4 | RNA | GGCCGAGGTCGACTACCGGCNRG | GGgCGAGtTCG--cACCtGCAGG | + | 4 | 2 | 0 | 4.41 | 2.21 |
| Chr4:230159861-230159883 | 230159866 | 3 | RNA | GGCCGAGGTCGACTACCGGCNRG | GGCC-AGGTCGtCcACtGGtGGG | - | 4 | 1 | 0 | 4.41 | 2.21 |
| Chr4:15656113-15656135 | 15656129 | 4 | RNA | GGCCGAGGTCGACTACCGGCNRG | GGC--AGGcCGAgcACtaGCCGG | + | 5 | 2 | 0 | 4.41 | 2.21 |
| Chr4:156382348-156382370 | 156382353 | 3 | DNA | GGCCGA-GGTCGACTACCGGCNRG | GGCgGACGGTCcgCgACCGaCCAG | - | 5 | 1 | 0 | 4.41 | 2.21 |
| Chr7:57927816-57927838 | 57927834 | 2 | RNA | GGCCGAGGTCGACTACCGGCNRG | GGCgGAGGTgGACgAaa--CCAG | + | 5 | 2 | 0 | 4.41 | 2.21 |
| Chr1:245947671-245947693 | 245947689 | 2 | RNA | GGCCGAGGTCGACTACCGGCNRG | t--CGgGGaCGtCTtCCGGCTGG | + | 5 | 2 | 0 | 4.41 | 2.21 |
| Chr4:191564214-191564236 | 191564220 | 4 | RNA | GGCCGAGGTCGACTACCGGCNRG | GGgCGAG--CGAtgAggGGCAAG | - | 5 | 2 | 0 | 4.41 | 2.21 |
| Chr5:141834972-141834994 | 141834977 | 3 | RNA | GGCCGAGGTCGACTACCGGCNRG | G--tGAGGTCGACaACgGctAAG | - | 5 | 2 | 0 | 4.41 | 2.21 |
| Chr3:22899129-22899151 | 22899145 | 4 | RNA | GGCCGAGGTCGACTACCGGCNRG | GtCCG--GTgcACcACCGGaCAG | + | 5 | 2 | 0 | 4.41 | 2.21 |
| Chr5:53466171-53466193 | 53466188 | 3 | RNA | GGCCGAGGTCGACTACCGGCNRG | GGtCGAGcTCatCTAgC--CTGG | + | 5 | 2 | 0 | 4.41 | 2.21 |
| Chr8:15070972-15070994 | 15070989 | 3 | RNA | GGCCGAGGTCGACTACCGGCNRG | GGtCGAGGcCGAgT--CcGgGGG | + | 5 | 2 | 0 | 4.41 | 2.21 |
| Chr4:214932465-214932487 | 214932482 | 3 | RNA | GGCCGAGGTCGACTACCGGCNRG | GGtgGtGGTCGACgAC--GaAGG | + | 5 | 2 | 0 | 4.41 | 2.21 |
| Chr1:107398153-107398175 | 107398158 | 3 | RNA | GGCCGAGGTCGACTACCGGCNRG | GGCCGAGGTC--CTtCttttCGG | - | 5 | 2 | 0 | 4.41 | 2.21 |
| Chr4:18490741-18490763 | 18490745 | 2 | RNA | GGCCGAGGTCGACTACCGGCNRG | GGaCGAcGaCGACgACa--CTGG | - | 5 | 2 | 0 | 4.41 | 2.21 |
| Chr4:181163887-181163909 | 181163892 | 3 | RNA | GGCCGAGGTCGACTACCGGCNRG | aGC--tcGcgGACTACCGGCTGG | - | 5 | 2 | 0 | 4.41 | 2.21 |
| Chr1:69266492-69266514 | 69266508 | 4 | RNA | GGCCGAGGTCGACTACCGGCNRG | G-aCGgGGTCGACaACgaGCGGG | + | 5 | 1 | 0 | 4.41 | 2.21 |
| Chr4:142111391-142111413 | 142111406 | 5 | RNA | GGCCGAGGTCGACTACCGGCNRG | acCCGAtGTCG--TACgaGCTAG | + | 5 | 2 | 0 | 4.41 | 2.21 |
| Chr3:76297619-76297641 | 76297623 | 2 | RNA | GGCCGAGGTCGACTACCGGCNRG | GGaCGAGcTCGACTAa-GGgTGG | - | 4 | 1 | 0 | 4.41 | 2.21 |
| Chr1:60782069-60782091 | 60782074 | 3 | DNA | GGC-CGAGGTCGACTACCGGCNRG | GGCTCcAtGTCGACTACCaGCAGG | - | 3 | 1 | 0 | 4.41 | 2.21 |
| Chr5:69002134-69002156 | 69002151 | 3 | RNA | GGCCGAGGTCGACTACCGGCNRG | GGtCGAGGT--AacttCGGCGAG | + | 5 | 2 | 0 | 4.41 | 2.21 |
| Chr2:21783874-21783896 | 21783881 | 5 | RNA | GGCCGAGGTCGACTACCGGCNRG | GaaCGAGaTCGcCT-CCGGgGAG | - | 5 | 1 | 0 | 4.41 | 2.21 |
| Chr4:979278-979300 | 979283 | 3 | RNA | GGCCGAGGTCGACTACCGGCNRG | GGaCaAcGaCGACgAC--GCCGG | - | 5 | 2 | 0 | 4.41 | 2.21 |
| Chr2:133539387-133539409 | 133539402 | 5 | RNA | GGCCGAGGTCGACTACCGGCNRG | caCCaAGtTCGcCT-CCGGCCAG | + | 5 | 1 | 0 | 4.41 | 2.21 |
| Chr2:77850122-77850144 | 77850137 | 5 | RNA | GGCCGAGGTCGACTACCGGCNRG | GGC--AGGcaaACctCCGGCCGG | + | 5 | 2 | 0 | 4.41 | 2.21 |
| Chr5:108160504-108160526 | 108160522 | 2 | RNA | GGCCGAGGTCGACTACCGGCNRG | G--CGAGGgtGACgcCCaGCAAG | + | 5 | 2 | 0 | 4.41 | 2.21 |
| Chr1:199867954-199867976 | 199867961 | 5 | RNA | GGCCGAGGTCGACTACCGGCNRG | GGC-GtGGcCGAactCCGGCGAG | - | 5 | 1 | 0 | 4.41 | 2.21 |
| Chr2:178852051-178852073 | 178852058 | 5 | RNA | GGCCGAGGTCGACTACCGGCNRG | cGgCGgGGcgG--TACCGGCGAG | - | 5 | 2 | 0 | 4.41 | 2.21 |
| Chr4:183543935-183543957 | 183543950 | 5 | RNA | GGCCGAGGTCGACTACCGGCNRG | GGCCGAGGTCG--ggCgaGgCGG | + | 5 | 2 | 0 | 4.41 | 2.21 |
| Chr2:81903156-81903178 | 81903173 | 3 | RNA | GGCCGAGGTCGACTACCGGCNRG | GGCgGAGGTCG--TtCgGcaGAG | + | 5 | 2 | 0 | 4.41 | 2.21 |
| Chr9:89455202-89455224 | 89455207 | 3 | RNA | GGCCGAGGTCGACTACCGGCNRG | GGggGgaGTaG--TACCGGCAGG | - | 5 | 2 | 0 | 4.41 | 2.21 |
| Chr8:121283895-121283917 | 121283900 | 3 | RNA | GGCCGAGGTCGACTACCGGCNRG | G--tGAGGTaatCaACCGGCAGG | - | 5 | 2 | 0 | 4.41 | 2.21 |
| Chr4:82989186-82989208 | 82989204 | 2 | RNA | GGCCGAGGTCGACTACCGGCNRG | GGaCcAGGTCG--TcCCaGtTGG | + | 5 | 2 | 0 | 4.41 | 2.21 |
| Chr1:4753025-4753047 | 4753030 | 3 | RNA | GGCCGAGGTCGACTACCGGCNRG | GaCaG-GGTCGACgACaaGCGGG | - | 5 | 1 | 0 | 4.41 | 2.21 |
| Chr1:127154321-127154343 | 127154337 | 4 | RNA | GGCCGAGGTCGACTACCGGCNRG | GcCCGAGGaCaACcACC--aGAG | + | 5 | 2 | 0 | 4.41 | 2.21 |
| Chr8:140452244-140452266 | 140452259 | 5 | RNA | GGCCGAGGTCGACTACCGGCNRG | a--CGgGGTCGACgACgaGCGGG | + | 5 | 2 | 0 | 4.41 | 2.21 |
| Chr4:217051907-217051929 | 217051914 | 5 | RNA | GGCCGAGGTCGACTACCGGCNRG | GGgCGAGcTCGggTA--GGgAGG | - | 5 | 2 | 0 | 4.41 | 2.21 |
| Chr9:100658636-100658658 | 100658652 | 4 | RNA | GGCCGAGGTCGACTACCGGCNRG | cGCCGAtcTCGA-TAgtGGCAAG | + | 5 | 1 | 0 | 4.41 | 2.21 |
| Chr2:85713475-85713497 | 85713491 | 4 | RNA | GGCCGAGGTCGACTACCGGCNRG | tG--GAGGagGACgACCtGCTGG | + | 5 | 2 | 0 | 4.41 | 2.21 |
| Chr8:60988167-60988189 | 60988173 | 4 | RNA | GGCCGAGGTCGACTACCGGCNRG | t--CGgGGaCGtCTtCCGGCTGG | - | 5 | 2 | 0 | 4.41 | 2.21 |
| Chr5:181078453-181078475 | 181078457 | 2 | RNA | GGCCGAGGTCGACTACCGGCNRG | GGCgGAGGTCG--TtCgGcaGAG | - | 5 | 2 | 0 | 4.41 | 2.21 |
| Chr5:45229043-45229065 | 45229060 | 3 | RNA | GGCCGAGGTCGACTACCGGCNRG | cGgCGAGcT--AaTACtGGCCAG | + | 5 | 2 | 0 | 4.41 | 2.21 |
| Chr3:229193108-229193130 | 229193113 | 3 | RNA | GGCCGAGGTCGACTACCGGCNRG | GGC--tcGcgGACTACCGGCTGG | - | 4 | 2 | 0 | 4.41 | 2.21 |
| Chr4:6514333-6514355 | 6514340 | 5 | RNA | GGCCGAGGTCGACTACCGGCNRG | caCCaAGtTCGcCT-CCGGCCAG | - | 5 | 1 | 0 | 4.41 | 2.21 |
| Chr9:99959380-99959402 | 99959397 | 3 | RNA | GGCCGAGGTCGACTACCGGCNRG | GGaCcAGGTCG--TcCCaGtTGG | + | 5 | 2 | 0 | 4.41 | 2.21 |
| Chr2:234019439-234019461 | 234019457 | 2 | RNA | GGCCGAGGTCGACTACCGGCNRG | cGCgcAGGTCGAC--CgGGaCAG | + | 5 | 2 | 0 | 4.41 | 2.21 |
| Chr10:147367339-147367361 | 147367354 | 5 | RNA | GGCCGAGGTCGACTACCGGCNRG | G--CGAGtaCaACgACaGGCCGG | + | 5 | 2 | 0 | 4.41 | 2.21 |
| Chr5:107568350-107568372 | 107568368 | 2 | RNA | GGCCGAGGTCGACTACCGGCNRG | GGCCGAtcTCG--cAtCaGCAAG | + | 5 | 2 | 0 | 4.41 | 2.21 |
| Chr1:236302922-236302944 | 236302938 | 4 | RNA | GGCCGAGGTCGACTACCGGCNRG | G-aCGgGGTCGACaACgaGCGGG | + | 5 | 1 | 0 | 4.41 | 2.21 |
| Chr5:138518702-138518724 | 138518717 | 5 | RNA | GGCCGAGGTCGACTACCGGCNRG | GGgtGgGGgtGA--ACCGGCCGG | + | 5 | 2 | 0 | 4.41 | 2.21 |
| Chr9:58193135-58193157 | 58193153 | 2 | DNA | GGCCGAGGT--CGACTACCGGCNRG | GGCCaAGGTATCGAgaAgCaGCAAG | + | 5 | 2 | 0 | 4.41 | 2.21 |
| Chr1:33043023-33043045 | 33043040 | 3 | RNA | GGCCGAGGTCGACTACCGGCNRG | tGaCGAGaTCaACgA--GGCTAG | + | 5 | 2 | 0 | 4.41 | 2.21 |
| Chr8:100063800-100063822 | 100063815 | 5 | RNA | GGCCGAGGTCGACTACCGGCNRG | caCCaAGtTCGcCT-CCGGCCAG | + | 5 | 1 | 0 | 4.41 | 2.21 |
| Chr7:66275684-66275706 | 66275689 | 3 | RNA | GGCCGAGGTCGACTACCGGCNRG | GcCCatccTCGACT-CCGGCAAG | - | 5 | 1 | 0 | 4.41 | 2.21 |
| Chr2:33024235-33024257 | 33024241 | 4 | RNA | GGCCGAGGTCGACTACCGGCNRG | G-aCGgGGTCGACgACgaGCGGG | - | 5 | 1 | 0 | 4.41 | 2.21 |
| Chr5:50647281-50647303 | 50647299 | 2 | RNA | GGCCGAGGTCGACTACCGGCNRG | GGaCGAGGcgG--cAaCGGCAAG | + | 5 | 2 | 0 | 4.41 | 2.21 |
| Chr4:74571845-74571867 | 74571862 | 3 | RNA | GGCCGAGGTCGACTACCGGCNRG | GGC--tcGcaGACTAtCGGCTGG | + | 5 | 2 | 0 | 4.41 | 2.21 |
| Chr8:100462307-100462329 | 100462314 | 5 | RNA | GGCCGAGGTCGACTACCGGCNRG | cGCCGtGGataACT-CCGGCCAG | - | 5 | 1 | 0 | 4.41 | 2.21 |
| Chr8:25433199-25433221 | 25433217 | 2 | DNA | GGCCGAGGTCGAC-TACCGGCNRG | cGCCGAcGTCGACCggCCGGCCGG | + | 4 | 1 | 0 | 4.41 | 2.21 |
| Chr8:76767194-76767216 | 76767211 | 3 | RNA | GGCCGAGGTCGACTACCGGCNRG | GGaCGAGGTtGAC--CCtatGGG | + | 5 | 2 | 0 | 4.41 | 2.21 |
| Chr4:174694702-174694724 | 174694718 | 4 | RNA | GGCCGAGGTCGACTACCGGCNRG | G-CCGgGGTCGgCTcCgGGtCAG | + | 5 | 1 | 0 | 4.41 | 2.21 |
| Chr8:14624935-14624957 | 14624951 | 4 | RNA | GGCCGAGGTCGACTACCGGCNRG | G-aCGgGGTCGACaACgaGCGGG | + | 5 | 1 | 0 | 4.41 | 2.21 |
| Chr10:146259686-146259708 | 146259691 | 3 | RNA | GGCCGAGGTCGACTACCGGCNRG | GGgCGAGGaCGACggC--GCCAG | - | 4 | 2 | 0 | 4.41 | 2.21 |
| Chr2:126177717-126177739 | 126177735 | 2 | DNA | GGCCGAGGTCGACT-ACCGGCNRG | GGttGAGGaCGACTGACaGGtGGG | + | 5 | 1 | 0 | 4.41 | 2.21 |
| Chr7:16224428-16224450 | 16224432 | 2 | RNA | GGCCGAGGTCGACTACCGGCNRG | aGCaGAG--CGAacgCCGGCGGG | - | 5 | 2 | 0 | 4.41 | 2.21 |
| Chr3:15414838-15414860 | 15414844 | 4 | RNA | GGCCGAGGTCGACTACCGGCNRG | GcCCGAGGcCaAC--CCtGgGAG | - | 5 | 2 | 0 | 4.41 | 2.21 |
| Chr2:243544327-243544349 | 243544331 | 2 | RNA | GGCCGAGGTCGACTACCGGCNRG | GG--GcGGTgGtCTAaCGcCTAG | - | 5 | 2 | 0 | 4.41 | 2.21 |
| Chr3:233249283-233249305 | 233249299 | 4 | DNA | GGCCGAGGTCGACTA--CCGGCNRG | ctCCtAGGcCGACcAATCCGGCAGG | + | 5 | 2 | 0 | 4.41 | 2.21 |
| Chr9:70137970-70137992 | 70137986 | 4 | RNA | GGCCGAGGTCGACTACCGGCNRG | GGCaGAGGgCG--TAgaGGgGAG | + | 5 | 2 | 0 | 4.41 | 2.21 |
| Chr4:119179746-119179768 | 119179761 | 5 | RNA | GGCCGAGGTCGACTACCGGCNRG | GGgCGAGcTCGggTA--GGaAGG | + | 5 | 2 | 0 | 4.41 | 2.21 |
| Chr7:173203467-173203489 | 173203472 | 3 | RNA | GGCCGAGGTCGACTACCGGCNRG | GGa-GAcGgCGAgcACCGGCGAG | - | 5 | 1 | 0 | 4.41 | 2.21 |
| Chr2:94781043-94781065 | 94781058 | 5 | RNA | GGCCGAGGTCGACTACCGGCNRG | GcCaGtGGaCGAC--CCGGCCAG | + | 4 | 2 | 0 | 4.41 | 2.21 |
| Chr1:169593712-169593734 | 169593719 | 5 | RNA | GGCCGAGGTCGACTACCGGCNRG | aGaCGAGcTCGAgTA--GGaAGG | - | 5 | 2 | 0 | 4.41 | 2.21 |
| Chr4:108825550-108825572 | 108825566 | 4 | RNA | GGCCGAGGTCGACTACCGGCNRG | GGC-GAGcaCGtagACCGGCCGG | + | 5 | 1 | 0 | 4.41 | 2.21 |
| Chr1:84537561-84537583 | 84537579 | 2 | RNA | GGCCGAGGTCGACTACCGGCNRG | GGCCcAtcTtGgCT--CGGCCAG | + | 5 | 2 | 0 | 4.41 | 2.21 |
| Chr2:191368621-191368643 | 191368627 | 4 | RNA | GGCCGAGGTCGACTACCGGCNRG | GGCaGAGGTCGACag--tGgCGG | - | 5 | 2 | 0 | 4.41 | 2.21 |
| Chr3:207125593-207125615 | 207125600 | 5 | RNA | GGCCGAGGTCGACTACCGGCNRG | GGgCG-GGcCaACctCCGGCCGG | - | 5 | 1 | 0 | 4.41 | 2.21 |
| Chr8:25883914-25883936 | 25883921 | 5 | RNA | GGCCGAGGTCGACTACCGGCNRG | GttCGgGGaCGtCT-CCGGCGAG | - | 5 | 1 | 0 | 4.41 | 2.21 |
| Chr10:46944409-46944431 | 46944414 | 3 | X | GGCCGAGGTCGACTACCGGCNRG | GGaaGAGGTgGcCTAgCGGCTGG | - | 5 | 0 | 1 | 3.30 | 2.15 |
| Chr9:104276813-104276835 | 104276831 | 2 | RNA | GGCCGAGGTCGACTACCGGCNRG | GcCCGAGGgC--CTAaCaaCAAG | + | 5 | 2 | 1 | 3.30 | 2.15 |
| Chr5:140916743-140916765 | 140916760 | 3 | DNA | GGCCGAGGTCGACTAC--CGGCNRG | GaCCGtGGTCGACgACAACtcCGAG | + | 5 | 2 | 1 | 3.30 | 2.15 |
| Chr3:178918303-178918325 | 178918318 | 5 | RNA | GGCCGAGGTCGACTACCGGCNRG | aGC--AGGgCaACagCCGGCCGG | + | 5 | 2 | 1 | 3.30 | 2.15 |
| Chr6:150262725-150262747 | 150262743 | 2 | DNA | GGCCGAGGTCGAC-TACCGGCNRG | GGCtGAGcaCcACGTcCCGGCCGG | + | 5 | 1 | 2 | 2.20 | 2.10 |
| Chr2:34358768-34358790 | 34358784 | 4 | RNA | GGCCGAGGTCGACTACCGGCNRG | GGaCGAGcTCGggTA--GGgGAG | + | 5 | 2 | 2 | 2.20 | 2.10 |
| Chr2:170250011-170250033 | 170250026 | 5 | RNA | GGCCGAGGTCGACTACCGGCNRG | GGC--ttGcgGACTACCGGCTGG | + | 4 | 2 | 3 | 1.10 | 2.05 |
| Chr7:115961873-115961895 | 115961891 | 2 | RNA | GGCCGAGGTCGACTACCGGCNRG | GGCCGAGGTgGgtTgCC--tGAG | + | 5 | 2 | 3 | 1.10 | 2.05 |
| Chr2:120616378-120616400 | 120616385 | 5 | RNA | GGCCGAGGTCGACTACCGGCNRG | cGCCGAGaTC--CTctCcGCTGG | - | 5 | 2 | 4 | 0.00 | 2.00 |
| Chr8:33112794-33112816 | 33112811 | 3 | RNA | GGCCGAGGTCGACTACCGGCNRG | a--CGAGGTCGACccCgGGCAGG | + | 4 | 2 | 4 | 0.00 | 2.00 |
| Chr5:103082943-103082965 | 103082960 | 3 | RNA | GGCCGAGGTCGACTACCGGCNRG | tcgCaAGGTCGAaT-CCGGCTGG | + | 5 | 1 | 4 | 0.00 | 2.00 |
| Chr9:44214025-44214047 | 44214032 | 5 | RNA | GGCCGAGGTCGACTACCGGCNRG | cGgCttGGcCGACT-CCGGCGGG | - | 5 | 1 | 4 | 0.00 | 2.00 |
| Chr4:200401971-200401993 | 200401978 | 5 | RNA | GGCCGAGGTCGACTACCGGCNRG | GGCCGAGGcCGg-TgCgGGgAAG | - | 5 | 1 | 4 | 0.00 | 2.00 |
| Chr8:148563265-148563287 | 148563282 | 3 | RNA | GGCCGAGGTCGACTACCGGCNRG | GtCCG--GTgcACcACCGGaCAG | + | 5 | 2 | 4 | 0.00 | 2.00 |
| Chr7:104372058-104372080 | 104372065 | 5 | RNA | GGCCGAGGTCGACTACCGGCNRG | GtCCG--GTgcACcACCGGaTAG | - | 5 | 2 | 4 | 0.00 | 2.00 |
| Chr1:175297382-175297404 | 175297389 | 5 | RNA | GGCCGAGGTCGACTACCGGCNRG | caCCaAGtTCGcCT-CCGGCCAG | - | 5 | 1 | 4 | 0.00 | 2.00 |
| Chr4:119510804-119510826 | 119510821 | 3 | RNA | GGCCGAGGTCGACTACCGGCNRG | GGgCGAGcTCGtC-ggCGGCGGG | + | 5 | 1 | 4 | 0.00 | 2.00 |
| Chr8:103125843-103125865 | 103125860 | 3 | RNA | GGCCGAGGTCGACTACCGGCNRG | GGC--tcGcgGACTACCGGCTGG | + | 4 | 2 | 4 | 0.00 | 2.00 |
| Chr5:25703564-25703586 | 25703581 | 3 | RNA | GGCCGAGGTCGACTACCGGCNRG | GGaCaAG--CGACTcCCaGtAGG | + | 5 | 2 | 4 | 0.00 | 2.00 |
| Chr1:36106154-36106176 | 36106171 | 3 | RNA | GGCCGAGGTCGACTACCGGCNRG | caCCGAGtTCGcCT-CCGcCGGG | + | 5 | 1 | 4 | 0.00 | 2.00 |

| | | | | | | | | | | | |
|---|---|---|---|---|---|---|---|---|---|---|---|
| Chr5:10008881-10008903 | 10008898 | 3 | RNA | GGCCGAGGTCGACTACCGGCNRG | GGCCacGGTgG--cgCCGGCGAG | + | 5 | 2 | 4 | 0.00 | 2.00 |
| Chr6:124220122-124220144 | 124220129 | 5 | RNA | GGCCGAGGTCGACTACCGGCNRG | GGC--ttGcgGAtTACCGGCTGG | - | 5 | 2 | 4 | 0.00 | 2.00 |
| Chr6:67659448-67659470 | 67659465 | 3 | RNA | GGCCGAGGTCGACTACCGGCNRG | caCaGAcGTCGAC-gCCGGCGAG | + | 5 | 1 | 4 | 0.00 | 2.00 |
| Chr6:134674774-134674796 | 134674791 | 3 | DNA | GGCCGAGGTCGAC--TACCGGCNRG | GGtCGtGGTCGACGTTgttGGCCGG | + | 5 | 2 | 4 | 0.00 | 2.00 |
| Chr2:146280432-146280454 | 146280449 | 3 | RNA | GGCCGAGGTCGACTACCGGCNRG | GGgC-AGGcCaACctCCGGCCGG | + | 5 | 1 | 4 | 0.00 | 2.00 |
| Chr6:62117489-62117511 | 62117496 | 5 | RNA | GGCCGAGGTCGACTACCGGCNRG | aGCCGAGGgCGcCT-CgGGCAGG | - | 4 | 1 | 4 | 0.00 | 2.00 |
| Chr7:134571105-134571127 | 134571122 | 3 | RNA | GGCCGAGGTCGACTACCGGCNRG | t--CGgGGaCGtCTtCCGGCTGG | + | 5 | 2 | 4 | 0.00 | 2.00 |
| Chr1:182262755-182262777 | 182262762 | 5 | RNA | GGCCGAGGTCGACTACCGGCNRG | G--CGAaGaCcACcACCtGCAGG | - | 5 | 2 | 4 | 0.00 | 2.00 |
| Chr8:91840490-91840512 | 91840507 | 3 | RNA | GGCCGAGGTCGACTACCGGCNRG | G-aCGgGGTCGACaACgaGCGGG | + | 5 | 1 | 4 | 0.00 | 2.00 |
| Chr2:160145330-160145352 | 160145337 | 5 | RNA | GGCCGAGGTCGACTACCGGCNRG | GtCCG--GTgcACcACCGGaTAG | - | 5 | 2 | 4 | 0.00 | 2.00 |
| Chr10:13123129-13123151 | 13123136 | 5 | RNA | GGCCGAGGTCGACTACCGGCNRG | G-aCGgGGTCGACgACgaGCGGG | - | 5 | 1 | 4 | 0.00 | 2.00 |
| Chr7:3261505-3261527 | 3261522 | 3 | RNA | GGCCGAGGTCGACTACCGGCNRG | GGCCGAGtaCG--TtgCcGCAGG | + | 5 | 2 | 4 | 0.00 | 2.00 |
| Chr1:273999940-273999962 | 273999947 | 5 | RNA | GGCCGAGGTCGACTACCGGCNRG | GGC--tcGTgGACTACCGttTGG | - | 5 | 2 | 4 | 0.00 | 2.00 |
| Chr1:180020357-180020379 | 180020374 | 3 | RNA | GGCCGAGGTCGACTACCGGCNRG | GaC--AGGcacACTAgCGGCCAG | + | 5 | 2 | 4 | 0.00 | 2.00 |
| Chr1:129321638-129321660 | 129321645 | 5 | RNA | GGCCGAGGTCGACTACCGGCNRG | GGtCGgGGTtG--ggCCGGCTGG | - | 5 | 2 | 4 | 0.00 | 2.00 |
| Chr2:142398122-142398144 | 142398139 | 3 | RNA | GGCCGAGGTCGACTACCGGCNRG | GGCCGAtGgCG--ggCgGGCGGG | + | 5 | 2 | 4 | 0.00 | 2.00 |
| Chr5:101667792-101667814 | 101667799 | 5 | DNA | GGCCGAGGTCGAC-TACCGGCNRG | GGCCGgcGgCGAgGaACCGGCCGG | - | 5 | 1 | 4 | 0.00 | 2.00 |
| Chr10:100218755-100218777 | 100218762 | 5 | RNA | GGCCGAGGTCGACTACCGGCNRG | GG--GAGGTCGgCTAggaGtTGG | - | 5 | 2 | 4 | 0.00 | 2.00 |
| Chr1:31114334-31114356 | 31114351 | 3 | RNA | GGCCGAGGTCGACTACCGGCNRG | GGCCGAGGc--ACcACgtGaCGG | + | 5 | 2 | 4 | 0.00 | 2.00 |
| Chr6:46957466-46957488 | 46957473 | 5 | RNA | GGCCGAGGTCGACTACCGGCNRG | GGCCGActTCG--TgtgGGCAGG | - | 5 | 2 | 4 | 0.00 | 2.00 |
| Chr1:124945096-124945118 | 124945103 | 5 | RNA | GGCCGAGGTCGACTACCGGCNRG | GcCgGAGGcCGACTt--GGaAGG | - | 5 | 2 | 4 | 0.00 | 2.00 |
| Chr1:21226460-21226482 | 21226477 | 3 | RNA | GGCCGAGGTCGACTACCGGCNRG | GtCttgGGT-GACcACCGGCGGG | + | 5 | 1 | 4 | 0.00 | 2.00 |
| Chr1:195135647-195135669 | 195135654 | 5 | DNA | GGCCGAGGTCGACTA--CCGGCNRG | cGCaaAGGcCGACcAATCCGGCAGG | - | 5 | 2 | 4 | 0.00 | 2.00 |
| Chr3:41464102-41464124 | 41464119 | 3 | RNA | GGCCGAGGTCGACTACCGGCNRG | aGCaGAG--CGAacgCCGGCTGG | + | 5 | 2 | 4 | 0.00 | 2.00 |
| Chr7:155080042-155080064 | 155080059 | 3 | RNA | GGCCGAGGTCGACTACCGGCNRG | G-aCGgGGTCGACaACgaGCGGG | + | 5 | 1 | 4 | 0.00 | 2.00 |
| Chr4:51993306-51993328 | 51993313 | 5 | RNA | GGCCGAGGTCGACTACCGGCNRG | GGCCGcGccCG-CTcCCGGCCGG | - | 4 | 1 | 4 | 0.00 | 2.00 |
| Chr10:37363083-37363105 | 37363090 | 5 | RNA | GGCCGAGGTCGACTACCGGCNRG | GaCCGgGGTCGAC-gCgaGCAGG | - | 5 | 1 | 4 | 0.00 | 2.00 |
| Chr8:84846561-84846583 | 84846568 | 5 | RNA | GGCCGAGGTCGACTACCGGCNRG | GGgCG-GGcCaACctCCGGCCAG | - | 5 | 1 | 4 | 0.00 | 2.00 |
| Chr10:136196658-136196680 | 136196665 | 5 | RNA | GGCCGAGGTCGACTACCGGCNRG | GGC-GAGGTCGAggACCttgGGG | - | 5 | 1 | 4 | 0.00 | 2.00 |
| Chr4:119702439-119702461 | 119702446 | 5 | DNA | GGCCGAGGTCGACTACCG--GCNRG | GGaaGAcGTCGcCgACCGCCGCGGG | - | 5 | 2 | 4 | 0.00 | 2.00 |
| Chr1:86560489-86560511 | 86560506 | 3 | RNA | GGCCGAGGTCGACTACCGGCNRG | GtCCG--GTgcACcACCGGaCAG | + | 5 | 2 | 4 | 0.00 | 2.00 |
| Chr9:58389501-58389523 | 58389508 | 5 | RNA | GGCCGAGGTCGACTACCGGCNRG | GGgCGAGG-CGcCTctaGGCAAG | - | 5 | 1 | 4 | 0.00 | 2.00 |
| Chr5:11844047-11844069 | 11844064 | 3 | RNA | GGCCGAGGTCGACTACCGGCNRG | GGatGAGGagGACT--CGGaCGG | + | 5 | 2 | 4 | 0.00 | 2.00 |
| Chr3:124078529-124078551 | 124078546 | 3 | RNA | GGCCGAGGTCGACTACCGGCNRG | GaCCGAGG-CGACgACaaGaAGG | + | 5 | 1 | 4 | 0.00 | 2.00 |
| Chr3:205364199-205364221 | 205364206 | 5 | RNA | GGCCGAGGTCGACTACCGGCNRG | GtaCGtGGTtG--TgCCGGCTAG | - | 5 | 2 | 4 | 0.00 | 2.00 |
| Chr1:183235274-183235296 | 183235281 | 5 | RNA | GGCCGAGGTCGACTACCGGCNRG | cGtCGAGGaCGAC-AgaGGCAAG | - | 5 | 1 | 4 | 0.00 | 2.00 |
| Chr1:16240986-16241008 | 16240993 | 5 | RNA | GGCCGAGGTCGACTACCGGCNRG | G--CGcGGTCGcacgCCGGCGAG | - | 5 | 2 | 4 | 0.00 | 2.00 |
| Chr1:262648365-262648387 | 262648382 | 3 | RNA | GGCCGAGGTCGACTACCGGCNRG | GGgCGAGGaCaAg--CCGcCAAG | + | 5 | 2 | 4 | 0.00 | 2.00 |
| Chr9:96047903-96047925 | 96047920 | 3 | RNA | GGCCGAGGTCGACTACCGGCNRG | tGCaGAGcTCGACTA--GGtTGG | + | 4 | 2 | 4 | 0.00 | 2.00 |
| Chr2:180716190-180716212 | 180716207 | 3 | X | GGCCGAGGTCGACTACCGGCNRG | GGCCGAGGTgcACcACCtGtCGG | + | 5 | 0 | 4 | 0.00 | 2.00 |
| Chr3:227746244-227746266 | 227746251 | 5 | DNA | GGCCGAGGTCGAC-TACCGGCNRG | aGCacAGGTaGACTTACCaGCCGG | - | 5 | 1 | 4 | 0.00 | 2.00 |
| Chr8:38451032-38451054 | 38451049 | 3 | DNA | GGCCGAGG--TCGACTACCGGCNRG | GGgCGAGGGCTCccCTACtGGaTAG | + | 5 | 2 | 4 | 0.00 | 2.00 |
| Chr8:20852244-20852266 | 20852261 | 3 | RNA | GGCCGAGGTCGACTACCGGCNRG | aGCCGAGGgtGcCT-CgGGCTGG | + | 5 | 1 | 4 | 0.00 | 2.00 |
| Chr2:48116260-48116282 | 48116267 | 5 | DNA | GGCCGAGGTCGA-CTACCGGCNRG | cGCCGAGGTCGAGCTtCCGGCCGG | - | 2 | 1 | 4 | 0.00 | 2.00 |
| Chr1:125436389-125436411 | 125436406 | 3 | DNA | GGCCGAGGTCGACTACCGG--CNRG | GGtCGAGGTCGAgTcCgaGCCCCGG | + | 5 | 2 | 4 | 0.00 | 2.00 |
| Chr9:132705053-132705075 | 132705060 | 5 | RNA | GGCCGAGGTCGACTACCGGCNRG | G-aCGgGGTCGACaACgaGCGGG | - | 5 | 1 | 4 | 0.00 | 2.00 |
| Chr7:85009248-85009270 | 85009265 | 3 | DNA | GGCC-GAGGTCGACTACCGGCNRG | GGCCGGAGcTCGAtcACCcGgAGG | + | 5 | 1 | 4 | 0.00 | 2.00 |
| Chr2:31242370-31242392 | 31242377 | 5 | RNA | GGCCGAGGTCGACTACCGGCNRG | G-aCGgGGTCGACaACgaGCGGG | - | 5 | 1 | 4 | 0.00 | 2.00 |
| Chr4:232476389-232476411 | 232476396 | 5 | RNA | GGCCGAGGTCGACTACCGGCNRG | GcCtcgGGTCGACgAC-GGCGGG | - | 5 | 1 | 4 | 0.00 | 2.00 |
| Chr9:93973918-93973940 | 93973935 | 3 | RNA | GGCCGAGGTCGACTACCGGCNRG | G-aCGgGGTCGACgACgaGCGGG | + | 5 | 1 | 4 | 0.00 | 2.00 |
| Chr6:171250440-171250462 | 171250457 | 3 | RNA | GGCCGAGGTCGACTACCGGCNRG | G-aCGgGGTCGACgACaaGCGGG | + | 5 | 1 | 4 | 0.00 | 2.00 |
| Chr4:168158943-168158965 | 168158960 | 3 | RNA | GGCCGAGGTCGACTACCGGCNRG | GG--GtGGcgGgCTgCCGGCTGG | + | 5 | 2 | 4 | 0.00 | 2.00 |
| Chr7:19952895-19952917 | 19952902 | 5 | RNA | GGCCGAGGTCGACTACCGGCNRG | cGatGgcGTCGACT-CCGGCGAG | - | 5 | 1 | 4 | 0.00 | 2.00 |
| Chr8:97477095-97477117 | 97477102 | 5 | RNA | GGCCGAGGTCGACTACCGGCNRG | G-aCGgGGTCGACaACgaGCGGG | - | 5 | 1 | 4 | 0.00 | 2.00 |
| Chr5:26300924-26300946 | 26300941 | 3 | RNA | GGCCGAGGTCGACTACCGGCNRG | ccCCGAaGTCGAtTAtC--CCGG | + | 5 | 2 | 4 | 0.00 | 2.00 |
| Chr4:181155692-181155714 | 181155699 | 5 | RNA | GGCCGAGGTCGACTACCGGCNRG | GtCCG--GTaGAtcACCcGCAGG | - | 5 | 2 | 4 | 0.00 | 2.00 |
| Chr2:212975375-212975397 | 212975392 | 3 | RNA | GGCCGAGGTCGACTACCGGCNRG | GGCgGAGGaCa--cgCCGGCGAG | + | 5 | 2 | 4 | 0.00 | 2.00 |
| Chr7:158315712-158315734 | 158315719 | 5 | RNA | GGCCGAGGTCGACTACCGGCNRG | tGCgGgcGaC--CTACCGGCGGG | - | 5 | 2 | 4 | 0.00 | 2.00 |
| Chr2:117163140-117163162 | 117163157 | 3 | RNA | GGCCGAGGTCGACTACCGGCNRG | GGgCGAG-TCGcCTggCGGgCGG | + | 5 | 1 | 4 | 0.00 | 2.00 |
| Chr5:159362988-159363010 | 159363005 | 3 | RNA | GGCCGAGGTCGACTACCGGCNRG | GGggGAGGTgG--TAgtGGCTAG | + | 5 | 2 | 4 | 0.00 | 2.00 |
| Chr3:86696092-86696114 | 86696109 | 3 | RNA | GGCCGAGGTCGACTACCGGCNRG | tGaaGAGGc--ACTACCtGCGGG | + | 5 | 2 | 4 | 0.00 | 2.00 |
| Chr4:114353967-114353989 | 114353984 | 3 | RNA | GGCCGAGGTCGACTACCGGCNRG | G-aCGgGGTCGACaACgaGCGGG | + | 5 | 1 | 4 | 0.00 | 2.00 |
| Chr2:15249276-15249298 | 15249293 | 3 | RNA | GGCCGAGGTCGACTACCGGCNRG | GGC--tcGcgGACTACCGtCTGG | + | 5 | 2 | 4 | 0.00 | 2.00 |
| Chr2:206861186-206861208 | 206861193 | 5 | RNA | GGCCGAGGTCGACTACCGGCNRG | G-aCGgGGTCGACaACgaGCGGG | - | 5 | 1 | 4 | 0.00 | 2.00 |
| Chr7:108634793-108634815 | 108634800 | 5 | RNA | GGCCGAGGTCGACTACCGGCNRG | G-CCGAacTCaACgtCCGGCCGG | - | 5 | 1 | 4 | 0.00 | 2.00 |
| Chr10:84119606-84119628 | 84119613 | 5 | RNA | GGCCGAGGTCGACTACCGGCNRG | GGCCGAGG-CGggTAgCtcCCGG | - | 5 | 1 | 4 | 0.00 | 2.00 |
| Chr3:175881186-175881208 | 175881193 | 5 | DNA | GGCCGAGGTCGACTA--CCGGCNRG | ctCCaAGGcCGACcAATCCGGCAGG | - | 5 | 2 | 4 | 0.00 | 2.00 |
| Chr8:53735225-53735247 | 53735232 | 5 | RNA | GGCCGAGGTCGACTACCGGCNRG | GGC-GAGGTCagCTgaCGGCAGG | - | 4 | 1 | 4 | 0.00 | 2.00 |
| Chr2:48182860-48182882 | 48182867 | 5 | RNA | GGCCGAGGTCGACTACCGGCNRG | GGC--AGGgCGACcACgcGCGGG | - | 4 | 2 | 4 | 0.00 | 2.00 |
| Chr4:82988329-82988351 | 82988346 | 3 | RNA | GGCCGAGGTCGACTACCGGCNRG | G-aCGgGGTCGACaACgaGCGGG | + | 5 | 1 | 4 | 0.00 | 2.00 |
| Chr5:179004863-179004885 | 179004870 | 5 | RNA | GGCCGAGGTCGACTACCGGCNRG | GcCCGAGGaCaAC-cCCGGgCGG | - | 5 | 1 | 4 | 0.00 | 2.00 |
| Chr5:214597217-214597239 | 214597234 | 3 | RNA | GGCCGAGGTCGACTACCGGCNRG | G-aCGgGGTCGACgACgaGCGGG | + | 5 | 1 | 4 | 0.00 | 2.00 |
| Chr4:85349755-85349777 | 85349772 | 3 | RNA | GGCCGAGGTCGACTACCGGCNRG | GGCtGAGGgCaA--ACCaGgAGG | + | 5 | 2 | 4 | 0.00 | 2.00 |
| Chr5:33943533-33943555 | 33943550 | 3 | RNA | GGCCGAGGTCGACTACCGGCNRG | aaCCGAGcTCG--gAaCGGCGGG | + | 5 | 2 | 4 | 0.00 | 2.00 |
| Chr1:31715609-31715631 | 31715626 | 3 | DNA | GGCCG-AGGTCGACTACCGGCNRG | GGCCGCAGGTCaAtTAgCcGCGGG | + | 4 | 1 | 4 | 0.00 | 2.00 |
| Chr4:113471414-113471436 | 113471431 | 3 | RNA | GGCCGAGGTCGACTACCGGCNRG | G-aCGgGGTCGACgACgaGCGGG | + | 5 | 1 | 4 | 0.00 | 2.00 |
| Chr2:139524103-139524125 | 139524110 | 5 | RNA | GGCCGAGGTCGACTACCGGCNRG | tGaCGAcGTgGA--AgCGGCAGG | - | 5 | 2 | 4 | 0.00 | 2.00 |
| Chr10:23197380-23197402 | 23197387 | 5 | RNA | GGCCGAGGTCGACTACCGGCNRG | GtCttgGGT-GACcACCGGCGGG | - | 5 | 1 | 4 | 0.00 | 2.00 |
| Chr2:140990156-140990178 | 140990173 | 3 | RNA | GGCCGAGGTCGACTACCGGCNRG | GGagGAGcTCGACcACCG-gTAG | + | 5 | 1 | 4 | 0.00 | 2.00 |
| Chr5:5911337-5911359 | 5911344 | 5 | RNA | GGCCGAGGTCGACTACCGGCNRG | GGCgGcG--CGAaTcgCGGCGGG | - | 5 | 2 | 4 | 0.00 | 2.00 |
| Chr2:46513443-46513465 | 46513460 | 3 | DNA | GGCCGAG--GTCGACTACCGGCNRG | aGCtcAGCTGTCGACaACCtGCAGG | + | 5 | 2 | 4 | 0.00 | 2.00 |
| Chr10:131493815-131493837 | 131493822 | 5 | RNA | GGCCGAGGTCGACTACCGGCNRG | cGgCGAGGTCaAC--CCaGCAGG | - | 4 | 2 | 4 | 0.00 | 2.00 |
| Chr5:176620259-176620281 | 176620276 | 3 | X | GGCCGAGGTCGACTACCGGCNRG | GGaaGAGGTgGcCTAgCGGCTGG | + | 5 | 0 | 4 | 0.00 | 2.00 |
| Chr5:80606171-80606193 | 80606178 | 5 | DNA | GGCCGAGGTCGAC-TACCGGCNRG | GGCgGcGGcCaACCTACaGGCCGG | - | 5 | 1 | 4 | 0.00 | 2.00 |
| Chr2:180498762-180498784 | 180498769 | 5 | RNA | GGCCGAGGTCGACTACCGGCNRG | GGCCGAaaaCGACgAt-GGCAGG | - | 5 | 1 | 4 | 0.00 | 2.00 |
| Chr5:33265972-33265994 | 33265979 | 5 | RNA | GGCCGAGGTCGACTACCGGCNRG | GGatGAGGTgGACaA--GGCGGG | - | 4 | 2 | 4 | 0.00 | 2.00 |
| Chr10:128991903-128991925 | 128991920 | 3 | RNA | GGCCGAGGTCGACTACCGGCNRG | GGCCGAGcgCGA--AggGGgGAG | + | 5 | 2 | 4 | 0.00 | 2.00 |
| Chr2:34640592-34640614 | 34640599 | 5 | DNA | GGCCGAGGTCGACTACCGG--CNRG | GGCCGAGGTCGAgTcCgaGCCCTGG | - | 4 | 2 | 4 | 0.00 | 2.00 |
| Chr3:351798-351820 | 351805 | 5 | RNA | GGCCGAGGTCGACTACCGGCNRG | GGCtcAcG--GACTAtCGGCTGG | - | 4 | 2 | 4 | 0.00 | 2.00 |
| Chr1:162877173-162877195 | 162877180 | 5 | RNA | GGCCGAGGTCGACTACCGGCNRG | GcCCatGGcCGACTgCC--CCGG | - | 5 | 2 | 4 | 0.00 | 2.00 |
| Chr1:201320643-201320665 | 201320660 | 3 | DNA | GGCCGAGGTCGAC-TACCGGCNRG | GGCCGAGGTgaAgActCCGGCGAG | + | 5 | 1 | 4 | 0.00 | 2.00 |
| Chr1:127874646-127874668 | 127874663 | 3 | RNA | GGCCGAGGTCGACTACCGGCNRG | GGCaGcGaTgGcCT--CGGCTAG | + | 5 | 2 | 4 | 0.00 | 2.00 |
| Chr6:129825767-129825789 | 129825784 | 3 | RNA | GGCCGAGGTCGACTACCGGCNRG | GaCaG-GGTCGACaACaaGCGGG | + | 5 | 1 | 4 | 0.00 | 2.00 |
| Chr6:75850568-75850590 | 75850585 | 3 | RNA | GGCCGAGGTCGACTACCGGCNRG | GttCGAGaTCt--TACCGGaAAG | + | 5 | 2 | 4 | 0.00 | 2.00 |
| Chr2:171121862-171121884 | 171121869 | 5 | RNA | GGCCGAGGTCGACTACCGGCNRG | GGaCGA--TCGcgTAtCtGCTAG | - | 5 | 2 | 4 | 0.00 | 2.00 |
| Chr8:108234740-108234762 | 108234747 | 5 | RNA | GGCCGAGGTCGACTACCGGCNRG | GGCgGtGGgCG--TgCCGGgCGG | - | 5 | 2 | 4 | 0.00 | 2.00 |
| Chr1:46853504-46853526 | 46853521 | 3 | RNA | GGCCGAGGTCGACTACCGGCNRG | GaCgGAGGcCcACTtCC--CGAG | + | 5 | 2 | 4 | 0.00 | 2.00 |
| Chr3:221333190-221333212 | 221333207 | 3 | DNA | GGCCGAGGTCGACTACCG-GCNRG | GGCCGAaGTCGAaggCCGCtCAGG | + | 5 | 1 | 4 | 0.00 | 2.00 |
| Chr2:68901534-68901556 | 68901541 | 5 | RNA | GGCCGAGGTCGACTACCGGCNRG | GtCCG--GTgcACcACCGGaCAG | - | 5 | 2 | 4 | 0.00 | 2.00 |
| Chr2:127858470-127858492 | 127858487 | 3 | RNA | GGCCGAGGTCGACTACCGGCNRG | GGCtGgGGgCGA--ACCGagGGG | + | 5 | 2 | 4 | 0.00 | 2.00 |
| Chr1:17372208-17372230 | 17372225 | 3 | RNA | GGCCGAGGTCGACTACCGGCNRG | GGC-GAGGTCGACggCgaGaTGG | + | 5 | 1 | 4 | 0.00 | 2.00 |
| Chr6:3197598-3197620 | 3197605 | 5 | RNA | GGCCGAGGTCGACTACCGGCNRG | GGC--AGGcCGAgcACCaGCCGG | - | 4 | 2 | 4 | 0.00 | 2.00 |
| Chr1:134626818-134626840 | 134626835 | 3 | RNA | GGCCGAGGTCGACTACCGGCNRG | aGCgGAGGT-GctTACCGGgAGG | + | 5 | 1 | 4 | 0.00 | 2.00 |
| Chr5:47009621-47009643 | 47009638 | 3 | DNA | GGC--CGAGGTCGACTACCGGCNRG | GtCCTCGgGGTCGgCTcCgGGCCAG | + | 5 | 2 | 4 | 0.00 | 2.00 |
| Chr4:197050791-197050813 | 197050808 | 3 | RNA | GGCCGAGGTCGACTACCGGCNRG | GGggGtGGTCGAgaA--GGCGGG | + | 5 | 2 | 4 | 0.00 | 2.00 |
| Chr5:89093670-89093692 | 89093687 | 3 | RNA | GGCCGAGGTCGACTACCGGCNRG | GGCgGAGGTgG--TtgCGGCTGG | + | 4 | 2 | 4 | 0.00 | 2.00 |
| Chr5:100470162-100470184 | 100470179 | 3 | RNA | GGCCGAGGTCGACTACCGGCNRG | GGgtGcGGaCGAt--CCGGCCGG | + | 5 | 2 | 4 | 0.00 | 2.00 |
| Chr10:73256547-73256569 | 73256564 | 3 | RNA | GGCCGAGGTCGACTACCGGCNRG | GGaCGAcGaCGACTAC--GaTAG | + | 4 | 2 | 4 | 0.00 | 2.00 |
| Chr5:148634002-148634024 | 148634019 | 3 | DNA | GGC--CGAGGTCGACTACCGGCNRG | GGCCTCGgGGgCtACacCCGGCGGG | + | 5 | 2 | 4 | 0.00 | 2.00 |
| Chr2:175636650-175636672 | 175636657 | 5 | RNA | GGCCGAGGTCGACTACCGGCNRG | GtCCtcGG--GAtaACCGGCAGG | - | 5 | 2 | 4 | 0.00 | 2.00 |
| Chr1:123267511-123267533 | 123267518 | 5 | RNA | GGCCGAGGTCGACTACCGGCNRG | GGCCGAtcTCG--cAtCaGCAAG | - | 5 | 2 | 4 | 0.00 | 2.00 |
| Chr9:16169997-16170019 | 16170014 | 3 | RNA | GGCCGAGGTCGACTACCGGCNRG | GtCCG--GcaGAtTACCcGCAGG | + | 5 | 2 | 4 | 0.00 | 2.00 |
| Chr6:68618996-68619018 | 68619003 | 5 | DNA | GGCCGAGGTCGACTAC--CGGCNRG | GGgaGAGGTCcACgACGGCGGtGGG | - | 5 | 2 | 4 | 0.00 | 2.00 |
| Chr2:72340255-72340277 | 72340272 | 3 | RNA | GGCCGAGGTCGACTACCGGCNRG | GGCCGAtGcCGAaTgCC--aAGG | + | 5 | 2 | 4 | 0.00 | 2.00 |
| Chr8:90143521-90143543 | 90143538 | 3 | DNA | GGCCGAGGTCGA--CTACCGGCNRG | GcgCcAtcTCGATTCTACCGGCGAG | + | 5 | 2 | 4 | 0.00 | 2.00 |
| Chr4:187236831-187236853 | 187236848 | 3 | RNA | GGCCGAGGTCGACTACCGGCNRG | a--CGAGGgtGACTtCCaGCAAG | + | 5 | 2 | 4 | 0.00 | 2.00 |
| Chr8:44510252-44510274 | 44510259 | 5 | RNA | GGCCGAGGTCGACTACCGGCNRG | cGgCGAGGTCGtCTtCt--CCGG | - | 5 | 2 | 4 | 0.00 | 2.00 |
| Chr8:99886624-99886646 | 99886641 | 3 | RNA | GGCCGAGGTCGACTACCGGCNRG | GGgCGgGGTCcA-TgCCGGgCGG | + | 5 | 1 | 4 | 0.00 | 2.00 |
| Chr2:77221285-77221307 | 77221292 | 5 | RNA | GGCCGAGGTCGACTACCGGCNRG | cGaCGAGcctG--TACCGGCTGG | - | 5 | 2 | 4 | 0.00 | 2.00 |
| Chr1:27341220-27341242 | 27341227 | 5 | RNA | GGCCGAGGTCGACTACCGGCNRG | GGC--AcGcCGcCTACgGGaTGG | - | 5 | 2 | 4 | 0.00 | 2.00 |
| Chr4:103954320-103954342 | 103954337 | 3 | RNA | GGCCGAGGTCGACTACCGGCNRG | tGgCGAGG--GAggtCCGGCGAG | + | 5 | 2 | 4 | 0.00 | 2.00 |
| Chr5:110863208-110863230 | 110863215 | 5 | RNA | GGCCGAGGTCGACTACCGGCNRG | GGCgGAaGTCG--TtCgGcCGAG | - | 5 | 2 | 4 | 0.00 | 2.00 |
| Chr2:6786442-6786464 | 6786459 | 3 | RNA | GGCCGAGGTCGACTACCGGCNRG | GGC-tAGGTCGtCcACtGGtGGG | + | 5 | 1 | 4 | 0.00 | 2.00 |
| Chr10:33444934-33444956 | 33444951 | 3 | RNA | GGCCGAGGTCGACTACCGGCNRG | cGgCGAGcTCG--TACCGaCCAG | + | 4 | 2 | 4 | 0.00 | 2.00 |
| Chr10:19705090-19705112 | 19705107 | 3 | RNA | GGCCGAGGTCGACTACCGGCNRG | aGCCGAGGgtGcCT-CgGGCTGG | + | 5 | 1 | 4 | 0.00 | 2.00 |
| Chr8:136786018-136786040 | 136786035 | 3 | RNA | GGCCGAGGTCGACTACCGGCNRG | GaCCGAGcgCG--gACCGtCCGG | + | 5 | 2 | 4 | 0.00 | 2.00 |

| | | | | | | | | | | | |
|---|---|---|---|---|---|---|---|---|---|---|---|
| Chr3:73246765-73246787 | 73246772 | 5 | DNA | GGCCGAGGTCG-ACTACCGGCNRG | aGCCGAtGTCGGACcACgGcCTGG | - | 5 | 1 | 4 | 0.00 | 2.00 |
| Chr4:240094536-240094558 | 240094543 | 5 | RNA | GGCCGAGGTCGACTACCGGCNRG | GaCgGtGGTC--CTgCtGGCAAG | - | 5 | 2 | 4 | 0.00 | 2.00 |
| Chr7:147092467-147092489 | 147092474 | 5 | RNA | GGCCGAGGTCGACTACCGGCNRG | GagCGAG-TCGtCaACCGtCCAG | - | 5 | 1 | 4 | 0.00 | 2.00 |
| Chr2:123179232-123179254 | 123179239 | 5 | RNA | GGCCGAGGTCGACTACCGGCNRG | cGaCGcGGTgGgCT--CGGCTAG | - | 5 | 2 | 4 | 0.00 | 2.00 |
| Chr4:67253674-67253696 | 67253691 | 3 | RNA | GGCCGAGGTCGACTACCGGCNRG | G-aCGgGGTCGACgACgaGCGGG | + | 5 | 1 | 4 | 0.00 | 2.00 |
| Chr8:56289293-56289315 | 56289300 | 5 | RNA | GGCCGAGGTCGACTACCGGCNRG | GGCCGAtcTCG--cAtCaGCAAG | - | 5 | 2 | 4 | 0.00 | 2.00 |
| Chr4:8852307-8852329 | 8852324 | 3 | RNA | GGCCGAGGTCGACTACCGGCNRG | GGCCGAGGgCGA-gctCGGCCGG | + | 4 | 1 | 4 | 0.00 | 2.00 |
| Chr5:45238631-45238653 | 45238648 | 3 | RNA | GGCCGAGGTCGACTACCGGCNRG | cGgCGAGcT--AaTACtGGCTGG | + | 5 | 2 | 4 | 0.00 | 2.00 |
| Chr4:245727074-245727096 | 245727091 | 3 | RNA | GGCCGAGGTCGACTACCGGCNRG | GaCCGAGG-CGACgACaaGaAGG | + | 5 | 1 | 4 | 0.00 | 2.00 |
| Chr8:47229298-47229320 | 47229315 | 3 | DNA | GGCCGAGGT-CGACTACCGGCNRG | GaCgGAGaTCCGAgcACCGGCGAG | + | 5 | 1 | 4 | 0.00 | 2.00 |
| Chr3:11648339-11648361 | 11648356 | 3 | RNA | GGCCGAGGTCGACTACCGGCNRG | GGCCG-GGTCGtCTACtaGCGGG | + | 3 | 1 | 4 | 0.00 | 2.00 |
| Chr2:230278562-230278584 | 230278579 | 3 | RNA | GGCCGAGGTCGACTACCGGCNRG | GGC-GAGGTCGgCcACCaGgGAG | + | 4 | 1 | 4 | 0.00 | 2.00 |
| Chr3:138979077-138979099 | 138979084 | 5 | DNA | GGCCGAGGTCGACTAC--CGGCNRG | GcCCaAGGTCcACgACGACGGgCAG | - | 5 | 2 | 4 | 0.00 | 2.00 |
| Chr1:45965280-45965302 | 45965297 | 3 | RNA | GGCCGAGGTCGACTACCGGCNRG | G-aCGgGGTCGACaACgaGCGGG | + | 5 | 1 | 4 | 0.00 | 2.00 |
| Chr4:15893846-15893868 | 15893853 | 5 | RNA | GGCCGAGGTCGACTACCGGCNRG | GcCCGAGGcCaAC-ctCGGCGGG | - | 5 | 1 | 4 | 0.00 | 2.00 |
| Chr6:47055610-47055632 | 47055627 | 3 | RNA | GGCCGAGGTCGACTACCGGCNRG | GGC-GAGGTgGAggtCCGGCCGG | + | 4 | 1 | 4 | 0.00 | 2.00 |
| Chr2:207164952-207164974 | 207164959 | 5 | DNA | GGCCGAGGTCGACTACC--GGCNRG | GGCCGAGGcCGAgTcCgAGcGCTAG | - | 5 | 2 | 4 | 0.00 | 2.00 |
| Chr8:104457970-104457992 | 104457987 | 3 | RNA | GGCCGAGGTCGACTACCGGCNRG | GGCCcAGGTCGgtT--CtGCCAG | + | 4 | 2 | 4 | 0.00 | 2.00 |
| Chr4:98753567-98753589 | 98753574 | 5 | RNA | GGCCGAGGTCGACTACCGGCNRG | GGCgcAGtcCGACT-CCGGtAGG | - | 5 | 1 | 4 | 0.00 | 2.00 |
| Chr4:76254052-76254074 | 76254069 | 3 | RNA | GGCCGAGGTCGACTACCGGCNRG | aGCaGgGcTCGACTAC-GGaCGG | + | 5 | 1 | 4 | 0.00 | 2.00 |
| Chr7:146006310-146006332 | 146006327 | 3 | RNA | GGCCGAGGTCGACTACCGGCNRG | GGC--tcGcgGACTACCGcCTGG | + | 5 | 2 | 4 | 0.00 | 2.00 |
| Chr9:158471032-158471054 | 158471039 | 5 | RNA | GGCCGAGGTCGACTACCGGCNRG | GGaCGAGccCGACT-CtGGtTGG | - | 5 | 1 | 4 | 0.00 | 2.00 |
| Chr10:41765069-41765091 | 41765086 | 3 | RNA | GGCCGAGGTCGACTACCGGCNRG | tGCCGAGGTgGAtTgCC--tGAG | + | 5 | 2 | 4 | 0.00 | 2.00 |
| Chr4:173972750-173972772 | 173972757 | 5 | RNA | GGCCGAGGTCGACTACCGGCNRG | GGC--tcGcgGACTACCGGCTGG | - | 4 | 2 | 4 | 0.00 | 2.00 |
| Chr9:95151495-95151517 | 95151502 | 5 | RNA | GGCCGAGGTCGACTACCGGCNRG | G--CGAtGgtGACTtCgGGCGAG | - | 5 | 2 | 4 | 0.00 | 2.00 |
| Chr6:121337934-121337956 | 121337941 | 5 | DNA | GGC--CGAGGTCGACTACCGGCNRG | GGCCTCGAGGgCtACacCCGGCGGG | - | 4 | 2 | 4 | 0.00 | 2.00 |
| Chr3:135889218-135889240 | 135889235 | 3 | RNA | GGCCGAGGTCGACTACCGGCNRG | G-aCGgGGTCGACgACaaGCGGG | + | 5 | 1 | 4 | 0.00 | 2.00 |
| Chr7:74906280-74906302 | 74906297 | 3 | RNA | GGCCGAGGTCGACTACCGGCNRG | GGaCcAGGTCG--TcCCaGtTGG | + | 5 | 2 | 4 | 0.00 | 2.00 |
| Chr5:5979307-5979329 | 5979324 | 3 | RNA | GGCCGAGGTCGACTACCGGCNRG | GGC--tGGTtttgTACCGGCTGG | + | 5 | 2 | 4 | 0.00 | 2.00 |
| Chr10:48203183-48203205 | 48203190 | 5 | DNA | GGCCGAGGTCGACT--ACCGGCNRG | GGCCGAGGagGgCTGGAgtGGCCGG | - | 5 | 2 | 4 | 0.00 | 2.00 |
| Chr10:123821326-123821348 | 123821343 | 3 | DNA | GGC-CGAGGTCGACTACCGGCNRG | GcCTCGgGGaCGtCTtCCGGCTGG | + | 5 | 1 | 4 | 0.00 | 2.00 |
| Chr5:39492879-39492901 | 39492886 | 5 | RNA | GGCCGAGGTCGACTACCGGCNRG | GGgCGAGGg-GACcgCCGGtGAG | - | 5 | 1 | 4 | 0.00 | 2.00 |
| Chr10:73447433-73447455 | 73447450 | 3 | RNA | GGCCGAGGTCGACTACCGGCNRG | GGC-GtGGTCGAggACtGGCGAG | + | 4 | 1 | 4 | 0.00 | 2.00 |
| Chr8:47501040-47501062 | 47501047 | 5 | RNA | GGCCGAGGTCGACTACCGGCNRG | GGC-GtGGTCGAggACtGGtAGG | - | 5 | 1 | 4 | 0.00 | 2.00 |
| Chr1:306408702-306408724 | 306408709 | 5 | RNA | GGCCGAGGTCGACTACCGGCNRG | GGCCGAGGgC-ACctCgGGCTGG | - | 4 | 1 | 4 | 0.00 | 2.00 |
| Chr10:133023444-133023466 | 133023451 | 5 | RNA | GGCCGAGGTCGACTACCGGCNRG | GGCtGAaGTCGAC--CtGGgCGG | - | 4 | 2 | 4 | 0.00 | 2.00 |
| Chr10:67597707-67597729 | 67597724 | 3 | RNA | GGCCGAGGTCGACTACCGGCNRG | GGaaGAGGgtGA--AgCGGCTGG | + | 5 | 2 | 4 | 0.00 | 2.00 |
| Chr4:83768393-83768415 | 83768410 | 3 | RNA | GGCCGAGGTCGACTACCGGCNRG | G-CCGAcGTCGcCTtCCaGtCGG | + | 5 | 1 | 4 | 0.00 | 2.00 |
| Chr2:234081573-234081595 | 234081580 | 5 | RNA | GGCCGAGGTCGACTACCGGCNRG | GGgCGAG--CGctgACaGGCGGG | - | 5 | 2 | 4 | 0.00 | 2.00 |
| Chr2:159986500-159986522 | 159986517 | 3 | RNA | GGCCGAGGTCGACTACCGGCNRG | a--CGgGGTCGACgACgaGCGGG | + | 5 | 2 | 4 | 0.00 | 2.00 |
| Chr5:202419688-202419710 | 202419695 | 5 | RNA | GGCCGAGGTCGACTACCGGCNRG | GGCCGAGGcCGg-TgCaGGgAAG | - | 5 | 1 | 4 | 0.00 | 2.00 |
| Chr4:77960690-77960712 | 77960707 | 3 | RNA | GGCCGAGGTCGACTACCGGCNRG | G-aCGgGGTCGACaACgaGCGGG | + | 5 | 1 | 4 | 0.00 | 2.00 |
| Chr1:168924929-168924951 | 168924936 | 5 | RNA | GGCCGAGGTCGACTACCGGCNRG | GGCCGgGcaCGACgAC--GgTGG | - | 5 | 2 | 4 | 0.00 | 2.00 |
| Chr9:47542762-47542784 | 47542779 | 3 | RNA | GGCCGAGGTCGACTACCGGCNRG | GGaCGAaGaCGA--ACtaGCGAG | + | 5 | 2 | 4 | 0.00 | 2.00 |
| Chr10:58657433-58657455 | 58657440 | 5 | RNA | GGCCGAGGTCGACTACCGGCNRG | GGgCGAGcTCacCT--CGGgGGG | - | 5 | 2 | 4 | 0.00 | 2.00 |
| Chr5:45429843-45429865 | 45429850 | 5 | DNA | GGCCGAGGT-CGACTACCGGCNRG | GGCCGAGGTGCctCTtCCaGtCAG | - | 5 | 1 | 4 | 0.00 | 2.00 |
| Chr3:225236118-225236140 | 225236135 | 3 | RNA | GGCCGAGGTCGACTACCGGCNRG | GGgC-AGGcCaACccCCGGCTGG | + | 5 | 1 | 4 | 0.00 | 2.00 |
| Chr6:82232717-82232739 | 82232724 | 5 | RNA | GGCCGAGGTCGACTACCGGCNRG | GtCCGAGG--GACTAtttGaGAG | - | 5 | 2 | 4 | 0.00 | 2.00 |
| Chr8:149818340-149818362 | 149818347 | 5 | RNA | GGCCGAGGTCGACTACCGGCNRG | GtCCtcGG--GAtaACCGGCAGG | - | 5 | 2 | 4 | 0.00 | 2.00 |
| Chr6:98053960-98053982 | 98053977 | 3 | RNA | GGCCGAGGTCGACTACCGGCNRG | GGC--ttGcgGACTACCGGCTGG | + | 4 | 2 | 4 | 0.00 | 2.00 |
| Chr3:71561986-71562008 | 71562003 | 3 | RNA | GGCCGAGGTCGACTACCGGCNRG | GGCtGgGcTgGACTA--GGtTGG | + | 5 | 2 | 4 | 0.00 | 2.00 |
| Chr1:76772416-76772438 | 76772433 | 3 | RNA | GGCCGAGGTCGACTACCGGCNRG | GGCCtcGG-CGAgTgCCGGCCGG | + | 4 | 1 | 4 | 0.00 | 2.00 |
| Chr6:79695220-79695242 | 79695227 | 5 | RNA | GGCCGAGGTCGACTACCGGCNRG | cGaCGAGcTCGAC--CtGGgTAG | - | 5 | 2 | 4 | 0.00 | 2.00 |
| Chr4:182889866-182889888 | 182889883 | 3 | RNA | GGCCGAGGTCGACTACCGGCNRG | tcCCacGGTC--CTACCtGCAGG | + | 5 | 2 | 4 | 0.00 | 2.00 |
| Chr1:20533434-20533456 | 20533451 | 3 | RNA | GGCCGAGGTCGACTACCGGCNRG | GGCCGAtcTCcgCTgCCG-CCAG | + | 5 | 1 | 4 | 0.00 | 2.00 |
| Chr1:284438276-284438298 | 284438283 | 5 | RNA | GGCCGAGGTCGACTACCGGCNRG | cGCCG--GcCGACcAtCGcCAAG | - | 5 | 2 | 4 | 0.00 | 2.00 |
| Chr8:27778827-27778849 | 27778834 | 5 | RNA | GGCCGAGGTCGACTACCGGCNRG | GGCaatGGaCaACT-CCGGCGAG | - | 5 | 1 | 4 | 0.00 | 2.00 |
| Chr7:88047657-88047679 | 88047664 | 5 | RNA | GGCCGAGGTCGACTACCGGCNRG | GcCgGAGccCGAC--CCGtCAAG | - | 5 | 2 | 4 | 0.00 | 2.00 |
| Chr5:34883315-34883337 | 34883332 | 3 | RNA | GGCCGAGGTCGACTACCGGCNRG | GGCCcAGGTgtAC-AaCGGgGGG | + | 5 | 1 | 4 | 0.00 | 2.00 |
| Chr10:61211015-61211037 | 61211022 | 5 | RNA | GGCCGAGGTCGACTACCGGCNRG | GGC-GcGGTCGcggACCaGCAGG | - | 5 | 1 | 4 | 0.00 | 2.00 |
| Chr7:48103098-48103120 | 48103115 | 3 | RNA | GGCCGAGGTCGACTACCGGCNRG | cGtCGAttcCG-CTACCGGCGGG | + | 5 | 1 | 4 | 0.00 | 2.00 |
| Chr4:200397091-200397113 | 200397098 | 5 | RNA | GGCCGAGGTCGACTACCGGCNRG | GGCCGAGGcCGg-TgCgGGgAAG | - | 5 | 1 | 4 | 0.00 | 2.00 |
| Chr8:56239433-56239455 | 56239450 | 3 | RNA | GGCCGAGGTCGACTACCGGCNRG | GGCCtAtcTCG--cAtCGGCAAG | + | 5 | 2 | 4 | 0.00 | 2.00 |
| Chr4:167615394-167615416 | 167615401 | 5 | RNA | GGCCGAGGTCGACTACCGGCNRG | GGCacAGGaC-ACTACatGCAGG | - | 5 | 1 | 4 | 0.00 | 2.00 |
| Chr7:124047801-124047823 | 124047818 | 3 | RNA | GGCCGAGGTCGACTACCGGCNRG | G-aCGgGGTCGACgACgaGCGGG | + | 5 | 1 | 4 | 0.00 | 2.00 |
| Chr4:51152769-51152791 | 51152786 | 3 | RNA | GGCCGAGGTCGACTACCGGCNRG | GGCtGAGG-CaAggACCGGgGAG | + | 5 | 1 | 4 | 0.00 | 2.00 |
| Chr9:2720314-2720336 | 2720321 | 5 | RNA | GGCCGAGGTCGACTACCGGCNRG | GGC--AGGcCaACctCCGGCCGG | - | 4 | 2 | 4 | 0.00 | 2.00 |
| Chr4:163685203-163685225 | 163685220 | 3 | RNA | GGCCGAGGTCGACTACCGGCNRG | aGCCGtGG-CGtCatCCGGCCGG | + | 5 | 1 | 4 | 0.00 | 2.00 |
| Chr2:58624502-58624524 | 58624519 | 3 | RNA | GGCCGAGGTCGACTACCGGCNRG | GGCCGAGtaCG--TtgCcGCAGG | + | 5 | 2 | 4 | 0.00 | 2.00 |
| Chr10:23982545-23982567 | 23982552 | 5 | RNA | GGCCGAGGTCGACTACCGGCNRG | GGCaccGGTCGgCTtCC--CAGG | - | 5 | 2 | 4 | 0.00 | 2.00 |
| Chr10:36872891-36872913 | 36872898 | 5 | RNA | GGCCGAGGTCGACTACCGGCNRG | GGCgtAGGcCGACg--CGGtGGG | - | 5 | 2 | 4 | 0.00 | 2.00 |
| Chr4:102066013-102066035 | 102066020 | 5 | RNA | GGCCGAGGTCGACTACCGGCNRG | GGCtatGGTCGA--ACCGaCGGG | - | 4 | 2 | 4 | 0.00 | 2.00 |
| Chr5:37970769-37970791 | 37970776 | 5 | RNA | GGCCGAGGTCGACTACCGGCNRG | GGgCGAGcTCGgCTAg-GGCTGG | - | 4 | 1 | 4 | 0.00 | 2.00 |
| Chr10:3393507-3393529 | 3393524 | 3 | RNA | GGCCGAGGTCGACTACCGGCNRG | G-aCGgGGTCGACaACgaGCGGG | + | 5 | 1 | 4 | 0.00 | 2.00 |
| Chr1:43654628-43654650 | 43654635 | 5 | RNA | GGCCGAGGTCGACTACCGGCNRG | GGCgGAaGTCGtC-gCCGGtGAG | - | 5 | 1 | 4 | 0.00 | 2.00 |
| Chr8:99774357-99774379 | 99774374 | 3 | RNA | GGCCGAGGTCGACTACCGGCNRG | GGCCctGGTtGAC-AgCcGCGGG | + | 5 | 1 | 4 | 0.00 | 2.00 |
| Chr5:70344497-70344519 | 70344504 | 5 | RNA | GGCCGAGGTCGACTACCGGCNRG | cGCCaAGGgtGACTAC--GaCGG | - | 5 | 2 | 4 | 0.00 | 2.00 |
| Chr4:132130720-132130742 | 132130727 | 5 | RNA | GGCCGAGGTCGACTACCGGCNRG | GGCgGAGtTCGtCTtCC--gGGG | - | 5 | 2 | 4 | 0.00 | 2.00 |
| Chr1:185121778-185121800 | 185121785 | 5 | RNA | GGCCGAGGTCGACTACCGGCNRG | tGCtGAGGTgGAt--CtGGCCGG | - | 5 | 2 | 4 | 0.00 | 2.00 |
| Chr4:210483114-210483136 | 210483131 | 3 | RNA | GGCCGAGGTCGACTACCGGCNRG | cttCtAGGT-GACcACCGGCGGG | + | 5 | 1 | 4 | 0.00 | 2.00 |
| Chr8:23101791-23101813 | 23101798 | 5 | RNA | GGCCGAGGTCGACTACCGGCNRG | GGaCcAGGTCGgggAC--GCGGG | - | 5 | 2 | 4 | 0.00 | 2.00 |
| Chr2:126141962-126141984 | 126141969 | 5 | RNA | GGCCGAGGTCGACTACCGGCNRG | GGCCG--GTCcACggtgGGCGGG | - | 5 | 2 | 4 | 0.00 | 2.00 |
| Chr8:135158180-135158202 | 135158187 | 5 | RNA | GGCCGAGGTCGACTACCGGCNRG | GGgCGAGGcCGAt--CgGGtGGG | - | 5 | 2 | 4 | 0.00 | 2.00 |
| Chr7:148800280-148800302 | 148800287 | 5 | RNA | GGCCGAGGTCGACTACCGGCNRG | G-aCGAaGatGACTACgGGCCGG | - | 5 | 1 | 4 | 0.00 | 2.00 |
| Chr2:132303340-132303362 | 132303347 | 5 | RNA | GGCCGAGGTCGACTACCGGCNRG | GtCCG--GTgcACcACCGGaCAG | - | 5 | 2 | 4 | 0.00 | 2.00 |
| Chr9:112468027-112468049 | 112468034 | 5 | RNA | GGCCGAGGTCGACTACCGGCNRG | GGC--AGGcCaACctCCGGCCGG | - | 4 | 2 | 4 | 0.00 | 2.00 |
| Chr10:85028172-85028194 | 85028189 | 3 | RNA | GGCCGAGGTCGACTACCGGCNRG | GGgCG-GGcCaACctCCGGCCGG | + | 5 | 1 | 4 | 0.00 | 2.00 |
| Chr8:53635449-53635471 | 53635456 | 5 | RNA | GGCCGAGGTCGACTACCGGCNRG | GGCCGtGGcCGA-ggCCGtCGGG | - | 5 | 1 | 4 | 0.00 | 2.00 |
| Chr1:244737668-244737690 | 244737675 | 5 | RNA | GGCCGAGGTCGACTACCGGCNRG | tG--GAGGatGcCTACCGGaTAG | - | 5 | 2 | 4 | 0.00 | 2.00 |
| Chr5:140616156-140616178 | 140616173 | 3 | RNA | GGCCGAGGTCGACTACCGGCNRG | GGCaGAG--CGACagagGGCGAG | + | 5 | 2 | 4 | 0.00 | 2.00 |
| Chr2:46058366-46058388 | 46058373 | 5 | DNA | GGCCGAGGTCGACT--ACCGGCNRG | GGtCGtGGTCGcCTGCACCGGgTGG | - | 4 | 2 | 4 | 0.00 | 2.00 |
| Chr9:117900391-117900413 | 117900408 | 3 | RNA | GGCCGAGGTCGACTACCGGCNRG | GGaCGAGa-CGACTtaaGGCAGG | + | 5 | 1 | 4 | 0.00 | 2.00 |
| Chr1:184583020-184583042 | 184583027 | 5 | RNA | GGCCGAGGTCGACTACCGGCNRG | G-aCGgGGTCGACaACgaGCGGG | - | 5 | 1 | 4 | 0.00 | 2.00 |
| Chr10:97034593-97034615 | 97034610 | 3 | RNA | GGCCGAGGTCGACTACCGGCNRG | GcCCGAGGcCaAC-cCCGGgCGG | + | 5 | 1 | 4 | 0.00 | 2.00 |
| Chr5:207541741-207541763 | 207541748 | 5 | RNA | GGCCGAGGTCGACTACCGGCNRG | GGC--AGGcCGAgcACCaGCCGG | - | 4 | 2 | 4 | 0.00 | 2.00 |
| Chr9:141568180-141568202 | 141568187 | 5 | RNA | GGCCGAGGTCGACTACCGGCNRG | cGgCGAGGgCGACT--CcGgCGG | - | 5 | 2 | 4 | 0.00 | 2.00 |
| Chr4:146716386-146716408 | 146716393 | 5 | DNA | GGC-CGAGGTCGACTACCGGCNRG | GcCTCGgGGaCGtCTtCCGGCTGG | - | 5 | 1 | 4 | 0.00 | 2.00 |
| Chr8:67100173-67100195 | 67100190 | 3 | DNA | GGCCGAGGT--CGACTACCGGCNRG | GtaCGAGGgAACttCTACCGGCAGG | + | 5 | 2 | 4 | 0.00 | 2.00 |
| Chr10:69819188-69819210 | 69819204 | 4 | RNA | GGCCGAGGTCGACTACCGGCNRG | G-aCGgGGTCGACaACgaGCGGG | + | 5 | 1 | 0 | 3.86 | 1.93 |
| Chr2:228531948-228531970 | 228531965 | 3 | RNA | GGCCGAGGTCGACTACCGGCNRG | caCCGAcGT-GAtcACCGGCAAG | + | 5 | 1 | 0 | 3.86 | 1.93 |
| Chr10:72184785-72184807 | 72184792 | 5 | RNA | GGCCGAGGTCGACTACCGGCNRG | cGaCGAGGgCGACgAC-GaCAAG | - | 5 | 1 | 0 | 3.86 | 1.93 |
| Chr4:235263542-235263564 | 235263548 | 4 | RNA | GGCCGAGGTCGACTACCGGCNRG | GGCCGAG--CGggTcaCGGCGAG | - | 4 | 2 | 0 | 3.86 | 1.93 |
| Chr1:217753270-217753292 | 217753276 | 4 | DNA | GGC-CGAGGTCGACTACCGGCNRG | GcCTCGgGGaCGtCTtCCGGCTGG | - | 5 | 1 | 0 | 3.86 | 1.93 |
| Chr2:3196718-3196740 | 3196734 | 4 | RNA | GGCCGAGGTCGACTACCGGCNRG | GGCCacGG-CGgCTgCtGGCCGG | + | 5 | 1 | 0 | 3.86 | 1.93 |
| Chr2:21986432-21986454 | 21986436 | 2 | RNA | GGCCGAGGTCGACTACCGGCNRG | GcCgGAGccCGAC--CCGtCAAG | - | 5 | 2 | 0 | 3.86 | 1.93 |
| Chr2:205534476-205534498 | 205534482 | 4 | RNA | GGCCGAGGTCGACTACCGGCNRG | GGggGAGGTgG--TAgaGGCTAG | - | 5 | 2 | 0 | 3.86 | 1.93 |
| Chr7:76522128-76522150 | 76522134 | 4 | RNA | GGCCGAGGTCGACTACCGGCNRG | GGgCGAG--CGctgACaGGCAGG | - | 5 | 2 | 0 | 3.86 | 1.93 |
| Chr8:148286517-148286539 | 148286524 | 5 | RNA | GGCCGAGGTCGACTACCGGCNRG | GGC--AGGcCGAgcACCGGCCGG | - | 3 | 2 | 0 | 3.86 | 1.93 |
| Chr3:57289981-57290003 | 57289986 | 3 | RNA | GGCCGAGGTCGACTACCGGCNRG | GGC--AGGcCGAgcACCaGCCGG | - | 4 | 2 | 0 | 3.86 | 1.93 |
| Chr2:109842051-109842073 | 109842057 | 4 | RNA | GGCCGAGGTCGACTACCGGCNRG | atCCGAGtgCGA-TACCtGCCAG | - | 5 | 1 | 0 | 3.86 | 1.93 |
| Chr1:1321583-1321605 | 1321600 | 3 | DNA | GGCCGAGGTCGACTACC--GGCNRG | GGCgGAGGTCGtCggCaGAGGCCAG | + | 5 | 2 | 0 | 3.86 | 1.93 |
| Chr9:96883990-96884012 | 96884005 | 5 | DNA | GGCCGAGGTCGACTA--CCGGCNRG | GcCCGAcaTCGcCTACGCCGtCCAG | + | 5 | 2 | 0 | 3.86 | 1.93 |
| Chr1:72990496-72990518 | 72990501 | 3 | RNA | GGCCGAGGTCGACTACCGGCNRG | GGC--tcGcgGACTACCGGCTGG | - | 4 | 2 | 0 | 3.86 | 1.93 |
| Chr7:12939738-12939760 | 12939755 | 3 | RNA | GGCCGAGGTCGACTACCGGCNRG | GGCCGAc--CGACgAgaGGaGAG | + | 5 | 2 | 0 | 3.86 | 1.93 |
| Chr1:117576424-117576446 | 117576442 | 2 | RNA | GGCCGAGGTCGACTACCGGCNRG | GGgCGgGGTtGgCT--CGGaGAG | + | 5 | 2 | 0 | 3.86 | 1.93 |
| Chr4:24866793-24866815 | 24866811 | 2 | RNA | GGCCGAGGTCGACTACCGGCNRG | GGCCGAGcgCGA--AggGGgGAG | + | 5 | 2 | 0 | 3.86 | 1.93 |
| Chr10:112563692-112563714 | 112563707 | 5 | RNA | GGCCGAGGTCGACTACCGGCNRG | GGCCGAGGTCGAgctgCt-CCGG | + | 5 | 1 | 0 | 3.86 | 1.93 |
| Chr5:143946244-143946266 | 143946260 | 4 | DNA | GGC-CGAGGTCGACTACCGGCNRG | GcCTCGgGGaCGtCTtCCGGCTGG | + | 5 | 1 | 0 | 3.86 | 1.93 |
| Chr10:146316369-146316391 | 146316386 | 3 | RNA | GGCCGAGGTCGACTACCGGCNRG | caCCGAGtTgGAgTA--GGCGGG | + | 5 | 2 | 0 | 3.86 | 1.93 |
| Chr4:180298181-180298203 | 180298199 | 2 | RNA | GGCCGAGGTCGACTACCGGCNRG | tGgCGAGcTCGACcA--GGaCAG | + | 5 | 2 | 0 | 3.86 | 1.93 |
| Chr2:205730744-205730766 | 205730762 | 2 | RNA | GGCCGAGGTCGACTACCGGCNRG | GGgtcgGGTCGA--AgCGGCAGG | + | 5 | 2 | 0 | 3.86 | 1.93 |
| Chr10:89464995-89465017 | 89465000 | 3 | RNA | GGCCGAGGTCGACTACCGGCNRG | G-aCGgGGTCGACgACgaGCGGG | - | 5 | 1 | 0 | 3.86 | 1.93 |
| Chr4:84113897-84113919 | 84113914 | 3 | RNA | GGCCGAGGTCGACTACCGGCNRG | GGC--AtGcCGAgcACCGGCCGG | + | 4 | 2 | 0 | 3.86 | 1.93 |
| Chr2:27616155-27616177 | 27616170 | 5 | RNA | GGCCGAGGTCGACTACCGGCNRG | GGCacAGGaC-ACTACaGGCAGG | + | 4 | 1 | 0 | 3.86 | 1.93 |
| Chr4:144484666-144484688 | 144484673 | 5 | RNA | GGCCGAGGTCGACTACCGGCNRG | GGCtGccG-CGACcACCGGaTGG | - | 5 | 1 | 0 | 3.86 | 1.93 |
| Chr6:75123957-75123979 | 75123962 | 3 | RNA | GGCCGAGGTCGACTACCGGCNRG | c-CCGgGtTCGAtTcCCGGCTGG | - | 5 | 1 | 0 | 3.86 | 1.93 |

| | | | | | | | | | | | |
|---|---|---|---|---|---|---|---|---|---|---|---|
| Chr8:44190575-44190597 | 44190581 | 4 | X | GGCCGAGGTCGACTACCGGCNRG | GGCCtcGGgCGAgTACtGGCAGG | - | 5 | 0 | 0 | 3.86 | 1.93 |
| Chr6:160678466-160678488 | 160678472 | 4 | RNA | GGCCGAGGTCGACTACCGGCNRG | GGCCGtGGcCGtCTgCC--aAGG | - | 5 | 2 | 0 | 3.86 | 1.93 |
| Chr3:74291805-74291827 | 74291823 | 2 | RNA | GGCCGAGGTCGACTACCGGCNRG | GG--GtGGTCGAagtCCGGtGAG | + | 5 | 2 | 0 | 3.86 | 1.93 |
| Chr9:18823314-18823336 | 18823319 | 3 | DNA | GGCC--GAGGTCGACTACCGGCNRG | GGCCCTGAGtTCGAaTcCCaGtGAG | - | 5 | 2 | 0 | 3.86 | 1.93 |
| Chr8:177983553-177983575 | 177983571 | 2 | DNA | GGCCGAGGTCGACT--ACCGGCNRG | cGCgGAaGcCaACTCCACCGGCAGG | + | 5 | 2 | 0 | 3.86 | 1.93 |
| Chr1:127347576-127347598 | 127347593 | 3 | RNA | GGCCGAGGTCGACTACCGGCNRG | GagCGAG-TCGtCaACCGtCCAG | + | 5 | 1 | 0 | 3.86 | 1.93 |
| Chr4:155736638-155736660 | 155736644 | 4 | RNA | GGCCGAGGTCGACTACCGGCNRG | GGaCcAGGTCG--TcCCaGtTGG | - | 5 | 2 | 0 | 3.86 | 1.93 |
| Chr7:144874097-144874119 | 144874102 | 3 | RNA | GGCCGAGGTCGACTACCGGCNRG | GctCGAaGgCGA--ACCGGCGGG | - | 4 | 2 | 0 | 3.86 | 1.93 |
| Chr4:214892603-214892625 | 214892607 | 2 | RNA | GGCCGAGGTCGACTACCGGCNRG | GtCgGAGcTCGACTgC--GCCAG | - | 4 | 2 | 0 | 3.86 | 1.93 |
| Chr2:43582282-43582304 | 43582289 | 5 | RNA | GGCCGAGGTCGACTACCGGCNRG | GGaCGAacT--ACctCCGGCCGG | - | 5 | 2 | 0 | 3.86 | 1.93 |
| Chr4:111778063-111778085 | 111778080 | 3 | DNA | GGCCGAGGTCGACTACCG--GCNRG | GGCCGgGGTCGgCccaCGGCGCGGG | + | 5 | 2 | 0 | 3.86 | 1.93 |
| Chr7:66574006-66574028 | 66574021 | 5 | RNA | GGCCGAGGTCGACTACCGGCNRG | GGC--tGcTCGACaACCacCAAG | + | 5 | 2 | 0 | 3.86 | 1.93 |
| Chr7:26177613-26177635 | 26177629 | 4 | RNA | GGCCGAGGTCGACTACCGGCNRG | GGCCGAGGTgGgC-cgaGGCGGG | + | 5 | 1 | 0 | 3.86 | 1.93 |
| Chr2:233522922-233522944 | 233522926 | 2 | DNA | GGC--CGAGGTCGACTACCGGCNRG | GGCCTCGgGGgCtACacCCGGCGGG | - | 5 | 2 | 0 | 3.86 | 1.93 |
| Chr6:45111647-45111669 | 45111662 | 5 | RNA | GGCCGAGGTCGACTACCGGCNRG | GGCtGgGcTgGACTA--GGtTGG | + | 5 | 2 | 0 | 3.86 | 1.93 |
| Chr4:68139987-68140009 | 68140004 | 3 | DNA | GGCCGAGG--TCGACTACCGGCNRG | GtaCGAcGCCTCGACTcCCtGCGGG | + | 5 | 2 | 0 | 3.86 | 1.93 |
| Chr10:3395075-3395097 | 3395081 | 4 | RNA | GGCCGAGGTCGACTACCGGCNRG | GGgCG-GGcCaACctCCGGCCGG | - | 5 | 1 | 0 | 3.86 | 1.93 |
| Chr2:112130552-112130574 | 112130570 | 2 | RNA | GGCCGAGGTCGACTACCGGCNRG | GGCCGAGcgCGA--AggGGgAAG | + | 5 | 2 | 0 | 3.86 | 1.93 |
| Chr2:38389569-38389591 | 38389585 | 4 | RNA | GGCCGAGGTCGACTACCGGCNRG | GaCCGAGG-CGACgACaaGaAGG | + | 5 | 1 | 0 | 3.86 | 1.93 |
| Chr2:206036552-206036574 | 206036568 | 4 | RNA | GGCCGAGGTCGACTACCGGCNRG | G-aCGgGGTCGACgACgaGCGGG | + | 5 | 1 | 0 | 3.86 | 1.93 |
| Chr8:95939883-95939905 | 95939898 | 5 | RNA | GGCCGAGGTCGACTACCGGCNRG | cGgCGAGG--GAggtCCGGCGAG | + | 5 | 2 | 0 | 3.86 | 1.93 |
| Chr10:31129302-31129324 | 31129307 | 3 | RNA | GGCCGAGGTCGACTACCGGCNRG | GGCCGgGGcgGgC--CgGGCCGG | - | 5 | 2 | 0 | 3.86 | 1.93 |
| Chr1:114440489-114440511 | 114440505 | 4 | RNA | GGCCGAGGTCGACTACCGGCNRG | atCCtgGGT-GACTgCCGGCGGG | + | 5 | 1 | 0 | 3.86 | 1.93 |
| Chr5:171474749-171474771 | 171474764 | 5 | RNA | GGCCGAGGTCGACTACCGGCNRG | GGgaGAGGggGACaA--GGCCGG | + | 5 | 2 | 0 | 3.86 | 1.93 |
| Chr6:133846521-133846543 | 133846538 | 3 | RNA | GGCCGAGGTCGACTACCGGCNRG | GaCCGAcGaCGA-gAgCGGCTGG | + | 5 | 1 | 0 | 3.86 | 1.93 |
| Chr4:188949668-188949690 | 188949673 | 3 | RNA | GGCCGAGGTCGACTACCGGCNRG | GGC--tcGcgGAtTACCGGCTGG | - | 5 | 2 | 0 | 3.86 | 1.93 |
| Chr6:63861717-63861739 | 63861734 | 3 | RNA | GGCCGAGGTCGACTACCGGCNRG | GG--GcGGcCGAagtCCGGCGAG | + | 5 | 2 | 0 | 3.86 | 1.93 |
| Chr4:70742597-70742619 | 70742604 | 5 | DNA | GGCCGAGGTCGACTAC--CGGCNRG | GGCgGgtGTtGAaTACTACGGCTAG | - | 5 | 2 | 0 | 3.86 | 1.93 |
| Chr9:87693730-87693752 | 87693737 | 5 | RNA | GGCCGAGGTCGACTACCGGCNRG | GGCCGAGGgtGcCT-CgGGCCAG | - | 4 | 1 | 0 | 3.86 | 1.93 |
| Chr5:115279670-115279692 | 115279675 | 3 | RNA | GGCCGAGGTCGACTACCGGCNRG | GaCaG-GGTCGACaACgaGCGGG | - | 5 | 1 | 0 | 3.86 | 1.93 |
| Chr7:115193485-115193507 | 115193491 | 4 | DNA | GGCCGAGG--TCGACTACCGGCNRG | GGCtGAGGGCTCagCcACgGGCTAG | - | 5 | 2 | 0 | 3.86 | 1.93 |
| Chr2:117480301-117480323 | 117480319 | 2 | RNA | GGCCGAGGTCGACTACCGGCNRG | GtCCG--GTgcACcACCGGaCAG | + | 5 | 2 | 0 | 3.86 | 1.93 |
| Chr7:112568374-112568396 | 112568389 | 5 | DNA | GGCCGAGGTCGACTA--CCGGCNRG | ctCCaAGGcCGACcAATCCGGCAGG | + | 5 | 2 | 0 | 3.86 | 1.93 |
| Chr4:226154453-226154475 | 226154471 | 2 | RNA | GGCCGAGGTCGACTACCGGCNRG | GGaCGAGGcgtACaAC--GCTAG | + | 5 | 2 | 0 | 3.86 | 1.93 |
| Chr7:100627431-100627453 | 100627437 | 4 | RNA | GGCCGAGGTCGACTACCGGCNRG | cGtCatGGTCGgCT--CGGCGAG | - | 5 | 2 | 0 | 3.86 | 1.93 |
| Chr2:184513413-184513435 | 184513419 | 4 | DNA | GGCCGAGGTCGACTACCGG--CNRG | GGCCGAGGcCGAgTcCgaGCCCTGG | - | 5 | 2 | 0 | 3.86 | 1.93 |
| Chr5:101166180-101166202 | 101166186 | 4 | RNA | GGCCGAGGTCGACTACCGGCNRG | GGC--tcGcgGACTACCGcCTGG | - | 5 | 2 | 0 | 3.86 | 1.93 |
| Chr6:100461764-100461786 | 100461782 | 2 | RNA | GGCCGAGGTCGACTACCGGCNRG | GGagGgaGTaG--TACCGGCAGG | + | 5 | 2 | 0 | 3.86 | 1.93 |
| Chr10:59328656-59328678 | 59328671 | 5 | RNA | GGCCGAGGTCGACTACCGGCNRG | GGC--AGGcCGAgcACttGCCGG | + | 5 | 2 | 0 | 3.86 | 1.93 |
| Chr6:40894804-40894826 | 40894809 | 3 | DNA | GGCCGAGGTCGACTA--CCGGCNRG | cGCaaAGGcCGACcAATCCGGCAGG | - | 5 | 2 | 0 | 3.86 | 1.93 |
| Chr2:130749520-130749542 | 130749537 | 3 | RNA | GGCCGAGGTCGACTACCGGCNRG | GaCCGAGcTCGAaTg--GaCCAG | + | 5 | 2 | 0 | 3.86 | 1.93 |
| Chr8:148276540-148276562 | 148276555 | 5 | RNA | GGCCGAGGTCGACTACCGGCNRG | GGCgGAGtTCGtCTtCC--gAGG | + | 5 | 2 | 0 | 3.86 | 1.93 |
| Chr3:214814567-214814589 | 214814585 | 2 | RNA | GGCCGAGGTCGACTACCGGCNRG | GGCCGAGtgCGA--AggGGgGAG | + | 5 | 2 | 0 | 3.86 | 1.93 |
| Chr9:8615474-8615496 | 8615490 | 4 | RNA | GGCCGAGGTCGACTACCGGCNRG | GG--GcGGcCGtggACCGGCGAG | + | 5 | 2 | 0 | 3.86 | 1.93 |
| Chr10:58208907-58208929 | 58208922 | 5 | RNA | GGCCGAGGTCGACTACCGGCNRG | GagaGAGaTC--CTAaCGGCCGG | + | 5 | 2 | 0 | 3.86 | 1.93 |
| Chr7:86175542-86175564 | 86175546 | 2 | RNA | GGCCGAGGTCGACTACCGGCNRG | GGC--tcGcgGACTACCGGCTGG | - | 4 | 2 | 0 | 3.86 | 1.93 |
| Chr2:68949268-68949290 | 68949284 | 4 | RNA | GGCCGAGGTCGACTACCGGCNRG | GGCCaAatT-GgCTACaGGCAGG | + | 5 | 1 | 0 | 3.86 | 1.93 |
| Chr2:173491681-173491703 | 173491699 | 2 | RNA | GGCCGAGGTCGACTACCGGCNRG | GGgCG-GGcCaACctCCGGCTGG | + | 5 | 1 | 0 | 3.86 | 1.93 |
| Chr3:52895550-52895572 | 52895555 | 3 | RNA | GGCCGAGGTCGACTACCGGCNRG | tGCCG--GTgGACcACtGtCCAG | - | 5 | 2 | 0 | 3.86 | 1.93 |
| Chr1:98839477-98839499 | 98839482 | 3 | RNA | GGCCGAGGTCGACTACCGGCNRG | GGCgcgGGTCGACgt--GGCAGG | - | 5 | 2 | 0 | 3.86 | 1.93 |
| Chr1:182626386-182626408 | 182626401 | 5 | RNA | GGCCGAGGTCGACTACCGGCNRG | a--CGgGGTCGACgACgaGCGGG | + | 5 | 2 | 0 | 3.86 | 1.93 |
| Chr2:146523658-146523680 | 146523662 | 2 | RNA | GGCCGAGGTCGACTACCGGCNRG | tGCtGAaGgC--CTAgCGGCGGG | - | 5 | 2 | 0 | 3.86 | 1.93 |
| Chr2:172138984-172139006 | 172138991 | 5 | RNA | GGCCGAGGTCGACTACCGGCNRG | GGCCGAGGat-ACctCgGGCTGG | - | 5 | 1 | 0 | 3.86 | 1.93 |
| Chr2:126110818-126110840 | 126110836 | 2 | RNA | GGCCGAGGTCGACTACCGGCNRG | aGggGAGaTgGACTAC-GGCTAG | + | 5 | 1 | 0 | 3.86 | 1.93 |
| Chr4:243745475-243745497 | 243745480 | 3 | DNA | GGC--CGAGGTCGACTACCGGCNRG | GaCAACGgGGTCGACgACgaGCGGG | - | 5 | 2 | 0 | 3.86 | 1.93 |
| Chr1:173966036-173966058 | 173966054 | 2 | RNA | GGCCGAGGTCGACTACCGGCNRG | GGaCGAcGaCGACTAC--GaTAG | + | 4 | 2 | 0 | 3.86 | 1.93 |
| Chr10:82234963-82234985 | 82234969 | 4 | RNA | GGCCGAGGTCGACTACCGGCNRG | GGgCG-GGcCaACTtCCGGCCGG | - | 4 | 1 | 0 | 3.86 | 1.93 |
| Chr2:227378348-227378370 | 227378366 | 2 | RNA | GGCCGAGGTCGACTACCGGCNRG | GG--GAGGTCGgCTtCgtGtGAG | + | 5 | 2 | 0 | 3.86 | 1.93 |
| Chr4:118394836-118394858 | 118394851 | 5 | RNA | GGCCGAGGTCGACTACCGGCNRG | GGCtGcGcTCGgCTA--GGtAGG | + | 5 | 2 | 0 | 3.86 | 1.93 |
| Chr1:120162202-120162224 | 120162217 | 5 | RNA | GGCCGAGGTCGACTACCGGCNRG | GGC-GAGGTgGAgcACgGGgTAG | + | 5 | 1 | 0 | 3.86 | 1.93 |
| Chr10:98826797-98826819 | 98826814 | 3 | DNA | GGC-CGAGGTCGACTACCGGCNRG | GcCTCGAGGaCatCTtCCGGCTGG | + | 5 | 1 | 0 | 3.86 | 1.93 |
| Chr1:30188569-30188591 | 30188584 | 5 | RNA | GGCCGAGGTCGACTACCGGCNRG | GGC--tcGcgGACTACCGGCTGG | + | 4 | 2 | 0 | 3.86 | 1.93 |
| Chr2:88113739-88113761 | 88113743 | 2 | RNA | GGCCGAGGTCGACTACCGGCNRG | GGCttAGGgaGACT-CCGGtTGG | - | 5 | 1 | 0 | 3.86 | 1.93 |
| Chr7:72631088-72631110 | 72631095 | 5 | RNA | GGCCGAGGTCGACTACCGGCNRG | GGCCGAGcT-GcCgAaCGcCTGG | - | 5 | 1 | 0 | 3.86 | 1.93 |
| Chr7:166689941-166689963 | 166689957 | 4 | RNA | GGCCGAGGTCGACTACCGGCNRG | aGCCGAGGgtGcCT--CGGgTAG | + | 5 | 2 | 0 | 3.86 | 1.93 |
| Chr3:70168578-70168600 | 70168583 | 3 | RNA | GGCCGAGGTCGACTACCGGCNRG | aGCCGAa--CGAtgACtGGCTAG | - | 5 | 2 | 0 | 3.86 | 1.93 |
| Chr5:211550440-211550462 | 211550455 | 5 | RNA | GGCCGAGGTCGACTACCGGCNRG | GGC-aAGGTCGACagCCGagGAG | + | 5 | 1 | 0 | 3.86 | 1.93 |
| Chr2:84923420-84923442 | 84923435 | 5 | RNA | GGCCGAGGTCGACTACCGGCNRG | GGCCG-GGTCGtCcACtGGCGGG | + | 3 | 1 | 0 | 3.86 | 1.93 |
| Chr3:145466295-145466317 | 145466302 | 5 | RNA | GGCCGAGGTCGACTACCGGCNRG | GGCCGAaaaCGACgAt-GGCAGG | - | 5 | 1 | 0 | 3.86 | 1.93 |
| Chr1:234790404-234790426 | 234790410 | 4 | RNA | GGCCGAGGTCGACTACCGGCNRG | GtCCtcGG--GAtaACCGGCAGG | - | 5 | 2 | 0 | 3.86 | 1.93 |
| Chr4:109963533-109963555 | 109963537 | 2 | RNA | GGCCGAGGTCGACTACCGGCNRG | GGCCGAtcTCG--cAtCaGCAAG | - | 5 | 2 | 0 | 3.86 | 1.93 |
| Chr7:18440241-18440263 | 18440256 | 5 | RNA | GGCCGAGGTCGACTACCGGCNRG | caCCaAGtTCGcCT-CCGGCCAG | + | 5 | 1 | 0 | 3.86 | 1.93 |
| Chr1:22291976-22291998 | 22291992 | 4 | RNA | GGCCGAGGTCGACTACCGGCNRG | GGtCG-GGTCGtCcACtGGCGGG | + | 4 | 1 | 0 | 3.86 | 1.93 |
| Chr9:43305404-43305426 | 43305411 | 5 | RNA | GGCCGAGGTCGACTACCGGCNRG | GctCGA--TCGACTcatGGCCGG | - | 5 | 2 | 0 | 3.86 | 1.93 |
| Chr4:206040861-206040883 | 206040877 | 4 | RNA | GGCCGAGGTCGACTACCGGCNRG | G-CCtcGGgCGAgTACtGGCAGG | + | 5 | 1 | 0 | 3.86 | 1.93 |
| Chr2:112143688-112143710 | 112143704 | 4 | RNA | GGCCGAGGTCGACTACCGGCNRG | G-aCGgGGTCGACgACgaGCGGG | + | 5 | 1 | 0 | 3.86 | 1.93 |
| Chr8:106126726-106126748 | 106126731 | 3 | RNA | GGCCGAGGTCGACTACCGGCNRG | GtCaGAGaTCG--TACaGGCCAG | - | 4 | 2 | 0 | 3.86 | 1.93 |
| Chr2:2036572-2036594 | 2036578 | 4 | RNA | GGCCGAGGTCGACTACCGGCNRG | GGCgGAcGTgGAC-AaCGaCCAG | - | 5 | 1 | 0 | 3.86 | 1.93 |
| Chr5:15367646-15367668 | 15367653 | 5 | RNA | GGCCGAGGTCGACTACCGGCNRG | GGCCaAGGgCcACTACac-CGAG | - | 5 | 1 | 0 | 3.86 | 1.93 |
| Chr2:16626963-16626985 | 16626981 | 2 | RNA | GGCCGAGGTCGACTACCGGCNRG | GtCCG--GTgcACcACCGGgCAG | + | 5 | 2 | 0 | 3.86 | 1.93 |
| Chr9:52520696-52520718 | 52520714 | 2 | DNA | GGCCGAGGT--CGACTACCGGCNRG | GGCCGAcGTGGCcACTAaCcGtTGG | + | 5 | 2 | 0 | 3.86 | 1.93 |
| Chr7:33158870-33158892 | 33158876 | 4 | X | GGCCGAGGTCGACTACCGGCNRG | GGaCGAcGaCGACTAgCGGtAGG | - | 5 | 0 | 0 | 3.86 | 1.93 |
| Chr9:37991745-37991767 | 37991752 | 5 | RNA | GGCCGAGGTCGACTACCGGCNRG | GtCCG--GTgcACcACCGGaCAG | - | 5 | 2 | 0 | 3.86 | 1.93 |
| Chr7:97000429-97000451 | 97000435 | 4 | RNA | GGCCGAGGTCGACTACCGGCNRG | GGC--AGGgCGACcACgcGtGGG | - | 5 | 2 | 0 | 3.86 | 1.93 |
| Chr7:82868060-82868082 | 82868075 | 5 | RNA | GGCCGAGGTCGACTACCGGCNRG | GGC--tcGcgGACTACtGGCTGG | + | 5 | 2 | 0 | 3.86 | 1.93 |
| Chr3:163802895-163802917 | 163802900 | 3 | DNA | GGCCGAGGTCGACTA--CCGGCNRG | ctCCaAGGcCGACcAATCCGGCAGG | - | 5 | 2 | 1 | 2.75 | 1.88 |
| Chr1:107894824-107894846 | 107894829 | 3 | RNA | GGCCGAGGTCGACTACCGGCNRG | GGaCGAG--aGACTgaCGGgCGG | - | 5 | 2 | 0 | 3.30 | 1.65 |
| Chr9:155135-155157 | 155142 | 5 | RNA | GGCCGAGGTCGACTACCGGCNRG | GGC--AGtTgGAtggCCGGCCGG | - | 5 | 2 | 0 | 3.30 | 1.65 |
| Chr10:101005287-101005309 | 101005305 | 2 | RNA | GGCCGAGGTCGACTACCGGCNRG | GGCCGAGGcgGAgg--CGcCCAG | + | 5 | 2 | 0 | 3.30 | 1.65 |
| Chr5:122217141-122217163 | 122217146 | 3 | RNA | GGCCGAGGTCGACTACCGGCNRG | GGC-aAGGTCaACgACCGGtGAG | - | 4 | 1 | 0 | 3.30 | 1.65 |
| Chr5:79950097-79950119 | 79950113 | 4 | DNA | GGC--CGAGGTCGACTACCGGCNRG | GGCCTCGgGGgCtACacCCGGCGGG | + | 5 | 2 | 0 | 3.30 | 1.65 |
| Chr2:134230735-134230757 | 134230751 | 4 | RNA | GGCCGAGGTCGACTACCGGCNRG | ctCCGcGGTCaA--ACCGGgGGG | + | 5 | 2 | 0 | 3.30 | 1.65 |
| Chr2:87737890-87737912 | 87737894 | 2 | RNA | GGCCGAGGTCGACTACCGGCNRG | GG--GAGGagGtaTACgGGCAAG | - | 5 | 2 | 0 | 3.30 | 1.65 |
| Chr10:53575327-53575349 | 53575333 | 4 | RNA | GGCCGAGGTCGACTACCGGCNRG | GGCCaAGGcCGgCcAt-GGCGAG | - | 5 | 1 | 0 | 3.30 | 1.65 |
| Chr3:81163555-81163577 | 81163560 | 3 | RNA | GGCCGAGGTCGACTACCGGCNRG | GcCaGtGGaCGgC--CCGGCCAG | - | 5 | 2 | 0 | 3.30 | 1.65 |
| Chr1:203667593-203667615 | 203667609 | 4 | RNA | GGCCGAGGTCGACTACCGGCNRG | GGagGcGGTgGtC-ACCGGCGAG | + | 5 | 1 | 0 | 3.30 | 1.65 |
| Chr2:211201974-211201996 | 211201979 | 3 | RNA | GGCCGAGGTCGACTACCGGCNRG | GtCCG--GaaGAaTACCGGaAAG | - | 5 | 2 | 0 | 3.30 | 1.65 |
| Chr2:51176098-51176120 | 51176116 | 2 | RNA | GGCCGAGGTCGACTACCGGCNRG | GGCCGA--TaGcCcgCCcGCCGG | + | 5 | 2 | 0 | 3.30 | 1.65 |
| Chr8:17674943-17674965 | 17674961 | 2 | RNA | GGCCGAGGTCGACTACCGGCNRG | GGgCGAGGgaGACgA--GGgAGG | + | 5 | 2 | 0 | 3.30 | 1.65 |
| Chr8:78435436-78435458 | 78435442 | 4 | DNA | GGC-CGAGGTCGACTACCGGCNRG | GcCTCGgGGaCGtCTtCCGGCTGG | - | 5 | 1 | 0 | 3.30 | 1.65 |
| Chr8:28603190-28603212 | 28603208 | 2 | RNA | GGCCGAGGTCGACTACCGGCNRG | GcaCGAGG--GACTAtgGaCAAG | + | 5 | 2 | 0 | 3.30 | 1.65 |
| Chr5:30767483-30767505 | 30767487 | 2 | RNA | GGCCGAGGTCGACTACCGGCNRG | GGC--AtcTCGAaTgCCGGtGAG | - | 5 | 2 | 0 | 3.30 | 1.65 |
| Chr2:166609198-166609220 | 166609204 | 4 | DNA | GGCCGAGGTCGACTACCG--GCNRG | GGCCGgGGTCGgCccaCGGCGCGGG | - | 5 | 2 | 0 | 3.30 | 1.65 |
| Chr9:30580435-30580457 | 30580451 | 4 | RNA | GGCCGAGGTCGACTACCGGCNRG | GaCaG-GGTCGACgACaaGCGGG | + | 5 | 1 | 0 | 3.30 | 1.65 |
| Chr5:190986159-190986181 | 190986165 | 4 | RNA | GGCCGAGGTCGACTACCGGCNRG | GaCCaAGGTCttCTACC--gCGG | - | 5 | 2 | 0 | 3.30 | 1.65 |
| Chr1:270926327-270926349 | 270926342 | 5 | RNA | GGCCGAGGTCGACTACCGGCNRG | GGC--tcGcgGACTACCGGCTGG | + | 4 | 2 | 0 | 3.30 | 1.65 |
| Chr2:12880441-12880463 | 12880448 | 5 | RNA | GGCCGAGGTCGACTACCGGCNRG | tGgCGAGGTCGAggACC--tTGG | - | 5 | 2 | 0 | 3.30 | 1.65 |
| Chr8:87377392-87377414 | 87377398 | 4 | RNA | GGCCGAGGTCGACTACCGGCNRG | GGCC-AcGTtGcCgACCGtCCAG | - | 5 | 1 | 0 | 3.30 | 1.65 |
| Chr8:21761405-21761427 | 21761421 | 4 | RNA | GGCCGAGGTCGACTACCGGCNRG | G-aCGgGGTCGACgACgaGCGGG | + | 5 | 1 | 0 | 3.30 | 1.65 |
| Chr6:121253118-121253140 | 121253135 | 3 | RNA | GGCCGAGGTCGACTACCGGCNRG | GGC--AtGTCtACTgCCcGgGAG | + | 5 | 2 | 0 | 3.30 | 1.65 |
| Chr10:118840559-118840581 | 118840575 | 4 | DNA | GGCCGAGGTCGACTA--CCGGCNRG | ctCCtAGGcCGACcAATCCGGCAGG | + | 5 | 2 | 0 | 3.30 | 1.65 |
| Chr2:26369481-26369503 | 26369486 | 3 | RNA | GGCCGAGGTCGACTACCGGCNRG | GGtCGgGGcC--CgACCGGtCAG | - | 5 | 2 | 0 | 3.30 | 1.65 |
| Chr9:47366433-47366455 | 47366449 | 4 | RNA | GGCCGAGGTCGACTACCGGCNRG | G-aCGgGGTCGACgACgaGCGGG | + | 5 | 1 | 0 | 3.30 | 1.65 |
| Chr2:46847383-46847405 | 46847388 | 3 | DNA | GGC--CGAGGTCGACTACCGGCNRG | GGCCTCGgGGgCtACacCCGGCGGG | - | 5 | 2 | 0 | 3.30 | 1.65 |
| Chr1:197247089-197247111 | 197247104 | 5 | RNA | GGCCGAGGTCGACTACCGGCNRG | GGgC-AGGcCGAgcACCGGCCGG | + | 4 | 1 | 0 | 3.30 | 1.65 |
| Chr4:201985716-201985738 | 201985732 | 4 | RNA | GGCCGAGGTCGACTACCGGCNRG | GG--GtcGTCGcgcACCGGCGGG | + | 5 | 2 | 0 | 3.30 | 1.65 |
| Chr6:167238488-167238510 | 167238495 | 5 | DNA | GGCCGAG--GTCGACTACCGGCNRG | GGCCcAGCAGcaGACTAgCGGCCAG | - | 4 | 2 | 0 | 3.30 | 1.65 |
| Chr1:191590798-191590820 | 191590815 | 3 | RNA | GGCCGAGGTCGACTACCGGCNRG | GGgCGAGtgCG--gACaGGCGGG | + | 5 | 2 | 0 | 3.30 | 1.65 |
| Chr2:149852123-149852145 | 149852138 | 5 | RNA | GGCCGAGGTCGACTACCGGCNRG | GGCtGAGGcgtcCTAC--GCAGG | + | 5 | 2 | 0 | 3.30 | 1.65 |
| Chr8:23452031-23452053 | 23452036 | 3 | DNA | GGC--CGAGGTCGACTACCGGCNRG | GGCCTCGgGGgCtACacCCGGCGGG | - | 5 | 2 | 0 | 3.30 | 1.65 |
| Chr8:104466062-104466084 | 104466066 | 2 | RNA | GGCCGAGGTCGACTACCGGCNRG | GaggGAGG--GAgcACCGGCCAG | - | 5 | 2 | 0 | 3.30 | 1.65 |
| Chr4:204778178-204778200 | 204778193 | 5 | RNA | GGCCGAGGTCGACTACCGGCNRG | GGgCG-GGcCaACctCCGGCCGG | + | 5 | 1 | 0 | 3.30 | 1.65 |
| Chr7:86848533-86848555 | 86848550 | 3 | RNA | GGCCGAGGTCGACTACCGGCNRG | GGCCGAGtaCG--TtgCcGCAGG | + | 5 | 2 | 0 | 3.30 | 1.65 |
| Chr8:86712842-86712864 | 86712849 | 5 | RNA | GGCCGAGGTCGACTACCGGCNRG | aaCCGAGGgCGcCT-CgGGCAGG | - | 5 | 1 | 0 | 3.30 | 1.65 |
| Chr4:45194669-45194691 | 45194684 | 5 | RNA | GGCCGAGGTCGACTACCGGCNRG | GcCaGtGGaCGAC--CtGGCCAG | + | 5 | 2 | 0 | 3.30 | 1.65 |
| Chr4:224844686-224844708 | 224844691 | 3 | RNA | GGCCGAGGTCGACTACCGGCNRG | G-aCGgGGTCGACaACgaGCGGG | - | 5 | 1 | 0 | 3.30 | 1.65 |

| | | | | | | | | | | |
|---|---|---|---|---|---|---|---|---|---|---|
| Chr2:122879933-122879955 | 122879937 | 2 | RNA | GGCCGAGGTCGACTACCGGCNRG | cGCC-AGGcCGAgctCCGGCGAG | - | 5 | 1 | 0 | 3.30 | 1.65 |
| Chr5:158999624-158999646 | 158999640 | 4 | RNA | GGCCGAGGTCGACTACCGGCNRG | GGgaGAGGggGAgT--CGGCCAG | + | 5 | 2 | 0 | 3.30 | 1.65 |
| Chr10:48356902-48356924 | 48356906 | 2 | DNA | GGCCGAGGTCGACTACCG--GCNRG | GGCgGAGGTCGtCTACaGAAGCCAG | - | 3 | 2 | 0 | 3.30 | 1.65 |
| Chr7:135420232-135420254 | 135420237 | 3 | DNA | GGC-CGAGGTCGACTACCGGCNRG | aGCTCGAGGgCGAgTgaCGGCGAG | - | 5 | 1 | 0 | 3.30 | 1.65 |
| Chr7:31525853-31525875 | 31525859 | 4 | DNA | GGC--CGAGGTCGACTACCGGCNRG | GGCGACGAGcTCGACTcCgGtgTGG | - | 5 | 2 | 0 | 3.30 | 1.65 |
| Chr2:24119474-24119496 | 24119492 | 2 | DNA | GG-CCGAGGTCGACTACCGGCNRG | GGTCCGAGGcCGAggtCgGGCGAG | + | 5 | 1 | 0 | 3.30 | 1.65 |
| Chr5:9102752-9102774 | 9102758 | 4 | RNA | GGCCGAGGTCGACTACCGGCNRG | GGt-aAGGgCGgCTgCCGGCTGG | - | 5 | 1 | 0 | 3.30 | 1.65 |
| Chr1:249986873-249986895 | 249986889 | 4 | RNA | GGCCGAGGTCGACTACCGGCNRG | GG--GAGtTCaAaaACCGGaGGG | + | 5 | 2 | 0 | 3.30 | 1.65 |
| Chr2:79513821-79513843 | 79513838 | 3 | RNA | GGCCGAGGTCGACTACCGGCNRG | GGCCGAGc-CaACgAtCGGgAGG | + | 5 | 1 | 0 | 3.30 | 1.65 |
| Chr9:158828559-158828581 | 158828563 | 2 | RNA | GGCCGAGGTCGACTACCGGCNRG | tG--GAGGaaGACgACCtGCAAG | - | 5 | 2 | 0 | 3.30 | 1.65 |
| Chr1:304064781-304064803 | 304064798 | 3 | RNA | GGCCGAGGTCGACTACCGGCNRG | cGgCGAGG--GAaTACgGGaAGG | + | 5 | 2 | 0 | 3.30 | 1.65 |
| Chr9:125811554-125811576 | 125811569 | 5 | RNA | GGCCGAGGTCGACTACCGGCNRG | GGaCGAcGTgGAt--CtGGCGAG | + | 5 | 2 | 0 | 3.30 | 1.65 |
| Chr5:107175111-107175133 | 107175128 | 3 | RNA | GGCCGAGGTCGACTACCGGCNRG | GtCCaAGcaCaACTACC--CGAG | + | 5 | 2 | 0 | 3.30 | 1.65 |
| Chr8:38140537-38140559 | 38140542 | 3 | RNA | GGCCGAGGTCGACTACCGGCNRG | GGCCaAGGgtGcCT-CgGGCTAG | - | 5 | 1 | 0 | 3.30 | 1.65 |
| Chr4:144024909-144024931 | 144024925 | 4 | RNA | GGCCGAGGTCGACTACCGGCNRG | GGCCcActTCcACTAgCG-CAAG | + | 5 | 1 | 0 | 3.30 | 1.65 |
| Chr7:93519602-93519624 | 93519607 | 3 | DNA | GGCCGAGGTCGA--CTACCGGCNRG | GGtCGAGGgCGAGTCgAtCGGCAGG | - | 4 | 2 | 0 | 3.30 | 1.65 |
| Chr5:192960636-192960658 | 192960654 | 2 | DNA | GGCCGA--GGTCGACTACCGGCNRG | GtCaGACTGGcaGACTACaGGCCAG | + | 5 | 2 | 0 | 3.30 | 1.65 |
| Chr7:16047436-16047458 | 16047443 | 5 | RNA | GGCCGAGGTCGACTACCGGCNRG | GGgCGAG--CGctgACaGGCGGG | - | 5 | 2 | 0 | 3.30 | 1.65 |
| Chr4:143196923-143196945 | 143196941 | 2 | DNA | GGCCGAGGTCGA--CTACCGGCNRG | GGtCGAcGTCGAGGCcAgtGGCGGG | + | 5 | 2 | 0 | 3.30 | 1.65 |
| Chr9:69062577-69062599 | 69062595 | 2 | RNA | GGCCGAGGTCGACTACCGGCNRG | GGC-tAGGcCGAggACCtGCAGG | + | 5 | 1 | 0 | 3.30 | 1.65 |
| Chr3:7770701-7770723 | 7770718 | 3 | DNA | GGC-CGAGGTCGACTACCGGCNRG | GGCTCcAcGcCcACTACtGGCCAG | + | 5 | 1 | 0 | 3.30 | 1.65 |
| Chr10:121024132-121024154 | 121024136 | 2 | RNA | GGCCGAGGTCGACTACCGGCNRG | GGCCGAGtcCGAgTcCC--tGGG | - | 5 | 2 | 0 | 3.30 | 1.65 |
| Chr1:159291722-159291744 | 159291727 | 3 | DNA | GGCC-GAGGTCGACTACCGGCNRG | GGCCGGAGGTgGAagACgaGCAGG | - | 5 | 1 | 0 | 3.30 | 1.65 |
| Chr4:54536944-54536966 | 54536949 | 3 | DNA | GGCCGAGGTCGACT-ACCGGCNRG | GGttGAGGaCGACTGACaGGtGGG | - | 5 | 1 | 0 | 3.30 | 1.65 |
| Chr2:105621831-105621853 | 105621836 | 3 | RNA | GGCCGAGGTCGACTACCGGCNRG | t-CCGAcGgCGtCTAgCGGCGAG | - | 5 | 1 | 0 | 3.30 | 1.65 |
| Chr7:37988950-37988972 | 37988966 | 4 | RNA | GGCCGAGGTCGACTACCGGCNRG | aGCCGAGGgtGcCT-CgGGCTGG | + | 5 | 1 | 0 | 3.30 | 1.65 |
| Chr10:1696886-1696908 | 1696901 | 5 | RNA | GGCCGAGGTCGACTACCGGCNRG | GGCgGcGG--GgCggCCGGCAGG | + | 5 | 2 | 0 | 3.30 | 1.65 |
| Chr8:132268614-132268636 | 132268620 | 4 | RNA | GGCCGAGGTCGACTACCGGCNRG | GGCtGgGGTaG--cACCGGgAGG | - | 5 | 2 | 0 | 3.30 | 1.65 |
| Chr1:155052334-155052356 | 155052349 | 5 | RNA | GGCCGAGGTCGACTACCGGCNRG | GGCaGgGG-CGACcACaaGCGGG | + | 5 | 1 | 0 | 3.30 | 1.65 |
| Chr4:10986142-10986164 | 10986149 | 5 | DNA | GGCCGAGGTCGACTACC--GGCNRG | GGCgGAGGTCGtCTgCaGAGGCCAG | - | 4 | 2 | 0 | 3.30 | 1.65 |
| Chr2:72771318-72771340 | 72771334 | 4 | RNA | GGCCGAGGTCGACTACCGGCNRG | GGC--tcGcaGACTACCGGCTGG | + | 4 | 2 | 0 | 3.30 | 1.65 |
| Chr10:129636421-129636443 | 129636437 | 4 | RNA | GGCCGAGGTCGACTACCGGCNRG | GcCCGAGGcCGA--ACtcGgTGG | + | 5 | 2 | 0 | 3.30 | 1.65 |
| Chr3:103618456-103618478 | 103618474 | 2 | DNA | GGCCGAGGTCG-ACTACCGGCNRG | GGaCGAGGgCGTACaACCacCAAG | + | 5 | 1 | 0 | 3.30 | 1.65 |
| Chr2:185635696-185635718 | 185635701 | 3 | DNA | GGCCGAGGTCGACT-ACCGGCNRG | cGCaGAGGTtGtCTGACCGGtCGG | - | 5 | 1 | 0 | 3.30 | 1.65 |
| Chr7:86575947-86575969 | 86575952 | 3 | DNA | GGC-CGAGGTCGACTACCGGCNRG | GcCTCGgGGaCGtCTtCCGGCTGG | - | 5 | 1 | 0 | 3.30 | 1.65 |
| Chr5:97641978-97642000 | 97641993 | 5 | RNA | GGCCGAGGTCGACTACCGGCNRG | GtCCG--GTgcACcACCGGaCAG | + | 5 | 2 | 0 | 3.30 | 1.65 |
| Chr9:128092340-128092362 | 128092355 | 5 | RNA | GGCCGAGGTCGACTACCGGCNRG | GGaCcAGGTCG--TcCCaGtTGG | + | 5 | 2 | 0 | 3.30 | 1.65 |
| Chr8:139942205-139942227 | 139942222 | 3 | DNA | GGCCGAGGTCGACTACC--GGCNRG | GGCgGAGGTCGtCggCaGAGGCCAG | + | 5 | 2 | 0 | 3.30 | 1.65 |
| Chr3:4825390-4825412 | 4825396 | 4 | RNA | GGCCGAGGTCGACTACCGGCNRG | GcCgGAGccCGAC--CCGtCAAG | - | 5 | 2 | 0 | 3.30 | 1.65 |
| Chr7:62390275-62390297 | 62390291 | 4 | RNA | GGCCGAGGTCGACTACCGGCNRG | GGgCGAGcTCGgCcAC-GGCAAG | + | 4 | 1 | 0 | 3.30 | 1.65 |
| Chr8:153505433-153505455 | 153505438 | 3 | RNA | GGCCGAGGTCGACTACCGGCNRG | GGtCGAGcTC--CTcCCcGCCGG | - | 4 | 2 | 0 | 3.30 | 1.65 |
| Chr8:119767152-119767174 | 119767169 | 3 | RNA | GGCCGAGGTCGACTACCGGCNRG | GGatGAGGTgGACgA--GGCGGG | + | 4 | 2 | 0 | 3.30 | 1.65 |
| Chr1:73387788-73387810 | 73387795 | 5 | RNA | GGCCGAGGTCGACTACCGGCNRG | G-aCGgGGTCGACaACgaGCGGG | - | 5 | 1 | 0 | 3.30 | 1.65 |
| Chr7:48100604-48100626 | 48100620 | 4 | RNA | GGCCGAGGTCGACTACCGGCNRG | G-aCGgGGTCGACaACgaGCGGG | + | 5 | 1 | 0 | 3.30 | 1.65 |
| Chr2:44995534-44995556 | 44995552 | 2 | RNA | GGCCGAGGTCGACTACCGGCNRG | GGCCGA--TaGcCcACCcGCCGG | + | 4 | 2 | 0 | 3.30 | 1.65 |
| Chr8:158801857-158801879 | 158801872 | 5 | RNA | GGCCGAGGTCGACTACCGGCNRG | GGC--AGGcCGAgcACCGGCCGG | + | 3 | 2 | 0 | 3.30 | 1.65 |
| Chr2:115610922-115610944 | 115610927 | 3 | RNA | GGCCGAGGTCGACTACCGGCNRG | aGC--AGGTgGACTtCgGGaGAG | - | 5 | 2 | 0 | 3.30 | 1.65 |
| Chr5:159188090-159188112 | 159188097 | 5 | RNA | GGCCGAGGTCGACTACCGGCNRG | GGCCGAGGcCGg-TgCaGGgAAG | - | 5 | 1 | 0 | 3.30 | 1.65 |
| Chr2:108882451-108882473 | 108882467 | 4 | DNA | GGCCGAGGTCGACT--ACCGGCNRG | tcCCGAGGcCGACTCCtCCGtCGAG | + | 5 | 2 | 0 | 3.30 | 1.65 |
| Chr4:120350896-120350918 | 120350914 | 2 | RNA | GGCCGAGGTCGACTACCGGCNRG | GGCCGtGtTC-ACcgCCGtCGGG | + | 5 | 1 | 0 | 3.30 | 1.65 |
| Chr3:39270810-39270832 | 39270815 | 3 | DNA | GGCCGAGGTCGACT-ACCGGCNRG | GGgtGAGacaGACTGACCGGCGGG | - | 5 | 1 | 0 | 3.30 | 1.65 |
| Chr6:29714832-29714854 | 29714850 | 2 | RNA | GGCCGAGGTCGACTACCGGCNRG | GGggGAGGgCG--TACaGaCGAG | + | 5 | 2 | 0 | 3.30 | 1.65 |
| Chr5:199787056-199787078 | 199787060 | 2 | RNA | GGCCGAGGTCGACTACCGGCNRG | GGCCGAGGcCGg-TgCgGGgAAG | - | 5 | 1 | 0 | 3.30 | 1.65 |
| Chr8:139103175-139103197 | 139103182 | 5 | RNA | GGCCGAGGTCGACTACCGGCNRG | GGCgGAaGTCG--TtCgGcCGAG | - | 5 | 2 | 0 | 3.30 | 1.65 |
| Chr3:154781338-154781360 | 154781356 | 2 | RNA | GGCCGAGGTCGACTACCGGCNRG | G--CGAGGaCGgCatCCcGCCGG | + | 5 | 2 | 0 | 3.30 | 1.65 |
| Chr1:18438664-18438686 | 18438670 | 4 | RNA | GGCCGAGGTCGACTACCGGCNRG | GGCgGAGGTCG--TtCgGcaGAG | - | 5 | 2 | 0 | 3.30 | 1.65 |
| Chr8:153051902-153051924 | 153051918 | 4 | RNA | GGCCGAGGTCGACTACCGGCNRG | GGCCGAGccCGAg--CCGagGGG | + | 5 | 2 | 0 | 3.30 | 1.65 |
| Chr2:9239214-9239236 | 9239218 | 2 | RNA | GGCCGAGGTCGACTACCGGCNRG | GGgCG-GGcCaACctCCGGCCGG | - | 5 | 1 | 0 | 3.30 | 1.65 |
| Chr7:135879138-135879160 | 135879144 | 4 | DNA | GGC-CGAGGTCGACTACCGGCNRG | GGCTCGgaGaCGtCTtCCGGCTGG | - | 5 | 1 | 0 | 3.30 | 1.65 |
| Chr9:15295368-15295390 | 15295374 | 4 | RNA | GGCCGAGGTCGACTACCGGCNRG | GGC--ttGctGACTACCGGCTGG | - | 4 | 2 | 0 | 3.30 | 1.65 |
| Chr5:168752859-168752881 | 168752865 | 4 | RNA | GGCCGAGGTCGACTACCGGCNRG | tGaCGAGGaCGAagAC-GGCAGG | - | 5 | 1 | 0 | 3.30 | 1.65 |
| Chr4:68993740-68993762 | 68993755 | 5 | RNA | GGCCGAGGTCGACTACCGGCNRG | aGCaGAGGcCGA--AgCGaCCAG | + | 5 | 2 | 0 | 3.30 | 1.65 |
| Chr2:1392809-1392831 | 1392825 | 4 | RNA | GGCCGAGGTCGACTACCGGCNRG | G-aCGAGGTCGACgACgaGCGGG | + | 4 | 1 | 0 | 3.30 | 1.65 |
| Chr6:169119644-169119666 | 169119649 | 3 | RNA | GGCCGAGGTCGACTACCGGCNRG | GcCgGAGccCGAC--CCGtCAGG | - | 5 | 2 | 0 | 3.30 | 1.65 |
| Chr2:90107750-90107772 | 90107756 | 4 | RNA | GGCCGAGGTCGACTACCGGCNRG | G-aCGgGGTCGACgACgaGCGGG | - | 5 | 1 | 0 | 3.30 | 1.65 |
| Chr6:84118713-84118735 | 84118717 | 2 | RNA | GGCCGAGGTCGACTACCGGCNRG | GGCCacGGTCGAC-AaCaaCCAG | - | 5 | 1 | 0 | 3.30 | 1.65 |
| Chr1:259601813-259601835 | 259601829 | 4 | DNA | GGCCGAGG--TCGACTACCGGCNRG | GcCgcAGcCATCGACTcCCGGCCAG | + | 5 | 2 | 0 | 3.30 | 1.65 |
| Chr10:149762722-149762744 | 149762737 | 5 | DNA | GGCCG--AGGTCGACTACCGGCNRG | GGCCGGTAtGgCGcCTcCCtGCGAG | + | 5 | 2 | 0 | 3.30 | 1.65 |
| Chr1:79911649-79911671 | 79911667 | 2 | RNA | GGCCGAGGTCGACTACCGGCNRG | GGCgGAGGcCaAaTt--GGCGAG | + | 5 | 2 | 0 | 3.30 | 1.65 |
| Chr4:142083947-142083969 | 142083963 | 4 | RNA | GGCCGAGGTCGACTACCGGCNRG | GGCgacGcaCGACTACC--CGAG | + | 5 | 2 | 0 | 3.30 | 1.65 |
| Chr2:112183309-112183331 | 112183315 | 4 | RNA | GGCCGAGGTCGACTACCGGCNRG | GGCCGAtGTgGAgTA--GagAAG | - | 5 | 2 | 0 | 3.30 | 1.65 |
| Chr6:148381872-148381894 | 148381887 | 5 | RNA | GGCCGAGGTCGACTACCGGCNRG | GtC--tccTtGACTACCGGCCGG | + | 5 | 2 | 0 | 3.30 | 1.65 |
| Chr6:79844790-79844812 | 79844806 | 4 | DNA | GGCCGAGGTCGA--CTACCGGCNRG | GGCCGAGGTCGAGTCTgagccCTGG | + | 5 | 2 | 0 | 3.30 | 1.65 |
| Chr3:76184669-76184691 | 76184687 | 2 | RNA | GGCCGAGGTCGACTACCGGCNRG | GGCgGtGGct--CTAaCGGCGAG | + | 5 | 2 | 0 | 3.30 | 1.65 |
| Chr1:69014233-69014255 | 69014249 | 4 | RNA | GGCCGAGGTCGACTACCGGCNRG | acCCGAGGTtGgC--CCcGCGAG | + | 5 | 2 | 0 | 3.30 | 1.65 |
| Chr6:148966046-148966068 | 148966062 | 4 | RNA | GGCCGAGGTCGACTACCGGCNRG | GGCCccG--aGACTtCCaGCTAG | + | 5 | 2 | 0 | 3.30 | 1.65 |
| Chr4:34640935-34640957 | 34640950 | 5 | X | GGCCGAGGTCGACTACCGGCNRG | GGCgGAGGaCGcCTACCtGCTAG | + | 4 | 0 | 0 | 3.30 | 1.65 |
| Chr1:14600836-14600858 | 14600843 | 5 | DNA | GGCCGAG-GTCGACTACCGGCNRG | GttCGAGCGTCaACgAtCGGCAGG | - | 5 | 1 | 0 | 3.30 | 1.65 |
| Chr5:150381051-150381073 | 150381057 | 4 | RNA | GGCCGAGGTCGACTACCGGCNRG | GGCCG--GTCGgCaACtGcaCGG | - | 5 | 2 | 0 | 3.30 | 1.65 |
| Chr8:59459813-59459835 | 59459817 | 2 | RNA | GGCCGAGGTCGACTACCGGCNRG | cGCCG--GTCatCTtCCtGCGGG | - | 5 | 2 | 0 | 3.30 | 1.65 |
| Chr5:81399374-81399396 | 81399391 | 3 | RNA | GGCCGAGGTCGACTACCGGCNRG | GcCCGAGccCGAgT--CGGtAGG | + | 5 | 2 | 0 | 3.30 | 1.65 |
| Chr7:56199891-56199913 | 56199895 | 2 | RNA | GGCCGAGGTCGACTACCGGCNRG | GGtCGAGGTgG--gtgCGGCGAG | - | 5 | 2 | 0 | 3.30 | 1.65 |
| Chr8:27680198-27680220 | 27680215 | 3 | RNA | GGCCGAGGTCGACTACCGGCNRG | GGC--AGGcCagCctCCGGCCGG | + | 5 | 2 | 0 | 3.30 | 1.65 |
| Chr7:60205438-60205460 | 60205442 | 2 | RNA | GGCCGAGGTCGACTACCGGCNRG | aGCCtcGGgCGACTACt--CTGG | - | 5 | 2 | 0 | 3.30 | 1.65 |
| Chr2:46487787-46487809 | 46487804 | 3 | RNA | GGCCGAGGTCGACTACCGGCNRG | GGaCcAGGTCG--TcCCaGtTGG | + | 5 | 2 | 0 | 3.30 | 1.65 |
| Chr4:122650852-122650874 | 122650870 | 2 | RNA | GGCCGAGGTCGACTACCGGCNRG | GGCCGAGGcCtcCT-CgGGCTGG | + | 4 | 1 | 0 | 3.30 | 1.65 |
| Chr9:121338305-121338327 | 121338320 | 5 | RNA | GGCCGAGGTCGACTACCGGCNRG | GGCtcAGG--GgCTACtGtCGGG | + | 5 | 2 | 0 | 3.30 | 1.65 |
| Chr6:81106471-81106493 | 81106489 | 2 | RNA | GGCCGAGGTCGACTACCGGCNRG | GcCgGAGccCGAC--CCGtCAGG | + | 5 | 2 | 0 | 3.30 | 1.65 |
| Chr5:34015526-34015548 | 34015544 | 2 | RNA | GGCCGAGGTCGACTACCGGCNRG | tGCCacGGT--ACTACttGCTAG | + | 5 | 2 | 0 | 3.30 | 1.65 |
| Chr8:113133356-113133378 | 113133360 | 2 | RNA | GGCCGAGGTCGACTACCGGCNRG | cGCCGgGGTCGACTt--GGaGGG | - | 4 | 2 | 0 | 3.30 | 1.65 |
| Chr7:47143964-47143986 | 47143970 | 4 | RNA | GGCCGAGGTCGACTACCGGCNRG | GGCtGAGGcCGAgg--CGGgCAG | - | 5 | 2 | 0 | 3.30 | 1.65 |
| Chr10:130917066-130917088 | 130917072 | 4 | RNA | GGCCGAGGTCGACTACCGGCNRG | cGgCGAGGaCcACTAC--GgAGG | - | 5 | 2 | 0 | 3.30 | 1.65 |
| Chr5:64893484-64893506 | 64893489 | 3 | RNA | GGCCGAGGTCGACTACCGGCNRG | GGgaGgGGgCGA--ACCGGCCGG | - | 4 | 2 | 0 | 3.30 | 1.65 |
| Chr5:223339829-223339851 | 223339846 | 3 | RNA | GGCCGAGGTCGACTACCGGCNRG | GGC--tcGcgGACTACCGtCTGG | + | 5 | 2 | 0 | 3.30 | 1.65 |
| Chr6:153101756-153101778 | 153101761 | 3 | DNA | GGCCGAGGTCGACTACCG-GCNRG | GGCCGgGGcCGACatCgGTGCTGG | - | 5 | 1 | 0 | 3.30 | 1.65 |
| Chr1:286042778-286042800 | 286042794 | 4 | RNA | GGCCGAGGTCGACTACCGGCNRG | G-CCGcGcTCGACTgtCGGtTGG | + | 5 | 1 | 0 | 3.30 | 1.65 |
| Chr3:116178561-116178583 | 116178568 | 5 | RNA | GGCCGAGGTCGACTACCGGCNRG | GGCCGAGGgt-ACctCgGGCTAG | - | 5 | 1 | 0 | 3.30 | 1.65 |
| Chr4:213167543-213167565 | 213167558 | 5 | RNA | GGCCGAGGTCGACTACCGGCNRG | GGCCaAGG--GAgTACaacCAAG | + | 5 | 2 | 0 | 3.30 | 1.65 |
| Chr2:142259580-142259602 | 142259598 | 2 | RNA | GGCCGAGGTCGACTACCGGCNRG | GGCCtAGGTgGgC--CgaGCTGG | + | 5 | 2 | 0 | 3.30 | 1.65 |
| Chr7:28220809-28220831 | 28220824 | 5 | RNA | GGCCGAGGTCGACTACCGGCNRG | GGCaGAGG--GAgaACCGagAGG | + | 5 | 2 | 0 | 3.30 | 1.65 |
| Chr8:110954114-110954136 | 110954119 | 3 | DNA | GGCCGAGGTCGACT--ACCGGCNRG | GGgtGgGGTCaAtTTGACCGGCGGG | - | 5 | 2 | 1 | 2.20 | 1.60 |
| Chr8:178808992-178809014 | 178809008 | 4 | RNA | GGCCGAGGTCGACTACCGGCNRG | GGCCG-GGTCatCcACtGGCGGG | + | 4 | 1 | 1 | 2.20 | 1.60 |
| Chr2:66024995-66025017 | 66025010 | 5 | RNA | GGCCGAGGTCGACTACCGGCNRG | GGC--AGGcCGAgcACtaGCCGG | + | 5 | 2 | 1 | 2.20 | 1.60 |
| Chr1:217527608-217527630 | 217527626 | 2 | RNA | GGCCGAGGTCGACTACCGGCNRG | GGCCGAGcgCGA--AggGGgGAG | + | 5 | 2 | 1 | 2.20 | 1.60 |
| Chr5:57806840-57806862 | 57806845 | 3 | RNA | GGCCGAGGTCGACTACCGGCNRG | GGC--tcGcgGACTACCGGCTGG | - | 4 | 2 | 1 | 2.20 | 1.60 |
| Chr3:112228848-112228870 | 112228864 | 4 | RNA | GGCCGAGGTCGACTACCGGCNRG | GaggGAGtTCGAC--CgGGCAAG | + | 5 | 2 | 1 | 2.20 | 1.60 |
| Chr5:11517314-11517336 | 11517329 | 5 | RNA | GGCCGAGGTCGACTACCGGCNRG | GGCtcgGG--GgCTACtGGCGGG | + | 5 | 2 | 2 | 1.10 | 1.55 |
| Chr2:126822592-126822614 | 126822597 | 3 | RNA | GGCCGAGGTCGACTACCGGCNRG | G-aCGgGGTCGACgACgaGCGGG | - | 5 | 1 | 2 | 1.10 | 1.55 |
| Chr5:6293703-6293725 | 6293720 | 3 | DNA | GGC-CGAGGTCGACTACCGGCNRG | GGCTCGgGGaCGtCTtCCGGCTGG | + | 4 | 1 | 2 | 1.10 | 1.55 |
| Chr2:222205784-222205806 | 222205802 | 2 | DNA | GGC-CGAGGTCGACTACCGGCNRG | GcCTCGgGGaCGtCTtCCGGCTGG | + | 5 | 1 | 2 | 1.10 | 1.55 |
| Chr4:79497029-79497051 | 79497036 | 5 | DNA | GGCCGAGGT-CGACTACCGGCNRG | GGCCGAGGTGCctCTtCCaGtCAG | - | 5 | 1 | 3 | 0.00 | 1.50 |
| Chr8:100501490-100501512 | 100501497 | 5 | RNA | GGCCGAGGTCGACTACCGGCNRG | GtCCGcGtTCGACTcaC--CGAG | - | 5 | 2 | 3 | 0.00 | 1.50 |
| Chr9:4458431-4458453 | 4458448 | 3 | RNA | GGCCGAGGTCGACTACCGGCNRG | GGCgatGGTCaA--AgCGGCAGG | + | 5 | 2 | 3 | 0.00 | 1.50 |
| Chr7:42527345-42527367 | 42527352 | 5 | DNA | GGCCGAGGTCGACTACCGG--CNRG | GGCCGAGGTtGAggACgaGCCCTGG | - | 5 | 2 | 3 | 0.00 | 1.50 |
| Chr5:29045810-29045832 | 29045817 | 5 | DNA | GGCCGAGGTCGA--CTACCGGCNRG | cGCCGAGGggGAGGCcACCGGCGGG | - | 4 | 2 | 3 | 0.00 | 1.50 |
| Chr1:123873628-123873650 | 123873635 | 5 | RNA | GGCCGAGGTCGACTACCGGCNRG | GGC--tcGcgGACTACCGaCTGG | - | 5 | 2 | 3 | 0.00 | 1.50 |
| Chr10:70952836-70952858 | 70952853 | 3 | RNA | GGCCGAGGTCGACTACCGGCNRG | cGCCGAcGT-GAtcACCGaCAAG | + | 5 | 1 | 3 | 0.00 | 1.50 |
| Chr7:104956963-104956985 | 104956970 | 5 | RNA | GGCCGAGGTCGACTACCGGCNRG | GGCCccGGTgGcCT--CGaCAGG | - | 5 | 2 | 3 | 0.00 | 1.50 |
| Chr10:102562470-102562492 | 102562487 | 3 | RNA | GGCCGAGGTCGACTACCGGCNRG | GGC--AGacCGAgctCCGGCCGG | + | 5 | 2 | 3 | 0.00 | 1.50 |
| Chr1:257485659-257485681 | 257485666 | 5 | RNA | GGCCGAGGTCGACTACCGGCNRG | GGaCcAGGTCG--TcCCaGtTGG | - | 5 | 2 | 3 | 0.00 | 1.50 |
| Chr5:208851535-208851557 | 208851542 | 5 | RNA | GGCCGAGGTCGACTACCGGCNRG | G-aCGgGGTCGACaACgaGCGGG | - | 5 | 1 | 3 | 0.00 | 1.50 |
| Chr7:126396588-126396610 | 126396605 | 3 | RNA | GGCCGAGGTCGACTACCGGCNRG | G--tGcGGgCGAgTtCCGGCGGG | + | 5 | 2 | 3 | 0.00 | 1.50 |
| Chr4:116539376-116539398 | 116539383 | 5 | RNA | GGCCGAGGTCGACTACCGGCNRG | caCCaAGtTCGcCT-CCGGCCAG | - | 5 | 1 | 3 | 0.00 | 1.50 |
| Chr4:118075945-118075967 | 118075952 | 5 | RNA | GGCCGAGGTCGACTACCGGCNRG | GGaCGc--TCGgCTACttGCAGG | - | 5 | 2 | 3 | 0.00 | 1.50 |
| Chr2:114660869-114660891 | 114660886 | 3 | RNA | GGCCGAGGTCGACTACCGGCNRG | GGtCGtGGTCG-CgAgtGGCTGG | + | 5 | 1 | 3 | 0.00 | 1.50 |
| Chr5:29752652-29752674 | 29752659 | 5 | RNA | GGCCGAGGTCGACTACCGGCNRG | GGC--AGGcCGAgcACCGGCCGG | - | 3 | 2 | 3 | 0.00 | 1.50 |

| | | | | | | | | | | | |
|---|---|---|---|---|---|---|---|---|---|---|---|
| Chr3:195863035-195863057 | 195863042 | 5 | DNA | GGCCGAGGTCGACTA--CCGGCNRG | ctCCaAGGcCGACcAATCCGGCAGG | - | 5 | 2 | 3 | 0.00 | 1.50 |
| Chr9:9851479-9851501 | 9851496 | 3 | RNA | GGCCGAGGTCGACTACCGGCNRG | GGtCaAGGgCGAg--CtGGCCGG | + | 5 | 2 | 3 | 0.00 | 1.50 |
| Chr9:22545982-22546004 | 22545999 | 3 | RNA | GGCCGAGGTCGACTACCGGCNRG | GGtCGcaGTCGgC--CCGtCGAG | + | 5 | 2 | 3 | 0.00 | 1.50 |
| Chr8:53892169-53892191 | 53892186 | 3 | RNA | GGCCGAGGTCGACTACCGGCNRG | G-aCGgGGTCGACaACgaGCGGG | + | 5 | 1 | 3 | 0.00 | 1.50 |
| Chr4:45405850-45405872 | 45405857 | 5 | RNA | GGCCGAGGTCGACTACCGGCNRG | GGaCcAGGTCG--TcCCaGaTGG | - | 5 | 2 | 3 | 0.00 | 1.50 |
| Chr3:66790130-66790152 | 66790137 | 5 | RNA | GGCCGAGGTCGACTACCGGCNRG | cGtCGAGGgCGACT--CcGgAAG | - | 5 | 2 | 3 | 0.00 | 1.50 |
| Chr2:58887090-58887112 | 58887107 | 3 | RNA | GGCCGAGGTCGACTACCGGCNRG | GGgCG-GGcCaACctCCGGCCGG | + | 5 | 1 | 3 | 0.00 | 1.50 |
| Chr3:193999605-193999627 | 193999622 | 3 | RNA | GGCCGAGGTCGACTACCGGCNRG | GGCCGAGGcCGAC--CgGGaCAG | + | 3 | 2 | 3 | 0.00 | 1.50 |
| Chr4:136515459-136515481 | 136515466 | 5 | RNA | GGCCGAGGTCGACTACCGGCNRG | aGCCGAG--aGAgTcCCtGCGGG | - | 5 | 2 | 3 | 0.00 | 1.50 |
| Chr3:230050058-230050080 | 230050065 | 5 | RNA | GGCCGAGGTCGACTACCGGCNRG | GtCCG--GTgcACcACCGGaTAG | - | 5 | 2 | 3 | 0.00 | 1.50 |
| Chr6:55177304-55177326 | 55177321 | 3 | RNA | GGCCGAGGTCGACTACCGGCNRG | GcgCGAGGTCtgtTAC--GCCAG | + | 5 | 2 | 3 | 0.00 | 1.50 |
| Chr10:100294613-100294635 | 100294630 | 3 | RNA | GGCCGAGGTCGACTACCGGCNRG | t--CGAGGgCGACgtCCtGCAGG | + | 5 | 2 | 3 | 0.00 | 1.50 |
| Chr10:32442091-32442113 | 32442098 | 5 | RNA | GGCCGAGGTCGACTACCGGCNRG | GGCtGgGcTgGACTA--GGtTGG | - | 5 | 2 | 3 | 0.00 | 1.50 |
| Chr8:64079907-64079929 | 64079924 | 3 | RNA | GGCCGAGGTCGACTACCGGCNRG | GGaCaAcGaCGACTAC--GaTAG | + | 5 | 2 | 3 | 0.00 | 1.50 |
| Chr9:7723232-7723254 | 7723239 | 5 | DNA | GGCCGAGGTCGA--CTACCGGCNRG | GGCCGAGGcCGAGGCTgagGcCGAG | - | 5 | 2 | 3 | 0.00 | 1.50 |
| Chr2:155169783-155169805 | 155169800 | 3 | RNA | GGCCGAGGTCGACTACCGGCNRG | GGtCG--GTCtACTcCtGGgAAG | + | 5 | 2 | 3 | 0.00 | 1.50 |
| Chr4:33876484-33876506 | 33876501 | 3 | RNA | GGCCGAGGTCGACTACCGGCNRG | tGgCt-cGTgGACTACCGGCTGG | + | 5 | 1 | 3 | 0.00 | 1.50 |
| Chr6:73589961-73589983 | 73589968 | 5 | DNA | GGCCGAGGTCGACTACCGG--CNRG | GGCtGAGGgCGAtTgCCtGCTCCAG | - | 5 | 2 | 3 | 0.00 | 1.50 |
| Chr8:40668907-40668929 | 40668914 | 5 | RNA | GGCCGAGGTCGACTACCGGCNRG | GtCCGAGGT-GAgaAaCaGCCGG | - | 5 | 1 | 3 | 0.00 | 1.50 |
| Chr2:128137627-128137649 | 128137644 | 3 | DNA | GGCCGAGGTCGACTACCGG-CNRG | cGCCGAcGTCtACTcCaGGCCCGG | + | 5 | 1 | 3 | 0.00 | 1.50 |
| Chr4:228599171-228599193 | 228599178 | 5 | RNA | GGCCGAGGTCGACTACCGGCNRG | aaCCaAGtTCGcCT-CCGGCCAG | - | 5 | 1 | 3 | 0.00 | 1.50 |
| Chr2:213719094-213719116 | 213719101 | 5 | RNA | GGCCGAGGTCGACTACCGGCNRG | GtCCG--GcCGAtcACCcGCAGG | - | 5 | 2 | 3 | 0.00 | 1.50 |
| Chr2:124931731-124931753 | 124931748 | 3 | RNA | GGCCGAGGTCGACTACCGGCNRG | tGCCGtGGTCGcCg--CGGCAGG | + | 4 | 2 | 3 | 0.00 | 1.50 |
| Chr5:84770055-84770077 | 84770062 | 5 | RNA | GGCCGAGGTCGACTACCGGCNRG | GGC--AGGcCGAgcACCGGCCGG | - | 3 | 2 | 3 | 0.00 | 1.50 |
| Chr4:63176355-63176377 | 63176372 | 3 | DNA | GGCCGAGGTCGA--CTACCGGCNRG | GcgCcAtcTCGATTCTACCGGCGAG | + | 5 | 2 | 3 | 0.00 | 1.50 |
| Chr6:40000900-40000922 | 40000907 | 5 | RNA | GGCCGAGGTCGACTACCGGCNRG | GGaCGAtGTCGACgACt--gTAG | - | 5 | 2 | 3 | 0.00 | 1.50 |
| Chr4:111008653-111008675 | 111008660 | 5 | X | GGCCGAGGTCGACTACCGGCNRG | GGaaGAGGTgGcCTAgCGGCTGG | - | 5 | 0 | 3 | 0.00 | 1.50 |
| Chr4:188538030-188538052 | 188538037 | 5 | RNA | GGCCGAGGTCGACTACCGGCNRG | G-aCGgGGTCGACgACgaGCGGG | - | 5 | 1 | 3 | 0.00 | 1.50 |
| Chr3:77466751-77466773 | 77466768 | 3 | RNA | GGCCGAGGTCGACTACCGGCNRG | tG--GAtGTCGcCTAgCGGgGGG | + | 5 | 2 | 3 | 0.00 | 1.50 |
| Chr5:157330107-157330129 | 157330114 | 5 | DNA | GGC-CGAGGTCGACTACCGGCNRG | GcCTCGgGGaCGtCTtCCGGCTGG | - | 5 | 1 | 3 | 0.00 | 1.50 |
| Chr5:11328005-11328027 | 11328012 | 5 | DNA | GGCCGAG-GTCGACTACCGGCNRG | GttCGAGCGTCaACgAtCGGCGGG | - | 5 | 1 | 3 | 0.00 | 1.50 |
| Chr10:39633639-39633661 | 39633656 | 3 | DNA | GGCCGA-GGTCGACTACCGGCNRG | GGCCaACGGTCGACTACCacCGGG | + | 3 | 1 | 3 | 0.00 | 1.50 |
| Chr2:219570108-219570130 | 219570125 | 3 | DNA | GGCCGAGGTCGACTACC--GGCNRG | GGCgGAGGTCGtCggCtGAGGCCAG | + | 5 | 2 | 3 | 0.00 | 1.50 |
| Chr8:77297147-77297169 | 77297164 | 3 | RNA | GGCCGAGGTCGACTACCGGCNRG | GGCgGAtGTgGACTA--GaCTAG | + | 4 | 2 | 3 | 0.00 | 1.50 |
| Chr1:74967262-74967284 | 74967279 | 3 | RNA | GGCCGAGGTCGACTACCGGCNRG | GaCaG-GGTCGACgACaaGCGGG | + | 5 | 1 | 3 | 0.00 | 1.50 |
| Chr1:78346621-78346643 | 78346628 | 5 | RNA | GGCCGAGGTCGACTACCGGCNRG | GtCCG--GTgcACcACCGGaCAG | - | 5 | 2 | 3 | 0.00 | 1.50 |
| Chr4:91865786-91865808 | 91865793 | 5 | RNA | GGCCGAGGTCGACTACCGGCNRG | GGCacAGaTCaACTtC-GGCGAG | - | 5 | 1 | 3 | 0.00 | 1.50 |
| Chr6:80911482-80911504 | 80911499 | 3 | RNA | GGCCGAGGTCGACTACCGGCNRG | GGgCGAGG--GtaTggCGGCTGG | + | 5 | 2 | 3 | 0.00 | 1.50 |
| Chr8:85467880-85467902 | 85467887 | 5 | DNA | GGCCGAGGTCGACT-ACCGGCNRG | cGCCGcGGatGACTCACCGGaTAG | - | 5 | 1 | 3 | 0.00 | 1.50 |
| Chr5:11142263-11142285 | 11142280 | 3 | DNA | GGCCGAG-GTCGACTACCGGCNRG | GttCGAGCGTCaACgAtCGGCAGG | + | 5 | 1 | 3 | 0.00 | 1.50 |
| Chr4:42889199-42889221 | 42889206 | 5 | RNA | GGCCGAGGTCGACTACCGGCNRG | GGC--tcGcgGACTACCGGCTGG | - | 4 | 2 | 3 | 0.00 | 1.50 |
| Chr1:85673274-85673296 | 85673281 | 5 | RNA | GGCCGAGGTCGACTACCGGCNRG | GGC--tcGcgGACTACCGtCTGG | - | 5 | 2 | 3 | 0.00 | 1.50 |
| Chr2:206150669-206150691 | 206150676 | 5 | RNA | GGCCGAGGTCGACTACCGGCNRG | caCCaAGtTCGcCT-CCGGCCAG | - | 5 | 1 | 3 | 0.00 | 1.50 |
| Chr1:286083702-286083724 | 286083719 | 3 | RNA | GGCCGAGGTCGACTACCGGCNRG | GGCCGAG--CGAgTgCttGaTGG | + | 5 | 2 | 3 | 0.00 | 1.50 |
| Chr1:81781405-81781427 | 81781422 | 3 | RNA | GGCCGAGGTCGACTACCGGCNRG | GGgCGAG--CGctgACaGGCGGG | + | 5 | 2 | 3 | 0.00 | 1.50 |
| Chr8:69059183-69059205 | 69059190 | 5 | RNA | GGCCGAGGTCGACTACCGGCNRG | GGCCGAGGgt-ACctCaGGCTAG | - | 5 | 1 | 3 | 0.00 | 1.50 |
| Chr7:43056476-43056498 | 43056493 | 3 | RNA | GGCCGAGGTCGACTACCGGCNRG | GGCCgGaTC--CTcCCaGCGAG | + | 5 | 2 | 3 | 0.00 | 1.50 |
| Chr7:85795292-85795314 | 85795299 | 5 | RNA | GGCCGAGGTCGACTACCGGCNRG | GGCCatGGcCGAgT--CcGCTAG | - | 5 | 2 | 3 | 0.00 | 1.50 |
| Chr9:107862653-107862675 | 107862670 | 3 | RNA | GGCCGAGGTCGACTACCGGCNRG | GGC--tcGcgGACTACCGGCTGG | + | 4 | 2 | 3 | 0.00 | 1.50 |
| Chr2:188710859-188710881 | 188710876 | 3 | DNA | GGCCGAGG-TCGACTACCGGCNRG | GGCCGAGaAgCGcCTcCCGGCGGG | + | 4 | 1 | 3 | 0.00 | 1.50 |
| Chr10:53404957-53404979 | 53404964 | 5 | RNA | GGCCGAGGTCGACTACCGGCNRG | GGgaGAGGct--CTACtGGCCGG | - | 5 | 2 | 3 | 0.00 | 1.50 |
| Chr3:202112108-202112130 | 202112125 | 3 | RNA | GGCCGAGGTCGACTACCGGCNRG | GGtCGAtGcCGACaAC--GaGAG | + | 5 | 2 | 3 | 0.00 | 1.50 |
| Chr1:223701304-223701326 | 223701321 | 3 | RNA | GGCCGAGGTCGACTACCGGCNRG | GGtgGAGaTCaAC-ACCGGgAGG | + | 5 | 1 | 3 | 0.00 | 1.50 |
| Chr1:244285271-244285293 | 244285288 | 3 | DNA | GGCCGAGGTCGACTACCG-GCNRG | GGCtGAGGaCGACaAggGAGCGGG | + | 5 | 1 | 3 | 0.00 | 1.50 |
| Chr9:47069710-47069732 | 47069727 | 3 | RNA | GGCCGAGGTCGACTACCGGCNRG | GGCgcAGcTCaACT-CCGGCGAG | + | 4 | 1 | 3 | 0.00 | 1.50 |
| Chr7:145989044-145989066 | 145989061 | 3 | DNA | GGCCGAGGTCGACTACCGG--CNRG | GGCCGAGtcCGAgccCCGGGTCGGG | + | 5 | 2 | 3 | 0.00 | 1.50 |
| Chr10:96085458-96085480 | 96085465 | 5 | RNA | GGCCGAGGTCGACTACCGGCNRG | aGCaGAGGcCGA--AgCGaCCAG | - | 5 | 2 | 3 | 0.00 | 1.50 |
| Chr3:71530987-71531009 | 71531004 | 3 | RNA | GGCCGAGGTCGACTACCGGCNRG | GGCCGgGGcgGgC--CgGGCCGG | + | 5 | 2 | 3 | 0.00 | 1.50 |
| Chr1:134632198-134632220 | 134632215 | 3 | RNA | GGCCGAGGTCGACTACCGGCNRG | cGCgGgGGTC-ACcAgCGGCGAG | + | 5 | 1 | 3 | 0.00 | 1.50 |
| Chr9:1353433-1353455 | 1353450 | 3 | RNA | GGCCGAGGTCGACTACCGGCNRG | GG--GccGTCGACgACCGagAAG | + | 5 | 2 | 3 | 0.00 | 1.50 |
| Chr7:8233790-8233812 | 8233797 | 5 | RNA | GGCCGAGGTCGACTACCGGCNRG | GGCCGAGcgCGA--AggGGgGAG | - | 5 | 2 | 3 | 0.00 | 1.50 |
| Chr4:243915434-243915456 | 243915441 | 5 | RNA | GGCCGAGGTCGACTACCGGCNRG | G-aCGgGGTCGACaACgaGCGGG | - | 5 | 1 | 3 | 0.00 | 1.50 |
| Chr6:36665946-36665968 | 36665953 | 5 | RNA | GGCCGAGGTCGACTACCGGCNRG | GGagGcGG-CGAgcACCGGCGAG | - | 5 | 1 | 3 | 0.00 | 1.50 |
| Chr2:106984011-106984033 | 106984018 | 5 | RNA | GGCCGAGGTCGACTACCGGCNRG | GG--GAGGTCGACaAggGagGAG | - | 5 | 2 | 3 | 0.00 | 1.50 |
| Chr8:63793283-63793305 | 63793290 | 5 | RNA | GGCCGAGGTCGACTACCGGCNRG | GGC--tcGcaGACTACCGGCTGG | - | 4 | 2 | 3 | 0.00 | 1.50 |
| Chr8:98099744-98099766 | 98099761 | 3 | RNA | GGCCGAGGTCGACTACCGGCNRG | GGCgGAGGTCtAtTA--GGgAGG | + | 4 | 2 | 3 | 0.00 | 1.50 |
| Chr2:49880304-49880326 | 49880311 | 5 | DNA | GGCCGAG--GTCGACTACCGGCNRG | cGCCGAaTCGTCGtCcACCtGCCAG | - | 5 | 2 | 3 | 0.00 | 1.50 |
| Chr8:94951551-94951573 | 94951568 | 3 | DNA | GGCCGAGGTCGAC-TACCGGCNRG | aGCtGAGGaCGACGTAgCGGgCGG | + | 5 | 1 | 3 | 0.00 | 1.50 |
| Chr4:282017-282039 | 282034 | 3 | DNA | GGCCGAGGTCGA--CTACCGGCNRG | GcgCcAtaTCGATTCTACCGGCGAG | + | 5 | 2 | 3 | 0.00 | 1.50 |
| Chr4:146362552-146362574 | 146362569 | 3 | RNA | GGCCGAGGTCGACTACCGGCNRG | G-aCGgGGTCGACgACgaGCGGG | + | 5 | 1 | 3 | 0.00 | 1.50 |
| Chr6:150702946-150702968 | 150702953 | 5 | RNA | GGCCGAGGTCGACTACCGGCNRG | GGaCGAcccCGACT-CCGGtTGG | - | 5 | 1 | 3 | 0.00 | 1.50 |
| Chr6:6554771-6554793 | 6554788 | 3 | RNA | GGCCGAGGTCGACTACCGGCNRG | GcCCGAGGcCGA--ACtcGgTGG | + | 5 | 2 | 3 | 0.00 | 1.50 |
| Chr8:139110353-139110375 | 139110360 | 5 | RNA | GGCCGAGGTCGACTACCGGCNRG | cGCCGAaGTCGAtcA--GtCGAG | - | 5 | 2 | 3 | 0.00 | 1.50 |
| Chr4:246679674-246679696 | 246679681 | 5 | RNA | GGCCGAGGTCGACTACCGGCNRG | GGCCt--GcCaACTtCCGGgCAG | - | 5 | 2 | 3 | 0.00 | 1.50 |
| Chr4:148403328-148403350 | 148403335 | 5 | RNA | GGCCGAGGTCGACTACCGGCNRG | GGaCtcG--CGACTtCCGcCGGG | - | 5 | 2 | 3 | 0.00 | 1.50 |
| Chr8:44495456-44495478 | 44495473 | 3 | RNA | GGCCGAGGTCGACTACCGGCNRG | GGCCGAcGgCa--TACCtGgAGG | + | 5 | 2 | 3 | 0.00 | 1.50 |
| Chr7:137697183-137697205 | 137697190 | 5 | RNA | GGCCGAGGTCGACTACCGGCNRG | GGaCcAGGTCG--TcCCaGtTGG | - | 5 | 2 | 3 | 0.00 | 1.50 |
| Chr6:100859359-100859381 | 100859366 | 5 | RNA | GGCCGAGGTCGACTACCGGCNRG | GGC--AGGcCaACccCCGGCCGG | - | 4 | 2 | 3 | 0.00 | 1.50 |
| Chr2:134158352-134158374 | 134158359 | 5 | RNA | GGCCGAGGTCGACTACCGGCNRG | GGC--tcGcaGACTACCGGCTGG | - | 4 | 2 | 3 | 0.00 | 1.50 |
| Chr6:172138342-172138364 | 172138359 | 3 | RNA | GGCCGAGGTCGACTACCGGCNRG | t--CGgGGaCGtCTtCCGGCTGG | + | 5 | 2 | 3 | 0.00 | 1.50 |
| Chr9:34716853-34716875 | 34716870 | 3 | RNA | GGCCGAGGTCGACTACCGGCNRG | aGCCtAGGTtG--TACaGaCCAG | + | 5 | 2 | 3 | 0.00 | 1.50 |
| Chr2:58230730-58230752 | 58230747 | 3 | RNA | GGCCGAGGTCGACTACCGGCNRG | GaCgaAGGTgGACTA--GtCAGG | + | 5 | 2 | 3 | 0.00 | 1.50 |
| Chr7:66577596-66577618 | 66577603 | 5 | RNA | GGCCGAGGTCGACTACCGGCNRG | GGCCGAGccCGAg--CCGtgGGG | - | 5 | 2 | 3 | 0.00 | 1.50 |
| Chr10:74059386-74059408 | 74059403 | 3 | RNA | GGCCGAGGTCGACTACCGGCNRG | GGgCG-GGcCaACctCCGGCCGG | + | 5 | 1 | 3 | 0.00 | 1.50 |
| Chr1:258847360-258847382 | 258847367 | 5 | RNA | GGCCGAGGTCGACTACCGGCNRG | GGC--AGGaCGcCcAgCGGCAGG | - | 4 | 2 | 3 | 0.00 | 1.50 |
| Chr5:87876141-87876163 | 87876148 | 5 | DNA | GGCCGAGGTCGACTA--CCGGCNRG | tGCaaAGGTCGACcAATCCGGCAGG | - | 4 | 2 | 3 | 0.00 | 1.50 |
| Chr10:22027543-22027565 | 22027560 | 3 | RNA | GGCCGAGGTCGACTACCGGCNRG | G--aGcGGgaGACgACCGGCTGG | + | 5 | 2 | 3 | 0.00 | 1.50 |
| Chr3:132257332-132257354 | 132257349 | 3 | DNA | GGC-CGAGGTCGACTACCGGCNRG | GcCTCGgGGaCGtCTtCCGGCTGG | + | 5 | 1 | 3 | 0.00 | 1.50 |
| Chr4:138186651-138186673 | 138186668 | 3 | RNA | GGCCGAGGTCGACTACCGGCNRG | GGgCGcGG-CGAggtCCGGCCGG | + | 5 | 1 | 3 | 0.00 | 1.50 |
| Chr2:26553003-26553025 | 26553010 | 5 | RNA | GGCCGAGGTCGACTACCGGCNRG | GGCCGAGGTCGgC--CCaGgGAG | - | 3 | 2 | 3 | 0.00 | 1.50 |
| Chr3:205099959-205099981 | 205099966 | 5 | RNA | GGCCGAGGTCGACTACCGGCNRG | tGCtGAGGTCG--TcCCtGaTAG | - | 5 | 2 | 3 | 0.00 | 1.50 |
| Chr3:12054105-12054127 | 12054122 | 3 | RNA | GGCCGAGGTCGACTACCGGCNRG | tGgaGAGGTaGACcAC--GCTGG | + | 5 | 2 | 3 | 0.00 | 1.50 |
| Chr4:9116443-9116465 | 9116450 | 5 | RNA | GGCCGAGGTCGACTACCGGCNRG | cGaCGAGaTCGACcAC--GCCAG | - | 4 | 2 | 3 | 0.00 | 1.50 |
| Chr7:72561296-72561318 | 72561313 | 3 | RNA | GGCCGAGGTCGACTACCGGCNRG | GGgCGAGcTCtt-TAgCGGCGAG | + | 5 | 1 | 3 | 0.00 | 1.50 |
| Chr7:45607765-45607787 | 45607782 | 3 | DNA | GGCCGAGGTCGACTACCGG-CNRG | cGCCGtGGTCGAgcACCGGCgAAG | + | 5 | 1 | 3 | 0.00 | 1.50 |
| Chr9:63648216-63648238 | 63648233 | 3 | RNA | GGCCGAGGTCGACTACCGGCNRG | GGCCcAGaTtGAaT--CGcCCAG | + | 5 | 2 | 3 | 0.00 | 1.50 |
| Chr8:145308585-145308607 | 145308592 | 5 | DNA | GGCCGAGGT--CGACTACCGGCNRG | aGaCGAGGTTCCGACcAagGGCAGG | - | 5 | 2 | 3 | 0.00 | 1.50 |
| Chr6:90307141-90307163 | 90307158 | 3 | DNA | GGCCGAGGTCGAC-TACCGGCNRG | GtCCtcGGaCGACGTgCCGGCCGG | + | 5 | 1 | 3 | 0.00 | 1.50 |
| Chr4:85644323-85644345 | 85644340 | 3 | RNA | GGCCGAGGTCGACTACCGGCNRG | GGggGgGtTCGAC--CgGGCAGG | + | 5 | 2 | 3 | 0.00 | 1.50 |
| Chr6:65905996-65906018 | 65906003 | 5 | RNA | GGCCGAGGTCGACTACCGGCNRG | aGCCGcG--tGAaTtCCGGCCAG | - | 5 | 2 | 3 | 0.00 | 1.50 |
| Chr5:62958383-62958405 | 62958400 | 3 | RNA | GGCCGAGGTCGACTACCGGCNRG | GGgCG-GGcCaACctCCGGCCGG | + | 5 | 1 | 3 | 0.00 | 1.50 |
| Chr3:221386973-221386995 | 221386990 | 3 | RNA | GGCCGAGGTCGACTACCGGCNRG | GtCCG--GTgcACcACCGGaCAG | + | 5 | 2 | 3 | 0.00 | 1.50 |
| Chr9:107556476-107556498 | 107556483 | 5 | RNA | GGCCGAGGTCGACTACCGGCNRG | GtCCG--GTgcACcACCGGaCAG | - | 5 | 2 | 3 | 0.00 | 1.50 |
| Chr8:17884352-17884374 | 17884359 | 5 | RNA | GGCCGAGGTCGACTACCGGCNRG | G-aCGgGGTCGACgACgaGCGGG | - | 5 | 1 | 3 | 0.00 | 1.50 |
| Chr3:200994462-200994484 | 200994479 | 3 | RNA | GGCCGAGGTCGACTACCGGCNRG | GGC-GcaGTCaACggCCGGCGGG | + | 5 | 1 | 3 | 0.00 | 1.50 |
| Chr2:168093613-168093635 | 168093630 | 3 | RNA | GGCCGAGGTCGACTACCGGCNRG | GGC--ttGcgGACTACCGGCTGG | + | 4 | 2 | 3 | 0.00 | 1.50 |
| Chr1:104336389-104336411 | 104336406 | 3 | RNA | GGCCGAGGTCGACTACCGGCNRG | GGCgGAGcTtGACTAC-GGCGAG | + | 3 | 1 | 3 | 0.00 | 1.50 |
| Chr5:204482443-204482465 | 204482450 | 5 | DNA | GGCCGAGGTCGAC-TACCGGCNRG | aGCtGAGGaCGACGTAgCGGgCGG | - | 5 | 1 | 3 | 0.00 | 1.50 |
| Chr5:10862470-10862492 | 10862487 | 3 | RNA | GGCCGAGGTCGACTACCGGCNRG | cGCC-AGGTCGcggACCGtCCGG | + | 5 | 1 | 3 | 0.00 | 1.50 |
| Chr6:75926789-75926811 | 75926806 | 3 | RNA | GGCCGAGGTCGACTACCGGCNRG | G-CtGAGaTCGcCTAgCtGCGGG | + | 5 | 1 | 3 | 0.00 | 1.50 |
| Chr7:38878640-38878662 | 38878657 | 3 | RNA | GGCCGAGGTCGACTACCGGCNRG | caCCGAGtTgGAgTA--GGCGGG | + | 5 | 2 | 3 | 0.00 | 1.50 |
| Chr3:160758395-160758417 | 160758412 | 3 | RNA | GGCCGAGGTCGACTACCGGCNRG | cGaCGAGcTaGACT-CCGGtGAG | + | 5 | 1 | 3 | 0.00 | 1.50 |
| Chr10:53898069-53898091 | 53898086 | 3 | DNA | G--GCCGAGGTCGACTACCGGCNRG | GCTGCCGAGGTCGAaTgtCtcCGGG | + | 5 | 2 | 3 | 0.00 | 1.50 |
| Chr7:97736536-97736558 | 97736553 | 3 | RNA | GGCCGAGGTCGACTACCGGCNRG | GGtCGgcGgCGAC-AgCGGCAGG | + | 5 | 1 | 3 | 0.00 | 1.50 |
| Chr2:88893807-88893829 | 88893814 | 5 | RNA | GGCCGAGGTCGACTACCGGCNRG | GG--GAcaTCcgCTACCtGCGGG | - | 5 | 2 | 3 | 0.00 | 1.50 |
| Chr4:132618196-132618218 | 132618213 | 3 | RNA | GGCCGAGGTCGACTACCGGCNRG | GaCtGcGGcCGACTAC-GGaGGG | + | 5 | 1 | 3 | 0.00 | 1.50 |
| Chr2:121424822-121424844 | 121424829 | 5 | RNA | GGCCGAGGTCGACTACCGGCNRG | tGCaGAGGTCcACa--CtGCAGG | - | 5 | 2 | 3 | 0.00 | 1.50 |
| Chr1:119438081-119438103 | 119438098 | 3 | RNA | GGCCGAGGTCGACTACCGGCNRG | GGCaGtGGct--CTAaCGGCGAG | + | 5 | 2 | 3 | 0.00 | 1.50 |
| Chr8:93256144-93256166 | 93256151 | 5 | RNA | GGCCGAGGTCGACTACCGGCNRG | GGCCt--GcCaACTtCCGGgCAG | - | 5 | 2 | 3 | 0.00 | 1.50 |
| Chr3:68340422-68340444 | 68340429 | 5 | RNA | GGCCGAGGTCGACTACCGGCNRG | GGC-GAGaTCGACTtggGGaAAG | - | 5 | 1 | 3 | 0.00 | 1.50 |
| Chr2:116257352-116257374 | 116257359 | 5 | RNA | GGCCGAGGTCGACTACCGGCNRG | GaCgaAGGTgGACTA--GtCAGG | - | 5 | 2 | 3 | 0.00 | 1.50 |
| Chr5:70429653-70429675 | 70429660 | 5 | RNA | GGCCGAGGTCGACTACCGGCNRG | GGCCGAGGgtGcCT-CgGGCTGG | - | 4 | 1 | 3 | 0.00 | 1.50 |
| Chr1:119107875-119107897 | 119107892 | 3 | RNA | GGCCGAGGTCGACTACCGGCNRG | G-aCGgGGTCGACgACgaGCGGG | + | 5 | 1 | 3 | 0.00 | 1.50 |
| Chr4:187429626-187429648 | 187429643 | 3 | RNA | GGCCGAGGTCGACTACCGGCNRG | G--CGAGGTCGACgACgcGaTGG | + | 4 | 2 | 3 | 0.00 | 1.50 |
| Chr7:64653817-64653839 | 64653834 | 3 | RNA | GGCCGAGGTCGACTACCGGCNRG | GGCgGAGGTgtgCT-CgGGCCGG | + | 5 | 1 | 3 | 0.00 | 1.50 |
| Chr8:64332994-64333016 | 64333001 | 5 | RNA | GGCCGAGGTCGACTACCGGCNRG | aGCCcAGGaCG--TACaaGCTGG | - | 5 | 2 | 3 | 0.00 | 1.50 |

| | | | | | | | | | | | |
|---|---|---|---|---|---|---|---|---|---|---|---|
| Chr10:78654684-78654706 | 78654691 | 5 | RNA | GGCCGAGGTCGACTACCGGCNRG | GGC-GAGGTCGAggACCttgGGG | - | 5 | 1 | 3 | 0.00 | 1.50 |
| Chr1:267012530-267012552 | 267012537 | 5 | RNA | GGCCGAGGTCGACTACCGGCNRG | G-aCGgGGTCGACaACgaGCGGG | - | 5 | 1 | 3 | 0.00 | 1.50 |
| Chr10:125820073-125820095 | 125820080 | 5 | DNA | GGCCGAGGTCGACTACC--GGCNRG | GGCgGAGGTCGtCggCaGAGGCCAG | - | 5 | 2 | 3 | 0.00 | 1.50 |
| Chr4:94800265-94800287 | 94800272 | 5 | RNA | GGCCGAGGTCGACTACCGGCNRG | tGCgGgGcTCGACTAC-GGaCGG | - | 5 | 1 | 3 | 0.00 | 1.50 |
| Chr4:10013239-10013261 | 10013256 | 3 | RNA | GGCCGAGGTCGACTACCGGCNRG | ttCCGAtGcCGAtTA--GGCAGG | + | 5 | 2 | 3 | 0.00 | 1.50 |
| Chr9:120983383-120983405 | 120983400 | 3 | DNA | GGCCGAGGTCGA--CTACCGGCNRG | GGgCGAcGaCGATTCTACtaGCCAG | + | 5 | 2 | 3 | 0.00 | 1.50 |
| Chr4:130009606-130009628 | 130009613 | 5 | RNA | GGCCGAGGTCGACTACCGGCNRG | GGgCGAGGTC-ACgcgCGGgCAG | - | 5 | 1 | 3 | 0.00 | 1.50 |
| Chr4:6496967-6496989 | 6496984 | 3 | RNA | GGCCGAGGTCGACTACCGGCNRG | GGCgGcGGgCG--gACaGGCGGG | + | 5 | 2 | 3 | 0.00 | 1.50 |
| Chr6:36624904-36624926 | 36624911 | 5 | RNA | GGCCGAGGTCGACTACCGGCNRG | tGCCGAGGTCG--TAttGctGGG | - | 5 | 2 | 3 | 0.00 | 1.50 |
| Chr5:101643973-101643995 | 101643980 | 5 | RNA | GGCCGAGGTCGACTACCGGCNRG | GGC--ttGcgGACTACtGGCTAG | - | 5 | 2 | 3 | 0.00 | 1.50 |
| Chr2:222670902-222670924 | 222670919 | 3 | RNA | GGCCGAGGTCGACTACCGGCNRG | G--tGcGGgCGAgTtCCGGCGGG | + | 5 | 2 | 3 | 0.00 | 1.50 |
| Chr7:21137739-21137761 | 21137756 | 3 | DNA | GGCCGAGGTC-GACTACCGGCNRG | GGCtGAGGcCTGACTACCtGaGGG | + | 4 | 1 | 3 | 0.00 | 1.50 |
| Chr8:172012767-172012789 | 172012784 | 3 | RNA | GGCCGAGGTCGACTACCGGCNRG | caCC-AGGTCGcCTACCtGgAGG | + | 5 | 1 | 3 | 0.00 | 1.50 |
| Chr3:54020223-54020245 | 54020230 | 5 | RNA | GGCCGAGGTCGACTACCGGCNRG | tGtCG--GaCGAaTACgGGCAGG | - | 5 | 2 | 3 | 0.00 | 1.50 |
| Chr4:121470030-121470052 | 121470037 | 5 | RNA | GGCCGAGGTCGACTACCGGCNRG | G-CCtcGGgCGAgTACtGGCAGG | - | 5 | 1 | 3 | 0.00 | 1.50 |
| Chr6:168809714-168809736 | 168809731 | 3 | RNA | GGCCGAGGTCGACTACCGGCNRG | GGCgGAGG-CGgCTAtgGaCTGG | + | 5 | 1 | 3 | 0.00 | 1.50 |
| Chr1:42321946-42321968 | 42321963 | 3 | RNA | GGCCGAGGTCGACTACCGGCNRG | tGCCGAGGTgG--TggCaGCTGG | + | 5 | 2 | 3 | 0.00 | 1.50 |
| Chr7:26196527-26196549 | 26196544 | 3 | RNA | GGCCGAGGTCGACTACCGGCNRG | GaCaG-GGTCGACaACgaGCGGG | + | 5 | 1 | 3 | 0.00 | 1.50 |
| Chr3:183744311-183744333 | 183744318 | 5 | RNA | GGCCGAGGTCGACTACCGGCNRG | GGCCGAGGgCG--cgCgcGCGGG | - | 5 | 2 | 3 | 0.00 | 1.50 |
| Chr1:2335196-2335218 | 2335213 | 3 | RNA | GGCCGAGGTCGACTACCGGCNRG | G-aCGgGGTCGACgACgaGCGGG | + | 5 | 1 | 3 | 0.00 | 1.50 |
| Chr9:81333146-81333168 | 81333153 | 5 | RNA | GGCCGAGGTCGACTACCGGCNRG | GGC--tcGcgGACTACCGGCTGG | - | 4 | 2 | 3 | 0.00 | 1.50 |
| Chr1:61110248-61110270 | 61110265 | 3 | RNA | GGCCGAGGTCGACTACCGGCNRG | GGC--gGGcCaACctCCGGCCGG | + | 5 | 2 | 3 | 0.00 | 1.50 |
| Chr5:11950623-11950645 | 11950640 | 3 | DNA | GG-CCGAGGTCGACTACCGGCNRG | GGTaCGAGGgCGACacCCaGCAAG | + | 5 | 1 | 3 | 0.00 | 1.50 |
| Chr2:51023454-51023476 | 51023461 | 5 | RNA | GGCCGAGGTCGACTACCGGCNRG | G-CCtcGGgCGAgTACtGGCAGG | - | 5 | 1 | 3 | 0.00 | 1.50 |
| Chr6:70088222-70088244 | 70088239 | 3 | RNA | GGCCGAGGTCGACTACCGGCNRG | GGCaGAaG--GACTcCgGGCCGG | + | 4 | 2 | 3 | 0.00 | 1.50 |
| Chr1:43873663-43873685 | 43873680 | 3 | RNA | GGCCGAGGTCGACTACCGGCNRG | tGgCGAGGTCGAggACC--tTGG | + | 5 | 2 | 3 | 0.00 | 1.50 |
| Chr1:165704390-165704412 | 165704407 | 3 | RNA | GGCCGAGGTCGACTACCGGCNRG | G-aCGgGGTCGACgACgaGCGGG | + | 5 | 1 | 3 | 0.00 | 1.50 |
| Chr5:122722957-122722979 | 122722964 | 5 | DNA | GGCCGAGGTCGACTAC--CGGCNRG | GGCgGgtGTtGgCTACTACGGCTAG | - | 5 | 2 | 3 | 0.00 | 1.50 |
| Chr1:150154701-150154723 | 150154718 | 3 | RNA | GGCCGAGGTCGACTACCGGCNRG | tGCaGgGcTCGACTA--GGtTGG | + | 5 | 2 | 3 | 0.00 | 1.50 |
| Chr4:95396893-95396915 | 95396900 | 5 | RNA | GGCCGAGGTCGACTACCGGCNRG | GGgCGgGGTCGgCT-CtGGgAGG | - | 5 | 1 | 3 | 0.00 | 1.50 |
| Chr5:115739814-115739836 | 115739821 | 5 | RNA | GGCCGAGGTCGACTACCGGCNRG | GGCtGgGcTgGACTA--GGtTGG | - | 5 | 2 | 3 | 0.00 | 1.50 |
| Chr2:164461449-164461471 | 164461456 | 5 | RNA | GGCCGAGGTCGACTACCGGCNRG | GGgCGAGGgCGA--AggGGgGAG | - | 5 | 2 | 3 | 0.00 | 1.50 |
| Chr2:160086853-160086875 | 160086860 | 5 | RNA | GGCCGAGGTCGACTACCGGCNRG | GGggGAGGTgG--TAgaGGCTGG | - | 5 | 2 | 3 | 0.00 | 1.50 |
| Chr5:22807892-22807914 | 22807899 | 5 | RNA | GGCCGAGGTCGACTACCGGCNRG | GGCtGAacTCG-CTACCttCGGG | - | 5 | 1 | 3 | 0.00 | 1.50 |
| Chr9:35485798-35485820 | 35485805 | 5 | RNA | GGCCGAGGTCGACTACCGGCNRG | GGCCGtctTCGAgTA--GtCCGG | - | 5 | 2 | 3 | 0.00 | 1.50 |
| Chr2:17754457-17754479 | 17754474 | 3 | RNA | GGCCGAGGTCGACTACCGGCNRG | GtCatAGGTC--CTAtCGcCGAG | + | 5 | 2 | 3 | 0.00 | 1.50 |
| Chr9:7548854-7548876 | 7548861 | 5 | RNA | GGCCGAGGTCGACTACCGGCNRG | GGtCGAGGgtGcCT-CgGGCTGG | - | 5 | 1 | 3 | 0.00 | 1.50 |
| Chr5:85959665-85959687 | 85959672 | 5 | RNA | GGCCGAGGTCGACTACCGGCNRG | GcCgGAGccCGAC--CCGtCAAG | - | 5 | 2 | 3 | 0.00 | 1.50 |
| Chr1:119836610-119836632 | 119836617 | 5 | RNA | GGCCGAGGTCGACTACCGGCNRG | GGC-GAGGTCGACgACgaGaTAG | - | 4 | 1 | 3 | 0.00 | 1.50 |
| Chr2:5972866-5972888 | 5972883 | 3 | RNA | GGCCGAGGTCGACTACCGGCNRG | tGCCGtGGcCt--TcCCGGCCGG | + | 5 | 2 | 3 | 0.00 | 1.50 |
| Chr3:38682391-38682413 | 38682398 | 5 | RNA | GGCCGAGGTCGACTACCGGCNRG | aGCCacGGTCaACTAgC--CAGG | - | 5 | 2 | 3 | 0.00 | 1.50 |
| Chr6:135776647-135776669 | 135776654 | 5 | DNA | GGC--CGAGGTCGACTACCGGCNRG | GGCCTCGgGGgCtACacCCGGCGGG | - | 5 | 2 | 3 | 0.00 | 1.50 |
| Chr6:170124276-170124298 | 170124283 | 5 | RNA | GGCCGAGGTCGACTACCGGCNRG | GGCgGAGcTCGgCTtC-GtCGAG | - | 5 | 1 | 3 | 0.00 | 1.50 |
| Chr1:18355817-18355839 | 18355834 | 3 | RNA | GGCCGAGGTCGACTACCGGCNRG | cGCCa--GTCGtgTACCGGgAAG | + | 5 | 2 | 3 | 0.00 | 1.50 |
| Chr9:144412821-144412843 | 144412828 | 5 | RNA | GGCCGAGGTCGACTACCGGCNRG | tGCCGtGGcCt--TcCCGGCCGG | - | 5 | 2 | 3 | 0.00 | 1.50 |
| Chr7:124852216-124852238 | 124852233 | 3 | RNA | GGCCGAGGTCGACTACCGGCNRG | GGC--ttGcgGACTAtCGGCTGG | + | 5 | 2 | 3 | 0.00 | 1.50 |
| Chr8:149453620-149453642 | 149453627 | 5 | RNA | GGCCGAGGTCGACTACCGGCNRG | GG--GAGGTCGAggACCaGgAGG | - | 4 | 2 | 3 | 0.00 | 1.50 |
| Chr5:2510066-2510088 | 2510083 | 3 | RNA | GGCCGAGGTCGACTACCGGCNRG | GGC--ttGcgGACTACCGGCTGG | + | 4 | 2 | 3 | 0.00 | 1.50 |
| Chr3:5740871-5740893 | 5740888 | 3 | RNA | GGCCGAGGTCGACTACCGGCNRG | caCCaAGtTCGcCT-CCGGCCAG | + | 5 | 1 | 3 | 0.00 | 1.50 |
| Chr9:113641452-113641474 | 113641459 | 5 | DNA | GGCCGAGG-TCGACTACCGGCNRG | GGCtGtctTTCcACTACCGGCTGG | - | 5 | 1 | 3 | 0.00 | 1.50 |
| Chr4:176923490-176923512 | 176923497 | 5 | RNA | GGCCGAGGTCGACTACCGGCNRG | GGC--AGGcCaACctCCGGCCGG | - | 4 | 2 | 3 | 0.00 | 1.50 |
| Chr4:211891563-211891585 | 211891580 | 3 | DNA | GGCCGAGGTCGACTACCG-GCNRG | GGaCGAGGTCtcCTACtGAGaAGG | + | 5 | 1 | 3 | 0.00 | 1.50 |
| Chr2:124461429-124461451 | 124461446 | 3 | RNA | GGCCGAGGTCGACTACCGGCNRG | GGtCGA--TCtACTcCtGGgAAG | + | 5 | 2 | 3 | 0.00 | 1.50 |
| Chr2:54091487-54091509 | 54091504 | 3 | RNA | GGCCGAGGTCGACTACCGGCNRG | cGCCGAaGTCGAtcA--GtCGAG | + | 5 | 2 | 3 | 0.00 | 1.50 |
| Chr1:147825700-147825722 | 147825717 | 3 | RNA | GGCCGAGGTCGACTACCGGCNRG | GGggGgGGgCGA--ACCGGtCGG | + | 5 | 2 | 3 | 0.00 | 1.50 |
| Chr10:128745627-128745649 | 128745634 | 5 | RNA | GGCCGAGGTCGACTACCGGCNRG | GcCgGAGccCGAC--CCGtCAGG | - | 5 | 2 | 3 | 0.00 | 1.50 |
| Chr4:29567025-29567047 | 29567042 | 3 | DNA | GGCCGAGGTCGACTACC--GGCNRG | GGCgGAGGTCGtCggCaGAGGCCAG | + | 5 | 2 | 3 | 0.00 | 1.50 |
| Chr6:132897142-132897164 | 132897159 | 3 | RNA | GGCCGAGGTCGACTACCGGCNRG | GaCaG-GGTCGACgACaaGCGGG | + | 5 | 1 | 3 | 0.00 | 1.50 |
| Chr7:81881669-81881691 | 81881676 | 5 | DNA | GGCCGAGGTCGACT-ACCGGCNRG | GGgCGAGaTgGtCTGACCtGCCGG | - | 5 | 1 | 3 | 0.00 | 1.50 |
| Chr2:60223890-60223912 | 60223897 | 5 | RNA | GGCCGAGGTCGACTACCGGCNRG | GGCCG--GTCtACcAgCGagAGG | - | 5 | 2 | 3 | 0.00 | 1.50 |
| Chr10:112183284-112183306 | 112183291 | 5 | RNA | GGCCGAGGTCGACTACCGGCNRG | aGCaGAG--CGAacgCCGGCGGG | - | 5 | 2 | 3 | 0.00 | 1.50 |
| Chr9:32186804-32186826 | 32186811 | 5 | DNA | GGCCGAGGTCGACT-ACCGGCNRG | cGCCGtGGaCatCTCACCGGCGGG | - | 5 | 1 | 3 | 0.00 | 1.50 |
| Chr10:88430537-88430559 | 88430544 | 5 | RNA | GGCCGAGGTCGACTACCGGCNRG | GGC--AGGcCGAgcACCaGCCAG | - | 4 | 2 | 3 | 0.00 | 1.50 |
| Chr5:195505904-195505926 | 195505911 | 5 | RNA | GGCCGAGGTCGACTACCGGCNRG | GaCtGcGGcCGtCT--CGGCTGG | - | 5 | 2 | 3 | 0.00 | 1.50 |
| Chr4:186335262-186335284 | 186335269 | 5 | RNA | GGCCGAGGTCGACTACCGGCNRG | GGCCctGGc--ACTACgGGaTGG | - | 5 | 2 | 3 | 0.00 | 1.50 |
| Chr7:177237341-177237363 | 177237358 | 3 | RNA | GGCCGAGGTCGACTACCGGCNRG | cGCCGAGGcCGgggA--GGCCAG | + | 5 | 2 | 3 | 0.00 | 1.50 |
| Chr5:201524946-201524968 | 201524953 | 5 | RNA | GGCCGAGGTCGACTACCGGCNRG | atCCtgGGT-GACcACCGGCGGG | - | 5 | 1 | 3 | 0.00 | 1.50 |
| Chr5:27832865-27832887 | 27832872 | 5 | RNA | GGCCGAGGTCGACTACCGGCNRG | GGC--AGGcCaACctCCGGCCGG | - | 4 | 2 | 3 | 0.00 | 1.50 |
| Chr1:181021666-181021688 | 181021673 | 5 | RNA | GGCCGAGGTCGACTACCGGCNRG | GGC--ttGcgGACTACCGGCTGG | - | 4 | 2 | 3 | 0.00 | 1.50 |
| Chr8:15550544-15550566 | 15550561 | 3 | RNA | GGCCGAGGTCGACTACCGGCNRG | G-CCGAGGTCGAggtCgGGCGAG | + | 4 | 1 | 3 | 0.00 | 1.50 |
| Chr4:204248123-204248145 | 204248140 | 3 | RNA | GGCCGAGGTCGACTACCGGCNRG | cGCCGActgCGACg--CGGCTAG | + | 5 | 2 | 3 | 0.00 | 1.50 |
| Chr1:28789432-28789454 | 28789439 | 5 | RNA | GGCCGAGGTCGACTACCGGCNRG | GcCCtAGGgC--CcACCGGtCAG | - | 5 | 2 | 3 | 0.00 | 1.50 |
| Chr8:148618540-148618562 | 148618557 | 3 | RNA | GGCCGAGGTCGACTACCGGCNRG | GtCCG--GTgcACcACCGGaCAG | + | 5 | 2 | 3 | 0.00 | 1.50 |
| Chr1:93501870-93501892 | 93501877 | 5 | RNA | GGCCGAGGTCGACTACCGGCNRG | GGC--AGGcCGAgcACtGGCCGG | - | 4 | 2 | 3 | 0.00 | 1.50 |
| Chr1:102556097-102556119 | 102556114 | 3 | RNA | GGCCGAGGTCGACTACCGGCNRG | GtCCG--GTgcACcACCGGaCAG | + | 5 | 2 | 3 | 0.00 | 1.50 |
| Chr6:19490954-19490976 | 19490961 | 5 | RNA | GGCCGAGGTCGACTACCGGCNRG | GGaCGAGGcgcACaAC--GCTAG | - | 5 | 2 | 3 | 0.00 | 1.50 |
| Chr1:4946356-4946378 | 4946363 | 5 | RNA | GGCCGAGGTCGACTACCGGCNRG | GaCCGAGG-CGACgACaaGaAGG | - | 5 | 1 | 3 | 0.00 | 1.50 |
| Chr8:135074276-135074298 | 135074293 | 3 | RNA | GGCCGAGGTCGACTACCGGCNRG | GaCCGAGGT-GACgACaaGaAGG | + | 5 | 1 | 3 | 0.00 | 1.50 |
| Chr6:36494689-36494711 | 36494696 | 5 | RNA | GGCCGAGGTCGACTACCGGCNRG | GGCaGctGTC--CTtCCGcCAGG | - | 5 | 2 | 3 | 0.00 | 1.50 |
| Chr8:103269032-103269054 | 103269049 | 3 | RNA | GGCCGAGGTCGACTACCGGCNRG | GcCaGcGGTCGA--AagGGCGGG | + | 5 | 2 | 3 | 0.00 | 1.50 |
| Chr8:53054923-53054945 | 53054940 | 3 | RNA | GGCCGAGGTCGACTACCGGCNRG | G-aCGgGGTCGACgACgaGCGGG | + | 5 | 1 | 3 | 0.00 | 1.50 |
| Chr5:42636727-42636749 | 42636744 | 3 | RNA | GGCCGAGGTCGACTACCGGCNRG | ctCCaAGGTCGtC-gCCGGCCAG | + | 5 | 1 | 3 | 0.00 | 1.50 |
| Chr1:190416490-190416512 | 190416497 | 5 | RNA | GGCCGAGGTCGACTACCGGCNRG | GGC--tcGcgGACTACCGaCTGG | - | 5 | 2 | 3 | 0.00 | 1.50 |
| Chr7:65705760-65705782 | 65705767 | 5 | RNA | GGCCGAGGTCGACTACCGGCNRG | GGC--tcGctGACTACCGtCTGG | - | 5 | 2 | 3 | 0.00 | 1.50 |
| Chr8:82885005-82885027 | 82885022 | 3 | RNA | GGCCGAGGTCGACTACCGGCNRG | cGgCGAGGTtG--TggCGGCGAG | + | 5 | 2 | 3 | 0.00 | 1.50 |
| Chr1:114525209-114525231 | 114525216 | 5 | RNA | GGCCGAGGTCGACTACCGGCNRG | GGCCGAtcTCG--cAtCaGCAAG | - | 5 | 2 | 3 | 0.00 | 1.50 |
| Chr8:84291806-84291828 | 84291813 | 5 | RNA | GGCCGAGGTCGACTACCGGCNRG | GGCgcAacTCtACT-CCGGCTAG | - | 5 | 1 | 3 | 0.00 | 1.50 |
| Chr1:56666526-56666548 | 56666533 | 5 | DNA | GGC-CGAGGTCGACTACCGGCNRG | GcCTCGgGGaCGACTtCCGGtTGG | - | 5 | 1 | 3 | 0.00 | 1.50 |
| Chr5:62925596-62925618 | 62925603 | 5 | X | GGCCGAGGTCGACTACCGGCNRG | GcCCGAGGTCaACctCgGGCGGG | - | 5 | 0 | 3 | 0.00 | 1.50 |
| Chr2:12763024-12763046 | 12763041 | 3 | RNA | GGCCGAGGTCGACTACCGGCNRG | GGtCcAGGgC--CTtCCtGCGAG | + | 5 | 2 | 3 | 0.00 | 1.50 |
| Chr6:172063662-172063684 | 172063669 | 5 | DNA | GGC-CGAGGTCGACTACCGGCNRG | GcCTCGgGGaCGtCTtCCGGCTGG | - | 5 | 1 | 3 | 0.00 | 1.50 |
| Chr1:190593432-190593454 | 190593449 | 3 | RNA | GGCCGAGGTCGACTACCGGCNRG | ttCCGAtGTCGAtTA--tGCCGG | + | 5 | 2 | 3 | 0.00 | 1.50 |
| Chr2:54183719-54183741 | 54183736 | 3 | DNA | GGC-CGAGGTCGACTACCGGCNRG | GcCTCGgGGaCGtCTtCCGGCTGG | + | 5 | 1 | 3 | 0.00 | 1.50 |
| Chr1:266876252-266876274 | 266876269 | 3 | RNA | GGCCGAGGTCGACTACCGGCNRG | G-aCGgGGTCGACgACgaGCGGG | + | 5 | 1 | 3 | 0.00 | 1.50 |
| Chr5:162335113-162335135 | 162335120 | 5 | DNA | GGCCGAGGTCGACTACCGG--CNRG | GGtCGAGGTCGAgTcCgaGCCCCGG | - | 5 | 2 | 3 | 0.00 | 1.50 |
| Chr4:96949999-96950021 | 96950016 | 3 | RNA | GGCCGAGGTCGACTACCGGCNRG | tGCgcAGGTCGACatCC--CTGG | + | 5 | 2 | 3 | 0.00 | 1.50 |
| Chr2:11257790-11257812 | 11257807 | 3 | RNA | GGCCGAGGTCGACTACCGGCNRG | GcCCGAGGcCGA--ACttGgTGG | + | 5 | 2 | 3 | 0.00 | 1.50 |
| Chr5:188062070-188062092 | 188062087 | 3 | RNA | GGCCGAGGTCGACTACCGGCNRG | GGaCtAGGTCG--TcCCaGtTGG | + | 5 | 2 | 3 | 0.00 | 1.50 |
| Chr5:27782968-27782990 | 27782975 | 5 | RNA | GGCCGAGGTCGACTACCGGCNRG | GGgCG-GGcCaACctCCGGCCGG | - | 5 | 1 | 3 | 0.00 | 1.50 |
| Chr2:124722901-124722923 | 124722918 | 3 | RNA | GGCCGAGGTCGACTACCGGCNRG | aGCCGAtcTCcAC--CCaGCCAG | + | 5 | 2 | 3 | 0.00 | 1.50 |
| Chr9:105929883-105929905 | 105929890 | 5 | RNA | GGCCGAGGTCGACTACCGGCNRG | GGaCtAGGTCG--TcCCaGtTGG | - | 5 | 2 | 3 | 0.00 | 1.50 |
| Chr8:42910028-42910050 | 42910045 | 3 | RNA | GGCCGAGGTCGACTACCGGCNRG | cG--GtGGcCGACTctCGGCTGG | + | 5 | 2 | 3 | 0.00 | 1.50 |
| Chr2:75928676-75928698 | 75928693 | 3 | RNA | GGCCGAGGTCGACTACCGGCNRG | GtCCGAGGTC--CTtagGaCAAG | + | 5 | 2 | 3 | 0.00 | 1.50 |
| Chr10:26275850-26275872 | 26275857 | 5 | RNA | GGCCGAGGTCGACTACCGGCNRG | GGCtcAcG--GACTACCGtCTGG | - | 4 | 2 | 3 | 0.00 | 1.50 |
| Chr2:109061746-109061768 | 109061763 | 3 | RNA | GGCCGAGGTCGACTACCGGCNRG | GGC--AGcctGACgACCGtCTGG | + | 5 | 2 | 3 | 0.00 | 1.50 |
| Chr4:239222933-239222955 | 239222950 | 3 | RNA | GGCCGAGGTCGACTACCGGCNRG | GGC--tcGcgGACTACCGGCTGG | + | 4 | 2 | 3 | 0.00 | 1.50 |
| Chr5:82742673-82742695 | 82742690 | 3 | DNA | GGC-CGAGGTCGACTACCGGCNRG | GcCTCGAGGgCtACacCCGGCGGG | + | 5 | 1 | 3 | 0.00 | 1.50 |
| Chr3:52847749-52847771 | 52847766 | 3 | RNA | GGCCGAGGTCGACTACCGGCNRG | GGCCatGGctG--TAaCGGCCGG | + | 5 | 2 | 3 | 0.00 | 1.50 |
| Chr2:209578199-209578221 | 209578216 | 3 | RNA | GGCCGAGGTCGACTACCGGCNRG | GGaCcAGGTCG--TcCCaGtTGG | + | 5 | 2 | 3 | 0.00 | 1.50 |
| Chr6:120885104-120885126 | 120885121 | 3 | RNA | GGCCGAGGTCGACTACCGGCNRG | aGgCGAGcTCGAgTA--GGgAAG | + | 5 | 2 | 3 | 0.00 | 1.50 |
| Chr3:133752817-133752839 | 133752834 | 3 | RNA | GGCCGAGGTCGACTACCGGCNRG | aGC--AGGcCaACTACCGctTGG | + | 5 | 2 | 3 | 0.00 | 1.50 |
| Chr5:141772940-141772962 | 141772957 | 3 | RNA | GGCCGAGGTCGACTACCGGCNRG | tGaCcAGGTCGAC--CCaGtCGG | + | 5 | 2 | 3 | 0.00 | 1.50 |
| Chr4:82715624-82715646 | 82715631 | 5 | DNA | GGC--CGAGGTCGACTACCGGCNRG | GcCAACGcGGTCGAggACtGGCGGG | - | 5 | 2 | 3 | 0.00 | 1.50 |
| Chr7:24225185-24225207 | 24225202 | 3 | DNA | GGCCGAGGTCGA--CTACCGGCNRG | GcgCcAtcTCGATTCTACCGGCGAG | + | 5 | 2 | 3 | 0.00 | 1.50 |
| Chr4:92024077-92024099 | 92024084 | 5 | RNA | GGCCGAGGTCGACTACCGGCNRG | GGCgcAcaTCGACT-CtGGCAAG | - | 5 | 1 | 3 | 0.00 | 1.50 |
| Chr5:10078589-10078611 | 10078596 | 5 | DNA | GGC--CGAGGTCGACTACCGGCNRG | GaCAACGgGGTCGACgACgaGCGGG | - | 5 | 2 | 3 | 0.00 | 1.50 |
| Chr6:66953869-66953891 | 66953876 | 5 | RNA | GGCCGAGGTCGACTACCGGCNRG | GGCCcAcGaCG--TttCGGCTGG | - | 5 | 2 | 3 | 0.00 | 1.50 |
| Chr3:65067584-65067606 | 65067591 | 5 | RNA | GGCCGAGGTCGACTACCGGCNRG | G-aCGgGGTCGACaACgaGCGGG | - | 5 | 1 | 3 | 0.00 | 1.50 |
| Chr2:8301729-8301751 | 8301746 | 3 | DNA | GGCC-GAGGTCGACTACCGGCNRG | GGCCAGAGcgtGAtTAaCGGCTGG | + | 5 | 1 | 3 | 0.00 | 1.50 |
| Chr4:25469952-25469974 | 25469969 | 3 | RNA | GGCCGAGGTCGACTACCGGCNRG | G-aCGgGGTCGACaACgaGCGGG | + | 5 | 1 | 3 | 0.00 | 1.50 |
| Chr5:116664173-116664195 | 116664190 | 3 | RNA | GGCCGAGGTCGACTACCGGCNRG | tGCCcAGG--GACaAgCGGgCAG | + | 5 | 2 | 3 | 0.00 | 1.50 |
| Chr1:210041938-210041960 | 210041945 | 5 | RNA | GGCCGAGGTCGACTACCGGCNRG | GGCgGAGGTaGcCcA--GGCCAG | - | 4 | 2 | 3 | 0.00 | 1.50 |
| Chr3:187183718-187183740 | 187183725 | 5 | RNA | GGCCGAGGTCGACTACCGGCNRG | GGCCtAcGgC--CTACaGGgCGG | - | 5 | 2 | 3 | 0.00 | 1.50 |
| Chr9:132756515-132756537 | 132756532 | 3 | RNA | GGCCGAGGTCGACTACCGGCNRG | GGtCGAcGgCGACaACa--CGAG | + | 5 | 2 | 3 | 0.00 | 1.50 |
| Chr2:154133748-154133770 | 154133765 | 3 | RNA | GGCCGAGGTCGACTACCGGCNRG | GGC--tcGcgGACTACCGtCTGG | + | 5 | 2 | 3 | 0.00 | 1.50 |
| Chr4:61672270-61672292 | 61672287 | 3 | RNA | GGCCGAGGTCGACTACCGGCNRG | caCCGAGGTCGtCTtCaG-CAGG | + | 5 | 1 | 3 | 0.00 | 1.50 |

| | | | | | | | | | | | |
|---|---|---|---|---|---|---|---|---|---|---|---|
| Chr1:101957580-101957602 | 101957587 | 5 | RNA | GGCCGAGGTCGACTACCGGCNRG | GGgCGAGGcgGACcACC--gTGG | - | 5 | 2 | 3 | 0.00 | 1.50 |
| Chr10:57906985-57907007 | 57907002 | 3 | RNA | GGCCGAGGTCGACTACCGGCNRG | GGCCtcGGaCGgCgAC--GCAGG | + | 5 | 2 | 3 | 0.00 | 1.50 |
| Chr2:197149932-197149954 | 197149949 | 3 | RNA | GGCCGAGGTCGACTACCGGCNRG | GGC--tcGcgGACTACCGGCTGG | + | 4 | 2 | 3 | 0.00 | 1.50 |
| Chr1:214293557-214293579 | 214293564 | 5 | DNA | GG-CCGAGGTCGACTACCGGCNRG | GGTaCGAGGgCGACgtCCtGCAAG | - | 5 | 1 | 3 | 0.00 | 1.50 |
| Chr4:77977161-77977183 | 77977178 | 3 | RNA | GGCCGAGGTCGACTACCGGCNRG | GGgaGAG--aGAgTAaCGGCTGG | + | 5 | 2 | 3 | 0.00 | 1.50 |
| Chr3:215419943-215419965 | 215419950 | 5 | RNA | GGCCGAGGTCGACTACCGGCNRG | GGCCG-GtaCGAaTcaCGGCAGG | - | 5 | 1 | 3 | 0.00 | 1.50 |
| Chr1:99480813-99480835 | 99480820 | 5 | RNA | GGCCGAGGTCGACTACCGGCNRG | GGCCGtGtTCatCTgCC--CAAG | - | 5 | 2 | 3 | 0.00 | 1.50 |
| Chr5:214299556-214299578 | 214299563 | 5 | RNA | GGCCGAGGTCGACTACCGGCNRG | GaaaGAGGTCGtCT-CCGGaAAG | - | 5 | 1 | 3 | 0.00 | 1.50 |
| Chr7:13713100-13713122 | 13713107 | 5 | RNA | GGCCGAGGTCGACTACCGGCNRG | aaCCcAGGT--ACcACCaGCCAG | - | 5 | 2 | 3 | 0.00 | 1.50 |
| Chr1:214344093-214344115 | 214344109 | 4 | RNA | GGCCGAGGTCGACTACCGGCNRG | tG--GcGGcCGACaACCcGCCAG | + | 5 | 2 | 0 | 2.75 | 1.38 |
| Chr3:150252466-150252488 | 150252471 | 3 | RNA | GGCCGAGGTCGACTACCGGCNRG | tGCCGA--TacACcgCCGGCCGG | - | 5 | 2 | 0 | 2.75 | 1.38 |
| Chr8:151972397-151972419 | 151972404 | 5 | DNA | GGCCGAGGTCGACTAC-CGGCNRG | GGgCGAGGcCGgCggCGCGGCGGG | - | 5 | 1 | 0 | 2.75 | 1.38 |
| Chr3:102002994-102003016 | 102003010 | 4 | RNA | GGCCGAGGTCGACTACCGGCNRG | G-aCGAGGTCaACaACgaGCGGG | + | 5 | 1 | 0 | 2.75 | 1.38 |
| Chr10:105202045-105202067 | 105202063 | 2 | RNA | GGCCGAGGTCGACTACCGGCNRG | GcgCGcGG--GgCTgCCGGCCGG | + | 5 | 2 | 0 | 2.75 | 1.38 |
| Chr1:90735162-90735184 | 90735169 | 5 | RNA | GGCCGAGGTCGACTACCGGCNRG | GGgCGAGcTCGtC-ggCGGCGAG | - | 5 | 1 | 0 | 2.75 | 1.38 |
| Chr8:38663694-38663716 | 38663709 | 5 | RNA | GGCCGAGGTCGACTACCGGCNRG | cGCCGtGGTCGcCg--CGGCAGG | + | 4 | 2 | 0 | 2.75 | 1.38 |
| Chr5:143935928-143935950 | 143935935 | 5 | RNA | GGCCGAGGTCGACTACCGGCNRG | GGC--AGGcCaACcgCCGGCCAG | - | 4 | 2 | 0 | 2.75 | 1.38 |
| Chr6:124161874-124161896 | 124161892 | 2 | DNA | GG-CCGAGGTCGACTACCGGCNRG | GGTtCGAGGgCGACgtCCtGCAGG | + | 5 | 1 | 0 | 2.75 | 1.38 |
| Chr3:209532291-209532313 | 209532309 | 2 | X | GGCCGAGGTCGACTACCGGCNRG | GGCCGAGcTCacCTcCCtGCTAG | + | 5 | 0 | 0 | 2.75 | 1.38 |
| Chr9:154838347-154838369 | 154838353 | 4 | RNA | GGCCGAGGTCGACTACCGGCNRG | GGCCGAGGctGgCg--CGGgTGG | - | 5 | 2 | 0 | 2.75 | 1.38 |
| Chr4:243949172-243949194 | 243949179 | 5 | RNA | GGCCGAGGTCGACTACCGGCNRG | GGCCG-GGcCatCcACtGGCGGG | - | 5 | 1 | 0 | 2.75 | 1.38 |
| Chr7:70311146-70311168 | 70311164 | 2 | DNA | GGCCGAGGTCGACTA-CCGGCNRG | GGCCGgGGTaatCaATCCGGCGAG | + | 5 | 1 | 0 | 2.75 | 1.38 |
| Chr2:2866583-2866605 | 2866587 | 2 | RNA | GGCCGAGGTCGACTACCGGCNRG | GGCCGgaGgtGACTtC-GGCGAG | - | 5 | 1 | 0 | 2.75 | 1.38 |
| Chr1:179744933-179744955 | 179744938 | 3 | DNA | GGCCGAGGTCGAC--TACCGGCNRG | cGgCGccGTCGACGGTACCGcCCGG | - | 5 | 2 | 0 | 2.75 | 1.38 |
| Chr7:9428967-9428989 | 9428985 | 2 | DNA | GGCCGAGGTCGACTACCGG--CNRG | GGCtGAGGgCGAtTgCCtGCTCCAG | + | 5 | 2 | 0 | 2.75 | 1.38 |
| Chr1:286042775-286042797 | 286042791 | 4 | DNA | G--GCCGAGGTCGACTACCGGCNRG | GCCGCCGcGcTCGACTgtCGGtTGG | + | 5 | 2 | 0 | 2.75 | 1.38 |
| Chr5:206571495-206571517 | 206571501 | 4 | RNA | GGCCGAGGTCGACTACCGGCNRG | GGCCGAGtaCG--TtgCcGCAGG | - | 5 | 2 | 0 | 2.75 | 1.38 |
| Chr5:108783657-108783679 | 108783673 | 4 | RNA | GGCCGAGGTCGACTACCGGCNRG | GGtCaAttTCGACTtC--GCTGG | + | 5 | 2 | 0 | 2.75 | 1.38 |
| Chr7:133960443-133960465 | 133960461 | 2 | RNA | GGCCGAGGTCGACTACCGGCNRG | GGCCGgGGcgGgC--CgGGCCGG | + | 5 | 2 | 0 | 2.75 | 1.38 |
| Chr7:117839999-117840021 | 117840017 | 2 | DNA | GGCCGAGGTCGACTACCGG-CNRG | GGgaGAGGgCGAtcACCGGCCTAG | + | 5 | 1 | 0 | 2.75 | 1.38 |
| Chr5:182293559-182293581 | 182293577 | 2 | RNA | GGCCGAGGTCGACTACCGGCNRG | G--CGAcGTCGACTACCacgAGG | + | 4 | 2 | 0 | 2.75 | 1.38 |
| Chr4:78527600-78527622 | 78527618 | 2 | RNA | GGCCGAGGTCGACTACCGGCNRG | cGaCGgaGTC--CcACCGGCGAG | + | 5 | 2 | 0 | 2.75 | 1.38 |
| Chr7:111091542-111091564 | 111091547 | 3 | RNA | GGCCGAGGTCGACTACCGGCNRG | aaCCGAGGTCGcCT-CaGGCTGG | - | 4 | 1 | 0 | 2.75 | 1.38 |
| Chr7:88825173-88825195 | 88825179 | 4 | RNA | GGCCGAGGTCGACTACCGGCNRG | GGatGAGGTCGcgTcCC--CTAG | - | 5 | 2 | 0 | 2.75 | 1.38 |
| Chr9:29556821-29556843 | 29556836 | 5 | RNA | GGCCGAGGTCGACTACCGGCNRG | GGCCG-GtaCGAaTcaCGGCAGG | + | 5 | 1 | 0 | 2.75 | 1.38 |
| Chr4:151727579-151727601 | 151727597 | 2 | RNA | GGCCGAGGTCGACTACCGGCNRG | cGaCGAGccCGACT-CCGGtTGG | + | 5 | 1 | 0 | 2.75 | 1.38 |
| Chr4:20359371-20359393 | 20359376 | 3 | RNA | GGCCGAGGTCGACTACCGGCNRG | GGC--gGGcCaACccCCGGCCGG | - | 5 | 2 | 0 | 2.75 | 1.38 |
| Chr5:65989555-65989577 | 65989560 | 3 | RNA | GGCCGAGGTCGACTACCGGCNRG | GtCCG--GTgcACcACCGGaCAG | - | 5 | 2 | 0 | 2.75 | 1.38 |
| Chr8:56771348-56771370 | 56771366 | 2 | RNA | GGCCGAGGTCGACTACCGGCNRG | GGCCatGGcCGAgTtC--GCCAG | + | 5 | 2 | 0 | 2.75 | 1.38 |
| Chr8:151877558-151877580 | 151877576 | 2 | DNA | GGCCGAG--GTCGACTACCGGCNRG | GctCGAGCGGgaGACgACCGGCTGG | + | 5 | 2 | 0 | 2.75 | 1.38 |
| Chr3:29061998-29062020 | 29062016 | 2 | RNA | GGCCGAGGTCGACTACCGGCNRG | GGCgGActTtGACaAC--GCGGG | + | 5 | 2 | 0 | 2.75 | 1.38 |
| Chr1:41943708-41943730 | 41943724 | 4 | RNA | GGCCGAGGTCGACTACCGGCNRG | GGCCaAttTCGcCTtC--GCCGG | + | 5 | 2 | 0 | 2.75 | 1.38 |
| Chr8:138785201-138785223 | 138785219 | 2 | RNA | GGCCGAGGTCGACTACCGGCNRG | GaCaGAGcT-GACaACCGGaGAG | + | 5 | 1 | 0 | 2.75 | 1.38 |
| Chr5:20511163-20511185 | 20511180 | 3 | RNA | GGCCGAGGTCGACTACCGGCNRG | GGtCGAtGagGACcACC--CCAG | + | 5 | 2 | 0 | 2.75 | 1.38 |
| Chr6:129542768-129542790 | 129542783 | 5 | RNA | GGCCGAGGTCGACTACCGGCNRG | GGgCGAGGgCGA-gAatGGCAGG | + | 5 | 1 | 0 | 2.75 | 1.38 |
| Chr2:183741875-183741897 | 183741893 | 2 | RNA | GGCCGAGGTCGACTACCGGCNRG | a--CGgGGTCGACaACgaGCGGG | + | 5 | 2 | 0 | 2.75 | 1.38 |
| Chr6:10901384-10901406 | 10901399 | 5 | RNA | GGCCGAGGTCGACTACCGGCNRG | GGCtGgGcTgGACTA--GGtTGG | + | 5 | 2 | 0 | 2.75 | 1.38 |
| Chr7:53803649-53803671 | 53803664 | 5 | RNA | GGCCGAGGTCGACTACCGGCNRG | GGC--ttGcgGAtTACCGGCTGG | + | 5 | 2 | 0 | 2.75 | 1.38 |
| Chr4:127578360-127578382 | 127578377 | 3 | X | GGCCGAGGTCGACTACCGGCNRG | GGaaGAGGTgGcCTAgCGGCTGG | + | 5 | 0 | 0 | 2.75 | 1.38 |
| Chr6:157417855-157417877 | 157417871 | 4 | DNA | G--GCCGAGGTCGACTACCGGCNRG | GCCGCCGtGGTCGcCTtCgcGCTGG | + | 5 | 2 | 0 | 2.75 | 1.38 |
| Chr3:43512456-43512478 | 43512474 | 2 | RNA | GGCCGAGGTCGACTACCGGCNRG | GG--GAaGggGACcAgCGGCTGG | + | 5 | 2 | 0 | 2.75 | 1.38 |
| Chr2:186668118-186668140 | 186668136 | 2 | RNA | GGCCGAGGTCGACTACCGGCNRG | GGC--tcGcgGACTACtGGCTGG | + | 5 | 2 | 0 | 2.75 | 1.38 |
| Chr1:166206945-166206967 | 166206950 | 3 | RNA | GGCCGAGGTCGACTACCGGCNRG | G-aCGgGGTCGACaACgaGCGGG | - | 5 | 1 | 0 | 2.75 | 1.38 |
| Chr1:205588764-205588786 | 205588771 | 5 | RNA | GGCCGAGGTCGACTACCGGCNRG | aGCCGAGGgtGcCT-CgGGCTGG | - | 5 | 1 | 0 | 2.75 | 1.38 |
| Chr3:115593458-115593480 | 115593463 | 3 | RNA | GGCCGAGGTCGACTACCGGCNRG | cGCCG-GGcCGAgctCCGGCGAG | - | 5 | 1 | 0 | 2.75 | 1.38 |
| Chr8:119783066-119783088 | 119783071 | 3 | RNA | GGCCGAGGTCGACTACCGGCNRG | GtC--tccTtGACTACCGGCCGG | - | 5 | 2 | 0 | 2.75 | 1.38 |
| Chr6:170578096-170578118 | 170578112 | 4 | RNA | GGCCGAGGTCGACTACCGGCNRG | G-aCGAGGTCGACaACgaGCGGG | + | 4 | 1 | 0 | 2.75 | 1.38 |
| Chr1:56665462-56665484 | 56665477 | 5 | RNA | GGCCGAGGTCGACTACCGGCNRG | GGgC-AGGcCGAgcACCaGCCGG | + | 5 | 1 | 0 | 2.75 | 1.38 |
| Chr6:5030952-5030974 | 5030970 | 2 | RNA | GGCCGAGGTCGACTACCGGCNRG | GaCCGAGGaCaAgg--CGGCAAG | + | 5 | 2 | 0 | 2.75 | 1.38 |
| Chr8:133099564-133099586 | 133099581 | 3 | DNA | GGCCGAGGTCGACTACC--GGCNRG | GGCgGAGaTCtcCTcCCGAGGCCGG | + | 5 | 2 | 0 | 2.75 | 1.38 |
| Chr5:24782159-24782181 | 24782174 | 5 | RNA | GGCCGAGGTCGACTACCGGCNRG | GGC--tcGcgGACTACCGtCTGG | + | 5 | 2 | 0 | 2.75 | 1.38 |
| Chr2:152935761-152935783 | 152935766 | 3 | RNA | GGCCGAGGTCGACTACCGGCNRG | aGC--AGcgCGACTtCCGGgAGG | - | 5 | 2 | 0 | 2.75 | 1.38 |
| Chr4:13285912-13285934 | 13285917 | 3 | RNA | GGCCGAGGTCGACTACCGGCNRG | G-aCGgGGTCGACaACgaGCGGG | - | 5 | 1 | 0 | 2.75 | 1.38 |
| Chr7:171631366-171631388 | 171631381 | 5 | RNA | GGCCGAGGTCGACTACCGGCNRG | GcgaGAGGTCGAt--CCGGaTAG | + | 5 | 2 | 0 | 2.75 | 1.38 |
| Chr5:129045322-129045344 | 129045327 | 3 | RNA | GGCCGAGGTCGACTACCGGCNRG | GGgCGcGGTCGAggACaG-CGGG | - | 5 | 1 | 0 | 2.75 | 1.38 |
| Chr8:99323920-99323942 | 99323936 | 4 | RNA | GGCCGAGGTCGACTACCGGCNRG | aaCCGAGGaCGgCT-CgGGCTGG | + | 5 | 1 | 0 | 2.75 | 1.38 |
| Chr2:44189994-44190016 | 44190000 | 4 | RNA | GGCCGAGGTCGACTACCGGCNRG | GGgCGgGGTtGgCT-CCGGgAGG | - | 5 | 1 | 0 | 2.75 | 1.38 |
| Chr8:101358178-101358200 | 101358195 | 3 | RNA | GGCCGAGGTCGACTACCGGCNRG | GcCtGAGtcCGAC--CCGtCAGG | + | 5 | 2 | 0 | 2.75 | 1.38 |
| Chr6:44061726-44061748 | 44061743 | 3 | RNA | GGCCGAGGTCGACTACCGGCNRG | GGC-GAGGTCaACcACgGcgTAG | + | 5 | 1 | 0 | 2.75 | 1.38 |
| Chr8:140926581-140926603 | 140926598 | 3 | RNA | GGCCGAGGTCGACTACCGGCNRG | GGCaGAGGaC--CTAgaGGgGGG | + | 5 | 2 | 0 | 2.75 | 1.38 |
| Chr1:35686356-35686378 | 35686372 | 4 | RNA | GGCCGAGGTCGACTACCGGCNRG | GGaCGAGG-CGcCcACCGcCCGG | + | 4 | 1 | 0 | 2.75 | 1.38 |
| Chr1:251452902-251452924 | 251452919 | 3 | DNA | GGCCGAGGTCG-ACTACCGGCNRG | GGCCGAacaCGAACcgCCGGCTGG | + | 5 | 1 | 0 | 2.75 | 1.38 |
| Chr5:153755342-153755364 | 153755360 | 2 | RNA | GGCCGAGGTCGACTACCGGCNRG | GGCgGAGGTCG--TtCgGcaGAG | + | 5 | 2 | 0 | 2.75 | 1.38 |
| Chr4:71622045-71622067 | 71622051 | 4 | RNA | GGCCGAGGTCGACTACCGGCNRG | GaCCGAGG-CGACgACaaGaAGG | - | 5 | 1 | 0 | 2.75 | 1.38 |
| Chr5:14166142-14166164 | 14166160 | 2 | RNA | GGCCGAGGTCGACTACCGGCNRG | GG--GAGGTCGgCgcCaGGaTAG | + | 5 | 2 | 0 | 2.75 | 1.38 |
| Chr10:129625891-129625913 | 129625897 | 4 | RNA | GGCCGAGGTCGACTACCGGCNRG | GGaCGAcccCGACT-CCGGtTGG | - | 5 | 1 | 0 | 2.75 | 1.38 |
| Chr7:26792873-26792895 | 26792889 | 4 | RNA | GGCCGAGGTCGACTACCGGCNRG | GGC--AGGgCGAgcACCaGaAGG | + | 5 | 2 | 0 | 2.75 | 1.38 |
| Chr1:138278932-138278954 | 138278947 | 5 | RNA | GGCCGAGGTCGACTACCGGCNRG | cGCCGccGTCGgCcACCG-CCGG | + | 5 | 1 | 0 | 2.75 | 1.38 |
| Chr2:22260813-22260835 | 22260828 | 5 | RNA | GGCCGAGGTCGACTACCGGCNRG | GGC--tcGcgGACTACCGGCTGG | + | 4 | 2 | 0 | 2.75 | 1.38 |
| Chr2:182782102-182782124 | 182782117 | 5 | RNA | GGCCGAGGTCGACTACCGGCNRG | GcCgGAGGcCGACTt--GGaGGG | + | 5 | 2 | 0 | 2.75 | 1.38 |
| Chr2:149090399-149090421 | 149090417 | 2 | RNA | GGCCGAGGTCGACTACCGGCNRG | GGC--tcGcgGACTACCGaCTGG | + | 5 | 2 | 0 | 2.75 | 1.38 |
| Chr3:810172-810194 | 810177 | 3 | RNA | GGCCGAGGTCGACTACCGGCNRG | cGtCGA--TCaACTcCaGGCCAG | - | 5 | 2 | 0 | 2.75 | 1.38 |
| Chr1:238778460-238778482 | 238778466 | 4 | RNA | GGCCGAGGTCGACTACCGGCNRG | cGaCGAGtTCtAtTACC--CGAG | - | 5 | 2 | 0 | 2.75 | 1.38 |
| Chr5:174122720-174122742 | 174122737 | 3 | RNA | GGCCGAGGTCGACTACCGGCNRG | GGCgGAGGTCG--TtCgGcaGAG | + | 5 | 2 | 0 | 2.75 | 1.38 |
| Chr6:45449881-45449903 | 45449888 | 5 | RNA | GGCCGAGGTCGACTACCGGCNRG | GGC-GAaaTCGACcgCCGGtGAG | - | 5 | 1 | 0 | 2.75 | 1.38 |
| Chr8:11223615-11223637 | 11223622 | 5 | RNA | GGCCGAGGTCGACTACCGGCNRG | tGCCGAGGatGcCT-CgGGCTGG | - | 5 | 1 | 0 | 2.75 | 1.38 |
| Chr2:226206648-226206670 | 226206655 | 5 | RNA | GGCCGAGGTCGACTACCGGCNRG | GtgCGgGGTtGgCT--CGGCCGG | - | 5 | 2 | 0 | 2.75 | 1.38 |
| Chr1:12003580-12003602 | 12003595 | 5 | RNA | GGCCGAGGTCGACTACCGGCNRG | GGtCG--GTCtACTcCtGGgAAG | + | 5 | 2 | 0 | 2.75 | 1.38 |
| Chr9:124681722-124681744 | 124681727 | 3 | RNA | GGCCGAGGTCGACTACCGGCNRG | GGCtcAGGTaGAagA--GGCAAG | - | 5 | 2 | 0 | 2.75 | 1.38 |
| Chr4:18429693-18429715 | 18429697 | 2 | RNA | GGCCGAGGTCGACTACCGGCNRG | G-CaGAGGTCaAaTACaGGaCAG | - | 5 | 1 | 0 | 2.75 | 1.38 |
| Chr1:254308288-254308310 | 254308303 | 5 | RNA | GGCCGAGGTCGACTACCGGCNRG | GGCCcAtGaCG--TttCGGCCGG | + | 5 | 2 | 0 | 2.75 | 1.38 |
| Chr6:171867766-171867788 | 171867781 | 5 | RNA | GGCCGAGGTCGACTACCGGCNRG | GGaC-AGGTCGAggAaCGcCAGG | + | 5 | 1 | 0 | 2.75 | 1.38 |
| Chr2:51291025-51291047 | 51291030 | 3 | RNA | GGCCGAGGTCGACTACCGGCNRG | G-aCGgGGTCGACgACgaGCGGG | - | 5 | 1 | 0 | 2.75 | 1.38 |
| Chr1:173336304-173336326 | 173336322 | 2 | RNA | GGCCGAGGTCGACTACCGGCNRG | GGCgGAGGcCGAga--CGGtGAG | + | 5 | 2 | 0 | 2.75 | 1.38 |
| Chr8:169947930-169947952 | 169947946 | 4 | RNA | GGCCGAGGTCGACTACCGGCNRG | GGCCt-GGTgGgCgAgCGGCAGG | + | 5 | 1 | 0 | 2.75 | 1.38 |
| Chr1:234197394-234197416 | 234197399 | 3 | RNA | GGCCGAGGTCGACTACCGGCNRG | G-aCGgGGTCGACaACgaGCGGG | - | 5 | 1 | 0 | 2.75 | 1.38 |
| Chr4:164613100-164613122 | 164613106 | 4 | RNA | GGCCGAGGTCGACTACCGGCNRG | GtCCtcGG--GAtaACCGGCAGG | - | 5 | 2 | 0 | 2.75 | 1.38 |
| Chr2:64910493-64910515 | 64910508 | 5 | RNA | GGCCGAGGTCGACTACCGGCNRG | GGCCG-GGTCGtCcACtGGCGGG | + | 3 | 1 | 0 | 2.75 | 1.38 |
| Chr10:114264179-114264201 | 114264194 | 5 | RNA | GGCCGAGGTCGACTACCGGCNRG | GGC--tcGcgGACTACCGGCTGG | + | 4 | 2 | 0 | 2.75 | 1.38 |
| Chr10:65497996-65498018 | 65498003 | 5 | RNA | GGCCGAGGTCGACTACCGGCNRG | GtCCG--GTgcACcACCGGaCAG | - | 5 | 2 | 0 | 2.75 | 1.38 |
| Chr3:80245059-80245081 | 80245064 | 3 | RNA | GGCCGAGGTCGACTACCGGCNRG | ttCCGAGGTC-ACTAaaGtCAGG | - | 5 | 1 | 0 | 2.75 | 1.38 |
| Chr4:142823194-142823216 | 142823212 | 2 | RNA | GGCCGAGGTCGACTACCGGCNRG | cG--GAGcTCGAgcACgGGCGGG | + | 5 | 2 | 0 | 2.75 | 1.38 |
| Chr1:182443334-182443356 | 182443339 | 3 | RNA | GGCCGAGGTCGACTACCGGCNRG | G--CGAGGgCGAgaACgGGaGAG | - | 5 | 2 | 0 | 2.75 | 1.38 |
| Chr7:57779610-57779632 | 57779628 | 2 | RNA | GGCCGAGGTCGACTACCGGCNRG | GGgCcgGGcCGgCTA--GGCTGG | + | 5 | 2 | 0 | 2.75 | 1.38 |
| Chr5:130328571-130328593 | 130328586 | 5 | RNA | GGCCGAGGTCGACTACCGGCNRG | GGCCGAGG--GAtggCCcaCGAG | + | 5 | 2 | 0 | 2.75 | 1.38 |
| Chr1:99262878-99262900 | 99262884 | 4 | RNA | GGCCGAGGTCGACTACCGGCNRG | GcCaGtGGaCGAC--CCGGCCAG | - | 4 | 2 | 0 | 2.75 | 1.38 |
| Chr1:22343569-22343591 | 22343587 | 2 | DNA | GGC-CGAGGTCGACTACCGGCNRG | GcCTCGgGGaCGtCTtCCGGCTGG | + | 5 | 1 | 0 | 2.75 | 1.38 |
| Chr2:124769964-124769986 | 124769982 | 2 | DNA | GGCCGAGGTCGACTA-CCGGCNRG | GGgCGAGacgGcCTAACCGGCCGG | + | 5 | 1 | 0 | 2.75 | 1.38 |
| Chr9:107832983-107833005 | 107832999 | 4 | RNA | GGCCGAGGTCGACTACCGGCNRG | G-CaGgGGgCGAggACCGGCTGG | + | 5 | 1 | 0 | 2.75 | 1.38 |
| Chr2:41740076-41740098 | 41740081 | 3 | RNA | GGCCGAGGTCGACTACCGGCNRG | GGC--ttGcgGACTACCGGCTGG | - | 4 | 2 | 0 | 2.75 | 1.38 |
| Chr2:177862013-177862035 | 177862030 | 3 | RNA | GGCCGAGGTCGACTACCGGCNRG | GcCCtAGGTgG--TggCGGCTGG | + | 5 | 2 | 0 | 2.75 | 1.38 |
| Chr1:240236903-240236925 | 240236919 | 4 | RNA | GGCCGAGGTCGACTACCGGCNRG | GGCCGcGGcCGACat--GtCCAG | + | 5 | 2 | 0 | 2.75 | 1.38 |
| Chr10:87827845-87827867 | 87827860 | 5 | RNA | GGCCGAGGTCGACTACCGGCNRG | GGC--tcGcaGACTACCGGCTGG | + | 4 | 2 | 0 | 2.75 | 1.38 |
| Chr8:72382689-72382711 | 72382704 | 5 | RNA | GGCCGAGGTCGACTACCGGCNRG | G-CCtcGGgCGAaTACtGGCAGG | + | 5 | 1 | 0 | 2.75 | 1.38 |
| Chr1:218293233-218293255 | 218293251 | 2 | RNA | GGCCGAGGTCGACTACCGGCNRG | GGgCGAGcTtGggTA--GGCAGG | + | 5 | 2 | 0 | 2.75 | 1.38 |
| Chr3:196423753-196423775 | 196423758 | 3 | RNA | GGCCGAGGTCGACTACCGGCNRG | G-aCGgGGTCGACgACgaGCGGG | - | 5 | 1 | 0 | 2.75 | 1.38 |
| Chr3:141716132-141716154 | 141716148 | 4 | DNA | GGCCGAGGTCGA--CTACCGGCNRG | GGCCGAGGcCGAGGCTtggGGtCGG | + | 5 | 2 | 0 | 2.75 | 1.38 |
| Chr3:205618639-205618661 | 205618657 | 2 | RNA | GGCCGAGGTCGACTACCGGCNRG | GG--GcGGTCGtggACCGGCGAG | + | 4 | 2 | 0 | 2.75 | 1.38 |
| Chr7:153120997-153121019 | 153121002 | 3 | RNA | GGCCGAGGTCGACTACCGGCNRG | GtCttgGGT-GACcACCGGCGGG | - | 5 | 1 | 0 | 2.75 | 1.38 |
| Chr9:70838213-70838235 | 70838218 | 3 | RNA | GGCCGAGGTCGACTACCGGCNRG | GGCCGAGGTCGAggAC-GtCAGG | - | 3 | 1 | 0 | 2.75 | 1.38 |
| Chr4:111121788-111121810 | 111121793 | 3 | RNA | GGCCGAGGTCGACTACCGGCNRG | GG--GAaGTgGgCTgCCGGgGAG | - | 5 | 2 | 0 | 2.75 | 1.38 |
| Chr9:80534347-80534369 | 80534353 | 4 | RNA | GGCCGAGGTCGACTACCGGCNRG | GGC--AGGcCGAgctCCGGtCGG | - | 5 | 2 | 0 | 2.75 | 1.38 |
| Chr4:1224057-1224079 | 1224075 | 2 | RNA | GGCCGAGGTCGACTACCGGCNRG | GGCCGAGtaCG--TtgCcGCAGG | + | 5 | 2 | 0 | 2.75 | 1.38 |
| Chr5:71490334-71490356 | 71490340 | 4 | RNA | GGCCGAGGTCGACTACCGGCNRG | GtCCG--GTgcACcACCGGaTAG | - | 5 | 2 | 0 | 2.75 | 1.38 |
| Chr7:142493491-142493513 | 142493496 | 3 | DNA | GGCCGAGGTCGACT-ACCGGCNRG | aaCCtAGGTCcgCTCACCGGCAGG | - | 5 | 1 | 0 | 2.75 | 1.38 |
| Chr2:139404628-139404650 | 139404644 | 4 | RNA | GGCCGAGGTCGACTACCGGCNRG | GaCCG-GaTCGACaACgaGCGGG | + | 5 | 1 | 0 | 2.75 | 1.38 |

| | | | | | | | | | | | |
|---|---|---|---|---|---|---|---|---|---|---|---|
| Chr10:75795316-75795338 | 75795333 | 3 | RNA | GGCCGAGGTCGACTACCGGCNRG | GcCgGAGccCGAC--CCGtCAAG | + | 5 | 2 | 0 | 2.75 | 1.38 |
| Chr5:133413000-133413022 | 133413006 | 4 | RNA | GGCCGAGGTCGACTACCGGCNRG | GGtgGAGGgCaACTAtC--CCGG | - | 5 | 2 | 0 | 2.75 | 1.38 |
| Chr4:243565738-243565760 | 243565742 | 2 | RNA | GGCCGAGGTCGACTACCGGCNRG | GGCCGgaGgtGACTtC-GGCGAG | - | 5 | 1 | 0 | 2.75 | 1.38 |
| Chr8:38783048-38783070 | 38783064 | 4 | RNA | GGCCGAGGTCGACTACCGGCNRG | GtgCGAtG-CGgCTgCCGGCCGG | + | 5 | 1 | 0 | 2.75 | 1.38 |
| Chr9:133812211-133812233 | 133812227 | 4 | RNA | GGCCGAGGTCGACTACCGGCNRG | G-aCGgGGTCGACaACgaGCGGG | + | 5 | 1 | 0 | 2.75 | 1.38 |
| Chr2:16595188-16595210 | 16595203 | 5 | RNA | GGCCGAGGTCGACTACCGGCNRG | GGCtcAcG--GACTAttGGCTGG | + | 5 | 2 | 0 | 2.75 | 1.38 |
| Chr7:64593047-64593069 | 64593064 | 3 | RNA | GGCCGAGGTCGACTACCGGCNRG | GcCCaAGccCGACTtC--GCCAG | + | 5 | 2 | 0 | 2.75 | 1.38 |
| Chr2:50610977-50610999 | 50610995 | 2 | RNA | GGCCGAGGTCGACTACCGGCNRG | GGtCGAGGTCG--TggCcGCGGG | + | 4 | 2 | 0 | 2.75 | 1.38 |
| Chr5:171048843-171048865 | 171048848 | 3 | RNA | GGCCGAGGTCGACTACCGGCNRG | GGCtcAcG--GACTACaGtCTGG | - | 5 | 2 | 0 | 2.75 | 1.38 |
| Chr3:129227775-129227797 | 129227782 | 5 | RNA | GGCCGAGGTCGACTACCGGCNRG | GcaCGAG--CGAaTACaGGaTAG | - | 5 | 2 | 0 | 2.75 | 1.38 |
| Chr1:245550493-245550515 | 245550511 | 2 | RNA | GGCCGAGGTCGACTACCGGCNRG | GGC--AGGcCaAgctCCGGCCGG | + | 5 | 2 | 0 | 2.75 | 1.38 |
| Chr2:45460337-45460359 | 45460355 | 2 | RNA | GGCCGAGGTCGACTACCGGCNRG | GGgCGAGGaC--CgAgaGGCGAG | + | 5 | 2 | 0 | 2.75 | 1.38 |
| Chr5:222727445-222727467 | 222727450 | 3 | RNA | GGCCGAGGTCGACTACCGGCNRG | GGCaGcGGTCGAC-ggCGGgCGG | - | 5 | 1 | 0 | 2.75 | 1.38 |
| Chr4:188318694-188318716 | 188318699 | 3 | RNA | GGCCGAGGTCGACTACCGGCNRG | G-aCGgGGTCGACaACgaGCGGG | - | 5 | 1 | 0 | 2.75 | 1.38 |
| Chr3:201853421-201853443 | 201853427 | 4 | RNA | GGCCGAGGTCGACTACCGGCNRG | aGCCctGcTCGACT--CcGCTGG | - | 5 | 2 | 0 | 2.75 | 1.38 |
| Chr8:78781894-78781916 | 78781900 | 4 | X | GGCCGAGGTCGACTACCGGCNRG | GGaaGAGGTgGcCTAgCGGCTGG | - | 5 | 0 | 1 | 1.65 | 1.33 |
| Chr5:28634198-28634220 | 28634205 | 5 | RNA | GGCCGAGGTCGACTACCGGCNRG | GGCtGcGcTaGcCT-CCGGCCAG | - | 5 | 1 | 1 | 1.65 | 1.33 |
| Chr3:198373903-198373925 | 198373919 | 4 | RNA | GGCCGAGGTCGACTACCGGCNRG | G-aCGgGGaCGgCgACCGGCGGG | + | 5 | 1 | 1 | 1.65 | 1.33 |
| Chr3:211808730-211808752 | 211808748 | 2 | RNA | GGCCGAGGTCGACTACCGGCNRG | tGgCGAGcTCa--TACCGGCCGG | + | 4 | 2 | 1 | 1.65 | 1.33 |
| Chr1:122460244-122460266 | 122460248 | 2 | RNA | GGCCGAGGTCGACTACCGGCNRG | GaCCaAGGaatACT-CCGGCGAG | - | 5 | 1 | 2 | 0.55 | 1.28 |
| Chr6:20347024-20347046 | 20347041 | 3 | DNA | GGC-CGAGGTCGACTACCGGCNRG | GcCTCGgGGaCGtCTtCCGGCTGG | + | 5 | 1 | 2 | 0.55 | 1.28 |
| Chr5:11477335-11477357 | 11477341 | 4 | RNA | GGCCGAGGTCGACTACCGGCNRG | G-aCGgGGTCGACgACgaGCGGG | - | 5 | 1 | 2 | 0.55 | 1.28 |
| Chr1:91102570-91102592 | 91102576 | 4 | RNA | GGCCGAGGTCGACTACCGGCNRG | GGCCGgGccgGAC--CgGGCCGG | - | 5 | 2 | 0 | 2.20 | 1.10 |
| Chr4:40308772-40308794 | 40308778 | 4 | RNA | GGCCGAGGTCGACTACCGGCNRG | GGgCGAaGatGACT--CGGgCGG | - | 5 | 2 | 0 | 2.20 | 1.10 |
| Chr1:141832898-141832920 | 141832914 | 4 | RNA | GGCCGAGGTCGACTACCGGCNRG | GtCCGgaGTt--CTtCCGGCTGG | + | 5 | 2 | 0 | 2.20 | 1.10 |
| Chr10:25201964-25201986 | 25201969 | 3 | RNA | GGCCGAGGTCGACTACCGGCNRG | G-aCGgGGTCGACaACgaGCGGG | - | 5 | 1 | 0 | 2.20 | 1.10 |
| Chr7:70569246-70569268 | 70569251 | 3 | DNA | GGC--CGAGGTCGACTACCGGCNRG | GGCCTCGgGGgCtACacCCGGCGGG | - | 5 | 2 | 0 | 2.20 | 1.10 |
| Chr5:216940342-216940364 | 216940347 | 3 | RNA | GGCCGAGGTCGACTACCGGCNRG | GGgCG-GGcCaACctCCGGCCGG | - | 5 | 1 | 0 | 2.20 | 1.10 |
| Chr5:84347854-84347876 | 84347859 | 3 | X | GGCCGAGGTCGACTACCGGCNRG | GGaCGAGcTCGgCgAgCGGCAGG | - | 5 | 0 | 0 | 2.20 | 1.10 |
| Chr1:115548034-115548056 | 115548050 | 4 | RNA | GGCCGAGGTCGACTACCGGCNRG | GGCgGAGGg-GctTACCGGCGGG | + | 4 | 1 | 0 | 2.20 | 1.10 |
| Chr7:48721400-48721422 | 48721416 | 4 | RNA | GGCCGAGGTCGACTACCGGCNRG | cG--GAGGgaGAaTACCcGCTGG | + | 5 | 2 | 0 | 2.20 | 1.10 |
| Chr8:23077540-23077562 | 23077547 | 5 | RNA | GGCCGAGGTCGACTACCGGCNRG | GGaCGAtcTCG--TAgaGGCGAG | - | 5 | 2 | 0 | 2.20 | 1.10 |
| Chr7:114036794-114036816 | 114036811 | 3 | DNA | GGC-CGAGGTCGACTACCGGCNRG | GcCTCGAGGaCGtCTtCCGGCTGG | + | 4 | 1 | 0 | 2.20 | 1.10 |
| Chr3:20538837-20538859 | 20538841 | 2 | RNA | GGCCGAGGTCGACTACCGGCNRG | GtCCG--GTgcACcACCGGaCAG | - | 5 | 2 | 0 | 2.20 | 1.10 |
| Chr4:202411281-202411303 | 202411298 | 3 | RNA | GGCCGAGGTCGACTACCGGCNRG | GtCCG--GTgcACcACCGGaCAG | + | 5 | 2 | 0 | 2.20 | 1.10 |
| Chr8:80758363-80758385 | 80758381 | 2 | RNA | GGCCGAGGTCGACTACCGGCNRG | GcC--gGGTCGtCcACCGGCGGG | + | 4 | 2 | 0 | 2.20 | 1.10 |
| Chr5:203358148-203358170 | 203358154 | 4 | RNA | GGCCGAGGTCGACTACCGGCNRG | GGaCcAGGTCG--TcCCaGtTGG | - | 5 | 2 | 0 | 2.20 | 1.10 |
| Chr7:103576040-103576062 | 103576046 | 4 | RNA | GGCCGAGGTCGACTACCGGCNRG | aGCtGAGGTCttCT--CGGCCAG | - | 4 | 2 | 0 | 2.20 | 1.10 |
| Chr6:139959918-139959940 | 139959933 | 5 | RNA | GGCCGAGGTCGACTACCGGCNRG | GGC--tcGcgGACTACCGGtTGG | + | 5 | 2 | 0 | 2.20 | 1.10 |
| Chr7:62338175-62338197 | 62338190 | 5 | RNA | GGCCGAGGTCGACTACCGGCNRG | GcCCGAGaTCGACctCa--CCGG | + | 5 | 2 | 0 | 2.20 | 1.10 |
| Chr1:121232818-121232840 | 121232825 | 5 | RNA | GGCCGAGGTCGACTACCGGCNRG | GGCtGAG--CGcaTggCGGCGGG | - | 5 | 2 | 0 | 2.20 | 1.10 |
| Chr5:153362641-153362663 | 153362659 | 2 | RNA | GGCCGAGGTCGACTACCGGCNRG | GtCCGAGGa--ACaACgGcCGAG | + | 5 | 2 | 0 | 2.20 | 1.10 |
| Chr2:105895567-105895589 | 105895583 | 4 | RNA | GGCCGAGGTCGACTACCGGCNRG | cGgCGAGGa--ACcACCGtCGGG | + | 5 | 2 | 0 | 2.20 | 1.10 |
| Chr3:181915416-181915438 | 181915433 | 3 | RNA | GGCCGAGGTCGACTACCGGCNRG | GGC--tcGcgGACTACCGGCTGG | + | 4 | 2 | 0 | 2.20 | 1.10 |
| Chr6:160902460-160902482 | 160902465 | 3 | RNA | GGCCGAGGTCGACTACCGGCNRG | G-CCGAGGcCGcggAgCGGCGGG | - | 5 | 1 | 0 | 2.20 | 1.10 |
| Chr2:82786509-82786531 | 82786513 | 2 | RNA | GGCCGAGGTCGACTACCGGCNRG | GaCCGAGG-CGACgACaaGaAGG | - | 5 | 1 | 0 | 2.20 | 1.10 |
| Chr10:60737498-60737520 | 60737515 | 3 | RNA | GGCCGAGGTCGACTACCGGCNRG | caCCaAGG--GAtTACCGtCAAG | + | 5 | 2 | 0 | 2.20 | 1.10 |
| Chr4:83929226-83929248 | 83929231 | 3 | RNA | GGCCGAGGTCGACTACCGGCNRG | tGCCGAGGTCGgCcgC--GCCGG | - | 4 | 2 | 0 | 2.20 | 1.10 |
| Chr1:15964999-15965021 | 15965005 | 4 | RNA | GGCCGAGGTCGACTACCGGCNRG | GGCaGAGG-CGcggACCGaCAAG | - | 5 | 1 | 0 | 2.20 | 1.10 |
| Chr4:45643856-45643878 | 45643871 | 5 | RNA | GGCCGAGGTCGACTACCGGCNRG | GcgCGAGGcCGA--ACgGGCCGG | + | 4 | 2 | 0 | 2.20 | 1.10 |
| Chr3:23439613-23439635 | 23439617 | 2 | RNA | GGCCGAGGTCGACTACCGGCNRG | GtCCG--GTgcACcACCGGaTAG | - | 5 | 2 | 0 | 2.20 | 1.10 |
| Chr2:146215252-146215274 | 146215258 | 4 | RNA | GGCCGAGGTCGACTACCGGCNRG | GaCCGActcCGACcACCG-CCAG | - | 5 | 1 | 0 | 2.20 | 1.10 |
| Chr2:151785958-151785980 | 151785973 | 5 | RNA | GGCCGAGGTCGACTACCGGCNRG | G-CCGgGGTCGgCTcCgGGtCAG | + | 5 | 1 | 0 | 2.20 | 1.10 |
| Chr2:53620626-53620648 | 53620632 | 4 | DNA | GGCCGA-GGTCGACTACCGGCNRG | GGCgGACGGTCcgCgACCGaCCAG | - | 5 | 1 | 0 | 2.20 | 1.10 |
| Chr2:101555183-101555205 | 101555187 | 2 | DNA | GGCCGAGGTCGACTACC--GGCNRG | GGCCGAGcTCGcCgAgCTGGGgCGG | - | 5 | 2 | 0 | 2.20 | 1.10 |
| Chr2:162836192-162836214 | 162836198 | 4 | RNA | GGCCGAGGTCGACTACCGGCNRG | GGC--AGGcCaACctCCGGCCGG | - | 4 | 2 | 0 | 2.20 | 1.10 |
| Chr1:96702091-96702113 | 96702106 | 5 | DNA | GGC--CGAGGTCGACTACCGGCNRG | cGCACgGAGGTCGgCTACaGcCAGG | + | 5 | 2 | 0 | 2.20 | 1.10 |
| Chr3:206541231-206541253 | 206541237 | 4 | RNA | GGCCGAGGTCGACTACCGGCNRG | GGC--tcGcgGAtTACCGGCTGG | - | 5 | 2 | 0 | 2.20 | 1.10 |
| Chr6:17339644-17339666 | 17339648 | 2 | DNA | GGCCGAGGTCGAC--TACCGGCNRG | tGCCGAcGTCGACCGTcCgGGgGAG | - | 5 | 2 | 0 | 2.20 | 1.10 |
| Chr1:116457339-116457361 | 116457345 | 4 | RNA | GGCCGAGGTCGACTACCGGCNRG | GGCaGcGaTaGcCT--CGGCCGG | - | 5 | 2 | 0 | 2.20 | 1.10 |
| Chr10:110164474-110164496 | 110164481 | 5 | RNA | GGCCGAGGTCGACTACCGGCNRG | GGCCGAGGgCGAggACC--aTGG | - | 4 | 2 | 0 | 2.20 | 1.10 |
| Chr5:98942464-98942486 | 98942471 | 5 | RNA | GGCCGAGGTCGACTACCGGCNRG | GcCCGAGGaCGctT--CaGCAGG | - | 5 | 2 | 0 | 2.20 | 1.10 |
| Chr8:64697155-64697177 | 64697162 | 5 | RNA | GGCCGAGGTCGACTACCGGCNRG | GGgCGgGGTtGgCT-CCGGgAGG | - | 5 | 1 | 0 | 2.20 | 1.10 |
| Chr9:70823798-70823820 | 70823814 | 4 | RNA | GGCCGAGGTCGACTACCGGCNRG | GGCatAtG-CGgCaACCGGCAAG | + | 5 | 1 | 0 | 2.20 | 1.10 |
| Chr6:42712995-42713017 | 42712999 | 2 | RNA | GGCCGAGGTCGACTACCGGCNRG | GGCtGAGGaaccCTA--GGCCAG | - | 5 | 2 | 0 | 2.20 | 1.10 |
| Chr4:168633378-168633400 | 168633394 | 4 | RNA | GGCCGAGGTCGACTACCGGCNRG | GcCgGAGGT-GACgACtGGtGGG | + | 5 | 1 | 0 | 2.20 | 1.10 |
| Chr2:166306364-166306386 | 166306381 | 3 | RNA | GGCCGAGGTCGACTACCGGCNRG | GGgCGcGGTCGACTgC-GagGAG | + | 5 | 1 | 0 | 2.20 | 1.10 |
| Chr4:36465107-36465129 | 36465123 | 4 | RNA | GGCCGAGGTCGACTACCGGCNRG | GGCCGccGT-GAgTACaGGtGAG | + | 5 | 1 | 0 | 2.20 | 1.10 |
| Chr3:190137786-190137808 | 190137804 | 2 | RNA | GGCCGAGGTCGACTACCGGCNRG | GGCCG-GGcCatCcACtGGCGGG | + | 5 | 1 | 0 | 2.20 | 1.10 |
| Chr8:173555957-173555979 | 173555963 | 4 | RNA | GGCCGAGGTCGACTACCGGCNRG | aGCCGAGGatGtCT--CGGgCAG | - | 5 | 2 | 0 | 2.20 | 1.10 |
| Chr8:40897227-40897249 | 40897243 | 4 | RNA | GGCCGAGGTCGACTACCGGCNRG | cGCCGActTCG--TAgCGGtGGG | + | 5 | 2 | 0 | 2.20 | 1.10 |
| Chr9:68573271-68573293 | 68573288 | 3 | RNA | GGCCGAGGTCGACTACCGGCNRG | GGaCcAGGTCG--TcCCaGtTGG | + | 5 | 2 | 0 | 2.20 | 1.10 |
| Chr2:3354111-3354133 | 3354127 | 4 | RNA | GGCCGAGGTCGACTACCGGCNRG | cGCCGAGGggGAagAC--GCTGG | + | 5 | 2 | 0 | 2.20 | 1.10 |
| Chr3:182620682-182620704 | 182620699 | 3 | RNA | GGCCGAGGTCGACTACCGGCNRG | GGC--tcGcgGACTACCGGCTGG | + | 4 | 2 | 0 | 2.20 | 1.10 |
| Chr1:13638939-13638961 | 13638945 | 4 | RNA | GGCCGAGGTCGACTACCGGCNRG | GGCCttGcTacACT--CGGCGAG | - | 5 | 2 | 0 | 2.20 | 1.10 |
| Chr6:133118535-133118557 | 133118540 | 3 | RNA | GGCCGAGGTCGACTACCGGCNRG | GGC--tcGcgGACTACCGGCTGG | - | 4 | 2 | 0 | 2.20 | 1.10 |
| Chr7:77522969-77522991 | 77522976 | 5 | RNA | GGCCGAGGTCGACTACCGGCNRG | GGCgcAGcTCcACT-CCaGCGAG | - | 5 | 1 | 0 | 2.20 | 1.10 |
| Chr6:78565157-78565179 | 78565172 | 5 | RNA | GGCCGAGGTCGACTACCGGCNRG | GGttGAGaT--AaTACCGtCTGG | + | 5 | 2 | 0 | 2.20 | 1.10 |
| Chr2:140635144-140635166 | 140635150 | 4 | RNA | GGCCGAGGTCGACTACCGGCNRG | GGaCGAcGaCGACTA--tGaTAG | - | 5 | 2 | 0 | 2.20 | 1.10 |
| Chr2:68204360-68204382 | 68204364 | 2 | RNA | GGCCGAGGTCGACTACCGGCNRG | GaCCGAGGaCGcCT-CgGGaTGG | - | 5 | 1 | 0 | 2.20 | 1.10 |
| Chr9:134814353-134814375 | 134814369 | 4 | RNA | GGCCGAGGTCGACTACCGGCNRG | GGgtGgGGTCGgCT--CGGgGAG | + | 5 | 2 | 0 | 2.20 | 1.10 |
| Chr7:90520160-90520182 | 90520178 | 2 | RNA | GGCCGAGGTCGACTACCGGCNRG | acCCGAaGTCGAtTAtC--CCGG | + | 5 | 2 | 0 | 2.20 | 1.10 |
| Chr10:90152598-90152620 | 90152605 | 5 | RNA | GGCCGAGGTCGACTACCGGCNRG | GGggGAGagCGA--AgCGGCCGG | - | 5 | 2 | 0 | 2.20 | 1.10 |
| Chr2:224205374-224205396 | 224205389 | 5 | RNA | GGCCGAGGTCGACTACCGGCNRG | GGgCG-GGcCaACccCCGGCCAG | + | 5 | 1 | 0 | 2.20 | 1.10 |
| Chr5:169614276-169614298 | 169614292 | 4 | RNA | GGCCGAGGTCGACTACCGGCNRG | G-aCGgGGTCGACaACgaGCGGG | + | 5 | 1 | 0 | 2.20 | 1.10 |
| Chr4:59754561-59754583 | 59754565 | 2 | RNA | GGCCGAGGTCGACTACCGGCNRG | GGaCcAGGTCG--TcCCaGtTGG | - | 5 | 2 | 0 | 2.20 | 1.10 |
| Chr8:36653470-36653492 | 36653476 | 4 | DNA | GGC-CGAGGTCGACTACCGGCNRG | GcCTCGAGGaCGtCTtCCaGCTGG | - | 5 | 1 | 0 | 2.20 | 1.10 |
| Chr1:118844117-118844139 | 118844122 | 3 | DNA | GGCCGAGGTCGAC-TACCGGCNRG | GGtCGAGGTCGACGTAgtcGCGGG | - | 4 | 1 | 0 | 2.20 | 1.10 |
| Chr2:84659413-84659435 | 84659431 | 2 | RNA | GGCCGAGGTCGACTACCGGCNRG | GGCCGAGcgCGA--AggGGgGAG | + | 5 | 2 | 0 | 2.20 | 1.10 |
| Chr6:71113471-71113493 | 71113489 | 2 | RNA | GGCCGAGGTCGACTACCGGCNRG | GGCCGAcaTgGA--ACtGGaCGG | + | 5 | 2 | 0 | 2.20 | 1.10 |
| Chr4:38243720-38243742 | 38243738 | 2 | RNA | GGCCGAGGTCGACTACCGGCNRG | GGaCcAGGTCG--TcCCaGtTGG | + | 5 | 2 | 0 | 2.20 | 1.10 |
| Chr1:121532612-121532634 | 121532618 | 4 | RNA | GGCCGAGGTCGACTACCGGCNRG | GtCCcAGGTCGAacACC--tGGG | - | 5 | 2 | 0 | 2.20 | 1.10 |
| Chr9:81920425-81920447 | 81920429 | 2 | RNA | GGCCGAGGTCGACTACCGGCNRG | GGttcctGTCGACTAC-GGCGAG | - | 5 | 1 | 0 | 2.20 | 1.10 |
| Chr4:183774110-183774132 | 183774126 | 4 | RNA | GGCCGAGGTCGACTACCGGCNRG | GGCCGA--TaGcCcgCCcGCCGG | + | 5 | 2 | 0 | 2.20 | 1.10 |
| Chr7:156354200-156354222 | 156354206 | 4 | RNA | GGCCGAGGTCGACTACCGGCNRG | GGaCGAcGaCGACTAC--GaTAG | - | 4 | 2 | 0 | 2.20 | 1.10 |
| Chr1:42844389-42844411 | 42844405 | 4 | RNA | GGCCGAGGTCGACTACCGGCNRG | G-aCGAGGTCGACgACgaGCGGG | + | 4 | 1 | 0 | 2.20 | 1.10 |
| Chr5:50647306-50647328 | 50647324 | 2 | RNA | GGCCGAGGTCGACTACCGGCNRG | GGaCGAGGcgG--cAaCGGCAAG | + | 5 | 2 | 0 | 2.20 | 1.10 |
| Chr4:219504700-219504722 | 219504706 | 4 | RNA | GGCCGAGGTCGACTACCGGCNRG | taCCG--GTCGACaACCaGCAGG | - | 4 | 2 | 0 | 2.20 | 1.10 |
| Chr9:138160150-138160172 | 138160165 | 5 | RNA | GGCCGAGGTCGACTACCGGCNRG | GagCGA--TtGACTACtGGCTGG | + | 4 | 2 | 0 | 2.20 | 1.10 |
| Chr2:18023142-18023164 | 18023147 | 3 | RNA | GGCCGAGGTCGACTACCGGCNRG | GcCCGccGTCGACctCC--CCAG | - | 5 | 2 | 0 | 2.20 | 1.10 |
| Chr2:4196458-4196480 | 4196475 | 3 | RNA | GGCCGAGGTCGACTACCGGCNRG | GGCgGcGGTCGACggC--GaCGG | + | 5 | 2 | 0 | 2.20 | 1.10 |
| Chr7:111741043-111741065 | 111741061 | 2 | DNA | GGCCGAGGTCGACTACCGG--CNRG | GGtCGAGGTCGAgTcCgaGCCCCGG | + | 5 | 2 | 0 | 2.20 | 1.10 |
| Chr6:5126873-5126895 | 5126880 | 5 | RNA | GGCCGAGGTCGACTACCGGCNRG | GGgCGAGGgCagCT--CGaCCGG | - | 5 | 2 | 0 | 2.20 | 1.10 |
| Chr7:41639652-41639674 | 41639669 | 3 | X | GGCCGAGGTCGACTACCGGCNRG | GGCactGGgCGACTACCaGCGGG | + | 5 | 0 | 0 | 2.20 | 1.10 |
| Chr6:137454491-137454513 | 137454508 | 3 | RNA | GGCCGAGGTCGACTACCGGCNRG | GGCCGAGcgCGA--ACgGGgGAG | + | 4 | 2 | 0 | 2.20 | 1.10 |
| Chr1:269237381-269237403 | 269237387 | 4 | RNA | GGCCGAGGTCGACTACCGGCNRG | G-aCGgGGTCGACgACgaGCGGG | - | 5 | 1 | 0 | 2.20 | 1.10 |
| Chr3:73434971-73434993 | 73434987 | 4 | RNA | GGCCGAGGTCGACTACCGGCNRG | GGgCGAG--CaACaACaGGgGAG | + | 5 | 2 | 0 | 2.20 | 1.10 |
| Chr2:43076225-43076247 | 43076242 | 3 | RNA | GGCCGAGGTCGACTACCGGCNRG | GcCCGAGccCGAgT--CGGtAGG | + | 5 | 2 | 0 | 2.20 | 1.10 |
| Chr3:190096978-190097000 | 190096995 | 3 | RNA | GGCCGAGGTCGACTACCGGCNRG | GGCtGAGGcCG-CTgCgGGgAAG | + | 5 | 1 | 0 | 2.20 | 1.10 |
| Chr5:157797674-157797696 | 157797691 | 3 | DNA | GGC-CGAGGTCGACTACCGGCNRG | GcCTCGgGGaCGtCTtCCGGCTGG | + | 5 | 1 | 0 | 2.20 | 1.10 |
| Chr3:75038537-75038559 | 75038542 | 3 | RNA | GGCCGAGGTCGACTACCGGCNRG | G-aCGgGGTCGACgACgaGCGGG | - | 5 | 1 | 0 | 2.20 | 1.10 |
| Chr1:149668086-149668108 | 149668090 | 2 | RNA | GGCCGAGGTCGACTACCGGCNRG | GGCaGcGGc--ACTtCgGGCGGG | - | 5 | 2 | 0 | 2.20 | 1.10 |
| Chr4:191273402-191273424 | 191273420 | 2 | RNA | GGCCGAGGTCGACTACCGGCNRG | G--CGAcGaCGACaACaGcCGAG | + | 5 | 2 | 0 | 2.20 | 1.10 |
| Chr8:68050886-68050908 | 68050902 | 4 | RNA | GGCCGAGGTCGACTACCGGCNRG | aGCCGcGGTCG--TACgtGgTGG | + | 5 | 2 | 0 | 2.20 | 1.10 |
| Chr1:293039009-293039031 | 293039014 | 3 | RNA | GGCCGAGGTCGACTACCGGCNRG | GGaaGAGtTCt--TACCaGCAGG | - | 5 | 2 | 0 | 2.20 | 1.10 |
| Chr8:130549515-130549537 | 130549533 | 2 | DNA | GGCCGAG--GTCGACTACCGGCNRG | GGCCGAGCCGTCtACTcggGcCGGG | + | 5 | 2 | 0 | 2.20 | 1.10 |
| Chr3:90533195-90533217 | 90533210 | 5 | RNA | GGCCGAGGTCGACTACCGGCNRG | GGCgGAGGTCG--TtCgGcaGAG | + | 5 | 2 | 0 | 2.20 | 1.10 |
| Chr4:94317152-94317174 | 94317167 | 5 | RNA | GGCCGAGGTCGACTACCGGCNRG | GGCCGAtcTCG--cAtCaGCAAG | + | 5 | 2 | 0 | 2.20 | 1.10 |
| Chr7:114277476-114277498 | 114277482 | 4 | RNA | GGCCGAGGTCGACTACCGGCNRG | GGgCcAGGTCGAgTg--GGgGAG | - | 5 | 2 | 0 | 2.20 | 1.10 |
| Chr9:68194936-68194958 | 68194953 | 3 | RNA | GGCCGAGGTCGACTACCGGCNRG | cGCCGtGGT-GtCTACtGGaGGG | + | 5 | 1 | 0 | 2.20 | 1.10 |
| Chr4:128501752-128501774 | 128501758 | 4 | DNA | GGCCGAGGTCGACTACCGG--CNRG | GGCCGAGGcCGAgTcCgaGCCCTGG | - | 5 | 2 | 0 | 2.20 | 1.10 |
| Chr6:22010571-22010593 | 22010588 | 3 | DNA | GGCCGAGGTCGA--CTACCGGCNRG | GcgCcAtcTCGATTCTACCGGCGAG | + | 5 | 2 | 0 | 2.20 | 1.10 |
| Chr3:187613975-187613997 | 187613992 | 3 | RNA | GGCCGAGGTCGACTACCGGCNRG | GGCCGAGGcCGAgctC--GCCGG | + | 4 | 2 | 0 | 2.20 | 1.10 |
| Chr3:140909124-140909146 | 140909139 | 5 | RNA | GGCCGAGGTCGACTACCGGCNRG | GGCC-AGaTCtACaACgaGCTGG | + | 5 | 1 | 0 | 2.20 | 1.10 |
| Chr6:85084128-85084150 | 85084144 | 4 | RNA | GGCCGAGGTCGACTACCGGCNRG | GGaCGAcGcCG-CTgCCtGCAGG | + | 5 | 1 | 0 | 2.20 | 1.10 |
| Chr3:41909868-41909890 | 41909874 | 4 | RNA | GGCCGAGGTCGACTACCGGCNRG | GGCCGAcGgC--CcgCCcGCCGG | - | 5 | 2 | 0 | 2.20 | 1.10 |

| | | | | | | | | | | | |
|---|---|---|---|---|---|---|---|---|---|---|---|
| Chr8:47843164-47843186 | 47843170 | 4 | RNA | GGCCGAGGTCGACTACCGGCNRG | GGCCGgGcTgGgCTg--GGCTGG | - | 5 | 2 | 0 | 2.20 | 1.10 |
| Chr5:209544940-209544962 | 209544945 | 3 | DNA | GG--CCGAGGTCGACTACCGGCNRG | GGACCCtAcaTCcACTACCaGCAGG | - | 5 | 2 | 0 | 2.20 | 1.10 |
| Chr5:204417049-204417071 | 204417056 | 5 | RNA | GGCCGAGGTCGACTACCGGCNRG | G-aCGgGGTCGACaACgaGCGGG | - | 5 | 1 | 0 | 2.20 | 1.10 |
| Chr3:203539718-203539740 | 203539722 | 2 | RNA | GGCCGAGGTCGACTACCGGCNRG | G--CGAGGTCGcCcAtCtGCGAG | - | 4 | 2 | 0 | 2.20 | 1.10 |
| Chr4:79045468-79045490 | 79045472 | 2 | RNA | GGCCGAGGTCGACTACCGGCNRG | caCCGAGtTgGAgTA--GGCGGG | - | 5 | 2 | 0 | 2.20 | 1.10 |
| Chr4:73255875-73255897 | 73255893 | 2 | RNA | GGCCGAGGTCGACTACCGGCNRG | tG--GAGGTtGcCaACCGGaCAG | + | 5 | 2 | 0 | 2.20 | 1.10 |
| Chr4:241491282-241491304 | 241491300 | 2 | RNA | GGCCGAGGTCGACTACCGGCNRG | GGCgGAGGTCG--TtCgGcaGAG | + | 5 | 2 | 0 | 2.20 | 1.10 |
| Chr10:127161537-127161559 | 127161555 | 2 | DNA | GG--CCGAGGTCGACTACCGGCNRG | GGTCCCGAGGTCGAtccCCctCGGG | + | 5 | 2 | 0 | 2.20 | 1.10 |
| Chr1:286188610-286188632 | 286188615 | 3 | RNA | GGCCGAGGTCGACTACCGGCNRG | GGCCGAGtcCGAgccCC--CGGG | - | 5 | 2 | 0 | 2.20 | 1.10 |
| Chr1:204172671-204172693 | 204172675 | 2 | RNA | GGCCGAGGTCGACTACCGGCNRG | GGtCGAGGcCGAgT--CcGgGGG | - | 5 | 2 | 0 | 2.20 | 1.10 |
| Chr10:43160552-43160574 | 43160568 | 4 | RNA | GGCCGAGGTCGACTACCGGCNRG | GGC-GAGGTCGAgagCgGGCGGG | + | 4 | 1 | 0 | 2.20 | 1.10 |
| Chr7:17750473-17750495 | 17750478 | 3 | DNA | GGCCGAGGTCGACTA--CCGGCNRG | ctCCaAGGcCGACcAATCCGGCAAG | - | 5 | 2 | 0 | 2.20 | 1.10 |
| Chr3:99690263-99690285 | 99690267 | 2 | RNA | GGCCGAGGTCGACTACCGGCNRG | GGCCG-cGTCtACTAtCaGtAGG | - | 5 | 1 | 0 | 2.20 | 1.10 |
| Chr10:120823197-120823219 | 120823201 | 2 | RNA | GGCCGAGGTCGACTACCGGCNRG | GGCCGAGGcCtcCT-CgGGCTGG | - | 4 | 1 | 0 | 2.20 | 1.10 |
| Chr7:74686790-74686812 | 74686808 | 2 | DNA | GGCCGAGGTC-GACTACCGGCNRG | GGCCGAGGcCTGAggtCgGGCGAG | + | 5 | 1 | 0 | 2.20 | 1.10 |
| Chr3:56717446-56717468 | 56717451 | 3 | DNA | GGCCGAGGTCGACTACC--GGCNRG | GcCCGAGagCaACcACCGTGGCCAG | - | 5 | 2 | 0 | 2.20 | 1.10 |
| Chr8:160479712-160479734 | 160479717 | 3 | RNA | GGCCGAGGTCGACTACCGGCNRG | GGCCG-GGTCatCcACtGGCGGG | - | 4 | 1 | 0 | 2.20 | 1.10 |
| Chr1:120664858-120664880 | 120664864 | 4 | RNA | GGCCGAGGTCGACTACCGGCNRG | GG--GAGGTCGAgcACCGGCCGG | - | 2 | 2 | 0 | 2.20 | 1.10 |
| Chr7:93432459-93432481 | 93432476 | 3 | DNA | GGCCGAGGT--CGACTACCGGCNRG | GGtCGtGGTGGCGAactCCGGCGAG | + | 5 | 2 | 0 | 2.20 | 1.10 |
| Chr2:112185830-112185852 | 112185835 | 3 | DNA | GGC--CGAGGTCGACTACCGGCNRG | GGCCTCGgGGgCtACacCCGGCGGG | - | 5 | 2 | 0 | 2.20 | 1.10 |
| Chr7:19971692-19971714 | 19971697 | 3 | DNA | GGCCG--AGGTCGACTACCGGCNRG | GGtCGTTAGGTCagCTACCttCCGG | - | 5 | 2 | 0 | 2.20 | 1.10 |
| Chr1:16101231-16101253 | 16101237 | 4 | RNA | GGCCGAGGTCGACTACCGGCNRG | aGCCGAGGgC-ACctCgGGCAAG | - | 5 | 1 | 0 | 2.20 | 1.10 |
| Chr10:30147091-30147113 | 30147108 | 3 | RNA | GGCCGAGGTCGACTACCGGCNRG | G-CCGcGGcCGAacACCGaCGAG | + | 5 | 1 | 0 | 2.20 | 1.10 |
| Chr1:122440334-122440356 | 122440350 | 4 | RNA | GGCCGAGGTCGACTACCGGCNRG | GGCCt--GcCaACTtCCGGgCAG | + | 5 | 2 | 0 | 2.20 | 1.10 |
| Chr4:83007020-83007042 | 83007036 | 4 | RNA | GGCCGAGGTCGACTACCGGCNRG | GGCCGAGccCGAg--CCaGgGGG | + | 5 | 2 | 0 | 2.20 | 1.10 |
| Chr2:49908336-49908358 | 49908341 | 3 | RNA | GGCCGAGGTCGACTACCGGCNRG | GtgCaAGcTC--CTcCCGGCGAG | - | 5 | 2 | 0 | 2.20 | 1.10 |
| Chr5:179981708-179981730 | 179981726 | 2 | RNA | GGCCGAGGTCGACTACCGGCNRG | GGCCGAGGgCG--cgCgcGCGGG | + | 5 | 2 | 0 | 2.20 | 1.10 |
| Chr6:116352215-116352237 | 116352222 | 5 | RNA | GGCCGAGGTCGACTACCGGCNRG | GGC--ttGcgGACTACCGGCTGG | - | 4 | 2 | 0 | 2.20 | 1.10 |
| Chr1:54618101-54618123 | 54618106 | 3 | RNA | GGCCGAGGTCGACTACCGGCNRG | GGC--tcGTgGACTACCGtCTGG | - | 4 | 2 | 0 | 2.20 | 1.10 |
| Chr3:142032089-142032111 | 142032096 | 5 | RNA | GGCCGAGGTCGACTACCGGCNRG | G--CaAGGaCGACTAgCGGgGAG | - | 4 | 2 | 0 | 2.20 | 1.10 |
| Chr1:116354814-116354836 | 116354831 | 3 | RNA | GGCCGAGGTCGACTACCGGCNRG | GGCCcAcGgCG--TttCGGCCGG | + | 5 | 2 | 0 | 2.20 | 1.10 |
| Chr4:47466419-47466441 | 47466425 | 4 | RNA | GGCCGAGGTCGACTACCGGCNRG | GtCCG--GTgcACcACCGGaCAG | - | 5 | 2 | 0 | 2.20 | 1.10 |
| Chr10:104950422-104950444 | 104950437 | 5 | RNA | GGCCGAGGTCGACTACCGGCNRG | GGC--AGGcCaAgctCCGGCCAG | + | 5 | 2 | 0 | 2.20 | 1.10 |
| Chr10:45567296-45567318 | 45567312 | 4 | DNA | G--GCCGAGGTCGACTACCGGCNRG | GCCGCCGgGtTCGACTcCgGGtCAG | + | 5 | 2 | 0 | 2.20 | 1.10 |
| Chr7:169893467-169893489 | 169893482 | 5 | RNA | GGCCGAGGTCGACTACCGGCNRG | GGCCGAGc-CGtCcACtaGCGGG | + | 5 | 1 | 0 | 2.20 | 1.10 |
| Chr4:189298535-189298557 | 189298551 | 4 | RNA | GGCCGAGGTCGACTACCGGCNRG | GGaCGAGccCGACT-CtGGtTAG | + | 5 | 1 | 0 | 2.20 | 1.10 |
| Chr8:42713073-42713095 | 42713088 | 5 | DNA | GGCCGAGGTCGAC--TACCGGCNRG | GGCCGActTCGACCATcCCcGaCGG | + | 5 | 2 | 0 | 2.20 | 1.10 |
| Chr3:225281898-225281920 | 225281913 | 5 | RNA | GGCCGAGGTCGACTACCGGCNRG | GcCaGtGGaCGgC--CCGGCCAG | + | 5 | 2 | 0 | 2.20 | 1.10 |
| Chr5:214864117-214864139 | 214864135 | 2 | RNA | GGCCGAGGTCGACTACCGGCNRG | GGCtGgGGTaG--cACCGGgAGG | + | 5 | 2 | 0 | 2.20 | 1.10 |
| Chr9:102201445-102201467 | 102201450 | 3 | RNA | GGCCGAGGTCGACTACCGGCNRG | GGggGAGGTgG--TAgaGGCTGG | - | 5 | 2 | 0 | 2.20 | 1.10 |
| Chr6:165165861-165165883 | 165165866 | 3 | RNA | GGCCGAGGTCGACTACCGGCNRG | GGatGAGGagGACT--CGGaCGG | - | 5 | 2 | 0 | 2.20 | 1.10 |
| Chr3:111276764-111276786 | 111276768 | 2 | RNA | GGCCGAGGTCGACTACCGGCNRG | GtCCG--GTaGAtcACCcGCAGG | - | 5 | 2 | 0 | 2.20 | 1.10 |
| Chr6:66915847-66915869 | 66915863 | 4 | RNA | GGCCGAGGTCGACTACCGGCNRG | cGCCGAGcTCGgCcAC--GtCAG | + | 5 | 2 | 0 | 2.20 | 1.10 |
| Chr3:135142620-135142642 | 135142626 | 4 | RNA | GGCCGAGGTCGACTACCGGCNRG | GGCCGAGGgt-ACctCgGGCTAG | - | 5 | 1 | 0 | 2.20 | 1.10 |
| Chr10:146259682-146259704 | 146259687 | 3 | DNA | GGCCGAGGTCGAC--TACCGGCNRG | GGgCGAGGaCGACGGcgCCaGCGAG | - | 5 | 2 | 0 | 2.20 | 1.10 |
| Chr8:129664790-129664812 | 129664796 | 4 | RNA | GGCCGAGGTCGACTACCGGCNRG | G-aCGgGGTCGACgACgaGCGGG | - | 5 | 1 | 0 | 2.20 | 1.10 |
| Chr4:209203159-209203181 | 209203163 | 2 | RNA | GGCCGAGGTCGACTACCGGCNRG | GG--GAGGaCtAaTAtCGGgGGG | - | 5 | 2 | 0 | 2.20 | 1.10 |
| Chr8:42382106-42382128 | 42382112 | 4 | DNA | GGCC--GAGGTCGACTACCGGCNRG | cGCCTTGgGGaCGtCTtCCGGCTGG | - | 5 | 2 | 1 | 1.10 | 1.05 |
| Chr2:166531357-166531379 | 166531361 | 2 | RNA | GGCCGAGGTCGACTACCGGCNRG | GGC--AGGcCGAgTACCGGCCGG | - | 2 | 2 | 1 | 1.10 | 1.05 |
| Chr7:162222442-162222464 | 162222448 | 4 | DNA | GGC-CGAGGTCGACTACCGGCNRG | GcCTCGAGGaCGtCTtCCGGCTGG | - | 4 | 1 | 1 | 1.10 | 1.05 |
| Chr6:153883486-153883508 | 153883492 | 4 | RNA | GGCCGAGGTCGACTACCGGCNRG | GGCgGctGTCGA--ACCtGaAAG | - | 5 | 2 | 1 | 1.10 | 1.05 |
| Chr4:157776264-157776286 | 157776281 | 3 | DNA | GGCCGA--GGTCGACTACCGGCNRG | GGCgcATCGGgCGAgTACtGGCAGG | + | 5 | 2 | 2 | 0.00 | 1.00 |
| Chr5:70912068-70912090 | 70912085 | 3 | RNA | GGCCGAGGTCGACTACCGGCNRG | GtCCG--GTgcACcACCGGaCAG | + | 5 | 2 | 2 | 0.00 | 1.00 |
| Chr2:192335731-192335753 | 192335738 | 5 | RNA | GGCCGAGGTCGACTACCGGCNRG | GGgCG-GGcCaACctCCGGCCGG | - | 5 | 1 | 2 | 0.00 | 1.00 |
| Chr7:41494072-41494094 | 41494089 | 3 | RNA | GGCCGAGGTCGACTACCGGCNRG | GGgCG-GGcCaACctCCGGCCGG | + | 5 | 1 | 2 | 0.00 | 1.00 |
| Chr9:85864022-85864044 | 85864029 | 5 | RNA | GGCCGAGGTCGACTACCGGCNRG | GGCCGAGcTgGgC--CgGGCCGG | - | 4 | 2 | 2 | 0.00 | 1.00 |
| Chr8:17821069-17821091 | 17821076 | 5 | RNA | GGCCGAGGTCGACTACCGGCNRG | GGC-GAGGTCGAggACCttgGGG | - | 5 | 1 | 2 | 0.00 | 1.00 |
| Chr5:82156214-82156236 | 82156221 | 5 | DNA | GGCCGAGGTCGACTA-CCGGCNRG | GtgCGAGGcgGACTAACCGGCCGG | - | 4 | 1 | 2 | 0.00 | 1.00 |
| Chr8:134202546-134202568 | 134202553 | 5 | DNA | GGC--CGAGGTCGACTACCGGCNRG | GGCCTCGgGGgCtACacCCGGCGGG | - | 5 | 2 | 2 | 0.00 | 1.00 |
| Chr1:29555838-29555860 | 29555845 | 5 | RNA | GGCCGAGGTCGACTACCGGCNRG | GcCCGAGGcCGA--ACtcGtGGG | - | 5 | 2 | 2 | 0.00 | 1.00 |
| Chr8:70500976-70500998 | 70500983 | 5 | RNA | GGCCGAGGTCGACTACCGGCNRG | GGCaGAGGgtG--cACCGGaTAG | - | 5 | 2 | 2 | 0.00 | 1.00 |
| Chr7:46564084-46564106 | 46564101 | 3 | RNA | GGCCGAGGTCGACTACCGGCNRG | G--CGAGcTCGACTgtgGGaGGG | + | 5 | 2 | 2 | 0.00 | 1.00 |
| Chr4:25306659-25306681 | 25306666 | 5 | RNA | GGCCGAGGTCGACTACCGGCNRG | GGCC-AGGTCGtCcACtGGCGGG | - | 3 | 1 | 2 | 0.00 | 1.00 |
| Chr2:237257-237279 | 237264 | 5 | RNA | GGCCGAGGTCGACTACCGGCNRG | aGCCcAGGaCG--TACaaGCTGG | - | 5 | 2 | 2 | 0.00 | 1.00 |
| Chr7:147128045-147128067 | 147128052 | 5 | RNA | GGCCGAGGTCGACTACCGGCNRG | GGC--tcGcgGACTACCGGCTGG | - | 4 | 2 | 2 | 0.00 | 1.00 |
| Chr3:177682486-177682508 | 177682503 | 3 | RNA | GGCCGAGGTCGACTACCGGCNRG | GGCCGAcGTCGAggAa-GaCGAG | + | 5 | 1 | 2 | 0.00 | 1.00 |
| Chr4:96989885-96989907 | 96989902 | 3 | DNA | GGCCGAGGTCGACTAC--CGGCNRG | GcCCGAcaTCGcCTACGTCGtCCAG | + | 5 | 2 | 2 | 0.00 | 1.00 |
| Chr5:4621852-4621874 | 4621859 | 5 | RNA | GGCCGAGGTCGACTACCGGCNRG | cGCCtAGGgCtgCTACC--CTAG | - | 5 | 2 | 2 | 0.00 | 1.00 |
| Chr5:148227169-148227191 | 148227186 | 3 | RNA | GGCCGAGGTCGACTACCGGCNRG | G--CGAGGTCctCctCCtGCCAG | + | 5 | 2 | 2 | 0.00 | 1.00 |
| Chr9:107839649-107839671 | 107839656 | 5 | RNA | GGCCGAGGTCGACTACCGGCNRG | cGaCGAGcTCGACT-CtGGtTGG | - | 5 | 1 | 2 | 0.00 | 1.00 |
| Chr1:238446048-238446070 | 238446055 | 5 | RNA | GGCCGAGGTCGACTACCGGCNRG | GGgCGcG--aGgCTACtGGCGGG | - | 5 | 2 | 2 | 0.00 | 1.00 |
| Chr9:44328395-44328417 | 44328402 | 5 | RNA | GGCCGAGGTCGACTACCGGCNRG | GaCCGtGcTCcACcACCG-CCAG | - | 5 | 1 | 2 | 0.00 | 1.00 |
| Chr5:134292209-134292231 | 134292216 | 5 | RNA | GGCCGAGGTCGACTACCGGCNRG | GGtaGAGGTCGgCTgCC--CCAG | - | 4 | 2 | 2 | 0.00 | 1.00 |
| Chr5:108954613-108954635 | 108954620 | 5 | RNA | GGCCGAGGTCGACTACCGGCNRG | GGC-GAGGTCGACgACgcGaTGG | - | 4 | 1 | 2 | 0.00 | 1.00 |
| Chr3:101905042-101905064 | 101905059 | 3 | RNA | GGCCGAGGTCGACTACCGGCNRG | GGCCGAGcTgGgCcAC--GCTAG | + | 4 | 2 | 2 | 0.00 | 1.00 |
| Chr4:116631328-116631350 | 116631335 | 5 | RNA | GGCCGAGGTCGACTACCGGCNRG | GGCgGcGGT-GACTACtcGCTGG | - | 4 | 1 | 2 | 0.00 | 1.00 |
| Chr3:215262195-215262217 | 215262212 | 3 | RNA | GGCCGAGGTCGACTACCGGCNRG | GtCaGAGacCGAt--CCGGCCAG | + | 5 | 2 | 2 | 0.00 | 1.00 |
| Chr6:25868780-25868802 | 25868787 | 5 | DNA | GGCCGAGGTCGACTACCGG--CNRG | GGCCGgGGTCGgCcACtGGCAtGAG | - | 5 | 2 | 2 | 0.00 | 1.00 |
| Chr10:60042644-60042666 | 60042651 | 5 | DNA | GGCCGAGGT--CGACTACCGGCNRG | GGCCtAGaTCTCGcCTcCCGGgAGG | - | 5 | 2 | 2 | 0.00 | 1.00 |
| Chr4:67494805-67494827 | 67494812 | 5 | RNA | GGCCGAGGTCGACTACCGGCNRG | GG--GAcGTCGACaggCGGgCAG | - | 5 | 2 | 2 | 0.00 | 1.00 |
| Chr6:48736027-48736049 | 48736044 | 3 | RNA | GGCCGAGGTCGACTACCGGCNRG | GGCCGAG--CGcCagCCacCAGG | + | 5 | 2 | 2 | 0.00 | 1.00 |
| Chr1:7611163-7611185 | 7611180 | 3 | DNA | G--GCCGAGGTCGACTACCGGCNRG | GATGaCtAGGcgGACcACCGGCGGG | + | 5 | 2 | 2 | 0.00 | 1.00 |
| Chr7:33669792-33669814 | 33669799 | 5 | DNA | GGCCGAGGT-CGACTACCGGCNRG | GGCgGAGGTGCtgtTACCGtCGAG | - | 5 | 1 | 2 | 0.00 | 1.00 |
| Chr3:181977050-181977072 | 181977057 | 5 | RNA | GGCCGAGGTCGACTACCGGCNRG | GaCgaAGGTgGACTA--GtCAGG | - | 5 | 2 | 2 | 0.00 | 1.00 |
| Chr2:240883021-240883043 | 240883038 | 3 | RNA | GGCCGAGGTCGACTACCGGCNRG | G-CCGAGGgCGcCgACCaaCTGG | + | 5 | 1 | 2 | 0.00 | 1.00 |
| Chr2:34587738-34587760 | 34587745 | 5 | RNA | GGCCGAGGTCGACTACCGGCNRG | aGCCGAGGgtGcCT-CgGGCGGG | - | 5 | 1 | 2 | 0.00 | 1.00 |
| Chr6:171197823-171197845 | 171197830 | 5 | RNA | GGCCGAGGTCGACTACCGGCNRG | cGgCGAGcTtG--TACCGGCCGG | - | 4 | 2 | 2 | 0.00 | 1.00 |
| Chr5:88124067-88124089 | 88124074 | 5 | RNA | GGCCGAGGTCGACTACCGGCNRG | GGatGAGGagGACT--CGGaCGG | - | 5 | 2 | 2 | 0.00 | 1.00 |
| Chr4:25873734-25873756 | 25873751 | 3 | RNA | GGCCGAGGTCGACTACCGGCNRG | GGaCGAtccCGACT-CCGGtTAG | + | 5 | 1 | 2 | 0.00 | 1.00 |
| Chr8:34018605-34018627 | 34018622 | 3 | RNA | GGCCGAGGTCGACTACCGGCNRG | GGaCcAGtTCGACTA--aGtCAG | + | 5 | 2 | 2 | 0.00 | 1.00 |
| Chr6:36346864-36346886 | 36346871 | 5 | RNA | GGCCGAGGTCGACTACCGGCNRG | GaCCGgGGaatACT-CCGGCGAG | - | 5 | 1 | 2 | 0.00 | 1.00 |
| Chr2:188034390-188034412 | 188034407 | 3 | RNA | GGCCGAGGTCGACTACCGGCNRG | GcCCGAGaTCGACctCa--CCGG | + | 5 | 2 | 2 | 0.00 | 1.00 |
| Chr3:64478044-64478066 | 64478051 | 5 | RNA | GGCCGAGGTCGACTACCGGCNRG | G-aCGgGGTCGACgACgaGCGGG | - | 5 | 1 | 2 | 0.00 | 1.00 |
| Chr7:73682382-73682404 | 73682389 | 5 | RNA | GGCCGAGGTCGACTACCGGCNRG | tGCCtgGGcCGACTg--GGCTGG | - | 5 | 2 | 2 | 0.00 | 1.00 |
| Chr2:71741824-71741846 | 71741831 | 5 | RNA | GGCCGAGGTCGACTACCGGCNRG | GaCtaAGGTCaACTACt--CGGG | - | 5 | 2 | 2 | 0.00 | 1.00 |
| Chr4:153428986-153429008 | 153428993 | 5 | RNA | GGCCGAGGTCGACTACCGGCNRG | GGCCGcGGcCG--cAgCGtCGGG | - | 5 | 2 | 2 | 0.00 | 1.00 |
| Chr9:58337476-58337498 | 58337493 | 3 | DNA | GGCCGAGGTCGA--CTACCGGCNRG | GcgCcAtcTCGATTCTACCGGCGAG | + | 5 | 2 | 2 | 0.00 | 1.00 |
| Chr9:132678364-132678386 | 132678381 | 3 | RNA | GGCCGAGGTCGACTACCGGCNRG | GGCaGAG--CaAgcACCGGCCGG | + | 4 | 2 | 2 | 0.00 | 1.00 |
| Chr2:207168615-207168637 | 207168622 | 5 | RNA | GGCCGAGGTCGACTACCGGCNRG | G-aCGgGGTCGACaACgaGCTGG | - | 5 | 1 | 2 | 0.00 | 1.00 |
| Chr4:179402651-179402673 | 179402658 | 5 | RNA | GGCCGAGGTCGACTACCGGCNRG | GGCtGAGGgtGcCT-CgGGCTGG | - | 5 | 1 | 2 | 0.00 | 1.00 |
| Chr6:78088383-78088405 | 78088390 | 5 | RNA | GGCCGAGGTCGACTACCGGCNRG | tGCCGAaGctGA--ACCGaCCGG | - | 5 | 2 | 2 | 0.00 | 1.00 |
| Chr2:240810882-240810904 | 240810889 | 5 | RNA | GGCCGAGGTCGACTACCGGCNRG | GGC-GtGGgCGACTAgCGGCTGG | - | 3 | 1 | 2 | 0.00 | 1.00 |
| Chr2:72299808-72299830 | 72299825 | 3 | DNA | GGCCGA--GGTCGACTACCGGCNRG | GGCgGACAGGTgcACgACaGGCGGG | + | 5 | 2 | 2 | 0.00 | 1.00 |
| Chr2:194745311-194745333 | 194745328 | 3 | RNA | GGCCGAGGTCGACTACCGGCNRG | GGCtGAGccCGgC--CCGGaGAG | + | 5 | 2 | 2 | 0.00 | 1.00 |
| Chr2:112259603-112259625 | 112259610 | 5 | RNA | GGCCGAGGTCGACTACCGGCNRG | GctCGAGGTgcACTAC-GGgTAG | - | 5 | 1 | 2 | 0.00 | 1.00 |
| Chr10:88229475-88229497 | 88229492 | 3 | DNA | GGCCGAGGTCGACTACCGG--CNRG | GGtCGAGGTCGAgTcCgaGCCCCGG | + | 5 | 2 | 2 | 0.00 | 1.00 |
| Chr3:235106549-235106571 | 235106566 | 3 | DNA | GGCCGAGGTCGACTAC--CGGCNRG | cGCCGAtGcCGACgACGACGGCCAG | + | 4 | 2 | 2 | 0.00 | 1.00 |
| Chr5:112932903-112932925 | 112932910 | 5 | DNA | GGCCGAGGTCG--ACTACCGGCNRG | GGCCGgGGTCGGGcacACCGGCGGG | - | 4 | 2 | 2 | 0.00 | 1.00 |
| Chr7:21230373-21230395 | 21230380 | 5 | RNA | GGCCGAGGTCGACTACCGGCNRG | GGCCGAGcgCGA--AggGGgGAG | - | 5 | 2 | 2 | 0.00 | 1.00 |
| Chr6:125991478-125991500 | 125991485 | 5 | RNA | GGCCGAGGTCGACTACCGGCNRG | GGC-GcGGTCGAactCtGGCGAG | - | 5 | 1 | 2 | 0.00 | 1.00 |
| Chr7:159024964-159024986 | 159024971 | 5 | DNA | GGCCGAGGTCGACTACC--GGCNRG | GGCaGAGacCGtCTtCCGAGGCCGG | - | 5 | 2 | 2 | 0.00 | 1.00 |
| Chr1:255154078-255154100 | 255154095 | 3 | RNA | GGCCGAGGTCGACTACCGGCNRG | G-aCGgGGTCGACgACgaGCGGG | + | 5 | 1 | 2 | 0.00 | 1.00 |
| Chr7:34149954-34149976 | 34149971 | 3 | RNA | GGCCGAGGTCGACTACCGGCNRG | tGtCGtGcTCGAC--CCtGCAAG | + | 5 | 2 | 2 | 0.00 | 1.00 |
| Chr2:240043495-240043517 | 240043502 | 5 | RNA | GGCCGAGGTCGACTACCGGCNRG | GtCCG--GTgcACcACCGGaTAG | - | 5 | 2 | 2 | 0.00 | 1.00 |
| Chr4:137440406-137440428 | 137440413 | 5 | DNA | GGCCGAGGTCGACT--ACCGGCNRG | GtCgGAGGTgGACTGGAaCtGCAGG | - | 5 | 2 | 2 | 0.00 | 1.00 |
| Chr8:107558521-107558543 | 107558538 | 3 | RNA | GGCCGAGGTCGACTACCGGCNRG | GcCCGAG--CGcCcACCtGCCGG | + | 4 | 2 | 2 | 0.00 | 1.00 |
| Chr1:304699677-304699699 | 304699684 | 5 | RNA | GGCCGAGGTCGACTACCGGCNRG | G-aCGgGGTCGACaACgaGCGGG | - | 5 | 1 | 2 | 0.00 | 1.00 |
| Chr4:40328260-40328282 | 40328277 | 3 | RNA | GGCCGAGGTCGACTACCGGCNRG | GGC--ttGcgGACTACCGGCTGG | + | 4 | 2 | 2 | 0.00 | 1.00 |
| Chr9:127799939-127799961 | 127799956 | 3 | RNA | GGCCGAGGTCGACTACCGGCNRG | GGaCtAGGTCG--TcCCaGtTGG | + | 5 | 2 | 2 | 0.00 | 1.00 |
| Chr5:112627098-112627120 | 112627115 | 3 | RNA | GGCCGAGGTCGACTACCGGCNRG | tGCCcAGG--GACaAgCGGgCAG | + | 5 | 2 | 2 | 0.00 | 1.00 |
| Chr5:23639270-23639292 | 23639277 | 5 | DNA | GGC--CGAGGTCGACTACCGGCNRG | GGCCTCGgGGgCtACacCCGGCGGG | - | 5 | 2 | 2 | 0.00 | 1.00 |
| Chr6:63927539-63927561 | 63927546 | 5 | RNA | GGCCGAGGTCGACTACCGGCNRG | aaCaGAGGc--ACgACCGGCAGG | - | 5 | 2 | 2 | 0.00 | 1.00 |
| Chr7:146105289-146105311 | 146105306 | 3 | RNA | GGCCGAGGTCGACTACCGGCNRG | GGgCG-GGcCaACctCCGGCCAG | + | 5 | 1 | 2 | 0.00 | 1.00 |
| Chr2:114464788-114464810 | 114464795 | 5 | RNA | GGCCGAGGTCGACTACCGGCNRG | GGCCGAGGTCGA-TggaGaCTGG | - | 4 | 1 | 2 | 0.00 | 1.00 |
| Chr1:54429794-54429816 | 54429811 | 3 | DNA | G--GCCGAGGTCGACTACCGGCNRG | GATGaCGgGGTCGACaACgaGCGGG | + | 5 | 2 | 2 | 0.00 | 1.00 |
| Chr2:16510172-16510194 | 16510189 | 3 | RNA | GGCCGAGGTCGACTACCGGCNRG | GtCCG--GaaGAaTACCGGaAAG | + | 5 | 2 | 2 | 0.00 | 1.00 |

| | | | | | | | | | | |
|---|---|---|---|---|---|---|---|---|---|---|
| Chr2:1074862-1074884 | 1074879 | 3 | DNA | GGCC-GAGGTCGACTACCGGCNRG | GGCCCGAGGgCGgCctCgGGCAAG | + | 5 | 1 | 2 | 0.00 | 1.00 |
| Chr8:13708258-13708280 | 13708275 | 3 | RNA | GGCCGAGGTCGACTACCGGCNRG | GGtCGAGGcCGAgT--CtGaGGG | + | 5 | 2 | 2 | 0.00 | 1.00 |
| Chr1:127278942-127278964 | 127278949 | 5 | RNA | GGCCGAGGTCGACTACCGGCNRG | GGCCcAtGgCG--TttCGGCCGG | - | 5 | 2 | 2 | 0.00 | 1.00 |
| Chr7:182137973-182137995 | 182137980 | 5 | X | GGCCGAGGTCGACTACCGGCNRG | GGCCGAGGcaGACTgCaGcCTGG | - | 5 | 0 | 2 | 0.00 | 1.00 |
| Chr7:138466026-138466048 | 138466043 | 3 | RNA | GGCCGAGGTCGACTACCGGCNRG | G-tCGgGGTCGACaACgaGCGGG | + | 5 | 1 | 2 | 0.00 | 1.00 |
| Chr1:23354265-23354287 | 23354272 | 5 | RNA | GGCCGAGGTCGACTACCGGCNRG | GcCgGAGccCGAC--CCGtCAGG | - | 5 | 2 | 2 | 0.00 | 1.00 |
| Chr6:21058460-21058482 | 21058467 | 5 | DNA | GGCCGAGGTCGAC-TACCGGCNRG | GGCCGAGacgGACGTAtgGGCTGG | - | 5 | 1 | 2 | 0.00 | 1.00 |
| Chr5:150123206-150123228 | 150123213 | 5 | RNA | GGCCGAGGTCGACTACCGGCNRG | GGCCGAtGatGgCTA--GGgAGG | - | 5 | 2 | 2 | 0.00 | 1.00 |
| Chr3:93193878-93193900 | 93193885 | 5 | RNA | GGCCGAGGTCGACTACCGGCNRG | GcCCGAGGaCaACcACC--aTGG | - | 5 | 2 | 2 | 0.00 | 1.00 |
| Chr2:189372054-189372076 | 189372071 | 3 | RNA | GGCCGAGGTCGACTACCGGCNRG | GGCCGAttggGgCTAC-GGCTGG | + | 5 | 1 | 2 | 0.00 | 1.00 |
| Chr2:153252942-153252964 | 153252959 | 3 | RNA | GGCCGAGGTCGACTACCGGCNRG | cGaCGAGGTgGA-ggCCGGCAGG | + | 5 | 1 | 2 | 0.00 | 1.00 |
| Chr2:42268940-42268962 | 42268957 | 3 | RNA | GGCCGAGGTCGACTACCGGCNRG | GGCgGAGGTCG--TtCgGcaGAG | + | 5 | 2 | 2 | 0.00 | 1.00 |
| Chr1:10436165-10436187 | 10436172 | 5 | RNA | GGCCGAGGTCGACTACCGGCNRG | G-aCGgGGTCGACaACgaGCGGG | - | 5 | 1 | 2 | 0.00 | 1.00 |
| Chr7:135877158-135877180 | 135877165 | 5 | RNA | GGCCGAGGTCGACTACCGGCNRG | cGtCGAt-TCcACTACCGGCGGG | - | 4 | 1 | 2 | 0.00 | 1.00 |
| Chr5:195365636-195365658 | 195365643 | 5 | RNA | GGCCGAGGTCGACTACCGGCNRG | GGCaGtGGTaGACgAg-GGCTGG | - | 5 | 1 | 2 | 0.00 | 1.00 |
| Chr3:196723329-196723351 | 196723336 | 5 | RNA | GGCCGAGGTCGACTACCGGCNRG | GGCCGgaGTCGA--ACgtGaGAG | - | 5 | 2 | 2 | 0.00 | 1.00 |
| Chr5:189152310-189152332 | 189152317 | 5 | RNA | GGCCGAGGTCGACTACCGGCNRG | GGCCGgcGgCGACaAC--GCCGG | - | 4 | 2 | 2 | 0.00 | 1.00 |
| Chr1:75649191-75649213 | 75649198 | 5 | DNA | GGCCGAGGTCGACTACCG-GCNRG | GGtCGAGGTCGtCTgCaGTGgTGG | - | 5 | 1 | 2 | 0.00 | 1.00 |
| Chr7:115060114-115060136 | 115060131 | 3 | RNA | GGCCGAGGTCGACTACCGGCNRG | GcagGAaGTCGAt--CCGGCGAG | + | 5 | 2 | 2 | 0.00 | 1.00 |
| Chr7:9117462-9117484 | 9117469 | 5 | RNA | GGCCGAGGTCGACTACCGGCNRG | GGC-GcGGaCaACgACCGcCCGG | - | 5 | 1 | 2 | 0.00 | 1.00 |
| Chr1:79935085-79935107 | 79935092 | 5 | RNA | GGCCGAGGTCGACTACCGGCNRG | GGaCcAGGTCG--TcCCaGtTGG | - | 5 | 2 | 2 | 0.00 | 1.00 |
| Chr7:158525418-158525440 | 158525435 | 3 | RNA | GGCCGAGGTCGACTACCGGCNRG | GaCCG--GcCaACTcCtGGCCGG | + | 5 | 2 | 2 | 0.00 | 1.00 |
| Chr1:174887175-174887197 | 174887192 | 3 | RNA | GGCCGAGGTCGACTACCGGCNRG | aG--GAGGTCcACacCaGGCAGG | + | 5 | 2 | 2 | 0.00 | 1.00 |
| Chr8:19981006-19981028 | 19981023 | 3 | RNA | GGCCGAGGTCGACTACCGGCNRG | GGg-tAGGcCGAgcACCGGCCGG | + | 5 | 1 | 2 | 0.00 | 1.00 |
| Chr5:78952372-78952394 | 78952389 | 3 | RNA | GGCCGAGGTCGACTACCGGCNRG | GGgCG-GGcCaACctCCGGCCGG | + | 5 | 1 | 2 | 0.00 | 1.00 |
| Chr1:251915702-251915724 | 251915709 | 5 | RNA | GGCCGAGGTCGACTACCGGCNRG | tGCCGA--TacACcgCCGGCCGG | - | 5 | 2 | 2 | 0.00 | 1.00 |
| Chr5:5491921-5491943 | 5491928 | 5 | RNA | GGCCGAGGTCGACTACCGGCNRG | GaCCGAGG-CGACgACaaGaAGG | - | 5 | 1 | 2 | 0.00 | 1.00 |
| Chr1:227453116-227453138 | 227453133 | 3 | RNA | GGCCGAGGTCGACTACCGGCNRG | GGCCtc--TCGtCTctCGGCCAG | + | 5 | 2 | 2 | 0.00 | 1.00 |
| Chr6:130803392-130803414 | 130803409 | 3 | RNA | GGCCGAGGTCGACTACCGGCNRG | GGCCc--GcCaACTtCCGGgCAG | + | 5 | 2 | 2 | 0.00 | 1.00 |
| Chr6:125785691-125785713 | 125785708 | 3 | RNA | GGCCGAGGTCGACTACCGGCNRG | GGCC-AtGTCGtCcACtGGCGGG | + | 4 | 1 | 2 | 0.00 | 1.00 |
| Chr7:123720490-123720512 | 123720507 | 3 | RNA | GGCCGAGGTCGACTACCGGCNRG | GcCtcgGGTCGACgAC-GGCGGG | + | 5 | 1 | 2 | 0.00 | 1.00 |
| Chr5:204222342-204222364 | 204222349 | 5 | RNA | GGCCGAGGTCGACTACCGGCNRG | GtCCtcGG--GAtaACCGGCAGG | - | 5 | 2 | 2 | 0.00 | 1.00 |
| Chr1:58198497-58198519 | 58198504 | 5 | DNA | GGCCGAGGTCGACTACCG--GCNRG | GGCgcAGGTCGgCgACCGAAtCGAG | - | 5 | 2 | 2 | 0.00 | 1.00 |
| Chr3:125996440-125996462 | 125996457 | 3 | RNA | GGCCGAGGTCGACTACCGGCNRG | GGaCcAGGTCG--TcCCaGtTGG | + | 5 | 2 | 2 | 0.00 | 1.00 |
| Chr1:24117810-24117832 | 24117817 | 5 | RNA | GGCCGAGGTCGACTACCGGCNRG | GGtCGA--TtGAtgcCCGGCCGG | - | 5 | 2 | 2 | 0.00 | 1.00 |
| Chr4:221793263-221793285 | 221793270 | 5 | RNA | GGCCGAGGTCGACTACCGGCNRG | GtCCG--GTgcACcACCGGaTAG | - | 5 | 2 | 2 | 0.00 | 1.00 |
| Chr4:234088684-234088706 | 234088701 | 3 | RNA | GGCCGAGGTCGACTACCGGCNRG | cGCCaAcGTCGgC--CCcGCCAG | + | 5 | 2 | 2 | 0.00 | 1.00 |
| Chr10:6006147-6006169 | 6006154 | 5 | RNA | GGCCGAGGTCGACTACCGGCNRG | GagCGtGGcCGAtT--CGGCGAG | - | 5 | 2 | 2 | 0.00 | 1.00 |
| Chr4:233088295-233088317 | 233088302 | 5 | RNA | GGCCGAGGTCGACTACCGGCNRG | a-CCGcGGTCGACcACCcGCCGG | - | 4 | 1 | 2 | 0.00 | 1.00 |
| Chr2:161210380-161210402 | 161210397 | 3 | RNA | GGCCGAGGTCGACTACCGGCNRG | t--CGAGGaCatCTtCCGGCTGG | + | 5 | 2 | 2 | 0.00 | 1.00 |
| Chr10:119832362-119832384 | 119832379 | 3 | RNA | GGCCGAGGTCGACTACCGGCNRG | GcCgGcGGTCGACT-CCGtCCGG | + | 4 | 1 | 2 | 0.00 | 1.00 |
| Chr10:39633642-39633664 | 39633659 | 3 | RNA | GGCCGAGGTCGACTACCGGCNRG | caaCG--GTCGACTACCacCGGG | + | 5 | 2 | 2 | 0.00 | 1.00 |
| Chr10:120975499-120975521 | 120975506 | 5 | RNA | GGCCGAGGTCGACTACCGGCNRG | GtCCtcGG--GAtaACCGGCAGG | - | 5 | 2 | 2 | 0.00 | 1.00 |
| Chr5:137632765-137632787 | 137632772 | 5 | DNA | GGCCGAGGTCGACTACCGG--CNRG | GGtCGAGGTCGAgTcCgaGCCCCGG | - | 5 | 2 | 2 | 0.00 | 1.00 |
| Chr2:213131578-213131600 | 213131585 | 5 | RNA | GGCCGAGGTCGACTACCGGCNRG | GGaCGAcGgCGACTt--GGCAAG | - | 4 | 2 | 2 | 0.00 | 1.00 |
| Chr10:150008745-150008767 | 150008762 | 3 | DNA | GGCCGAGGTCGACT--ACCGGCNRG | GaCtGtGGTCGgCTGCACCGGgGGG | + | 5 | 2 | 2 | 0.00 | 1.00 |
| Chr7:157084213-157084235 | 157084230 | 3 | DNA | GGC--CGAGGTCGACTACCGGCNRG | GGCCTCGgGGgCtACacCCGGCGGG | + | 5 | 2 | 2 | 0.00 | 1.00 |
| Chr9:129870864-129870886 | 129870881 | 3 | RNA | GGCCGAGGTCGACTACCGGCNRG | GGC--AGGgCGACcAagGcCAAG | + | 5 | 2 | 2 | 0.00 | 1.00 |
| Chr1:248328296-248328318 | 248328303 | 5 | RNA | GGCCGAGGTCGACTACCGGCNRG | GGCtGAGGgtGcCTtC-GGCAGG | - | 5 | 1 | 2 | 0.00 | 1.00 |
| Chr1:217174659-217174681 | 217174676 | 3 | RNA | GGCCGAGGTCGACTACCGGCNRG | GGttaAGaTC-ACTACCGGCTGG | + | 4 | 1 | 2 | 0.00 | 1.00 |
| Chr3:146234967-146234989 | 146234984 | 3 | RNA | GGCCGAGGTCGACTACCGGCNRG | caCCaAGtTCGcCT-CCGGCCAG | + | 5 | 1 | 2 | 0.00 | 1.00 |
| Chr5:20792377-20792399 | 20792394 | 3 | RNA | GGCCGAGGTCGACTACCGGCNRG | cG--GAGtTCGACcACCGGtGAG | + | 4 | 2 | 2 | 0.00 | 1.00 |
| Chr10:129515239-129515261 | 129515246 | 5 | RNA | GGCCGAGGTCGACTACCGGCNRG | GGCgGAG--CcAagACCGGtAAG | - | 5 | 2 | 2 | 0.00 | 1.00 |
| Chr4:134692008-134692030 | 134692015 | 5 | RNA | GGCCGAGGTCGACTACCGGCNRG | aGggGAGaTgGACTAC-GGCTAG | - | 5 | 1 | 2 | 0.00 | 1.00 |
| Chr3:231653432-231653454 | 231653439 | 5 | RNA | GGCCGAGGTCGACTACCGGCNRG | GcCgGAGccCGAC--CCGtCAAG | - | 5 | 2 | 2 | 0.00 | 1.00 |
| Chr6:139056990-139057012 | 139056997 | 5 | RNA | GGCCGAGGTCGACTACCGGCNRG | GaCgGAG-TCGACaACgaGCGGG | - | 5 | 1 | 2 | 0.00 | 1.00 |
| Chr2:36345316-36345338 | 36345323 | 5 | RNA | GGCCGAGGTCGACTACCGGCNRG | tGCCGAGGTCG--TcCCtGaTAG | - | 4 | 2 | 2 | 0.00 | 1.00 |
| Chr6:159092141-159092163 | 159092148 | 5 | DNA | GGCCGA-GGTCGACTACCGGCNRG | GGCgGACGGTCcgCgACCGaCCAG | - | 5 | 1 | 2 | 0.00 | 1.00 |
| Chr6:48756286-48756308 | 48756303 | 3 | RNA | GGCCGAGGTCGACTACCGGCNRG | G-aCGgGGTCGACaACgaGCGGG | + | 5 | 1 | 2 | 0.00 | 1.00 |
| Chr1:203648489-203648511 | 203648496 | 5 | RNA | GGCCGAGGTCGACTACCGGCNRG | GGCCG-GGTCGtCcACtGGCGGG | - | 3 | 1 | 2 | 0.00 | 1.00 |
| Chr6:67437711-67437733 | 67437718 | 5 | RNA | GGCCGAGGTCGACTACCGGCNRG | GG--GAaGTCGACTgtCtcCAAG | - | 5 | 2 | 2 | 0.00 | 1.00 |
| Chr3:120021351-120021373 | 120021358 | 5 | RNA | GGCCGAGGTCGACTACCGGCNRG | GtCCG--GTgcACcACCGGaTGG | - | 5 | 2 | 2 | 0.00 | 1.00 |
| Chr8:135492607-135492629 | 135492624 | 3 | RNA | GGCCGAGGTCGACTACCGGCNRG | GGCCaAGGcC--CTgCgtGCGAG | + | 5 | 2 | 2 | 0.00 | 1.00 |
| Chr6:70495846-70495868 | 70495863 | 3 | RNA | GGCCGAGGTCGACTACCGGCNRG | GcCCGAGGcCGA--ACtcGgTGG | + | 5 | 2 | 2 | 0.00 | 1.00 |
| Chr7:77638660-77638682 | 77638677 | 3 | RNA | GGCCGAGGTCGACTACCGGCNRG | GGCtGAtGTCaAgTACt--CAGG | + | 5 | 2 | 2 | 0.00 | 1.00 |
| Chr1:301206840-301206862 | 301206847 | 5 | RNA | GGCCGAGGTCGACTACCGGCNRG | GtttGAGGT--ACgACCGGaGAG | - | 5 | 2 | 2 | 0.00 | 1.00 |
| Chr10:130123197-130123219 | 130123214 | 3 | RNA | GGCCGAGGTCGACTACCGGCNRG | GGCgGtG--CGcCcACCcGCAGG | + | 5 | 2 | 2 | 0.00 | 1.00 |
| Chr9:119632212-119632234 | 119632229 | 3 | RNA | GGCCGAGGTCGACTACCGGCNRG | GGgCG-GGcCGACctCCGGCCGG | + | 4 | 1 | 2 | 0.00 | 1.00 |
| Chr7:169088060-169088082 | 169088077 | 3 | RNA | GGCCGAGGTCGACTACCGGCNRG | G-aCGgGGTCGACgACgaGCGGG | + | 5 | 1 | 2 | 0.00 | 1.00 |
| Chr5:158274315-158274337 | 158274322 | 5 | RNA | GGCCGAGGTCGACTACCGGCNRG | ccCCGgGGTCGA--AaCGcCGAG | - | 5 | 2 | 2 | 0.00 | 1.00 |
| Chr8:164180904-164180926 | 164180921 | 3 | RNA | GGCCGAGGTCGACTACCGGCNRG | atCCcAtGTCGA--ACgGGCAAG | + | 5 | 2 | 2 | 0.00 | 1.00 |
| Chr5:12129976-12129998 | 12129983 | 5 | RNA | GGCCGAGGTCGACTACCGGCNRG | t--CGAGaTCGACcACgcGCGGG | - | 5 | 2 | 2 | 0.00 | 1.00 |
| Chr1:27877816-27877838 | 27877833 | 3 | RNA | GGCCGAGGTCGACTACCGGCNRG | a--CGgGGTCGACgACgaGCGGG | + | 5 | 2 | 2 | 0.00 | 1.00 |
| Chr7:148639167-148639189 | 148639184 | 3 | RNA | GGCCGAGGTCGACTACCGGCNRG | GGC--tcGcgGACTACCGtCTGG | + | 5 | 2 | 2 | 0.00 | 1.00 |
| Chr8:26367980-26368002 | 26367997 | 3 | RNA | GGCCGAGGTCGACTACCGGCNRG | ttCCGAtGTCGAtTA--tGCTGG | + | 5 | 2 | 2 | 0.00 | 1.00 |
| Chr8:152819325-152819347 | 152819342 | 3 | RNA | GGCCGAGGTCGACTACCGGCNRG | GGCtGAGcgCGgCTg--GGCTGG | + | 5 | 2 | 2 | 0.00 | 1.00 |
| Chr1:102734820-102734842 | 102734837 | 3 | RNA | GGCCGAGGTCGACTACCGGCNRG | GtCCG--GTgcACcACCGGaCAG | + | 5 | 2 | 2 | 0.00 | 1.00 |
| Chr9:148787260-148787282 | 148787267 | 5 | DNA | GGCCGAGGTCGACTACCG-GCNRG | GGCCGgcGTCGAgcACCtCGCTGG | - | 5 | 1 | 2 | 0.00 | 1.00 |
| Chr9:136164941-136164963 | 136164948 | 5 | RNA | GGCCGAGGTCGACTACCGGCNRG | GGCCatGG-CGcCgtCCGGCCGG | - | 5 | 1 | 2 | 0.00 | 1.00 |
| Chr6:46578926-46578948 | 46578933 | 5 | RNA | GGCCGAGGTCGACTACCGGCNRG | tGgCtAGGTCGtCgA--GGCAGG | - | 5 | 2 | 2 | 0.00 | 1.00 |
| Chr2:166012334-166012356 | 166012351 | 3 | RNA | GGCCGAGGTCGACTACCGGCNRG | GG--GAGGagGtaTACgGGCAAG | + | 5 | 2 | 2 | 0.00 | 1.00 |
| Chr2:61178642-61178664 | 61178649 | 5 | RNA | GGCCGAGGTCGACTACCGGCNRG | aGCCGgGGaCGAg--CCGaCGAG | - | 5 | 2 | 2 | 0.00 | 1.00 |
| Chr6:156689741-156689763 | 156689748 | 5 | RNA | GGCCGAGGTCGACTACCGGCNRG | GGCCc--GTtGAggtCCGGCTGG | - | 5 | 2 | 2 | 0.00 | 1.00 |
| Chr6:157449386-157449408 | 157449393 | 5 | RNA | GGCCGAGGTCGACTACCGGCNRG | GtCCG-GGTCGACcACCGGCAGG | - | 2 | 1 | 2 | 0.00 | 1.00 |
| Chr2:119055300-119055322 | 119055307 | 5 | RNA | GGCCGAGGTCGACTACCGGCNRG | aGCCGAGGgCGcCT--CGGgCAG | - | 4 | 2 | 2 | 0.00 | 1.00 |
| Chr6:2318479-2318501 | 2318486 | 5 | RNA | GGCCGAGGTCGACTACCGGCNRG | GGC--ttGcgGACTACCGGCTGG | - | 4 | 2 | 2 | 0.00 | 1.00 |
| Chr1:212138712-212138734 | 212138719 | 5 | RNA | GGCCGAGGTCGACTACCGGCNRG | GGCCG-GGTCGtCcACtGGCGGG | - | 3 | 1 | 2 | 0.00 | 1.00 |
| Chr4:65547281-65547303 | 65547288 | 5 | RNA | GGCCGAGGTCGACTACCGGCNRG | GGaCcAGGTCG--TcCCaGtTGG | - | 5 | 2 | 2 | 0.00 | 1.00 |
| Chr8:120328231-120328253 | 120328248 | 3 | DNA | G-GCCGAGGTCGACTACCGGCNRG | GCGCCGgcGTCGACTcCCGcCGGG | + | 4 | 1 | 2 | 0.00 | 1.00 |
| Chr8:172420228-172420250 | 172420245 | 3 | RNA | GGCCGAGGTCGACTACCGGCNRG | GGCCGgaGcC--CTACCcGtCAG | + | 5 | 2 | 2 | 0.00 | 1.00 |
| Chr2:103165038-103165060 | 103165045 | 5 | RNA | GGCCGAGGTCGACTACCGGCNRG | GGagGAGGTgG--TAgaGGCTAG | - | 5 | 2 | 2 | 0.00 | 1.00 |
| Chr2:104196829-104196851 | 104196846 | 3 | RNA | GGCCGAGGTCGACTACCGGCNRG | GGaCGAGGcgcACaAC--GCTAG | + | 5 | 2 | 2 | 0.00 | 1.00 |
| Chr9:138983243-138983265 | 138983250 | 5 | RNA | GGCCGAGGTCGACTACCGGCNRG | GGaCGAcccCGACT-CCGGtTGG | - | 5 | 1 | 2 | 0.00 | 1.00 |
| Chr1:148312942-148312964 | 148312959 | 3 | RNA | GGCCGAGGTCGACTACCGGCNRG | GGCaGAGG--GgCTcCtcGCCGG | + | 5 | 2 | 2 | 0.00 | 1.00 |
| Chr7:177801759-177801781 | 177801766 | 5 | RNA | GGCCGAGGTCGACTACCGGCNRG | GatCGAGcaCG--TACCaGCTGG | - | 5 | 2 | 2 | 0.00 | 1.00 |
| Chr4:146949056-146949078 | 146949073 | 3 | RNA | GGCCGAGGTCGACTACCGGCNRG | GcaCGccGTCG-CTgCCGGCCGG | + | 5 | 1 | 2 | 0.00 | 1.00 |
| Chr2:58318422-58318444 | 58318429 | 5 | RNA | GGCCGAGGTCGACTACCGGCNRG | GGa-GAGGTtGtCTgCtGGCCAG | - | 5 | 1 | 2 | 0.00 | 1.00 |
| Chr8:91881872-91881894 | 91881879 | 5 | RNA | GGCCGAGGTCGACTACCGGCNRG | GtCCGAcGaCGACT-CCaaCGGG | - | 5 | 1 | 2 | 0.00 | 1.00 |
| Chr5:29957185-29957207 | 29957192 | 5 | RNA | GGCCGAGGTCGACTACCGGCNRG | GtCCG--GTgcACcACCGGaTAG | - | 5 | 2 | 2 | 0.00 | 1.00 |
| Chr10:103294100-103294122 | 103294117 | 3 | RNA | GGCCGAGGTCGACTACCGGCNRG | GGtCGtcGTCG--TACCaaCCGG | + | 5 | 2 | 2 | 0.00 | 1.00 |
| Chr5:123241243-123241265 | 123241260 | 3 | RNA | GGCCGAGGTCGACTACCGGCNRG | GGCCGAG--CcAaTgCtGGtGAG | + | 5 | 2 | 2 | 0.00 | 1.00 |
| Chr5:116131541-116131563 | 116131558 | 3 | DNA | GGCCGAGGTCG-ACTACCGGCNRG | GGCgGcGGTgGCtCTAaCGGCGAG | + | 5 | 1 | 2 | 0.00 | 1.00 |
| Chr1:165772361-165772383 | 165772378 | 3 | DNA | GGC-CGAGGTCGACTACCGGCNRG | GcCTCGgGGaCGtCTtCCGGCTGG | + | 5 | 1 | 2 | 0.00 | 1.00 |
| Chr5:167857191-167857213 | 167857198 | 5 | RNA | GGCCGAGGTCGACTACCGGCNRG | GGCCGtGGTC--CgggCGGgAGG | - | 5 | 2 | 2 | 0.00 | 1.00 |
| Chr3:174368949-174368971 | 174368956 | 5 | DNA | GGCCGAGGTCGAC-TACCGGCNRG | GGCCGAGcTCGgCATgggGGCAGG | - | 5 | 1 | 2 | 0.00 | 1.00 |
| Chr7:41034208-41034230 | 41034215 | 5 | RNA | GGCCGAGGTCGACTACCGGCNRG | GGCCGAGGatGcCT-CgGGCTAG | - | 4 | 1 | 2 | 0.00 | 1.00 |
| Chr4:171838956-171838978 | 171838963 | 5 | X | GGCCGAGGTCGACTACCGGCNRG | GGCCGtGGcCGAgTAgaGGCGGG | - | 5 | 0 | 2 | 0.00 | 1.00 |
| Chr4:142088464-142088486 | 142088481 | 3 | RNA | GGCCGAGGTCGACTACCGGCNRG | GGCgacGcaCGACTACC--CGAG | + | 5 | 2 | 2 | 0.00 | 1.00 |
| Chr8:81559583-81559605 | 81559590 | 5 | RNA | GGCCGAGGTCGACTACCGGCNRG | GGtCGgaaTCGtCT--CGGCAGG | - | 5 | 2 | 2 | 0.00 | 1.00 |
| Chr10:149625639-149625661 | 149625646 | 5 | RNA | GGCCGAGGTCGACTACCGGCNRG | cGCtGAGGTCGAgcA--GGaGAG | - | 5 | 2 | 2 | 0.00 | 1.00 |
| Chr10:123319772-123319794 | 123319779 | 5 | RNA | GGCCGAGGTCGACTACCGGCNRG | GGCaGAGGgCG--cACCGGaCAG | - | 4 | 2 | 2 | 0.00 | 1.00 |
| Chr4:13670781-13670803 | 13670798 | 3 | RNA | GGCCGAGGTCGACTACCGGCNRG | GGC--AGGcCGAgcACCGGCCAG | + | 3 | 2 | 2 | 0.00 | 1.00 |
| Chr10:78120118-78120140 | 78120135 | 3 | RNA | GGCCGAGGTCGACTACCGGCNRG | G-aCGgGGTCGACgACgaGCGGG | + | 5 | 1 | 2 | 0.00 | 1.00 |
| Chr7:4021755-4021777 | 4021762 | 5 | X | GGCCGAGGTCGACTACCGGCNRG | GGCCGAGcTCGAgTtatGGCCGG | - | 5 | 0 | 2 | 0.00 | 1.00 |
| Chr7:93049525-93049547 | 93049532 | 5 | RNA | GGCCGAGGTCGACTACCGGCNRG | G-aCGgGGTCGACaACgaGCGGG | - | 5 | 1 | 2 | 0.00 | 1.00 |
| Chr1:24667508-24667530 | 24667525 | 3 | RNA | GGCCGAGGTCGACTACCGGCNRG | GGCCGAGG-CGggTAgCtcCTGG | + | 5 | 1 | 2 | 0.00 | 1.00 |
| Chr3:80034544-80034566 | 80034551 | 5 | RNA | GGCCGAGGTCGACTACCGGCNRG | GGCaGgGcgCGACT-CCGGtGAG | - | 5 | 1 | 2 | 0.00 | 1.00 |
| Chr7:46462110-46462132 | 46462127 | 3 | X | GGCCGAGGTCGACTACCGGCNRG | GcCCGAGcTgGACTtCgGGCGAG | + | 5 | 0 | 2 | 0.00 | 1.00 |
| Chr8:130883778-130883800 | 130883795 | 3 | RNA | GGCCGAGGTCGACTACCGGCNRG | a--CGgGGTCGACgACgaGCGGG | + | 5 | 2 | 2 | 0.00 | 1.00 |
| Chr1:94436136-94436158 | 94436153 | 3 | RNA | GGCCGAGGTCGACTACCGGCNRG | GGCCGAaGcCGA--AgCtaCCGG | + | 5 | 2 | 2 | 0.00 | 1.00 |
| Chr4:76727200-76727222 | 76727217 | 3 | RNA | GGCCGAGGTCGACTACCGGCNRG | tGCtGAGGTgGAtTACC--tGAG | + | 5 | 2 | 2 | 0.00 | 1.00 |
| Chr6:121585436-121585458 | 121585453 | 3 | RNA | GGCCGAGGTCGACTACCGGCNRG | tGCgGAGc-CGACgACCaGCCGG | + | 5 | 1 | 2 | 0.00 | 1.00 |
| Chr1:288551640-288551662 | 288551657 | 3 | RNA | GGCCGAGGTCGACTACCGGCNRG | GGt-GtGGTCGAggACtGGCGGG | + | 5 | 1 | 2 | 0.00 | 1.00 |
| Chr7:173363549-173363571 | 173363556 | 5 | RNA | GGCCGAGGTCGACTACCGGCNRG | GGtgGcGG-CGgCTACCGGaGGG | - | 5 | 1 | 2 | 0.00 | 1.00 |
| Chr4:147342829-147342851 | 147342846 | 3 | RNA | GGCCGAGGTCGACTACCGGCNRG | aGCCGAGtT-GAacAgCGGCTGG | + | 5 | 1 | 2 | 0.00 | 1.00 |
| Chr5:131149495-131149517 | 131149502 | 5 | RNA | GGCCGAGGTCGACTACCGGCNRG | GGCgGcGGgCGAC-ggCGGCGGG | - | 5 | 1 | 2 | 0.00 | 1.00 |

| | | | | | | | | | | | |
|---|---|---|---|---|---|---|---|---|---|---|---|
| Chr10:129019630-129019652 | 129019647 | 3 | RNA | GGCCGAGGTCGACTACCGGCNRG | G-aCGgGGTCGACaACgaGCGGG | + | 5 | 1 | 2 | 0.00 | 1.00 |
| Chr10:123327514-123327536 | 123327531 | 3 | RNA | GGCCGAGGTCGACTACCGGCNRG | GGCCGAGGTaGACgAa-GtaGAG | + | 5 | 1 | 2 | 0.00 | 1.00 |
| Chr10:57278910-57278932 | 57278917 | 5 | RNA | GGCCGAGGTCGACTACCGGCNRG | GGCCGAGcgCGA--AggGGgGGG | - | 5 | 2 | 2 | 0.00 | 1.00 |
| Chr2:160691875-160691897 | 160691892 | 3 | RNA | GGCCGAGGTCGACTACCGGCNRG | GG--GgGaTCGACccCCaGCCGG | + | 5 | 2 | 2 | 0.00 | 1.00 |
| Chr4:139741790-139741812 | 139741807 | 3 | RNA | GGCCGAGGTCGACTACCGGCNRG | aGaCGAGcTCGACT-CtGGtTGG | + | 5 | 1 | 2 | 0.00 | 1.00 |
| Chr1:141335412-141335434 | 141335419 | 5 | RNA | GGCCGAGGTCGACTACCGGCNRG | GGCCGAGG--GAtggCCcaCGAG | - | 5 | 2 | 2 | 0.00 | 1.00 |
| Chr5:19999870-19999892 | 19999887 | 3 | RNA | GGCCGAGGTCGACTACCGGCNRG | G-aCGgGGTCGACaACgaGCGGG | + | 5 | 1 | 2 | 0.00 | 1.00 |
| Chr5:154323098-154323120 | 154323105 | 5 | RNA | GGCCGAGGTCGACTACCGGCNRG | GGC--ttGcgGACTACaGGCTGG | - | 5 | 2 | 2 | 0.00 | 1.00 |
| Chr2:115844734-115844756 | 115844741 | 5 | RNA | GGCCGAGGTCGACTACCGGCNRG | GGCCaAGcgCG--gACCGtCCGG | - | 5 | 2 | 2 | 0.00 | 1.00 |
| Chr4:165533463-165533485 | 165533470 | 5 | RNA | GGCCGAGGTCGACTACCGGCNRG | GGCgGAGG--GgCagCCGGCCGG | - | 4 | 2 | 2 | 0.00 | 1.00 |
| Chr2:45333383-45333405 | 45333400 | 3 | RNA | GGCCGAGGTCGACTACCGGCNRG | G-aCGAGGTCGACaACgaGCGGG | + | 4 | 1 | 2 | 0.00 | 1.00 |
| Chr1:265313976-265313998 | 265313993 | 3 | RNA | GGCCGAGGTCGACTACCGGCNRG | aGCCGAGtTCcACTAC-GGaGAG | + | 4 | 1 | 2 | 0.00 | 1.00 |
| Chr9:26400028-26400050 | 26400045 | 3 | RNA | GGCCGAGGTCGACTACCGGCNRG | GtCCG--GTgcACcACCGGaTAG | + | 5 | 2 | 2 | 0.00 | 1.00 |
| Chr7:84852488-84852510 | 84852505 | 3 | RNA | GGCCGAGGTCGACTACCGGCNRG | GGCCGAGtaCG--TtgCcGCAGG | + | 5 | 2 | 2 | 0.00 | 1.00 |
| Chr10:94000556-94000578 | 94000573 | 3 | RNA | GGCCGAGGTCGACTACCGGCNRG | GaCgaAGGTgGACTA--GtCAGG | + | 5 | 2 | 2 | 0.00 | 1.00 |
| Chr10:66150444-66150466 | 66150451 | 5 | RNA | GGCCGAGGTCGACTACCGGCNRG | tcCCtAGGTC--CgAtCGGCCAG | - | 5 | 2 | 2 | 0.00 | 1.00 |
| Chr1:117161254-117161276 | 117161261 | 5 | RNA | GGCCGAGGTCGACTACCGGCNRG | GGCCGgG--CcACcAgCGGaAGG | - | 5 | 2 | 2 | 0.00 | 1.00 |
| Chr2:86752278-86752300 | 86752295 | 3 | RNA | GGCCGAGGTCGACTACCGGCNRG | GG--GcGGTCGtggACCGtCGAG | + | 5 | 2 | 2 | 0.00 | 1.00 |
| Chr1:229450438-229450460 | 229450455 | 3 | DNA | GGCCGAGGT--CGACTACCGGCNRG | GGaCGAGGTTCCGACcAagGGCGGG | + | 4 | 2 | 2 | 0.00 | 1.00 |
| Chr3:93391071-93391093 | 93391078 | 5 | RNA | GGCCGAGGTCGACTACCGGCNRG | GaCaG-GGTCGACgACaaGCGGG | - | 5 | 1 | 2 | 0.00 | 1.00 |
| Chr2:138645561-138645583 | 138645578 | 3 | RNA | GGCCGAGGTCGACTACCGGCNRG | GGCgGtGG--GAtcgCCGGCGAG | + | 5 | 2 | 2 | 0.00 | 1.00 |
| Chr2:101562640-101562662 | 101562657 | 3 | RNA | GGCCGAGGTCGACTACCGGCNRG | GGC-GAaGTtGgCTACCtGgAGG | + | 5 | 1 | 2 | 0.00 | 1.00 |
| Chr8:15152296-15152318 | 15152303 | 5 | RNA | GGCCGAGGTCGACTACCGGCNRG | GGaCcAGGTCG--TcCCaGtTGG | - | 5 | 2 | 2 | 0.00 | 1.00 |
| Chr3:178007838-178007860 | 178007855 | 3 | RNA | GGCCGAGGTCGACTACCGGCNRG | GGC-GAGcaCGtagACCGGCCAG | + | 5 | 1 | 2 | 0.00 | 1.00 |
| Chr5:204965973-204965995 | 204965990 | 3 | RNA | GGCCGAGGTCGACTACCGGCNRG | c--CGAGaTaGACTAtCaGCTAG | + | 5 | 2 | 2 | 0.00 | 1.00 |
| Chr2:225889857-225889879 | 225889864 | 5 | RNA | GGCCGAGGTCGACTACCGGCNRG | aGCCatGGcCGACT-CaGGCGGG | - | 5 | 1 | 2 | 0.00 | 1.00 |
| Chr9:142788515-142788537 | 142788522 | 5 | RNA | GGCCGAGGTCGACTACCGGCNRG | GG--GAGaTCGAtctCCGtCGGG | - | 5 | 2 | 2 | 0.00 | 1.00 |
| Chr5:4062455-4062477 | 4062462 | 5 | RNA | GGCCGAGGTCGACTACCGGCNRG | GGCgtA--TCtgCTgCCGGCGGG | - | 5 | 2 | 2 | 0.00 | 1.00 |
| Chr1:55835919-55835941 | 55835926 | 5 | DNA | GGCCGAGG-TCGACTACCGGCNRG | cGCCGAGaCTCGcCTgtCGGCTAG | - | 5 | 1 | 2 | 0.00 | 1.00 |
| Chr2:14263116-14263138 | 14263133 | 3 | RNA | GGCCGAGGTCGACTACCGGCNRG | GtgCcAGGTCG-CTgCCaGCAGG | + | 5 | 1 | 2 | 0.00 | 1.00 |
| Chr5:199934854-199934876 | 199934861 | 5 | RNA | GGCCGAGGTCGACTACCGGCNRG | GtCCG--GTgcACcACCGGaCAG | - | 5 | 2 | 2 | 0.00 | 1.00 |
| Chr1:197047925-197047947 | 197047942 | 3 | RNA | GGCCGAGGTCGACTACCGGCNRG | G-aCGAcGaCGACgACCtGCTGG | + | 5 | 1 | 2 | 0.00 | 1.00 |
| Chr1:32192321-32192343 | 32192338 | 3 | RNA | GGCCGAGGTCGACTACCGGCNRG | GaCCGgGGggGA--ACCGGtCGG | + | 5 | 2 | 2 | 0.00 | 1.00 |
| Chr5:93937940-93937962 | 93937947 | 5 | RNA | GGCCGAGGTCGACTACCGGCNRG | GGCgttGGaCGACTAC--GCCGG | - | 4 | 2 | 2 | 0.00 | 1.00 |
| Chr6:107757665-107757687 | 107757682 | 3 | RNA | GGCCGAGGTCGACTACCGGCNRG | GGCCtAGGcCGA--AattGCTAG | + | 5 | 2 | 2 | 0.00 | 1.00 |
| Chr1:112271048-112271070 | 112271065 | 3 | RNA | GGCCGAGGTCGACTACCGGCNRG | GG--GAGGaCGAgcACCcGtAGG | + | 5 | 2 | 2 | 0.00 | 1.00 |
| Chr2:127387923-127387945 | 127387940 | 3 | RNA | GGCCGAGGTCGACTACCGGCNRG | GGgCGgGGTtGgCT-CCGGgAGG | + | 5 | 1 | 2 | 0.00 | 1.00 |
| Chr9:7940908-7940930 | 7940925 | 3 | RNA | GGCCGAGGTCGACTACCGGCNRG | GGgCGAGcTCGA--ACgGGCTAG | + | 3 | 2 | 2 | 0.00 | 1.00 |
| Chr1:38747461-38747483 | 38747468 | 5 | RNA | GGCCGAGGTCGACTACCGGCNRG | GGCgGAGGTCGAgTAg-GGgCAG | - | 4 | 1 | 2 | 0.00 | 1.00 |
| Chr9:152449405-152449427 | 152449422 | 3 | RNA | GGCCGAGGTCGACTACCGGCNRG | GGCgGAGG--GACagCtGtCGGG | + | 5 | 2 | 2 | 0.00 | 1.00 |
| Chr1:22495432-22495454 | 22495449 | 3 | RNA | GGCCGAGGTCGACTACCGGCNRG | GcCCGAGGgCaACcACC--gTGG | + | 5 | 2 | 2 | 0.00 | 1.00 |
| Chr6:54009023-54009045 | 54009040 | 3 | RNA | GGCCGAGGTCGACTACCGGCNRG | GGCCGgGGTgGA-TctCGcCGAG | + | 5 | 1 | 2 | 0.00 | 1.00 |
| Chr7:137588483-137588505 | 137588490 | 5 | DNA | GGC--CGAGGTCGACTACCGGCNRG | GGCACCGccGTCtAgTAgCGGCTGG | - | 5 | 2 | 2 | 0.00 | 1.00 |
| Chr2:124122825-124122847 | 124122842 | 3 | RNA | GGCCGAGGTCGACTACCGGCNRG | GGt-GAGGTCGACgACatGaTGG | + | 5 | 1 | 2 | 0.00 | 1.00 |
| Chr2:163194446-163194468 | 163194463 | 3 | RNA | GGCCGAGGTCGACTACCGGCNRG | GGCCGAGtaCG--TtgCcGCAAG | + | 5 | 2 | 2 | 0.00 | 1.00 |
| Chr5:13561317-13561339 | 13561334 | 3 | RNA | GGCCGAGGTCGACTACCGGCNRG | G-aCGgGGTCGACgACgaGCGGG | + | 5 | 1 | 2 | 0.00 | 1.00 |
| Chr4:208437914-208437936 | 208437931 | 3 | RNA | GGCCGAGGTCGACTACCGGCNRG | GGatGAGGagGACT--CGGaCGG | + | 5 | 2 | 2 | 0.00 | 1.00 |
| Chr3:232570199-232570221 | 232570216 | 3 | RNA | GGCCGAGGTCGACTACCGGCNRG | GGa-GAGGgaGACgAtCGGCTGG | + | 5 | 1 | 2 | 0.00 | 1.00 |
| Chr4:173354999-173355021 | 173355016 | 3 | RNA | GGCCGAGGTCGACTACCGGCNRG | GtCCG--GTgcACcACCGGaCAG | + | 5 | 2 | 2 | 0.00 | 1.00 |
| Chr5:2760202-2760224 | 2760209 | 5 | RNA | GGCCGAGGTCGACTACCGGCNRG | GGCCGAcagC--CcACCcGCCGG | - | 5 | 2 | 2 | 0.00 | 1.00 |
| Chr3:214825383-214825405 | 214825400 | 3 | RNA | GGCCGAGGTCGACTACCGGCNRG | GGtCG-GGTgcACTcCCGcCAGG | + | 5 | 1 | 2 | 0.00 | 1.00 |
| Chr9:77697514-77697536 | 77697531 | 3 | RNA | GGCCGAGGTCGACTACCGGCNRG | GGgC-AGGcCGAgcACCGGCCGG | + | 4 | 1 | 2 | 0.00 | 1.00 |
| Chr8:95710211-95710233 | 95710218 | 5 | RNA | GGCCGAGGTCGACTACCGGCNRG | G-aCGgGGTCGACaACgaGCGGG | - | 5 | 1 | 2 | 0.00 | 1.00 |
| Chr10:120745055-120745077 | 120745062 | 5 | RNA | GGCCGAGGTCGACTACCGGCNRG | GGC--AGacCGAgctCCGGCCGG | - | 5 | 2 | 2 | 0.00 | 1.00 |
| Chr9:53478402-53478424 | 53478409 | 5 | RNA | GGCCGAGGTCGACTACCGGCNRG | GctCGAG--tGACTcCaGGCGAG | - | 5 | 2 | 2 | 0.00 | 1.00 |
| Chr1:86156470-86156492 | 86156477 | 5 | RNA | GGCCGAGGTCGACTACCGGCNRG | GG--GAGGgCGcCgACgGGaGGG | - | 5 | 2 | 2 | 0.00 | 1.00 |
| Chr7:159036223-159036245 | 159036240 | 3 | RNA | GGCCGAGGTCGACTACCGGCNRG | cGCCaAacTCtAC--CCGGCCGG | + | 5 | 2 | 2 | 0.00 | 1.00 |
| Chr7:72618723-72618745 | 72618730 | 5 | DNA | GGC-CGAGGTCGACTACCGGCNRG | GcCTCGAGGaCGtCTtCCGGCTGG | - | 4 | 1 | 2 | 0.00 | 1.00 |
| Chr1:306256510-306256532 | 306256517 | 5 | DNA | GGCCGAGG-TCGACTACCGGCNRG | GGCtGAaGATCGACTACatGtTGG | - | 5 | 1 | 2 | 0.00 | 1.00 |
| Chr3:195835568-195835590 | 195835585 | 3 | RNA | GGCCGAGGTCGACTACCGGCNRG | GGCgGAaGTCG--TtCgGcCGAG | + | 5 | 2 | 2 | 0.00 | 1.00 |
| Chr7:181053709-181053731 | 181053726 | 3 | RNA | GGCCGAGGTCGACTACCGGCNRG | GGagGAGGTCGACgAC-GagCGG | + | 5 | 1 | 2 | 0.00 | 1.00 |
| Chr1:200694720-200694742 | 200694737 | 3 | RNA | GGCCGAGGTCGACTACCGGCNRG | GtCCG--GaaGAaTACCGGaAAG | + | 5 | 2 | 2 | 0.00 | 1.00 |
| Chr1:10504032-10504054 | 10504049 | 3 | X | GGCCGAGGTCGACTACCGGCNRG | GGCCGAGaTCccCTggCGGCGGG | + | 5 | 0 | 2 | 0.00 | 1.00 |
| Chr5:172702656-172702678 | 172702663 | 5 | RNA | GGCCGAGGTCGACTACCGGCNRG | caCCaAGtTCGcCT-CCGGCCAG | - | 5 | 1 | 2 | 0.00 | 1.00 |
| Chr2:64885770-64885792 | 64885777 | 5 | RNA | GGCCGAGGTCGACTACCGGCNRG | cGCCGAcGT-GACcgCCGaCAAG | - | 5 | 1 | 2 | 0.00 | 1.00 |
| Chr7:68005428-68005450 | 68005445 | 3 | RNA | GGCCGAGGTCGACTACCGGCNRG | GcCCGAGGgCaACcACC--aTGG | + | 5 | 2 | 2 | 0.00 | 1.00 |
| Chr1:266544125-266544147 | 266544132 | 5 | RNA | GGCCGAGGTCGACTACCGGCNRG | GGC-aAGGTgGcaTACCGGCTGG | - | 4 | 1 | 2 | 0.00 | 1.00 |
| Chr4:228259976-228259998 | 228259993 | 3 | RNA | GGCCGAGGTCGACTACCGGCNRG | tGgCGAGGTCGAggACC--tTGG | + | 5 | 2 | 2 | 0.00 | 1.00 |
| Chr8:106375413-106375435 | 106375430 | 3 | RNA | GGCCGAGGTCGACTACCGGCNRG | c--CGcGGcCGAacACCGGCAAG | + | 5 | 2 | 2 | 0.00 | 1.00 |
| Chr1:151542562-151542584 | 151542579 | 3 | RNA | GGCCGAGGTCGACTACCGGCNRG | GGCCt--GcCaACTtCCGGgCAG | + | 5 | 2 | 2 | 0.00 | 1.00 |
| Chr2:17800002-17800024 | 17800009 | 5 | RNA | GGCCGAGGTCGACTACCGGCNRG | GtCCG--GTgcACcACCGGaCAG | - | 5 | 2 | 2 | 0.00 | 1.00 |
| Chr5:93820288-93820310 | 93820305 | 3 | DNA | G--GCCGAGGTCGACTACCGGCNRG | GTAGCCGAGGcCGACctCaGGCGAG | + | 4 | 2 | 2 | 0.00 | 1.00 |
| Chr8:146627299-146627321 | 146627306 | 5 | DNA | GGC--CGAGGTCGACTACCGGCNRG | cGCTTCGgGGaCGtCTtCCGGCTGG | - | 5 | 2 | 2 | 0.00 | 1.00 |
| Chr4:228197456-228197478 | 228197463 | 5 | RNA | GGCCGAGGTCGACTACCGGCNRG | GGCtcAcG--GACTACCtGCTGG | - | 4 | 2 | 2 | 0.00 | 1.00 |
| Chr2:152700927-152700949 | 152700944 | 3 | RNA | GGCCGAGGTCGACTACCGGCNRG | GaCCGAGG-CGACgACaaGaAGG | + | 5 | 1 | 2 | 0.00 | 1.00 |
| Chr1:251528727-251528749 | 251528734 | 5 | RNA | GGCCGAGGTCGACTACCGGCNRG | GaCCG-GGTCGACaACgaGCGGG | - | 4 | 1 | 2 | 0.00 | 1.00 |
| Chr3:86293443-86293465 | 86293450 | 5 | RNA | GGCCGAGGTCGACTACCGGCNRG | GGaCGAGGTCG--ggatGGCCGG | - | 5 | 2 | 2 | 0.00 | 1.00 |
| Chr4:82435430-82435452 | 82435447 | 3 | RNA | GGCCGAGGTCGACTACCGGCNRG | tGCaGgGcTCGgCTA--GGCTGG | + | 5 | 2 | 2 | 0.00 | 1.00 |
| Chr9:88565741-88565763 | 88565748 | 5 | X | GGCCGAGGTCGACTACCGGCNRG | GaCCGAGGggGAaTACCcGCTGG | - | 5 | 0 | 2 | 0.00 | 1.00 |
| Chr2:193040511-193040533 | 193040518 | 5 | RNA | GGCCGAGGTCGACTACCGGCNRG | GGCCGAGGcCGAgccCC--tGGG | - | 5 | 2 | 2 | 0.00 | 1.00 |
| Chr8:62989458-62989480 | 62989475 | 3 | RNA | GGCCGAGGTCGACTACCGGCNRG | cGgtGAGcTtG--TACCGGCCGG | + | 5 | 2 | 2 | 0.00 | 1.00 |
| Chr6:18764727-18764749 | 18764734 | 5 | RNA | GGCCGAGGTCGACTACCGGCNRG | GtCCG--GTgGcCcACCGGaTAG | - | 5 | 2 | 2 | 0.00 | 1.00 |
| Chr8:4558797-4558819 | 4558804 | 5 | RNA | GGCCGAGGTCGACTACCGGCNRG | GaCC-AGGTCttCTcCCaGCAGG | - | 5 | 1 | 2 | 0.00 | 1.00 |
| Chr1:82134215-82134237 | 82134222 | 5 | RNA | GGCCGAGGTCGACTACCGGCNRG | GGC--tcGcgGACTACCGGCTGG | - | 4 | 2 | 2 | 0.00 | 1.00 |
| Chr7:125319594-125319616 | 125319601 | 5 | RNA | GGCCGAGGTCGACTACCGGCNRG | GcaCGAGGgCGACTt--GGCAAG | - | 4 | 2 | 2 | 0.00 | 1.00 |
| Chr4:99787853-99787875 | 99787870 | 3 | RNA | GGCCGAGGTCGACTACCGGCNRG | GGCCGAGGgC-ACctCgGGCTGG | + | 4 | 1 | 2 | 0.00 | 1.00 |
| Chr1:276024506-276024528 | 276024523 | 3 | RNA | GGCCGAGGTCGACTACCGGCNRG | tGgCtgcG--GACTACCGGCTGG | + | 5 | 2 | 2 | 0.00 | 1.00 |
| Chr3:30024630-30024652 | 30024637 | 5 | X | GGCCGAGGTCGACTACCGGCNRG | GGCCGAGGcCGAgctCgGGCGAG | - | 5 | 0 | 2 | 0.00 | 1.00 |
| Chr4:127812308-127812330 | 127812315 | 5 | RNA | GGCCGAGGTCGACTACCGGCNRG | GGCaGAGGgtG--cACCGGaCAG | - | 5 | 2 | 2 | 0.00 | 1.00 |
| Chr3:84602825-84602847 | 84602842 | 3 | RNA | GGCCGAGGTCGACTACCGGCNRG | GGgCGAGcTCGtC-ggCGGCGAG | + | 5 | 1 | 2 | 0.00 | 1.00 |
| Chr8:5994399-5994421 | 5994416 | 3 | RNA | GGCCGAGGTCGACTACCGGCNRG | G-aCGgGGTCGACaACgaGCGGG | + | 5 | 1 | 2 | 0.00 | 1.00 |
| Chr4:144602546-144602568 | 144602563 | 3 | X | GGCCGAGGTCGACTACCGGCNRG | GGCtGAGacCGACTtCgGGCGAG | + | 5 | 0 | 2 | 0.00 | 1.00 |
| Chr2:59678565-59678587 | 59678582 | 3 | RNA | GGCCGAGGTCGACTACCGGCNRG | GGC--ttGcgGACTACCGGCTGG | + | 4 | 2 | 2 | 0.00 | 1.00 |
| Chr8:63187388-63187410 | 63187405 | 3 | DNA | GGCCGAGGTCGACTA--CCGGCNRG | GGgCGAGtcgGACaACTCCGGCGAG | + | 5 | 2 | 2 | 0.00 | 1.00 |
| Chr7:33714278-33714300 | 33714285 | 5 | DNA | GGC-CGAGGTCGACTACCGGCNRG | aGCTCGAatTCtACcACCGGCTGG | - | 5 | 1 | 2 | 0.00 | 1.00 |
| Chr3:185874988-185875010 | 185874995 | 5 | RNA | GGCCGAGGTCGACTACCGGCNRG | GtCCG--GTgcACcACCGGaCAG | - | 5 | 2 | 2 | 0.00 | 1.00 |
| Chr5:124273353-124273375 | 124273370 | 3 | DNA | GGCCGAGGTCGACTACC--GGCNRG | cGaCGAGGgCGACgACaGTGGCAAG | + | 5 | 2 | 2 | 0.00 | 1.00 |
| Chr2:242848948-242848970 | 242848965 | 3 | RNA | GGCCGAGGTCGACTACCGGCNRG | GGC--ttGTgGAtTACCGGCTGG | + | 4 | 2 | 2 | 0.00 | 1.00 |
| Chr2:89511218-89511240 | 89511235 | 3 | RNA | GGCCGAGGTCGACTACCGGCNRG | tGCCGAtGgCGctT--CGGCCAG | + | 5 | 2 | 2 | 0.00 | 1.00 |
| Chr4:167507325-167507347 | 167507342 | 3 | RNA | GGCCGAGGTCGACTACCGGCNRG | GGgaGAGaTCaACT-CgGGCAAG | + | 5 | 1 | 2 | 0.00 | 1.00 |
| Chr9:153190486-153190508 | 153190503 | 3 | DNA | GGCCGAGGTCGACTACCGG--CNRG | GGCCGAGGcCGAgTcCgaGCCCTGG | + | 5 | 2 | 2 | 0.00 | 1.00 |
| Chr4:123629510-123629532 | 123629527 | 3 | DNA | GGCCGAGGTCGACTACC--GGCNRG | GcCCGAGGgCaACcACCGTGGCCAG | + | 4 | 2 | 2 | 0.00 | 1.00 |
| Chr1:216585878-216585900 | 216585885 | 5 | RNA | GGCCGAGGTCGACTACCGGCNRG | GGC--AGGctGAgcACCGGCCGG | - | 4 | 2 | 2 | 0.00 | 1.00 |
| Chr1:192014236-192014258 | 192014253 | 3 | RNA | GGCCGAGGTCGACTACCGGCNRG | GGaCG--GTCGgCgACgtGCGGG | + | 5 | 2 | 2 | 0.00 | 1.00 |
| Chr4:52822882-52822904 | 52822889 | 5 | RNA | GGCCGAGGTCGACTACCGGCNRG | G--CGAGGgCGcggAgCGGCTGG | - | 5 | 2 | 2 | 0.00 | 1.00 |
| Chr5:15475695-15475717 | 15475712 | 3 | RNA | GGCCGAGGTCGACTACCGGCNRG | aGCCGAGGgtGcCT-CgGGCCGG | + | 5 | 1 | 2 | 0.00 | 1.00 |
| Chr9:113739620-113739642 | 113739627 | 5 | RNA | GGCCGAGGTCGACTACCGGCNRG | GtCCG--GaaGAgTACCGGaAAG | - | 5 | 2 | 2 | 0.00 | 1.00 |
| Chr10:58567884-58567906 | 58567891 | 5 | RNA | GGCCGAGGTCGACTACCGGCNRG | GGC--AGGcCGAgcgCCGGCCGG | - | 4 | 2 | 2 | 0.00 | 1.00 |
| Chr7:21748109-21748131 | 21748116 | 5 | RNA | GGCCGAGGTCGACTACCGGCNRG | GGCCaAGGTCa--TcggGGCAGG | - | 5 | 2 | 2 | 0.00 | 1.00 |
| Chr1:160337997-160338019 | 160338004 | 5 | RNA | GGCCGAGGTCGACTACCGGCNRG | acCCaAGGTCGA--AaCGGaCGG | - | 5 | 2 | 2 | 0.00 | 1.00 |
| Chr3:151701046-151701068 | 151701053 | 5 | X | GGCCGAGGTCGACTACCGGCNRG | GGCCGAGGTCGAgctCgaGCGAG | - | 5 | 0 | 2 | 0.00 | 1.00 |
| Chr8:162144561-162144583 | 162144578 | 3 | RNA | GGCCGAGGTCGACTACCGGCNRG | aGaCGAtGTCGACa--CaGCAAG | + | 5 | 2 | 2 | 0.00 | 1.00 |
| Chr7:106843587-106843609 | 106843594 | 5 | RNA | GGCCGAGGTCGACTACCGGCNRG | GGCgaActTCGgCTAC--GCGGG | - | 5 | 2 | 2 | 0.00 | 1.00 |
| Chr4:139754924-139754946 | 139754941 | 3 | RNA | GGCCGAGGTCGACTACCGGCNRG | GGCCaAcG--GgCTACtGGgTGG | + | 5 | 2 | 2 | 0.00 | 1.00 |
| Chr3:4244332-4244354 | 4244339 | 5 | RNA | GGCCGAGGTCGACTACCGGCNRG | G-CCtcGGgCGAgTACCaGCAAG | - | 5 | 1 | 2 | 0.00 | 1.00 |
| Chr5:35326229-35326251 | 35326236 | 5 | DNA | GGCCGAGGTCGACTA--CCGGCNRG | ctCCtAGGcCGACcAATCCGGCAGG | - | 5 | 2 | 2 | 0.00 | 1.00 |
| Chr4:165114006-165114028 | 165114013 | 5 | RNA | GGCCGAGGTCGACTACCGGCNRG | GGCgaAGcTCatCT--CGGCAAG | - | 5 | 2 | 2 | 0.00 | 1.00 |
| Chr1:266477269-266477291 | 266477286 | 3 | DNA | GGCCGAGGTCGACTACCGG--CNRG | GGCCGAGGTCGAgTcCgaGCCCTGG | + | 4 | 2 | 2 | 0.00 | 1.00 |
| Chr10:120789913-120789935 | 120789920 | 5 | RNA | GGCCGAGGTCGACTACCGGCNRG | GGCgGAGGTCG--TtCgGcaGAG | - | 5 | 2 | 2 | 0.00 | 1.00 |
| Chr5:117396773-117396795 | 117396790 | 3 | RNA | GGCCGAGGTCGACTACCGGCNRG | GGC--AGGcCaAgctCCGGCCGG | + | 5 | 2 | 2 | 0.00 | 1.00 |
| Chr8:162200504-162200526 | 162200511 | 5 | RNA | GGCCGAGGTCGACTACCGGCNRG | GGC--tcGcgGACTACCGcCTAG | - | 5 | 2 | 2 | 0.00 | 1.00 |
| Chr8:81361636-81361658 | 81361653 | 3 | RNA | GGCCGAGGTCGACTACCGGCNRG | GGC--tcGcaGACTACCGcCTGG | + | 5 | 2 | 2 | 0.00 | 1.00 |
| Chr4:100294488-100294510 | 100294505 | 3 | RNA | GGCCGAGGTCGACTACCGGCNRG | aG--GAGGTCGACTtggcGCTGG | + | 5 | 2 | 2 | 0.00 | 1.00 |
| Chr2:113204267-113204289 | 113204284 | 3 | RNA | GGCCGAGGTCGACTACCGGCNRG | GGgCGAGGgCGgC-gCCGGtCGG | + | 5 | 1 | 2 | 0.00 | 1.00 |
| Chr5:148949111-148949133 | 148949118 | 5 | RNA | GGCCGAGGTCGACTACCGGCNRG | GGa--AGGcCGAtgACaGGCGGG | - | 5 | 2 | 2 | 0.00 | 1.00 |
| Chr5:170139479-170139501 | 170139486 | 5 | DNA | GGCCGAGGTCGACTA--CCGGCNRG | ctCCtAGGcCGACcAATCCGGCAGG | - | 5 | 2 | 2 | 0.00 | 1.00 |

| | | | | | | | | | | | |
|---|---|---|---|---|---|---|---|---|---|---|---|
| Chr1:121846086-121846108 | 121846093 | 5 | RNA | GGCCGAGGTCGACTACCGGCNRG | GGCtGAGaTt-AgaACCGGCGGG | - | 5 | 1 | 2 | 0.00 | 1.00 |
| Chr7:156838989-156839011 | 156839006 | 3 | RNA | GGCCGAGGTCGACTACCGGCNRG | caCCaAGtTCGcCT-CCGGCCAG | + | 5 | 1 | 2 | 0.00 | 1.00 |
| Chr8:15436603-15436625 | 15436620 | 3 | RNA | GGCCGAGGTCGACTACCGGCNRG | GGgCG-GGcCtACctCCGGCCGG | + | 5 | 1 | 2 | 0.00 | 1.00 |
| Chr7:88111977-88111999 | 88111994 | 3 | DNA | GGCCGAGGTCGA-CTACCGGCNRG | aGCCGAGGcCGATCgACCGatGAG | + | 5 | 1 | 2 | 0.00 | 1.00 |
| Chr1:106347042-106347064 | 106347049 | 5 | RNA | GGCCGAGGTCGACTACCGGCNRG | GGC--AGGaCGAaTAaaGaCCGG | - | 5 | 2 | 2 | 0.00 | 1.00 |
| Chr7:165223996-165224018 | 165224013 | 3 | RNA | GGCCGAGGTCGACTACCGGCNRG | G-CCGAGGcCGAgctCgGGCGAG | + | 5 | 1 | 2 | 0.00 | 1.00 |
| Chr3:220370964-220370986 | 220370981 | 3 | RNA | GGCCGAGGTCGACTACCGGCNRG | GGC--ttGcgGAtTACCGGCTGG | + | 5 | 2 | 2 | 0.00 | 1.00 |
| Chr8:4996049-4996071 | 4996056 | 5 | RNA | GGCCGAGGTCGACTACCGGCNRG | ccCCGgGGTCGA--AaCGcCGAG | - | 5 | 2 | 2 | 0.00 | 1.00 |
| Chr2:3969609-3969631 | 3969616 | 5 | RNA | GGCCGAGGTCGACTACCGGCNRG | GGCCGAGGgt-ACctCgGGCTGG | - | 5 | 1 | 2 | 0.00 | 1.00 |
| Chr4:176776870-176776892 | 176776877 | 5 | DNA | GGCCGAGGTCGACTACCGG--CNRG | GGtCGAGGTCGAgTcCgaGCCCTGG | - | 5 | 2 | 2 | 0.00 | 1.00 |
| Chr8:79847512-79847534 | 79847519 | 5 | RNA | GGCCGAGGTCGACTACCGGCNRG | GGagGgGaTCGACTA--GGgGAG | - | 5 | 2 | 2 | 0.00 | 1.00 |
| Chr6:137270185-137270207 | 137270202 | 3 | RNA | GGCCGAGGTCGACTACCGGCNRG | aGCCGAGGgtGcCT-CgGGCAGG | + | 5 | 1 | 2 | 0.00 | 1.00 |
| Chr1:65383212-65383234 | 65383229 | 3 | RNA | GGCCGAGGTCGACTACCGGCNRG | cGCCGcGGTCGcCat--GGCAGG | + | 5 | 2 | 2 | 0.00 | 1.00 |
| Chr1:302484041-302484063 | 302484048 | 5 | RNA | GGCCGAGGTCGACTACCGGCNRG | GcCtcAGGTCGAC-ggCGGCGGG | - | 5 | 1 | 2 | 0.00 | 1.00 |
| Chr1:158686361-158686383 | 158686368 | 5 | RNA | GGCCGAGGTCGACTACCGGCNRG | GGgCGgGGTtGgCT-CCGGgAGG | - | 5 | 1 | 2 | 0.00 | 1.00 |
| Chr4:146127556-146127578 | 146127573 | 3 | RNA | GGCCGAGGTCGACTACCGGCNRG | GGCgaAcGT-GACaACCGGtAAG | + | 5 | 1 | 2 | 0.00 | 1.00 |
| Chr8:95686187-95686209 | 95686194 | 5 | RNA | GGCCGAGGTCGACTACCGGCNRG | GGC--tcGcgGACTACCGtCTGG | - | 5 | 2 | 2 | 0.00 | 1.00 |
| Chr6:19132057-19132079 | 19132074 | 3 | RNA | GGCCGAGGTCGACTACCGGCNRG | GcCgGgGGTCGACTt--GGaGGG | + | 5 | 2 | 2 | 0.00 | 1.00 |
| Chr5:59842963-59842985 | 59842980 | 3 | RNA | GGCCGAGGTCGACTACCGGCNRG | G--CGAGGaCaACaACCaGaAGG | + | 5 | 2 | 2 | 0.00 | 1.00 |
| Chr4:109179406-109179428 | 109179423 | 3 | RNA | GGCCGAGGTCGACTACCGGCNRG | GtCCGAGGaC--CTAaCtGCGAG | + | 4 | 2 | 2 | 0.00 | 1.00 |
| Chr7:68498628-68498650 | 68498645 | 3 | DNA | GGCCGAGGTCGACTACC--GGCNRG | GcCaGAGcTCGgCcACCATGGCCAG | + | 5 | 2 | 2 | 0.00 | 1.00 |
| Chr3:157868846-157868868 | 157868863 | 3 | RNA | GGCCGAGGTCGACTACCGGCNRG | aGCCcAGGaCG--TACaaGCTGG | + | 5 | 2 | 2 | 0.00 | 1.00 |
| Chr3:43610460-43610482 | 43610467 | 5 | RNA | GGCCGAGGTCGACTACCGGCNRG | GGC--tcGcaGACTACCGGCTGG | - | 4 | 2 | 2 | 0.00 | 1.00 |
| Chr3:880380-880402 | 880387 | 5 | RNA | GGCCGAGGTCGACTACCGGCNRG | GtCaGAG-TCGACctCgGGCGAG | - | 5 | 1 | 2 | 0.00 | 1.00 |
| Chr7:12856794-12856816 | 12856801 | 5 | RNA | GGCCGAGGTCGACTACCGGCNRG | aGCCGtGGT-GgCatCCGGCTGG | - | 5 | 1 | 2 | 0.00 | 1.00 |
| Chr5:18497644-18497666 | 18497661 | 3 | RNA | GGCCGAGGTCGACTACCGGCNRG | GGC--AGGcCGAgcACCaaCCGG | + | 5 | 2 | 2 | 0.00 | 1.00 |
| Chr8:96805245-96805267 | 96805262 | 3 | RNA | GGCCGAGGTCGACTACCGGCNRG | GaCCGAGGT-GACgACaaGaAGG | + | 5 | 1 | 2 | 0.00 | 1.00 |
| Chr3:207124871-207124893 | 207124888 | 3 | RNA | GGCCGAGGTCGACTACCGGCNRG | GGaCcAGGTCG--TcCCaGtTGG | + | 5 | 2 | 2 | 0.00 | 1.00 |
| Chr2:204542876-204542898 | 204542893 | 3 | RNA | GGCCGAGGTCGACTACCGGCNRG | GGC--tcGcgGACTACCGGCTGG | + | 4 | 2 | 2 | 0.00 | 1.00 |
| Chr4:117792293-117792315 | 117792300 | 5 | RNA | GGCCGAGGTCGACTACCGGCNRG | G-atGgGGTCGACaACgGGCGGG | - | 5 | 1 | 2 | 0.00 | 1.00 |
