# Supplementary material for: CRISPR-Cas9 Editing in Maize: Systematic Evaluation of Off-target Activity and Its Relevance in Crop Improvement
Source: Sci Rep. 2019 Apr 30;9:6729. doi: 10.1038/s41598-019-43141-6 (PMC6491584; doi:10.1038/s41598-019-43141-6)
Supplement: Supplementary file 3 — Supplementary Figure 1, 2 [file 41598_2019_43141_MOESM3_ESM.pdf]

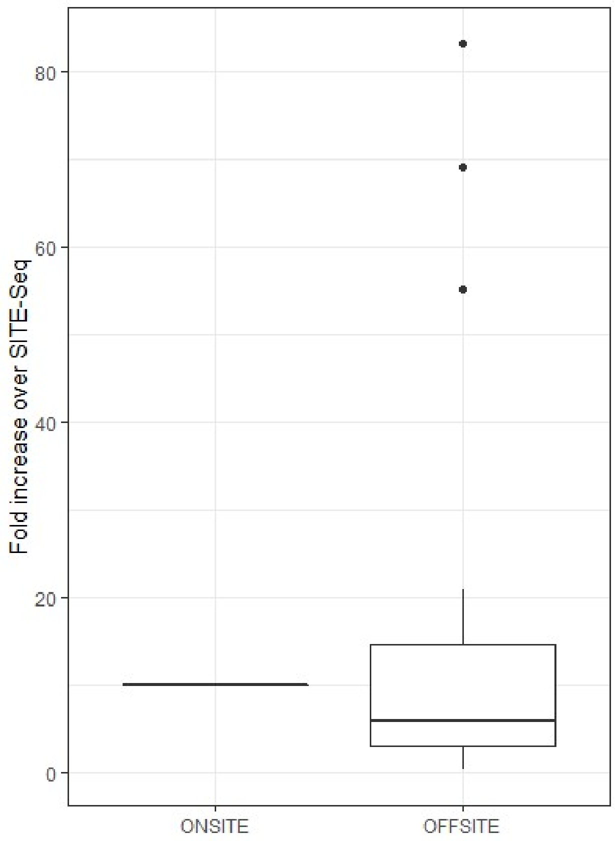

Supplementary Figure 1. Comparison between CLEAVE-Seq and SITE-seq. Box and whisker plot of the fold difference in read coverage between the collection of on- and off-target sites recovered using CLEAVE-Seq and SITE-Seq. The M4 target site, GGCGGCGGCGAGGTAGTGCGAGG (PAM in blue font), located within the liguleless 1 gene (Chr2:4233973-4233995 (AGPv4)) was used in the comparison. Two CLEAVE-Seq technical replicates and one control SITE-Seq replicate were processed, using protocols described here for CLEAVE-Seq (see Materials & Methods), and as previously described for SITE-Seq by Cameron *et al*. 2017[28]. Following library construction, sequencing was performed for all three samples on an Illumina HiSeq2500, and data were analyzed using the CLEAVE-Seq analytical approach described in the Materials & Methods. Results demonstrate that CLEAVE-Seq libraries provide an enhanced recovery at on- and off-sites relative to SITE-Seq, ~10-fold for the on-target and ~7-fold for the off-targets.

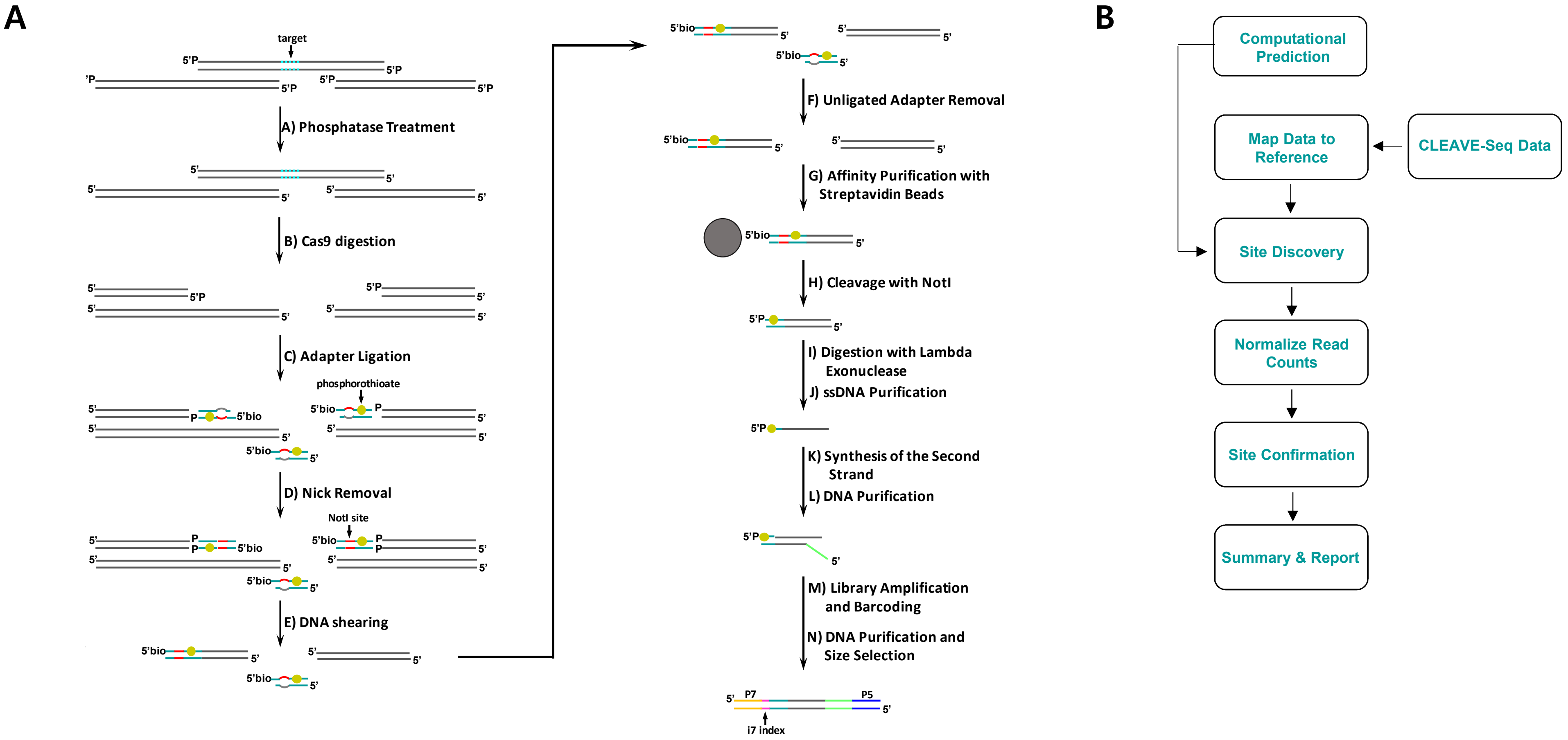

Supplementary Figure 2. A) Schematic of CLEAVE-Seq method. First, purified genomic DNA is dephosphorylated to reduce the number of free genomic DNA ends not generated by Cas9. Then, unphosphorylated adapters conjugated to biotin are used to capture Cas9 cleavage sites. Next, DNA fragment length is reduced and Cas9 cleaved fragments are enriched for by Streptavidin affinity purification. Finally, indexed libraries are generated for deep sequencing. B) Flow chart of target discovery informatics. First, computational prediction (e.g. Cas-OFFinder (Bae, Park et al. 2014) is used to identify putative genomic target sites. Next, CLEAVE-Seq data is combined with computation predictions to identify biochemically cleaved on- and off-target sites. The read count for each site is then normalized based on total read depth and confirmed as being unique to the Cas9 treated sample by comparisons to the control (reactions assembled in the absence of a guide RNA).
