# Supplementary material for: CRISPR-Cas9 Editing in Maize: Systematic Evaluation of Off-target Activity and Its Relevance in Crop Improvement
Source: Sci Rep. 2019 Apr 30;9:6729. doi: 10.1038/s41598-019-43141-6 (PMC6491584; doi:10.1038/s41598-019-43141-6)
Supplement: Supplementary file 4 — SI [file 41598_2019_43141_MOESM4_ESM.pdf]

Supplementary information

CRISPR-Cas9 Editing in Maize: Systematic Evaluation of Off-target Activity and Its Relevance in Crop Improvement

Joshua Young[1†], Gina Zastrow-Hayes[1†], Stéphane Deschamps[1†], Sergei Svitashev[1], Mindaugas Zaremba[2], Ananta Acharya[1], Sushmitha Paulraj[1], Brooke Peterson-Burch[1], Chris Schwartz[1], Vesna Djukanovic[1], Brian Lenderts[1], Lanie Feigenbutz[1], Lijuan Wang[1], Clara Alarcon[1], Virginijus Siksnys[2], Gregory May[1], Doane Chilcoat[1], Sandeep Kumar[1*]

[1]Corteva Agriscience™, Agriculture Division of DowDuPont™, Johnston, IA 50131, USA
[2]Institute of Biotechnology, Vilnius University, Vilnius, LT-10257, Lithuania
*Corresponding author sandeep.kumar@corteva.com
[†]These authors contributed equally to this work

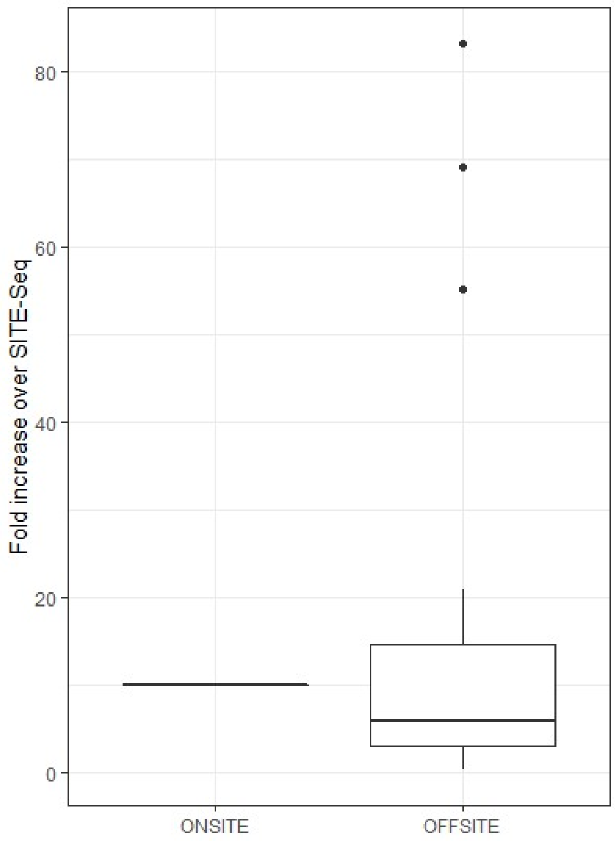

Supplementary Figure 1. Comparison between CLEAVE-Seq and SITE-seq. Box and whisker plot of the fold difference in read coverage between the collection of on- and off-target sites recovered using CLEAVE-Seq and SITE-Seq. The M4 target site, GGCGGCGGCGAGGTAGTGCGAGG (PAM in blue font), located within the liguleless 1 gene (Chr2:4233973-4233995 (AGPv4)) was used in the comparison. Two CLEAVE-Seq technical replicates and one control SITE-Seq replicate were processed, using protocols described here for CLEAVE-Seq (see Materials & Methods), and as previously described for SITE-Seq by Cameron *et al*. 2017[28]. Following library construction, sequencing was performed for all three samples on an Illumina HiSeq2500, and data were analyzed using the CLEAVE-Seq analytical approach described in the Materials & Methods. Results demonstrate that CLEAVE-Seq libraries provide an enhanced recovery at on- and off-sites relative to SITE-Seq, ~10-fold for the on-target and ~7-fold for the off-targets.

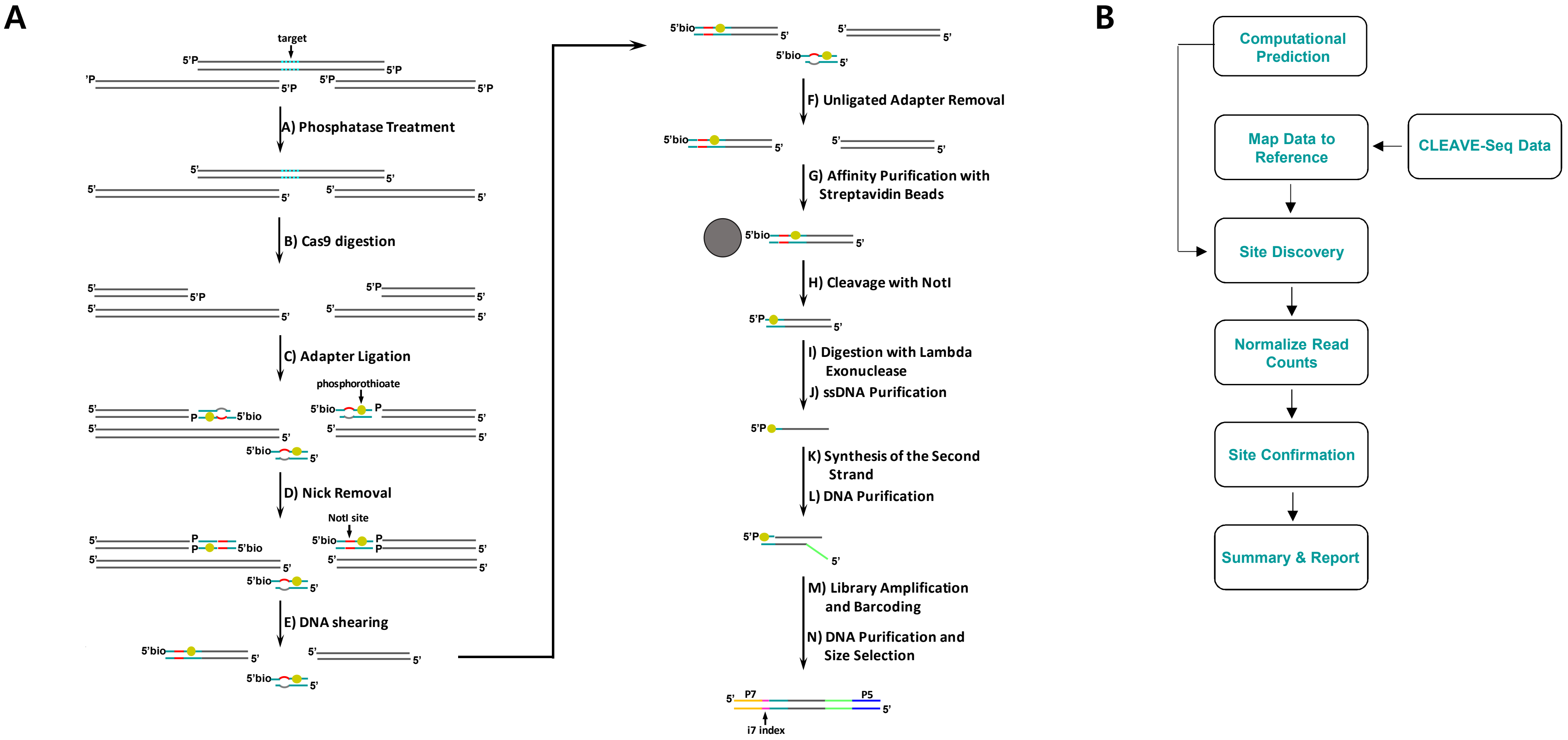

Supplementary Figure 2. A) Schematic of CLEAVE-Seq method. First, purified genomic DNA is dephosphorylated to reduce the number of free genomic DNA ends not generated by Cas9. Then, unphosphorylated adapters conjugated to biotin are used to capture Cas9 cleavage sites. Next, DNA fragment length is reduced and Cas9 cleaved fragments are enriched for by Streptavidin affinity purification. Finally, indexed libraries are generated for deep sequencing. B) Flow chart of target discovery informatics. First, computational prediction (e.g. Cas-OFFinder (Bae, Park et al. 2014) is used to identify putative genomic target sites. Next, CLEAVE-Seq data is combined with computation predictions to identify biochemically cleaved on- and off-target sites. The read count for each site is then normalized based on total read depth and confirmed as being unique to the Cas9 treated sample by comparisons to the control (reactions assembled in the absence of a guide RNA).

Supplementary Table 1 . A shortlist of computationally predicted M1 sites. On-target site is shown on the top. DNA and RNA Bulges are shown as gray and black boxes, respectively. Mismatches are shown in color boxes.

Site	Computational prediction		Sequence																								Genomic Location	Avg CLEAVE-Seq Reads
	Mismatch	*Bulge*	21	20	19	18	17	16	15	14	13	12	11	10	9	8	7	6	5	4	3	2	1	0	-1	-2		
	0	0	G	C	A	C	G	T	A	C	G	T	C	A	C	C	A	T	C	C	C	G	C	C	G	G	Chr1:14702765-14702788	200.78
1	2	1								■			G							A				C			Chr7:16502824-16502847	0.78
2	2	1								■			A							G				C			Chr3:12444708-12444731	0.00
3	2	1								■			G							G				C			Chr1:54992863-54992886	0.00
4	2	1								■			A				G							C			Chr8:38361070-38361093	1.41
5	2	1								■			G							G				C			Chr8:73407823-73407846	0.00
6	2	1								■			G							G				C			Chr10:26835370-26835393	1.46
7	2	1								■			G							G				C			Chr10:26867320-26867343	0.00
8	2	1								■			G							G				C			Chr10:142898890-142898913	0.21
9	2	1			T			■				G												C			Chr7:5753958-5753981	3.02
10	2	1							C						a							A		A			Chr7:156486399-156486422	3.02
11	2	1								■			G							G				C			Chr5:182622499-182622522	0.00
12	2	1								■			G							G				C			Chr1:77630220-77630243	0.00
13	2	1								■			A					G						C			Chr1:84645608-84645631	0.00
14	2	1								t							C					C		G	A		Chr3:56838689-56838712	0.00
15	2	1			T				■					C										C	A		Chr2:29281983-29282006	0.63

Supplementary Table 2. CLEAVE-seq data and A shortlist of computationally predicted M3 sites. On-target site is shown on the top. DNA and RNA Bulges are shown as gray and black boxes, respectively. Mismatches are shown in color boxes.

Site	Computational prediction		Sequence																							Genomic Location	Avg CLEAVE-Seq Reads
	Mismatch	*Bulge*	20	19	18	17	16	15	14	13	12	11	10	9	8	7	6	5	4	3	2	1	0	-1	-2		
	0	0	G	C	G	G	A	G	A	C	T	A	A	G	T	G	G	C	T	G	T	A	G	G	G	Chr2:4233941-4233964	164.19
1	2	1									A											G	T			Chr2:172786227-172786250	0.00
2	2	1															T	A					A			Chr7:43812401-43812424	2.46
3	2	1							C													G	A			Chr2:120060642-120060665	0.00
4	2	1									G				c						A		G	A		Chr5:93791118-93791140	0.00
5	2	1									G				c						A		G	A		Chr3:196197068-196197090	0.00
6	2	1							G				C										G	A		Chr5:168532712-168532734	0.00
7	2	1									G				c						A		G	A		Chr7:115695254-115695276	0.00
8	2	1									G				c			A					G	A		Chr7:62777273-62777295	0.00
9	2	1								G	C												G	A		Chr1:147217001-147217023	0.00
10	2	1																T				T	G	A		Chr1:286055591-286055613	0.00
11	2	1									G				c						A		G	A		Chr8:86546577-86546599	0.00
12	2	1									G				c						A		G	A		Chr1:76398359-76398381	0.00
13	2	1									G				c		A						G	A		Chr10:64318847-64318869	0.00
14	2	1									G				c						A		G	A		Chr8:86529185-86529207	0.00
15	2	1									G				c						A		G	A		Chr5:93800999-93801021	0.00

Supplementary Table 3. CLEAVE-seq data and a shortlist of computationally predicted M2 sites. On-target site is shown on the top. DNA and RNA Bulges are shown as gray and black boxes, respectively. Mismatches are shown in color boxes.

Site	Computational prediction		Sequence																							Genomic Location	Avg CLEAVE-Seq Reads
	Mismatch	*Bulge*	20	19	18	17	16	15	14	13	12	11	10	9	8	7	6	5	4	3	2	1	0	-1	-2		
1	0	0	G	G	C	C	G	A	G	G	T	C	G	A	C	T	A	C	C	G	G	C	C	G	G	Chr9:143631773-143631796	227.16
2	2	1			c		C		T														A			Chr2:37363776-37363799	14.68
3	1	1									G												A			Chr3:200475375-200475398	48.43
4	2	1						C			G												A			Chr10:5286571-5286594	111.10
5	2	1			G							G				g							C			Chr5:139807793-139807816	3.50
6	2	1									G			G									A			Chr5:196093089-196093112	0.00
7	1	1				A																	G			Chr6:166308906-166308929	12.21
8	2	1			A										T								G			Chr5:219494224-219494247	0.00
9	2	1		T												C							A			Chr6:157449386-157449409	1.00
10	1	1														C							C			Chr3:214572069-214572092	0.00
11	2	1												T		C							G			Chr6:9938751-993874	0.00
12	2	1												T		C							G			Chr9:3475286-3475309	0.00
13	2	1												T		C							G			Chr3:218090730-218090753	0.00
14	2	1												T		C							G			Chr8:80758362-80758385	0.00
15	2	1												T		C							G			Chr5:16787335-16787358	0.00
16	2	1												T		C							G			Chr5:174188899-174188922	0.00
17	2	1												T		C							G			Chr4:17679359-17679382	0.00
18	2	1												T		C							G			Chr3:174183068-174183091	0.00
19	2	1												T		C							G			Chr9:16372961-16372984	0.00
20	2	1				G								G									T			Chr6:35449294-35449317	0.00
21	2	1	C												g		T						C			Chr2:48116260-48116283	0.00
22	2	1	C												g		T						C			Chr10:120428313-120428336	0.00
23	2	1												T					T				G			Chr9:78374914-78374937	0.00
24	2	1												T					T				G			Chr6:40580933-40580956	0.00
25	2	1												T					T				G			Chr1:57503045-57503068	0.00
26	2	1												T					T				G			Chr3:216792332-216792355	0.00
27	2	1												T					T				G			Chr8:138192658-138192681	0.00
28	2	1												T					T				G			Chr10:117146380-117146403	0.00
29	2	1														C			G				A			Chr4:184633059-184633082	0.00
30	2	1														C			G				G			Chr8:123353866-123353889	0.00
31	2	1														C			G				G			Chr4:46962972-46962995	0.00
32	2	1														C			G				G			Chr4:46981868-46981891	0.00
33	2	1														C			G				G			Chr1:130948589-130948612	0.00
34	2	1														C			G				G			Chr8:101460540-101460563	0.00
35	2	1														C			G				G			Chr10:38760703-38760726	0.00
36	2	1														C			G				G			Chr10:38770324-38770347	0.00
37	2	1														C			G				A			Chr5:218532749-218532772	0.00
38	2	1														C			G				A			Chr5:218542334-218542357	0.00
39	2	1														C			T				G			Chr10:83019302-83019325	0.00
40	2	1												C					A				T			Chr3:5743937-5743960	0.00
41	2	1																G	G				G			Chr9:63992902-63992925	0.00
42	2	1							T														G			Chr9:57340264-57340287	0.00
43	2	1												C							C		T			Chr4:146163129-146163152	0.00
44	2	1												C							C		T			Chr2:213478153-213478176	0.00
45	2	1														C			A				A			Chr5:223273918-223273940	2.38
46	2	1										G				A							C	A		Chr1:137725782-137725804	0.00
47	2	1												T		C							G	A		Chr1:159151911-159151933	0.00
48	2	1												T		C							G	A		Chr1:199634954-199634976	0.00
49	2	1							C		A												G	A		Chr1:232879291-232879313	0.00
50	2	0															T		G				G	A		Chr1:304501362-304501384	0.00
51	2	0												C								A	C	A		Chr2:75055786-75055808	0.00
52	2	0															T		G				G	A		Chr3:145198993-145199015	0.00
53	2	1				G							c			C							G	A		Chr4:79878295-79878317	0.00
54	2	1							C										t				A	A		Chr5:2689067-2689089	0.00
55	2	1				G							c			C							G	A		Chr5:110101791-110101813	0.00
56	2	1				G							c			C							G	A		Chr5:110111414-110111436	0.00
57	2	1														G	G						T	A		Chr5:126305549-126305571	0.00
58	2	0															T		G				G	A		Chr6:125674373-125674395	0.00
59	2	1									C		A										G	A		Chr7:124810038-124810060	39.49
60	2	1				G							C		T								G	A		Chr8:15761268-15761290	0.00
61	2	1				G							C		T								G	A		Chr8:15770931-15770953	0.00
62	2	1														G	G						T	A		Chr8:50000473-50000495	0.00
63	2	1														C	G						A	A		Chr8:139620037-139620059	0.00
64	2	1					C						C	C									A	A		Chr9:22064095-22064117	0.00
65	2	1			T											G							T	A		Chr9:59915238-59915260	0.00
66	2	0															T		G				G	A		Chr9:159463178-159463200	0.00

Supplementary Table 4. Comprehensive list of cleaved genomic target sites detected from M2 guide RNA using CLEAVE-Seq

Location	Cut Site	CutSite_WRT_Target	MM_Type	DNA_Sequence	RNA_Sequence	Strand	Mismatches	Bulge Size	Normalized Read Count Rep 1	Normalized Read Count Rep 2	Avg CLEAVE-Seq Reads
Chr5:37860732-37860754	37860737	3	RNA	GGCCGAGGTCGACTACCGGCNRG	tG--GgGGTCGACTACCGGCAGG	-	2	2	469	589.33	529.17
Chr9:143631773-143631795	143631790	3	X	GGCCGAGGTCGACTACCGGCNRG	GGCCGAGGTCGACTACCGGCCGG	+	0	0	234	220.31	227.16
Chr10:5286571-5286593	5286586	5	RNA	GGCCGAGGTCGACTACCGGCNRG	G-CCGcGGgCGACTACCGGCAGG	+	2	1	79	143.20	111.10
Chr3:200475375-200475397	200475390	5	RNA	GGCCGAGGTCGACTACCGGCNRG	G-CCGAGGgCGACTACCGGCAGG	+	1	1	28	68.85	48.43
Chr7:124810038-124810060	124810053	5	RNA	GGCCGAGGTCGACTACCGGCNRG	GGC-GAGGcCaACTACCGGCGAG	+	2	1	25	53.98	39.49
Chr5:62435814-62435836	62435830	4	RNA	GGCCGAGGTCGACTACCGGCNRG	G-aCGAGaTCGACaACaaGCGGG	+	5	1	21	31.94	26.47
Chr5:144051759-144051781	144051776	3	DNA	GGCC-GAGGTCGACTACCGGCNRG	GGaCTGAaGggGAaTACCGGCTGG	+	5	1	26	12.67	19.34
Chr7:61544299-61544321	61544305	4	DNA	GGC-CGAGGTCGACTACCGGCNRG	GcCTCGAGGaCcACTtCCGGCTGG	-	4	1	13	24.78	18.89
Chr2:152062830-152062852	152062846	4	RNA	GGCCGAGGTCGACTACCGGCNRG	GaCaG-GGTCGACaACaaGCGGG	+	5	1	1	31.94	16.47
Chr10:84440789-84440811	84440794	3	RNA	GGCCGAGGTCGACTACCGGCNRG	G-aCGAGGTCGACaACaaGCGGG	-	4	1	13	19.83	16.42
Chr10:106127836-106127858	106127852	4	RNA	GGCCGAGGTCGACTACCGGCNRG	GaCaG-GGTCGACaACaaGCGGG	+	5	1	3	29.19	16.10
Chr2:37363776-37363798	37363794	2	DNA	GGCC-GAGGTCGACTACCGGCNRG	GGCCCcAtGTCGACTACCGGCAGG	+	2	1	20	9.36	14.68
Chr5:69134156-69134178	69134163	5	RNA	GGCCGAGGTCGACTACCGGCNRG	GG--GgacaCGcCTACCGGCGGG	-	5	2	26	0.00	13.00
Chr2:6864100-6864122	6864116	4	RNA	GGCCGAGGTCGACTACCGGCNRG	GGtCGgGGT-GAgTACCtGCGGG	+	4	1	10	14.87	12.44
Chr6:166308906-166308928	166308922	4	RNA	GGCCGAGGTCGACTACCGGCNRG	GGCaGAGGTCGA-TACCGGCGGG	+	1	1	20	4.41	12.21
Chr3:33020604-33020626	33020609	3	RNA	GGCCGAGGTCGACTACCGGCNRG	tGaCG-GGTCGACaACaaGCGGG	-	5	1	2	22.03	12.02
Chr1:259957715-259957737	259957732	3	X	GGCCGAGGTCGACTACCGGCNRG	GGCCGAGcTCtACcAgCGGCGGG	+	4	0	21	2.20	11.60
Chr7:173232468-173232490	173232473	3	RNA	GGCCGAGGTCGACTACCGGCNRG	G-aCtAGaTCGACTACCGGCGGG	-	3	1	11	11.57	11.29
Chr2:80845034-80845056	80845049	5	RNA	GGCCGAGGTCGACTACCGGCNRG	G--CGAGcgCGACTACCtGCGGG	+	3	2	17	4.41	10.71
Chr10:101313469-101313491	101313487	2	RNA	GGCCGAGGTCGACTACCGGCNRG	GGgCGAG--CGctgACaGGCGGG	+	5	2	3	18.18	10.59
Chr7:119054025-119054047	119054032	5	RNA	GGCCGAGGTCGACTACCGGCNRG	GGCgcAGccaGACT-CCGGCAAG	-	5	1	7	13.77	10.39
Chr3:232791371-232791393	232791387	4	RNA	GGCCGAGGTCGACTACCGGCNRG	G-aCGgGGTCGACaACgaGCGGG	+	5	1	4	16.52	10.26
Chr5:102819446-102819468	102819453	5	RNA	GGCCGAGGTCGACTACCGGCNRG	GGC-GAGtaCGAactCCGGCGAG	-	5	1	2	17.62	9.81
Chr1:17488618-17488640	17488625	5	DNA	GGC-CGAGGTCGACTACCGGCNRG	GcCTCGAGGaCGtCTtCCGGCTGG	-	4	1	19	0.00	9.50
Chr4:245455294-245455316	245455298	2	DNA	GGCCGAGGTCGACTAC-CGGCNRG	GGCCGAGGggGAaTcCGCGGtCGG	-	5	1	0	18.73	9.37
Chr4:240239220-240239242	240239225	3	RNA	GGCCGAGGTCGACTACCGGCNRG	G-aCGgGGTCGACgACgaGCGGG	-	5	1	11	7.71	9.36
Chr2:44981747-44981769	44981754	5	RNA	GGCCGAGGTCGACTACCGGCNRG	aGCCGAtaTCaACTACC--gGAG	-	5	2	18	0.00	9.00
Chr1:53187763-53187785	53187770	5	RNA	GGCCGAGGTCGACTACCGGCNRG	GGCaGAtcTgGgCTA--GGCAAG	-	5	2	18	0.00	9.00
Chr7:82662153-82662175	82662160	5	RNA	GGCCGAGGTCGACTACCGGCNRG	GttCGgGGaCGtCT-CCGGCGAG	-	5	1	18	0.00	9.00
Chr7:102664667-102664689	102664684	3	RNA	GGCCGAGGTCGACTACCGGCNRG	GaCCGAGGT-GACgACaaGaAGG	+	5	1	18	0.00	9.00
Chr9:54983896-54983918	54983902	4	RNA	GGCCGAGGTCGACTACCGGCNRG	GGC--AGGcCaACctCCGGCCGG	-	4	2	0	17.62	8.81
Chr3:152958354-152958376	152958369	5	RNA	GGCCGAGGTCGACTACCGGCNRG	cGgCGAacTtG--TACCGGCCGG	+	5	2	0	17.62	8.81
Chr9:70745680-70745702	70745697	3	DNA	GGC-CGAGGTCGACTACCGGCNRG	GcCTCGAGGaCGtCTtCCGGCTGG	+	4	1	2	15.42	8.71
Chr2:197687617-197687639	197687633	4	RNA	GGCCGAGGTCGACTACCGGCNRG	G-aCGAGaTCGACaACCaGCGGG	+	4	1	10	7.16	8.58
Chr5:134414881-134414903	134414887	4	RNA	GGCCGAGGTCGACTACCGGCNRG	GGaCGAGGTCGACcAaat-CCGG	-	5	1	0	17.07	8.54
Chr1:25488084-25488106	25488101	3	DNA	GG-CCGAGGTCGACTACCGGCNRG	GGTCCGAGtTCaAgaACCaGCAGG	+	5	1	0	17.07	8.54
Chr4:100282684-100282706	100282690	4	X	GGCCGAGGTCGACTACCGGCNRG	GGaaGAGGTgGcCTAgCGGCTGG	-	5	0	0	17.07	8.54
Chr5:99134781-99134803	99134798	3	RNA	GGCCGAGGTCGACTACCGGCNRG	GGCtGAGaTCaA--ACCGtgCAG	+	5	2	17	0.00	8.50
Chr4:220583733-220583755	220583749	4	X	GGCCGAGGTCGACTACCGGCNRG	GGCCGAGGTCcAtcACCtGCTGG	+	4	0	3	13.77	8.39
Chr2:56588382-56588404	56588398	4	RNA	GGCCGAGGTCGACTACCGGCNRG	G-aCGgGGTCGACgACgaGCGGG	+	5	1	0	16.52	8.26
Chr1:228584553-228584575	228584569	4	RNA	GGCCGAGGTCGACTACCGGCNRG	G-aCGgGGTCGACaACgaGCGGG	+	5	1	5	11.02	8.01
Chr2:59843341-59843363	59843348	5	DNA	GGC--CGAGGTCGACTACCGGCNRG	GGCGTCGAGGaCctCTACgtGCCGG	-	5	2	16	0.00	8.00
Chr5:90344751-90344773	90344768	3	DNA	GGCCGAGGTCGACTAC-CGGCNRG	GGCtGAGGTCGAggcCGaGGCGAG	+	5	1	16	0.00	8.00
Chr2:146608744-146608766	146608760	4	RNA	GGCCGAGGTCGACTACCGGCNRG	GGCtaAGcTgGACTA--GGtTGG	+	5	2	0	15.97	7.99
Chr2:52422323-52422345	52422330	5	RNA	GGCCGAGGTCGACTACCGGCNRG	GGgCGAGc-CaACctCCGGCCGG	-	5	1	0	15.97	7.99
Chr7:19666173-19666195	19666188	5	RNA	GGCCGAGGTCGACTACCGGCNRG	GGC--ttGcgGAtTACCGGCTGG	+	5	2	6	9.91	7.96
Chr2:187376830-187376852	187376848	2	RNA	GGCCGAGGTCGACTACCGGCNRG	G--CacGGgCGAgaACCGGCCAG	+	5	2	8	7.71	7.86
Chr6:42796472-42796494	42796478	4	RNA	GGCCGAGGTCGACTACCGGCNRG	G-CCGAaGTacACTgCCtGCTGG	-	5	1	9	6.61	7.81
Chr4:132703610-132703632	132703628	2	RNA	GGCCGAGGTCGACTACCGGCNRG	cGCgaAGGTgGA--ACgGGCAGG	+	5	2	0	15.42	7.71
Chr6:82325146-82325168	82325152	4	RNA	GGCCGAGGTCGACTACCGGCNRG	GGC--tcGcgGACTACCGaCTGG	-	5	2	0	15.42	7.71
Chr2:5765592-5765614	5765610	2	RNA	GGCCGAGGTCGACTACCGGCNRG	GGC--AGGgtGAgTgCgGGCGGG	+	5	2	0	15.42	7.71
Chr8:102976801-102976823	102976819	2	RNA	GGCCGAGGTCGACTACCGGCNRG	GGtCGtGGTCGAC--CgGaaCAG	+	5	2	0	15.42	7.71
Chr8:90266557-90266579	90266572	5	DNA	GGC--CGAGGTCGACTACCGGCNRG	GGCCTCGgGGgCtACacCCGGCGGG	+	5	2	0	15.42	7.71
Chr1:29132869-29132891	29132874	3	RNA	GGCCGAGGTCGACTACCGGCNRG	GaCaG-GGTCGACgACaaGCGGG	-	5	1	0	15.42	7.71
Chr3:147799357-147799379	147799362	3	RNA	GGCCGAGGTCGACTACCGGCNRG	G-aCGgGGTCGACgACCaGCGGG	-	4	1	0	15.42	7.71
Chr6:133774274-133774296	133774291	3	DNA	GGC-CGAGGTCGACTACCGGCNRG	GcCTCGAGGaCGtCTtCCGGCTGG	+	4	1	11	4.41	7.71
Chr7:101947601-101947623	101947608	5	DNA	GGC-CGAGGTCGACTACCGGCNRG	GGCGCGgGcTCGACactCGGCGAG	-	5	1	15	0.00	7.50
Chr5:190861491-190861513	190861498	5	RNA	GGCCGAGGTCGACTACCGGCNRG	GtCtGtGtTtGACT-CCGGCTGG	-	5	1	15	0.00	7.50
Chr8:137765681-137765703	137765688	5	RNA	GGCCGAGGTCGACTACCGGCNRG	GtCCGAaaT--AaTACCGGaAAG	-	5	2	15	0.00	7.50
Chr7:108353924-108353946	108353941	3	RNA	GGCCGAGGTCGACTACCGGCNRG	tGCCGAcGaCGACTgCt--CCAG	+	5	2	15	0.00	7.50
Chr3:2742482-2742504	2742489	5	DNA	GGCCGAGGTCGACT--ACCGGCNRG	aGCaGAGGTgGACTCGAaCGGCAGG	-	4	2	15	0.00	7.50
Chr2:228111375-228111397	228111382	5	RNA	GGCCGAGGTCGACTACCGGCNRG	GaCCGAGGgtGcCT-CgGGCTGG	-	5	1	15	0.00	7.50
Chr2:139725408-139725430	139725425	3	RNA	GGCCGAGGTCGACTACCGGCNRG	GGCCatGGTCGACTAttc-CCAG	+	5	1	0	14.87	7.44
Chr7:42529888-42529910	42529903	5	RNA	GGCCGAGGTCGACTACCGGCNRG	aGC--ttGTgGACTACCGGCTGG	+	4	2	0	14.87	7.44
Chr5:183185433-183185455	183185438	3	RNA	GGCCGAGGTCGACTACCGGCNRG	GGCCGAcGTCG-CTAgCaGCAGG	-	3	1	0	14.87	7.44
Chr4:202796965-202796987	202796980	5	RNA	GGCCGAGGTCGACTACCGGCNRG	ttCCc--GTCaACTACCGGCGGG	+	4	2	1	13.77	7.39
Chr4:142294326-142294348	142294343	3	DNA	GGC-CGAGGTCGACTACCGGCNRG	GcCTCGAGGaCGtCTtCCGGCTGG	+	4	1	4	10.46	7.23
Chr5:167972398-167972420	167972402	2	RNA	GGCCGAGGTCGACTACCGGCNRG	GGgCGA--TCGAgaACtaGCTGG	-	5	2	0	14.32	7.16
Chr2:238855968-238855990	238855984	4	RNA	GGCCGAGGTCGACTACCGGCNRG	GGaCGAGGgCGAaT-CCGGCGGG	+	3	1	0	14.32	7.16
Chr8:167939418-167939440	167939422	2	RNA	GGCCGAGGTCGACTACCGGCNRG	GG--GAGaTgaACTtCCGGaGAG	-	5	2	0	14.32	7.16
Chr4:178023389-178023411	178023407	2	RNA	GGCCGAGGTCGACTACCGGCNRG	t--CGcGGTCGgCTAaCGGtGGG	+	5	2	0	14.32	7.16
Chr2:85683422-85683444	85683428	4	RNA	GGCCGAGGTCGACTACCGGCNRG	GcCtcAGG-CGAgTACtGGCAGG	-	5	1	0	14.32	7.16
Chr1:189492830-189492852	189492835	3	RNA	GGCCGAGGTCGACTACCGGCNRG	GGCtGgGGTaG--cACCGGgAGG	-	5	2	0	14.32	7.16
Chr5:209743451-209743473	209743468	3	RNA	GGCCGAGGTCGACTACCGGCNRG	cGCCtcc--CaACTACCGGCAGG	+	5	2	0	14.32	7.16
Chr10:142955448-142955470	142955465	3	RNA	GGCCGAGGTCGACTACCGGCNRG	GGCCGAGGcCGAg--CCctaGGG	+	5	2	14	0.00	7.00
Chr7:165223993-165224015	165224010	3	DNA	G--GCCGAGGTCGACTACCGGCNRG	GTAGCCGAGGcCGAgctCgGGCGAG	+	5	2	14	0.00	7.00
Chr4:220064080-220064102	220064097	3	RNA	GGCCGAGGTCGACTACCGGCNRG	tGCCGAtGTCtg-TACCtGCAGG	+	5	1	14	0.00	7.00
Chr8:93987706-93987728	93987723	3	RNA	GGCCGAGGTCGACTACCGGCNRG	GGCCtcGGaCGgCgACC--CGGG	+	5	2	14	0.00	7.00
Chr7:125135211-125135233	125135216	3	DNA	GGCCGAGGTCGAC-TACCGGCNRG	GGCCatGGcCaACGcACCGGCGGG	-	5	1	5	8.81	6.91
Chr6:77861766-77861788	77861782	4	RNA	GGCCGAGGTCGACTACCGGCNRG	GGC--tcGcaGACTACCGcCTGG	+	5	2	0	13.77	6.89
Chr2:18015029-18015051	18015033	2	RNA	GGCCGAGGTCGACTACCGGCNRG	GGCCGAGcTgGAgTAt-GGCCGG	-	4	1	0	13.77	6.89
Chr2:215736339-215736361	215736355	4	RNA	GGCCGAGGTCGACTACCGGCNRG	GGC--tcGcgGACTACCGcCTGG	+	5	2	0	13.77	6.89
Chr10:148195714-148195736	148195720	4	RNA	GGCCGAGGTCGACTACCGGCNRG	GGCgGAGGTaGAtT--CGagGAG	-	5	2	0	13.77	6.89
Chr10:77277596-77277618	77277613	3	RNA	GGCCGAGGTCGACTACCGGCNRG	aGgCaAGtTCGAgTA--GGCAGG	+	5	2	0	13.77	6.89
Chr4:168992271-168992293	168992276	3	RNA	GGCCGAGGTCGACTACCGGCNRG	aGC--AGGcCaACTACCGctTGG	-	5	2	0	13.77	6.89
Chr4:45623225-45623247	45623241	4	RNA	GGCCGAGGTCGACTACCGGCNRG	G-aCGAGaTCGACaACgaGCGGG	+	5	1	0	13.77	6.89
Chr9:9413650-9413672	9413665	5	RNA	GGCCGAGGTCGACTACCGGCNRG	aGCCaAGtTg--CTcCCGGCGGG	+	5	2	0	13.77	6.89
Chr9:49772148-49772170	49772155	5	RNA	GGCCGAGGTCGACTACCGGCNRG	GGt-GAGGTCGgCcggCGGCTGG	-	5	1	7	6.61	6.81
Chr10:32474621-32474643	32474626	3	RNA	GGCCGAGGTCGACTACCGGCNRG	tGCCGA--TacACcgCCGGCCGG	-	5	2	0	13.22	6.61
Chr4:170766307-170766329	170766324	3	DNA	GGCCGAGGTCGACTACCG--GCNRG	GGCgGAGGTCGcCggCtGATGCCGG	+	5	2	0	13.22	6.61
Chr4:159977272-159977294	159977288	4	RNA	GGCCGAGGTCGACTACCGGCNRG	GGCaGtGGcgGACTAC-GGtCAG	+	5	1	0	13.22	6.61
Chr1:94016475-94016497	94016480	3	RNA	GGCCGAGGTCGACTACCGGCNRG	GcCCGAGccCGAgT--CGGtAGG	-	5	2	0	13.22	6.61
Chr2:10567713-10567735	10567719	4	RNA	GGCCGAGGTCGACTACCGGCNRG	GcggcAGGTgG--TACCGGCGAG	-	5	2	0	13.22	6.61
Chr2:178371913-178371935	178371928	5	RNA	GGCCGAGGTCGACTACCGGCNRG	GGC--tcGcgGACTACCGtCTGG	+	5	2	0	13.22	6.61
Chr3:25383214-25383236	25383231	3	DNA	GGC-CGAGGTCGACTACCGGCNRG	GcCTCGAGGaCGtCTtCCGGCTGG	+	4	1	1	12.12	6.56
Chr4:108817238-108817260	108817253	5	RNA	GGCCGAGGTCGACTACCGGCNRG	cGC--AGGgCtACTgCCGGCCGG	+	4	2	7	6.06	6.53
Chr3:110757482-110757504	110757489	5	RNA	GGCCGAGGTCGACTACCGGCNRG	G-aCGAGGTCGACgACgaGaTAG	-	5	1	13	0.00	6.50
Chr1:120789643-120789665	120789650	5	RNA	GGCCGAGGTCGACTACCGGCNRG	aGagcAG--CGgCTACCGGCTGG	-	5	2	13	0.00	6.50
Chr6:29254942-29254964	29254959	3	RNA	GGCCGAGGTCGACTACCGGCNRG	tGCaGAGcTCGgCTA--GGtTGG	+	5	2	13	0.00	6.50
Chr2:38124656-38124678	38124673	3	RNA	GGCCGAGGTCGACTACCGGCNRG	tGgCt-cGTgGACTACCGGCTGG	+	5	1	13	0.00	6.50
Chr5:26296327-26296349	26296344	3	RNA	GGCCGAGGTCGACTACCGGCNRG	ccCCGAaGTCGAtTAtC--CCGG	+	5	2	13	0.00	6.50
Chr8:12282731-12282753	12282748	3	DNA	GG-CCGAGGTCGACTACCGGCNRG	GGTCCGAGtTCaAgaACCtGCAGG	+	5	1	13	0.00	6.50
Chr7:129636568-129636590	129636585	3	RNA	GGCCGAGGTCGACTACCGGCNRG	GGCCGAGtaCG--TtgCcGCAGG	+	5	2	13	0.00	6.50
Chr5:30544853-30544875	30544860	5	RNA	GGCCGAGGTCGACTACCGGCNRG	GcCaGtGGaCGAC--CCGGCTAG	-	4	2	13	0.00	6.50
Chr4:79823897-79823919	79823914	3	RNA	GGCCGAGGTCGACTACCGGCNRG	GcCgGAGccCGAC--CCGtCAAG	+	5	2	13	0.00	6.50
Chr1:10571571-10571593	10571578	5	RNA	GGCCGAGGTCGACTACCGGCNRG	GGtaGctGTCGACT-CtGGCGGG	-	5	1	13	0.00	6.50
Chr4:218341908-218341930	218341925	3	RNA	GGCCGAGGTCGACTACCGGCNRG	GGCCGcGGcCG--TgCCGGaGAG	+	4	2	13	0.00	6.50
Chr7:3885860-3885882	3885877	3	RNA	GGCCGAGGTCGACTACCGGCNRG	aGCaGgGcTCGACTAC-GGaCGG	+	5	1	13	0.00	6.50
Chr5:5053680-5053702	5053697	3	RNA	GGCCGAGGTCGACTACCGGCNRG	GGCgGcGGgCGgCTt--GGCAGG	+	5	2	13	0.00	6.50
Chr6:644841-644863	644858	3	RNA	GGCCGAGGTCGACTACCGGCNRG	GGCCtAGtTgcACTACt--CAGG	+	5	2	4	8.81	6.41
Chr6:153932019-153932041	153932035	4	RNA	GGCCGAGGTCGACTACCGGCNRG	GGCaGAGGTtGgCgAg-GGCCGG	+	5	1	5	7.71	6.36
Chr7:105603645-105603667	105603652	5	RNA	GGCCGAGGTCGACTACCGGCNRG	aGttGAG--aGACTACCGGgGGG	-	5	2	0	12.67	6.34
Chr5:176778964-176778986	176778982	2	RNA	GGCCGAGGTCGACTACCGGCNRG	GGCtcAG--CGACTgaCGGgAAG	+	5	2	0	12.67	6.34
Chr5:34923149-34923171	34923154	3	DNA	GGC--CGAGGTCGACTACCGGCNRG	GGCCTCGAGGgCtACacCCGGtGGG	-	5	2	0	12.67	6.34
Chr4:163611678-163611700	163611696	2	RNA	GGCCGAGGTCGACTACCGGCNRG	GGC--AGGcCGAgcACtaGCCAG	+	5	2	0	12.67	6.34
Chr2:82089788-82089810	82089792	2	RNA	GGCCGAGGTCGACTACCGGCNRG	tGCaGAGcTCGgCTA--GGCTGG	-	4	2	0	12.67	6.34
Chr6:117209554-117209576	117209572	2	RNA	GGCCGAGGTCGACTACCGGCNRG	GaCCGAGG-CGACgACaaGaAGG	+	5	1	0	12.67	6.34
Chr8:70932227-70932249	70932245	2	RNA	GGCCGAGGTCGACTACCGGCNRG	GGgCGAGtTgGgTA--GGCAGG	+	5	2	0	12.67	6.34
Chr4:176320826-176320848	176320842	4	RNA	GGCCGAGGTCGACTACCGGCNRG	G-aCGgGGTCGACaACgaGCGGG	+	5	1	0	12.67	6.34
Chr2:78095139-78095161	78095144	3	RNA	GGCCGAGGTCGACTACCGGCNRG	GGCCGAGcTCccCTggCG-CAGG	-	5	1	0	12.67	6.34
Chr10:71971177-71971199	71971193	4	RNA	GGCCGAGGTCGACTACCGGCNRG	GGC-GtGGTCGAggACtGGCGGG	+	4	1	0	12.67	6.34
Chr1:96437551-96437573	96437557	4	DNA	GGC-CGAGGTCGACTACCGGCNRG	GcCTCGAGGaCGtCTtCCGGCTGG	-	4	1	7	5.51	6.26
Chr3:85941004-85941026	85941010	4	RNA	GGCCGAGGTCGACTACCGGCNRG	tGaaGAGGc--ACTACCtGCGGG	-	5	2	0	12.12	6.06
Chr1:147535889-147535911	147535904	5	RNA	GGCCGAGGTCGACTACCGGCNRG	t--CGcGtTgaACTACCGGCTGG	+	5	2	0	12.12	6.06

Chr6:47560230-47560252	47560246	4	RNA	GGCCGAGGTCGACTACCGGCNRG	G-aCGAGaTCGACaACgaGCGGG	+	5	1	0	12.12	6.06
Chr1:113110081-113110103	113110088	5	DNA	GGCCGAGGTCGACT-ACCGGCNRG	GGCtGgGGaCGACTGACaGGtGGG	-	5	1	0	12.12	6.06
Chr8:56668510-56668532	56668528	2	RNA	GGCCGAGGTCGACTACCGGCNRG	aGCCGAcG--GACTcgCtGCTGG	+	5	2	0	12.12	6.06
Chr3:158346111-158346133	158346128	3	RNA	GGCCGAGGTCGACTACCGGCNRG	GGCCGgGtcgGAC--CgGGCCGG	+	5	2	0	12.12	6.06
Chr6:160184621-160184643	160184639	2	RNA	GGCCGAGGTCGACTACCGGCNRG	GaCC-AGGTCttCTtCCaGCAGG	+	5	1	0	12.12	6.06
Chr9:109053816-109053838	109053831	5	RNA	GGCCGAGGTCGACTACCGGCNRG	G--tGAGGaCGACTgCaGGCGGG	+	4	2	0	12.12	6.06
Chr5:17487623-17487645	17487628	3	RNA	GGCCGAGGTCGACTACCGGCNRG	G-aCGAGGTCGACaACgaGCGGG	-	4	1	0	12.12	6.06
Chr6:101731284-101731306	101731290	4	DNA	GGC-CGAGGTCGACTACCGGCNRG	GcCTCGAGGgCatCTtCCGGCGGG	-	5	1	0	12.12	6.06
Chr8:153226687-153226709	153226694	5	RNA	GGCCGAGGTCGACTACCGGCNRG	ttCCGAtGTCGAtTg--GGCGGG	-	5	2	0	12.12	6.06
Chr3:42113853-42113875	42113869	4	RNA	GGCCGAGGTCGACTACCGGCNRG	GGCCGAcGcCGAtgA--GaCGAG	+	5	2	0	12.12	6.06
Chr1:164563816-164563838	164563831	5	RNA	GGCCGAGGTCGACTACCGGCNRG	GGCaGAGGT-GACggCCGGaGAG	+	4	1	0	12.12	6.06
Chr6:67380621-67380643	67380638	3	RNA	GGCCGAGGTCGACTACCGGCNRG	GGCCGcGGaCGACT-CtGGCGAG	+	3	1	12	0.00	6.00
Chr9:79804613-79804635	79804630	3	RNA	GGCCGAGGTCGACTACCGGCNRG	cGaCGAGcTCa--TACCGGCCGG	+	4	2	12	0.00	6.00
Chr2:57971352-57971374	57971369	3	RNA	GGCCGAGGTCGACTACCGGCNRG	GGaCGAG--CGctgACaGGCGGG	+	5	2	12	0.00	6.00
Chr6:75926787-75926809	75926804	3	DNA	G-GCCGAGGTCGACTACCGGCNRG	GTGCtGAGaTCGcCTAgCtGCGGG	+	5	1	12	0.00	6.00
Chr9:105659733-105659755	105659740	5	DNA	GGCCGAGGT--CGACTACCGGCNRG	GGCCGAGGTCGCGACTcatGGgCGG	-	4	2	12	0.00	6.00
Chr5:24959542-24959564	24959549	5	RNA	GGCCGAGGTCGACTACCGGCNRG	GG--GAGGggGgCTtCCGGCGGG	-	4	2	12	0.00	6.00
Chr1:163300941-163300963	163300958	3	RNA	GGCCGAGGTCGACTACCGGCNRG	GtgCGcGGaCG-CTACtGGCAGG	+	5	1	12	0.00	6.00
Chr7:152918617-152918639	152918624	5	DNA	GGCCGAGGTCGACTAC-CGGCNRG	cGCCaAGaTCGACaACGCGcCCGG	-	5	1	12	0.00	6.00
Chr9:133680153-133680175	133680170	3	RNA	GGCCGAGGTCGACTACCGGCNRG	GGaCcAGGTCG--TcCCaGtTGG	+	5	2	12	0.00	6.00
Chr6:66171617-66171639	66171634	3	RNA	GGCCGAGGTCGACTACCGGCNRG	G-aCGgGGTCGACaACgaGCGGG	+	5	1	12	0.00	6.00
Chr6:93690437-93690459	93690444	5	RNA	GGCCGAGGTCGACTACCGGCNRG	tGCCGAGGaC-ACTgCCtGgGAG	-	5	1	12	0.00	6.00
Chr1:122673697-122673719	122673704	5	RNA	GGCCGAGGTCGACTACCGGCNRG	GGCCGAaGc--ACTgCCaGaTAG	-	5	2	12	0.00	6.00
Chr4:80762391-80762413	80762408	3	RNA	GGCCGAGGTCGACTACCGGCNRG	G-aCGgGGTCGACaACgaGCGGG	+	5	1	12	0.00	6.00
Chr3:4545233-4545255	4545240	5	RNA	GGCCGAGGTCGACTACCGGCNRG	G-aCGgGGTCGACaACgaGCGGG	-	5	1	12	0.00	6.00
Chr4:108794616-108794638	108794633	3	RNA	GGCCGAGGTCGACTACCGGCNRG	tGgCt-cGTgGACTACCGGCTGG	+	5	1	12	0.00	6.00
Chr7:128072344-128072366	128072361	3	RNA	GGCCGAGGTCGACTACCGGCNRG	G-aCGAGGTCGAtaACgaGCGGG	+	5	1	12	0.00	6.00
Chr2:51154691-51154713	51154698	5	RNA	GGCCGAGGTCGACTACCGGCNRG	GGCCG-GGTCGtCcACtGGtGGG	-	4	1	12	0.00	6.00
Chr4:116151160-116151182	116151177	3	RNA	GGCCGAGGTCGACTACCGGCNRG	GGggGAGGTaGcCgA--GGCTGG	+	5	2	12	0.00	6.00
Chr2:6101136-6101158	6101143	5	RNA	GGCCGAGGTCGACTACCGGCNRG	cGCCaAGGTCGAggA--GGtCAG	-	5	2	12	0.00	6.00
Chr4:114880759-114880781	114880776	3	RNA	GGCCGAGGTCGACTACCGGCNRG	GGC-aAGGTCGACgACgtGaTGG	+	5	1	12	0.00	6.00
Chr8:27801455-27801477	27801462	5	RNA	GGCCGAGGTCGACTACCGGCNRG	G-aCGAGGTCGAtgACaaGCGGG	-	5	1	12	0.00	6.00
Chr5:120860746-120860768	120860764	2	DNA	GGCCGAGGTCGACTAC-CGGCNRG	GGtCGAGGgCGcgTcCACGGCGGG	+	5	1	5	6.61	5.81
Chr2:154881381-154881403	154881397	4	RNA	GGCCGAGGTCGACTACCGGCNRG	aGCaGgGcTCGACTAC-GGCCGG	+	4	1	0	11.57	5.79
Chr3:199019683-199019705	199019688	3	RNA	GGCCGAGGTCGACTACCGGCNRG	GGC--tcGgCGACaACaGGCTGG	-	5	2	0	11.57	5.79
Chr4:113372151-113372173	113372158	5	RNA	GGCCGAGGTCGACTACCGGCNRG	GGC-GtGGaCGAactCCGGCGAG	-	5	1	0	11.57	5.79
Chr6:154812669-154812691	154812687	2	RNA	GGCCGAGGTCGACTACCGGCNRG	GGCtcAGGctGACaAC--GCGGG	+	5	2	0	11.57	5.79
Chr4:159335495-159335517	159335500	3	RNA	GGCCGAGGTCGACTACCGGCNRG	GGCtcAcG--GACTACtGaCTGG	-	5	2	0	11.57	5.79
Chr8:93012665-93012687	93012681	4	RNA	GGCCGAGGTCGACTACCGGCNRG	G-aCGgGGTCGACgACgaGCGGG	+	5	1	0	11.57	5.79
Chr4:140692052-140692074	140692067	5	RNA	GGCCGAGGTCGACTACCGGCNRG	GGC-GAcGTCGACTACtacgAGG	+	5	1	0	11.57	5.79
Chr3:232121528-232121550	232121546	2	RNA	GGCCGAGGTCGACTACCGGCNRG	tG--GAGGagGAgcACCGGCTAG	+	5	2	0	11.57	5.79
Chr1:180423414-180423436	180423429	5	RNA	GGCCGAGGTCGACTACCGGCNRG	GGC--ttGcgGAtTACCGGCTGG	+	5	2	0	11.57	5.79
Chr5:208880944-208880966	208880960	4	RNA	GGCCGAGGTCGACTACCGGCNRG	GGCgGcGaTaGcCT--CGGCAAG	+	5	2	0	11.57	5.79
Chr7:2295613-2295635	2295620	5	DNA	GGCCGAGGTCGACTA--CCGGCNRG	GGCgGcGcTaGcCTACTCCGGCGAG	-	5	2	11	0.55	5.78
Chr8:161124161-161124183	161124167	4	DNA	GGC-CGAGGTCGACTACCGGCNRG	GcCTCGAGGaCGtCTtCCGGCTGG	-	4	1	6	5.51	5.76
Chr4:93494700-93494722	93494718	2	DNA	GGCCGAGGTC-GACTACCGGCNRG	GGCCGAGGcCTGAggtCgGGCGAG	+	5	1	6	5.51	5.76
Chr2:145249710-145249732	145249716	4	RNA	GGCCGAGGTCGACTACCGGCNRG	GGgCGAcGaCGgC-AgCGGCAAG	-	5	1	2	9.36	5.68
Chr4:154376219-154376241	154376223	2	RNA	GGCCGAGGTCGACTACCGGCNRG	GGCCGAtcTCG--cAgCaGCAAG	-	5	2	8	3.30	5.65
Chr6:144813984-144814006	144813991	5	RNA	GGCCGAGGTCGACTACCGGCNRG	GctCGAGaTCGACTg--GGaTGG	-	5	2	10	1.10	5.55
Chr2:169106015-169106037	169106030	5	RNA	GGCCGAGGTCGACTACCGGCNRG	G--tGAGGTaatCaACCGGCAGG	+	5	2	5	6.06	5.53
Chr1:80665235-80665257	80665239	2	RNA	GGCCGAGGTCGACTACCGGCNRG	GGCCa--GctGACgACCaGCTGG	-	5	2	0	11.02	5.51
Chr4:130428771-130428793	130428788	3	DNA	GGC-CGAGGTCGACTACCGGCNRG	GcCTCGAGGaCGtCTtCCGGCTGG	+	4	1	0	11.02	5.51
Chr5:197998205-197998227	197998222	3	RNA	GGCCGAGGTCGACTACCGGCNRG	GGCgGtGGTCGAtgAC--GaAGG	+	5	2	0	11.02	5.51
Chr7:105179774-105179796	105179791	3	RNA	GGCCGAGGTCGACTACCGGCNRG	GGCCGAGcgCGA--AggGGgAAG	+	5	2	0	11.02	5.51
Chr10:121776696-121776718	121776714	2	RNA	GGCCGAGGTCGACTACCGGCNRG	GGCCGgcGgCGAgg--CGGCGAG	+	5	2	0	11.02	5.51
Chr9:10406323-10406345	10406341	2	RNA	GGCCGAGGTCGACTACCGGCNRG	GGt--AGGcCaACctCCGGCCGG	+	5	2	0	11.02	5.51
Chr8:127021243-127021265	127021248	3	RNA	GGCCGAGGTCGACTACCGGCNRG	G-aCGgGGTCGACaACgaGCGGG	-	5	1	0	11.02	5.51
Chr4:177107054-177107076	177107070	4	DNA	GG-CCGAGGTCGACTACCGGCNRG	GGTCCGAGGTCGAggtCaGGCGAG	+	4	1	0	11.02	5.51
Chr9:82008064-82008086	82008081	3	RNA	GGCCGAGGTCGACTACCGGCNRG	GGaCGAGGgCG--cAggGGCAGG	+	5	2	0	11.02	5.51
Chr2:118622125-118622147	118622129	2	RNA	GGCCGAGGTCGACTACCGGCNRG	GGCCGAtcTCG--cAtCaGCAAG	-	5	2	0	11.02	5.51
Chr10:150131590-150131612	150131596	4	RNA	GGCCGAGGTCGACTACCGGCNRG	GGC--AGacCGAgcACCaGCCGG	-	5	2	0	11.02	5.51
Chr10:27044129-27044151	27044135	4	RNA	GGCCGAGGTCGACTACCGGCNRG	GGCCGccGTCGcCgtC--GCCAG	-	5	2	0	11.02	5.51
Chr5:75886405-75886427	75886420	5	RNA	GGCCGAGGTCGACTACCGGCNRG	GGC--tcGcgGACTACCGtCTGG	+	5	2	0	11.02	5.51
Chr4:215783270-215783292	215783274	2	RNA	GGCCGAGGTCGACTACCGGCNRG	GGCCGgGcTgGAC--CgGGCCGG	-	4	2	0	11.02	5.51
Chr10:93909137-93909159	93909154	3	RNA	GGCCGAGGTCGACTACCGGCNRG	GaCC-AGcTCGAgTACCtGgAGG	+	5	1	0	11.02	5.51
Chr10:70309314-70309336	70309331	3	RNA	GGCCGAGGTCGACTACCGGCNRG	ccCCcAG--CGAacACCGGCGAG	+	5	2	0	11.02	5.51
Chr1:30966896-30966918	30966912	4	DNA	GGCCGA-GGTCGACTACCGGCNRG	GGaCGATGGcCGACgAaCGGaAAG	+	5	1	0	11.02	5.51
Chr8:154260387-154260409	154260394	5	RNA	GGCCGAGGTCGACTACCGGCNRG	aGCCGtGtTCGACTACC--CGGG	-	3	2	11	0.00	5.50
Chr4:210393287-210393309	210393294	5	RNA	GGCCGAGGTCGACTACCGGCNRG	GGaCGAGGgC-ACTAtCGGCTGG	-	3	1	11	0.00	5.50
Chr4:82363661-82363683	82363668	5	RNA	GGCCGAGGTCGACTACCGGCNRG	cGaCGAcGTCGA-TcCCGGtGAG	-	5	1	11	0.00	5.50
Chr8:152619574-152619596	152619581	5	RNA	GGCCGAGGTCGACTACCGGCNRG	GaCCGAGG-CGACgACaaGaAGG	-	5	1	11	0.00	5.50
Chr10:40287830-40287852	40287837	5	RNA	GGCCGAGGTCGACTACCGGCNRG	caCCGAGtTgGAgTA--GGCAGG	-	5	2	11	0.00	5.50
Chr4:46003613-46003635	46003620	5	RNA	GGCCGAGGTCGACTACCGGCNRG	GagCGAG-TCGtCaACCGtCCAG	-	5	1	11	0.00	5.50
Chr5:49117147-49117169	49117164	3	RNA	GGCCGAGGTCGACTACCGGCNRG	GGCCGAGGaCcAggACC--CAAG	+	4	2	11	0.00	5.50
Chr1:121497457-121497479	121497474	3	RNA	GGCCGAGGTCGACTACCGGCNRG	GGaCG--GTCGcggACCGtCCGG	+	5	2	11	0.00	5.50
Chr2:224654169-224654191	224654186	3	DNA	GGCCGAGGTCGAC-TACCGGCNRG	aGCgGAGGgCGACGaACtGGCGGG	+	5	1	11	0.00	5.50
Chr8:162435245-162435267	162435252	5	RNA	GGCCGAGGTCGACTACCGGCNRG	cGCCGAc--CGAggACCtGCAGG	-	5	2	11	0.00	5.50
Chr6:161132228-161132250	161132235	5	RNA	GGCCGAGGTCGACTACCGGCNRG	tGgCGtGGTCctCTACC--CAGG	-	5	2	11	0.00	5.50
Chr5:178967961-178967983	178967978	3	DNA	GGCCG--AGGTCGACTACCGGCNRG	tGCCGGAAGGTaGgtTtCCGGCTAG	+	5	2	11	0.00	5.50
Chr1:71210030-71210052	71210037	5	RNA	GGCCGAGGTCGACTACCGGCNRG	GGCaGcGGTCGAC-AgCGGgCGG	-	4	1	11	0.00	5.50
Chr9:65874133-65874155	65874140	5	RNA	GGCCGAGGTCGACTACCGGCNRG	GGC--AGGTCGggcACtcGCCGG	-	5	2	11	0.00	5.50
Chr8:52557746-52557768	52557763	3	RNA	GGCCGAGGTCGACTACCGGCNRG	cGCCGAGG--GAgTtCCaGCAGG	+	4	2	11	0.00	5.50
Chr7:145657742-145657764	145657749	5	RNA	GGCCGAGGTCGACTACCGGCNRG	GtCttgGGT-GACcACCGGCGGG	-	5	1	11	0.00	5.50
Chr6:71767056-71767078	71767073	3	RNA	GGCCGAGGTCGACTACCGGCNRG	GGCaGtGa-CGACTACCaGCAGG	+	4	1	11	0.00	5.50
Chr6:97576679-97576701	97576696	3	RNA	GGCCGAGGTCGACTACCGGCNRG	GGC--tcGcgGACTACCGtCTGG	+	5	2	11	0.00	5.50
Chr1:273888216-273888238	273888233	3	RNA	GGCCGAGGTCGACTACCGGCNRG	GcCCGAGGaCaACcACC--aCGG	+	5	2	11	0.00	5.50
Chr1:85262125-85262147	85262130	3	RNA	GGCCGAGGTCGACTACCGGCNRG	GcCCGAGGcCGA--ACtcGgTGG	-	5	2	7	3.86	5.43
Chr7:77522872-77522894	77522877	3	RNA	GGCCGAGGTCGACTACCGGCNRG	GGaCct--TCGAaTAgCGGCAGG	-	5	2	3	7.71	5.36
Chr6:49789848-49789870	49789853	3	RNA	GGCCGAGGTCGACTACCGGCNRG	GcCCGAGGgCaACcACC--gTGG	-	5	2	4	6.61	5.31
Chr9:58610543-58610565	58610559	4	RNA	GGCCGAGGTCGACTACCGGCNRG	GGCCGAGGgCGcCT--CaGgCGG	+	4	2	10	0.55	5.28
Chr10:64036763-64036785	64036768	3	RNA	GGCCGAGGTCGACTACCGGCNRG	GGC--AGGcCGAgcACtcGCCGG	-	5	2	0	10.46	5.23
Chr8:11633727-11633749	11633732	3	RNA	GGCCGAGGTCGACTACCGGCNRG	GGC--tcGcgGACTACCGtCTGG	-	5	2	0	10.46	5.23
Chr2:194946823-194946845	194946830	5	RNA	GGCCGAGGTCGACTACCGGCNRG	GGC--AGGcCGAgcACtcGCCGG	-	5	2	0	10.46	5.23
Chr10:5403108-5403130	5403113	3	RNA	GGCCGAGGTCGACTACCGGCNRG	GGC-GgGGTCGAaTcCCGcCTGG	-	4	1	0	10.46	5.23
Chr9:52767017-52767039	52767023	4	RNA	GGCCGAGGTCGACTACCGGCNRG	GcCCGAGtTCGtCatC--GCCGG	-	5	2	0	10.46	5.23
Chr10:99687435-99687457	99687453	2	RNA	GGCCGAGGTCGACTACCGGCNRG	G--CGAGGTgGAgctCaGGCAGG	+	5	2	0	10.46	5.23
Chr4:35078794-35078816	35078799	3	RNA	GGCCGAGGTCGACTACCGGCNRG	GGgCG-GGcCaACctCCGGCCGG	-	5	1	0	10.46	5.23
Chr4:42312088-42312110	42312104	4	RNA	GGCCGAGGTCGACTACCGGCNRG	GGCtaAGG-CtACTAgCaGCAGG	+	5	1	0	10.46	5.23
Chr9:156369685-156369707	156369701	4	RNA	GGCCGAGGTCGACTACCGGCNRG	GGgCG-GGcCaACctCCGGCCGG	+	5	1	0	10.46	5.23
Chr2:152782016-152782038	152782032	4	RNA	GGCCGAGGTCGACTACCGGCNRG	a--CGAcGaCGACaACCGGCAAG	+	4	2	0	10.46	5.23
Chr6:125744945-125744967	125744952	5	RNA	GGCCGAGGTCGACTACCGGCNRG	caCCaAGtTCGcCT-CCGGCCAG	-	5	1	0	10.46	5.23
Chr4:230595515-230595537	230595520	3	RNA	GGCCGAGGTCGACTACCGGCNRG	GGC--tcGcgGACTACtGGCTGG	-	5	2	0	10.46	5.23
Chr8:78696806-78696828	78696813	5	DNA	GGC--CGAGGTCGACTACCGGCNRG	GGCCTCGgGGgCtACacCCGGCGGG	-	5	2	0	10.46	5.23
Chr10:58359901-58359923	58359916	5	RNA	GGCCGAGGTCGACTACCGGCNRG	GGCgGAaGTCG--TcCgGcCGAG	+	5	2	0	10.46	5.23
Chr4:158275718-158275740	158275735	3	RNA	GGCCGAGGTCGACTACCGGCNRG	GGC-GAGGTCGAggACCttgGGG	+	5	1	0	10.46	5.23
Chr3:481745-481767	481760	5	RNA	GGCCGAGGTCGACTACCGGCNRG	GGCCGgGGTgGAtgACCt-CGAG	+	5	1	0	10.46	5.23
Chr1:122869678-122869700	122869693	5	RNA	GGCCGAGGTCGACTACCGGCNRG	G--CGgGGaCatCTtCCGGCTGG	+	5	2	0	10.46	5.23
Chr8:70699892-70699914	70699897	3	RNA	GGCCGAGGTCGACTACCGGCNRG	GGC-GAGGTCGACgACgaGaTAG	-	4	1	0	10.46	5.23
Chr8:83477111-83477133	83477116	3	RNA	GGCCGAGGTCGACTACCGGCNRG	GGgCGAGcTCGgCcAC-GGCAAG	-	4	1	6	4.41	5.21
Chr1:228759322-228759344	228759339	3	RNA	GGCCGAGGTCGACTACCGGCNRG	tGCCtA--TCacCTACCaGCAGG	+	5	2	9	1.10	5.05
Chr2:184130574-184130596	184130581	5	RNA	GGCCGAGGTCGACTACCGGCNRG	G-aCGgGGTCGACaACgaGCGGG	-	5	1	10	0.00	5.00
Chr9:129865023-129865045	129865030	5	RNA	GGCCGAGGTCGACTACCGGCNRG	GGCCGAcGTCaAggAC-GGCGGG	-	4	1	10	0.00	5.00
Chr2:121388382-121388404	121388389	5	RNA	GGCCGAGGTCGACTACCGGCNRG	tGaCcAGGTCG--TcCCaGCTGG	-	5	2	10	0.00	5.00
Chr1:152646417-152646439	152646434	3	RNA	GGCCGAGGTCGACTACCGGCNRG	GGC-GAcGTCGACTACtacaAGG	+	5	1	10	0.00	5.00
Chr9:158630407-158630429	158630414	5	RNA	GGCCGAGGTCGACTACCGGCNRG	tGCCGAG--CGgaTAgCGGaGAG	-	5	2	10	0.00	5.00
Chr5:125101643-125101665	125101660	3	RNA	GGCCGAGGTCGACTACCGGCNRG	G-aCGgGGTCGACaACgaGCGGG	+	5	1	10	0.00	5.00
Chr4:100906350-100906372	100906357	5	DNA	GGCCGAGGTCGACTACC--GGCNRG	GcCCaAGGgCaACcACCGTGGCCAG	-	5	2	10	0.00	5.00
Chr6:167289319-167289341	167289336	3	RNA	GGCCGAGGTCGACTACCGGCNRG	tGCCtcGGTgGtC-ACCGGCCGG	+	5	1	10	0.00	5.00
Chr1:125171914-125171936	125171931	3	RNA	GGCCGAGGTCGACTACCGGCNRG	a--CGgGGTCGACgACgaGCGGG	+	5	2	10	0.00	5.00
Chr4:167248420-167248442	167248427	5	RNA	GGCCGAGGTCGACTACCGGCNRG	GGCgGAGG--GACagCtGtCGGG	-	5	2	10	0.00	5.00
Chr3:154208857-154208879	154208864	5	RNA	GGCCGAGGTCGACTACCGGCNRG	GGC--AGGcCaACctCCGGCCGG	-	4	2	10	0.00	5.00
Chr2:223655119-223655141	223655126	5	RNA	GGCCGAGGTCGACTACCGGCNRG	GGCacAGGaC-ACTACaGGCAGG	-	4	1	10	0.00	5.00
Chr9:141174795-141174817	141174802	5	RNA	GGCCGAGGTCGACTACCGGCNRG	GGCCc--GaCtACTACtGGtGAG	-	5	2	10	0.00	5.00
Chr3:176135769-176135791	176135776	5	RNA	GGCCGAGGTCGACTACCGGCNRG	G-aCGgGGTCGACgACgaGCGGG	-	5	1	10	0.00	5.00
Chr3:200728717-200728739	200728724	5	RNA	GGCCGAGGTCGACTACCGGCNRG	GGC--tcGcgGACTACCGcCTGG	-	5	2	10	0.00	5.00
Chr2:20386778-20386800	20386795	3	RNA	GGCCGAGGTCGACTACCGGCNRG	GGCaaAGG-CaACaACaGGCAGG	+	5	1	10	0.00	5.00

Chr1:117864051-117864073	117864068	3	RNA	GGCCGAGGTCGACTACCGGCNRG	GGCCGAGcTaGgC--CgGGCCGG	+	4	2	10	0.00	5.00
Chr1:106937550-106937572	106937567	3	RNA	GGCCGAGGTCGACTACCGGCNRG	GcCCGAGGgCaACcACC--gTGG	+	5	2	10	0.00	5.00
Chr7:178519715-178519737	178519722	5	RNA	GGCCGAGGTCGACTACCGGCNRG	GtCCG--GaaGAaTACCGGaAAG	-	5	2	10	0.00	5.00
Chr3:98731345-98731367	98731352	5	RNA	GGCCGAGGTCGACTACCGGCNRG	GtCCG--GTgcACcACCGGaCAG	-	5	2	10	0.00	5.00
Chr1:286311129-286311151	286311146	3	RNA	GGCCGAGGTCGACTACCGGCNRG	GGCgaAGGTtGAtaA--GGCGAG	+	5	2	10	0.00	5.00
Chr8:157911346-157911368	157911353	5	RNA	GGCCGAGGTCGACTACCGGCNRG	G-CCtcGGgCGAgTACtGGCAGG	-	5	1	10	0.00	5.00
Chr6:53635479-53635501	53635496	3	RNA	GGCCGAGGTCGACTACCGGCNRG	G--CGAGGgCGACgACgGctTGG	+	5	2	10	0.00	5.00
Chr6:123490784-123490806	123490791	5	RNA	GGCCGAGGTCGACTACCGGCNRG	aGCaGAG--CGAacgCCGGCTGG	-	5	2	10	0.00	5.00
Chr10:13540353-13540375	13540370	3	RNA	GGCCGAGGTCGACTACCGGCNRG	GttCGgGGT-GAgTACCtGCGGG	+	5	1	10	0.00	5.00
Chr2:195438829-195438851	195438846	3	RNA	GGCCGAGGTCGACTACCGGCNRG	aaCCGAGGcaGA--ACCGcCTAG	+	5	2	10	0.00	5.00
Chr10:39102347-39102369	39102354	5	RNA	GGCCGAGGTCGACTACCGGCNRG	GGtCGtGcTCGAC-ACgaGCTGG	-	5	1	10	0.00	5.00
Chr2:164611581-164611603	164611588	5	RNA	GGCCGAGGTCGACTACCGGCNRG	atCCtgGGT-GACcACCGGCGGG	-	5	1	10	0.00	5.00
Chr4:45652247-45652269	45652254	5	RNA	GGCCGAGGTCGACTACCGGCNRG	GGC--AGGcCGAgcACCGGCCGG	-	3	2	10	0.00	5.00
Chr6:97520676-97520698	97520693	3	RNA	GGCCGAGGTCGACTACCGGCNRG	GGCCG-GGcCGtCcACtGGCGGG	+	4	1	10	0.00	5.00
Chr7:147283183-147283205	147283200	3	RNA	GGCCGAGGTCGACTACCGGCNRG	GGC--tcGcgGAtTACCGGCTGG	+	5	2	10	0.00	5.00
Chr1:439322-439344	439329	5	DNA	GGCCGAGGTCGACTA-CCGGCNRG	GGCCGAGGcaGcCTcGCtGGCAGG	-	5	1	10	0.00	5.00
Chr1:103542857-103542879	103542874	3	DNA	GGC--CGAGGTCGACTACCGGCNRG	GGCGGCGAGtaCGAactCCGGCGAG	+	5	2	10	0.00	5.00
Chr6:140378297-140378319	140378303	4	RNA	GGCCGAGGTCGACTACCGGCNRG	caCCGAtGcCGACTg--GGCGGG	-	5	2	0	9.91	4.96
Chr4:138289321-138289343	138289336	5	DNA	GGCCGAGGTCGACTACC--GGCNRG	GGCCGAGaTCtcCTctCGAGGCCGG	+	5	2	0	9.91	4.96
Chr4:158402256-158402278	158402260	2	RNA	GGCCGAGGTCGACTACCGGCNRG	GGaCG--GTCcACgACCtGgGAG	-	5	2	0	9.91	4.96
Chr2:204784597-204784619	204784612	5	RNA	GGCCGAGGTCGACTACCGGCNRG	GcCgGAGccCGAC--CCGtCAAG	+	5	2	0	9.91	4.96
Chr9:124699320-124699342	124699326	4	DNA	GGCCGAGGTCGACT--ACCGGCNRG	GGCtcAGGctGACTGGtCCGGCTGG	-	5	2	0	9.91	4.96
Chr4:73619090-73619112	73619106	4	RNA	GGCCGAGGTCGACTACCGGCNRG	G-aCGgGGTCGACaACgaGCGGG	+	5	1	0	9.91	4.96
Chr3:174099641-174099663	174099657	4	DNA	GGCCGAGGTCGACTACCGG-CNRG	GcCgGAGGTgGACTACaGGCtCAG	+	5	1	0	9.91	4.96
Chr3:96583646-96583668	96583650	2	RNA	GGCCGAGGTCGACTACCGGCNRG	GGCCGAGG--GgtcAgCGaCTGG	-	5	2	0	9.91	4.96
Chr4:130192593-130192615	130192608	5	RNA	GGCCGAGGTCGACTACCGGCNRG	GGC-GtGtTCaACTACaGGgAAG	+	5	1	0	9.91	4.96
Chr5:93537567-93537589	93537572	3	RNA	GGCCGAGGTCGACTACCGGCNRG	GGCCGAGGcCtcCT-CaGGCTGG	-	4	1	0	9.91	4.96
Chr9:128563722-128563744	128563727	3	DNA	GGCCGAGGT--CGACTACCGGCNRG	GGgCGAGGTTGCGACTcCtccCGAG	-	5	2	0	9.91	4.96
Chr3:170087571-170087593	170087577	4	RNA	GGCCGAGGTCGACTACCGGCNRG	GaCCGAGG-CGACgACaaGaAGG	-	5	1	0	9.91	4.96
Chr4:144380215-144380237	144380233	2	RNA	GGCCGAGGTCGACTACCGGCNRG	GGCCGAGGTgGgtTgCC--tGAG	+	5	2	0	9.91	4.96
Chr10:150293314-150293336	150293331	3	DNA	GGCCGAGGT--CGACTACCGGCNRG	aGaCGAGGTTCCGACcAagGGCAGG	+	5	2	0	9.91	4.96
Chr7:152524654-152524676	152524669	5	RNA	GGCCGAGGTCGACTACCGGCNRG	GGCaGcGGTCG--TtCCcGaGGG	+	5	2	0	9.91	4.96
Chr5:115554458-115554480	115554464	4	RNA	GGCCGAGGTCGACTACCGGCNRG	GGCtGAGtTCGA--ACCctgGGG	-	5	2	0	9.91	4.96
Chr6:150089663-150089685	150089669	4	RNA	GGCCGAGGTCGACTACCGGCNRG	GaCC-AGGTCGAggctCGGCGGG	-	5	1	0	9.91	4.96
Chr5:60029242-60029264	60029259	3	RNA	GGCCGAGGTCGACTACCGGCNRG	GGCCatGGctGA--ACCcGCTAG	+	5	2	0	9.91	4.96
Chr7:67938984-67939006	67939002	2	RNA	GGCCGAGGTCGACTACCGGCNRG	GGCaGAGGa--ACTcCgGGCCGG	+	4	2	0	9.91	4.96
Chr5:205219580-205219602	205219584	2	RNA	GGCCGAGGTCGACTACCGGCNRG	GGC-GAaGTCGAagACCtcCCAG	-	5	1	0	9.91	4.96
Chr4:27992760-27992782	27992776	4	RNA	GGCCGAGGTCGACTACCGGCNRG	G-aCGgGGTCGACaACgaGCGGG	+	5	1	0	9.91	4.96
Chr1:216532936-216532958	216532954	2	RNA	GGCCGAGGTCGACTACCGGCNRG	GGCCGAacTgGgtTA--GGCTGG	+	5	2	0	9.91	4.96
Chr9:36909819-36909841	36909837	2	RNA	GGCCGAGGTCGACTACCGGCNRG	GGgaGAGGTCaAC-ACCctCAGG	+	5	1	0	9.91	4.96
Chr5:67199808-67199830	67199825	3	DNA	GGCCGAGGTCGACTACC--GGCNRG	GGCgGAGGTCGtCggCaGAGGCCGG	+	5	2	1	8.81	4.91
Chr4:68033576-68033598	68033581	3	RNA	GGCCGAGGTCGACTACCGGCNRG	tGgCtAGGa-GACTACCGGCTGG	-	4	1	7	2.75	4.88
Chr1:247722181-247722203	247722187	4	RNA	GGCCGAGGTCGACTACCGGCNRG	a-CCGtGGaCGACTACCttCCGG	-	5	1	0	9.36	4.68
Chr10:88759429-88759451	88759447	2	DNA	GGCCGAGGTCG-ACTACCGGCNRG	GGCCGAGcaCGAACcgCCGGCTGG	+	4	1	0	9.36	4.68
Chr5:213831551-213831573	213831567	4	RNA	GGCCGAGGTCGACTACCGGCNRG	GGC--AGGggGAgTtgCGGCCGG	+	5	2	0	9.36	4.68
Chr1:52136970-52136992	52136986	4	RNA	GGCCGAGGTCGACTACCGGCNRG	GG--GAGcTCGgCTggCGtCCAG	+	5	2	0	9.36	4.68
Chr2:184496767-184496789	184496782	5	RNA	GGCCGAGGTCGACTACCGGCNRG	GtCCG--GTgcACcACCGGaCAG	+	5	2	0	9.36	4.68
Chr1:228514745-228514767	228514751	4	RNA	GGCCGAGGTCGACTACCGGCNRG	GGCCGAGGcgGA-TctCGtCGAG	-	5	1	0	9.36	4.68
Chr1:2293685-2293707	2293702	3	RNA	GGCCGAGGTCGACTACCGGCNRG	G-CCcAGGTgGACgAtCtGCTGG	+	5	1	0	9.36	4.68
Chr10:16554047-16554069	16554063	4	RNA	GGCCGAGGTCGACTACCGGCNRG	G-aCGgGGTCGACaACgaGCGGG	+	5	1	0	9.36	4.68
Chr9:143499986-143500008	143500004	2	RNA	GGCCGAGGTCGACTACCGGCNRG	GG--GAcGgCGAgTgCCGaCGAG	+	5	2	0	9.36	4.68
Chr1:158090635-158090657	158090653	2	RNA	GGCCGAGGTCGACTACCGGCNRG	GGgCGAGGgCatgTAC-GGCTGG	+	5	1	0	9.36	4.68
Chr1:154432586-154432608	154432604	2	RNA	GGCCGAGGTCGACTACCGGCNRG	GGCCGAG--CGctgACaGGCGGG	+	4	2	0	9.36	4.68
Chr2:161607900-161607922	161607905	3	RNA	GGCCGAGGTCGACTACCGGCNRG	GGCCGAGGctGAaTtC--GgGGG	-	5	2	0	9.36	4.68
Chr4:64043402-64043424	64043408	4	RNA	GGCCGAGGTCGACTACCGGCNRG	GGgCGgGGTtGgCT--CGGgGAG	-	5	2	0	9.36	4.68
Chr1:146058732-146058754	146058748	4	RNA	GGCCGAGGTCGACTACCGGCNRG	G-aCGtGGTCGACaACaaGCGGG	+	5	1	0	9.36	4.68
Chr8:22695348-22695370	22695364	4	RNA	GGCCGAGGTCGACTACCGGCNRG	GGCCaAGGaaGACcAaC--CAAG	+	5	2	0	9.36	4.68
Chr1:3849157-3849179	3849172	5	RNA	GGCCGAGGTCGACTACCGGCNRG	GtaCGtGtTC--CTACCGGCCGG	+	4	2	0	9.36	4.68
Chr4:238876534-238876556	238876539	3	RNA	GGCCGAGGTCGACTACCGGCNRG	GGCCGAGtaCG--TtgCcGCAGG	-	5	2	0	9.36	4.68
Chr8:78722620-78722642	78722638	2	RNA	GGCCGAGGTCGACTACCGGCNRG	tcCCGAG--CagCTAaCGGCGAG	+	5	2	0	9.36	4.68
Chr4:144606847-144606869	144606865	2	RNA	GGCCGAGGTCGACTACCGGCNRG	GaCCGAGG-CGACgACaaGaAGG	+	5	1	0	9.36	4.68
Chr5:78742960-78742982	78742978	2	RNA	GGCCGAGGTCGACTACCGGCNRG	c--CGAGcTgGACTtCgGGCGAG	+	5	2	0	9.36	4.68
Chr8:96590069-96590091	96590074	3	X	GGCCGAGGTCGACTACCGGCNRG	GcCCaAGGaCGACatCCGGCGGG	-	5	0	0	9.36	4.68
Chr1:163321856-163321878	163321874	2	RNA	GGCCGAGGTCGACTACCGGCNRG	tGCtGAGGTtGACTgCC--tGAG	+	5	2	0	9.36	4.68
Chr2:99680837-99680859	99680854	3	RNA	GGCCGAGGTCGACTACCGGCNRG	GGCCaAG-TCcACgAaCGaCCAG	+	5	1	0	9.36	4.68
Chr2:231931085-231931107	231931091	4	RNA	GGCCGAGGTCGACTACCGGCNRG	ttCCGAtGTCGAtTg--GGCAGG	-	5	2	0	9.36	4.68
Chr5:61784087-61784109	61784093	4	RNA	GGCCGAGGTCGACTACCGGCNRG	G-aCGgGGTCGACcACgaGCGGG	-	5	1	0	9.36	4.68
Chr1:246411365-246411387	246411370	3	RNA	GGCCGAGGTCGACTACCGGCNRG	GGCCaAttTCGcCTtC--GCCGG	-	5	2	0	9.36	4.68
Chr9:87557604-87557626	87557608	2	DNA	GGCCGAG-GTCGACTACCGGCNRG	GttCGAGCGTCaACgAtCGGCAAG	-	5	1	0	9.36	4.68
Chr7:138229160-138229182	138229176	4	RNA	GGCCGAGGTCGACTACCGGCNRG	cGCCGtGaTCG--cACCaGCCAG	+	5	2	0	9.36	4.68
Chr4:139245577-139245599	139245582	3	RNA	GGCCGAGGTCGACTACCGGCNRG	GagCGAG--aGACaACCGGCTGG	-	4	2	0	9.36	4.68
Chr3:98763891-98763913	98763908	3	RNA	GGCCGAGGTCGACTACCGGCNRG	cGaCGgGGTCGgCT--CGGCTGG	+	4	2	0	9.36	4.68
Chr8:69628163-69628185	69628181	2	DNA	GGCCGAGGTCGA--CTACCGGCNRG	GcgCcAtcTCGATTCTACCGGCGAG	+	5	2	0	9.36	4.68
Chr7:11948366-11948388	11948372	4	RNA	GGCCGAGGTCGACTACCGGCNRG	GtCCG--GTgcACcACCGGaTAG	-	5	2	0	9.36	4.68
Chr7:180826397-180826419	180826403	4	DNA	GGCCGAGGTCGACTACC-GGCNRG	GGCCaAGGgCctCaACCAGGCCAG	-	5	1	0	9.36	4.68
Chr7:141948844-141948866	141948848	2	RNA	GGCCGAGGTCGACTACCGGCNRG	GtCCGAGGTC--CgAgCGGaGAG	-	4	2	0	9.36	4.68
Chr1:7417887-7417909	7417891	2	RNA	GGCCGAGGTCGACTACCGGCNRG	GGC-GcGGTCGAggACtGGtGGG	-	5	1	0	9.36	4.68
Chr6:118300599-118300621	118300605	4	RNA	GGCCGAGGTCGACTACCGGCNRG	GGC--tcGcgGACTACCGGtTGG	-	5	2	0	9.36	4.68
Chr3:77262867-77262889	77262883	4	RNA	GGCCGAGGTCGACTACCGGCNRG	G-aCGgGGTCGACaACgaGCGGG	+	5	1	0	9.36	4.68
Chr9:27097749-27097771	27097766	3	X	GGCCGAGGTCGACTACCGGCNRG	GGaCGAcGaCGACcACCaGCAGG	+	5	0	0	9.36	4.68
Chr4:151090887-151090909	151090891	2	RNA	GGCCGAGGTCGACTACCGGCNRG	GGCCGAGcaCcACgAC--GCCAG	-	4	2	0	9.36	4.68
Chr4:141042986-141043008	141043002	4	RNA	GGCCGAGGTCGACTACCGGCNRG	G-aCGgGGTCGACaACgaGCGGG	+	5	1	1	8.26	4.63
Chr2:40890230-40890252	40890248	2	DNA	GGCC-GAGGTCGACTACCGGCNRG	GcCCCGAGcTatACTACCGGCGGG	+	4	1	3	6.06	4.53
Chr3:227789873-227789895	227789878	3	RNA	GGCCGAGGTCGACTACCGGCNRG	GGCacAGGTCGtCgAa-GGCAAG	-	5	1	3	6.06	4.53
Chr1:20944079-20944101	20944096	3	RNA	GGCCGAGGTCGACTACCGGCNRG	GGC--gGGcCaACctCCGGCCGG	+	5	2	9	0.00	4.50
Chr10:53678961-53678983	53678978	3	RNA	GGCCGAGGTCGACTACCGGCNRG	GGC--tcGcgGACTACCGGCTGG	+	4	2	9	0.00	4.50
Chr7:69146911-69146933	69146918	5	RNA	GGCCGAGGTCGACTACCGGCNRG	GGCtGgGcTgGgCTA--GGCTGG	-	5	2	9	0.00	4.50
Chr3:57766932-57766954	57766949	3	RNA	GGCCGAGGTCGACTACCGGCNRG	cGCatAGGaC-ACTACaGGCAGG	+	5	1	9	0.00	4.50
Chr7:58504461-58504483	58504478	3	RNA	GGCCGAGGTCGACTACCGGCNRG	GGCCGAtcTCG--cAtCaGCAAG	+	5	2	9	0.00	4.50
Chr7:113839294-113839316	113839311	3	RNA	GGCCGAGGTCGACTACCGGCNRG	aaCCGAGGTtGgCTA--GGtCAG	+	5	2	9	0.00	4.50
Chr2:79782349-79782371	79782366	3	RNA	GGCCGAGGTCGACTACCGGCNRG	GGgCG-GGcCaACccCCGGCCGG	+	5	1	9	0.00	4.50
Chr9:149956927-149956949	149956944	3	RNA	GGCCGAGGTCGACTACCGGCNRG	GGCtGgGGTaG--cACCGGgAGG	+	5	2	9	0.00	4.50
Chr6:40897005-40897027	40897012	5	RNA	GGCCGAGGTCGACTACCGGCNRG	t--CGAGGTCaACctCaGGCGGG	-	5	2	9	0.00	4.50
Chr6:98810749-98810771	98810756	5	RNA	GGCCGAGGTCGACTACCGGCNRG	GGgCGAGcTCGACTAg-GGCTGG	-	3	1	9	0.00	4.50
Chr10:73879415-73879437	73879432	3	RNA	GGCCGAGGTCGACTACCGGCNRG	GGCCGtttTC--CcACtGGCCGG	+	5	2	9	0.00	4.50
Chr4:65946711-65946733	65946718	5	RNA	GGCCGAGGTCGACTACCGGCNRG	GGCttgGGa--ACTACCGGaAGG	-	5	2	9	0.00	4.50
Chr8:132764657-132764679	132764664	5	RNA	GGCCGAGGTCGACTACCGGCNRG	GGC--AGGcgGAgcACCGGCCGG	-	4	2	9	0.00	4.50
Chr5:160726065-160726087	160726082	3	RNA	GGCCGAGGTCGACTACCGGCNRG	tGC--AaGTacACcACCGGCCGG	+	5	2	9	0.00	4.50
Chr2:216933376-216933398	216933393	3	RNA	GGCCGAGGTCGACTACCGGCNRG	GGCCGAGGTgcAtgAC--GaAGG	+	5	2	9	0.00	4.50
Chr1:3030226-3030248	3030233	5	RNA	GGCCGAGGTCGACTACCGGCNRG	GGaCcAGtTCGACTA--aGtTAG	-	5	2	9	0.00	4.50
Chr3:162654656-162654678	162654673	3	DNA	GGCCGAGG-TCGACTACCGGCNRG	tGCCGAGGGTtGgCcAgCGGCCGG	+	5	1	9	0.00	4.50
Chr2:183134698-183134720	183134705	5	RNA	GGCCGAGGTCGACTACCGGCNRG	GcCCGAGcTtGtCT--CGGaCAG	-	5	2	9	0.00	4.50
Chr7:59259610-59259632	59259627	3	DNA	GGCC-GAGGTCGACTACCGGCNRG	GGCCCGAGGTgGAagACgGcCAGG	+	5	1	9	0.00	4.50
Chr1:59065802-59065824	59065809	5	RNA	GGCCGAGGTCGACTACCGGCNRG	GGC--tcGcgGACTAtCGGCTGG	-	5	2	9	0.00	4.50
Chr3:32260847-32260869	32260854	5	RNA	GGCCGAGGTCGACTACCGGCNRG	GGgCGAG--CGctgACaGGCAGG	-	5	2	9	0.00	4.50
Chr1:228816797-228816819	228816814	3	RNA	GGCCGAGGTCGACTACCGGCNRG	GGCCGAGtaCG--TtgCcGCAGG	+	5	2	9	0.00	4.50
Chr2:213671633-213671655	213671650	3	RNA	GGCCGAGGTCGACTACCGGCNRG	GGgCG-GGcCaACctCCGGCCGG	+	5	1	9	0.00	4.50
Chr8:129460902-129460924	129460919	3	RNA	GGCCGAGGTCGACTACCGGCNRG	GcCCGAGGgCaACcACC--aTGG	+	5	2	9	0.00	4.50
Chr8:53789705-53789727	53789722	3	RNA	GGCCGAGGTCGACTACCGGCNRG	GGCCGAGGTCGgC--CCaGgGAG	+	3	2	9	0.00	4.50
Chr5:7822135-7822157	7822142	5	DNA	GGCCGAGGTCGACTACCGG--CNRG	GGtCGAGGTCGAgTcCaaGCCCCAG	-	5	2	9	0.00	4.50
Chr5:42726464-42726486	42726481	3	RNA	GGCCGAGGTCGACTACCGGCNRG	G-aCGgGGTCGACaACgaGCGGG	+	5	1	9	0.00	4.50
Chr3:158374613-158374635	158374620	5	RNA	GGCCGAGGTCGACTACCGGCNRG	GGggGAGGTgG--TAgaGGCTGG	-	5	2	9	0.00	4.50
Chr2:159474844-159474866	159474861	3	RNA	GGCCGAGGTCGACTACCGGCNRG	cGgCGAGGTCGcCTg--GGtCGG	+	5	2	9	0.00	4.50
Chr10:126724882-126724904	126724899	3	RNA	GGCCGAGGTCGACTACCGGCNRG	GGC--tcGcgGAtTACCGGCTGG	+	5	2	9	0.00	4.50
Chr3:13873822-13873844	13873839	3	RNA	GGCCGAGGTCGACTACCGGCNRG	GGCgGAccTC--CTAgCGaCGGG	+	5	2	9	0.00	4.50
Chr4:199352157-199352179	199352164	5	RNA	GGCCGAGGTCGACTACCGGCNRG	GaCCGAGtgCtACTACCt-CCGG	-	5	1	9	0.00	4.50
Chr9:146062728-146062750	146062735	5	DNA	GGCCGAGGTCGAC--TACCGGCNRG	cGCCGAGaTCGACGCTgCgGtCGGG	-	5	2	9	0.00	4.50
Chr7:135866544-135866566	135866561	3	RNA	GGCCGAGGTCGACTACCGGCNRG	GGCCGgGGgCGgCTg--GGCCGG	+	4	2	9	0.00	4.50
Chr1:129947164-129947186	129947171	5	RNA	GGCCGAGGTCGACTACCGGCNRG	GGaatAGGTC-ACcACCGaCGAG	-	5	1	9	0.00	4.50
Chr10:148670436-148670458	148670453	3	RNA	GGCCGAGGTCGACTACCGGCNRG	cG--GAGGcCGgCgACCGGaAGG	+	5	2	9	0.00	4.50
Chr3:202339574-202339596	202339581	5	RNA	GGCCGAGGTCGACTACCGGCNRG	GGCtcAGG-CagCTACtGGCTGG	-	5	1	9	0.00	4.50
Chr2:41347030-41347052	41347047	3	RNA	GGCCGAGGTCGACTACCGGCNRG	GGaCtAGGTCG--TcCCaGtTGG	+	5	2	9	0.00	4.50
Chr8:150902144-150902166	150902151	5	RNA	GGCCGAGGTCGACTACCGGCNRG	GtCttgGGT-GACcACCGGCGGG	-	5	1	9	0.00	4.50
Chr2:57503831-57503853	57503848	3	RNA	GGCCGAGGTCGACTACCGGCNRG	GGttGAGaT--AaTACCGtCTGG	+	5	2	9	0.00	4.50
Chr4:50364334-50364356	50364351	3	RNA	GGCCGAGGTCGACTACCGGCNRG	G-CCGAGGTCGAgcACatGgTGG	+	5	1	9	0.00	4.50
Chr5:200279639-200279661	200279646	5	RNA	GGCCGAGGTCGACTACCGGCNRG	G-CCGAaGTgcACTgCCtGCTGG	-	5	1	9	0.00	4.50

Chr2:202024331-202024353	202024348	3	RNA	GGCCGAGGTCGACTACCGGCNRG	GGC--tcGcgGACTACCGtCTGG	+	5	2	9	0.00	4.50
Chr5:209656316-209656338	209656322	4	RNA	GGCCGAGGTCGACTACCGGCNRG	GGCCtcGaTCGtCTt--GGCCAG	-	5	2	0	8.81	4.41
Chr7:73014299-73014321	73014304	3	RNA	GGCCGAGGTCGACTACCGGCNRG	aGCCGAGGg-GACgACCtGCTGG	-	4	1	0	8.81	4.41
Chr10:83133264-83133286	83133279	5	RNA	GGCCGAGGTCGACTACCGGCNRG	G--CtAGGTCGcggACCGtCCGG	+	5	2	0	8.81	4.41
Chr5:188279279-188279301	188279284	3	DNA	GGCCGAGGTCG-ACTACCGGCNRG	cGaCGtGGgCGCACcACCGGCCGG	-	5	1	0	8.81	4.41
Chr10:11351215-11351237	11351220	3	RNA	GGCCGAGGTCGACTACCGGCNRG	GGCtG-cGTCGcCggCCGGCGGG	-	5	1	0	8.81	4.41
Chr8:107236058-107236080	107236063	3	RNA	GGCCGAGGTCGACTACCGGCNRG	GGC--tcGcgGACTACCGGCTGG	-	4	2	0	8.81	4.41
Chr7:92808129-92808151	92808147	2	RNA	GGCCGAGGTCGACTACCGGCNRG	GcCCaAtGcCaACTACC--CAGG	+	5	2	0	8.81	4.41
Chr6:112250306-112250328	112250324	2	RNA	GGCCGAGGTCGACTACCGGCNRG	GtCCG--GTgcACcACCGGaCAG	+	5	2	0	8.81	4.41
Chr5:147641274-147641296	147641291	3	X	GGCCGAGGTCGACTACCGGCNRG	GGaCGAGGTaGACcAgCaGCAGG	+	5	0	0	8.81	4.41
Chr4:176279491-176279513	176279498	5	RNA	GGCCGAGGTCGACTACCGGCNRG	GtCCG--GTgcACcACCGGaCAG	-	5	2	0	8.81	4.41
Chr8:78639353-78639375	78639358	3	RNA	GGCCGAGGTCGACTACCGGCNRG	G-aCGgGGTCGACgACgaGCGGG	-	5	1	0	8.81	4.41
Chr1:5942859-5942881	5942865	4	RNA	GGCCGAGGTCGACTACCGGCNRG	G-CCGAcGgCGgCTACCGGCAGG	-	3	1	0	8.81	4.41
Chr1:231826861-231826883	231826877	4	RNA	GGCCGAGGTCGACTACCGGCNRG	ttCCGActTCGACTA--tGCTGG	+	5	2	0	8.81	4.41
Chr1:160764689-160764711	160764704	5	RNA	GGCCGAGGTCGACTACCGGCNRG	cGCCGccGTCGgCTA--GtCTGG	+	5	2	0	8.81	4.41
Chr5:85731455-85731477	85731471	4	RNA	GGCCGAGGTCGACTACCGGCNRG	G-aCGgGGTCGACaACgaGCGGG	+	5	1	0	8.81	4.41
Chr2:193924808-193924830	193924814	4	RNA	GGCCGAGGTCGACTACCGGCNRG	GGCCG-GGcCatCcACtGGCGGG	-	5	1	0	8.81	4.41
Chr8:134545370-134545392	134545377	5	RNA	GGCCGAGGTCGACTACCGGCNRG	GGCCGAGGatGcCT-CgGGCTGG	-	4	1	0	8.81	4.41
Chr4:109407677-109407699	109407684	5	RNA	GGCCGAGGTCGACTACCGGCNRG	GGCCaAtcTCG--TAgCaGCAAG	-	5	2	0	8.81	4.41
Chr7:54629060-54629082	54629076	4	RNA	GGCCGAGGTCGACTACCGGCNRG	GGC-GAGGTCGgCcACCaGgGAG	+	4	1	0	8.81	4.41
Chr4:101066696-101066718	101066713	3	RNA	GGCCGAGGTCGACTACCGGCNRG	GcCCGAGGcCGA--ACtcGtGGG	+	5	2	0	8.81	4.41
Chr7:100980934-100980956	100980951	3	RNA	GGCCGAGGTCGACTACCGGCNRG	aaCaGAGGT--ACTACtGGaGAG	+	5	2	0	8.81	4.41
Chr2:205897453-205897475	205897471	2	RNA	GGCCGAGGTCGACTACCGGCNRG	GaCCGAGG-CGACgACaaGaAGG	+	5	1	0	8.81	4.41
Chr3:83773525-83773547	83773532	5	RNA	GGCCGAGGTCGACTACCGGCNRG	tGCgGAGG--GAgTACgGcCGAG	-	5	2	0	8.81	4.41
Chr7:5414793-5414815	5414808	5	RNA	GGCCGAGGTCGACTACCGGCNRG	GaC--tGGctaACTACCGGCAGG	+	5	2	0	8.81	4.41
Chr4:182848845-182848867	182848852	5	RNA	GGCCGAGGTCGACTACCGGCNRG	caCCaAGtTCGcCT-CCGGCCAG	-	5	1	0	8.81	4.41
Chr1:266828847-266828869	266828864	3	RNA	GGCCGAGGTCGACTACCGGCNRG	GGgCGAGGTaGcCgA--GGCTGG	+	4	2	0	8.81	4.41
Chr8:165249561-165249583	165249568	5	RNA	GGCCGAGGTCGACTACCGGCNRG	GGCCa--GTCGACTAtgGGgTGG	-	4	2	0	8.81	4.41
Chr2:143611350-143611372	143611357	5	RNA	GGCCGAGGTCGACTACCGGCNRG	GtCCG--GTgcACcACCGGaCAG	-	5	2	0	8.81	4.41
Chr1:17167918-17167940	17167933	5	DNA	G-GCCGAGGTCGACTACCGGCNRG	GCGCCcAGGTCGcggACCGtCCGG	+	5	1	0	8.81	4.41
Chr3:141127837-141127859	141127842	3	RNA	GGCCGAGGTCGACTACCGGCNRG	GGCCGAGtaCG--TtgCcGCAGG	-	5	2	0	8.81	4.41
Chr1:121275645-121275667	121275652	5	RNA	GGCCGAGGTCGACTACCGGCNRG	GG--GAGcaCGAactCCGGCGAG	-	5	2	7	1.65	4.33
Chr1:27905964-27905986	27905980	4	RNA	GGCCGAGGTCGACTACCGGCNRG	G-aCGgGGTCGACgACgaGCGGG	+	5	1	8	0.55	4.28
Chr4:67266523-67266545	67266541	2	DNA	GGCCGAGGT-CGACTACCGGCNRG	cGgCGAGGTACGAactCCGGCGAG	+	5	1	8	0.55	4.28
Chr8:96868831-96868853	96868848	3	RNA	GGCCGAGGTCGACTACCGGCNRG	cGCCtAGGTCGgCTgC--GCGAG	+	4	2	3	5.51	4.26
Chr2:91712420-91712442	91712425	3	DNA	GGCCGAGGTCGACTA--CCGGCNRG	ctCCaAGGcCGACcAATCCGGCAGG	-	5	2	3	5.51	4.26
Chr3:176910066-176910088	176910072	4	RNA	GGCCGAGGTCGACTACCGGCNRG	GGgCGAGcTCGACTAg-GGCTGG	-	3	1	5	3.30	4.15
Chr10:96088780-96088802	96088786	4	X	GGCCGAGGTCGACTACCGGCNRG	GaCCGAGGggGAaTACCcGCTGG	-	5	0	5	3.30	4.15
Chr6:83243418-83243440	83243425	5	DNA	GGCCGAGGTCGACTAC--CGGCNRG	GGCgGgtGTtGAaTACTACGGCTAG	-	5	2	0	8.26	4.13
Chr8:126721138-126721160	126721144	4	DNA	GGCCGAGGTCGAC-TACCGGCNRG	GGCCGAtGTCGtCGaAgCaGCAGG	-	5	1	0	8.26	4.13
Chr8:78342334-78342356	78342339	3	RNA	GGCCGAGGTCGACTACCGGCNRG	G-aCGgGGTCGACaACgaGCGGG	-	5	1	0	8.26	4.13
Chr10:23777922-23777944	23777928	4	RNA	GGCCGAGGTCGACTACCGGCNRG	GGaCcAGGTCG--TcCCaGtTGG	-	5	2	0	8.26	4.13
Chr8:84425660-84425682	84425675	5	RNA	GGCCGAGGTCGACTACCGGCNRG	G--aGcGGgaGACgACCGGCTGG	+	5	2	0	8.26	4.13
Chr5:121247559-121247581	121247574	5	RNA	GGCCGAGGTCGACTACCGGCNRG	tGaCGAGGctG--TAgCGGCAGG	+	5	2	0	8.26	4.13
Chr10:56056617-56056639	56056634	3	RNA	GGCCGAGGTCGACTACCGGCNRG	GGaCGgGcTCGACa--CGGtCGG	+	5	2	0	8.26	4.13
Chr10:92262059-92262081	92262064	3	RNA	GGCCGAGGTCGACTACCGGCNRG	GGC--AGGcCGAgcACCGGCCGG	-	3	2	0	8.26	4.13
Chr7:160511260-160511282	160511277	3	RNA	GGCCGAGGTCGACTACCGGCNRG	GaCCGAGGgaGAgTACt--CAGG	+	5	2	0	8.26	4.13
Chr1:126987078-126987100	126987096	2	RNA	GGCCGAGGTCGACTACCGGCNRG	GGC--AGGgCtACcACCcGgGGG	+	5	2	0	8.26	4.13
Chr2:3657200-3657222	3657216	4	RNA	GGCCGAGGTCGACTACCGGCNRG	G-CCGAGGcCaACctCgGGCGGG	+	5	1	0	8.26	4.13
Chr4:81670489-81670511	81670507	2	RNA	GGCCGAGGTCGACTACCGGCNRG	GGCaGAGGT-GAgcACCaGgGAG	+	5	1	0	8.26	4.13
Chr7:83863920-83863942	83863924	2	RNA	GGCCGAGGTCGACTACCGGCNRG	GcCCc--GTCGAgctCCGGCGAG	-	5	2	0	8.26	4.13
Chr1:8847411-8847433	8847417	4	RNA	GGCCGAGGTCGACTACCGGCNRG	GGaCGAGGaCGAtgAC--GaCGG	-	5	2	0	8.26	4.13
Chr1:168799184-168799206	168799201	3	DNA	GGC-CGAGGTCGACTACCGGCNRG	GcCTCGAGGaCGtCTtCCGGCTGG	+	4	1	0	8.26	4.13
Chr3:28133551-28133573	28133567	4	RNA	GGCCGAGGTCGACTACCGGCNRG	GGaCGgGGTCa--gACCGGCCGG	+	4	2	0	8.26	4.13
Chr10:85106046-85106068	85106053	5	RNA	GGCCGAGGTCGACTACCGGCNRG	GGC--AGGcCaACctCCGGCCGG	-	4	2	0	8.26	4.13
Chr1:93460470-93460492	93460477	5	RNA	GGCCGAGGTCGACTACCGGCNRG	GGgCGAc--CGAagtCCGGCGAG	-	5	2	0	8.26	4.13
Chr10:87882332-87882354	87882347	5	RNA	GGCCGAGGTCGACTACCGGCNRG	GGCtcAcG--GACTACCGGtTGG	+	4	2	0	8.26	4.13
Chr4:228199996-228200018	228200011	5	RNA	GGCCGAGGTCGACTACCGGCNRG	GGCgGAGaTCGtCTtCC--gTGG	+	5	2	0	8.26	4.13
Chr5:148957471-148957493	148957477	4	RNA	GGCCGAGGTCGACTACCGGCNRG	GGtgGAGGcCGcCTgCC--CCGG	-	5	2	0	8.26	4.13
Chr6:96825790-96825812	96825797	5	DNA	GGC-CGAGGTCGACTACCGGCNRG	GcCTCGgGGaCGtCTtCCGGCTGG	-	5	1	0	8.26	4.13
Chr9:25819071-25819093	25819086	5	RNA	GGCCGAGGTCGACTACCGGCNRG	GGC--ttGcgGACTAtCGGCTGG	+	5	2	0	8.26	4.13
Chr1:286677288-286677310	286677304	4	RNA	GGCCGAGGTCGACTACCGGCNRG	cGCCGAGG-CaACTAgtGcCCAG	+	5	1	0	8.26	4.13
Chr5:190521127-190521149	190521134	5	RNA	GGCCGAGGTCGACTACCGGCNRG	GcCgGAGccCGAC--CCGtCAGG	-	5	2	0	8.26	4.13
Chr5:86294324-86294346	86294339	5	RNA	GGCCGAGGTCGACTACCGGCNRG	tGgCt-cGTgGACTACCGGCTGG	+	5	1	0	8.26	4.13
Chr6:130743476-130743498	130743494	2	RNA	GGCCGAGGTCGACTACCGGCNRG	G-CCGgGtTCGACTcCgGGtCAG	+	5	1	0	8.26	4.13
Chr4:128461665-128461687	128461670	3	RNA	GGCCGAGGTCGACTACCGGCNRG	GGaCGAGGag--CaACgGGCGAG	-	5	2	0	8.26	4.13
Chr5:129459716-129459738	129459732	4	RNA	GGCCGAGGTCGACTACCGGCNRG	G-aCGgGGTCGACaACgaGCGGG	+	5	1	0	8.26	4.13
Chr1:274397528-274397550	274397532	2	RNA	GGCCGAGGTCGACTACCGGCNRG	GGCC-AGGcCGcCggCCGGCCGG	-	4	1	0	8.26	4.13
Chr10:37323842-37323864	37323857	5	RNA	GGCCGAGGTCGACTACCGGCNRG	GGCgGccGcCG--cACCGGCTGG	+	5	2	0	8.26	4.13
Chr9:12599199-12599221	12599206	5	RNA	GGCCGAGGTCGACTACCGGCNRG	GGCCGccGT-GAgTACaGGtGAG	-	5	1	0	8.26	4.13
Chr7:80448651-80448673	80448669	2	DNA	GGC--CGAGGTCGACTACCGGCNRG	GaCAACGgGGTCGACgACgaGCGGG	+	5	2	0	8.26	4.13
Chr8:95012880-95012902	95012886	4	RNA	GGCCGAGGTCGACTACCGGCNRG	GtCCtcGG--GAtaACCGGCAGG	-	5	2	0	8.26	4.13
Chr8:142802791-142802813	142802796	3	RNA	GGCCGAGGTCGACTACCGGCNRG	G-aCGAGGTCaACaACgaGCGGG	-	5	1	0	8.26	4.13
Chr3:28930689-28930711	28930707	2	RNA	GGCCGAGGTCGACTACCGGCNRG	GtCCG--GTgcACcACCGGaTAG	+	5	2	0	8.26	4.13
Chr3:89804446-89804468	89804452	4	RNA	GGCCGAGGTCGACTACCGGCNRG	GGC--ttGTgGACTACCGGCTGG	-	3	2	0	8.26	4.13
Chr2:60165521-60165543	60165538	3	DNA	G--GCCGAGGTCGACTACCGGCNRG	GATGaCGgGGTCGACaACgaGCGGG	+	5	2	0	8.26	4.13
Chr4:237728625-237728647	237728643	2	RNA	GGCCGAGGTCGACTACCGGCNRG	GGgCGAGGgCt--TgCCGGtGAG	+	5	2	0	8.26	4.13
Chr6:162934695-162934717	162934701	4	DNA	GGC-CGAGGTCGACTACCGGCNRG	GcCTCGAGGaCatCTtCCGGCTGG	-	5	1	0	8.26	4.13
Chr1:223867058-223867080	223867073	5	DNA	GGC--CGAGGTCGACTACCGGCNRG	GGCCTCGgGGgCtACacCCGGCGGG	+	5	2	0	8.26	4.13
Chr9:147054948-147054970	147054964	4	RNA	GGCCGAGGTCGACTACCGGCNRG	G-aCGgGGTCGACgACaaGCGGG	+	5	1	0	8.26	4.13
Chr7:111060700-111060722	111060717	3	RNA	GGCCGAGGTCGACTACCGGCNRG	GtCCG--GTgcACcACCGGaCAG	+	5	2	0	8.26	4.13
Chr6:163883708-163883730	163883726	2	RNA	GGCCGAGGTCGACTACCGGCNRG	GaCaGAGGgCG--cACCGGaCAG	+	5	2	0	8.26	4.13
Chr8:104410705-104410727	104410711	4	RNA	GGCCGAGGTCGACTACCGGCNRG	GcCgGAGccCGAC--CCGtCAAG	-	5	2	0	8.26	4.13
Chr5:205212791-205212813	205212807	4	RNA	GGCCGAGGTCGACTACCGGCNRG	G-CCGAaGTacACcACCtGCTGG	+	5	1	0	8.26	4.13
Chr10:15875069-15875091	15875074	3	RNA	GGCCGAGGTCGACTACCGGCNRG	GaCaGAGGTCGAC-gtCaGCAGG	-	5	1	0	8.26	4.13
Chr7:43839142-43839164	43839147	3	RNA	GGCCGAGGTCGACTACCGGCNRG	GcCCGAGGcCGA--ACttGgGGG	-	5	2	1	7.16	4.08
Chr6:138881986-138882008	138881993	5	DNA	GGCCGAGGTCGAC-TACCGGCNRG	GGCgGAGGgCGcCTTAggGGCGGG	-	5	1	8	0.00	4.00
Chr1:137743674-137743696	137743691	3	RNA	GGCCGAGGTCGACTACCGGCNRG	GGCCGAGG--GgagtCCGGCGGG	+	4	2	8	0.00	4.00
Chr5:54424582-54424604	54424589	5	RNA	GGCCGAGGTCGACTACCGGCNRG	cGCCGAtGa--ACcACgGGCGGG	-	5	2	8	0.00	4.00
Chr7:25947108-25947130	25947115	5	RNA	GGCCGAGGTCGACTACCGGCNRG	GcCCGAGGTgGcCT-CgGcCTAG	-	5	1	8	0.00	4.00
Chr2:205493225-205493247	205493242	3	RNA	GGCCGAGGTCGACTACCGGCNRG	GtCttgGGT-GACcACCGGCGAG	+	5	1	8	0.00	4.00
Chr6:135951500-135951522	135951517	3	RNA	GGCCGAGGTCGACTACCGGCNRG	GGaCaAGG--GACTAgaGaCTAG	+	5	2	8	0.00	4.00
Chr10:52256073-52256095	52256080	5	RNA	GGCCGAGGTCGACTACCGGCNRG	GGCCGgG--CcACTggCGGaAGG	-	5	2	8	0.00	4.00
Chr8:20984843-20984865	20984850	5	RNA	GGCCGAGGTCGACTACCGGCNRG	GtCttgGGT-GACcACCGGCGGG	-	5	1	8	0.00	4.00
Chr10:122462295-122462317	122462312	3	RNA	GGCCGAGGTCGACTACCGGCNRG	GG--GAcGcCGAggACCGGgGAG	+	5	2	8	0.00	4.00
Chr3:126204066-126204088	126204083	3	RNA	GGCCGAGGTCGACTACCGGCNRG	a--CGgGGTCGACaACgaGCGGG	+	5	2	8	0.00	4.00
Chr3:76958152-76958174	76958159	5	RNA	GGCCGAGGTCGACTACCGGCNRG	GGCCGAGGaCG--TgCgGctGGG	-	5	2	8	0.00	4.00
Chr9:65249312-65249334	65249329	3	RNA	GGCCGAGGTCGACTACCGGCNRG	G--CGAGGTgGACggCgGGgTGG	+	5	2	8	0.00	4.00
Chr1:205586244-205586266	205586251	5	RNA	GGCCGAGGTCGACTACCGGCNRG	GGC-GgGGTCGACaACgaGCGGG	-	4	1	8	0.00	4.00
Chr1:270302293-270302315	270302310	3	RNA	GGCCGAGGTCGACTACCGGCNRG	G--CGAGGaCGAggACgGGaCAG	+	5	2	8	0.00	4.00
Chr4:179369305-179369327	179369312	5	DNA	GGCCGAGGTCGAC--TACCGGCNRG	aGCCGAGGTCGAgCTTAgCatCGGG	-	5	2	8	0.00	4.00
Chr2:97653979-97654001	97653996	3	DNA	GGCCGAGGTCGACT--ACCGGCNRG	GcCCGAcaTCGcCTACACCGtCCAG	+	5	2	8	0.00	4.00
Chr1:254800155-254800177	254800162	5	RNA	GGCCGAGGTCGACTACCGGCNRG	G--CGtGGcCGACTgCCtGaTGG	-	5	2	8	0.00	4.00
Chr8:74268261-74268283	74268268	5	DNA	GGCCGAGGTCGACTA--CCGGCNRG	ctCCaAGGcCGACcAATCCGGCAGG	-	5	2	8	0.00	4.00
Chr2:12385408-12385430	12385415	5	RNA	GGCCGAGGTCGACTACCGGCNRG	G-aCGgGGTCGACaACgaGCGGG	-	5	1	8	0.00	4.00
Chr1:61400776-61400798	61400783	5	RNA	GGCCGAGGTCGACTACCGGCNRG	GtCCG--GTgcACcACCGGaTAG	-	5	2	8	0.00	4.00
Chr7:60861832-60861854	60861839	5	DNA	GGCCGAGGTCGACTACCG-GCNRG	cGCCaccGTCGtCTACCGCGCCGG	-	5	1	8	0.00	4.00
Chr1:94763135-94763157	94763152	3	RNA	GGCCGAGGTCGACTACCGGCNRG	cGgtGAGcTtG--TACCGGCCAG	+	5	2	8	0.00	4.00
Chr9:128187526-128187548	128187533	5	RNA	GGCCGAGGTCGACTACCGGCNRG	GGgCGAGG-CGcaTctCGGCAAG	-	5	1	8	0.00	4.00
Chr1:158127319-158127341	158127326	5	DNA	GGC-CGAGGTCGACTACCGGCNRG	GcCTCGAGGaCGtCTtCCGGCTGG	-	4	1	8	0.00	4.00
Chr3:145198993-145199015	145199010	3	X	GGCCGAGGTCGACTACCGGCNRG	GGCCGAGGTCGACTtCgGGCGAG	+	2	0	8	0.00	4.00
Chr4:120988901-120988923	120988918	3	RNA	GGCCGAGGTCGACTACCGGCNRG	G--CGAcGaCGACgACgGGgGGG	+	5	2	8	0.00	4.00
Chr2:12705817-12705839	12705834	3	RNA	GGCCGAGGTCGACTACCGGCNRG	GGCCaAGcgCG--gACCGtCCGG	+	5	2	8	0.00	4.00
Chr4:221567798-221567820	221567805	5	RNA	GGCCGAGGTCGACTACCGGCNRG	GGCCGAGGgtGcCT-CgGGCTGG	-	4	1	8	0.00	4.00
Chr3:84288772-84288794	84288789	3	RNA	GGCCGAGGTCGACTACCGGCNRG	GGCCGtGtTCatCTtCC--CGAG	+	5	2	8	0.00	4.00
Chr4:103530190-103530212	103530207	3	DNA	GGCCGAGGTCGACTACC--GGCNRG	GGCgGAGGTCGtCagCaGAGGCCGG	+	5	2	8	0.00	4.00
Chr3:214293275-214293297	214293292	3	RNA	GGCCGAGGTCGACTACCGGCNRG	GGCCGAGtaCG--TtgCcGCAGG	+	5	2	8	0.00	4.00
Chr5:82998323-82998345	82998330	5	RNA	GGCCGAGGTCGACTACCGGCNRG	GaagGAGGTCGtCgA--GGCCGG	-	5	2	8	0.00	4.00
Chr8:130151606-130151628	130151623	3	RNA	GGCCGAGGTCGACTACCGGCNRG	GGaCGAGGaCGAggA--GGgGAG	+	5	2	8	0.00	4.00
Chr6:125230154-125230176	125230161	5	RNA	GGCCGAGGTCGACTACCGGCNRG	GctCGAGcTCGACT--CtGaTGG	-	5	2	8	0.00	4.00
Chr10:91548707-91548729	91548714	5	RNA	GGCCGAGGTCGACTACCGGCNRG	GGaCcAGGTCG--TcCCaGtTGG	-	5	2	8	0.00	4.00
Chr5:63299889-63299911	63299906	3	RNA	GGCCGAGGTCGACTACCGGCNRG	cGCCGcGGaCGACTcaC--CGAG	+	5	2	8	0.00	4.00
Chr1:276234159-276234181	276234166	5	RNA	GGCCGAGGTCGACTACCGGCNRG	aGCgaAGG--GACTAatGGCGAG	-	5	2	8	0.00	4.00
Chr8:21667646-21667668	21667663	3	RNA	GGCCGAGGTCGACTACCGGCNRG	GGgCG-GGcCaACctCCGGCCGG	+	5	1	8	0.00	4.00
Chr5:65294307-65294329	65294324	3	RNA	GGCCGAGGTCGACTACCGGCNRG	GGCtGgGcTgGgCTA--GGCTGG	+	5	2	8	0.00	4.00
Chr2:171195536-171195558	171195543	5	RNA	GGCCGAGGTCGACTACCGGCNRG	GGCCGgGGcgGgC--CgGGCCGG	-	5	2	8	0.00	4.00

Chr8:18781622-18781644	18781629	5	RNA	GGCCGAGGTCGACTACCGGCNRG	GGCCaAGcgCG--gACCGtCCGG	-	5	2	8	0.00	4.00
Chr10:110702323-110702345	110702340	3	DNA	GGC-CGAGGTCGACTACCGGCNRG	GcCTCGAGGgCtACacCCGGCGGG	+	5	1	8	0.00	4.00
Chr5:161945647-161945669	161945654	5	DNA	GGCCGAGGT-CGACTACCGGCNRG	GGCCGAGGTGCctCTtCCaGtCAG	-	5	1	8	0.00	4.00
Chr1:194552141-194552163	194552158	3	RNA	GGCCGAGGTCGACTACCGGCNRG	GG--GAGGagGtaTACgGGCAAG	+	5	2	8	0.00	4.00
Chr5:4113674-4113696	4113681	5	RNA	GGCCGAGGTCGACTACCGGCNRG	GGCCGAGaTgtAtcAC--GCAGG	-	5	2	8	0.00	4.00
Chr8:41479679-41479701	41479686	5	RNA	GGCCGAGGTCGACTACCGGCNRG	GGCCtAcaTCtACT-CCGGCGAG	-	4	1	8	0.00	4.00
Chr10:91548575-91548597	91548592	3	RNA	GGCCGAGGTCGACTACCGGCNRG	GGC--tcGcgGACTACCGGCTGG	+	4	2	8	0.00	4.00
Chr5:11905700-11905722	11905707	5	DNA	GGCCGAGGTCGACT-ACCGGCNRG	cGCCGcGGatGACTCACCGGaCAG	-	5	1	8	0.00	4.00
Chr6:3324436-3324458	3324443	5	DNA	GGCCGAGGTCGA-CTACCGGCNRG	GGCCGAGGTCGAGCTcgaGGCTGG	-	3	1	8	0.00	4.00
Chr5:102936921-102936943	102936938	3	RNA	GGCCGAGGTCGACTACCGGCNRG	GGCCGgGcTgaACT-CCGaCTAG	+	5	1	0	7.71	3.86
Chr10:59019062-59019084	59019067	3	DNA	GGC--CGAGGTCGACTACCGGCNRG	GaCAACGgGGTCGACgACgaGCGGG	-	5	2	0	7.71	3.86
Chr5:22797751-22797773	22797758	5	DNA	GGCCGAGGTCGACTA-CCGGCNRG	GGtgGAGGTtGtCTcTCCGGCGAG	-	5	1	0	7.71	3.86
Chr2:209209610-209209632	209209628	2	RNA	GGCCGAGGTCGACTACCGGCNRG	aGCCGgGG--GAaTcCCtGCGGG	+	5	2	0	7.71	3.86
Chr2:42120543-42120565	42120561	2	RNA	GGCCGAGGTCGACTACCGGCNRG	GGaCcAGGTgG--TtCgGGCGAG	+	5	2	0	7.71	3.86
Chr10:150122156-150122178	150122160	2	RNA	GGCCGAGGTCGACTACCGGCNRG	aGCCGAGGTCGACTgg-GGgCGG	-	4	1	0	7.71	3.86
Chr10:63152603-63152625	63152609	4	RNA	GGCCGAGGTCGACTACCGGCNRG	GGC--tcGcgGACTACCGGCTGG	-	4	2	0	7.71	3.86
Chr3:188615147-188615169	188615151	2	RNA	GGCCGAGGTCGACTACCGGCNRG	aGCCGAcGTaG-CcACtGGCGAG	-	5	1	0	7.71	3.86
Chr5:111129257-111129279	111129262	3	DNA	GGC--CGAGGTCGACTACCGGCNRG	GGCCTCGgGGgCtACacCCGGCGGG	-	5	2	0	7.71	3.86
Chr3:147343929-147343951	147343946	3	RNA	GGCCGAGGTCGACTACCGGCNRG	cGaCGgGGTCGACgAC--GaGAG	+	5	2	0	7.71	3.86
Chr5:205698867-205698889	205698872	3	RNA	GGCCGAGGTCGACTACCGGCNRG	GaCaG-GGTCGACgACgaGCGGG	-	5	1	0	7.71	3.86
Chr4:171574757-171574779	171574762	3	RNA	GGCCGAGGTCGACTACCGGCNRG	G-aCGgGGTCGACaACgaGCGGG	-	5	1	0	7.71	3.86
Chr10:97282160-97282182	97282178	2	RNA	GGCCGAGGTCGACTACCGGCNRG	GG--GAGGTCGggTtgCGGgAGG	+	5	2	0	7.71	3.86
Chr7:10258808-10258830	10258823	5	RNA	GGCCGAGGTCGACTACCGGCNRG	tGCtGAGGTgGAtTACC--tGAG	+	5	2	0	7.71	3.86
Chr10:129800836-129800858	129800841	3	RNA	GGCCGAGGTCGACTACCGGCNRG	GGC--tcGcgGACTACCGGCTGG	-	4	2	0	7.71	3.86
Chr10:147635855-147635877	147635870	5	RNA	GGCCGAGGTCGACTACCGGCNRG	GGC--tcGcgGACTACCGcCTGG	+	5	2	0	7.71	3.86
Chr1:100983773-100983795	100983777	2	RNA	GGCCGAGGTCGACTACCGGCNRG	tGCCGAGGTCG--TAtgGctGGG	-	5	2	0	7.71	3.86
Chr10:139168803-139168825	139168807	2	DNA	GGCCGAGGTCGACTACCGG--CNRG	GGCCGAGGTCGAggcCgaGCCCTGG	-	5	2	0	7.71	3.86
Chr2:66966821-66966843	66966825	2	RNA	GGCCGAGGTCGACTACCGGCNRG	GGC--AGcTCGAggACgaGCTGG	-	5	2	0	7.71	3.86
Chr3:212883751-212883773	212883766	5	RNA	GGCCGAGGTCGACTACCGGCNRG	GGCtcAcG--GACTACCGGCTGG	+	3	2	0	7.71	3.86
Chr5:93592105-93592127	93592110	3	RNA	GGCCGAGGTCGACTACCGGCNRG	GGCCGtcGgC--CTgCCcGCCGG	-	5	2	0	7.71	3.86
Chr5:148721181-148721203	148721186	3	RNA	GGCCGAGGTCGACTACCGGCNRG	GGCCGtGGTaGA--AaCcGtCGG	-	5	2	0	7.71	3.86
Chr2:35713287-35713309	35713302	5	RNA	GGCCGAGGTCGACTACCGGCNRG	GGCttgGGa--ACTACCGGaAGG	+	5	2	0	7.71	3.86
Chr5:123207231-123207253	123207235	2	RNA	GGCCGAGGTCGACTACCGGCNRG	GGCtatGGTCaA--ACCGaCGGG	-	5	2	0	7.71	3.86
Chr2:78413225-78413247	78413231	4	DNA	GGC--CGAGGTCGACTACCGGCNRG	GGCCTCGgGGgCtACacCCGGCGGG	-	5	2	0	7.71	3.86
Chr3:39499815-39499837	39499820	3	RNA	GGCCGAGGTCGACTACCGGCNRG	GGCaGtGGT--ACTcCgGtCGAG	-	5	2	0	7.71	3.86
Chr1:171522793-171522815	171522810	3	DNA	GGC-CGAGGTCGACTACCGGCNRG	GcCTCGgGGaCGtCTtCCGGCTGG	+	5	1	0	7.71	3.86
Chr10:128767734-128767756	128767741	5	RNA	GGCCGAGGTCGACTACCGGCNRG	GGgCG-GGcCaACctCCGGCCGG	-	5	1	0	7.71	3.86
Chr2:182828126-182828148	182828131	3	RNA	GGCCGAGGTCGACTACCGGCNRG	GtCCG--GTgcACcACCGGaCAG	-	5	2	0	7.71	3.86
Chr2:126913098-126913120	126913103	3	RNA	GGCCGAGGTCGACTACCGGCNRG	cGCCGAaG-CtACcACCGGaTGG	-	5	1	0	7.71	3.86
Chr3:209871649-209871671	209871655	4	DNA	GGCCGAGGTCGACTA--CCGGCNRG	GGgCGgGGTCGgCgAAGCaGGCCAG	-	5	2	0	7.71	3.86
Chr3:57466224-57466246	57466241	3	RNA	GGCCGAGGTCGACTACCGGCNRG	GaCCcAaGTCaACTACt--CAGG	+	5	2	0	7.71	3.86
Chr1:102297391-102297413	102297397	4	RNA	GGCCGAGGTCGACTACCGGCNRG	GaCCGAGGaCttCTACC--aTGG	-	5	2	0	7.71	3.86
Chr1:129682246-129682268	129682251	3	RNA	GGCCGAGGTCGACTACCGGCNRG	GGCCGgGGatACT-CtGGCGAG	-	5	1	0	7.71	3.86
Chr7:52124860-52124882	52124878	2	RNA	GGCCGAGGTCGACTACCGGCNRG	GGCgGcGGTgG--TgCtGGCGGG	+	5	2	0	7.71	3.86
Chr10:53136815-53136837	53136822	5	RNA	GGCCGAGGTCGACTACCGGCNRG	caCCaAGtTCGcCT-CCGGCTAG	-	5	1	0	7.71	3.86
Chr3:83227638-83227660	83227644	4	RNA	GGCCGAGGTCGACTACCGGCNRG	GGCCGAtcTCG--cAtCaGCAAG	-	5	2	0	7.71	3.86
Chr2:145347124-145347146	145347139	5	RNA	GGCCGAGGTCGACTACCGGCNRG	GtCgGActTCGACaAC--GCGGG	+	5	2	0	7.71	3.86
Chr7:167250194-167250216	167250199	3	RNA	GGCCGAGGTCGACTACCGGCNRG	GGCgGAGG--GACagCtGtCGGG	-	5	2	0	7.71	3.86
Chr5:201556778-201556800	201556785	5	RNA	GGCCGAGGTCGACTACCGGCNRG	GGgCaAGGgCGACgtCC--CCGG	-	5	2	0	7.71	3.86
Chr6:159401411-159401433	159401416	3	RNA	GGCCGAGGTCGACTACCGGCNRG	G-aCGgGGTCGACgACgaGCGGG	-	5	1	0	7.71	3.86
Chr10:41545147-41545169	41545152	3	DNA	GGCCGAGGTCGACTACC--GGCNRG	GcCCGAGagCaACcACCGTGGCCAG	-	5	2	0	7.71	3.86
Chr8:21977857-21977879	21977875	2	DNA	GGCCGAGG-TCGACTACCGGCNRG	GaCCcgGGTTCGAtTcCCGGCTGG	+	5	1	0	7.71	3.86
Chr7:41944476-41944498	41944481	3	RNA	GGCCGAGGTCGACTACCGGCNRG	GtaCG--GTtGAaTACtGGCCGG	-	5	2	0	7.71	3.86
Chr4:79739608-79739630	79739623	5	RNA	GGCCGAGGTCGACTACCGGCNRG	GGgCGgGGTtGgCT--CGGgGAG	+	5	2	0	7.71	3.86
Chr7:20017349-20017371	20017365	4	RNA	GGCCGAGGTCGACTACCGGCNRG	GtCaGActTCGACaAC--GCAGG	+	5	2	0	7.71	3.86
Chr5:132284412-132284434	132284430	2	RNA	GGCCGAGGTCGACTACCGGCNRG	tGCaGAGcTCGgCTA--GGCTGG	+	4	2	0	7.71	3.86
Chr6:83985168-83985190	83985172	2	RNA	GGCCGAGGTCGACTACCGGCNRG	GGaCGAGG--GtCcgCCGGgAAG	-	5	2	0	7.71	3.86
Chr6:86858920-86858942	86858937	3	DNA	GGC-CGAGGTCGACTACCGGCNRG	GGCTCGAatTCtACcACCGGCTGG	+	4	1	0	7.71	3.86
Chr10:28362556-28362578	28362562	4	RNA	GGCCGAGGTCGACTACCGGCNRG	aGCCtcGGgCGACTACt--CTAG	-	5	2	0	7.71	3.86
Chr2:39860358-39860380	39860363	3	RNA	GGCCGAGGTCGACTACCGGCNRG	GGtCcAGGgC--CTtCCtGCGGG	-	5	2	0	7.71	3.86
Chr4:244201160-244201182	244201165	3	RNA	GGCCGAGGTCGACTACCGGCNRG	G-aCGgGGTCGACcACgaGCGGG	-	5	1	0	7.71	3.86
Chr1:259160861-259160883	259160868	5	RNA	GGCCGAGGTCGACTACCGGCNRG	GGC--tcGcaGACTACtGGCTGG	-	5	2	0	7.71	3.86
Chr9:156236554-156236576	156236570	4	RNA	GGCCGAGGTCGACTACCGGCNRG	aGCCGAGGgtG-CcACaGGCTGG	+	5	1	0	7.71	3.86
Chr10:132199051-132199073	132199058	5	RNA	GGCCGAGGTCGACTACCGGCNRG	G--CGAGGTCGgCggCCGcgAAG	-	5	2	0	7.71	3.86
Chr6:146032127-146032149	146032132	3	RNA	GGCCGAGGTCGACTACCGGCNRG	G-aCGgGGTCGACgACgaGCGGG	-	5	1	0	7.71	3.86
Chr3:117491968-117491990	117491986	2	DNA	GGC--CGAGGTCGACTACCGGCNRG	GGCCTCGAGGgCtACacCCGGCGGG	+	4	2	0	7.71	3.86
Chr9:119038444-119038466	119038448	2	DNA	GGCCGAGGTCGACTACC--GGCNRG	cGCCGccGTCtACTACCGCGcCGGG	-	5	2	0	7.71	3.86
Chr4:21100458-21100480	21100462	2	RNA	GGCCGAGGTCGACTACCGGCNRG	GGCCcAGGTgtAC-AatGGCGGG	-	5	1	0	7.71	3.86
Chr6:165163533-165163555	165163551	2	DNA	G-GCCGAGGTCGACTACCGGCNRG	GCGCCtcGGgCGAgTACtGGCAGG	+	5	1	0	7.71	3.86
Chr9:133600039-133600061	133600054	5	RNA	GGCCGAGGTCGACTACCGGCNRG	GGC--ttGcgGACTACCGGCTGG	+	4	2	6	1.65	3.83
Chr3:216843243-216843265	216843261	2	RNA	GGCCGAGGTCGACTACCGGCNRG	GGC--gGGTaGACgAgCaGCAAG	+	5	2	1	6.61	3.81
Chr4:142295394-142295416	142295400	4	RNA	GGCCGAGGTCGACTACCGGCNRG	GGC--AGGcCGAgcACCGGCCGG	-	3	2	7	0.55	3.78
Chr6:71670360-71670382	71670365	3	RNA	GGCCGAGGTCGACTACCGGCNRG	G-CCGgaGTCGACTgCCGGtGAG	-	4	1	7	0.55	3.78
Chr10:123030488-123030510	123030494	4	RNA	GGCCGAGGTCGACTACCGGCNRG	tG--GAGGTtGcCaACCGGaTAG	-	5	2	2	5.51	3.76
Chr3:129365996-129366018	129366011	5	RNA	GGCCGAGGTCGACTACCGGCNRG	GGgC-AGGcCGAgcACCGGCCGG	+	4	1	0	7.16	3.58
Chr7:22380372-22380394	22380387	5	RNA	GGCCGAGGTCGACTACCGGCNRG	a--CGgGGTCGACgACgaGCGGG	+	5	2	0	7.16	3.58
Chr9:91636078-91636100	91636094	4	RNA	GGCCGAGGTCGACTACCGGCNRG	G-aCGgGGTCGACaACgaGCGGG	+	5	1	0	7.16	3.58
Chr2:8288552-8288574	8288570	2	RNA	GGCCGAGGTCGACTACCGGCNRG	GaCCGAGG-CGACgACaaGaAGG	+	5	1	0	7.16	3.58
Chr6:349520-349542	349527	5	RNA	GGCCGAGGTCGACTACCGGCNRG	GcCCGAGGgCaACcACC--gTGG	-	5	2	0	7.16	3.58
Chr7:70339169-70339191	70339174	3	RNA	GGCCGAGGTCGACTACCGGCNRG	G-aCGgGGTCGACaACgaGCGGG	-	5	1	0	7.16	3.58
Chr6:144446673-144446695	144446690	3	RNA	GGCCGAGGTCGACTACCGGCNRG	aGt--tGGTgGAaTACCGGCAAG	+	5	2	0	7.16	3.58
Chr7:133222422-133222444	133222428	4	RNA	GGCCGAGGTCGACTACCGGCNRG	GGgCG-GGcCaACcACCGGCCGG	-	4	1	0	7.16	3.58
Chr2:216374341-216374363	216374347	4	RNA	GGCCGAGGTCGACTACCGGCNRG	GGCgGcGGTCGAC-AtCtcCCGG	-	5	1	0	7.16	3.58
Chr3:163017842-163017864	163017858	4	RNA	GGCCGAGGTCGACTACCGGCNRG	G-aCGgGGTCGACaACgaGCAGG	+	5	1	0	7.16	3.58
Chr4:28897450-28897472	28897455	3	RNA	GGCCGAGGTCGACTACCGGCNRG	tGCCGAGGTCG--TAtgGctGGG	-	5	2	0	7.16	3.58
Chr7:171206166-171206188	171206184	2	RNA	GGCCGAGGTCGACTACCGGCNRG	GGCCGAGcgCGA--AggGGgAAG	+	5	2	0	7.16	3.58
Chr4:70715583-70715605	70715601	2	RNA	GGCCGAGGTCGACTACCGGCNRG	GGCgGAGGTCGttTggC--CGAG	+	5	2	0	7.16	3.58
Chr10:9074274-9074296	9074290	4	RNA	GGCCGAGGTCGACTACCGGCNRG	GaCaG-GGTCGACgACaaGCGGG	+	5	1	0	7.16	3.58
Chr1:87264724-87264746	87264741	3	RNA	GGCCGAGGTCGACTACCGGCNRG	GaCCGAGGagGgCT-CtGGCGAG	+	5	1	0	7.16	3.58
Chr7:149461770-149461792	149461775	3	RNA	GGCCGAGGTCGACTACCGGCNRG	cGCCGA--TCGAtTtCCGGCAGG	-	3	2	0	7.16	3.58
Chr10:67516012-67516034	67516018	4	RNA	GGCCGAGGTCGACTACCGGCNRG	GtCCG--GTgcACcACCGGaCAG	-	5	2	0	7.16	3.58
Chr5:172175136-172175158	172175141	3	RNA	GGCCGAGGTCGACTACCGGCNRG	GGC--tcGcgGACTACCGaCTGG	-	5	2	0	7.16	3.58
Chr7:30880933-30880955	30880938	3	RNA	GGCCGAGGTCGACTACCGGCNRG	GaCaG-GGTCGACgACaaGCGGG	-	5	1	0	7.16	3.58
Chr6:158971260-158971282	158971276	4	RNA	GGCCGAGGTCGACTACCGGCNRG	GGa--AaGTCGACTAtCtcCAAG	+	5	2	0	7.16	3.58
Chr4:182081075-182081097	182081080	3	RNA	GGCCGAGGTCGACTACCGGCNRG	GaCgGgGGgCGA--ACCGGCCGG	-	4	2	0	7.16	3.58
Chr7:34243873-34243895	34243879	4	RNA	GGCCGAGGTCGACTACCGGCNRG	GGCatAGGaC-ACTACaGGCAGG	-	4	1	0	7.16	3.58
Chr7:50901975-50901997	50901993	2	RNA	GGCCGAGGTCGACTACCGGCNRG	GGC-GAcGTtGACTACCacgAGG	+	5	1	0	7.16	3.58
Chr5:153506362-153506384	153506378	4	RNA	GGCCGAGGTCGACTACCGGCNRG	GGCaGcaGT-GACTACtcGCTGG	+	5	1	0	7.16	3.58
Chr4:123030529-123030551	123030545	4	RNA	GGCCGAGGTCGACTACCGGCNRG	tG--GAcGTCGAggACaGGCGGG	+	5	2	0	7.16	3.58
Chr7:67571569-67571591	67571585	4	DNA	GGCCGAGGT--CGACTACCGGCNRG	GGCtGAGGgGCttACTAaCGGCGAG	+	5	2	0	7.16	3.58
Chr10:33397048-33397070	33397064	4	RNA	GGCCGAGGTCGACTACCGGCNRG	G-aCGAGaTCGACaACgaGCGGG	+	5	1	0	7.16	3.58
Chr4:6330673-6330695	6330689	4	RNA	GGCCGAGGTCGACTACCGGCNRG	GGCgGAGGg-GACgACtGGtTAG	+	5	1	0	7.16	3.58
Chr4:226502511-226502533	226502518	5	RNA	GGCCGAGGTCGACTACCGGCNRG	GcCCGAGGgCaACcACC--aTGG	-	5	2	0	7.16	3.58
Chr4:59004096-59004118	59004111	5	RNA	GGCCGAGGTCGACTACCGGCNRG	GGCCGAGcgCGA--AggGGgGAG	+	5	2	0	7.16	3.58
Chr5:17433511-17433533	17433527	4	RNA	GGCCGAGGTCGACTACCGGCNRG	GcCgGgGGTCGACTt--GGaGGG	+	5	2	0	7.16	3.58
Chr6:73639609-73639631	73639614	3	RNA	GGCCGAGGTCGACTACCGGCNRG	GcCgGAGccCGAC--CCGtCAAG	-	5	2	0	7.16	3.58
Chr5:115200351-115200373	115200367	4	DNA	GGCCGAGGTCGACTACCG--GCNRG	GGtCGAGGgCGAaTgaCGATGCGAG	+	5	2	0	7.16	3.58
Chr1:78790585-78790607	78790603	2	RNA	GGCCGAGGTCGACTACCGGCNRG	tGCaGAGcTCGACTA--GGtTGG	+	4	2	0	7.16	3.58
Chr3:402259-402281	402265	4	RNA	GGCCGAGGTCGACTACCGGCNRG	tGgCGAGGTCGAggACC--tTGG	-	5	2	0	7.16	3.58
Chr1:93474325-93474347	93474329	2	RNA	GGCCGAGGTCGACTACCGGCNRG	GGCCGAGGTCGgCT-CaGGgAGG	-	3	1	0	7.16	3.58
Chr2:197224569-197224591	197224574	3	RNA	GGCCGAGGTCGACTACCGGCNRG	GGtCGAc--CGACggCCGGgGGG	-	5	2	0	7.16	3.58
Chr10:16220929-16220951	16220947	2	RNA	GGCCGAGGTCGACTACCGGCNRG	GGgCG-GGcCaACctCCGGCTGG	+	5	1	0	7.16	3.58
Chr7:22342053-22342075	22342060	5	RNA	GGCCGAGGTCGACTACCGGCNRG	cGaCGAGcTCGACT-CtGGtTGG	-	5	1	0	7.16	3.58
Chr6:9863390-9863412	9863406	4	RNA	GGCCGAGGTCGACTACCGGCNRG	G-aCGgGGTCGACaACgaGCGGG	+	5	1	0	7.16	3.58
Chr4:243994844-243994866	243994859	5	RNA	GGCCGAGGTCGACTACCGGCNRG	GGgCG-GGcCaACatCCGGCCGG	+	5	1	0	7.16	3.58
Chr3:210835976-210835998	210835982	4	RNA	GGCCGAGGTCGACTACCGGCNRG	GGCCGAaGcCG--gACCaGaTGG	-	5	2	0	7.16	3.58
Chr5:119332567-119332589	119332573	4	RNA	GGCCGAGGTCGACTACCGGCNRG	GGCCGAaGcgGA--ACCaGaTGG	-	5	2	0	7.16	3.58
Chr1:111870679-111870701	111870695	4	DNA	GGCCGAGGTCGACTACC--GGCNRG	GGCCGActTgGgCTAaCTGGGCTAG	+	5	2	0	7.16	3.58
Chr6:91323980-91324002	91323985	3	RNA	GGCCGAGGTCGACTACCGGCNRG	GGgCGAGagCGAC--CtGGCGGG	-	4	2	0	7.16	3.58
Chr7:77885290-77885312	77885306	4	RNA	GGCCGAGGTCGACTACCGGCNRG	G-aCGgGGTCGACgACgaGCGGG	+	5	1	0	7.16	3.58
Chr6:79443078-79443100	79443083	3	RNA	GGCCGAGGTCGACTACCGGCNRG	G-aCGgGGTCGACgACgaGCGGG	-	5	1	0	7.16	3.58
Chr2:52246652-52246674	52246667	5	RNA	GGCCGAGGTCGACTACCGGCNRG	GGtCacGGTCGAgTgC--GCTGG	+	5	2	0	7.16	3.58
Chr10:58332207-58332229	58332224	3	DNA	G--GCCGAGGTCGACTACCGGCNRG	GATGaCGgGGTCGACgACgaGCGGG	+	5	2	0	7.16	3.58
Chr5:123649676-123649698	123649681	3	RNA	GGCCGAGGTCGACTACCGGCNRG	GGC--ttGcgGACTACCGGCTGG	-	4	2	0	7.16	3.58
Chr4:44343443-44343465	44343448	3	RNA	GGCCGAGGTCGACTACCGGCNRG	GG--GAGGgCGggTgCgGGCGAG	-	5	2	0	7.16	3.58
Chr8:27381695-27381717	27381710	5	RNA	GGCCGAGGTCGACTACCGGCNRG	GGCCGgGccgGACTA--GGtTGG	+	5	2	0	7.16	3.58

Chr7:22832794-22832816	22832799	3	RNA	GGCCGAGGTCGACTACCGGCNRG	GGCttAcG--GACTACCGGtTGG	-	4	2	0	7.16	3.58
Chr10:148732123-148732145	148732138	5	RNA	GGCCGAGGTCGACTACCGGCNRG	GGCCGAGGT--AaaAggGGaTGG	+	5	2	0	7.16	3.58
Chr7:5877505-5877527	5877510	3	DNA	GGCCGAGGTCG-ACTACCGGCNRG	GGCgGAGtcCGCACgAaCGGCGAG	-	5	1	0	7.16	3.58
Chr2:177707135-177707157	177707141	4	RNA	GGCCGAGGTCGACTACCGGCNRG	caCCaAGtTCGcCT-CCGGCCAG	-	5	1	0	7.16	3.58
Chr2:55203621-55203643	55203628	5	RNA	GGCCGAGGTCGACTACCGGCNRG	GGatGAGGagGACT--CGGaCGG	-	5	2	0	7.16	3.58
Chr7:29338454-29338476	29338470	4	RNA	GGCCGAGGTCGACTACCGGCNRG	G-aCGgGGTCGACcACgaGCGGG	+	5	1	0	7.16	3.58
Chr5:203929589-203929611	203929607	2	RNA	GGCCGAGGTCGACTACCGGCNRG	cGCCGtcGcCG--TACCGGCCGG	+	4	2	0	7.16	3.58
Chr4:220932677-220932699	220932695	2	DNA	GGCCGAGGTCG-ACTACCGGCNRG	GGgCtAGGaaGCgCTACCGGCTGG	+	5	1	0	7.16	3.58
Chr6:96275518-96275540	96275534	4	RNA	GGCCGAGGTCGACTACCGGCNRG	GGC-tAGGcCGAggACCtGCCGG	+	5	1	0	7.16	3.58
Chr1:298705082-298705104	298705087	3	RNA	GGCCGAGGTCGACTACCGGCNRG	GaCCGtGGaCGgCaAC--GCAAG	-	5	2	0	7.16	3.58
Chr4:11090905-11090927	11090922	3	RNA	GGCCGAGGTCGACTACCGGCNRG	GGCtGgGaTCGcCTt--GGCAGG	+	5	2	0	7.16	3.58
Chr3:106976798-106976820	106976816	2	DNA	GGCCGAGGTCGAC-TACCGGCNRG	cGCgGgcGTCGACGTACaGGCGGG	+	5	1	0	7.16	3.58
Chr10:128414405-128414427	128414423	2	DNA	GGCCGAGGTCGACT--ACCGGCNRG	GGgtGAGGTtaAtTGGACCGGCGGG	+	5	2	0	7.16	3.58
Chr1:129829565-129829587	129829582	3	RNA	GGCCGAGGTCGACTACCGGCNRG	GGgCGAGcTCGgCTg--GGCGAG	+	4	2	0	7.16	3.58
Chr3:59076867-59076889	59076872	3	RNA	GGCCGAGGTCGACTACCGGCNRG	GG--GtGGcgGACTtCgGGCGGG	-	5	2	0	7.16	3.58
Chr4:151252361-151252383	151252377	4	RNA	GGCCGAGGTCGACTACCGGCNRG	G-aCGgGGTCGACgACaaGCGGG	+	5	1	0	7.16	3.58
Chr8:79028944-79028966	79028949	3	RNA	GGCCGAGGTCGACTACCGGCNRG	G-CCGAaGTCGAagACCttCGGG	-	5	1	0	7.16	3.58
Chr4:22645704-22645726	22645708	2	RNA	GGCCGAGGTCGACTACCGGCNRG	GGaaGAGGTCGAC-AaCGGgCGG	-	4	1	6	1.10	3.55
Chr6:153135198-153135220	153135215	3	RNA	GGCCGAGGTCGACTACCGGCNRG	GttCGAaGgCGA--ACtGGCGGG	+	5	2	7	0.00	3.50
Chr2:203927648-203927670	203927665	3	DNA	G--GCCGAGGTCGACTACCGGCNRG	GATGaCGgGGTCGACaACgaGCGGG	+	5	2	7	0.00	3.50
Chr5:43118194-43118216	43118211	3	RNA	GGCCGAGGTCGACTACCGGCNRG	GtCCG--GTgcACcACCGGgCAG	+	5	2	7	0.00	3.50
Chr5:34213261-34213283	34213278	3	RNA	GGCCGAGGTCGACTACCGGCNRG	G-aCGgGGTCGACgACgaGCGGG	+	5	1	7	0.00	3.50
Chr1:118890949-118890971	118890956	5	RNA	GGCCGAGGTCGACTACCGGCNRG	GGCCGgGccgGACTg--GGCTGG	-	5	2	7	0.00	3.50
Chr10:50507707-50507729	50507714	5	RNA	GGCCGAGGTCGACTACCGGCNRG	GtCCG--GTgcACcACCGGaCAG	-	5	2	7	0.00	3.50
Chr5:184500071-184500093	184500078	5	RNA	GGCCGAGGTCGACTACCGGCNRG	GGCtGgGcTgGgCTA--GGCTGG	-	5	2	7	0.00	3.50
Chr8:101357451-101357473	101357458	5	RNA	GGCCGAGGTCGACTACCGGCNRG	G-aCGgGGTCGACgACgaGCGGG	-	5	1	7	0.00	3.50
Chr3:122575340-122575362	122575357	3	RNA	GGCCGAGGTCGACTACCGGCNRG	GGCCGgGcTCtA--AaCGcCCAG	+	5	2	7	0.00	3.50
Chr5:139807793-139807815	139807810	3	DNA	GGCCGAGGTCGACT-ACCGGCNRG	GGgCGAGGTgGACTGACCGGCCGG	+	2	1	7	0.00	3.50
Chr1:132397497-132397519	132397514	3	RNA	GGCCGAGGTCGACTACCGGCNRG	GGCatAGGaC-ACTACaGGCAGG	+	4	1	7	0.00	3.50
Chr10:75544386-75544408	75544403	3	X	GGCCGAGGTCGACTACCGGCNRG	GGCCGAGGTCGAgcttgGGCGAG	+	5	0	7	0.00	3.50
Chr1:82565696-82565718	82565713	3	RNA	GGCCGAGGTCGACTACCGGCNRG	ctCCGtcGTCaA--ACCGGCAAG	+	5	2	7	0.00	3.50
Chr4:94360303-94360325	94360310	5	DNA	GGCCGAGGT--CGACTACCGGCNRG	GtaCGAGGgAACttCTACCGGCAGG	-	5	2	7	0.00	3.50
Chr6:6243148-6243170	6243155	5	RNA	GGCCGAGGTCGACTACCGGCNRG	GGCCGAGcTgGgCTg--GGtCGG	-	5	2	7	0.00	3.50
Chr3:147242959-147242981	147242976	3	RNA	GGCCGAGGTCGACTACCGGCNRG	GGC--ttGTgGAtTACCGGCTGG	+	4	2	7	0.00	3.50
Chr4:79771225-79771247	79771242	3	RNA	GGCCGAGGTCGACTACCGGCNRG	cGCCc--GTCGtggACCGGCGAG	+	5	2	7	0.00	3.50
Chr2:210440077-210440099	210440084	5	RNA	GGCCGAGGTCGACTACCGGCNRG	aGCaGAG--CGAacgCCGGCGGG	-	5	2	7	0.00	3.50
Chr8:2789127-2789149	2789144	3	RNA	GGCCGAGGTCGACTACCGGCNRG	GGC--tcGcgGACTACCGtCTGG	+	5	2	7	0.00	3.50
Chr9:114322401-114322423	114322418	3	RNA	GGCCGAGGTCGACTACCGGCNRG	GGC--AtGTCcAaTACCttCAAG	+	5	2	7	0.00	3.50
Chr1:96423243-96423265	96423250	5	RNA	GGCCGAGGTCGACTACCGGCNRG	GGgCG-GGcCaACctCCGGCCGG	-	5	1	7	0.00	3.50
Chr6:126437749-126437771	126437766	3	RNA	GGCCGAGGTCGACTACCGGCNRG	GGCCGAGtaCG--TtgCcGCAGG	+	5	2	7	0.00	3.50
Chr4:82973383-82973405	82973390	5	RNA	GGCCGAGGTCGACTACCGGCNRG	tG--GAGGaaGACgACCtGCAAG	-	5	2	7	0.00	3.50
Chr1:83586315-83586337	83586322	5	RNA	GGCCGAGGTCGACTACCGGCNRG	GaCaG-GGTCGACaACgaGCGGG	-	5	1	7	0.00	3.50
Chr9:81332157-81332179	81332174	3	RNA	GGCCGAGGTCGACTACCGGCNRG	G-aCGgGGTCGACaACgaGCGGG	+	5	1	7	0.00	3.50
Chr2:123904469-123904491	123904486	3	RNA	GGCCGAGGTCGACTACCGGCNRG	G-CCGAGGaCGcCgAgCaGCTGG	+	5	1	7	0.00	3.50
Chr7:72631503-72631525	72631510	5	RNA	GGCCGAGGTCGACTACCGGCNRG	GGC-GAGtTCGACgACgaGaTAG	-	5	1	7	0.00	3.50
Chr7:89813178-89813200	89813195	3	RNA	GGCCGAGGTCGACTACCGGCNRG	acgCGAGGgC--CcACCGGCCGG	+	5	2	7	0.00	3.50
Chr9:18535881-18535903	18535888	5	DNA	GGCCGAGGT--CGACTACCGGCNRG	GGCtcAGGTGAaGAgcACCGGCCGG	-	5	2	7	0.00	3.50
Chr5:98526228-98526250	98526245	3	RNA	GGCCGAGGTCGACTACCGGCNRG	GGgCGAG--CGACTAagatCAGG	+	5	2	7	0.00	3.50
Chr4:44065607-44065629	44065614	5	DNA	GGC-CGAGGTCGACTACCGGCNRG	GcCTCGAGGaCGtCTtCCGGCTGG	-	4	1	7	0.00	3.50
Chr2:14961540-14961562	14961557	3	RNA	GGCCGAGGTCGACTACCGGCNRG	GtCgGActTCGACaAC--GCGGG	+	5	2	7	0.00	3.50
Chr7:129103879-129103901	129103886	5	RNA	GGCCGAGGTCGACTACCGGCNRG	GGC--tcGcgGACTACCGGCTGG	-	4	2	7	0.00	3.50
Chr6:154784775-154784797	154784782	5	RNA	GGCCGAGGTCGACTACCGGCNRG	G-CaGtGGagcACTACCGGCCGG	-	5	1	7	0.00	3.50
Chr3:171376425-171376447	171376442	3	RNA	GGCCGAGGTCGACTACCGGCNRG	GGCgGAGGTCG--TtCaGcaGAG	+	5	2	7	0.00	3.50
Chr7:73073809-73073831	73073816	5	RNA	GGCCGAGGTCGACTACCGGCNRG	cGCCG--GTCctCatCCGGCCGG	-	5	2	7	0.00	3.50
Chr4:67786921-67786943	67786938	3	DNA	GGC-CGAGGTCGACTACCGGCNRG	GcCACGgGGaCtACTtCCGGCTGG	+	5	1	7	0.00	3.50
Chr4:170665740-170665762	170665747	5	RNA	GGCCGAGGTCGACTACCGGCNRG	GGCCGAGG-CGAggcCgGGtAGG	-	5	1	7	0.00	3.50
Chr2:78577394-78577416	78577401	5	DNA	GGCCGAGGTCGACTACC--GGCNRG	GcCCaAGcaCGACcACCGTGGCCAG	-	5	2	7	0.00	3.50
Chr8:46329914-46329936	46329931	3	RNA	GGCCGAGGTCGACTACCGGCNRG	GGCCGtctTCGAgTA--GaCCGG	+	5	2	7	0.00	3.50
Chr8:162864120-162864142	162864137	3	DNA	GGC-CGAGGTCGACTACCGGCNRG	GtCTCGAGGaCGtCTtCCGGCTGG	+	4	1	7	0.00	3.50
Chr5:136819775-136819797	136819782	5	RNA	GGCCGAGGTCGACTACCGGCNRG	GGCCG--GcaaACTACCaGtAAG	-	5	2	7	0.00	3.50
Chr5:17629262-17629284	17629269	5	RNA	GGCCGAGGTCGACTACCGGCNRG	GaCCGAGGT-GACgACaaGaAGG	-	5	1	7	0.00	3.50
Chr1:208079284-208079306	208079291	5	RNA	GGCCGAGGTCGACTACCGGCNRG	GttCaAGGTCt--TACCGGaGAG	-	5	2	7	0.00	3.50
Chr10:87020568-87020590	87020585	3	RNA	GGCCGAGGTCGACTACCGGCNRG	G-aCGgGGTCGACaACgaGCGGG	+	5	1	7	0.00	3.50
Chr2:132714464-132714486	132714481	3	RNA	GGCCGAGGTCGACTACCGGCNRG	a--CGgGGTCGACgACgaGCGGG	+	5	2	7	0.00	3.50
Chr2:196222822-196222844	196222839	3	DNA	GGCCGAG-GTCGACTACCGGCNRG	GttCGAGCGTCaACgAtCGGCAGG	+	5	1	7	0.00	3.50
Chr8:115703111-115703133	115703128	3	RNA	GGCCGAGGTCGACTACCGGCNRG	GGgC-AGGcCGAgcACCaGCCGG	+	5	1	7	0.00	3.50
Chr1:182096085-182096107	182096092	5	RNA	GGCCGAGGTCGACTACCGGCNRG	G--CGAGGTCGtCcgtCtGCGAG	-	5	2	7	0.00	3.50
Chr1:58868607-58868629	58868624	3	RNA	GGCCGAGGTCGACTACCGGCNRG	G-CtGAGGcCGACTtCaGaCGAG	+	5	1	7	0.00	3.50
Chr10:73019873-73019895	73019880	5	RNA	GGCCGAGGTCGACTACCGGCNRG	G-CtGAGaTCGcCTAgCtGCGGG	-	5	1	7	0.00	3.50
Chr2:188582221-188582243	188582238	3	RNA	GGCCGAGGTCGACTACCGGCNRG	GGC--tcGcaGACTACCGcCTGG	+	5	2	7	0.00	3.50
Chr10:39089907-39089929	39089914	5	RNA	GGCCGAGGTCGACTACCGGCNRG	GG--GAGGagGtaTACgGGCAAG	-	5	2	7	0.00	3.50
Chr1:101069060-101069082	101069067	5	DNA	GGCCGAGGTCGACTACCGG--CNRG	GGCtGAGGgCGAtTgCCtGCTCCAG	-	5	2	7	0.00	3.50
Chr8:155249330-155249352	155249337	5	RNA	GGCCGAGGTCGACTACCGGCNRG	tGCCGAtGT-GACTgtCGGaGAG	-	5	1	7	0.00	3.50
Chr4:60783612-60783634	60783619	5	DNA	GGC--CGAGGTCGACTACCGGCNRG	GGCCTCGgGGgCtACacCCGGCGGG	-	5	2	7	0.00	3.50
Chr2:183742862-183742884	183742869	5	RNA	GGCCGAGGTCGACTACCGGCNRG	GGC--ttGcaGACTACCGGtTGG	-	5	2	7	0.00	3.50
Chr4:194036935-194036957	194036942	5	RNA	GGCCGAGGTCGACTACCGGCNRG	GGtCGAGGcgcACTAC-GGgTAG	-	5	1	7	0.00	3.50
Chr10:20678206-20678228	20678223	3	RNA	GGCCGAGGTCGACTACCGGCNRG	GGC-GAtGaCGAactCCGGCGAG	+	5	1	7	0.00	3.50
Chr4:176549488-176549510	176549495	5	RNA	GGCCGAGGTCGACTACCGGCNRG	caCC-AGGTCGcCTACCtGgAGG	-	5	1	7	0.00	3.50
Chr8:70163945-70163967	70163952	5	RNA	GGCCGAGGTCGACTACCGGCNRG	GGCttgGGa--ACTACCGGaAGG	-	5	2	7	0.00	3.50
Chr5:218107646-218107668	218107653	5	DNA	GGCCGAGGTCGACTA-CCGGCNRG	aGCCaAGGTCaACTAGCCGcaTGG	-	5	1	7	0.00	3.50
Chr5:128710966-128710988	128710983	3	RNA	GGCCGAGGTCGACTACCGGCNRG	GaCaG-GGTCGACgACaaGCGGG	+	5	1	7	0.00	3.50
Chr3:78246029-78246051	78246036	5	RNA	GGCCGAGGTCGACTACCGGCNRG	GGCCGAGcTgGAg--CtGGgAAG	-	5	2	7	0.00	3.50
Chr1:69482345-69482367	69482362	3	RNA	GGCCGAGGTCGACTACCGGCNRG	GGC-GAGcaCctgTACCGGCCGG	+	5	1	7	0.00	3.50
Chr7:104890395-104890417	104890402	5	RNA	GGCCGAGGTCGACTACCGGCNRG	GGCCGgGcTgGgCTg--GGCTGG	-	5	2	7	0.00	3.50
Chr1:290345962-290345984	290345979	3	RNA	GGCCGAGGTCGACTACCGGCNRG	GGaCGAGG-CGACggaCtGCTGG	+	5	1	7	0.00	3.50
Chr3:59473846-59473868	59473853	5	DNA	GGCCGAGGTCGACTA--CCGGCNRG	GGtCaAGGTCGACcAAGCCacCAAG	-	5	2	7	0.00	3.50
Chr8:111280383-111280405	111280390	5	RNA	GGCCGAGGTCGACTACCGGCNRG	GGgCG-GGcCaACctCCGGCCGG	-	5	1	7	0.00	3.50
Chr5:32392655-32392677	32392672	3	RNA	GGCCGAGGTCGACTACCGGCNRG	GttCGAGaT--ACTtCgGGCAAG	+	5	2	7	0.00	3.50
Chr6:119742892-119742914	119742909	3	RNA	GGCCGAGGTCGACTACCGGCNRG	GtCttgGGT-GACcACCGGCGGG	+	5	1	7	0.00	3.50
Chr1:252608099-252608121	252608106	5	RNA	GGCCGAGGTCGACTACCGGCNRG	caCCaAGtTCGcCT-CCGGCCAG	-	5	1	7	0.00	3.50
Chr6:142107425-142107447	142107442	3	RNA	GGCCGAGGTCGACTACCGGCNRG	GGC--AcactGACcACCGGCGGG	+	5	2	7	0.00	3.50
Chr2:83285025-83285047	83285042	3	RNA	GGCCGAGGTCGACTACCGGCNRG	GGCCcAG--CGAagACCcGgGGG	+	5	2	7	0.00	3.50
Chr7:69146935-69146957	69146942	5	RNA	GGCCGAGGTCGACTACCGGCNRG	GGCCGgGtcgGAC--CgGGCCGG	-	5	2	7	0.00	3.50
Chr10:128566492-128566514	128566499	5	RNA	GGCCGAGGTCGACTACCGGCNRG	GGgCGAaGgCaACT-CgGGCGGG	-	5	1	7	0.00	3.50
Chr4:82725659-82725681	82725666	5	RNA	GGCCGAGGTCGACTACCGGCNRG	GGCgtgGGgCaACT-CCGGCGAG	-	5	1	7	0.00	3.50
Chr8:155273821-155273843	155273828	5	RNA	GGCCGAGGTCGACTACCGGCNRG	aaaCGAG--aGACaACCGGCTGG	-	5	2	7	0.00	3.50
Chr3:152917112-152917134	152917129	3	RNA	GGCCGAGGTCGACTACCGGCNRG	GGgCGgGGTtGgCT--CGGgGAG	+	5	2	7	0.00	3.50
Chr7:48025358-48025380	48025375	3	DNA	GGCCGAGGT--CGACTACCGGCNRG	aGaCGAGGTTCCGACcAagGGCAGG	+	5	2	7	0.00	3.50
Chr8:123372560-123372582	123372577	3	RNA	GGCCGAGGTCGACTACCGGCNRG	GGCgGAGG--GACagCtGtCGGG	+	5	2	7	0.00	3.50
Chr3:224266456-224266478	224266473	3	RNA	GGCCGAGGTCGACTACCGGCNRG	GGC--tcGcgGACTACCGcCTGG	+	5	2	7	0.00	3.50
Chr4:145494802-145494824	145494809	5	RNA	GGCCGAGGTCGACTACCGGCNRG	tGCCGAaGgaGACTACtG-CTGG	-	5	1	7	0.00	3.50
Chr8:124423370-124423392	124423377	5	RNA	GGCCGAGGTCGACTACCGGCNRG	aGgaGAGGTgGACTACa--CCAG	-	5	2	7	0.00	3.50
Chr7:56578178-56578200	56578195	3	RNA	GGCCGAGGTCGACTACCGGCNRG	GGCCGAtcTCG--cAtCaGCAAG	+	5	2	7	0.00	3.50
Chr7:151409140-151409162	151409147	5	RNA	GGCCGAGGTCGACTACCGGCNRG	G-aCGgGGTCGACaACgaGCGGG	-	5	1	7	0.00	3.50
Chr5:114748179-114748201	114748186	5	RNA	GGCCGAGGTCGACTACCGGCNRG	tagCGAGcTCG--TACtGGCCAG	-	5	2	7	0.00	3.50
Chr5:68831643-68831665	68831650	5	RNA	GGCCGAGGTCGACTACCGGCNRG	GGa-GcGGgaGACaACCGGCTGG	-	5	1	7	0.00	3.50
Chr5:117397363-117397385	117397380	3	RNA	GGCCGAGGTCGACTACCGGCNRG	GGC--tcGcgGACTACCGGCTGG	+	4	2	7	0.00	3.50
Chr8:59232310-59232332	59232317	5	RNA	GGCCGAGGTCGACTACCGGCNRG	GGgCacGGTCGgCTACC--CTGG	-	4	2	7	0.00	3.50
Chr4:73384049-73384071	73384066	3	RNA	GGCCGAGGTCGACTACCGGCNRG	GGC--AGGcCGAgcACCGGCCGG	+	3	2	7	0.00	3.50
Chr8:115764999-115765021	115765006	5	DNA	GGCCGAGGTC-GACTACCGGCNRG	GGCCGAGGcCTGAggtCgGGCGAG	-	5	1	7	0.00	3.50
Chr7:74380977-74380999	74380994	3	RNA	GGCCGAGGTCGACTACCGGCNRG	cGCCGAGGagGACcAg-GGCCGG	+	5	1	7	0.00	3.50
Chr10:67921131-67921153	67921148	3	RNA	GGCCGAGGTCGACTACCGGCNRG	aGCaGgGcTCGACTAC-GGaCGG	+	5	1	7	0.00	3.50
Chr8:87053422-87053444	87053439	3	DNA	GGCCGAGGTCGACTA-CCGGCNRG	cGtCGAGGTCcACcATCaGGCGGG	+	5	1	7	0.00	3.50
Chr5:82849175-82849197	82849182	5	RNA	GGCCGAGGTCGACTACCGGCNRG	G-aCGgGGTCGACgACgaGCGGG	-	5	1	7	0.00	3.50
Chr2:170258467-170258489	170258484	3	RNA	GGCCGAGGTCGACTACCGGCNRG	GGtCcAGGgC--CTtCCtGCGAG	+	5	2	7	0.00	3.50
Chr1:214812603-214812625	214812610	5	DNA	GGCCGAGGTCGACTACCGG--CNRG	GGtCGAGGcCGAgTcCgGGGTCGGG	-	5	2	7	0.00	3.50
Chr1:275415405-275415427	275415422	3	RNA	GGCCGAGGTCGACTACCGGCNRG	ttCCGAtGTCGAtTA--tGCCGG	+	5	2	7	0.00	3.50
Chr2:174401136-174401158	174401153	3	RNA	GGCCGAGGTCGACTACCGGCNRG	G-aCGgGGTCGACaACgaGCGGG	+	5	1	7	0.00	3.50
Chr2:121584147-121584169	121584154	5	RNA	GGCCGAGGTCGACTACCGGCNRG	G-CtGAGaTCGcCTAgCtGCGGG	-	5	1	7	0.00	3.50
Chr8:53101329-53101351	53101336	5	RNA	GGCCGAGGTCGACTACCGGCNRG	GGC--AGGcCGAactCCGGCCGG	-	4	2	7	0.00	3.50
Chr10:43246327-43246349	43246334	5	DNA	GGCCGAGGTCGACTACC--GGCNRG	GcCCGAGagCaACcACCGTGGCCAG	-	5	2	7	0.00	3.50
Chr5:128592874-128592896	128592891	3	RNA	GGCCGAGGTCGACTACCGGCNRG	cGgCGAGcctG--TACCGGCTGG	+	5	2	7	0.00	3.50
Chr4:210515291-210515313	210515308	3	RNA	GGCCGAGGTCGACTACCGGCNRG	GaCCGcG--CGtCTAgCGaCCAG	+	5	2	7	0.00	3.50
Chr1:88901509-88901531	88901526	3	RNA	GGCCGAGGTCGACTACCGGCNRG	GGCCGAGcTC--CggCgaGCTGG	+	5	2	7	0.00	3.50
Chr4:29844905-29844927	29844912	5	DNA	GGCCGAGGTCGACTA--CCGGCNRG	ctCCtAGGcCGACcAATCCGGCAGG	-	5	2	7	0.00	3.50
Chr9:37132733-37132755	37132740	5	RNA	GGCCGAGGTCGACTACCGGCNRG	cGCCGgGGcCGAC-ACCGaCGGG	-	4	1	7	0.00	3.50

Chr10:101058734-101058756	101058741	5	RNA	GGCCGAGGTCGACTACCGGCNRG	GtCCGAcGTaGcCT--CGGaCGG	-	5	2	7	0.00	3.50
Chr3:76087062-76087084	76087079	3	RNA	GGCCGAGGTCGACTACCGGCNRG	GGgCGcGGTCGgCg--CGGgGAG	+	5	2	7	0.00	3.50
Chr2:109839679-109839701	109839696	3	RNA	GGCCGAGGTCGACTACCGGCNRG	GGCCacGGgCGgCT-CtGGCGGG	+	5	1	7	0.00	3.50
Chr9:17097128-17097150	17097133	3	RNA	GGCCGAGGTCGACTACCGGCNRG	GGC--tcGcgGACTACCGGCTGG	-	4	2	2	4.96	3.48
Chr2:118375013-118375035	118375018	3	RNA	GGCCGAGGTCGACTACCGGCNRG	G--aGcGGgaGACgACCGGCTGG	-	5	2	3	3.86	3.43
Chr7:122722114-122722136	122722132	2	RNA	GGCCGAGGTCGACTACCGGCNRG	GcCCGgGGa--AaTACCcGCTAG	+	5	2	4	2.75	3.38
Chr2:220603800-220603822	220603817	3	RNA	GGCCGAGGTCGACTACCGGCNRG	tG--GAGGTCGACTgCgGtaCGG	+	5	2	4	2.75	3.38
Chr5:50331645-50331667	50331663	2	RNA	GGCCGAGGTCGACTACCGGCNRG	GcCCatGGTCGAgTtC--GCCAG	+	5	2	5	1.65	3.33
Chr7:132883954-132883976	132883959	3	RNA	GGCCGAGGTCGACTACCGGCNRG	G-aCGgGGTCGACaACgaGCGGG	-	5	1	5	1.65	3.33
Chr5:132656602-132656624	132656617	5	RNA	GGCCGAGGTCGACTACCGGCNRG	GGCtGgGcTgGACTA--GGtTGG	+	5	2	0	6.61	3.31
Chr2:191357245-191357267	191357250	3	RNA	GGCCGAGGTCGACTACCGGCNRG	GGC--AGGcCGggcACtGGCCGG	-	5	2	0	6.61	3.31
Chr7:112767413-112767435	112767420	5	RNA	GGCCGAGGTCGACTACCGGCNRG	GtCCGgtGT--ACcACCGGaCAG	-	5	2	0	6.61	3.31
Chr4:209877700-209877722	209877705	3	RNA	GGCCGAGGTCGACTACCGGCNRG	GGCCGAGGagGAaggC--GCGGG	-	5	2	0	6.61	3.31
Chr1:149914618-149914640	149914633	5	RNA	GGCCGAGGTCGACTACCGGCNRG	aG--GAcGaCGACTAgCaGCAGG	+	5	2	0	6.61	3.31
Chr5:141994705-141994727	141994721	4	RNA	GGCCGAGGTCGACTACCGGCNRG	cGgCGAcGTCGA--AgCtGCTGG	+	5	2	0	6.61	3.31
Chr1:62812157-62812179	62812175	2	RNA	GGCCGAGGTCGACTACCGGCNRG	GcCgGAGccCGAC--CCGtCAAG	+	5	2	0	6.61	3.31
Chr7:150943166-150943188	150943171	3	RNA	GGCCGAGGTCGACTACCGGCNRG	G-aCGgGGTCGACaACgaGCGGG	-	5	1	0	6.61	3.31
Chr8:436501-436523	436508	5	RNA	GGCCGAGGTCGACTACCGGCNRG	cG--GtGGTCGACgAgCtGCTGG	-	5	2	0	6.61	3.31
Chr9:62104788-62104810	62104792	2	DNA	GGCCGAGGTCGACTACCGG--CNRG	GGCaGAtaTCGcgTACCGGTTCAGG	-	5	2	0	6.61	3.31
Chr8:126611744-126611766	126611762	2	RNA	GGCCGAGGTCGACTACCGGCNRG	GGCCctGGTC--CcACCtGCAGG	+	4	2	0	6.61	3.31
Chr7:154322906-154322928	154322913	5	RNA	GGCCGAGGTCGACTACCGGCNRG	GGCCGAGGgtGcCT-CgGGCTGG	-	4	1	0	6.61	3.31
Chr8:104798108-104798130	104798125	3	RNA	GGCCGAGGTCGACTACCGGCNRG	caCCaAGtTCGcCT-CCGGCCAG	+	5	1	0	6.61	3.31
Chr3:210542609-210542631	210542614	3	DNA	GGCCGAGGTCGACTA--CCGGCNRG	ctCCtAGGcCGACcAATCCGGCAGG	-	5	2	0	6.61	3.31
Chr2:13460991-13461013	13460996	3	RNA	GGCCGAGGTCGACTACCGGCNRG	GGgCG-GGcCaACctCCGGCCGG	-	5	1	0	6.61	3.31
Chr2:118665339-118665361	118665343	2	RNA	GGCCGAGGTCGACTACCGGCNRG	GGtCcAGGagGA--ACaGGCAAG	-	5	2	0	6.61	3.31
Chr1:706593-706615	706597	2	DNA	GGCCGAGGTCGACTA--CCGGCNRG	GaCCGgaGTCGACgAAGCCGGtGGG	-	5	2	0	6.61	3.31
Chr2:170420095-170420117	170420100	3	DNA	GGCCGAGGTCGACTA--CCGGCNRG	ctCCtAGGcCGACcAATCCGGCAGG	-	5	2	0	6.61	3.31
Chr8:154675293-154675315	154675310	3	DNA	G-GCCGAGGTCGACTACCGGCNRG	GCGCCtcGGgCGAgTACtGGCAGG	+	5	1	0	6.61	3.31
Chr4:140230920-140230942	140230938	2	RNA	GGCCGAGGTCGACTACCGGCNRG	a--CGgGGTCGACaACgaGCGGG	+	5	2	0	6.61	3.31
Chr4:93762624-93762646	93762639	5	RNA	GGCCGAGGTCGACTACCGGCNRG	cGgCGAGcctGA--ACCGGCCGG	+	5	2	0	6.61	3.31
Chr7:110131344-110131366	110131350	4	RNA	GGCCGAGGTCGACTACCGGCNRG	cG--GAGGggGAaTACCtGCTGG	-	5	2	0	6.61	3.31
Chr7:117228090-117228112	117228094	2	RNA	GGCCGAGGTCGACTACCGGCNRG	cGaCGAtGTCGACcACC--tGAG	-	5	2	0	6.61	3.31
Chr5:72879057-72879079	72879072	5	RNA	GGCCGAGGTCGACTACCGGCNRG	GGCCGAaaTCccCTcCC--CTGG	+	5	2	0	6.61	3.31
Chr7:113449336-113449358	113449351	5	RNA	GGCCGAGGTCGACTACCGGCNRG	GGC--tcGTgGACTACCGGCTGG	+	3	2	0	6.61	3.31
Chr2:132941984-132942006	132941990	4	RNA	GGCCGAGGTCGACTACCGGCNRG	tGCCGAGGT-GACaAagGaCAGG	-	5	1	0	6.61	3.31
Chr1:286460736-286460758	286460742	4	RNA	GGCCGAGGTCGACTACCGGCNRG	GGaCcAGGTCG--TcCCaGtTGG	-	5	2	0	6.61	3.31
Chr1:36204131-36204153	36204136	3	RNA	GGCCGAGGTCGACTACCGGCNRG	GGgCG-GGcCaACccCCGGCCGG	-	5	1	0	6.61	3.31
Chr3:134841581-134841603	134841596	5	RNA	GGCCGAGGTCGACTACCGGCNRG	GGgCG-GGcCaACccCCGGCCGG	+	5	1	0	6.61	3.31
Chr7:107967449-107967471	107967464	5	RNA	GGCCGAGGTCGACTACCGGCNRG	GGCCGAGcgCGA--AggGGgGAG	+	5	2	0	6.61	3.31
Chr1:246890071-246890093	246890076	3	RNA	GGCCGAGGTCGACTACCGGCNRG	G-aCGgGGTCGACaACaaGCGGG	-	5	1	0	6.61	3.31
Chr1:134587246-134587268	134587251	3	RNA	GGCCGAGGTCGACTACCGGCNRG	acCCGAGcTCGAgTtC--GCCGG	-	5	2	0	6.61	3.31
Chr8:76911959-76911981	76911965	4	DNA	GGCCGAGGTCGACTAC--CGGCNRG	tGaCGAGGgCGACgACGGCGaCAAG	-	5	2	0	6.61	3.31
Chr5:223334173-223334195	223334188	5	RNA	GGCCGAGGTCGACTACCGGCNRG	GGa-GAGGaaGACgAgCGGCGGG	+	5	1	0	6.61	3.31
Chr5:31582421-31582443	31582436	5	DNA	GGC--CGAGGTCGACTACCGGCNRG	GGCCTCGgGGgCtACacCCGGCGGG	+	5	2	0	6.61	3.31
Chr1:62811430-62811452	62811435	3	RNA	GGCCGAGGTCGACTACCGGCNRG	G-aCGgGGTCGACgACgaGCGGG	-	5	1	0	6.61	3.31
Chr8:26953026-26953048	26953042	4	RNA	GGCCGAGGTCGACTACCGGCNRG	G-aCGgGGTCGACaACgaGCGGG	+	5	1	0	6.61	3.31
Chr2:158280105-158280127	158280109	2	RNA	GGCCGAGGTCGACTACCGGCNRG	GGCCcAc--CGACTgCCGcgGGG	-	5	2	0	6.61	3.31
Chr2:167850650-167850672	167850657	5	RNA	GGCCGAGGTCGACTACCGGCNRG	GttCGAGcTCG--TACtGGaCAG	-	5	2	0	6.61	3.31
Chr4:222910238-222910260	222910256	2	RNA	GGCCGAGGTCGACTACCGGCNRG	GtCCG--GaaGAgTACCGGaAAG	+	5	2	0	6.61	3.31
Chr10:42821618-42821640	42821624	4	RNA	GGCCGAGGTCGACTACCGGCNRG	G--CGtGGatGACTACCGcgTGG	-	5	2	0	6.61	3.31
Chr2:26710193-26710215	26710197	2	RNA	GGCCGAGGTCGACTACCGGCNRG	GGgCgGGGTCGACgAC-GcgAGG	-	5	1	0	6.61	3.31
Chr4:39844606-39844628	39844621	5	RNA	GGCCGAGGTCGACTACCGGCNRG	GGC--tcGagGACTACCGGCTGG	+	4	2	0	6.61	3.31
Chr1:242800982-242801004	242800999	3	RNA	GGCCGAGGTCGACTACCGGCNRG	G--tGAGGctGAgcACCGGCCAG	+	5	2	0	6.61	3.31
Chr1:119652003-119652025	119652009	4	DNA	GGC-CGAGGTCGACTACCGGCNRG	GcCTCGAGGaCGtCTtCCaGCTGG	-	5	1	0	6.61	3.31
Chr4:195261284-195261306	195261289	3	RNA	GGCCGAGGTCGACTACCGGCNRG	GGCtcAc--aGACTACCGGCTGG	-	4	2	0	6.61	3.31
Chr10:36951947-36951969	36951963	4	RNA	GGCCGAGGTCGACTACCGGCNRG	GcCgGAGccCGAC--CCGtCAAG	+	5	2	0	6.61	3.31
Chr3:26127355-26127377	26127371	4	RNA	GGCCGAGGTCGACTACCGGCNRG	aaCaGgGGTCGACTA--GGgGAG	+	5	2	0	6.61	3.31
Chr1:29483704-29483726	29483708	2	DNA	GGC-CGAGGTCGACTACCGGCNRG	cGCTCGAGGTCaACctCgGGCGAG	-	5	1	0	6.61	3.31
Chr9:58043514-58043536	58043521	5	RNA	GGCCGAGGTCGACTACCGGCNRG	GGCCGAGcTC-ACccCCtGCCAG	-	4	1	0	6.61	3.31
Chr3:232572372-232572394	232572390	2	RNA	GGCCGAGGTCGACTACCGGCNRG	tGCgGAGGTgGACa--CcGCGAG	+	5	2	0	6.61	3.31
Chr2:4237640-4237662	4237644	2	DNA	GGCC-GAGGTCGACTACCGGCNRG	GtCCAGAGGaCGACacaCGGCCGG	-	5	1	0	6.61	3.31
Chr3:226146250-226146272	226146254	2	RNA	GGCCGAGGTCGACTACCGGCNRG	GcCCGAGGcCGA--ACtcGgTGG	-	5	2	0	6.61	3.31
Chr2:228863194-228863216	228863212	2	RNA	GGCCGAGGTCGACTACCGGCNRG	GcCgGAGccCGAC--CCGtCAGG	+	5	2	0	6.61	3.31
Chr8:160556769-160556791	160556774	3	RNA	GGCCGAGGTCGACTACCGGCNRG	G-aCGgGGTCGACaACaaGCGGG	-	5	1	0	6.61	3.31
Chr9:78187110-78187132	78187116	4	RNA	GGCCGAGGTCGACTACCGGCNRG	cGCCGtGGTCGcCg--CGGCAGG	-	4	2	0	6.61	3.31
Chr5:179994095-179994117	179994099	2	RNA	GGCCGAGGTCGACTACCGGCNRG	GtCttgGGT-GACcACCGGCGGG	-	5	1	0	6.61	3.31
Chr1:262657980-262658002	262657997	3	RNA	GGCCGAGGTCGACTACCGGCNRG	GGCCGAGGgCtA-gACCcGtGGG	+	5	1	0	6.61	3.31
Chr6:48834209-48834231	48834213	2	DNA	GGCCGAGGTCGACTACCGG-CNRG	GGCCGAGtTgGcCTggCGGCCTAG	-	5	1	0	6.61	3.31
Chr1:173662124-173662146	173662141	3	RNA	GGCCGAGGTCGACTACCGGCNRG	GGC--AGGcCaACctCCGGCTGG	+	4	2	0	6.61	3.31
Chr2:129399458-129399480	129399474	4	DNA	GGCCGAGGTCGAC--TACCGGCNRG	GcgCcAtcTCGACTCTACCGGCGAG	+	5	2	0	6.61	3.31
Chr9:76346098-76346120	76346113	5	RNA	GGCCGAGGTCGACTACCGGCNRG	GaCgaAGGTgGACTA--GtCAGG	+	5	2	0	6.61	3.31
Chr7:56579233-56579255	56579239	4	RNA	GGCCGAGGTCGACTACCGGCNRG	GGCCGAtcTCG--cAtCaGCAAG	-	5	2	0	6.61	3.31
Chr8:59924109-59924131	59924124	5	DNA	GGCCGAG--GTCGACTACCGGCNRG	GctCGAGCGGgaGACgACCGGCTGG	+	5	2	0	6.61	3.31
Chr8:126646991-126647013	126646995	2	RNA	GGCCGAGGTCGACTACCGGCNRG	GGCCGAGGTCG--ggCgaGgCGG	-	5	2	0	6.61	3.31
Chr3:152155382-152155404	152155388	4	DNA	GGC--CGAGGTCGACTACCGGCNRG	GGCCTCagGGaCGtCTtCCGGCAAG	-	5	2	0	6.61	3.31
Chr1:32038881-32038903	32038897	4	RNA	GGCCGAGGTCGACTACCGGCNRG	G-aCGgGGTCGACaACgaGCGGG	+	5	1	0	6.61	3.31
Chr5:92606775-92606797	92606790	5	RNA	GGCCGAGGTCGACTACCGGCNRG	GGCCatGGcCGA--ACCcGCCAG	+	4	2	0	6.61	3.31
Chr6:84303473-84303495	84303477	2	RNA	GGCCGAGGTCGACTACCGGCNRG	GcCCGAGccCGAgT--CGGtAGG	-	5	2	0	6.61	3.31
Chr2:22314149-22314171	22314154	3	RNA	GGCCGAGGTCGACTACCGGCNRG	cGtCGAGGTCcA-gACtGGCAAG	-	5	1	0	6.61	3.31
Chr1:20374787-20374809	20374802	5	RNA	GGCCGAGGTCGACTACCGGCNRG	GGC--AGGcCGAgcACtcGCCGG	+	5	2	0	6.61	3.31
Chr2:190325651-190325673	190325657	4	RNA	GGCCGAGGTCGACTACCGGCNRG	tGtaGAGtT-GAaTACCGGCTGG	-	5	1	0	6.61	3.31
Chr4:62678695-62678717	62678701	4	DNA	GGC-CGAGGTCGACTACCGGCNRG	GcCTCGgGGaCGtCTtCCGGCTGG	-	5	1	0	6.61	3.31
Chr1:116873093-116873115	116873111	2	RNA	GGCCGAGGTCGACTACCGGCNRG	GG--GAtGgCGAgcACCGGtGAG	+	5	2	0	6.61	3.31
Chr3:26264873-26264895	26264878	3	RNA	GGCCGAGGTCGACTACCGGCNRG	G-aCGgGGTCGACaACgaGCGGG	-	5	1	0	6.61	3.31
Chr2:149630338-149630360	149630356	2	RNA	GGCCGAGGTCGACTACCGGCNRG	GGC--AGGcCaACctCCGGCCGG	+	4	2	0	6.61	3.31
Chr1:117372166-117372188	117372182	4	RNA	GGCCGAGGTCGACTACCGGCNRG	GGC-GAGGTCGgCcACCaGgGAG	+	4	1	0	6.61	3.31
Chr2:217698302-217698324	217698319	3	RNA	GGCCGAGGTCGACTACCGGCNRG	GGgCGccGgCGA--ACCaGCAGG	+	5	2	0	6.61	3.31
Chr3:160982243-160982265	160982250	5	RNA	GGCCGAGGTCGACTACCGGCNRG	GGC--tcGcaGACTACCGcCTGG	-	5	2	0	6.61	3.31
Chr2:162923118-162923140	162923133	5	RNA	GGCCGAGGTCGACTACCGGCNRG	GGCCGAGccCGA-gAgtGGCCAG	+	5	1	0	6.61	3.31
Chr1:101470370-101470392	101470375	3	DNA	GGCCGAG-GTCGACTACCGGCNRG	GttCGAGCGTCaACgAtCGGCAGG	-	5	1	0	6.61	3.31
Chr9:15776841-15776863	15776859	2	DNA	GGCCGAGGTCGACTACC--GGCNRG	GGCgGAGGTCGtCTgCaGAGGCCAG	+	4	2	0	6.61	3.31
Chr4:200141171-200141193	200141176	3	DNA	GGCCGAGGTCGACTA--CCGGCNRG	cGtaaAGGTCGACcAATCCGGCAGG	-	5	2	0	6.61	3.31
Chr1:168558804-168558826	168558809	3	RNA	GGCCGAGGTCGACTACCGGCNRG	G-aCGAGGTCGACaACgaGCGGG	-	4	1	0	6.61	3.31
Chr2:206435290-206435312	206435296	4	RNA	GGCCGAGGTCGACTACCGGCNRG	G-CCtcGGgCGAaTACtGGCAGG	-	5	1	0	6.61	3.31
Chr1:68850688-68850710	68850695	5	RNA	GGCCGAGGTCGACTACCGGCNRG	GGC--AGGcCaACctCCGGCCGG	-	4	2	0	6.61	3.31
Chr5:28838900-28838922	28838915	5	RNA	GGCCGAGGTCGACTACCGGCNRG	GGtCGcGGaCGAgT--CGGCGGG	+	4	2	0	6.61	3.31
Chr3:73329873-73329895	73329878	3	DNA	GGCCGAGGTCGACTA--CCGGCNRG	GGgCGgGaTgGACaACTCCGGCGAG	-	5	2	6	0.55	3.28
Chr1:129429467-129429489	129429483	4	DNA	GGCCGAGGTCGACTACC--GGCNRG	GcCCGAGagCaACcACCATGGCCAG	+	5	2	6	0.55	3.28
Chr4:83512534-83512556	83512552	2	DNA	GGCCGAGGTCGA-CTACCGGCNRG	GaCtcAGGTCGATCTggCGGCGGG	+	5	1	6	0.55	3.28
Chr4:183244851-183244873	183244856	3	RNA	GGCCGAGGTCGACTACCGGCNRG	G-aCGgGGTCGACaACgaGCGGG	-	5	1	6	0.55	3.28
Chr6:113403735-113403757	113403740	3	RNA	GGCCGAGGTCGACTACCGGCNRG	GGC--ttGcgGACTACCGGCTGG	-	4	2	6	0.55	3.28
Chr5:65009067-65009089	65009083	4	RNA	GGCCGAGGTCGACTACCGGCNRG	G-aCGgGaTCGACaACCaGCGGG	+	5	1	2	4.41	3.21
Chr7:43329720-43329742	43329725	3	RNA	GGCCGAGGTCGACTACCGGCNRG	t--CttcGTCGACTACCGGCGGG	-	4	2	2	4.41	3.21
Chr4:44208712-44208734	44208727	5	RNA	GGCCGAGGTCGACTACCGGCNRG	GtC--tccTtGACTACCGGCCGG	+	5	2	3	3.30	3.15
Chr8:130821435-130821457	130821440	3	RNA	GGCCGAGGTCGACTACCGGCNRG	GGC--AGGcCGAgcACCGGCCGG	-	3	2	4	2.20	3.10
Chr5:81287185-81287207	81287191	4	RNA	GGCCGAGGTCGACTACCGGCNRG	GGC--ttGcgGACTACCGGCTGG	-	4	2	4	2.20	3.10
Chr4:226970908-226970930	226970914	4	RNA	GGCCGAGGTCGACTACCGGCNRG	GGgCGtGGTgGACcACC--aCGG	-	5	2	4	2.20	3.10
Chr10:31757182-31757204	31757189	5	RNA	GGCCGAGGTCGACTACCGGCNRG	aGCaGgGcTCGACTAC-GGaCGG	-	5	1	5	1.10	3.05
Chr2:216234751-216234773	216234756	3	RNA	GGCCGAGGTCGACTACCGGCNRG	G-aCGgGGTCGACaACgaGCGGG	-	5	1	5	1.10	3.05
Chr1:245881204-245881226	245881209	3	RNA	GGCCGAGGTCGACTACCGGCNRG	G-aCGgGGTCGACaACgaGCGGG	-	5	1	0	6.06	3.03
Chr2:66950351-66950373	66950367	4	RNA	GGCCGAGGTCGACTACCGGCNRG	GaCaG-GGTCGACgACaaGCGGG	+	5	1	0	6.06	3.03
Chr4:154841632-154841654	154841648	4	RNA	GGCCGAGGTCGACTACCGGCNRG	G-aCGgGGTCGACaACgaGCGGG	+	5	1	0	6.06	3.03
Chr4:39033658-39033680	39033664	4	RNA	GGCCGAGGTCGACTACCGGCNRG	aGt--AGGaCGACTACgaGCAGG	-	5	2	0	6.06	3.03
Chr5:33134336-33134358	33134351	5	RNA	GGCCGAGGTCGACTACCGGCNRG	GGCgGAGG--GACaACtGtCGGG	+	4	2	0	6.06	3.03
Chr7:6227987-6228009	6228005	2	RNA	GGCCGAGGTCGACTACCGGCNRG	GGCtGgGcTgGACTA--GGtTGG	+	5	2	0	6.06	3.03
Chr4:65506444-65506466	65506462	2	RNA	GGCCGAGGTCGACTACCGGCNRG	GGCCG--GTCcACTttCGaaAAG	+	5	2	0	6.06	3.03
Chr2:40662144-40662166	40662160	4	RNA	GGCCGAGGTCGACTACCGGCNRG	G-aCGgGGTCGACaACgaGCGGG	+	5	1	0	6.06	3.03
Chr9:93885367-93885389	93885383	4	RNA	GGCCGAGGTCGACTACCGGCNRG	tGCCGAtGTgG--cACCGGaGAG	+	5	2	0	6.06	3.03
Chr1:269001932-269001954	269001938	4	RNA	GGCCGAGGTCGACTACCGGCNRG	cGCgGcGG-CGACTACtGGCCAG	-	4	1	0	6.06	3.03
Chr2:7791386-7791408	7791403	3	RNA	GGCCGAGGTCGACTACCGGCNRG	GGaCcAGGTCG--TcCCaGtTGG	+	5	2	0	6.06	3.03
Chr9:89937215-89937237	89937230	5	RNA	GGCCGAGGTCGACTACCGGCNRG	GGC--tcGcgGACTACCGGCTGG	+	4	2	0	6.06	3.03
Chr1:142432589-142432611	142432593	2	RNA	GGCCGAGGTCGACTACCGGCNRG	GGCCaAGcTCaAaTg--GGCCAG	-	5	2	0	6.06	3.03
Chr7:166764771-166764793	166764775	2	RNA	GGCCGAGGTCGACTACCGGCNRG	GcCCGAGGcCGA--ACtcGgTGG	-	5	2	0	6.06	3.03
Chr6:55724835-55724857	55724841	4	X	GGCCGAGGTCGACTACCGGCNRG	GGCaGAGGaCGAacAaCGGCGAG	-	5	0	0	6.06	3.03
Chr1:174719672-174719694	174719677	3	RNA	GGCCGAGGTCGACTACCGGCNRG	GGCgGAGGTC--CaAaCctCTGG	-	5	2	0	6.06	3.03
Chr2:80020140-80020162	80020158	2	RNA	GGCCGAGGTCGACTACCGGCNRG	cG--GAGGTgaAtcACCGGCCAG	+	5	2	0	6.06	3.03

Chr5:47727559-47727581	47727566	5	DNA	GGCCGAGGTCGACT--ACCGGCNRG	GGCCtAGGTacAtTGAAaCGGCCAG	-	5	2	0	6.06	3.03
Chr8:94051057-94051079	94051073	4	RNA	GGCCGAGGTCGACTACCGGCNRG	GGCCGAGGTCG--ggCgaGgCGG	+	5	2	0	6.06	3.03
Chr1:290316383-290316405	290316387	2	RNA	GGCCGAGGTCGACTACCGGCNRG	GGCCGAGGTCG--ggCgaGgCGG	-	5	2	0	6.06	3.03
Chr4:180668802-180668824	180668818	4	RNA	GGCCGAGGTCGACTACCGGCNRG	G-aCGgGGTCGACgACaaGCGGG	+	5	1	0	6.06	3.03
Chr1:39102091-39102113	39102109	2	RNA	GGCCGAGGTCGACTACCGGCNRG	c--CGAGGTCaACcACCacCAAG	+	5	2	0	6.06	3.03
Chr2:164201552-164201574	164201558	4	DNA	GGC-CGAGGTCGACTACCGGCNRG	GcCTCGAGGaCGtCTtCCGGCTGG	-	4	1	0	6.06	3.03
Chr10:49463482-49463504	49463489	5	RNA	GGCCGAGGTCGACTACCGGCNRG	caCCaAGG--GACTgCCGtCGAG	-	5	2	0	6.06	3.03
Chr5:119153448-119153470	119153466	2	RNA	GGCCGAGGTCGACTACCGGCNRG	GGCCGAGGTgGgtTgCC--tGAG	+	5	2	0	6.06	3.03
Chr10:57989987-57990009	57990002	5	RNA	GGCCGAGGTCGACTACCGGCNRG	GGC--tcGcgGACTACCGtCTGG	+	5	2	0	6.06	3.03
Chr1:257619477-257619499	257619494	3	RNA	GGCCGAGGTCGACTACCGGCNRG	GGCCGAcGagt--gACCGGCTGG	+	5	2	0	6.06	3.03
Chr9:59711973-59711995	59711988	5	RNA	GGCCGAGGTCGACTACCGGCNRG	GtC--tccTtGACTACCGGCCGG	+	5	2	0	6.06	3.03
Chr5:735500-735522	735516	4	RNA	GGCCGAGGTCGACTACCGGCNRG	GGC-GAGGTCGAggACCttgGGG	+	5	1	0	6.06	3.03
Chr3:79080994-79081016	79081010	4	RNA	GGCCGAGGTCGACTACCGGCNRG	GGtCGtGGTCGAC--CgGaaCGG	+	5	2	0	6.06	3.03
Chr5:182735082-182735104	182735086	2	RNA	GGCCGAGGTCGACTACCGGCNRG	GG--GAGGaaGgCgACCGGgAAG	-	5	2	0	6.06	3.03
Chr9:132573667-132573689	132573672	3	RNA	GGCCGAGGTCGACTACCGGCNRG	G-aCGgGGTCGACaACgaGCGGG	-	5	1	0	6.06	3.03
Chr5:61844852-61844874	61844856	2	RNA	GGCCGAGGTCGACTACCGGCNRG	GGCgGAGaTCGtCTtCC--gGGG	-	5	2	0	6.06	3.03
Chr3:199670664-199670686	199670679	5	RNA	GGCCGAGGTCGACTACCGGCNRG	cGC--tcGTgGACTACCGGCTGG	+	4	2	0	6.06	3.03
Chr10:73463374-73463396	73463381	5	RNA	GGCCGAGGTCGACTACCGGCNRG	GGt-GtGGTCGAggACtGGCGAG	-	5	1	0	6.06	3.03
Chr3:45464703-45464725	45464710	5	DNA	GGCCGAGGTCGACTACCGG--CNRG	GGtCGAGGTCGAgTcCgaGCCCTGG	-	5	2	0	6.06	3.03
Chr1:109199431-109199453	109199448	3	RNA	GGCCGAGGTCGACTACCGGCNRG	G--CGAcGTCaAtTACCGcaTGG	+	5	2	0	6.06	3.03
Chr2:106623030-106623052	106623034	2	RNA	GGCCGAGGTCGACTACCGGCNRG	GGCCGAGG-CGcgTgggGGCGGG	-	5	1	0	6.06	3.03
Chr5:55733352-55733374	55733357	3	RNA	GGCCGAGGTCGACTACCGGCNRG	GGgtGgGGgCGA--ACCGGCCGG	-	4	2	0	6.06	3.03
Chr7:52762499-52762521	52762517	2	DNA	GGCC-GAGGTCGACTACCGGCNRG	GGCCAGgGGagGACTACtGGCGGG	+	4	1	0	6.06	3.03
Chr2:40021028-40021050	40021034	4	RNA	GGCCGAGGTCGACTACCGGCNRG	t--CGAGGcCtACacCCGGCGGG	-	5	2	0	6.06	3.03
Chr8:33512836-33512858	33512853	3	DNA	GGCCGAGGTCGACT--ACCGGCNRG	GGtgGAGGTgGgCTGGAgCGGCCGG	+	5	2	0	6.06	3.03
Chr5:180616727-180616749	180616734	5	RNA	GGCCGAGGTCGACTACCGGCNRG	caCCaAGtTCGcCT-CCGGCCAG	-	5	1	0	6.06	3.03
Chr8:45366222-45366244	45366237	5	RNA	GGCCGAGGTCGACTACCGGCNRG	GGgC-AGGcCaACctCCGGCCGG	+	5	1	0	6.06	3.03
Chr9:97776243-97776265	97776250	5	RNA	GGCCGAGGTCGACTACCGGCNRG	GGCCGAatTCaACT-CCtGgAGG	-	5	1	0	6.06	3.03
Chr2:204428188-204428210	204428195	5	RNA	GGCCGAGGTCGACTACCGGCNRG	GGC--AGGcCagCctCCGGCCGG	-	5	2	0	6.06	3.03
Chr9:4324450-4324472	4324455	3	DNA	GG--CCGAGGTCGACTACCGGCNRG	GGATCCaAGaTCcACTtCaGGCAAG	-	5	2	0	6.06	3.03
Chr6:61842309-61842331	61842314	3	RNA	GGCCGAGGTCGACTACCGGCNRG	GGtCGtGcTCGAC-ACgaGCTGG	-	5	1	0	6.06	3.03
Chr8:51395291-51395313	51395309	2	RNA	GGCCGAGGTCGACTACCGGCNRG	GGC--AGcTCGgCTcCCtGCTGG	+	4	2	0	6.06	3.03
Chr2:192273889-192273911	192273893	2	RNA	GGCCGAGGTCGACTACCGGCNRG	GG--GAGcTacACTggCGGCGGG	-	5	2	0	6.06	3.03
Chr2:31312277-31312299	31312295	2	RNA	GGCCGAGGTCGACTACCGGCNRG	GGC--tcGTgGACTACCGaCTGG	+	4	2	0	6.06	3.03
Chr10:21375920-21375942	21375924	2	RNA	GGCCGAGGTCGACTACCGGCNRG	GagtGAGGTCtACTACC--aGAG	-	5	2	0	6.06	3.03
Chr6:77225701-77225723	77225716	5	DNA	GGCCGAGGTCGACTACCGG--CNRG	cGCCGAGGcCGAaagCCGGGCCGAG	+	5	2	0	6.06	3.03
Chr8:59936404-59936426	59936421	3	DNA	GGCCGAGGTCGACTA--CCGGCNRG	GGCgGAGcTtatCTACTCCGGCGAG	+	5	2	0	6.06	3.03
Chr2:84193865-84193887	84193881	4	RNA	GGCCGAGGTCGACTACCGGCNRG	tGCCcAGGTC-ACgACacGCAGG	+	5	1	0	6.06	3.03
Chr4:151080712-151080734	151080727	5	RNA	GGCCGAGGTCGACTACCGGCNRG	tGCgcAGGTCGACgtC--GCGGG	+	5	2	0	6.06	3.03
Chr2:20305841-20305863	20305847	4	RNA	GGCCGAGGTCGACTACCGGCNRG	GGCtcAGG--GgCTACtGtCGGG	-	5	2	0	6.06	3.03
Chr2:151877121-151877143	151877138	3	DNA	G--GCCGAGGTCGACTACCGGCNRG	GATGaCGgGGTCGACcACgaGCGGG	+	5	2	0	6.06	3.03
Chr4:33372328-33372350	33372344	4	RNA	GGCCGAGGTCGACTACCGGCNRG	aGaCGAGGgCGAC--CCtGgCAG	+	5	2	0	6.06	3.03
Chr8:100193135-100193157	100193140	3	RNA	GGCCGAGGTCGACTACCGGCNRG	G-aCGgGGTCGACgACgaGCGGG	-	5	1	0	6.06	3.03
Chr4:161446845-161446867	161446861	4	RNA	GGCCGAGGTCGACTACCGGCNRG	tG--GAGGaaGACgACCtGCAAG	+	5	2	0	6.06	3.03
Chr4:181076698-181076720	181076704	4	RNA	GGCCGAGGTCGACTACCGGCNRG	aGaCGAcGaCGACTAC--GaTAG	-	5	2	0	6.06	3.03
Chr1:170076236-170076258	170076254	2	DNA	GGC-CGAGGTCGACTACCGGCNRG	GcCTCGgGGaCGtCTtCCGGCTGG	+	5	1	0	6.06	3.03
Chr5:166179889-166179911	166179894	3	RNA	GGCCGAGGTCGACTACCGGCNRG	GGC--AGGcCaACctCCGGCCGG	-	4	2	0	6.06	3.03
Chr3:68259948-68259970	68259964	4	RNA	GGCCGAGGTCGACTACCGGCNRG	G-aCGgGGTCGACgACaaGCGGG	+	5	1	0	6.06	3.03
Chr5:20291371-20291393	20291387	4	RNA	GGCCGAGGTCGACTACCGGCNRG	GGgCGAGGgCtA--ACCGatCGG	+	5	2	0	6.06	3.03
Chr1:52972018-52972040	52972034	4	RNA	GGCCGAGGTCGACTACCGGCNRG	GtCCG--GTgcACcACCGGaCAG	+	5	2	0	6.06	3.03
Chr4:234537134-234537156	234537150	4	DNA	GGCCGAGGTCGACTACC--GGCNRG	GGCaGAGGTCGtCggCaGAGGCCAG	+	5	2	0	6.06	3.03
Chr3:106063514-106063536	106063521	5	RNA	GGCCGAGGTCGACTACCGGCNRG	GtCCG--GTgcACcACCGGaCAG	-	5	2	0	6.06	3.03
Chr1:117362439-117362461	117362443	2	RNA	GGCCGAGGTCGACTACCGGCNRG	a--CGAGcTCGACcACCaGaGAG	-	5	2	0	6.06	3.03
Chr7:95664010-95664032	95664028	2	RNA	GGCCGAGGTCGACTACCGGCNRG	GGtCGtGGaCGAgT--CGGCGGG	+	4	2	0	6.06	3.03
Chr8:78627447-78627469	78627462	5	RNA	GGCCGAGGTCGACTACCGGCNRG	G--CaAGGcCGAgctCCGGCAAG	+	5	2	0	6.06	3.03
Chr1:271077305-271077327	271077309	2	RNA	GGCCGAGGTCGACTACCGGCNRG	GGCgGAGGTCG--TtCgGcaGAG	-	5	2	0	6.06	3.03
Chr8:163063070-163063092	163063086	4	RNA	GGCCGAGGTCGACTACCGGCNRG	GGCCGAGG-CtACTcttGGgTGG	+	5	1	0	6.06	3.03
Chr1:230928743-230928765	230928759	4	RNA	GGCCGAGGTCGACTACCGGCNRG	caCCaAGtTCGcCT-CCGGCCAG	+	5	1	0	6.06	3.03
Chr1:162962298-162962320	162962303	3	RNA	GGCCGAGGTCGACTACCGGCNRG	GGCCtAtcTgGACTA--GGtTGG	-	5	2	0	6.06	3.03
Chr8:130470592-130470614	130470608	4	RNA	GGCCGAGGTCGACTACCGGCNRG	G-aCGgGGTCGACaACgaGCGGG	+	5	1	0	6.06	3.03
Chr5:141155973-141155995	141155988	5	RNA	GGCCGAGGTCGACTACCGGCNRG	GaCCGAGtTCagCcACCG-CCAG	+	5	1	0	6.06	3.03
Chr4:155949350-155949372	155949354	2	RNA	GGCCGAGGTCGACTACCGGCNRG	GGCCaAtGTCGACaA--tGCAAG	-	4	2	0	6.06	3.03
Chr5:15533161-15533183	15533179	2	RNA	GGCCGAGGTCGACTACCGGCNRG	GGt-GAGGTCGAgTttCGGgTGG	+	5	1	0	6.06	3.03
Chr1:268746054-268746076	268746072	2	RNA	GGCCGAGGTCGACTACCGGCNRG	GtCCG--GTgcACcACCGGaCAG	+	5	2	0	6.06	3.03
Chr5:106271711-106271733	106271717	4	RNA	GGCCGAGGTCGACTACCGGCNRG	tGaaGAGGc--ACTACCtGCGGG	-	5	2	0	6.06	3.03
Chr3:234332595-234332617	234332599	2	RNA	GGCCGAGGTCGACTACCGGCNRG	GGCCG-GGcCGtCcACtaGCGGG	-	5	1	0	6.06	3.03
Chr4:49010447-49010469	49010463	4	RNA	GGCCGAGGTCGACTACCGGCNRG	G--CGAGGTCGcCcgtCtGCGAG	+	5	2	0	6.06	3.03
Chr10:29619551-29619573	29619566	5	RNA	GGCCGAGGTCGACTACCGGCNRG	GG--GcaGTCGtggACCGGCGAG	+	5	2	0	6.06	3.03
Chr3:211952508-211952530	211952524	4	RNA	GGCCGAGGTCGACTACCGGCNRG	G-aCGgGGTCGACgACgaGCGGG	+	5	1	0	6.06	3.03
Chr8:39542492-39542514	39542496	2	RNA	GGCCGAGGTCGACTACCGGCNRG	GtCgcAGGTCGA-gACCGaCCGG	-	5	1	0	6.06	3.03
Chr10:6663614-6663636	6663631	3	DNA	GGC-CGAGGTCGACTACCGGCNRG	GcCTCGgGGaCGtCTtCCGGCTGG	+	5	1	0	6.06	3.03
Chr6:81702532-81702554	81702536	2	RNA	GGCCGAGGTCGACTACCGGCNRG	GtCCGAa--aGAgTACCGGaAAG	-	5	2	0	6.06	3.03
Chr4:40538516-40538538	40538532	4	DNA	GGCCGAGGTCGACTA--CCGGCNRG	GcCCGAcaTCGcCTACGCCGtCCAG	+	5	2	0	6.06	3.03
Chr6:83379081-83379103	83379088	5	RNA	GGCCGAGGTCGACTACCGGCNRG	G-aCtAGGcgGACcACCGGCGGG	-	5	1	6	0.00	3.00
Chr9:143900795-143900817	143900812	3	RNA	GGCCGAGGTCGACTACCGGCNRG	GcCCGAGGcCGA--ACttGgTGG	+	5	2	6	0.00	3.00
Chr2:62452759-62452781	62452776	3	RNA	GGCCGAGGTCGACTACCGGCNRG	GaatGAGaTCGACTACt--CCAG	+	5	2	6	0.00	3.00
Chr1:239824175-239824197	239824192	3	RNA	GGCCGAGGTCGACTACCGGCNRG	GcCCGAGGcCGA--ACtcGgGGG	+	5	2	6	0.00	3.00
Chr2:183812566-183812588	183812573	5	DNA	GGCCGAGGTCGA-CTACCGGCNRG	GcCCGAGGgaGAGCgACCGaCCGG	-	5	1	6	0.00	3.00
Chr4:44956370-44956392	44956387	3	RNA	GGCCGAGGTCGACTACCGGCNRG	tGC--AGGTgcACggCCGGCAGG	+	5	2	6	0.00	3.00
Chr4:201718685-201718707	201718702	3	RNA	GGCCGAGGTCGACTACCGGCNRG	GctCGAGGTCG-CcgCCGGtGAG	+	5	1	6	0.00	3.00
Chr3:13342931-13342953	13342948	3	RNA	GGCCGAGGTCGACTACCGGCNRG	GGC--AGGcCGAgcACCcGCCGG	+	4	2	6	0.00	3.00
Chr1:267072992-267073014	267072999	5	DNA	GGC--CGAGGTCGACTACCGGCNRG	GGCCTCGgGGgCtACacCCGGCGGG	-	5	2	6	0.00	3.00
Chr2:117524235-117524257	117524252	3	RNA	GGCCGAGGTCGACTACCGGCNRG	cGC--AcaaCGACTACCGcCCGG	+	5	2	6	0.00	3.00
Chr5:138036569-138036591	138036586	3	RNA	GGCCGAGGTCGACTACCGGCNRG	GGC--AGGcCaACcACCGGCCGG	+	3	2	6	0.00	3.00
Chr9:17768554-17768576	17768571	3	RNA	GGCCGAGGTCGACTACCGGCNRG	GGC--AGGcCGggcACCGaCCGG	+	5	2	6	0.00	3.00
Chr2:20891575-20891597	20891582	5	RNA	GGCCGAGGTCGACTACCGGCNRG	cGaCGAGcTta--TACCGGCCGG	-	5	2	6	0.00	3.00
Chr6:159625937-159625959	159625954	3	DNA	G--GCCGAGGTCGACTACCGGCNRG	GAGGCCGAGGcCGAggtCgGGCGAG	+	5	2	6	0.00	3.00
Chr5:24489734-24489756	24489751	3	RNA	GGCCGAGGTCGACTACCGGCNRG	GtCCGAGGaC--CTAaCtGCGAG	+	4	2	6	0.00	3.00
Chr4:927905-927927	927922	3	RNA	GGCCGAGGTCGACTACCGGCNRG	GGCCGcGG-CGACcAaCtGaAAG	+	5	1	6	0.00	3.00
Chr5:18605364-18605386	18605381	3	RNA	GGCCGAGGTCGACTACCGGCNRG	GGCCGAGtaCG--TtgCcGCAGG	+	5	2	6	0.00	3.00
Chr6:100600754-100600776	100600761	5	RNA	GGCCGAGGTCGACTACCGGCNRG	GaCCGAGGTCctCgAC--GaCGG	-	5	2	6	0.00	3.00
Chr3:179281155-179281177	179281162	5	RNA	GGCCGAGGTCGACTACCGGCNRG	GGCCGAGGTgGcCTtC--GCGGG	-	3	2	6	0.00	3.00
Chr1:89413185-89413207	89413192	5	RNA	GGCCGAGGTCGACTACCGGCNRG	aGCCGAGGgC-ACctCgGGCAGG	-	5	1	6	0.00	3.00
Chr8:37497992-37498014	37498009	3	RNA	GGCCGAGGTCGACTACCGGCNRG	GGgCGAGGgCGA-gAatGGCAGG	+	5	1	6	0.00	3.00
Chr8:83380376-83380398	83380393	3	RNA	GGCCGAGGTCGACTACCGGCNRG	GGCCG-GGTCGAgaAgCatCAAG	+	5	1	6	0.00	3.00
Chr4:85007047-85007069	85007054	5	DNA	GGCCGAGGTCG--ACTACCGGCNRG	GcCCGAGGTCGCTtgTACCctCGGG	-	5	2	6	0.00	3.00
Chr4:91487901-91487923	91487918	3	RNA	GGCCGAGGTCGACTACCGGCNRG	GGCCGAGcTtGcCTtC-GcCCAG	+	5	1	6	0.00	3.00
Chr8:134314717-134314739	134314724	5	DNA	GGCCGAGGTCGACTACCG-GCNRG	GGCCGAGGTCGcCTcagGCtCGGG	-	5	1	6	0.00	3.00
Chr1:17752534-17752556	17752551	3	DNA	GGCCGAGGTCGACTAC-CGGCNRG	GaCCaAGGTCaACTgCTCGGaTGG	+	5	1	6	0.00	3.00
Chr1:248317774-248317796	248317781	5	RNA	GGCCGAGGTCGACTACCGGCNRG	GGttGAG--CcACTAaCGtCAGG	-	5	2	6	0.00	3.00
Chr2:33213866-33213888	33213883	3	RNA	GGCCGAGGTCGACTACCGGCNRG	GGCgcAaGTCGAgg--CGGCCAG	+	5	2	6	0.00	3.00
Chr8:75568215-75568237	75568232	3	RNA	GGCCGAGGTCGACTACCGGCNRG	GagCGAG-TCGtCaACCGtCCAG	+	5	1	6	0.00	3.00
Chr8:58903585-58903607	58903592	5	X	GGCCGAGGTCGACTACCGGCNRG	GGCCGAcGgCGACcAaCaGCCAG	-	5	0	6	0.00	3.00
Chr2:147395006-147395028	147395023	3	DNA	GGCCGAGGTC-GACTACCGGCNRG	GGCCGAGGcCTGAggtCgGGCGAG	+	5	1	6	0.00	3.00
Chr2:104049539-104049561	104049546	5	RNA	GGCCGAGGTCGACTACCGGCNRG	tttCGAGGgCGA--ACCGGgCAG	-	5	2	6	0.00	3.00
Chr8:120988300-120988322	120988317	3	RNA	GGCCGAGGTCGACTACCGGCNRG	GGtCGAGa-CGtCcACCaGCCGG	+	5	1	6	0.00	3.00
Chr3:54316439-54316461	54316456	3	DNA	GGCC-GAGGTCGACTACCGGCNRG	tGCCTGAGGTCatCcACaGGCGGG	+	5	1	6	0.00	3.00
Chr3:3896525-3896547	3896532	5	RNA	GGCCGAGGTCGACTACCGGCNRG	aGCCGAGGgtGcCT-CgGGCAGG	-	5	1	6	0.00	3.00
Chr2:20807348-20807370	20807365	3	RNA	GGCCGAGGTCGACTACCGGCNRG	G-CCGgGGTCGgCTcCgGGtCAG	+	5	1	6	0.00	3.00
Chr1:214809013-214809035	214809030	3	RNA	GGCCGAGGTCGACTACCGGCNRG	GGCCGcG--CGtgcACgGGCCAG	+	5	2	6	0.00	3.00
Chr8:34046458-34046480	34046475	3	RNA	GGCCGAGGTCGACTACCGGCNRG	GGCCGgGaT--AtcAtCGGCAGG	+	5	2	6	0.00	3.00
Chr5:165138320-165138342	165138327	5	RNA	GGCCGAGGTCGACTACCGGCNRG	tGCgcAGGTCGACatCC--CTGG	-	5	2	6	0.00	3.00
Chr8:88896833-88896855	88896840	5	RNA	GGCCGAGGTCGACTACCGGCNRG	G-aCGgGGTCGACaACgaGCGGG	-	5	1	6	0.00	3.00
Chr7:65079196-65079218	65079213	3	RNA	GGCCGAGGTCGACTACCGGCNRG	cGgCGgGGaCGA-TAgCGGCAAG	+	5	1	6	0.00	3.00
Chr3:61761079-61761101	61761096	3	RNA	GGCCGAGGTCGACTACCGGCNRG	G--CGAGGTCGAtgAatGGgTAG	+	5	2	6	0.00	3.00
Chr2:187105452-187105474	187105459	5	DNA	GGCCGAGGTCGACTACCGG-CNRG	GaCCGAGGTCGAggcCaGGTCCGG	-	5	1	6	0.00	3.00
Chr1:45735557-45735579	45735574	3	RNA	GGCCGAGGTCGACTACCGGCNRG	GGC--tcGTgGACTACCGGCTGG	+	3	2	6	0.00	3.00
Chr2:8554078-8554100	8554095	3	RNA	GGCCGAGGTCGACTACCGGCNRG	GG--GAGtTCGgCTAggGGCAAG	+	4	2	6	0.00	3.00
Chr4:57838553-57838575	57838560	5	RNA	GGCCGAGGTCGACTACCGGCNRG	GGCCGcG--tGACTACCccaAGG	-	5	2	6	0.00	3.00
Chr10:76813792-76813814	76813809	3	DNA	GGCCGAGGTCGACT-ACCGGCNRG	cGCCGcGGatGACTCACCGGaCAG	+	5	1	6	0.00	3.00
Chr9:21642208-21642230	21642215	5	RNA	GGCCGAGGTCGACTACCGGCNRG	GG--GAGGgCGcgggCCGGCCGG	-	5	2	6	0.00	3.00
Chr2:219367667-219367689	219367684	3	RNA	GGCCGAGGTCGACTACCGGCNRG	GGC--AGGcCGAgcACCaGCCGG	+	4	2	6	0.00	3.00
Chr2:27010006-27010028	27010023	3	RNA	GGCCGAGGTCGACTACCGGCNRG	GcCCGAG-TCGgCTcgCGGtAGG	+	5	1	6	0.00	3.00
Chr4:14797194-14797216	14797201	5	RNA	GGCCGAGGTCGACTACCGGCNRG	GtCCG--GaaGAgTACCGGaAAG	-	5	2	6	0.00	3.00
Chr1:142633277-142633299	142633284	5	RNA	GGCCGAGGTCGACTACCGGCNRG	GGCCGcGGaCagCgAC--GCGGG	-	5	2	6	0.00	3.00
Chr5:78069463-78069485	78069480	3	DNA	GGCCG--AGGTCGACTACCGGCNRG	GGCCGCAAGGTCagtgACCaGCGAG	+	5	2	6	0.00	3.00

Chr1:67539943-67539965	67539950	5	RNA	GGCCGAGGTCGACTACCGGCNRG	GcCCGAGccCGAgT--CGGtAGG	-	5	2	6	0.00	3.00
Chr5:216890104-216890126	216890121	3	RNA	GGCCGAGGTCGACTACCGGCNRG	GG--GAaGTCGACTAtCtcCAAG	+	4	2	6	0.00	3.00
Chr4:65705658-65705680	65705675	3	RNA	GGCCGAGGTCGACTACCGGCNRG	GGCgGAGGT-GctTACCaGgAGG	+	5	1	6	0.00	3.00
Chr8:95257752-95257774	95257769	3	RNA	GGCCGAGGTCGACTACCGGCNRG	GaCCGAGG-CGACgACaaGaAGG	+	5	1	6	0.00	3.00
Chr6:159611040-159611062	159611057	3	RNA	GGCCGAGGTCGACTACCGGCNRG	cGtCGAGGTC--CTcCCaGgTAG	+	5	2	6	0.00	3.00
Chr7:106612436-106612458	106612443	5	RNA	GGCCGAGGTCGACTACCGGCNRG	GGC-GtGGTCGAggACtGGCGGG	-	4	1	6	0.00	3.00
Chr2:131651307-131651329	131651314	5	RNA	GGCCGAGGTCGACTACCGGCNRG	GGgCGAG--CGctgACaGGCGGG	-	5	2	6	0.00	3.00
Chr2:63726311-63726333	63726318	5	RNA	GGCCGAGGTCGACTACCGGCNRG	aGCCtAtGTCGACTACC--tCGG	-	4	2	6	0.00	3.00
Chr3:132856517-132856539	132856534	3	DNA	GGC--CGAGGTCGACTACCGGCNRG	GGCGTCGAGGTCaACggCCGGgCGG	+	4	2	6	0.00	3.00
Chr5:211208289-211208311	211208296	5	RNA	GGCCGAGGTCGACTACCGGCNRG	GcCCGAcGTC--CTcCCaGgTGG	-	5	2	6	0.00	3.00
Chr5:99721071-99721093	99721078	5	RNA	GGCCGAGGTCGACTACCGGCNRG	GGgCGgGG-CGACcACacGCGGG	-	5	1	6	0.00	3.00
Chr2:191038543-191038565	191038550	5	RNA	GGCCGAGGTCGACTACCGGCNRG	cGCCGccGTCGAC--CtGGtTAG	-	5	2	6	0.00	3.00
Chr9:20794407-20794429	20794414	5	RNA	GGCCGAGGTCGACTACCGGCNRG	G-CCtcGGgCGAgTACtGGCAGG	-	5	1	6	0.00	3.00
Chr3:12643969-12643991	12643976	5	DNA	GGCCGAGGTCGACTACCG-GCNRG	GGaCGgaGTCGtCTACCGGGgTGG	-	5	1	6	0.00	3.00
Chr1:146155367-146155389	146155384	3	RNA	GGCCGAGGTCGACTACCGGCNRG	G-CCtcGGgCGAgTACtGGCAGG	+	5	1	6	0.00	3.00
Chr2:72470284-72470306	72470301	3	DNA	GGCCGA--GGTCGACTACCGGCNRG	GGgtGACCGaaCGACgACCGGCAAG	+	5	2	6	0.00	3.00
Chr1:190346228-190346250	190346245	3	RNA	GGCCGAGGTCGACTACCGGCNRG	GGaCcAGGTCG--TcCCaGtTGG	+	5	2	6	0.00	3.00
Chr3:70899041-70899063	70899048	5	RNA	GGCCGAGGTCGACTACCGGCNRG	GaCCGAGGaCttCTACCG-CGGG	-	4	1	6	0.00	3.00
Chr9:77411136-77411158	77411143	5	RNA	GGCCGAGGTCGACTACCGGCNRG	G-CCGAGGTtGgCctCgGGCAAG	-	5	1	6	0.00	3.00
Chr1:108825688-108825710	108825705	3	RNA	GGCCGAGGTCGACTACCGGCNRG	G-CCGAaGTacACcACCtGCTGG	+	5	1	6	0.00	3.00
Chr2:225361920-225361942	225361927	5	RNA	GGCCGAGGTCGACTACCGGCNRG	actgGcGGTCGACT-CCGGCCGG	-	5	1	6	0.00	3.00
Chr5:71895882-71895904	71895889	5	RNA	GGCCGAGGTCGACTACCGGCNRG	GGCCGAaGTCGgCaACC--tTGG	-	4	2	6	0.00	3.00
Chr10:93666644-93666666	93666651	5	RNA	GGCCGAGGTCGACTACCGGCNRG	GctCGAaGcC--CTAaCGGCAGG	-	5	2	6	0.00	3.00
Chr3:34385661-34385683	34385678	3	RNA	GGCCGAGGTCGACTACCGGCNRG	G--CGAGcTCatCggCCGGCCGG	+	5	2	6	0.00	3.00
Chr6:113142918-113142940	113142935	3	RNA	GGCCGAGGTCGACTACCGGCNRG	atCCtgGGT-GACcACCGGCGGG	+	5	1	6	0.00	3.00
Chr4:141664652-141664674	141664669	3	RNA	GGCCGAGGTCGACTACCGGCNRG	G-aCGgGGTCGACaACgaGCGGG	+	5	1	6	0.00	3.00
Chr1:132746271-132746293	132746288	3	RNA	GGCCGAGGTCGACTACCGGCNRG	GGaCGtcGTCGACcgCCG-CGGG	+	5	1	6	0.00	3.00
Chr3:59560648-59560670	59560665	3	RNA	GGCCGAGGTCGACTACCGGCNRG	GGaCGgtcTCGACT-CCGGtTGG	+	5	1	6	0.00	3.00
Chr9:118995348-118995370	118995365	3	DNA	GGCCGAGGTCGACTAC-CGGCNRG	GGCCGAGGctGACacCGaGGCCGG	+	5	1	6	0.00	3.00
Chr1:35071769-35071791	35071776	5	X	GGCCGAGGTCGACTACCGGCNRG	GGCgGtGGTCGgCgAgCGGCGGG	-	5	0	6	0.00	3.00
Chr10:99023149-99023171	99023156	5	RNA	GGCCGAGGTCGACTACCGGCNRG	GGC--tcGcaGACTACCGGCTGG	-	4	2	6	0.00	3.00
Chr6:43494395-43494417	43494402	5	DNA	GGCCGAGGTCGACT-ACCGGCNRG	GGgCGcGGctGACTCACCGGaTAG	-	5	1	6	0.00	3.00
Chr5:185200067-185200089	185200084	3	RNA	GGCCGAGGTCGACTACCGGCNRG	cGCgcAcGTCGACTgCC--CCGG	+	5	2	6	0.00	3.00
Chr10:142691979-142692001	142691986	5	DNA	GGC--CGAGGTCGACTACCGGCNRG	GGCCTCGgGGgCtACacCCGGCGGG	-	5	2	6	0.00	3.00
Chr4:225409632-225409654	225409649	3	RNA	GGCCGAGGTCGACTACCGGCNRG	GGCCGAGtcCG-CgACCaGaTAG	+	5	1	6	0.00	3.00
Chr1:123925346-123925368	123925353	5	RNA	GGCCGAGGTCGACTACCGGCNRG	GGCCttGtTCGctTA--GGCGGG	-	5	2	6	0.00	3.00
Chr2:124766213-124766235	124766230	3	DNA	GGC--CGAGGTCGACTACCGGCNRG	GGCGGCGtGGTCGACggCgaGCAAG	+	5	2	6	0.00	3.00
Chr5:178180723-178180745	178180740	3	RNA	GGCCGAGGTCGACTACCGGCNRG	t--CGgGGaCGtCTtCCGGCTGG	+	5	2	6	0.00	3.00
Chr8:106889847-106889869	106889864	3	RNA	GGCCGAGGTCGACTACCGGCNRG	G-aCGgGGTCGACaACgaGCGGG	+	5	1	6	0.00	3.00
Chr7:46237825-46237847	46237842	3	RNA	GGCCGAGGTCGACTACCGGCNRG	GG--GAGtTCcACTcaaGGCAGG	+	5	2	6	0.00	3.00
Chr2:157494426-157494448	157494433	5	RNA	GGCCGAGGTCGACTACCGGCNRG	GGgCGAtGaCGACgAC--GgAGG	-	5	2	6	0.00	3.00
Chr9:14443523-14443545	14443540	3	DNA	GGCCGAG--GTCGACTACCGGCNRG	GttCGAGATGTCGAtTAtgGGCGAG	+	5	2	6	0.00	3.00
Chr8:94676777-94676799	94676784	5	RNA	GGCCGAGGTCGACTACCGGCNRG	GGC--tcGagGACTACCGGCTGG	-	4	2	6	0.00	3.00
Chr2:118140559-118140581	118140566	5	RNA	GGCCGAGGTCGACTACCGGCNRG	cGCCaAaGaCGACTgC--GCGGG	-	5	2	6	0.00	3.00
Chr9:81776195-81776217	81776212	3	RNA	GGCCGAGGTCGACTACCGGCNRG	GGgCG-GGcCaACccCCGGCCGG	+	5	1	6	0.00	3.00
Chr4:155102967-155102989	155102984	3	RNA	GGCCGAGGTCGACTACCGGCNRG	ttCCGAtGTCGAtTg--GGCAGG	+	5	2	6	0.00	3.00
Chr2:40746979-40747001	40746986	5	RNA	GGCCGAGGTCGACTACCGGCNRG	GcCCGAGGcCGA--ACtcGgTGG	-	5	2	6	0.00	3.00
Chr5:38850092-38850114	38850099	5	RNA	GGCCGAGGTCGACTACCGGCNRG	tG--GAGGTtGcCaACCGGaTAG	-	5	2	6	0.00	3.00
Chr2:199881248-199881270	199881255	5	RNA	GGCCGAGGTCGACTACCGGCNRG	t--CGgGGaCGtCTtCCGGCTGG	-	5	2	6	0.00	3.00
Chr9:52723075-52723097	52723092	3	RNA	GGCCGAGGTCGACTACCGGCNRG	GGCaGAG--CGAtTAgaaGCTAG	+	5	2	6	0.00	3.00
Chr6:135676726-135676748	135676743	3	RNA	GGCCGAGGTCGACTACCGGCNRG	aaCCGAGcTCG--gAaCGGCGGG	+	5	2	6	0.00	3.00
Chr2:149630337-149630359	149630354	3	RNA	GGCCGAGGTCGACTACCGGCNRG	GGgC-AGGcCaACctCCGGCCGG	+	5	1	6	0.00	3.00
Chr8:120335079-120335101	120335086	5	RNA	GGCCGAGGTCGACTACCGGCNRG	GGC--ttGcgGACTACCGGCTGG	-	4	2	6	0.00	3.00
Chr5:88785285-88785307	88785302	3	RNA	GGCCGAGGTCGACTACCGGCNRG	GGaCcAGtTCGACTA--aGtCAG	+	5	2	6	0.00	3.00
Chr5:211083589-211083611	211083606	3	RNA	GGCCGAGGTCGACTACCGGCNRG	GcCgGAGccCGAC--CCGtCAAG	+	5	2	6	0.00	3.00
Chr8:60853116-60853138	60853133	3	RNA	GGCCGAGGTCGACTACCGGCNRG	GGC--tcGcgGACTAtCGGCTGG	+	5	2	6	0.00	3.00
Chr4:38855777-38855799	38855794	3	RNA	GGCCGAGGTCGACTACCGGCNRG	GGC--AGGcCGAgcACCGGCCGG	+	3	2	6	0.00	3.00
Chr2:103752146-103752168	103752163	3	DNA	GGCCGAGGTCGACTACC--GGCNRG	GGCCGcGGTaGACTAaCCTcGaCGG	+	5	2	6	0.00	3.00
Chr4:22135726-22135748	22135743	3	RNA	GGCCGAGGTCGACTACCGGCNRG	cGtCGAGtTCGcCT-CCGcCGGG	+	5	1	6	0.00	3.00
Chr1:281412146-281412168	281412153	5	DNA	GGC--CGAGGTCGACTACCGGCNRG	GGCCTCGgGGgCtACacCCGGCGGG	-	5	2	6	0.00	3.00
Chr9:55197101-55197123	55197108	5	RNA	GGCCGAGGTCGACTACCGGCNRG	GGC-GAccTCGgCTtCCtGCTGG	-	5	1	6	0.00	3.00
Chr2:119960514-119960536	119960531	3	DNA	G-GCCGAGGTCGACTACCGGCNRG	GAGCtGAGcaCGgCcACCGGCGGG	+	5	1	6	0.00	3.00
Chr10:76266404-76266426	76266411	5	RNA	GGCCGAGGTCGACTACCGGCNRG	GcCgGAGccCGAC--CCGtCAAG	-	5	2	6	0.00	3.00
Chr4:69757015-69757037	69757022	5	RNA	GGCCGAGGTCGACTACCGGCNRG	G-aCGAGGTCGACaACgaGCGGG	-	4	1	6	0.00	3.00
Chr2:29891318-29891340	29891325	5	RNA	GGCCGAGGTCGACTACCGGCNRG	GG--GAGcTCcACTACtGGgAAG	-	4	2	6	0.00	3.00
Chr8:38341080-38341102	38341087	5	DNA	GGCCGAGGTCGACT-ACCGGCNRG	GGgCGtGGgCGACTGACaGGgGGG	-	5	1	6	0.00	3.00
Chr3:231393788-231393810	231393795	5	RNA	GGCCGAGGTCGACTACCGGCNRG	G-tCGccGTCGtCgACCGGCGGG	-	5	1	6	0.00	3.00
Chr2:116690125-116690147	116690132	5	RNA	GGCCGAGGTCGACTACCGGCNRG	GGC-atGGTCGAggACtGGCGGG	-	5	1	6	0.00	3.00
Chr2:5869429-5869451	5869436	5	RNA	GGCCGAGGTCGACTACCGGCNRG	GGCCGAGGgCtA-gACatGCCGG	-	5	1	6	0.00	3.00
Chr6:97775764-97775786	97775781	3	RNA	GGCCGAGGTCGACTACCGGCNRG	aGCCGAGcTC--CTtCCGcgGAG	+	5	2	6	0.00	3.00
Chr9:61102069-61102091	61102076	5	RNA	GGCCGAGGTCGACTACCGGCNRG	tG--GAGGagGACgACCtGCTGG	-	5	2	6	0.00	3.00
Chr1:276604348-276604370	276604355	5	RNA	GGCCGAGGTCGACTACCGGCNRG	GaagGAGGTC-AtgACCGGCAAG	-	5	1	6	0.00	3.00
Chr5:65822139-65822161	65822146	5	RNA	GGCCGAGGTCGACTACCGGCNRG	GGC--tcGcgGACTACCGtCTGG	-	5	2	6	0.00	3.00
Chr10:50542966-50542988	50542983	3	RNA	GGCCGAGGTCGACTACCGGCNRG	GGC--AGGcCGAgcACtaGCCGG	+	5	2	6	0.00	3.00
Chr7:1563558-1563580	1563565	5	RNA	GGCCGAGGTCGACTACCGGCNRG	aGCCGAGGatGACcAC-GtCCAG	-	5	1	6	0.00	3.00
Chr5:218328664-218328686	218328671	5	RNA	GGCCGAGGTCGACTACCGGCNRG	GaCC-AGGTCGtCTcCCaGtAGG	-	5	1	6	0.00	3.00
Chr3:210170747-210170769	210170764	3	RNA	GGCCGAGGTCGACTACCGGCNRG	GGCCGtGcTtG--TcCCGGaGGG	+	5	2	6	0.00	3.00
Chr9:65079073-65079095	65079090	3	RNA	GGCCGAGGTCGACTACCGGCNRG	GGC--AGGcCaACctCCGGCCGG	+	4	2	6	0.00	3.00
Chr6:72911352-72911374	72911368	4	RNA	GGCCGAGGTCGACTACCGGCNRG	G-aCGgGGTCGACaACgaGCGGG	+	5	1	1	4.96	2.98
Chr3:62192763-62192785	62192779	4	RNA	GGCCGAGGTCGACTACCGGCNRG	cGCCGAGtcCGAaTAtC--CTAG	+	5	2	1	4.96	2.98
Chr2:33514086-33514108	33514092	4	RNA	GGCCGAGGTCGACTACCGGCNRG	GGC--AGGctaACctCCGGCCGG	-	5	2	2	3.86	2.93
Chr4:191426183-191426205	191426189	4	RNA	GGCCGAGGTCGACTACCGGCNRG	caCCaAGtTCGcCT-CCGGCCAG	-	5	1	2	3.86	2.93
Chr5:82350877-82350899	82350882	3	RNA	GGCCGAGGTCGACTACCGGCNRG	G-tCGAGGcCcACTACCGaCGGG	-	4	1	2	3.86	2.93
Chr1:73313951-73313973	73313957	4	RNA	GGCCGAGGTCGACTACCGGCNRG	GGC-GAGGTCGACggCgaGaTGG	-	5	1	3	2.75	2.88
Chr10:60613212-60613234	60613219	5	RNA	GGCCGAGGTCGACTACCGGCNRG	GGCgcAGcTCaACT-CCGGCGAG	-	4	1	4	1.65	2.83
Chr10:103260982-103261004	103261000	2	RNA	GGCCGAGGTCGACTACCGGCNRG	GG--GAcGaCGACgAtCtGCTAG	+	5	2	4	1.65	2.83
Chr3:123060771-123060793	123060776	3	RNA	GGCCGAGGTCGACTACCGGCNRG	atCCGAGaTCGAt--CgGGCGAG	-	5	2	5	0.55	2.78
Chr3:27939723-27939745	27939728	3	DNA	GGCCGAGGTCGACTAC-CGGCNRG	cGaCGAGGTCGACcACGCGGtCGG	-	4	1	0	5.51	2.76
Chr4:32708440-32708462	32708458	2	RNA	GGCCGAGGTCGACTACCGGCNRG	cGgCGAGGaCGA--ACaGGgGAG	+	5	2	0	5.51	2.76
Chr10:24810560-24810582	24810577	3	RNA	GGCCGAGGTCGACTACCGGCNRG	GG--GcGGTCaACTgCtGaCAGG	+	5	2	0	5.51	2.76
Chr2:161671483-161671505	161671500	3	RNA	GGCCGAGGTCGACTACCGGCNRG	GGCCGAGGTaGgCaAta--CTGG	+	5	2	0	5.51	2.76
Chr8:131056168-131056190	131056184	4	RNA	GGCCGAGGTCGACTACCGGCNRG	GGC--tcGcgGACTACCGGCTGG	+	4	2	0	5.51	2.76
Chr5:39204058-39204080	39204074	4	RNA	GGCCGAGGTCGACTACCGGCNRG	GGaCcAGGTCG--TcCCaGtTGG	+	5	2	0	5.51	2.76
Chr5:114002789-114002811	114002794	3	DNA	GGC--CGAGGTCGACTACCGGCNRG	GaCAACGgGGTCGACgACgaGCGGG	-	5	2	0	5.51	2.76
Chr4:12783698-12783720	12783716	2	DNA	GGCCGAGGTCGACT-ACCGGCNRG	GGgCGAGcgCGtCTGACaGGCGAG	+	5	1	0	5.51	2.76
Chr8:94701654-94701676	94701659	3	RNA	GGCCGAGGTCGACTACCGGCNRG	GaCCGAGG-CGACgACaaGaAGG	-	5	1	0	5.51	2.76
Chr4:160175695-160175717	160175710	5	RNA	GGCCGAGGTCGACTACCGGCNRG	GGC--tcGcaGACTACCGGCTGG	+	4	2	0	5.51	2.76
Chr9:51977210-51977232	51977228	2	DNA	GGCCGAGGTCGACTACC--GGCNRG	GcCCGAGcaCGACcACCGTGGCCAG	+	4	2	0	5.51	2.76
Chr2:24626185-24626207	24626202	3	RNA	GGCCGAGGTCGACTACCGGCNRG	GGC--tcGcgGACTACCGGCTGG	+	4	2	0	5.51	2.76
Chr8:136445037-136445059	136445055	2	RNA	GGCCGAGGTCGACTACCGGCNRG	GGCCG--GTCtgCcAgCGGgAGG	+	5	2	0	5.51	2.76
Chr8:124912135-124912157	124912142	5	DNA	GGCCGAGGTCGACTACCGG-CNRG	GGCCGAGGTCGgCactCGGCaAAG	-	5	1	0	5.51	2.76
Chr6:91836601-91836623	91836605	2	RNA	GGCCGAGGTCGACTACCGGCNRG	GGC-GAGcTCcACcAtCtGCCGG	-	5	1	0	5.51	2.76
Chr8:143752936-143752958	143752952	4	RNA	GGCCGAGGTCGACTACCGGCNRG	GG--GAGGctGtCTgCCGaCGGG	+	5	2	0	5.51	2.76
Chr5:20481950-20481972	20481966	4	DNA	GGC-CGAGGTCGACTACCGGCNRG	GcCTCGgGGaCGtCTtCCGGCTGG	+	5	1	0	5.51	2.76
Chr1:70047759-70047781	70047764	3	RNA	GGCCGAGGTCGACTACCGGCNRG	GGtCG--GTCGgCTACCcGaCGG	-	4	2	0	5.51	2.76
Chr3:44982986-44983008	44982992	4	RNA	GGCCGAGGTCGACTACCGGCNRG	GaCCGgGGTCGcCcA--GaCGGG	-	5	2	0	5.51	2.76
Chr5:102052323-102052345	102052330	5	RNA	GGCCGAGGTCGACTACCGGCNRG	cGaCGAtGgCGAC--CtGGCCAG	-	5	2	0	5.51	2.76
Chr9:72567266-72567288	72567273	5	RNA	GGCCGAGGTCGACTACCGGCNRG	GGtCGgGGT--AactCCGGCGAG	-	5	2	0	5.51	2.76
Chr7:139865706-139865728	139865713	5	RNA	GGCCGAGGTCGACTACCGGCNRG	GGCtGAacTCG-CTACCttCGGG	-	5	1	0	5.51	2.76
Chr2:136391856-136391878	136391873	3	RNA	GGCCGAGGTCGACTACCGGCNRG	GGaCtAGGTCG--TcCCaGtTGG	+	5	2	0	5.51	2.76
Chr5:34201656-34201678	34201661	3	RNA	GGCCGAGGTCGACTACCGGCNRG	GGtCGAGGTCG--TggCcGaGGG	-	5	2	0	5.51	2.76
Chr1:205218340-205218362	205218358	2	RNA	GGCCGAGGTCGACTACCGGCNRG	GGt--AGGcCGAgcACCaGCCGG	+	5	2	0	5.51	2.76
Chr2:63390237-63390259	63390242	3	RNA	GGCCGAGGTCGACTACCGGCNRG	GGC--AcGTCGACggCgGGaTGG	-	5	2	0	5.51	2.76
Chr1:102169381-102169403	102169386	3	RNA	GGCCGAGGTCGACTACCGGCNRG	tGCCGA--TacACcgCCGGCCGG	-	5	2	0	5.51	2.76
Chr7:126216859-126216881	126216866	5	DNA	GGCCGAGGT-CGACTACCGGCNRG	GGCgGAGGTCCGAtcACCGGCGAG	-	3	1	0	5.51	2.76
Chr1:253412138-253412160	253412143	3	RNA	GGCCGAGGTCGACTACCGGCNRG	G-aCGgGGTCGACaACgaGCGGG	-	5	1	0	5.51	2.76
Chr6:155049463-155049485	155049479	4	RNA	GGCCGAGGTCGACTACCGGCNRG	caCCaAGtTCGcCT-CCGGCCAG	+	5	1	0	5.51	2.76
Chr10:34498204-34498226	34498221	3	RNA	GGCCGAGGTCGACTACCGGCNRG	cGCCGcGGTCGcCa--CGGtAGG	+	5	2	0	5.51	2.76
Chr3:127034085-127034107	127034103	2	RNA	GGCCGAGGTCGACTACCGGCNRG	GGC--AGGagccCTACCGcCAAG	+	5	2	0	5.51	2.76
Chr4:91166567-91166589	91166583	4	DNA	GGCCGAGGTC--GACTACCGGCNRG	GGCCGAGaTCTGGtCTAggGaCGAG	+	5	2	0	5.51	2.76
Chr7:121933023-121933045	121933030	5	RNA	GGCCGAGGTCGACTACCGGCNRG	GGgCGAc--CGAagtCCGGCGAG	-	5	2	0	5.51	2.76
Chr10:24222972-24222994	24222978	4	RNA	GGCCGAGGTCGACTACCGGCNRG	GGC--ttGcgGACTACCGGCTGG	-	4	2	0	5.51	2.76
Chr4:146867394-146867416	146867412	2	RNA	GGCCGAGGTCGACTACCGGCNRG	cGCCcAGGTgcACg--CGGCAAG	+	5	2	0	5.51	2.76
Chr9:52196348-52196370	52196364	4	RNA	GGCCGAGGTCGACTACCGGCNRG	G-aCGgGGTCGACaACgaGCGGG	+	5	1	0	5.51	2.76
Chr1:49000424-49000446	49000441	3	DNA	GGC--CGAGGTCGACTACCGGCNRG	GGCCTCGgGGgCtACacCCGGCGGG	+	5	2	0	5.51	2.76
Chr5:30550117-30550139	30550134	3	DNA	GGC-CGAGGTCGACTACCGGCNRG	GcCTCGAGGaCGtCTtCCGGCTGG	+	4	1	0	5.51	2.76

Chr5:76511711-76511733	76511729	2	RNA	GGCCGAGGTCGACTACCGGCNRG	GaCCGAGG-CGACgACaaGaAGG	+	5	1	0	5.51	2.76
Chr8:65327322-65327344	65327326	2	RNA	GGCCGAGGTCGACTACCGGCNRG	cGCCGAcGTCGAaTcCCG-aGAG	-	5	1	0	5.51	2.76
Chr10:64853316-64853338	64853334	2	RNA	GGCCGAGGTCGACTACCGGCNRG	GGCgGAGcagGACT--CGcCAAG	+	5	2	0	5.51	2.76
Chr4:50998463-50998485	50998467	2	RNA	GGCCGAGGTCGACTACCGGCNRG	GagaGgGGTCGgCT--CGGCCGG	-	5	2	0	5.51	2.76
Chr5:138899230-138899252	138899246	4	RNA	GGCCGAGGTCGACTACCGGCNRG	ttCCGAtGTCGAtTA--tGCAGG	+	5	2	0	5.51	2.76
Chr2:18898307-18898329	18898311	2	RNA	GGCCGAGGTCGACTACCGGCNRG	cGaCGcGGTCG--cACaGGCTGG	-	5	2	0	5.51	2.76
Chr4:45282082-45282104	45282098	4	RNA	GGCCGAGGTCGACTACCGGCNRG	G-aCGgGGTCGACaACgaGCGGG	+	5	1	0	5.51	2.76
Chr7:10962590-10962612	10962597	5	RNA	GGCCGAGGTCGACTACCGGCNRG	GGaCGAcccCGACT-CCGGtTGG	-	5	1	0	5.51	2.76
Chr4:204318800-204318822	204318804	2	RNA	GGCCGAGGTCGACTACCGGCNRG	G--CGtGGTCGAtgACtGaCGGG	-	5	2	0	5.51	2.76
Chr4:20023417-20023439	20023435	2	RNA	GGCCGAGGTCGACTACCGGCNRG	GGCCaAGGTCGAgg--CGttGGG	+	5	2	0	5.51	2.76
Chr3:152334774-152334796	152334780	4	RNA	GGCCGAGGTCGACTACCGGCNRG	GGC--AGGcCGAgcACtaGCCGG	-	5	2	0	5.51	2.76
Chr1:111975106-111975128	111975111	3	DNA	GGC--CGAGGTCGACTACCGGCNRG	GaCAACGgGGTCGACgACgaGCGGG	-	5	2	0	5.51	2.76
Chr4:173332208-173332230	173332226	2	RNA	GGCCGAGGTCGACTACCGGCNRG	GGCgGAGGTgGAgg--CGGtGGG	+	5	2	0	5.51	2.76
Chr2:194479906-194479928	194479910	2	RNA	GGCCGAGGTCGACTACCGGCNRG	tGtaGAGtT-GAaTACCGGCTGG	-	5	1	0	5.51	2.76
Chr5:57230321-57230343	57230339	2	RNA	GGCCGAGGTCGACTACCGGCNRG	GGCCGccGcCGtCT--CtGCTGG	+	5	2	0	5.51	2.76
Chr5:112241586-112241608	112241602	4	RNA	GGCCGAGGTCGACTACCGGCNRG	GGCCctGGTCGAC-ggCGGCGGG	+	4	1	0	5.51	2.76
Chr2:122542398-122542420	122542405	5	RNA	GGCCGAGGTCGACTACCGGCNRG	GGCgcAacTCtACT-CCGGCGAG	-	5	1	0	5.51	2.76
Chr8:75250363-75250385	75250369	4	RNA	GGCCGAGGTCGACTACCGGCNRG	GGCCGAGGgtGcCTtg-GGCTGG	-	5	1	0	5.51	2.76
Chr4:8562958-8562980	8562964	4	DNA	GGCC-GAGGTCGACTACCGGCNRG	aGCCGGAccTCGACTACCGcCAAG	-	4	1	0	5.51	2.76
Chr5:145171136-145171158	145171151	5	RNA	GGCCGAGGTCGACTACCGGCNRG	G-CCGAaGTacACTgCCtGCTGG	+	5	1	0	5.51	2.76
Chr2:108353742-108353764	108353758	4	RNA	GGCCGAGGTCGACTACCGGCNRG	GaC--AGGTCGACcAaCtGaGAG	+	5	2	0	5.51	2.76
Chr6:139324076-139324098	139324093	3	RNA	GGCCGAGGTCGACTACCGGCNRG	GcCgGAGccCGAC--CCGtCAAG	+	5	2	0	5.51	2.76
Chr1:95966370-95966392	95966375	3	DNA	GGCCGAGGTCGACTACCG-GCNRG	GGCCGAGGcCGACcAtgGAGtAGG	-	5	1	0	5.51	2.76
Chr7:116767343-116767365	116767347	2	DNA	GGCCGAGGTCGACTAC-CGGCNRG	GGCCGgGGcCGAgggCTCGGCGGG	-	5	1	0	5.51	2.76
Chr8:102046492-102046514	102046510	2	RNA	GGCCGAGGTCGACTACCGGCNRG	GGCC-AGGTCGtCcACtGGCGGG	+	3	1	0	5.51	2.76
Chr10:105155678-105155700	105155683	3	RNA	GGCCGAGGTCGACTACCGGCNRG	GcCCGgGGaaG--TACCcGCTGG	-	5	2	0	5.51	2.76
Chr4:85296426-85296448	85296441	5	DNA	GGCC--GAGGTCGACTACCGGCNRG	GaCCTGGAGGTacACTACCacCCAG	+	5	2	0	5.51	2.76
Chr7:96700804-96700826	96700820	4	RNA	GGCCGAGGTCGACTACCGGCNRG	GGaCcAGGTCGAggAC-GGCGGG	+	4	1	0	5.51	2.76
Chr8:106926614-106926636	106926631	3	DNA	GGCC--GAGGTCGACTACCGGCNRG	GGCCCGGcGcTCGACcttCGGCAGG	+	5	2	0	5.51	2.76
Chr8:22618385-22618407	22618389	2	DNA	GGCCGAGGTCGACTA-CCGGCNRG	GGCCaAGGTCGACgAGgaaGCAGG	-	5	1	0	5.51	2.76
Chr9:100306060-100306082	100306075	5	RNA	GGCCGAGGTCGACTACCGGCNRG	GGagGAGcgCGAC--CCcGCGGG	+	5	2	0	5.51	2.76
Chr1:218649619-218649641	218649625	4	RNA	GGCCGAGGTCGACTACCGGCNRG	tcCCGAG--CagCTAaCGGCGAG	-	5	2	0	5.51	2.76
Chr4:35292644-35292666	35292662	2	RNA	GGCCGAGGTCGACTACCGGCNRG	aGaCaAGGTC--CTACaGGCCAG	+	4	2	0	5.51	2.76
Chr2:214053615-214053637	214053620	3	RNA	GGCCGAGGTCGACTACCGGCNRG	G-aCGgGGTCGACaACgaGCGGG	-	5	1	0	5.51	2.76
Chr8:47814225-47814247	47814231	4	RNA	GGCCGAGGTCGACTACCGGCNRG	GG--GcGGcCGtggACCGGCGAG	-	5	2	0	5.51	2.76
Chr3:158011792-158011814	158011808	4	RNA	GGCCGAGGTCGACTACCGGCNRG	G-aCGgGGTCGACaACgaGCGGG	+	5	1	0	5.51	2.76
Chr9:76069600-76069622	76069604	2	DNA	GGCCGAGGTCGACTACCGG--CNRG	GGCCGctGTgGgaTACCGGTCCCGG	-	5	2	0	5.51	2.76
Chr5:50499058-50499080	50499063	3	RNA	GGCCGAGGTCGACTACCGGCNRG	tGCtGgGGTCccCTACC--CTGG	-	5	2	0	5.51	2.76
Chr4:117007892-117007914	117007908	4	RNA	GGCCGAGGTCGACTACCGGCNRG	GtCCGcGGTC-ACcACCGGCCGG	+	3	1	0	5.51	2.76
Chr8:118315427-118315449	118315443	4	X	GGCCGAGGTCGACTACCGGCNRG	GGaCGAcGaCGACTAgCaGCAGG	+	5	0	0	5.51	2.76
Chr3:64552259-64552281	64552277	2	DNA	GGCCGAGGTCGACT-ACCGGCNRG	GGggGAGGagaACTCACCGGCGGG	+	5	1	0	5.51	2.76
Chr5:111781100-111781122	111781107	5	RNA	GGCCGAGGTCGACTACCGGCNRG	GGCacAacTCaACT-CCGGCGAG	-	5	1	0	5.51	2.76
Chr4:11473236-11473258	11473240	2	RNA	GGCCGAGGTCGACTACCGGCNRG	aGgttAGGTCGACaA--GGCCGG	-	5	2	0	5.51	2.76
Chr7:129386860-129386882	129386865	3	RNA	GGCCGAGGTCGACTACCGGCNRG	G-aCGAGGTCGACaACgaGCGGG	-	4	1	1	4.41	2.71
Chr9:130431026-130431048	130431031	3	RNA	GGCCGAGGTCGACTACCGGCNRG	G-aCGgGGTCGACaACgaGCGGG	-	5	1	2	3.30	2.65
Chr2:74528155-74528177	74528172	3	DNA	GGC-CGAGGTCGACTACCGGCNRG	GcCTCGAGGaCGtCTtCCGGCTGG	+	4	1	2	3.30	2.65
Chr1:183300743-183300765	183300749	4	RNA	GGCCGAGGTCGACTACCGGCNRG	G-CCtcGGgCGAaTACtGGCAGG	-	5	1	3	2.20	2.60
Chr2:151640455-151640477	151640460	3	RNA	GGCCGAGGTCGACTACCGGCNRG	G-aCGgGGTCGACgACgaGCGGG	-	5	1	4	1.10	2.55
Chr5:218406195-218406217	218406212	3	RNA	GGCCGAGGTCGACTACCGGCNRG	cG--GAGGggGAaTACCcGCTGG	+	5	2	4	1.10	2.55
Chr7:172676964-172676986	172676981	3	RNA	GGCCGAGGTCGACTACCGGCNRG	tGCCGAGtTgG--TcCCtGCTGG	+	5	2	5	0.00	2.50
Chr9:7785646-7785668	7785653	5	DNA	GGCCGAGG--TCGACTACCGGCNRG	cGgCGAGGCATCGACcACCtGaGAG	-	5	2	5	0.00	2.50
Chr3:41459758-41459780	41459775	3	RNA	GGCCGAGGTCGACTACCGGCNRG	caCCGAGGTtGAt--CCGGtGAG	+	5	2	5	0.00	2.50
Chr2:127323580-127323602	127323587	5	RNA	GGCCGAGGTCGACTACCGGCNRG	aGttGAGcTCGACTACt--CGAG	-	5	2	5	0.00	2.50
Chr8:58388506-58388528	58388513	5	RNA	GGCCGAGGTCGACTACCGGCNRG	GGtCGAG-TCtACTACactCAAG	-	5	1	5	0.00	2.50
Chr7:55023024-55023046	55023031	5	RNA	GGCCGAGGTCGACTACCGGCNRG	aGCCGAGGgtGcCT-CgGGCTGG	-	5	1	5	0.00	2.50
Chr3:120787484-120787506	120787501	3	RNA	GGCCGAGGTCGACTACCGGCNRG	GG--GAGGTCGgCTtCgtGtGAG	+	5	2	5	0.00	2.50
Chr5:45619841-45619863	45619858	3	RNA	GGCCGAGGTCGACTACCGGCNRG	G--CGAGGTgGACTgCgGGCAAG	+	3	2	5	0.00	2.50
Chr3:177913195-177913217	177913202	5	RNA	GGCCGAGGTCGACTACCGGCNRG	cGaCaAcGTCGACTACCa-CGAG	-	5	1	5	0.00	2.50
Chr4:133565679-133565701	133565686	5	RNA	GGCCGAGGTCGACTACCGGCNRG	GGgCtcGGTC--CTgCgGGCAGG	-	5	2	5	0.00	2.50
Chr9:154291337-154291359	154291344	5	RNA	GGCCGAGGTCGACTACCGGCNRG	aGCaGAG--CaACaACCGaCAAG	-	5	2	5	0.00	2.50
Chr10:65936308-65936330	65936315	5	RNA	GGCCGAGGTCGACTACCGGCNRG	GtCCG--GTgcACcACCGGaCAG	-	5	2	5	0.00	2.50
Chr2:231454238-231454260	231454255	3	RNA	GGCCGAGGTCGACTACCGGCNRG	GGCCaAcGaCaACgACC--CGAG	+	5	2	5	0.00	2.50
Chr7:120068997-120069019	120069014	3	RNA	GGCCGAGGTCGACTACCGGCNRG	GGCtaAGGTtGcCTtC-GGCAGG	+	5	1	5	0.00	2.50
Chr1:192787286-192787308	192787303	3	DNA	GGCCGAGGTCG-ACTACCGGCNRG	GGgCGAGGTCGGACTtCgGcgAGG	+	5	1	5	0.00	2.50
Chr5:103391091-103391113	103391098	5	RNA	GGCCGAGGTCGACTACCGGCNRG	GG--GAaGcCGAaatCCGGCGAG	-	5	2	5	0.00	2.50
Chr10:38036968-38036990	38036975	5	RNA	GGCCGAGGTCGACTACCGGCNRG	GaagGAGcTCGACTg--GGCGAG	-	5	2	5	0.00	2.50
Chr8:171311548-171311570	171311555	5	DNA	GGCC-GAGGTCGACTACCGGCNRG	cGCCTGAGGTCaACctCgGGCAGG	-	5	1	5	0.00	2.50
Chr1:97695165-97695187	97695182	3	RNA	GGCCGAGGTCGACTACCGGCNRG	GG--GAGaTgGACgcCCGGaGAG	+	5	2	5	0.00	2.50
Chr8:144061144-144061166	144061151	5	RNA	GGCCGAGGTCGACTACCGGCNRG	GGgCG-GGcCaACctCCGGCTGG	-	5	1	5	0.00	2.50
Chr5:223785019-223785041	223785026	5	RNA	GGCCGAGGTCGACTACCGGCNRG	GGCCGgGccaGACcA--GGCTGG	-	5	2	5	0.00	2.50
Chr1:217765429-217765451	217765436	5	DNA	GGCCGAGGTCGACT--ACCGGCNRG	GaCCtAGtcCGACTCAACCGGaTGG	-	5	2	5	0.00	2.50
Chr8:85459239-85459261	85459256	3	RNA	GGCCGAGGTCGACTACCGGCNRG	GGaCGAGGTtG--ggCCGGCCGG	+	4	2	5	0.00	2.50
Chr7:1512817-1512839	1512824	5	RNA	GGCCGAGGTCGACTACCGGCNRG	GGtCGAGGcCGAgT--CtGgGGG	-	5	2	5	0.00	2.50
Chr1:211205132-211205154	211205139	5	RNA	GGCCGAGGTCGACTACCGGCNRG	G-aCGgGGTCGACaACgaGCGGG	-	5	1	5	0.00	2.50
Chr6:159078741-159078763	159078758	3	RNA	GGCCGAGGTCGACTACCGGCNRG	GcCgGAGccCGAC--CCGtCAGG	+	5	2	5	0.00	2.50
Chr1:130411725-130411747	130411742	3	RNA	GGCCGAGGTCGACTACCGGCNRG	GGtCGtGGTCGAC--CgGaaCGG	+	5	2	5	0.00	2.50
Chr1:162784612-162784634	162784619	5	RNA	GGCCGAGGTCGACTACCGGCNRG	GGCCGAtcTCG--cAtCaGCAAG	-	5	2	5	0.00	2.50
Chr6:144539158-144539180	144539175	3	DNA	GGC--CGAGGTCGACTACCGGCNRG	GaCAACGgGGTCGACgACgaGCGGG	+	5	2	5	0.00	2.50
Chr2:65855349-65855371	65855356	5	DNA	GGCCGAGGTCGACTACCGG--CNRG	GGtCGAGGTCGAgTcCaaGCCCCGG	-	5	2	5	0.00	2.50
Chr10:86321315-86321337	86321322	5	RNA	GGCCGAGGTCGACTACCGGCNRG	aGCCGAGGgtGcCT-CgGGCTGG	-	5	1	5	0.00	2.50
Chr4:46369098-46369120	46369115	3	RNA	GGCCGAGGTCGACTACCGGCNRG	GaCCGAtGgCGAggAC--GCGGG	+	5	2	5	0.00	2.50
Chr7:66541582-66541604	66541599	3	RNA	GGCCGAGGTCGACTACCGGCNRG	GGCaGAGGgaGcCaA--GGCGAG	+	5	2	5	0.00	2.50
Chr10:136500128-136500150	136500135	5	RNA	GGCCGAGGTCGACTACCGGCNRG	GGgCGAcGT--ACTAtCGGgGGG	-	4	2	5	0.00	2.50
Chr10:97117459-97117481	97117476	3	RNA	GGCCGAGGTCGACTACCGGCNRG	GGgaGAGGgC--CTACCttCAGG	+	5	2	5	0.00	2.50
Chr10:77052172-77052194	77052179	5	RNA	GGCCGAGGTCGACTACCGGCNRG	GGC--AGGcCaACctCCGGCTGG	-	4	2	5	0.00	2.50
Chr9:60479550-60479572	60479567	3	RNA	GGCCGAGGTCGACTACCGGCNRG	tGCCGAG--aGgCTtCgGGCAGG	+	5	2	5	0.00	2.50
Chr10:135176533-135176555	135176540	5	DNA	GGCCGAGGTCGACTACCG--GCNRG	GGCCGgGGTCGgCccaCGGCGCGGG	-	5	2	5	0.00	2.50
Chr1:208246274-208246296	208246291	3	RNA	GGCCGAGGTCGACTACCGGCNRG	GtCaGAGacCGAt--CCGGCCAG	+	5	2	5	0.00	2.50
Chr6:33387550-33387572	33387567	3	RNA	GGCCGAGGTCGACTACCGGCNRG	cGCCGAGGTtG--TcCaGGgAAG	+	5	2	5	0.00	2.50
Chr3:211293273-211293295	211293280	5	RNA	GGCCGAGGTCGACTACCGGCNRG	aGC--AGGgCGgCatCCGGCAGG	-	5	2	5	0.00	2.50
Chr7:66242738-66242760	66242745	5	RNA	GGCCGAGGTCGACTACCGGCNRG	GGCCG--GTCcACctgCGGaAGG	-	5	2	5	0.00	2.50
Chr10:111236500-111236522	111236507	5	RNA	GGCCGAGGTCGACTACCGGCNRG	cGaCt--GTCGACgACCtGCAGG	-	5	2	5	0.00	2.50
Chr10:124294629-124294651	124294646	3	RNA	GGCCGAGGTCGACTACCGGCNRG	G-aCGgGGTCGACgACgaGCGGG	+	5	1	5	0.00	2.50
Chr4:123728515-123728537	123728532	3	DNA	GGCCGAGGTCGACTACCG--GCNRG	GcCCGAGGgCaACcACCGTAGCCAG	+	4	2	5	0.00	2.50
Chr1:292730640-292730662	292730647	5	DNA	GGCCGAGGTC--GACTACCGGCNRG	GGCgGAGGTCTGGACTtaCatCCGG	-	5	2	5	0.00	2.50
Chr4:180225805-180225827	180225822	3	RNA	GGCCGAGGTCGACTACCGGCNRG	G-tCGgGGTCGACaACaaGCGGG	+	5	1	5	0.00	2.50
Chr2:159572671-159572693	159572688	3	DNA	GGCCGAGGTCG--ACTACCGGCNRG	GGCacAGGgaGAAACTAtCGGCGAG	+	5	2	5	0.00	2.50
Chr10:98651813-98651835	98651820	5	DNA	GGCCGA-GGTCGACTACCGGCNRG	GGCgGACGGTCcgCgACCGGCCAG	-	4	1	5	0.00	2.50
Chr5:109297039-109297061	109297046	5	RNA	GGCCGAGGTCGACTACCGGCNRG	GGtCGAtcTCG--TAtCaGCAAG	-	5	2	5	0.00	2.50
Chr2:16319893-16319915	16319900	5	RNA	GGCCGAGGTCGACTACCGGCNRG	GtCCtAGG-CaACaACaGGCCGG	-	5	1	5	0.00	2.50
Chr3:223007823-223007845	223007830	5	RNA	GGCCGAGGTCGACTACCGGCNRG	GaCCGAGaaacACT-CCGGCGAG	-	5	1	5	0.00	2.50
Chr5:176728105-176728127	176728112	5	RNA	GGCCGAGGTCGACTACCGGCNRG	GGC-GAGGTgGAtggaCGGCGAG	-	5	1	5	0.00	2.50
Chr5:21270867-21270889	21270874	5	RNA	GGCCGAGGTCGACTACCGGCNRG	GcCCGAGGgCaACcACC--gTGG	-	5	2	5	0.00	2.50
Chr1:299750699-299750721	299750716	3	RNA	GGCCGAGGTCGACTACCGGCNRG	GtCCG--GTgcACcACCGGaCAG	+	5	2	5	0.00	2.50
Chr8:134959558-134959580	134959565	5	RNA	GGCCGAGGTCGACTACCGGCNRG	GGCCG-GGTCGtCcACtGGgGGG	-	4	1	5	0.00	2.50
Chr2:201473809-201473831	201473826	3	RNA	GGCCGAGGTCGACTACCGGCNRG	GGC--AGGcCGAgcACCaGCCGG	+	4	2	5	0.00	2.50
Chr8:110600319-110600341	110600326	5	RNA	GGCCGAGGTCGACTACCGGCNRG	GGC--AGGcCGAgcACCGGCCGG	-	3	2	5	0.00	2.50
Chr1:114523409-114523431	114523426	3	RNA	GGCCGAGGTCGACTACCGGCNRG	GaCaGAGG--GAtTcCaGGCTAG	+	5	2	5	0.00	2.50
Chr9:89546799-89546821	89546806	5	RNA	GGCCGAGGTCGACTACCGGCNRG	cGCaGAGaT-GgCcACCGGCGAG	-	5	1	5	0.00	2.50
Chr10:29992070-29992092	29992087	3	RNA	GGCCGAGGTCGACTACCGGCNRG	tGgCGAGcctG--TACCGGCCAG	+	5	2	5	0.00	2.50
Chr7:27340483-27340505	27340490	5	RNA	GGCCGAGGTCGACTACCGGCNRG	GaCCGAGcTgGtCT-CCGtCCAG	-	5	1	5	0.00	2.50
Chr2:151303439-151303461	151303456	3	DNA	GGCCGAGGTCGACTACC--GGCNRG	GGCgGAGGTCGtCggCaGAGGCCAG	+	5	2	5	0.00	2.50
Chr1:207676943-207676965	207676960	3	RNA	GGCCGAGGTCGACTACCGGCNRG	GGCaGAGGcaGcCgACC--CAGG	+	5	2	5	0.00	2.50
Chr4:130241005-130241027	130241012	5	RNA	GGCCGAGGTCGACTACCGGCNRG	G--CGAGGTCcACggCaaGCCGG	-	5	2	5	0.00	2.50
Chr5:216916984-216917006	216917001	3	RNA	GGCCGAGGTCGACTACCGGCNRG	GGaCtAGGTCG--TcCCaGtTGG	+	5	2	5	0.00	2.50
Chr8:52567473-52567495	52567480	5	RNA	GGCCGAGGTCGACTACCGGCNRG	GGCCGAtcTCG--cAtCaGCAAG	-	5	2	5	0.00	2.50
Chr7:57909967-57909989	57909984	3	RNA	GGCCGAGGTCGACTACCGGCNRG	G-CCGAGcaCGACgACttGCTGG	+	5	1	5	0.00	2.50
Chr5:193317877-193317899	193317884	5	DNA	GGC--CGAGGTCGACTACCGGCNRG	GGCCTCGgGGgCtACacCCGGCGGG	-	5	2	5	0.00	2.50
Chr4:194333577-194333599	194333584	5	RNA	GGCCGAGGTCGACTACCGGCNRG	GGCCGAGGgCGcgaAC--GCGGG	-	4	2	5	0.00	2.50
Chr4:214388654-214388676	214388671	3	RNA	GGCCGAGGTCGACTACCGGCNRG	GGC-GcGGTCGACggCgaGCAAG	+	5	1	5	0.00	2.50
Chr3:17115923-17115945	17115930	5	RNA	GGCCGAGGTCGACTACCGGCNRG	GGtCGAGGTtGACTgC--GgTGG	-	4	2	5	0.00	2.50
Chr4:95457064-95457086	95457081	3	RNA	GGCCGAGGTCGACTACCGGCNRG	GcCCatGGTCGAgTtC--GCCAG	+	5	2	5	0.00	2.50
Chr7:123821724-123821746	123821731	5	DNA	GGC--CGAGGTCGACTACCGGCNRG	GGCCTCGgGGgCtACacCCGGCGGG	-	5	2	5	0.00	2.50
Chr4:230157569-230157591	230157576	5	RNA	GGCCGAGGTCGACTACCGGCNRG	aGCCGAGGgCGcCT-CgGGCAGG	-	4	1	5	0.00	2.50
Chr5:146175700-146175722	146175707	5	DNA	GGCCGAGGTCGAC-TACCGGCNRG	GGCCGgcGgCGAgGaACCGGCCGG	-	5	1	5	0.00	2.50
Chr3:217269250-217269272	217269267	3	RNA	GGCCGAGGTCGACTACCGGCNRG	GGCCtAGcT--ACTACtGatTGG	+	5	2	5	0.00	2.50

Chr4:70087974-70087996	70087991	3	RNA	GGCCGAGGTCGACTACCGGCNRG	GcCCGAGGaCcACcACC--tTGG	+	5	2	5	0.00	2.50
Chr6:61773294-61773316	61773311	3	DNA	GGCCGAGGTCG-ACTACCGGCNRG	GGCCGAGaTCGGACggCCcaCGGG	+	5	1	5	0.00	2.50
Chr3:9295106-9295128	9295123	3	RNA	GGCCGAGGTCGACTACCGGCNRG	GGgCG-GGcCaACctCCGGCCGG	+	5	1	5	0.00	2.50
Chr7:101933327-101933349	101933334	5	RNA	GGCCGAGGTCGACTACCGGCNRG	GGCCcAG--aGAtTgCaGGCCGG	-	5	2	5	0.00	2.50
Chr3:129442814-129442836	129442821	5	RNA	GGCCGAGGTCGACTACCGGCNRG	GaCaG-GGTCGACaACgaGCGGG	-	5	1	5	0.00	2.50
Chr3:145021398-145021420	145021415	3	RNA	GGCCGAGGTCGACTACCGGCNRG	GGCtGgGGgCGA-TACtGGgTGG	+	5	1	5	0.00	2.50
Chr1:239838730-239838752	239838737	5	RNA	GGCCGAGGTCGACTACCGGCNRG	GGC--ttGcgGACTACCGGCTGG	-	4	2	5	0.00	2.50
Chr1:198971584-198971606	198971601	3	DNA	GGCCGAGGT--CGACTACCGGCNRG	GcCCGAcGTGGCGACTtCCaGCAAG	+	4	2	5	0.00	2.50
Chr3:97830725-97830747	97830732	5	DNA	GGCCGAGGTCGA--CTACCGGCNRG	GGCgGAGGTgGAGGCTgtgGGCAAG	-	5	2	5	0.00	2.50
Chr1:194591431-194591453	194591438	5	RNA	GGCCGAGGTCGACTACCGGCNRG	GGaCGAcccCGACT-CCGGtTAG	-	5	1	5	0.00	2.50
Chr10:81885111-81885133	81885128	3	RNA	GGCCGAGGTCGACTACCGGCNRG	GtCCG--GTgcACcACCGGaCAG	+	5	2	5	0.00	2.50
Chr2:161400844-161400866	161400861	3	RNA	GGCCGAGGTCGACTACCGGCNRG	GGCCGAGGgtGtCT-CgGGCTGG	+	4	1	5	0.00	2.50
Chr4:23415534-23415556	23415551	3	RNA	GGCCGAGGTCGACTACCGGCNRG	tGCCGAaGaCGAC-AgCaGCAGG	+	5	1	5	0.00	2.50
Chr3:156398865-156398887	156398872	5	RNA	GGCCGAGGTCGACTACCGGCNRG	GGC--ttGcgGACTACtGGCTGG	-	5	2	5	0.00	2.50
Chr2:44925552-44925574	44925569	3	RNA	GGCCGAGGTCGACTACCGGCNRG	G-tCGAGcaCGAgTACCcGCAGG	+	5	1	5	0.00	2.50
Chr2:199451763-199451785	199451780	3	RNA	GGCCGAGGTCGACTACCGGCNRG	GGCCGAGtTCGAg--CCctgGGG	+	5	2	5	0.00	2.50
Chr1:182580711-182580733	182580718	5	RNA	GGCCGAGGTCGACTACCGGCNRG	GGCCGAccTCGgCaA--GGaCAG	-	5	2	5	0.00	2.50
Chr4:206598736-206598758	206598743	5	RNA	GGCCGAGGTCGACTACCGGCNRG	GGaCaAGcT-GACgACgGGCAGG	-	5	1	5	0.00	2.50
Chr3:100225721-100225743	100225738	3	RNA	GGCCGAGGTCGACTACCGGCNRG	GaCCaAGGTCaACTgCt--CGGG	+	5	2	5	0.00	2.50
Chr2:59873070-59873092	59873087	3	RNA	GGCCGAGGTCGACTACCGGCNRG	GGC--tGGcaGACTACCGGCTGG	+	3	2	5	0.00	2.50
Chr1:137635972-137635994	137635989	3	RNA	GGCCGAGGTCGACTACCGGCNRG	GGCCGAtcTCG--cAtCaGCAAG	+	5	2	5	0.00	2.50
Chr2:34031210-34031232	34031227	3	RNA	GGCCGAGGTCGACTACCGGCNRG	G--CGAcGaCGACccCgGGCGAG	+	5	2	5	0.00	2.50
Chr1:218040835-218040857	218040852	3	RNA	GGCCGAGGTCGACTACCGGCNRG	GGCgGAGGTCG--TtCgGtaGAG	+	5	2	5	0.00	2.50
Chr1:118689618-118689640	118689625	5	RNA	GGCCGAGGTCGACTACCGGCNRG	tagCGAGGTgGgCTAC--GCGGG	-	5	2	5	0.00	2.50
Chr10:137336849-137336871	137336866	3	RNA	GGCCGAGGTCGACTACCGGCNRG	GGtCGAGGT--ACTtCaaGCCGG	+	4	2	5	0.00	2.50
Chr7:132378711-132378733	132378718	5	RNA	GGCCGAGGTCGACTACCGGCNRG	GGCCGAGGcCGAgccCC--gGGG	-	5	2	5	0.00	2.50
Chr8:93717635-93717657	93717642	5	RNA	GGCCGAGGTCGACTACCGGCNRG	GGCCGAG--CaAggAgCGcCAAG	-	5	2	5	0.00	2.50
Chr2:30605462-30605484	30605479	3	RNA	GGCCGAGGTCGACTACCGGCNRG	GGgaGAGGTCcACgAC--GgCGG	+	5	2	5	0.00	2.50
Chr4:211794527-211794549	211794534	5	X	GGCCGAGGTCGACTACCGGCNRG	GGCCGAGGcCGAgctCgGGCGAG	-	5	0	5	0.00	2.50
Chr9:52942755-52942777	52942762	5	RNA	GGCCGAGGTCGACTACCGGCNRG	G-aCGgGGTCGACgACgaGCGGG	-	5	1	5	0.00	2.50
Chr9:88453297-88453319	88453314	3	RNA	GGCCGAGGTCGACTACCGGCNRG	GG--GAGGatGACTAgCGtgAGG	+	5	2	5	0.00	2.50
Chr5:3147718-3147740	3147725	5	RNA	GGCCGAGGTCGACTACCGGCNRG	GGCCaAa--CGACaAtCtGCTGG	-	5	2	5	0.00	2.50
Chr4:239488684-239488706	239488701	3	RNA	GGCCGAGGTCGACTACCGGCNRG	GGCCGcGGcCGAtT--CGGCCGG	+	3	2	5	0.00	2.50
Chr2:72470288-72470310	72470305	3	RNA	GGCCGAGGTCGACTACCGGCNRG	GaCCGAa--CGACgACCGGCAAG	+	3	2	5	0.00	2.50
Chr1:262569085-262569107	262569102	3	RNA	GGCCGAGGTCGACTACCGGCNRG	GGCCGctGcCG-CcgCCGGCGAG	+	5	1	5	0.00	2.50
Chr10:67750513-67750535	67750520	5	RNA	GGCCGAGGTCGACTACCGGCNRG	GGtaGAGGagGAC-gCCGGCTGG	-	5	1	5	0.00	2.50
Chr2:53062851-53062873	53062858	5	DNA	GGCCGAGGTCGACTA--CCGGCNRG	ctCCaAGGTCGACcAATCCGGCAGG	-	4	2	5	0.00	2.50
Chr9:7013318-7013340	7013325	5	DNA	GGC-CGAGGTCGACTACCGGCNRG	GcCTCGgGGaCGtCTtCCGGCTGG	-	5	1	5	0.00	2.50
Chr9:82081647-82081669	82081654	5	DNA	GGCCGAGGT-CGACTACCGGCNRG	cGgCGAGGTACGAactCCGGCGAG	-	5	1	5	0.00	2.50
Chr5:151398762-151398784	151398779	3	RNA	GGCCGAGGTCGACTACCGGCNRG	GG--GAGaTgaACTtCCGGaGGG	+	5	2	5	0.00	2.50
Chr1:304742668-304742690	304742685	3	RNA	GGCCGAGGTCGACTACCGGCNRG	aGCCGgG--aGAaTACCtGCGGG	+	5	2	5	0.00	2.50
Chr2:17907161-17907183	17907178	3	DNA	GGCCGAGGTCGACTA--CCGGCNRG	ctCCtAGGcCGACcAATCCGGCAGG	+	5	2	5	0.00	2.50
Chr1:92062580-92062602	92062587	5	RNA	GGCCGAGGTCGACTACCGGCNRG	GtCttgGGT-GACcACCGGCGAG	-	5	1	5	0.00	2.50
Chr2:133229184-133229206	133229201	3	RNA	GGCCGAGGTCGACTACCGGCNRG	G--CGAGGgCtgCTACCGGCCGG	+	3	2	5	0.00	2.50
Chr4:44510781-44510803	44510798	3	RNA	GGCCGAGGTCGACTACCGGCNRG	cGCCGAGtTaGAgTA--GGCAGG	+	4	2	5	0.00	2.50
Chr8:114282077-114282099	114282084	5	DNA	GGCCGAGGTCG-ACTACCGGCNRG	tcCCGAGGTCGCACggCtGGCCAG	-	5	1	5	0.00	2.50
Chr1:282902439-282902461	282902456	3	RNA	GGCCGAGGTCGACTACCGGCNRG	GGCCaAGcgCG--gACCGtCCGG	+	5	2	5	0.00	2.50
Chr7:182231025-182231047	182231042	3	RNA	GGCCGAGGTCGACTACCGGCNRG	GGC--tcGcgGACTACCGGCTGG	+	4	2	5	0.00	2.50
Chr2:58693796-58693818	58693803	5	RNA	GGCCGAGGTCGACTACCGGCNRG	GG--GAGaTgaACTtCCGGaGGG	-	5	2	5	0.00	2.50
Chr8:25629545-25629567	25629562	3	RNA	GGCCGAGGTCGACTACCGGCNRG	G-aCGgGGTCGACaACgaGCGGG	+	5	1	5	0.00	2.50
Chr7:146414653-146414675	146414670	3	RNA	GGCCGAGGTCGACTACCGGCNRG	GGgaGAGaTCGACTtC--GgTGG	+	5	2	5	0.00	2.50
Chr3:138783219-138783241	138783236	3	RNA	GGCCGAGGTCGACTACCGGCNRG	GGC--tcGcgGACTACCGGtTGG	+	5	2	5	0.00	2.50
Chr3:128477406-128477428	128477413	5	RNA	GGCCGAGGTCGACTACCGGCNRG	tGC--AGGcCGACcAaCaGCTAG	-	5	2	5	0.00	2.50
Chr5:165674116-165674138	165674123	5	RNA	GGCCGAGGTCGACTACCGGCNRG	G-aCGgGGTCGACgACgaGCGGG	-	5	1	5	0.00	2.50
Chr4:221870198-221870220	221870215	3	RNA	GGCCGAGGTCGACTACCGGCNRG	GGCgGAGGTCGACgACa--aCGG	+	4	2	5	0.00	2.50
Chr1:151859796-151859818	151859813	3	RNA	GGCCGAGGTCGACTACCGGCNRG	GGgCcAcGTCGACT--CaaCCAG	+	5	2	5	0.00	2.50
Chr1:139517907-139517929	139517924	3	RNA	GGCCGAGGTCGACTACCGGCNRG	GG--GtGtTCGACaACaaGCAAG	+	5	2	5	0.00	2.50
Chr8:171457896-171457918	171457903	5	RNA	GGCCGAGGTCGACTACCGGCNRG	GGCgGAGGTCG--TtCgGcaGAG	-	5	2	5	0.00	2.50
Chr2:131249967-131249989	131249974	5	DNA	GGCCGAGGTCGACTA--CCGGCNRG	cGCaaAGGcCGACcAATCCGGCAGG	-	5	2	5	0.00	2.50
Chr5:77159066-77159088	77159083	3	RNA	GGCCGAGGTCGACTACCGGCNRG	G-aCGgGGTCGACaACgaGCGGG	+	5	1	5	0.00	2.50
Chr10:266540-266562	266557	3	DNA	GGC-CGAGGTCGACTACCGGCNRG	cGCTCGAGGTtGgCcACaGGCGAG	+	5	1	5	0.00	2.50
Chr7:43887934-43887956	43887941	5	RNA	GGCCGAGGTCGACTACCGGCNRG	G-aCGgGGTCGACaACgaGCGGG	-	5	1	5	0.00	2.50
Chr2:88096193-88096215	88096200	5	X	GGCCGAGGTCGACTACCGGCNRG	GGaaGAGGTgGcCTAgCGGCTGG	-	5	0	5	0.00	2.50
Chr6:19224183-19224205	19224190	5	RNA	GGCCGAGGTCGACTACCGGCNRG	GGC--ttGcgGACTACCGGCTGG	-	4	2	5	0.00	2.50
Chr3:15524289-15524311	15524296	5	RNA	GGCCGAGGTCGACTACCGGCNRG	GcCaGAGGcCGACTt--GGaGGG	-	5	2	5	0.00	2.50
Chr2:28078546-28078568	28078563	3	RNA	GGCCGAGGTCGACTACCGGCNRG	tGCgGtGGTCG--TAggGGCGGG	+	5	2	5	0.00	2.50
Chr7:79915664-79915686	79915671	5	RNA	GGCCGAGGTCGACTACCGGCNRG	GGCacAGG-CGACTgCtGtCCGG	-	5	1	5	0.00	2.50
Chr7:74928865-74928887	74928872	5	RNA	GGCCGAGGTCGACTACCGGCNRG	GcCgGAGcTCtACTgCCG-CCGG	-	5	1	5	0.00	2.50
Chr8:170611572-170611594	170611589	3	RNA	GGCCGAGGTCGACTACCGGCNRG	GGCCGAGGgtGtCT-CgGGCTGG	+	4	1	5	0.00	2.50
Chr7:163533997-163534019	163534014	3	RNA	GGCCGAGGTCGACTACCGGCNRG	GGCCcAG--CGACcgCgaGCAGG	+	5	2	5	0.00	2.50
Chr7:119842350-119842372	119842367	3	DNA	GGCCGAGGTCGACTACCG-GCNRG	GGgCGAGcTCGACcAtgGAGCAGG	+	5	1	5	0.00	2.50
Chr2:66148672-66148694	66148689	3	RNA	GGCCGAGGTCGACTACCGGCNRG	GGCCcA--TCGgtcACgGGCCAG	+	5	2	5	0.00	2.50
Chr2:22445536-22445558	22445543	5	RNA	GGCCGAGGTCGACTACCGGCNRG	G-CCtcGGgCGAgTACtGGCAGG	-	5	1	5	0.00	2.50
Chr4:53984293-53984315	53984310	3	RNA	GGCCGAGGTCGACTACCGGCNRG	GGC-GAGGTCGAgcgCCaGCGGG	+	4	1	5	0.00	2.50
Chr4:12529706-12529728	12529723	3	DNA	GGC--CGAGGTCGACTACCGGCNRG	GGCCTCGgGGgCtACacCCGGCGGG	+	5	2	5	0.00	2.50
Chr9:36447980-36448002	36447997	3	RNA	GGCCGAGGTCGACTACCGGCNRG	GGtCtAGGTCttC-ACCGGaTGG	+	5	1	5	0.00	2.50
Chr8:7139077-7139099	7139084	5	RNA	GGCCGAGGTCGACTACCGGCNRG	GtCCG--GTgGtgTACCGGaCAG	-	5	2	5	0.00	2.50
Chr6:45157195-45157217	45157212	3	RNA	GGCCGAGGTCGACTACCGGCNRG	GaCCaAaGTCaACTACt--CAGG	+	5	2	5	0.00	2.50
Chr7:106275519-106275541	106275526	5	RNA	GGCCGAGGTCGACTACCGGCNRG	cGCCaAGcTCGA-TACCtGgTGG	-	5	1	5	0.00	2.50
Chr5:136248185-136248207	136248192	5	RNA	GGCCGAGGTCGACTACCGGCNRG	GGCgctaaTCGACT-CCGGCGAG	-	5	1	5	0.00	2.50
Chr4:160494645-160494667	160494652	5	DNA	GGCCGAGGTCGACTA--CCGGCNRG	ctCCaAGGcCGACcAATCCGGCAGG	-	5	2	5	0.00	2.50
Chr4:156124844-156124866	156124851	5	RNA	GGCCGAGGTCGACTACCGGCNRG	GGatGAGcTCGAC--CCGagGAG	-	5	2	5	0.00	2.50
Chr10:36509790-36509812	36509797	5	RNA	GGCCGAGGTCGACTACCGGCNRG	GGC-GAGGcCGgCcACCaGgCGG	-	5	1	5	0.00	2.50
Chr4:107995573-107995595	107995580	5	RNA	GGCCGAGGTCGACTACCGGCNRG	GGC-GAGGTCGACgACgaGaTAG	-	4	1	5	0.00	2.50
Chr5:36540877-36540899	36540894	3	RNA	GGCCGAGGTCGACTACCGGCNRG	tG--GAGGagGACgACCtGCTGG	+	5	2	5	0.00	2.50
Chr6:47196181-47196203	47196198	3	RNA	GGCCGAGGTCGACTACCGGCNRG	GGCCaAttTCGgCTtC--GCCAG	+	5	2	5	0.00	2.50
Chr7:77796091-77796113	77796108	3	DNA	G-GCCGAGGTCGACTACCGGCNRG	GCGCCtcGGgCGAgTACtGGCAGG	+	5	1	5	0.00	2.50
Chr3:96386434-96386456	96386451	3	RNA	GGCCGAGGTCGACTACCGGCNRG	GGtCGAGGTCacC--CCGGgTAG	+	4	2	5	0.00	2.50
Chr4:67320914-67320936	67320921	5	RNA	GGCCGAGGTCGACTACCGGCNRG	GGCCGgGcTaGgCTg--GGCTAG	-	5	2	5	0.00	2.50
Chr1:109314742-109314764	109314749	5	RNA	GGCCGAGGTCGACTACCGGCNRG	GGCCGAGGatGcCT-CgGGCTGG	-	4	1	5	0.00	2.50
Chr8:54564954-54564976	54564971	3	DNA	GGCC-GAGGTCGACTACCGGCNRG	GaCaTGAGGTtGACgACCaGCGGG	+	5	1	5	0.00	2.50
Chr8:127697101-127697123	127697118	3	RNA	GGCCGAGGTCGACTACCGGCNRG	G-aCGgGGTCGACaACgaGCGGG	+	5	1	5	0.00	2.50
Chr5:192640023-192640045	192640030	5	RNA	GGCCGAGGTCGACTACCGGCNRG	aGC--tcGcgGACTACCGGCTGG	-	5	2	5	0.00	2.50
Chr4:93186487-93186509	93186494	5	RNA	GGCCGAGGTCGACTACCGGCNRG	aGCCGcGGTgGcCgA--GGCTAG	-	5	2	5	0.00	2.50
Chr8:65248729-65248751	65248746	3	RNA	GGCCGAGGTCGACTACCGGCNRG	GGaCGAGaTtG--TttCGGCCGG	+	5	2	5	0.00	2.50
Chr7:68428927-68428949	68428934	5	RNA	GGCCGAGGTCGACTACCGGCNRG	GG--GAGcTCGACgAgCtGgAGG	-	5	2	5	0.00	2.50
Chr2:118505663-118505685	118505680	3	RNA	GGCCGAGGTCGACTACCGGCNRG	GGggGAGGTgG--TAgaGGCTGG	+	5	2	5	0.00	2.50
Chr8:5110933-5110955	5110940	5	DNA	GGCCGAGGTCGACTA--CCGGCNRG	ctCCtAGGcCGACcAATCCGGCAGG	-	5	2	5	0.00	2.50
Chr10:48124588-48124610	48124605	3	DNA	GGC--CGAGGTCGACTACCGGCNRG	GGCCTCGgGGgCtACacCCGGCGGG	+	5	2	5	0.00	2.50
Chr5:158087904-158087926	158087911	5	RNA	GGCCGAGGTCGACTACCGGCNRG	GaCgagGGgCGACT-CCGGCGAG	-	5	1	5	0.00	2.50
Chr3:44342876-44342898	44342883	5	RNA	GGCCGAGGTCGACTACCGGCNRG	GGgCGccGT-GACTgCtGGCCGG	-	5	1	5	0.00	2.50
Chr3:31201635-31201657	31201652	3	RNA	GGCCGAGGTCGACTACCGGCNRG	tGCCcAGG--GACgAtaGGCAAG	+	5	2	5	0.00	2.50
Chr1:15490356-15490378	15490373	3	RNA	GGCCGAGGTCGACTACCGGCNRG	GatCG-GGTCGACgACgaGCGGG	+	5	1	5	0.00	2.50
Chr10:72899570-72899592	72899587	3	RNA	GGCCGAGGTCGACTACCGGCNRG	GcCCGAGaTCGACctCa--CCGG	+	5	2	5	0.00	2.50
Chr1:18004391-18004413	18004398	5	RNA	GGCCGAGGTCGACTACCGGCNRG	GG--GAtaTCGAgTACCaaCAGG	-	5	2	5	0.00	2.50
Chr7:153162134-153162156	153162141	5	DNA	GGCCGAGGT--CGACTACCGGCNRG	aGaCGAGGTTCCGACcAagGGCAGG	-	5	2	5	0.00	2.50
Chr5:19559432-19559454	19559438	4	RNA	GGCCGAGGTCGACTACCGGCNRG	GcCaGtGGaCGgC--CCGGCTAG	-	5	2	0	4.96	2.48
Chr2:98913861-98913883	98913878	3	DNA	GGCCGAG--GTCGACTACCGGCNRG	GGCCGAGCAGagGAactCCGGCCGG	+	5	2	0	4.96	2.48
Chr5:195931969-195931991	195931975	4	RNA	GGCCGAGGTCGACTACCGGCNRG	GGC--tcGTgGACTACCGcCTGG	-	4	2	0	4.96	2.48
Chr6:128822413-128822435	128822418	3	RNA	GGCCGAGGTCGACTACCGGCNRG	GaCaG-GGTCGACaACgaGCGGG	-	5	1	0	4.96	2.48
Chr1:124111388-124111410	124111404	4	X	GGCCGAGGTCGACTACCGGCNRG	GGaaGAGGTgGcCTAgCGGCTGG	+	5	0	0	4.96	2.48
Chr1:109940088-109940110	109940094	4	RNA	GGCCGAGGTCGACTACCGGCNRG	GGaCG--GTCGACacttGGCAGG	-	5	2	0	4.96	2.48
Chr6:164441173-164441195	164441188	5	RNA	GGCCGAGGTCGACTACCGGCNRG	GcCaGAGccCGAC--CCGtCAGG	+	5	2	0	4.96	2.48
Chr4:8755605-8755627	8755620	5	DNA	GGCCGAGGT--CGACTACCGGCNRG	GGgCGAGGcGGaGAtTgCCGGCTGG	+	5	2	0	4.96	2.48
Chr3:168788394-168788416	168788400	4	RNA	GGCCGAGGTCGACTACCGGCNRG	G-aCGgGGTCGACTACgaGCGGG	-	4	1	0	4.96	2.48
Chr4:199315961-199315983	199315976	5	RNA	GGCCGAGGTCGACTACCGGCNRG	GcaCGgcGTCGACTgC-GGCGAG	+	5	1	0	4.96	2.48
Chr9:133783471-133783493	133783475	2	RNA	GGCCGAGGTCGACTACCGGCNRG	tGCaGtGGTCGtCTgC--GCCAG	-	5	2	0	4.96	2.48
Chr5:194108057-194108079	194108073	4	RNA	GGCCGAGGTCGACTACCGGCNRG	cGCCG-GcTacACTAaCGGCGGG	+	5	1	0	4.96	2.48
Chr10:86070399-86070421	86070416	3	RNA	GGCCGAGGTCGACTACCGGCNRG	GGC--ttGTgGAaTACCGGCTGG	+	4	2	0	4.96	2.48
Chr4:187534235-187534257	187534251	4	RNA	GGCCGAGGTCGACTACCGGCNRG	t--CGgGGaCGtCTtCCGGCTGG	+	5	2	0	4.96	2.48
Chr2:37800422-37800444	37800427	3	RNA	GGCCGAGGTCGACTACCGGCNRG	G-aCGgGGTCGACaACgaGCGGG	-	5	1	0	4.96	2.48
Chr4:125456219-125456241	125456223	2	RNA	GGCCGAGGTCGACTACCGGCNRG	GtCgGAGGTaaACT-CCaGCCAG	-	5	1	0	4.96	2.48
Chr1:241548677-241548699	241548693	4	RNA	GGCCGAGGTCGACTACCGGCNRG	tGCaGAGcTCGgCTA--GGCTGG	+	4	2	0	4.96	2.48
Chr8:31049932-31049954	31049938	4	RNA	GGCCGAGGTCGACTACCGGCNRG	GcCCGAGGgCaACcACC--gTGG	-	5	2	0	4.96	2.48
Chr7:1323940-1323962	1323955	5	DNA	GGCCGAGGTCGACTACC--GGCNRG	GGCgGAGGTCGtCggCaGAGGCCAG	+	5	2	0	4.96	2.48
Chr5:92060275-92060297	92060293	2	RNA	GGCCGAGGTCGACTACCGGCNRG	GttCGAGcTCG--TACtGGaCAG	+	5	2	0	4.96	2.48

Chr9:104833430-104833452	104833436	4	RNA	GGCCGAGGTCGACTACCGGCNRG	aGCCG--GcCGACaACCaGaGAG	-	5	2	0	4.96	2.48
Chr10:78339100-78339122	78339104	2	RNA	GGCCGAGGTCGACTACCGGCNRG	GcCgcAGGTgG--TgCCGGCAAG	-	5	2	0	4.96	2.48
Chr3:67721785-67721807	67721790	3	RNA	GGCCGAGGTCGACTACCGGCNRG	GGCCGAcGTCGACcACt--CGAG	-	3	2	0	4.96	2.48
Chr1:179482810-179482832	179482827	3	DNA	GGCCGAGGTCGACTAC--CGGCNRG	GGaCGAGcTCGcCggCGGCGGCTGG	+	5	2	0	4.96	2.48
Chr5:155563529-155563551	155563547	2	RNA	GGCCGAGGTCGACTACCGGCNRG	GGaCcAGGTCG--TcCCaGtTGG	+	5	2	0	4.96	2.48
Chr2:109941218-109941240	109941224	4	RNA	GGCCGAGGTCGACTACCGGCNRG	cG--GAGGgaGAaTACCcGCTGG	-	5	2	0	4.96	2.48
Chr4:70664764-70664786	70664770	4	RNA	GGCCGAGGTCGACTACCGGCNRG	GGaCcAGGTCG--TcCCaGtTGG	-	5	2	0	4.96	2.48
Chr7:26041820-26041842	26041838	2	RNA	GGCCGAGGTCGACTACCGGCNRG	GGCgGAGGTCG--TtCgGcaGAG	+	5	2	0	4.96	2.48
Chr9:145402222-145402244	145402227	3	RNA	GGCCGAGGTCGACTACCGGCNRG	GcgCGtGGTaGcC--CCGGCGAG	-	5	2	0	4.96	2.48
Chr1:167309177-167309199	167309192	5	RNA	GGCCGAGGTCGACTACCGGCNRG	acCCGAtGTCG--TACgaGCTAG	+	5	2	0	4.96	2.48
Chr5:193726588-193726610	193726595	5	RNA	GGCCGAGGTCGACTACCGGCNRG	GGCCaAGGTCGg-TACgtGCAAG	-	4	1	0	4.96	2.48
Chr1:147651919-147651941	147651925	4	DNA	GGCCGAGGTCGACTACC--GGCNRG	GGCgGAGGTCGtCTgCaGAGGCCAG	-	4	2	0	4.96	2.48
Chr4:239022854-239022876	239022861	5	RNA	GGCCGAGGTCGACTACCGGCNRG	GcCgGAGccCGAC--CCGtCAAG	-	5	2	0	4.96	2.48
Chr4:206793171-206793193	206793186	5	RNA	GGCCGAGGTCGACTACCGGCNRG	GGCCc--GcCaACTtCCGGgCAG	+	5	2	0	4.96	2.48
Chr2:162983071-162983093	162983076	3	RNA	GGCCGAGGTCGACTACCGGCNRG	GGtCGAGGTC-ACcAtaGaCAGG	-	5	1	0	4.96	2.48
Chr8:96802599-96802621	96802615	4	RNA	GGCCGAGGTCGACTACCGGCNRG	GGaCGAG-TCGACgACgGGCGGG	+	3	1	0	4.96	2.48
Chr2:134103104-134103126	134103109	3	RNA	GGCCGAGGTCGACTACCGGCNRG	GGgCGAGGgCGAagAt-GGCGGG	-	5	1	0	4.96	2.48
Chr6:65982269-65982291	65982284	5	RNA	GGCCGAGGTCGACTACCGGCNRG	aGC--AGGTCGACgtaaGGCCGG	+	5	2	0	4.96	2.48
Chr6:108385885-108385907	108385903	2	RNA	GGCCGAGGTCGACTACCGGCNRG	GGCgGAG-TCcACcACCcaCTAG	+	5	1	0	4.96	2.48
Chr10:71156174-71156196	71156181	5	RNA	GGCCGAGGTCGACTACCGGCNRG	GGCCGAaaTCccCTcCC--CTGG	-	5	2	0	4.96	2.48
Chr4:107995249-107995271	107995254	3	RNA	GGCCGAGGTCGACTACCGGCNRG	GGC-GAGcTgGACTgCtGGCCAG	-	4	1	0	4.96	2.48
Chr2:49221166-49221188	49221183	3	DNA	GGC-CGAGGTCGACTACCGGCNRG	GcCTCGAGGaCGtCTtCCGGCTGG	+	4	1	0	4.96	2.48
Chr9:16620561-16620583	16620578	3	RNA	GGCCGAGGTCGACTACCGGCNRG	G--CtAGGTCGcggACCGtCCGG	+	5	2	0	4.96	2.48
Chr5:96941919-96941941	96941935	4	RNA	GGCCGAGGTCGACTACCGGCNRG	G-aCGgGGTCGACaACgaGCGGG	+	5	1	0	4.96	2.48
Chr6:145848412-145848434	145848418	4	RNA	GGCCGAGGTCGACTACCGGCNRG	GGCCGAGtcCG-CgACCaGaTAG	-	5	1	0	4.96	2.48
Chr7:133210223-133210245	133210241	2	RNA	GGCCGAGGTCGACTACCGGCNRG	cGgCGAGG--GAggtCCGGCGAG	+	5	2	0	4.96	2.48
Chr4:142823192-142823214	142823208	4	X	GGCCGAGGTCGACTACCGGCNRG	GGCgGAGcTCGAgcACgGGCGGG	+	5	0	0	4.96	2.48
Chr8:22412025-22412047	22412041	4	RNA	GGCCGAGGTCGACTACCGGCNRG	GGgCG-GGcCaACccCCGGCCGG	+	5	1	0	4.96	2.48
Chr4:208161430-208161452	208161436	4	RNA	GGCCGAGGTCGACTACCGGCNRG	GGagGAGGaCGACT--CatCAAG	-	5	2	0	4.96	2.48
Chr6:62578280-62578302	62578298	2	RNA	GGCCGAGGTCGACTACCGGCNRG	aGC--AGaTCGACTtaaGGCCGG	+	5	2	0	4.96	2.48
Chr3:83892362-83892384	83892377	5	RNA	GGCCGAGGTCGACTACCGGCNRG	GG--GAcaTCataTACCGGCTAG	+	5	2	0	4.96	2.48
Chr10:99222241-99222263	99222246	3	RNA	GGCCGAGGTCGACTACCGGCNRG	GGCatAGGat-ACTACaGGCAGG	-	5	1	0	4.96	2.48
Chr2:75002590-75002612	75002596	4	DNA	GGCCGAG-GTCGACTACCGGCNRG	aGCtGAGCGgCGgCaACCGGCGAG	-	5	1	0	4.96	2.48
Chr7:43788995-43789017	43789012	3	RNA	GGCCGAGGTCGACTACCGGCNRG	tGCCGcGcTCGgCT--CGGgCGG	+	5	2	0	4.96	2.48
Chr2:111869271-111869293	111869289	2	RNA	GGCCGAGGTCGACTACCGGCNRG	GGCCGccGT-GAgTACaGGtGAG	+	5	1	0	4.96	2.48
Chr2:216239561-216239583	216239567	4	RNA	GGCCGAGGTCGACTACCGGCNRG	GGCCG-GGcCGtCcACtGGCGGG	-	4	1	0	4.96	2.48
Chr1:118097494-118097516	118097510	4	RNA	GGCCGAGGTCGACTACCGGCNRG	GGtCGtGcTCGAC-ACgaGCTGG	+	5	1	0	4.96	2.48
Chr2:182693714-182693736	182693730	4	DNA	GGCCGAGGTCGACTACC--GGCNRG	aGCCGAcGgCGACTgCaAGGGCGGG	+	5	2	0	4.96	2.48
Chr4:18892791-18892813	18892796	3	RNA	GGCCGAGGTCGACTACCGGCNRG	G-CCGttaTCcACcACCGGCAGG	-	5	1	0	4.96	2.48
Chr8:14811737-14811759	14811753	4	RNA	GGCCGAGGTCGACTACCGGCNRG	G-CCGAaGTacACcACCtGCTGG	+	5	1	0	4.96	2.48
Chr8:34557825-34557847	34557842	3	RNA	GGCCGAGGTCGACTACCGGCNRG	cGCCGccGTC--CTcCCGtCAAG	+	5	2	0	4.96	2.48
Chr2:1399700-1399722	1399716	4	DNA	GGCCGAGGTCG--ACTACCGGCNRG	GcCCGAGGTCGCTcgTACCctCGGG	+	5	2	0	4.96	2.48
Chr7:134261506-134261528	134261522	4	RNA	GGCCGAGGTCGACTACCGGCNRG	GGCgGAGa-CatCTtCCGGCTGG	+	5	1	0	4.96	2.48
Chr10:18704782-18704804	18704789	5	RNA	GGCCGAGGTCGACTACCGGCNRG	GctCGAGGTgcACTAC-GGgTAG	-	5	1	0	4.96	2.48
Chr10:89301823-89301845	89301827	2	RNA	GGCCGAGGTCGACTACCGGCNRG	GGagGAGGagGACT--CGGaCGG	-	5	2	0	4.96	2.48
Chr8:42999715-42999737	42999721	4	RNA	GGCCGAGGTCGACTACCGGCNRG	GGaCGAGG-CGAgaAgtGGCTGG	-	5	1	0	4.96	2.48
Chr8:73523361-73523383	73523378	3	RNA	GGCCGAGGTCGACTACCGGCNRG	tGCCGtGGTCGcCg--CGGCGGG	+	4	2	0	4.96	2.48
Chr2:6666036-6666058	6666052	4	RNA	GGCCGAGGTCGACTACCGGCNRG	cGaCGAcGTCcACT--CGGgAAG	+	5	2	0	4.96	2.48
Chr9:117044286-117044308	117044291	3	RNA	GGCCGAGGTCGACTACCGGCNRG	GcCCGAGccCGAgT--CGGtGGG	-	5	2	0	4.96	2.48
Chr2:98435223-98435245	98435240	3	DNA	GGCCGAGGTCGACTACC--GGCNRG	GcCCGAGcaCaACcACCATGGCCAG	+	5	2	0	4.96	2.48
Chr2:103423915-103423937	103423933	2	RNA	GGCCGAGGTCGACTACCGGCNRG	GGCaGAaG--GACTcCgGGCCAG	+	4	2	0	4.96	2.48
Chr3:131973043-131973065	131973058	5	RNA	GGCCGAGGTCGACTACCGGCNRG	GGgaGAaGTgGACgACCG-CTGG	+	5	1	0	4.96	2.48
Chr6:152415481-152415503	152415499	2	RNA	GGCCGAGGTCGACTACCGGCNRG	GGatGAG--aGACaACtGGCGAG	+	5	2	0	4.96	2.48
Chr8:109840741-109840763	109840756	5	RNA	GGCCGAGGTCGACTACCGGCNRG	GGtCG-GGcCGtCcACtGGCGGG	+	5	1	0	4.96	2.48
Chr6:11350926-11350948	11350930	2	RNA	GGCCGAGGTCGACTACCGGCNRG	GGCCaAGGgCGcCT-CgGGCAGG	-	4	1	0	4.96	2.48
Chr4:65515622-65515644	65515637	5	RNA	GGCCGAGGTCGACTACCGGCNRG	GGC--ttGcgGACTACCGGCTGG	+	4	2	0	4.96	2.48
Chr1:153168105-153168127	153168121	4	RNA	GGCCGAGGTCGACTACCGGCNRG	GGgCG-GGcCaACccCCGGCCGG	+	5	1	0	4.96	2.48
Chr3:179124793-179124815	179124810	3	RNA	GGCCGAGGTCGACTACCGGCNRG	GGC--tcGcgGACTACCGGCTGG	+	4	2	0	4.96	2.48
Chr1:304148669-304148691	304148684	5	RNA	GGCCGAGGTCGACTACCGGCNRG	GtCttgGGT-GACcACCGGCGAG	+	5	1	0	4.96	2.48
Chr2:149089809-149089831	149089825	4	RNA	GGCCGAGGTCGACTACCGGCNRG	GGC--AGGcCaACctCCGGCCGG	+	4	2	0	4.96	2.48
Chr8:74798064-74798086	74798081	3	RNA	GGCCGAGGTCGACTACCGGCNRG	GGCtGgGGTaG--cACCGGgAGG	+	5	2	0	4.96	2.48
Chr8:70236156-70236178	70236172	4	RNA	GGCCGAGGTCGACTACCGGCNRG	a-CCGAGGTCGAgctCgGGCGAG	+	5	1	0	4.96	2.48
Chr2:85574295-85574317	85574310	5	RNA	GGCCGAGGTCGACTACCGGCNRG	G-aCGAGGTCGACgACgaGCGGG	+	4	1	0	4.96	2.48
Chr7:34662937-34662959	34662942	3	RNA	GGCCGAGGTCGACTACCGGCNRG	GGC--ttGcaGACTACCGGCTGG	-	4	2	0	4.96	2.48
Chr4:106462938-106462960	106462954	4	DNA	GGCCGAGGTCG-ACTACCGGCNRG	aGCCtAGGTCGCAgaACaGGCTAG	+	5	1	0	4.96	2.48
Chr1:53396316-53396338	53396322	4	RNA	GGCCGAGGTCGACTACCGGCNRG	GcCCGAGtcCGA-gAgCGGCTGG	-	5	1	0	4.96	2.48
Chr7:180147340-180147362	180147357	3	RNA	GGCCGAGGTCGACTACCGGCNRG	G-CCGAGGagGACgACCGGgTGG	+	4	1	0	4.96	2.48
Chr4:145616709-145616731	145616724	5	RNA	GGCCGAGGTCGACTACCGGCNRG	GGgCGAGGgCG--gACCtGgAGG	+	5	2	0	4.96	2.48
Chr5:116752231-116752253	116752236	3	RNA	GGCCGAGGTCGACTACCGGCNRG	GcCCGAGGgCaACcACC--gTGG	-	5	2	0	4.96	2.48
Chr2:63483043-63483065	63483049	4	RNA	GGCCGAGGTCGACTACCGGCNRG	GtgCtcGG--GACaACCGGCAGG	-	5	2	0	4.96	2.48
Chr9:143141983-143142005	143141988	3	RNA	GGCCGAGGTCGACTACCGGCNRG	GGCacAGGTtGAgg--CGGCCGG	-	5	2	0	4.96	2.48
Chr5:95635826-95635848	95635843	3	RNA	GGCCGAGGTCGACTACCGGCNRG	GGCCt-GGTCcACgtgCGGCTGG	+	5	1	0	4.96	2.48
Chr1:205664183-205664205	205664200	3	DNA	GGC-CGAGGTCGACTACCGGCNRG	GcCTCGAGGaCGtCTtCCGGCTGG	+	4	1	0	4.96	2.48
Chr3:16510324-16510346	16510342	2	RNA	GGCCGAGGTCGACTACCGGCNRG	GGC-GAGGTCctCctCCtGCCAG	+	5	1	0	4.96	2.48
Chr8:110049406-110049428	110049410	2	RNA	GGCCGAGGTCGACTACCGGCNRG	tGaaGAGaTCGACTACaG-CAGG	-	5	1	0	4.96	2.48
Chr7:124425503-124425525	124425510	5	RNA	GGCCGAGGTCGACTACCGGCNRG	tGCgGgGcTCGACTAC-GGaCGG	-	5	1	0	4.96	2.48
Chr6:45977536-45977558	45977541	3	RNA	GGCCGAGGTCGACTACCGGCNRG	aGCaGAG--CGAgatCCGGCCAG	-	5	2	0	4.96	2.48
Chr5:206529952-206529974	206529968	4	RNA	GGCCGAGGTCGACTACCGGCNRG	GcgCGtGGTaGcC--CCGGCGAG	+	5	2	0	4.96	2.48
Chr1:94666673-94666695	94666678	3	RNA	GGCCGAGGTCGACTACCGGCNRG	GGC--AGGcCGtgcACCGGCCGG	-	4	2	0	4.96	2.48
Chr6:127441781-127441803	127441796	5	RNA	GGCCGAGGTCGACTACCGGCNRG	GGCaGAGG--GACaACtGtCGGG	+	4	2	0	4.96	2.48
Chr2:164236196-164236218	164236211	5	RNA	GGCCGAGGTCGACTACCGGCNRG	GaCCG-GGTCGtCcACtGGCGGG	+	4	1	0	4.96	2.48
Chr1:149384055-149384077	149384062	5	RNA	GGCCGAGGTCGACTACCGGCNRG	GGgCGAGGgCG--gACCtGgAGG	-	5	2	0	4.96	2.48
Chr1:137801723-137801745	137801738	5	RNA	GGCCGAGGTCGACTACCGGCNRG	GtCCGgtGT--ACcACCGGaCAG	+	5	2	0	4.96	2.48
Chr8:151879708-151879730	151879726	2	RNA	GGCCGAGGTCGACTACCGGCNRG	GG--GAGGagGtaTACgGGCAAG	+	5	2	0	4.96	2.48
Chr4:15127855-15127877	15127871	4	RNA	GGCCGAGGTCGACTACCGGCNRG	tG--GAGGagGACgACCtGCTGG	+	5	2	0	4.96	2.48
Chr1:263810281-263810303	263810299	2	RNA	GGCCGAGGTCGACTACCGGCNRG	GaCCGAGG-CGACgACaaGaAGG	+	5	1	0	4.96	2.48
Chr9:21156229-21156251	21156245	4	RNA	GGCCGAGGTCGACTACCGGCNRG	G-aCGgGGTCGACaACgaGCGGG	+	5	1	0	4.96	2.48
Chr7:31825811-31825833	31825818	5	RNA	GGCCGAGGTCGACTACCGGCNRG	GGatGgGGTCGgC-gCCGGCGGG	-	5	1	0	4.96	2.48
Chr7:165795458-165795480	165795476	2	RNA	GGCCGAGGTCGACTACCGGCNRG	GGCgGtGG--GgCTgtCGGCGAG	+	5	2	0	4.96	2.48
Chr5:33545793-33545815	33545808	5	RNA	GGCCGAGGTCGACTACCGGCNRG	tGgCt-cGTgGACTACCGGCTGG	+	5	1	0	4.96	2.48
Chr9:74121535-74121557	74121539	2	RNA	GGCCGAGGTCGACTACCGGCNRG	GGCaGtGGT--ACTcCgGtCGAG	-	5	2	0	4.96	2.48
Chr2:159436251-159436273	159436258	5	RNA	GGCCGAGGTCGACTACCGGCNRG	GGCCcAc--CGACcACCGtgGAG	-	5	2	0	4.96	2.48
Chr4:165980880-165980902	165980896	4	RNA	GGCCGAGGTCGACTACCGGCNRG	G-aCGgGGTCGACgACgaGCGGG	+	5	1	0	4.96	2.48
Chr3:214260258-214260280	214260273	5	RNA	GGCCGAGGTCGACTACCGGCNRG	GGC--ttGcgGACTACCGGCTGG	+	4	2	0	4.96	2.48
Chr2:121783603-121783625	121783608	3	RNA	GGCCGAGGTCGACTACCGGCNRG	GaC--AGGcCGAgctCCGGCGAG	-	5	2	0	4.96	2.48
Chr3:214892996-214893018	214893002	4	RNA	GGCCGAGGTCGACTACCGGCNRG	G-CCtcGGgCGAgTACtGGCAGG	-	5	1	2	2.75	2.38
Chr5:223273918-223273940	223273923	3	RNA	GGCCGAGGTCGACTACCGGCNRG	GGC-GAGGTCGACcACaGGCAGG	-	2	1	2	2.75	2.38
Chr5:176274493-176274515	176274500	5	RNA	GGCCGAGGTCGACTACCGGCNRG	GGCCGAGGgtGcCT-CgGGaTGG	-	5	1	2	2.75	2.38
Chr5:173848653-173848675	173848669	4	RNA	GGCCGAGGTCGACTACCGGCNRG	G-CCGAaGTCcACTgCCtGCTGG	+	4	1	3	1.65	2.33
Chr2:179678271-179678293	179678288	3	X	GGCCGAGGTCGACTACCGGCNRG	GGCacAGGcCGAgcACCGGCCGG	+	5	0	3	1.65	2.33
Chr4:121416807-121416829	121416812	3	DNA	GGCCGAGGTCGACTACC--GGCNRG	GaCaGAGGaCGACgtCCACGGCTGG	-	5	2	0	4.41	2.21
Chr4:156821274-156821296	156821290	4	RNA	GGCCGAGGTCGACTACCGGCNRG	G-aCGgGGTCGACgACgaGCGGG	+	5	1	0	4.41	2.21
Chr5:160510373-160510395	160510378	3	RNA	GGCCGAGGTCGACTACCGGCNRG	G-CCGAaGTacACcACCtGCTGG	-	5	1	0	4.41	2.21
Chr2:120563297-120563319	120563315	2	RNA	GGCCGAGGTCGACTACCGGCNRG	GGCCGAG--CGAtgcCCaGCCAG	+	4	2	0	4.41	2.21
Chr1:178319218-178319240	178319236	2	RNA	GGCCGAGGTCGACTACCGGCNRG	a--CGgGGTCGACgACgaGCGGG	+	5	2	0	4.41	2.21
Chr7:72811777-72811799	72811793	4	RNA	GGCCGAGGTCGACTACCGGCNRG	G-aCGgGGTCGACaACgaGCGGG	+	5	1	0	4.41	2.21
Chr1:151987562-151987584	151987568	4	DNA	GGC--CGAGGTCGACTACCGGCNRG	GGCAAtGAaGTCtgCTAaCGGCTAG	-	5	2	0	4.41	2.21
Chr2:109649192-109649214	109649210	2	RNA	GGCCGAGGTCGACTACCGGCNRG	GatCaAcGTCGACaA--GGCAAG	+	5	2	0	4.41	2.21
Chr9:90809908-90809930	90809913	3	DNA	GGCCGAGGTCGACT--ACCGGCNRG	GGCtGtGtTtaACTCAACCGGCTAG	-	5	2	0	4.41	2.21
Chr2:11791465-11791487	11791483	2	RNA	GGCCGAGGTCGACTACCGGCNRG	GttCGAGaT--ACTtCgGGCAAG	+	5	2	0	4.41	2.21
Chr5:66670790-66670812	66670808	2	RNA	GGCCGAGGTCGACTACCGGCNRG	GGC--tcGcgGACTACCGaCTGG	+	5	2	0	4.41	2.21
Chr2:134228263-134228285	134228270	5	RNA	GGCCGAGGTCGACTACCGGCNRG	GGgCtAtG-CGACTAtCGGaTGG	-	5	1	0	4.41	2.21
Chr4:84440416-84440438	84440433	3	RNA	GGCCGAGGTCGACTACCGGCNRG	GGCCtAGG--GAgaAaCGaCTAG	+	5	2	0	4.41	2.21
Chr8:9283741-9283763	9283757	4	RNA	GGCCGAGGTCGACTACCGGCNRG	GGaCagccTCGAC-ACCGGCGAG	+	5	1	0	4.41	2.21
Chr3:119452257-119452279	119452274	3	X	GGCCGAGGTCGACTACCGGCNRG	GGCCGAGGTtGAgctCgGGCGAG	+	5	0	0	4.41	2.21
Chr5:125789930-125789952	125789945	5	RNA	GGCCGAGGTCGACTACCGGCNRG	GGC--AGcTCGAggACgaGCTGG	+	5	2	0	4.41	2.21
Chr3:213544370-213544392	213544377	5	RNA	GGCCGAGGTCGACTACCGGCNRG	G-CCtcGGgCGAgTACtGGCAGG	-	5	1	0	4.41	2.21
Chr8:106891427-106891449	106891434	5	RNA	GGCCGAGGTCGACTACCGGCNRG	GGgCG-GGcCaACctCCGGCCGG	-	5	1	0	4.41	2.21
Chr3:57148199-57148221	57148203	2	RNA	GGCCGAGGTCGACTACCGGCNRG	GGC--tcGcgGACTACCGGCTAG	-	4	2	0	4.41	2.21
Chr3:145244821-145244843	145244839	2	RNA	GGCCGAGGTCGACTACCGGCNRG	GGCCGgGGTCGg-TcgaGGCGAG	+	5	1	0	4.41	2.21
Chr3:231991398-231991420	231991413	5	RNA	GGCCGAGGTCGACTACCGGCNRG	GGC--tGGTCcAtTACatGCTGG	+	5	2	0	4.41	2.21
Chr4:116217396-116217418	116217403	5	RNA	GGCCGAGGTCGACTACCGGCNRG	GGC-GtGGTCGAggACtaGCGGG	-	5	1	0	4.41	2.21
Chr3:64476367-64476389	64476383	4	RNA	GGCCGAGGTCGACTACCGGCNRG	GGaCGAGccCGACT-CtGGtTGG	+	5	1	0	4.41	2.21
Chr2:92306997-92307019	92307015	2	RNA	GGCCGAGGTCGACTACCGGCNRG	G-aCGAaGaCGACTcCCGGaGAG	+	5	1	0	4.41	2.21
Chr3:132432660-132432682	132432665	3	RNA	GGCCGAGGTCGACTACCGGCNRG	cGCCGtGGTCGcCg--CGGCAGG	-	4	2	0	4.41	2.21
Chr4:41834984-41835006	41834990	4	DNA	GGC-CGAGGTCGACTACCGGCNRG	GcCACGgGGaCGtCTtCCGGCTGG	-	5	1	0	4.41	2.21

Chr1:26437689-26437711	26437695	4	RNA	GGCCGAGGTCGACTACCGGCNRG	GtCgaAGGgCG--TACCGGaCAG	-	5	2	0	4.41	2.21
Chr6:169692089-169692111	169692104	5	RNA	GGCCGAGGTCGACTACCGGCNRG	GGCaG-GcTacACTACCaGCTAG	+	5	1	0	4.41	2.21
Chr8:20913523-20913545	20913540	3	DNA	GGCCGAGGTCGACTACCGG-CNRG	GctgGAGGTCGACTtCCtGCCCGG	+	5	1	0	4.41	2.21
Chr10:23768318-23768340	23768324	4	RNA	GGCCGAGGTCGACTACCGGCNRG	GGCCGAcGaCa--TACCtGgAGG	-	5	2	0	4.41	2.21
Chr2:90292371-90292393	90292376	3	RNA	GGCCGAGGTCGACTACCGGCNRG	GGCCG-GGTCGtCcACtGGCGGG	-	3	1	0	4.41	2.21
Chr6:71306205-71306227	71306221	4	DNA	GGCCGAGGTCGA--CTACCGGCNRG	GcgCcAtcTCGATTCTACCGGCGAG	+	5	2	0	4.41	2.21
Chr2:16285307-16285329	16285325	2	RNA	GGCCGAGGTCGACTACCGGCNRG	GcCCGAGccCGAgT--CGGtAGG	+	5	2	0	4.41	2.21
Chr5:36749334-36749356	36749338	2	RNA	GGCCGAGGTCGACTACCGGCNRG	GaCgGgGGTCGtCTAC-GGgTAG	-	5	1	0	4.41	2.21
Chr2:232550878-232550900	232550885	5	RNA	GGCCGAGGTCGACTACCGGCNRG	caCCaAGtTCGcCT-CCGGCCAG	-	5	1	0	4.41	2.21
Chr3:104176262-104176284	104176269	5	RNA	GGCCGAGGTCGACTACCGGCNRG	GGCCGAGGcCtcCT-CgGGCTGG	-	4	1	0	4.41	2.21
Chr2:210153486-210153508	210153493	5	RNA	GGCCGAGGTCGACTACCGGCNRG	GGCaaActTCGgCTAC--GCGGG	-	5	2	0	4.41	2.21
Chr7:93462714-93462736	93462729	5	RNA	GGCCGAGGTCGACTACCGGCNRG	GGC-GAaGTCGAagACCtcCCAG	+	5	1	0	4.41	2.21
Chr2:188670649-188670671	188670667	2	RNA	GGCCGAGGTCGACTACCGGCNRG	GtCCG--GTgcACcACCGGaCAG	+	5	2	0	4.41	2.21
Chr5:119886116-119886138	119886134	2	DNA	GGC-CGAGGTCGACTACCGGCNRG	GcCTCGgGGaCGtCTtCCGGCTGG	+	5	1	0	4.41	2.21
Chr7:65064182-65064204	65064200	2	RNA	GGCCGAGGTCGACTACCGGCNRG	cGCCGtGcTC--CgACCGGtGGG	+	5	2	0	4.41	2.21
Chr7:84651792-84651814	84651810	2	RNA	GGCCGAGGTCGACTACCGGCNRG	GGCCGtctTCGAgTA--GaCCGG	+	5	2	0	4.41	2.21
Chr7:101820957-101820979	101820963	4	DNA	GGC-CGAGGTCGACTACCGGCNRG	GcCTCGgGGaCGtCTtCCGGCTGG	-	5	1	0	4.41	2.21
Chr7:80303198-80303220	80303202	2	RNA	GGCCGAGGTCGACTACCGGCNRG	tGaCGAGGTCGAC-AaCGagCGG	-	5	1	0	4.41	2.21
Chr4:158415050-158415072	158415057	5	RNA	GGCCGAGGTCGACTACCGGCNRG	GaC--AGGTgGgCcACCGGaCAG	-	5	2	0	4.41	2.21
Chr10:26799035-26799057	26799042	5	RNA	GGCCGAGGTCGACTACCGGCNRG	cGCCGtGGataACT-CCGGCCAG	-	5	1	0	4.41	2.21
Chr4:222763126-222763148	222763131	3	RNA	GGCCGAGGTCGACTACCGGCNRG	G-aCGgGGTCGACgACgaGCGGG	-	5	1	0	4.41	2.21
Chr1:3169837-3169859	3169855	2	RNA	GGCCGAGGTCGACTACCGGCNRG	GGa--AGGcCtACTACCaGCAAG	+	4	2	0	4.41	2.21
Chr7:5224528-5224550	5224533	3	X	GGCCGAGGTCGACTACCGGCNRG	GaCCGtGGTCGACcgtCGGCTGG	-	5	0	0	4.41	2.21
Chr8:13338032-13338054	13338037	3	RNA	GGCCGAGGTCGACTACCGGCNRG	cGtCGAaGTCGACgACCt-CGAG	-	5	1	0	4.41	2.21
Chr2:154847211-154847233	154847228	3	RNA	GGCCGAGGTCGACTACCGGCNRG	GGCCaAGG--GACacCCacCTGG	+	5	2	0	4.41	2.21
Chr5:117804758-117804780	117804762	2	DNA	GGCCGAGG--TCGACTACCGGCNRG	GGCCGAGGCCTCGACgcgCGcgGAG	-	5	2	0	4.41	2.21
Chr10:24024196-24024218	24024200	2	RNA	GGCCGAGGTCGACTACCGGCNRG	G-aCGgGGTCGACgACgtGCGGG	-	5	1	0	4.41	2.21
Chr8:45808111-45808133	45808127	4	RNA	GGCCGAGGTCGACTACCGGCNRG	GGCgGAaGTCG--TtCgGcCGAG	+	5	2	0	4.41	2.21
Chr6:160577612-160577634	160577630	2	RNA	GGCCGAGGTCGACTACCGGCNRG	aGC--tGtTCGtCaACCGGCAAG	+	5	2	0	4.41	2.21
Chr1:28144461-28144483	28144477	4	RNA	GGCCGAGGTCGACTACCGGCNRG	GGgCGAGtTCG--cACCtGCAGG	+	4	2	0	4.41	2.21
Chr4:230159861-230159883	230159866	3	RNA	GGCCGAGGTCGACTACCGGCNRG	GGCC-AGGTCGtCcACtGGtGGG	-	4	1	0	4.41	2.21
Chr4:15656113-15656135	15656129	4	RNA	GGCCGAGGTCGACTACCGGCNRG	GGC--AGGcCGAgcACtaGCCGG	+	5	2	0	4.41	2.21
Chr4:156382348-156382370	156382353	3	DNA	GGCCGA-GGTCGACTACCGGCNRG	GGCgGACGGTCcgCgACCGaCCAG	-	5	1	0	4.41	2.21
Chr7:57927816-57927838	57927834	2	RNA	GGCCGAGGTCGACTACCGGCNRG	GGCgGAGGTgGACgAaa--CCAG	+	5	2	0	4.41	2.21
Chr1:245947671-245947693	245947689	2	RNA	GGCCGAGGTCGACTACCGGCNRG	t--CGgGGaCGtCTtCCGGCTGG	+	5	2	0	4.41	2.21
Chr4:191564214-191564236	191564220	4	RNA	GGCCGAGGTCGACTACCGGCNRG	GGgCGAG--CGAtgAggGGCAAG	-	5	2	0	4.41	2.21
Chr5:141834972-141834994	141834977	3	RNA	GGCCGAGGTCGACTACCGGCNRG	G--tGAGGTCGACaACgGctAAG	-	5	2	0	4.41	2.21
Chr3:22899129-22899151	22899145	4	RNA	GGCCGAGGTCGACTACCGGCNRG	GtCCG--GTgcACcACCGGaCAG	+	5	2	0	4.41	2.21
Chr5:53466171-53466193	53466188	3	RNA	GGCCGAGGTCGACTACCGGCNRG	GGtCGAGcTCatCTAgC--CTGG	+	5	2	0	4.41	2.21
Chr8:15070972-15070994	15070989	3	RNA	GGCCGAGGTCGACTACCGGCNRG	GGtCGAGGcCGAgT--CcGgGGG	+	5	2	0	4.41	2.21
Chr4:214932465-214932487	214932482	3	RNA	GGCCGAGGTCGACTACCGGCNRG	GGtgGtGGTCGACgAC--GaAGG	+	5	2	0	4.41	2.21
Chr1:107398153-107398175	107398158	3	RNA	GGCCGAGGTCGACTACCGGCNRG	GGCCGAGGTC--CTtCttttCGG	-	5	2	0	4.41	2.21
Chr4:18490741-18490763	18490745	2	RNA	GGCCGAGGTCGACTACCGGCNRG	GGaCGAcGaCGACgACa--CTGG	-	5	2	0	4.41	2.21
Chr4:181163887-181163909	181163892	3	RNA	GGCCGAGGTCGACTACCGGCNRG	aGC--tcGcgGACTACCGGCTGG	-	5	2	0	4.41	2.21
Chr1:69266492-69266514	69266508	4	RNA	GGCCGAGGTCGACTACCGGCNRG	G-aCGgGGTCGACaACgaGCGGG	+	5	1	0	4.41	2.21
Chr4:142111391-142111413	142111406	5	RNA	GGCCGAGGTCGACTACCGGCNRG	acCCGAtGTCG--TACgaGCTAG	+	5	2	0	4.41	2.21
Chr3:76297619-76297641	76297623	2	RNA	GGCCGAGGTCGACTACCGGCNRG	GGaCGAGcTCGACTAa-GGgTGG	-	4	1	0	4.41	2.21
Chr1:60782069-60782091	60782074	3	DNA	GGC-CGAGGTCGACTACCGGCNRG	GGCTCcAtGTCGACTACCaGCAGG	-	3	1	0	4.41	2.21
Chr5:69002134-69002156	69002151	3	RNA	GGCCGAGGTCGACTACCGGCNRG	GGtCGAGGT--AacttCGGCGAG	+	5	2	0	4.41	2.21
Chr2:21783874-21783896	21783881	5	RNA	GGCCGAGGTCGACTACCGGCNRG	GaaCGAGaTCGcCT-CCGGgGAG	-	5	1	0	4.41	2.21
Chr4:979278-979300	979283	3	RNA	GGCCGAGGTCGACTACCGGCNRG	GGaCaAcGaCGACgAC--GCCGG	-	5	2	0	4.41	2.21
Chr2:133539387-133539409	133539402	5	RNA	GGCCGAGGTCGACTACCGGCNRG	caCCaAGtTCGcCT-CCGGCCAG	+	5	1	0	4.41	2.21
Chr2:77850122-77850144	77850137	5	RNA	GGCCGAGGTCGACTACCGGCNRG	GGC--AGGcaaACctCCGGCCGG	+	5	2	0	4.41	2.21
Chr5:108160504-108160526	108160522	2	RNA	GGCCGAGGTCGACTACCGGCNRG	G--CGAGGgtGACgcCCaGCAAG	+	5	2	0	4.41	2.21
Chr1:199867954-199867976	199867961	5	RNA	GGCCGAGGTCGACTACCGGCNRG	GGC-GtGGcCGAactCCGGCGAG	-	5	1	0	4.41	2.21
Chr2:178852051-178852073	178852058	5	RNA	GGCCGAGGTCGACTACCGGCNRG	cGgCGgGGcgG--TACCGGCGAG	-	5	2	0	4.41	2.21
Chr4:183543935-183543957	183543950	5	RNA	GGCCGAGGTCGACTACCGGCNRG	GGCCGAGGTCG--ggCgaGgCGG	+	5	2	0	4.41	2.21
Chr2:81903156-81903178	81903173	3	RNA	GGCCGAGGTCGACTACCGGCNRG	GGCgGAGGTCG--TtCgGcaGAG	+	5	2	0	4.41	2.21
Chr9:89455202-89455224	89455207	3	RNA	GGCCGAGGTCGACTACCGGCNRG	GGggGgaGTaG--TACCGGCAGG	-	5	2	0	4.41	2.21
Chr8:121283895-121283917	121283900	3	RNA	GGCCGAGGTCGACTACCGGCNRG	G--tGAGGTaatCaACCGGCAGG	-	5	2	0	4.41	2.21
Chr4:82989186-82989208	82989204	2	RNA	GGCCGAGGTCGACTACCGGCNRG	GGaCcAGGTCG--TcCCaGtTGG	+	5	2	0	4.41	2.21
Chr1:4753025-4753047	4753030	3	RNA	GGCCGAGGTCGACTACCGGCNRG	GaCaG-GGTCGACgACaaGCGGG	-	5	1	0	4.41	2.21
Chr1:127154321-127154343	127154337	4	RNA	GGCCGAGGTCGACTACCGGCNRG	GcCCGAGGaCaACcACC--aGAG	+	5	2	0	4.41	2.21
Chr8:140452244-140452266	140452259	5	RNA	GGCCGAGGTCGACTACCGGCNRG	a--CGgGGTCGACgACgaGCGGG	+	5	2	0	4.41	2.21
Chr4:217051907-217051929	217051914	5	RNA	GGCCGAGGTCGACTACCGGCNRG	GGgCGAGcTCGggTA--GGgAGG	-	5	2	0	4.41	2.21
Chr9:100658636-100658658	100658652	4	RNA	GGCCGAGGTCGACTACCGGCNRG	cGCCGAtcTCGA-TAgtGGCAAG	+	5	1	0	4.41	2.21
Chr2:85713475-85713497	85713491	4	RNA	GGCCGAGGTCGACTACCGGCNRG	tG--GAGGagGACgACCtGCTGG	+	5	2	0	4.41	2.21
Chr8:60988167-60988189	60988173	4	RNA	GGCCGAGGTCGACTACCGGCNRG	t--CGgGGaCGtCTtCCGGCTGG	-	5	2	0	4.41	2.21
Chr5:181078453-181078475	181078457	2	RNA	GGCCGAGGTCGACTACCGGCNRG	GGCgGAGGTCG--TtCgGcaGAG	-	5	2	0	4.41	2.21
Chr5:45229043-45229065	45229060	3	RNA	GGCCGAGGTCGACTACCGGCNRG	cGgCGAGcT--AaTACtGGCCAG	+	5	2	0	4.41	2.21
Chr3:229193108-229193130	229193113	3	RNA	GGCCGAGGTCGACTACCGGCNRG	GGC--tcGcgGACTACCGGCTGG	-	4	2	0	4.41	2.21
Chr4:6514333-6514355	6514340	5	RNA	GGCCGAGGTCGACTACCGGCNRG	caCCaAGtTCGcCT-CCGGCCAG	-	5	1	0	4.41	2.21
Chr9:99959380-99959402	99959397	3	RNA	GGCCGAGGTCGACTACCGGCNRG	GGaCcAGGTCG--TcCCaGtTGG	+	5	2	0	4.41	2.21
Chr2:234019439-234019461	234019457	2	RNA	GGCCGAGGTCGACTACCGGCNRG	cGCgcAGGTCGAC--CgGGaCAG	+	5	2	0	4.41	2.21
Chr10:147367339-147367361	147367354	5	RNA	GGCCGAGGTCGACTACCGGCNRG	G--CGAGtaCaACgACaGGCCGG	+	5	2	0	4.41	2.21
Chr5:107568350-107568372	107568368	2	RNA	GGCCGAGGTCGACTACCGGCNRG	GGCCGAtcTCG--cAtCaGCAAG	+	5	2	0	4.41	2.21
Chr1:236302922-236302944	236302938	4	RNA	GGCCGAGGTCGACTACCGGCNRG	G-aCGgGGTCGACaACgaGCGGG	+	5	1	0	4.41	2.21
Chr5:138518702-138518724	138518717	5	RNA	GGCCGAGGTCGACTACCGGCNRG	GGgtGgGGgtGA--ACCGGCCGG	+	5	2	0	4.41	2.21
Chr9:58193135-58193157	58193153	2	DNA	GGCCGAGGT--CGACTACCGGCNRG	GGCCaAGGTATCGAgaAgCaGCAAG	+	5	2	0	4.41	2.21
Chr1:33043023-33043045	33043040	3	RNA	GGCCGAGGTCGACTACCGGCNRG	tGaCGAGaTCaACgA--GGCTAG	+	5	2	0	4.41	2.21
Chr8:100063800-100063822	100063815	5	RNA	GGCCGAGGTCGACTACCGGCNRG	caCCaAGtTCGcCT-CCGGCCAG	+	5	1	0	4.41	2.21
Chr7:66275684-66275706	66275689	3	RNA	GGCCGAGGTCGACTACCGGCNRG	GcCCatccTCGACT-CCGGCAAG	-	5	1	0	4.41	2.21
Chr2:33024235-33024257	33024241	4	RNA	GGCCGAGGTCGACTACCGGCNRG	G-aCGgGGTCGACgACgaGCGGG	-	5	1	0	4.41	2.21
Chr5:50647281-50647303	50647299	2	RNA	GGCCGAGGTCGACTACCGGCNRG	GGaCGAGGcgG--cAaCGGCAAG	+	5	2	0	4.41	2.21
Chr4:74571845-74571867	74571862	3	RNA	GGCCGAGGTCGACTACCGGCNRG	GGC--tcGcaGACTAtCGGCTGG	+	5	2	0	4.41	2.21
Chr8:100462307-100462329	100462314	5	RNA	GGCCGAGGTCGACTACCGGCNRG	cGCCGtGGataACT-CCGGCCAG	-	5	1	0	4.41	2.21
Chr8:25433199-25433221	25433217	2	DNA	GGCCGAGGTCGAC-TACCGGCNRG	cGCCGAcGTCGACCggCCGGCCGG	+	4	1	0	4.41	2.21
Chr8:76767194-76767216	76767211	3	RNA	GGCCGAGGTCGACTACCGGCNRG	GGaCGAGGTtGAC--CCtatGGG	+	5	2	0	4.41	2.21
Chr4:174694702-174694724	174694718	4	RNA	GGCCGAGGTCGACTACCGGCNRG	G-CCGgGGTCGgCTcCgGGtCAG	+	5	1	0	4.41	2.21
Chr8:14624935-14624957	14624951	4	RNA	GGCCGAGGTCGACTACCGGCNRG	G-aCGgGGTCGACaACgaGCGGG	+	5	1	0	4.41	2.21
Chr10:146259686-146259708	146259691	3	RNA	GGCCGAGGTCGACTACCGGCNRG	GGgCGAGGaCGACggC--GCCAG	-	4	2	0	4.41	2.21
Chr2:126177717-126177739	126177735	2	DNA	GGCCGAGGTCGACT-ACCGGCNRG	GGttGAGGaCGACTGACaGGtGGG	+	5	1	0	4.41	2.21
Chr7:16224428-16224450	16224432	2	RNA	GGCCGAGGTCGACTACCGGCNRG	aGCaGAG--CGAacgCCGGCGGG	-	5	2	0	4.41	2.21
Chr3:15414838-15414860	15414844	4	RNA	GGCCGAGGTCGACTACCGGCNRG	GcCCGAGGcCaAC--CCtGgGAG	-	5	2	0	4.41	2.21
Chr2:243544327-243544349	243544331	2	RNA	GGCCGAGGTCGACTACCGGCNRG	GG--GcGGTgGtCTAaCGcCTAG	-	5	2	0	4.41	2.21
Chr3:233249283-233249305	233249299	4	DNA	GGCCGAGGTCGACTA--CCGGCNRG	ctCCtAGGcCGACcAATCCGGCAGG	+	5	2	0	4.41	2.21
Chr9:70137970-70137992	70137986	4	RNA	GGCCGAGGTCGACTACCGGCNRG	GGCaGAGGgCG--TAgaGGgGAG	+	5	2	0	4.41	2.21
Chr4:119179746-119179768	119179761	5	RNA	GGCCGAGGTCGACTACCGGCNRG	GGgCGAGcTCGggTA--GGaAGG	+	5	2	0	4.41	2.21
Chr7:173203467-173203489	173203472	3	RNA	GGCCGAGGTCGACTACCGGCNRG	GGa-GAcGgCGAgcACCGGCGAG	-	5	1	0	4.41	2.21
Chr2:94781043-94781065	94781058	5	RNA	GGCCGAGGTCGACTACCGGCNRG	GcCaGtGGaCGAC--CCGGCCAG	+	4	2	0	4.41	2.21
Chr1:169593712-169593734	169593719	5	RNA	GGCCGAGGTCGACTACCGGCNRG	aGaCGAGcTCGAgTA--GGaAGG	-	5	2	0	4.41	2.21
Chr4:108825550-108825572	108825566	4	RNA	GGCCGAGGTCGACTACCGGCNRG	GGC-GAGcaCGtagACCGGCCGG	+	5	1	0	4.41	2.21
Chr1:84537561-84537583	84537579	2	RNA	GGCCGAGGTCGACTACCGGCNRG	GGCCcAtcTtGgCT--CGGCCAG	+	5	2	0	4.41	2.21
Chr2:191368621-191368643	191368627	4	RNA	GGCCGAGGTCGACTACCGGCNRG	GGCaGAGGTCGACag--tGgCGG	-	5	2	0	4.41	2.21
Chr3:207125593-207125615	207125600	5	RNA	GGCCGAGGTCGACTACCGGCNRG	GGgCG-GGcCaACctCCGGCCGG	-	5	1	0	4.41	2.21
Chr8:25883914-25883936	25883921	5	RNA	GGCCGAGGTCGACTACCGGCNRG	GttCGgGGaCGtCT-CCGGCGAG	-	5	1	0	4.41	2.21
Chr10:46944409-46944431	46944414	3	X	GGCCGAGGTCGACTACCGGCNRG	GGaaGAGGTgGcCTAgCGGCTGG	-	5	0	1	3.30	2.15
Chr9:104276813-104276835	104276831	2	RNA	GGCCGAGGTCGACTACCGGCNRG	GcCCGAGGgC--CTAaCaaCAAG	+	5	2	1	3.30	2.15
Chr5:140916743-140916765	140916760	3	DNA	GGCCGAGGTCGACTAC--CGGCNRG	GaCCGtGGTCGACgACAACtcCGAG	+	5	2	1	3.30	2.15
Chr3:178918303-178918325	178918318	5	RNA	GGCCGAGGTCGACTACCGGCNRG	aGC--AGGgCaACagCCGGCCGG	+	5	2	1	3.30	2.15
Chr6:150262725-150262747	150262743	2	DNA	GGCCGAGGTCGAC-TACCGGCNRG	GGCtGAGcaCcACGTcCCGGCCGG	+	5	1	2	2.20	2.10
Chr2:34358768-34358790	34358784	4	RNA	GGCCGAGGTCGACTACCGGCNRG	GGaCGAGcTCGggTA--GGgGAG	+	5	2	2	2.20	2.10
Chr2:170250011-170250033	170250026	5	RNA	GGCCGAGGTCGACTACCGGCNRG	GGC--ttGcgGACTACCGGCTGG	+	4	2	3	1.10	2.05
Chr7:115961873-115961895	115961891	2	RNA	GGCCGAGGTCGACTACCGGCNRG	GGCCGAGGTgGgtTgCC--tGAG	+	5	2	3	1.10	2.05
Chr2:120616378-120616400	120616385	5	RNA	GGCCGAGGTCGACTACCGGCNRG	cGCCGAGaTC--CTctCcGCTGG	-	5	2	4	0.00	2.00
Chr8:33112794-33112816	33112811	3	RNA	GGCCGAGGTCGACTACCGGCNRG	a--CGAGGTCGACccCgGGCAGG	+	4	2	4	0.00	2.00
Chr5:103082943-103082965	103082960	3	RNA	GGCCGAGGTCGACTACCGGCNRG	tcgCaAGGTCGAaT-CCGGCTGG	+	5	1	4	0.00	2.00
Chr9:44214025-44214047	44214032	5	RNA	GGCCGAGGTCGACTACCGGCNRG	cGgCttGGcCGACT-CCGGCGGG	-	5	1	4	0.00	2.00
Chr4:200401971-200401993	200401978	5	RNA	GGCCGAGGTCGACTACCGGCNRG	GGCCGAGGcCGg-TgCgGGgAAG	-	5	1	4	0.00	2.00
Chr8:148563265-148563287	148563282	3	RNA	GGCCGAGGTCGACTACCGGCNRG	GtCCG--GTgcACcACCGGaCAG	+	5	2	4	0.00	2.00
Chr7:104372058-104372080	104372065	5	RNA	GGCCGAGGTCGACTACCGGCNRG	GtCCG--GTgcACcACCGGaTAG	-	5	2	4	0.00	2.00
Chr1:175297382-175297404	175297389	5	RNA	GGCCGAGGTCGACTACCGGCNRG	caCCaAGtTCGcCT-CCGGCCAG	-	5	1	4	0.00	2.00
Chr4:119510804-119510826	119510821	3	RNA	GGCCGAGGTCGACTACCGGCNRG	GGgCGAGcTCGtC-ggCGGCGGG	+	5	1	4	0.00	2.00
Chr8:103125843-103125865	103125860	3	RNA	GGCCGAGGTCGACTACCGGCNRG	GGC--tcGcgGACTACCGGCTGG	+	4	2	4	0.00	2.00
Chr5:25703564-25703586	25703581	3	RNA	GGCCGAGGTCGACTACCGGCNRG	GGaCaAG--CGACTcCCaGtAGG	+	5	2	4	0.00	2.00
Chr1:36106154-36106176	36106171	3	RNA	GGCCGAGGTCGACTACCGGCNRG	caCCGAGtTCGcCT-CCGcCGGG	+	5	1	4	0.00	2.00

Chr5:10008881-10008903	10008898	3	RNA	GGCCGAGGTCGACTACCGGCNRG	GGCCacGGTgG--cgCCGGCGAG	+	5	2	4	0.00	2.00
Chr6:124220122-124220144	124220129	5	RNA	GGCCGAGGTCGACTACCGGCNRG	GGC--ttGcgGAtTACCGGCTGG	-	5	2	4	0.00	2.00
Chr6:67659448-67659470	67659465	3	RNA	GGCCGAGGTCGACTACCGGCNRG	caCaGAcGTCGAC-gCCGGCGAG	+	5	1	4	0.00	2.00
Chr6:134674774-134674796	134674791	3	DNA	GGCCGAGGTCGAC--TACCGGCNRG	GGtCGtGGTCGACGTTgttGGCCGG	+	5	2	4	0.00	2.00
Chr2:146280432-146280454	146280449	3	RNA	GGCCGAGGTCGACTACCGGCNRG	GGgC-AGGcCaACctCCGGCCGG	+	5	1	4	0.00	2.00
Chr6:62117489-62117511	62117496	5	RNA	GGCCGAGGTCGACTACCGGCNRG	aGCCGAGGgCGcCT-CgGGCAGG	-	4	1	4	0.00	2.00
Chr7:134571105-134571127	134571122	3	RNA	GGCCGAGGTCGACTACCGGCNRG	t--CGgGGaCGtCTtCCGGCTGG	+	5	2	4	0.00	2.00
Chr1:182262755-182262777	182262762	5	RNA	GGCCGAGGTCGACTACCGGCNRG	G--CGAaGaCcACcACCtGCAGG	-	5	2	4	0.00	2.00
Chr8:91840490-91840512	91840507	3	RNA	GGCCGAGGTCGACTACCGGCNRG	G-aCGgGGTCGACaACgaGCGGG	+	5	1	4	0.00	2.00
Chr2:160145330-160145352	160145337	5	RNA	GGCCGAGGTCGACTACCGGCNRG	GtCCG--GTgcACcACCGGaTAG	-	5	2	4	0.00	2.00
Chr10:13123129-13123151	13123136	5	RNA	GGCCGAGGTCGACTACCGGCNRG	G-aCGgGGTCGACgACgaGCGGG	-	5	1	4	0.00	2.00
Chr7:3261505-3261527	3261522	3	RNA	GGCCGAGGTCGACTACCGGCNRG	GGCCGAGtaCG--TtgCcGCAGG	+	5	2	4	0.00	2.00
Chr1:273999940-273999962	273999947	5	RNA	GGCCGAGGTCGACTACCGGCNRG	GGC--tcGTgGACTACCGttTGG	-	5	2	4	0.00	2.00
Chr1:180020357-180020379	180020374	3	RNA	GGCCGAGGTCGACTACCGGCNRG	GaC--AGGcacACTAgCGGCCAG	+	5	2	4	0.00	2.00
Chr1:129321638-129321660	129321645	5	RNA	GGCCGAGGTCGACTACCGGCNRG	GGtCGgGGTtG--ggCCGGCTGG	-	5	2	4	0.00	2.00
Chr2:142398122-142398144	142398139	3	RNA	GGCCGAGGTCGACTACCGGCNRG	GGCCGAtGgCG--ggCgGGCGGG	+	5	2	4	0.00	2.00
Chr5:101667792-101667814	101667799	5	DNA	GGCCGAGGTCGAC-TACCGGCNRG	GGCCGgcGgCGAgGaACCGGCCGG	-	5	1	4	0.00	2.00
Chr10:100218755-100218777	100218762	5	RNA	GGCCGAGGTCGACTACCGGCNRG	GG--GAGGTCGgCTAggaGtTGG	-	5	2	4	0.00	2.00
Chr1:31114334-31114356	31114351	3	RNA	GGCCGAGGTCGACTACCGGCNRG	GGCCGAGGc--ACcACgtGaCGG	+	5	2	4	0.00	2.00
Chr6:46957466-46957488	46957473	5	RNA	GGCCGAGGTCGACTACCGGCNRG	GGCCGActTCG--TgtgGGCAGG	-	5	2	4	0.00	2.00
Chr1:124945096-124945118	124945103	5	RNA	GGCCGAGGTCGACTACCGGCNRG	GcCgGAGGcCGACTt--GGaAGG	-	5	2	4	0.00	2.00
Chr1:21226460-21226482	21226477	3	RNA	GGCCGAGGTCGACTACCGGCNRG	GtCttgGGT-GACcACCGGCGGG	+	5	1	4	0.00	2.00
Chr1:195135647-195135669	195135654	5	DNA	GGCCGAGGTCGACTA--CCGGCNRG	cGCaaAGGcCGACcAATCCGGCAGG	-	5	2	4	0.00	2.00
Chr3:41464102-41464124	41464119	3	RNA	GGCCGAGGTCGACTACCGGCNRG	aGCaGAG--CGAacgCCGGCTGG	+	5	2	4	0.00	2.00
Chr7:155080042-155080064	155080059	3	RNA	GGCCGAGGTCGACTACCGGCNRG	G-aCGgGGTCGACaACgaGCGGG	+	5	1	4	0.00	2.00
Chr4:51993306-51993328	51993313	5	RNA	GGCCGAGGTCGACTACCGGCNRG	GGCCGcGccCG-CTcCCGGCCGG	-	4	1	4	0.00	2.00
Chr10:37363083-37363105	37363090	5	RNA	GGCCGAGGTCGACTACCGGCNRG	GaCCGgGGTCGAC-gCgaGCAGG	-	5	1	4	0.00	2.00
Chr8:84846561-84846583	84846568	5	RNA	GGCCGAGGTCGACTACCGGCNRG	GGgCG-GGcCaACctCCGGCCAG	-	5	1	4	0.00	2.00
Chr10:136196658-136196680	136196665	5	RNA	GGCCGAGGTCGACTACCGGCNRG	GGC-GAGGTCGAggACCttgGGG	-	5	1	4	0.00	2.00
Chr4:119702439-119702461	119702446	5	DNA	GGCCGAGGTCGACTACCG--GCNRG	GGaaGAcGTCGcCgACCGCCGCGGG	-	5	2	4	0.00	2.00
Chr1:86560489-86560511	86560506	3	RNA	GGCCGAGGTCGACTACCGGCNRG	GtCCG--GTgcACcACCGGaCAG	+	5	2	4	0.00	2.00
Chr9:58389501-58389523	58389508	5	RNA	GGCCGAGGTCGACTACCGGCNRG	GGgCGAGG-CGcCTctaGGCAAG	-	5	1	4	0.00	2.00
Chr5:11844047-11844069	11844064	3	RNA	GGCCGAGGTCGACTACCGGCNRG	GGatGAGGagGACT--CGGaCGG	+	5	2	4	0.00	2.00
Chr3:124078529-124078551	124078546	3	RNA	GGCCGAGGTCGACTACCGGCNRG	GaCCGAGG-CGACgACaaGaAGG	+	5	1	4	0.00	2.00
Chr3:205364199-205364221	205364206	5	RNA	GGCCGAGGTCGACTACCGGCNRG	GtaCGtGGTtG--TgCCGGCTAG	-	5	2	4	0.00	2.00
Chr1:183235274-183235296	183235281	5	RNA	GGCCGAGGTCGACTACCGGCNRG	cGtCGAGGaCGAC-AgaGGCAAG	-	5	1	4	0.00	2.00
Chr1:16240986-16241008	16240993	5	RNA	GGCCGAGGTCGACTACCGGCNRG	G--CGcGGTCGcacgCCGGCGAG	-	5	2	4	0.00	2.00
Chr1:262648365-262648387	262648382	3	RNA	GGCCGAGGTCGACTACCGGCNRG	GGgCGAGGaCaAg--CCGcCAAG	+	5	2	4	0.00	2.00
Chr9:96047903-96047925	96047920	3	RNA	GGCCGAGGTCGACTACCGGCNRG	tGCaGAGcTCGACTA--GGtTGG	+	4	2	4	0.00	2.00
Chr2:180716190-180716212	180716207	3	X	GGCCGAGGTCGACTACCGGCNRG	GGCCGAGGTgcACcACCtGtCGG	+	5	0	4	0.00	2.00
Chr3:227746244-227746266	227746251	5	DNA	GGCCGAGGTCGAC-TACCGGCNRG	aGCacAGGTaGACTTACCaGCCGG	-	5	1	4	0.00	2.00
Chr8:38451032-38451054	38451049	3	DNA	GGCCGAGG--TCGACTACCGGCNRG	GGgCGAGGGCTCccCTACtGGaTAG	+	5	2	4	0.00	2.00
Chr8:20852244-20852266	20852261	3	RNA	GGCCGAGGTCGACTACCGGCNRG	aGCCGAGGgtGcCT-CgGGCTGG	+	5	1	4	0.00	2.00
Chr2:48116260-48116282	48116267	5	DNA	GGCCGAGGTCGA-CTACCGGCNRG	cGCCGAGGTCGAGCTtCCGGCCGG	-	2	1	4	0.00	2.00
Chr1:125436389-125436411	125436406	3	DNA	GGCCGAGGTCGACTACCGG--CNRG	GGtCGAGGTCGAgTcCgaGCCCCGG	+	5	2	4	0.00	2.00
Chr9:132705053-132705075	132705060	5	RNA	GGCCGAGGTCGACTACCGGCNRG	G-aCGgGGTCGACaACgaGCGGG	-	5	1	4	0.00	2.00
Chr7:85009248-85009270	85009265	3	DNA	GGCC-GAGGTCGACTACCGGCNRG	GGCCGGAGcTCGAtcACCcGgAGG	+	5	1	4	0.00	2.00
Chr2:31242370-31242392	31242377	5	RNA	GGCCGAGGTCGACTACCGGCNRG	G-aCGgGGTCGACaACgaGCGGG	-	5	1	4	0.00	2.00
Chr4:232476389-232476411	232476396	5	RNA	GGCCGAGGTCGACTACCGGCNRG	GcCtcgGGTCGACgAC-GGCGGG	-	5	1	4	0.00	2.00
Chr9:93973918-93973940	93973935	3	RNA	GGCCGAGGTCGACTACCGGCNRG	G-aCGgGGTCGACgACgaGCGGG	+	5	1	4	0.00	2.00
Chr6:171250440-171250462	171250457	3	RNA	GGCCGAGGTCGACTACCGGCNRG	G-aCGgGGTCGACgACaaGCGGG	+	5	1	4	0.00	2.00
Chr4:168158943-168158965	168158960	3	RNA	GGCCGAGGTCGACTACCGGCNRG	GG--GtGGcgGgCTgCCGGCTGG	+	5	2	4	0.00	2.00
Chr7:19952895-19952917	19952902	5	RNA	GGCCGAGGTCGACTACCGGCNRG	cGatGgcGTCGACT-CCGGCGAG	-	5	1	4	0.00	2.00
Chr8:97477095-97477117	97477102	5	RNA	GGCCGAGGTCGACTACCGGCNRG	G-aCGgGGTCGACaACgaGCGGG	-	5	1	4	0.00	2.00
Chr5:26300924-26300946	26300941	3	RNA	GGCCGAGGTCGACTACCGGCNRG	ccCCGAaGTCGAtTAtC--CCGG	+	5	2	4	0.00	2.00
Chr4:181155692-181155714	181155699	5	RNA	GGCCGAGGTCGACTACCGGCNRG	GtCCG--GTaGAtcACCcGCAGG	-	5	2	4	0.00	2.00
Chr2:212975375-212975397	212975392	3	RNA	GGCCGAGGTCGACTACCGGCNRG	GGCgGAGGaCa--cgCCGGCGAG	+	5	2	4	0.00	2.00
Chr7:158315712-158315734	158315719	5	RNA	GGCCGAGGTCGACTACCGGCNRG	tGCgGgcGaC--CTACCGGCGGG	-	5	2	4	0.00	2.00
Chr2:117163140-117163162	117163157	3	RNA	GGCCGAGGTCGACTACCGGCNRG	GGgCGAG-TCGcCTggCGGgCGG	+	5	1	4	0.00	2.00
Chr5:159362988-159363010	159363005	3	RNA	GGCCGAGGTCGACTACCGGCNRG	GGggGAGGTgG--TAgtGGCTAG	+	5	2	4	0.00	2.00
Chr3:86696092-86696114	86696109	3	RNA	GGCCGAGGTCGACTACCGGCNRG	tGaaGAGGc--ACTACCtGCGGG	+	5	2	4	0.00	2.00
Chr4:114353967-114353989	114353984	3	RNA	GGCCGAGGTCGACTACCGGCNRG	G-aCGgGGTCGACaACgaGCGGG	+	5	1	4	0.00	2.00
Chr2:15249276-15249298	15249293	3	RNA	GGCCGAGGTCGACTACCGGCNRG	GGC--tcGcgGACTACCGtCTGG	+	5	2	4	0.00	2.00
Chr2:206861186-206861208	206861193	5	RNA	GGCCGAGGTCGACTACCGGCNRG	G-aCGgGGTCGACaACgaGCGGG	-	5	1	4	0.00	2.00
Chr7:108634793-108634815	108634800	5	RNA	GGCCGAGGTCGACTACCGGCNRG	G-CCGAacTCaACgtCCGGCCGG	-	5	1	4	0.00	2.00
Chr10:84119606-84119628	84119613	5	RNA	GGCCGAGGTCGACTACCGGCNRG	GGCCGAGG-CGggTAgCtcCCGG	-	5	1	4	0.00	2.00
Chr3:175881186-175881208	175881193	5	DNA	GGCCGAGGTCGACTA--CCGGCNRG	ctCCaAGGcCGACcAATCCGGCAGG	-	5	2	4	0.00	2.00
Chr8:53735225-53735247	53735232	5	RNA	GGCCGAGGTCGACTACCGGCNRG	GGC-GAGGTCagCTgaCGGCAGG	-	4	1	4	0.00	2.00
Chr2:48182860-48182882	48182867	5	RNA	GGCCGAGGTCGACTACCGGCNRG	GGC--AGGgCGACcACgcGCGGG	-	4	2	4	0.00	2.00
Chr4:82988329-82988351	82988346	3	RNA	GGCCGAGGTCGACTACCGGCNRG	G-aCGgGGTCGACaACgaGCGGG	+	5	1	4	0.00	2.00
Chr5:179004863-179004885	179004870	5	RNA	GGCCGAGGTCGACTACCGGCNRG	GcCCGAGGaCaAC-cCCGGgCGG	-	5	1	4	0.00	2.00
Chr5:214597217-214597239	214597234	3	RNA	GGCCGAGGTCGACTACCGGCNRG	G-aCGgGGTCGACgACgaGCGGG	+	5	1	4	0.00	2.00
Chr4:85349755-85349777	85349772	3	RNA	GGCCGAGGTCGACTACCGGCNRG	GGCtGAGGgCaA--ACCaGgAGG	+	5	2	4	0.00	2.00
Chr5:33943533-33943555	33943550	3	RNA	GGCCGAGGTCGACTACCGGCNRG	aaCCGAGcTCG--gAaCGGCGGG	+	5	2	4	0.00	2.00
Chr1:31715609-31715631	31715626	3	DNA	GGCCG-AGGTCGACTACCGGCNRG	GGCCGCAGGTCaAtTAgCcGCGGG	+	4	1	4	0.00	2.00
Chr4:113471414-113471436	113471431	3	RNA	GGCCGAGGTCGACTACCGGCNRG	G-aCGgGGTCGACgACgaGCGGG	+	5	1	4	0.00	2.00
Chr2:139524103-139524125	139524110	5	RNA	GGCCGAGGTCGACTACCGGCNRG	tGaCGAcGTgGA--AgCGGCAGG	-	5	2	4	0.00	2.00
Chr10:23197380-23197402	23197387	5	RNA	GGCCGAGGTCGACTACCGGCNRG	GtCttgGGT-GACcACCGGCGGG	-	5	1	4	0.00	2.00
Chr2:140990156-140990178	140990173	3	RNA	GGCCGAGGTCGACTACCGGCNRG	GGagGAGcTCGACcACCG-gTAG	+	5	1	4	0.00	2.00
Chr5:5911337-5911359	5911344	5	RNA	GGCCGAGGTCGACTACCGGCNRG	GGCgGcG--CGAaTcgCGGCGGG	-	5	2	4	0.00	2.00
Chr2:46513443-46513465	46513460	3	DNA	GGCCGAG--GTCGACTACCGGCNRG	aGCtcAGCTGTCGACaACCtGCAGG	+	5	2	4	0.00	2.00
Chr10:131493815-131493837	131493822	5	RNA	GGCCGAGGTCGACTACCGGCNRG	cGgCGAGGTCaAC--CCaGCAGG	-	4	2	4	0.00	2.00
Chr5:176620259-176620281	176620276	3	X	GGCCGAGGTCGACTACCGGCNRG	GGaaGAGGTgGcCTAgCGGCTGG	+	5	0	4	0.00	2.00
Chr5:80606171-80606193	80606178	5	DNA	GGCCGAGGTCGAC-TACCGGCNRG	GGCgGcGGcCaACCTACaGGCCGG	-	5	1	4	0.00	2.00
Chr2:180498762-180498784	180498769	5	RNA	GGCCGAGGTCGACTACCGGCNRG	GGCCGAaaaCGACgAt-GGCAGG	-	5	1	4	0.00	2.00
Chr5:33265972-33265994	33265979	5	RNA	GGCCGAGGTCGACTACCGGCNRG	GGatGAGGTgGACaA--GGCGGG	-	4	2	4	0.00	2.00
Chr10:128991903-128991925	128991920	3	RNA	GGCCGAGGTCGACTACCGGCNRG	GGCCGAGcgCGA--AggGGgGAG	+	5	2	4	0.00	2.00
Chr2:34640592-34640614	34640599	5	DNA	GGCCGAGGTCGACTACCGG--CNRG	GGCCGAGGTCGAgTcCgaGCCCTGG	-	4	2	4	0.00	2.00
Chr3:351798-351820	351805	5	RNA	GGCCGAGGTCGACTACCGGCNRG	GGCtcAcG--GACTAtCGGCTGG	-	4	2	4	0.00	2.00
Chr1:162877173-162877195	162877180	5	RNA	GGCCGAGGTCGACTACCGGCNRG	GcCCatGGcCGACTgCC--CCGG	-	5	2	4	0.00	2.00
Chr1:201320643-201320665	201320660	3	DNA	GGCCGAGGTCGAC-TACCGGCNRG	GGCCGAGGTgaAgActCCGGCGAG	+	5	1	4	0.00	2.00
Chr1:127874646-127874668	127874663	3	RNA	GGCCGAGGTCGACTACCGGCNRG	GGCaGcGaTgGcCT--CGGCTAG	+	5	2	4	0.00	2.00
Chr6:129825767-129825789	129825784	3	RNA	GGCCGAGGTCGACTACCGGCNRG	GaCaG-GGTCGACaACaaGCGGG	+	5	1	4	0.00	2.00
Chr6:75850568-75850590	75850585	3	RNA	GGCCGAGGTCGACTACCGGCNRG	GttCGAGaTCt--TACCGGaAAG	+	5	2	4	0.00	2.00
Chr2:171121862-171121884	171121869	5	RNA	GGCCGAGGTCGACTACCGGCNRG	GGaCGA--TCGcgTAtCtGCTAG	-	5	2	4	0.00	2.00
Chr8:108234740-108234762	108234747	5	RNA	GGCCGAGGTCGACTACCGGCNRG	GGCgGtGGgCG--TgCCGGgCGG	-	5	2	4	0.00	2.00
Chr1:46853504-46853526	46853521	3	RNA	GGCCGAGGTCGACTACCGGCNRG	GaCgGAGGcCcACTtCC--CGAG	+	5	2	4	0.00	2.00
Chr3:221333190-221333212	221333207	3	DNA	GGCCGAGGTCGACTACCG-GCNRG	GGCCGAaGTCGAaggCCGCtCAGG	+	5	1	4	0.00	2.00
Chr2:68901534-68901556	68901541	5	RNA	GGCCGAGGTCGACTACCGGCNRG	GtCCG--GTgcACcACCGGaCAG	-	5	2	4	0.00	2.00
Chr2:127858470-127858492	127858487	3	RNA	GGCCGAGGTCGACTACCGGCNRG	GGCtGgGGgCGA--ACCGagGGG	+	5	2	4	0.00	2.00
Chr1:17372208-17372230	17372225	3	RNA	GGCCGAGGTCGACTACCGGCNRG	GGC-GAGGTCGACggCgaGaTGG	+	5	1	4	0.00	2.00
Chr6:3197598-3197620	3197605	5	RNA	GGCCGAGGTCGACTACCGGCNRG	GGC--AGGcCGAgcACCaGCCGG	-	4	2	4	0.00	2.00
Chr1:134626818-134626840	134626835	3	RNA	GGCCGAGGTCGACTACCGGCNRG	aGCgGAGGT-GctTACCGGgAGG	+	5	1	4	0.00	2.00
Chr5:47009621-47009643	47009638	3	DNA	GGC--CGAGGTCGACTACCGGCNRG	GtCCTCGgGGTCGgCTcCgGGCCAG	+	5	2	4	0.00	2.00
Chr4:197050791-197050813	197050808	3	RNA	GGCCGAGGTCGACTACCGGCNRG	GGggGtGGTCGAgaA--GGCGGG	+	5	2	4	0.00	2.00
Chr5:89093670-89093692	89093687	3	RNA	GGCCGAGGTCGACTACCGGCNRG	GGCgGAGGTgG--TtgCGGCTGG	+	4	2	4	0.00	2.00
Chr5:100470162-100470184	100470179	3	RNA	GGCCGAGGTCGACTACCGGCNRG	GGgtGcGGaCGAt--CCGGCCGG	+	5	2	4	0.00	2.00
Chr10:73256547-73256569	73256564	3	RNA	GGCCGAGGTCGACTACCGGCNRG	GGaCGAcGaCGACTAC--GaTAG	+	4	2	4	0.00	2.00
Chr5:148634002-148634024	148634019	3	DNA	GGC--CGAGGTCGACTACCGGCNRG	GGCCTCGgGGgCtACacCCGGCGGG	+	5	2	4	0.00	2.00
Chr2:175636650-175636672	175636657	5	RNA	GGCCGAGGTCGACTACCGGCNRG	GtCCtcGG--GAtaACCGGCAGG	-	5	2	4	0.00	2.00
Chr1:123267511-123267533	123267518	5	RNA	GGCCGAGGTCGACTACCGGCNRG	GGCCGAtcTCG--cAtCaGCAAG	-	5	2	4	0.00	2.00
Chr9:16169997-16170019	16170014	3	RNA	GGCCGAGGTCGACTACCGGCNRG	GtCCG--GcaGAtTACCcGCAGG	+	5	2	4	0.00	2.00
Chr6:68618996-68619018	68619003	5	DNA	GGCCGAGGTCGACTAC--CGGCNRG	GGgaGAGGTCcACgACGGCGGtGGG	-	5	2	4	0.00	2.00
Chr2:72340255-72340277	72340272	3	RNA	GGCCGAGGTCGACTACCGGCNRG	GGCCGAtGcCGAaTgCC--aAGG	+	5	2	4	0.00	2.00
Chr8:90143521-90143543	90143538	3	DNA	GGCCGAGGTCGA--CTACCGGCNRG	GcgCcAtcTCGATTCTACCGGCGAG	+	5	2	4	0.00	2.00
Chr4:187236831-187236853	187236848	3	RNA	GGCCGAGGTCGACTACCGGCNRG	a--CGAGGgtGACTtCCaGCAAG	+	5	2	4	0.00	2.00
Chr8:44510252-44510274	44510259	5	RNA	GGCCGAGGTCGACTACCGGCNRG	cGgCGAGGTCGtCTtCt--CCGG	-	5	2	4	0.00	2.00
Chr8:99886624-99886646	99886641	3	RNA	GGCCGAGGTCGACTACCGGCNRG	GGgCGgGGTCcA-TgCCGGgCGG	+	5	1	4	0.00	2.00
Chr2:77221285-77221307	77221292	5	RNA	GGCCGAGGTCGACTACCGGCNRG	cGaCGAGcctG--TACCGGCTGG	-	5	2	4	0.00	2.00
Chr1:27341220-27341242	27341227	5	RNA	GGCCGAGGTCGACTACCGGCNRG	GGC--AcGcCGcCTACgGGaTGG	-	5	2	4	0.00	2.00
Chr4:103954320-103954342	103954337	3	RNA	GGCCGAGGTCGACTACCGGCNRG	tGgCGAGG--GAggtCCGGCGAG	+	5	2	4	0.00	2.00
Chr5:110863208-110863230	110863215	5	RNA	GGCCGAGGTCGACTACCGGCNRG	GGCgGAaGTCG--TtCgGcCGAG	-	5	2	4	0.00	2.00
Chr2:6786442-6786464	6786459	3	RNA	GGCCGAGGTCGACTACCGGCNRG	GGC-tAGGTCGtCcACtGGtGGG	+	5	1	4	0.00	2.00
Chr10:33444934-33444956	33444951	3	RNA	GGCCGAGGTCGACTACCGGCNRG	cGgCGAGcTCG--TACCGaCCAG	+	4	2	4	0.00	2.00
Chr10:19705090-19705112	19705107	3	RNA	GGCCGAGGTCGACTACCGGCNRG	aGCCGAGGgtGcCT-CgGGCTGG	+	5	1	4	0.00	2.00
Chr8:136786018-136786040	136786035	3	RNA	GGCCGAGGTCGACTACCGGCNRG	GaCCGAGcgCG--gACCGtCCGG	+	5	2	4	0.00	2.00

Chr3:73246765-73246787	73246772	5	DNA	GGCCGAGGTCG-ACTACCGGCNRG	aGCCGAtGTCGGACcACgGcCTGG	-	5	1	4	0.00	2.00
Chr4:240094536-240094558	240094543	5	RNA	GGCCGAGGTCGACTACCGGCNRG	GaCgGtGGTC--CTgCtGGCAAG	-	5	2	4	0.00	2.00
Chr7:147092467-147092489	147092474	5	RNA	GGCCGAGGTCGACTACCGGCNRG	GagCGAG-TCGtCaACCGtCCAG	-	5	1	4	0.00	2.00
Chr2:123179232-123179254	123179239	5	RNA	GGCCGAGGTCGACTACCGGCNRG	cGaCGcGGTgGgCT--CGGCTAG	-	5	2	4	0.00	2.00
Chr4:67253674-67253696	67253691	3	RNA	GGCCGAGGTCGACTACCGGCNRG	G-aCGgGGTCGACgACgaGCGGG	+	5	1	4	0.00	2.00
Chr8:56289293-56289315	56289300	5	RNA	GGCCGAGGTCGACTACCGGCNRG	GGCCGAtcTCG--cAtCaGCAAG	-	5	2	4	0.00	2.00
Chr4:8852307-8852329	8852324	3	RNA	GGCCGAGGTCGACTACCGGCNRG	GGCCGAGGgCGA-gctCGGCCGG	+	4	1	4	0.00	2.00
Chr5:45238631-45238653	45238648	3	RNA	GGCCGAGGTCGACTACCGGCNRG	cGgCGAGcT--AaTACtGGCTGG	+	5	2	4	0.00	2.00
Chr4:245727074-245727096	245727091	3	RNA	GGCCGAGGTCGACTACCGGCNRG	GaCCGAGG-CGACgACaaGaAGG	+	5	1	4	0.00	2.00
Chr8:47229298-47229320	47229315	3	DNA	GGCCGAGGT-CGACTACCGGCNRG	GaCgGAGaTCCGAgcACCGGCGAG	+	5	1	4	0.00	2.00
Chr3:11648339-11648361	11648356	3	RNA	GGCCGAGGTCGACTACCGGCNRG	GGCCG-GGTCGtCTACtaGCGGG	+	3	1	4	0.00	2.00
Chr2:230278562-230278584	230278579	3	RNA	GGCCGAGGTCGACTACCGGCNRG	GGC-GAGGTCGgCcACCaGgGAG	+	4	1	4	0.00	2.00
Chr3:138979077-138979099	138979084	5	DNA	GGCCGAGGTCGACTAC--CGGCNRG	GcCCaAGGTCcACgACGACGGgCAG	-	5	2	4	0.00	2.00
Chr1:45965280-45965302	45965297	3	RNA	GGCCGAGGTCGACTACCGGCNRG	G-aCGgGGTCGACaACgaGCGGG	+	5	1	4	0.00	2.00
Chr4:15893846-15893868	15893853	5	RNA	GGCCGAGGTCGACTACCGGCNRG	GcCCGAGGcCaAC-ctCGGCGGG	-	5	1	4	0.00	2.00
Chr6:47055610-47055632	47055627	3	RNA	GGCCGAGGTCGACTACCGGCNRG	GGC-GAGGTgGAggtCCGGCCGG	+	4	1	4	0.00	2.00
Chr2:207164952-207164974	207164959	5	DNA	GGCCGAGGTCGACTACC--GGCNRG	GGCCGAGGcCGAgTcCgAGcGCTAG	-	5	2	4	0.00	2.00
Chr8:104457970-104457992	104457987	3	RNA	GGCCGAGGTCGACTACCGGCNRG	GGCCcAGGTCGgtT--CtGCCAG	+	4	2	4	0.00	2.00
Chr4:98753567-98753589	98753574	5	RNA	GGCCGAGGTCGACTACCGGCNRG	GGCgcAGtcCGACT-CCGGtAGG	-	5	1	4	0.00	2.00
Chr4:76254052-76254074	76254069	3	RNA	GGCCGAGGTCGACTACCGGCNRG	aGCaGgGcTCGACTAC-GGaCGG	+	5	1	4	0.00	2.00
Chr7:146006310-146006332	146006327	3	RNA	GGCCGAGGTCGACTACCGGCNRG	GGC--tcGcgGACTACCGcCTGG	+	5	2	4	0.00	2.00
Chr9:158471032-158471054	158471039	5	RNA	GGCCGAGGTCGACTACCGGCNRG	GGaCGAGccCGACT-CtGGtTGG	-	5	1	4	0.00	2.00
Chr10:41765069-41765091	41765086	3	RNA	GGCCGAGGTCGACTACCGGCNRG	tGCCGAGGTgGAtTgCC--tGAG	+	5	2	4	0.00	2.00
Chr4:173972750-173972772	173972757	5	RNA	GGCCGAGGTCGACTACCGGCNRG	GGC--tcGcgGACTACCGGCTGG	-	4	2	4	0.00	2.00
Chr9:95151495-95151517	95151502	5	RNA	GGCCGAGGTCGACTACCGGCNRG	G--CGAtGgtGACTtCgGGCGAG	-	5	2	4	0.00	2.00
Chr6:121337934-121337956	121337941	5	DNA	GGC--CGAGGTCGACTACCGGCNRG	GGCCTCGAGGgCtACacCCGGCGGG	-	4	2	4	0.00	2.00
Chr3:135889218-135889240	135889235	3	RNA	GGCCGAGGTCGACTACCGGCNRG	G-aCGgGGTCGACgACaaGCGGG	+	5	1	4	0.00	2.00
Chr7:74906280-74906302	74906297	3	RNA	GGCCGAGGTCGACTACCGGCNRG	GGaCcAGGTCG--TcCCaGtTGG	+	5	2	4	0.00	2.00
Chr5:5979307-5979329	5979324	3	RNA	GGCCGAGGTCGACTACCGGCNRG	GGC--tGGTtttgTACCGGCTGG	+	5	2	4	0.00	2.00
Chr10:48203183-48203205	48203190	5	DNA	GGCCGAGGTCGACT--ACCGGCNRG	GGCCGAGGagGgCTGGAgtGGCCGG	-	5	2	4	0.00	2.00
Chr10:123821326-123821348	123821343	3	DNA	GGC-CGAGGTCGACTACCGGCNRG	GcCTCGgGGaCGtCTtCCGGCTGG	+	5	1	4	0.00	2.00
Chr5:39492879-39492901	39492886	5	RNA	GGCCGAGGTCGACTACCGGCNRG	GGgCGAGGg-GACcgCCGGtGAG	-	5	1	4	0.00	2.00
Chr10:73447433-73447455	73447450	3	RNA	GGCCGAGGTCGACTACCGGCNRG	GGC-GtGGTCGAggACtGGCGAG	+	4	1	4	0.00	2.00
Chr8:47501040-47501062	47501047	5	RNA	GGCCGAGGTCGACTACCGGCNRG	GGC-GtGGTCGAggACtGGtAGG	-	5	1	4	0.00	2.00
Chr1:306408702-306408724	306408709	5	RNA	GGCCGAGGTCGACTACCGGCNRG	GGCCGAGGgC-ACctCgGGCTGG	-	4	1	4	0.00	2.00
Chr10:133023444-133023466	133023451	5	RNA	GGCCGAGGTCGACTACCGGCNRG	GGCtGAaGTCGAC--CtGGgCGG	-	4	2	4	0.00	2.00
Chr10:67597707-67597729	67597724	3	RNA	GGCCGAGGTCGACTACCGGCNRG	GGaaGAGGgtGA--AgCGGCTGG	+	5	2	4	0.00	2.00
Chr4:83768393-83768415	83768410	3	RNA	GGCCGAGGTCGACTACCGGCNRG	G-CCGAcGTCGcCTtCCaGtCGG	+	5	1	4	0.00	2.00
Chr2:234081573-234081595	234081580	5	RNA	GGCCGAGGTCGACTACCGGCNRG	GGgCGAG--CGctgACaGGCGGG	-	5	2	4	0.00	2.00
Chr2:159986500-159986522	159986517	3	RNA	GGCCGAGGTCGACTACCGGCNRG	a--CGgGGTCGACgACgaGCGGG	+	5	2	4	0.00	2.00
Chr5:202419688-202419710	202419695	5	RNA	GGCCGAGGTCGACTACCGGCNRG	GGCCGAGGcCGg-TgCaGGgAAG	-	5	1	4	0.00	2.00
Chr4:77960690-77960712	77960707	3	RNA	GGCCGAGGTCGACTACCGGCNRG	G-aCGgGGTCGACaACgaGCGGG	+	5	1	4	0.00	2.00
Chr1:168924929-168924951	168924936	5	RNA	GGCCGAGGTCGACTACCGGCNRG	GGCCGgGcaCGACgAC--GgTGG	-	5	2	4	0.00	2.00
Chr9:47542762-47542784	47542779	3	RNA	GGCCGAGGTCGACTACCGGCNRG	GGaCGAaGaCGA--ACtaGCGAG	+	5	2	4	0.00	2.00
Chr10:58657433-58657455	58657440	5	RNA	GGCCGAGGTCGACTACCGGCNRG	GGgCGAGcTCacCT--CGGgGGG	-	5	2	4	0.00	2.00
Chr5:45429843-45429865	45429850	5	DNA	GGCCGAGGT-CGACTACCGGCNRG	GGCCGAGGTGCctCTtCCaGtCAG	-	5	1	4	0.00	2.00
Chr3:225236118-225236140	225236135	3	RNA	GGCCGAGGTCGACTACCGGCNRG	GGgC-AGGcCaACccCCGGCTGG	+	5	1	4	0.00	2.00
Chr6:82232717-82232739	82232724	5	RNA	GGCCGAGGTCGACTACCGGCNRG	GtCCGAGG--GACTAtttGaGAG	-	5	2	4	0.00	2.00
Chr8:149818340-149818362	149818347	5	RNA	GGCCGAGGTCGACTACCGGCNRG	GtCCtcGG--GAtaACCGGCAGG	-	5	2	4	0.00	2.00
Chr6:98053960-98053982	98053977	3	RNA	GGCCGAGGTCGACTACCGGCNRG	GGC--ttGcgGACTACCGGCTGG	+	4	2	4	0.00	2.00
Chr3:71561986-71562008	71562003	3	RNA	GGCCGAGGTCGACTACCGGCNRG	GGCtGgGcTgGACTA--GGtTGG	+	5	2	4	0.00	2.00
Chr1:76772416-76772438	76772433	3	RNA	GGCCGAGGTCGACTACCGGCNRG	GGCCtcGG-CGAgTgCCGGCCGG	+	4	1	4	0.00	2.00
Chr6:79695220-79695242	79695227	5	RNA	GGCCGAGGTCGACTACCGGCNRG	cGaCGAGcTCGAC--CtGGgTAG	-	5	2	4	0.00	2.00
Chr4:182889866-182889888	182889883	3	RNA	GGCCGAGGTCGACTACCGGCNRG	tcCCacGGTC--CTACCtGCAGG	+	5	2	4	0.00	2.00
Chr1:20533434-20533456	20533451	3	RNA	GGCCGAGGTCGACTACCGGCNRG	GGCCGAtcTCcgCTgCCG-CCAG	+	5	1	4	0.00	2.00
Chr1:284438276-284438298	284438283	5	RNA	GGCCGAGGTCGACTACCGGCNRG	cGCCG--GcCGACcAtCGcCAAG	-	5	2	4	0.00	2.00
Chr8:27778827-27778849	27778834	5	RNA	GGCCGAGGTCGACTACCGGCNRG	GGCaatGGaCaACT-CCGGCGAG	-	5	1	4	0.00	2.00
Chr7:88047657-88047679	88047664	5	RNA	GGCCGAGGTCGACTACCGGCNRG	GcCgGAGccCGAC--CCGtCAAG	-	5	2	4	0.00	2.00
Chr5:34883315-34883337	34883332	3	RNA	GGCCGAGGTCGACTACCGGCNRG	GGCCcAGGTgtAC-AaCGGgGGG	+	5	1	4	0.00	2.00
Chr10:61211015-61211037	61211022	5	RNA	GGCCGAGGTCGACTACCGGCNRG	GGC-GcGGTCGcggACCaGCAGG	-	5	1	4	0.00	2.00
Chr7:48103098-48103120	48103115	3	RNA	GGCCGAGGTCGACTACCGGCNRG	cGtCGAttcCG-CTACCGGCGGG	+	5	1	4	0.00	2.00
Chr4:200397091-200397113	200397098	5	RNA	GGCCGAGGTCGACTACCGGCNRG	GGCCGAGGcCGg-TgCgGGgAAG	-	5	1	4	0.00	2.00
Chr8:56239433-56239455	56239450	3	RNA	GGCCGAGGTCGACTACCGGCNRG	GGCCtAtcTCG--cAtCGGCAAG	+	5	2	4	0.00	2.00
Chr4:167615394-167615416	167615401	5	RNA	GGCCGAGGTCGACTACCGGCNRG	GGCacAGGaC-ACTACatGCAGG	-	5	1	4	0.00	2.00
Chr7:124047801-124047823	124047818	3	RNA	GGCCGAGGTCGACTACCGGCNRG	G-aCGgGGTCGACgACgaGCGGG	+	5	1	4	0.00	2.00
Chr4:51152769-51152791	51152786	3	RNA	GGCCGAGGTCGACTACCGGCNRG	GGCtGAGG-CaAggACCGGgGAG	+	5	1	4	0.00	2.00
Chr9:2720314-2720336	2720321	5	RNA	GGCCGAGGTCGACTACCGGCNRG	GGC--AGGcCaACctCCGGCCGG	-	4	2	4	0.00	2.00
Chr4:163685203-163685225	163685220	3	RNA	GGCCGAGGTCGACTACCGGCNRG	aGCCGtGG-CGtCatCCGGCCGG	+	5	1	4	0.00	2.00
Chr2:58624502-58624524	58624519	3	RNA	GGCCGAGGTCGACTACCGGCNRG	GGCCGAGtaCG--TtgCcGCAGG	+	5	2	4	0.00	2.00
Chr10:23982545-23982567	23982552	5	RNA	GGCCGAGGTCGACTACCGGCNRG	GGCaccGGTCGgCTtCC--CAGG	-	5	2	4	0.00	2.00
Chr10:36872891-36872913	36872898	5	RNA	GGCCGAGGTCGACTACCGGCNRG	GGCgtAGGcCGACg--CGGtGGG	-	5	2	4	0.00	2.00
Chr4:102066013-102066035	102066020	5	RNA	GGCCGAGGTCGACTACCGGCNRG	GGCtatGGTCGA--ACCGaCGGG	-	4	2	4	0.00	2.00
Chr5:37970769-37970791	37970776	5	RNA	GGCCGAGGTCGACTACCGGCNRG	GGgCGAGcTCGgCTAg-GGCTGG	-	4	1	4	0.00	2.00
Chr10:3393507-3393529	3393524	3	RNA	GGCCGAGGTCGACTACCGGCNRG	G-aCGgGGTCGACaACgaGCGGG	+	5	1	4	0.00	2.00
Chr1:43654628-43654650	43654635	5	RNA	GGCCGAGGTCGACTACCGGCNRG	GGCgGAaGTCGtC-gCCGGtGAG	-	5	1	4	0.00	2.00
Chr8:99774357-99774379	99774374	3	RNA	GGCCGAGGTCGACTACCGGCNRG	GGCCctGGTtGAC-AgCcGCGGG	+	5	1	4	0.00	2.00
Chr5:70344497-70344519	70344504	5	RNA	GGCCGAGGTCGACTACCGGCNRG	cGCCaAGGgtGACTAC--GaCGG	-	5	2	4	0.00	2.00
Chr4:132130720-132130742	132130727	5	RNA	GGCCGAGGTCGACTACCGGCNRG	GGCgGAGtTCGtCTtCC--gGGG	-	5	2	4	0.00	2.00
Chr1:185121778-185121800	185121785	5	RNA	GGCCGAGGTCGACTACCGGCNRG	tGCtGAGGTgGAt--CtGGCCGG	-	5	2	4	0.00	2.00
Chr4:210483114-210483136	210483131	3	RNA	GGCCGAGGTCGACTACCGGCNRG	cttCtAGGT-GACcACCGGCGGG	+	5	1	4	0.00	2.00
Chr8:23101791-23101813	23101798	5	RNA	GGCCGAGGTCGACTACCGGCNRG	GGaCcAGGTCGgggAC--GCGGG	-	5	2	4	0.00	2.00
Chr2:126141962-126141984	126141969	5	RNA	GGCCGAGGTCGACTACCGGCNRG	GGCCG--GTCcACggtgGGCGGG	-	5	2	4	0.00	2.00
Chr8:135158180-135158202	135158187	5	RNA	GGCCGAGGTCGACTACCGGCNRG	GGgCGAGGcCGAt--CgGGtGGG	-	5	2	4	0.00	2.00
Chr7:148800280-148800302	148800287	5	RNA	GGCCGAGGTCGACTACCGGCNRG	G-aCGAaGatGACTACgGGCCGG	-	5	1	4	0.00	2.00
Chr2:132303340-132303362	132303347	5	RNA	GGCCGAGGTCGACTACCGGCNRG	GtCCG--GTgcACcACCGGaCAG	-	5	2	4	0.00	2.00
Chr9:112468027-112468049	112468034	5	RNA	GGCCGAGGTCGACTACCGGCNRG	GGC--AGGcCaACctCCGGCCGG	-	4	2	4	0.00	2.00
Chr10:85028172-85028194	85028189	3	RNA	GGCCGAGGTCGACTACCGGCNRG	GGgCG-GGcCaACctCCGGCCGG	+	5	1	4	0.00	2.00
Chr8:53635449-53635471	53635456	5	RNA	GGCCGAGGTCGACTACCGGCNRG	GGCCGtGGcCGA-ggCCGtCGGG	-	5	1	4	0.00	2.00
Chr1:244737668-244737690	244737675	5	RNA	GGCCGAGGTCGACTACCGGCNRG	tG--GAGGatGcCTACCGGaTAG	-	5	2	4	0.00	2.00
Chr5:140616156-140616178	140616173	3	RNA	GGCCGAGGTCGACTACCGGCNRG	GGCaGAG--CGACagagGGCGAG	+	5	2	4	0.00	2.00
Chr2:46058366-46058388	46058373	5	DNA	GGCCGAGGTCGACT--ACCGGCNRG	GGtCGtGGTCGcCTGCACCGGgTGG	-	4	2	4	0.00	2.00
Chr9:117900391-117900413	117900408	3	RNA	GGCCGAGGTCGACTACCGGCNRG	GGaCGAGa-CGACTtaaGGCAGG	+	5	1	4	0.00	2.00
Chr1:184583020-184583042	184583027	5	RNA	GGCCGAGGTCGACTACCGGCNRG	G-aCGgGGTCGACaACgaGCGGG	-	5	1	4	0.00	2.00
Chr10:97034593-97034615	97034610	3	RNA	GGCCGAGGTCGACTACCGGCNRG	GcCCGAGGcCaAC-cCCGGgCGG	+	5	1	4	0.00	2.00
Chr5:207541741-207541763	207541748	5	RNA	GGCCGAGGTCGACTACCGGCNRG	GGC--AGGcCGAgcACCaGCCGG	-	4	2	4	0.00	2.00
Chr9:141568180-141568202	141568187	5	RNA	GGCCGAGGTCGACTACCGGCNRG	cGgCGAGGgCGACT--CcGgCGG	-	5	2	4	0.00	2.00
Chr4:146716386-146716408	146716393	5	DNA	GGC-CGAGGTCGACTACCGGCNRG	GcCTCGgGGaCGtCTtCCGGCTGG	-	5	1	4	0.00	2.00
Chr8:67100173-67100195	67100190	3	DNA	GGCCGAGGT--CGACTACCGGCNRG	GtaCGAGGgAACttCTACCGGCAGG	+	5	2	4	0.00	2.00
Chr10:69819188-69819210	69819204	4	RNA	GGCCGAGGTCGACTACCGGCNRG	G-aCGgGGTCGACaACgaGCGGG	+	5	1	0	3.86	1.93
Chr2:228531948-228531970	228531965	3	RNA	GGCCGAGGTCGACTACCGGCNRG	caCCGAcGT-GAtcACCGGCAAG	+	5	1	0	3.86	1.93
Chr10:72184785-72184807	72184792	5	RNA	GGCCGAGGTCGACTACCGGCNRG	cGaCGAGGgCGACgAC-GaCAAG	-	5	1	0	3.86	1.93
Chr4:235263542-235263564	235263548	4	RNA	GGCCGAGGTCGACTACCGGCNRG	GGCCGAG--CGggTcaCGGCGAG	-	4	2	0	3.86	1.93
Chr1:217753270-217753292	217753276	4	DNA	GGC-CGAGGTCGACTACCGGCNRG	GcCTCGgGGaCGtCTtCCGGCTGG	-	5	1	0	3.86	1.93
Chr2:3196718-3196740	3196734	4	RNA	GGCCGAGGTCGACTACCGGCNRG	GGCCacGG-CGgCTgCtGGCCGG	+	5	1	0	3.86	1.93
Chr2:21986432-21986454	21986436	2	RNA	GGCCGAGGTCGACTACCGGCNRG	GcCgGAGccCGAC--CCGtCAAG	-	5	2	0	3.86	1.93
Chr2:205534476-205534498	205534482	4	RNA	GGCCGAGGTCGACTACCGGCNRG	GGggGAGGTgG--TAgaGGCTAG	-	5	2	0	3.86	1.93
Chr7:76522128-76522150	76522134	4	RNA	GGCCGAGGTCGACTACCGGCNRG	GGgCGAG--CGctgACaGGCAGG	-	5	2	0	3.86	1.93
Chr8:148286517-148286539	148286524	5	RNA	GGCCGAGGTCGACTACCGGCNRG	GGC--AGGcCGAgcACCGGCCGG	-	3	2	0	3.86	1.93
Chr3:57289981-57290003	57289986	3	RNA	GGCCGAGGTCGACTACCGGCNRG	GGC--AGGcCGAgcACCaGCCGG	-	4	2	0	3.86	1.93
Chr2:109842051-109842073	109842057	4	RNA	GGCCGAGGTCGACTACCGGCNRG	atCCGAGtgCGA-TACCtGCCAG	-	5	1	0	3.86	1.93
Chr1:1321583-1321605	1321600	3	DNA	GGCCGAGGTCGACTACC--GGCNRG	GGCgGAGGTCGtCggCaGAGGCCAG	+	5	2	0	3.86	1.93
Chr9:96883990-96884012	96884005	5	DNA	GGCCGAGGTCGACTA--CCGGCNRG	GcCCGAcaTCGcCTACGCCGtCCAG	+	5	2	0	3.86	1.93
Chr1:72990496-72990518	72990501	3	RNA	GGCCGAGGTCGACTACCGGCNRG	GGC--tcGcgGACTACCGGCTGG	-	4	2	0	3.86	1.93
Chr7:12939738-12939760	12939755	3	RNA	GGCCGAGGTCGACTACCGGCNRG	GGCCGAc--CGACgAgaGGaGAG	+	5	2	0	3.86	1.93
Chr1:117576424-117576446	117576442	2	RNA	GGCCGAGGTCGACTACCGGCNRG	GGgCGgGGTtGgCT--CGGaGAG	+	5	2	0	3.86	1.93
Chr4:24866793-24866815	24866811	2	RNA	GGCCGAGGTCGACTACCGGCNRG	GGCCGAGcgCGA--AggGGgGAG	+	5	2	0	3.86	1.93
Chr10:112563692-112563714	112563707	5	RNA	GGCCGAGGTCGACTACCGGCNRG	GGCCGAGGTCGAgctgCt-CCGG	+	5	1	0	3.86	1.93
Chr5:143946244-143946266	143946260	4	DNA	GGC-CGAGGTCGACTACCGGCNRG	GcCTCGgGGaCGtCTtCCGGCTGG	+	5	1	0	3.86	1.93
Chr10:146316369-146316391	146316386	3	RNA	GGCCGAGGTCGACTACCGGCNRG	caCCGAGtTgGAgTA--GGCGGG	+	5	2	0	3.86	1.93
Chr4:180298181-180298203	180298199	2	RNA	GGCCGAGGTCGACTACCGGCNRG	tGgCGAGcTCGACcA--GGaCAG	+	5	2	0	3.86	1.93
Chr2:205730744-205730766	205730762	2	RNA	GGCCGAGGTCGACTACCGGCNRG	GGgtcgGGTCGA--AgCGGCAGG	+	5	2	0	3.86	1.93
Chr10:89464995-89465017	89465000	3	RNA	GGCCGAGGTCGACTACCGGCNRG	G-aCGgGGTCGACgACgaGCGGG	-	5	1	0	3.86	1.93
Chr4:84113897-84113919	84113914	3	RNA	GGCCGAGGTCGACTACCGGCNRG	GGC--AtGcCGAgcACCGGCCGG	+	4	2	0	3.86	1.93
Chr2:27616155-27616177	27616170	5	RNA	GGCCGAGGTCGACTACCGGCNRG	GGCacAGGaC-ACTACaGGCAGG	+	4	1	0	3.86	1.93
Chr4:144484666-144484688	144484673	5	RNA	GGCCGAGGTCGACTACCGGCNRG	GGCtGccG-CGACcACCGGaTGG	-	5	1	0	3.86	1.93
Chr6:75123957-75123979	75123962	3	RNA	GGCCGAGGTCGACTACCGGCNRG	c-CCGgGtTCGAtTcCCGGCTGG	-	5	1	0	3.86	1.93

Chr8:44190575-44190597	44190581	4	X	GGCCGAGGTCGACTACCGGCNRG	GGCCtcGGgCGAgTACtGGCAGG	-	5	0	0	3.86	1.93
Chr6:160678466-160678488	160678472	4	RNA	GGCCGAGGTCGACTACCGGCNRG	GGCCGtGGcCGtCTgCC--aAGG	-	5	2	0	3.86	1.93
Chr3:74291805-74291827	74291823	2	RNA	GGCCGAGGTCGACTACCGGCNRG	GG--GtGGTCGAagtCCGGtGAG	+	5	2	0	3.86	1.93
Chr9:18823314-18823336	18823319	3	DNA	GGCC--GAGGTCGACTACCGGCNRG	GGCCCTGAGtTCGAaTcCCaGtGAG	-	5	2	0	3.86	1.93
Chr8:177983553-177983575	177983571	2	DNA	GGCCGAGGTCGACT--ACCGGCNRG	cGCgGAaGcCaACTCCACCGGCAGG	+	5	2	0	3.86	1.93
Chr1:127347576-127347598	127347593	3	RNA	GGCCGAGGTCGACTACCGGCNRG	GagCGAG-TCGtCaACCGtCCAG	+	5	1	0	3.86	1.93
Chr4:155736638-155736660	155736644	4	RNA	GGCCGAGGTCGACTACCGGCNRG	GGaCcAGGTCG--TcCCaGtTGG	-	5	2	0	3.86	1.93
Chr7:144874097-144874119	144874102	3	RNA	GGCCGAGGTCGACTACCGGCNRG	GctCGAaGgCGA--ACCGGCGGG	-	4	2	0	3.86	1.93
Chr4:214892603-214892625	214892607	2	RNA	GGCCGAGGTCGACTACCGGCNRG	GtCgGAGcTCGACTgC--GCCAG	-	4	2	0	3.86	1.93
Chr2:43582282-43582304	43582289	5	RNA	GGCCGAGGTCGACTACCGGCNRG	GGaCGAacT--ACctCCGGCCGG	-	5	2	0	3.86	1.93
Chr4:111778063-111778085	111778080	3	DNA	GGCCGAGGTCGACTACCG--GCNRG	GGCCGgGGTCGgCccaCGGCGCGGG	+	5	2	0	3.86	1.93
Chr7:66574006-66574028	66574021	5	RNA	GGCCGAGGTCGACTACCGGCNRG	GGC--tGcTCGACaACCacCAAG	+	5	2	0	3.86	1.93
Chr7:26177613-26177635	26177629	4	RNA	GGCCGAGGTCGACTACCGGCNRG	GGCCGAGGTgGgC-cgaGGCGGG	+	5	1	0	3.86	1.93
Chr2:233522922-233522944	233522926	2	DNA	GGC--CGAGGTCGACTACCGGCNRG	GGCCTCGgGGgCtACacCCGGCGGG	-	5	2	0	3.86	1.93
Chr6:45111647-45111669	45111662	5	RNA	GGCCGAGGTCGACTACCGGCNRG	GGCtGgGcTgGACTA--GGtTGG	+	5	2	0	3.86	1.93
Chr4:68139987-68140009	68140004	3	DNA	GGCCGAGG--TCGACTACCGGCNRG	GtaCGAcGCCTCGACTcCCtGCGGG	+	5	2	0	3.86	1.93
Chr10:3395075-3395097	3395081	4	RNA	GGCCGAGGTCGACTACCGGCNRG	GGgCG-GGcCaACctCCGGCCGG	-	5	1	0	3.86	1.93
Chr2:112130552-112130574	112130570	2	RNA	GGCCGAGGTCGACTACCGGCNRG	GGCCGAGcgCGA--AggGGgAAG	+	5	2	0	3.86	1.93
Chr2:38389569-38389591	38389585	4	RNA	GGCCGAGGTCGACTACCGGCNRG	GaCCGAGG-CGACgACaaGaAGG	+	5	1	0	3.86	1.93
Chr2:206036552-206036574	206036568	4	RNA	GGCCGAGGTCGACTACCGGCNRG	G-aCGgGGTCGACgACgaGCGGG	+	5	1	0	3.86	1.93
Chr8:95939883-95939905	95939898	5	RNA	GGCCGAGGTCGACTACCGGCNRG	cGgCGAGG--GAggtCCGGCGAG	+	5	2	0	3.86	1.93
Chr10:31129302-31129324	31129307	3	RNA	GGCCGAGGTCGACTACCGGCNRG	GGCCGgGGcgGgC--CgGGCCGG	-	5	2	0	3.86	1.93
Chr1:114440489-114440511	114440505	4	RNA	GGCCGAGGTCGACTACCGGCNRG	atCCtgGGT-GACTgCCGGCGGG	+	5	1	0	3.86	1.93
Chr5:171474749-171474771	171474764	5	RNA	GGCCGAGGTCGACTACCGGCNRG	GGgaGAGGggGACaA--GGCCGG	+	5	2	0	3.86	1.93
Chr6:133846521-133846543	133846538	3	RNA	GGCCGAGGTCGACTACCGGCNRG	GaCCGAcGaCGA-gAgCGGCTGG	+	5	1	0	3.86	1.93
Chr4:188949668-188949690	188949673	3	RNA	GGCCGAGGTCGACTACCGGCNRG	GGC--tcGcgGAtTACCGGCTGG	-	5	2	0	3.86	1.93
Chr6:63861717-63861739	63861734	3	RNA	GGCCGAGGTCGACTACCGGCNRG	GG--GcGGcCGAagtCCGGCGAG	+	5	2	0	3.86	1.93
Chr4:70742597-70742619	70742604	5	DNA	GGCCGAGGTCGACTAC--CGGCNRG	GGCgGgtGTtGAaTACTACGGCTAG	-	5	2	0	3.86	1.93
Chr9:87693730-87693752	87693737	5	RNA	GGCCGAGGTCGACTACCGGCNRG	GGCCGAGGgtGcCT-CgGGCCAG	-	4	1	0	3.86	1.93
Chr5:115279670-115279692	115279675	3	RNA	GGCCGAGGTCGACTACCGGCNRG	GaCaG-GGTCGACaACgaGCGGG	-	5	1	0	3.86	1.93
Chr7:115193485-115193507	115193491	4	DNA	GGCCGAGG--TCGACTACCGGCNRG	GGCtGAGGGCTCagCcACgGGCTAG	-	5	2	0	3.86	1.93
Chr2:117480301-117480323	117480319	2	RNA	GGCCGAGGTCGACTACCGGCNRG	GtCCG--GTgcACcACCGGaCAG	+	5	2	0	3.86	1.93
Chr7:112568374-112568396	112568389	5	DNA	GGCCGAGGTCGACTA--CCGGCNRG	ctCCaAGGcCGACcAATCCGGCAGG	+	5	2	0	3.86	1.93
Chr4:226154453-226154475	226154471	2	RNA	GGCCGAGGTCGACTACCGGCNRG	GGaCGAGGcgtACaAC--GCTAG	+	5	2	0	3.86	1.93
Chr7:100627431-100627453	100627437	4	RNA	GGCCGAGGTCGACTACCGGCNRG	cGtCatGGTCGgCT--CGGCGAG	-	5	2	0	3.86	1.93
Chr2:184513413-184513435	184513419	4	DNA	GGCCGAGGTCGACTACCGG--CNRG	GGCCGAGGcCGAgTcCgaGCCCTGG	-	5	2	0	3.86	1.93
Chr5:101166180-101166202	101166186	4	RNA	GGCCGAGGTCGACTACCGGCNRG	GGC--tcGcgGACTACCGcCTGG	-	5	2	0	3.86	1.93
Chr6:100461764-100461786	100461782	2	RNA	GGCCGAGGTCGACTACCGGCNRG	GGagGgaGTaG--TACCGGCAGG	+	5	2	0	3.86	1.93
Chr10:59328656-59328678	59328671	5	RNA	GGCCGAGGTCGACTACCGGCNRG	GGC--AGGcCGAgcACttGCCGG	+	5	2	0	3.86	1.93
Chr6:40894804-40894826	40894809	3	DNA	GGCCGAGGTCGACTA--CCGGCNRG	cGCaaAGGcCGACcAATCCGGCAGG	-	5	2	0	3.86	1.93
Chr2:130749520-130749542	130749537	3	RNA	GGCCGAGGTCGACTACCGGCNRG	GaCCGAGcTCGAaTg--GaCCAG	+	5	2	0	3.86	1.93
Chr8:148276540-148276562	148276555	5	RNA	GGCCGAGGTCGACTACCGGCNRG	GGCgGAGtTCGtCTtCC--gAGG	+	5	2	0	3.86	1.93
Chr3:214814567-214814589	214814585	2	RNA	GGCCGAGGTCGACTACCGGCNRG	GGCCGAGtgCGA--AggGGgGAG	+	5	2	0	3.86	1.93
Chr9:8615474-8615496	8615490	4	RNA	GGCCGAGGTCGACTACCGGCNRG	GG--GcGGcCGtggACCGGCGAG	+	5	2	0	3.86	1.93
Chr10:58208907-58208929	58208922	5	RNA	GGCCGAGGTCGACTACCGGCNRG	GagaGAGaTC--CTAaCGGCCGG	+	5	2	0	3.86	1.93
Chr7:86175542-86175564	86175546	2	RNA	GGCCGAGGTCGACTACCGGCNRG	GGC--tcGcgGACTACCGGCTGG	-	4	2	0	3.86	1.93
Chr2:68949268-68949290	68949284	4	RNA	GGCCGAGGTCGACTACCGGCNRG	GGCCaAatT-GgCTACaGGCAGG	+	5	1	0	3.86	1.93
Chr2:173491681-173491703	173491699	2	RNA	GGCCGAGGTCGACTACCGGCNRG	GGgCG-GGcCaACctCCGGCTGG	+	5	1	0	3.86	1.93
Chr3:52895550-52895572	52895555	3	RNA	GGCCGAGGTCGACTACCGGCNRG	tGCCG--GTgGACcACtGtCCAG	-	5	2	0	3.86	1.93
Chr1:98839477-98839499	98839482	3	RNA	GGCCGAGGTCGACTACCGGCNRG	GGCgcgGGTCGACgt--GGCAGG	-	5	2	0	3.86	1.93
Chr1:182626386-182626408	182626401	5	RNA	GGCCGAGGTCGACTACCGGCNRG	a--CGgGGTCGACgACgaGCGGG	+	5	2	0	3.86	1.93
Chr2:146523658-146523680	146523662	2	RNA	GGCCGAGGTCGACTACCGGCNRG	tGCtGAaGgC--CTAgCGGCGGG	-	5	2	0	3.86	1.93
Chr2:172138984-172139006	172138991	5	RNA	GGCCGAGGTCGACTACCGGCNRG	GGCCGAGGat-ACctCgGGCTGG	-	5	1	0	3.86	1.93
Chr2:126110818-126110840	126110836	2	RNA	GGCCGAGGTCGACTACCGGCNRG	aGggGAGaTgGACTAC-GGCTAG	+	5	1	0	3.86	1.93
Chr4:243745475-243745497	243745480	3	DNA	GGC--CGAGGTCGACTACCGGCNRG	GaCAACGgGGTCGACgACgaGCGGG	-	5	2	0	3.86	1.93
Chr1:173966036-173966058	173966054	2	RNA	GGCCGAGGTCGACTACCGGCNRG	GGaCGAcGaCGACTAC--GaTAG	+	4	2	0	3.86	1.93
Chr10:82234963-82234985	82234969	4	RNA	GGCCGAGGTCGACTACCGGCNRG	GGgCG-GGcCaACTtCCGGCCGG	-	4	1	0	3.86	1.93
Chr2:227378348-227378370	227378366	2	RNA	GGCCGAGGTCGACTACCGGCNRG	GG--GAGGTCGgCTtCgtGtGAG	+	5	2	0	3.86	1.93
Chr4:118394836-118394858	118394851	5	RNA	GGCCGAGGTCGACTACCGGCNRG	GGCtGcGcTCGgCTA--GGtAGG	+	5	2	0	3.86	1.93
Chr1:120162202-120162224	120162217	5	RNA	GGCCGAGGTCGACTACCGGCNRG	GGC-GAGGTgGAgcACgGGgTAG	+	5	1	0	3.86	1.93
Chr10:98826797-98826819	98826814	3	DNA	GGC-CGAGGTCGACTACCGGCNRG	GcCTCGAGGaCatCTtCCGGCTGG	+	5	1	0	3.86	1.93
Chr1:30188569-30188591	30188584	5	RNA	GGCCGAGGTCGACTACCGGCNRG	GGC--tcGcgGACTACCGGCTGG	+	4	2	0	3.86	1.93
Chr2:88113739-88113761	88113743	2	RNA	GGCCGAGGTCGACTACCGGCNRG	GGCttAGGgaGACT-CCGGtTGG	-	5	1	0	3.86	1.93
Chr7:72631088-72631110	72631095	5	RNA	GGCCGAGGTCGACTACCGGCNRG	GGCCGAGcT-GcCgAaCGcCTGG	-	5	1	0	3.86	1.93
Chr7:166689941-166689963	166689957	4	RNA	GGCCGAGGTCGACTACCGGCNRG	aGCCGAGGgtGcCT--CGGgTAG	+	5	2	0	3.86	1.93
Chr3:70168578-70168600	70168583	3	RNA	GGCCGAGGTCGACTACCGGCNRG	aGCCGAa--CGAtgACtGGCTAG	-	5	2	0	3.86	1.93
Chr5:211550440-211550462	211550455	5	RNA	GGCCGAGGTCGACTACCGGCNRG	GGC-aAGGTCGACagCCGagGAG	+	5	1	0	3.86	1.93
Chr2:84923420-84923442	84923435	5	RNA	GGCCGAGGTCGACTACCGGCNRG	GGCCG-GGTCGtCcACtGGCGGG	+	3	1	0	3.86	1.93
Chr3:145466295-145466317	145466302	5	RNA	GGCCGAGGTCGACTACCGGCNRG	GGCCGAaaaCGACgAt-GGCAGG	-	5	1	0	3.86	1.93
Chr1:234790404-234790426	234790410	4	RNA	GGCCGAGGTCGACTACCGGCNRG	GtCCtcGG--GAtaACCGGCAGG	-	5	2	0	3.86	1.93
Chr4:109963533-109963555	109963537	2	RNA	GGCCGAGGTCGACTACCGGCNRG	GGCCGAtcTCG--cAtCaGCAAG	-	5	2	0	3.86	1.93
Chr7:18440241-18440263	18440256	5	RNA	GGCCGAGGTCGACTACCGGCNRG	caCCaAGtTCGcCT-CCGGCCAG	+	5	1	0	3.86	1.93
Chr1:22291976-22291998	22291992	4	RNA	GGCCGAGGTCGACTACCGGCNRG	GGtCG-GGTCGtCcACtGGCGGG	+	4	1	0	3.86	1.93
Chr9:43305404-43305426	43305411	5	RNA	GGCCGAGGTCGACTACCGGCNRG	GctCGA--TCGACTcatGGCCGG	-	5	2	0	3.86	1.93
Chr4:206040861-206040883	206040877	4	RNA	GGCCGAGGTCGACTACCGGCNRG	G-CCtcGGgCGAgTACtGGCAGG	+	5	1	0	3.86	1.93
Chr2:112143688-112143710	112143704	4	RNA	GGCCGAGGTCGACTACCGGCNRG	G-aCGgGGTCGACgACgaGCGGG	+	5	1	0	3.86	1.93
Chr8:106126726-106126748	106126731	3	RNA	GGCCGAGGTCGACTACCGGCNRG	GtCaGAGaTCG--TACaGGCCAG	-	4	2	0	3.86	1.93
Chr2:2036572-2036594	2036578	4	RNA	GGCCGAGGTCGACTACCGGCNRG	GGCgGAcGTgGAC-AaCGaCCAG	-	5	1	0	3.86	1.93
Chr5:15367646-15367668	15367653	5	RNA	GGCCGAGGTCGACTACCGGCNRG	GGCCaAGGgCcACTACac-CGAG	-	5	1	0	3.86	1.93
Chr2:16626963-16626985	16626981	2	RNA	GGCCGAGGTCGACTACCGGCNRG	GtCCG--GTgcACcACCGGgCAG	+	5	2	0	3.86	1.93
Chr9:52520696-52520718	52520714	2	DNA	GGCCGAGGT--CGACTACCGGCNRG	GGCCGAcGTGGCcACTAaCcGtTGG	+	5	2	0	3.86	1.93
Chr7:33158870-33158892	33158876	4	X	GGCCGAGGTCGACTACCGGCNRG	GGaCGAcGaCGACTAgCGGtAGG	-	5	0	0	3.86	1.93
Chr9:37991745-37991767	37991752	5	RNA	GGCCGAGGTCGACTACCGGCNRG	GtCCG--GTgcACcACCGGaCAG	-	5	2	0	3.86	1.93
Chr7:97000429-97000451	97000435	4	RNA	GGCCGAGGTCGACTACCGGCNRG	GGC--AGGgCGACcACgcGtGGG	-	5	2	0	3.86	1.93
Chr7:82868060-82868082	82868075	5	RNA	GGCCGAGGTCGACTACCGGCNRG	GGC--tcGcgGACTACtGGCTGG	+	5	2	0	3.86	1.93
Chr3:163802895-163802917	163802900	3	DNA	GGCCGAGGTCGACTA--CCGGCNRG	ctCCaAGGcCGACcAATCCGGCAGG	-	5	2	1	2.75	1.88
Chr1:107894824-107894846	107894829	3	RNA	GGCCGAGGTCGACTACCGGCNRG	GGaCGAG--aGACTgaCGGgCGG	-	5	2	0	3.30	1.65
Chr9:155135-155157	155142	5	RNA	GGCCGAGGTCGACTACCGGCNRG	GGC--AGtTgGAtggCCGGCCGG	-	5	2	0	3.30	1.65
Chr10:101005287-101005309	101005305	2	RNA	GGCCGAGGTCGACTACCGGCNRG	GGCCGAGGcgGAgg--CGcCCAG	+	5	2	0	3.30	1.65
Chr5:122217141-122217163	122217146	3	RNA	GGCCGAGGTCGACTACCGGCNRG	GGC-aAGGTCaACgACCGGtGAG	-	4	1	0	3.30	1.65
Chr5:79950097-79950119	79950113	4	DNA	GGC--CGAGGTCGACTACCGGCNRG	GGCCTCGgGGgCtACacCCGGCGGG	+	5	2	0	3.30	1.65
Chr2:134230735-134230757	134230751	4	RNA	GGCCGAGGTCGACTACCGGCNRG	ctCCGcGGTCaA--ACCGGgGGG	+	5	2	0	3.30	1.65
Chr2:87737890-87737912	87737894	2	RNA	GGCCGAGGTCGACTACCGGCNRG	GG--GAGGagGtaTACgGGCAAG	-	5	2	0	3.30	1.65
Chr10:53575327-53575349	53575333	4	RNA	GGCCGAGGTCGACTACCGGCNRG	GGCCaAGGcCGgCcAt-GGCGAG	-	5	1	0	3.30	1.65
Chr3:81163555-81163577	81163560	3	RNA	GGCCGAGGTCGACTACCGGCNRG	GcCaGtGGaCGgC--CCGGCCAG	-	5	2	0	3.30	1.65
Chr1:203667593-203667615	203667609	4	RNA	GGCCGAGGTCGACTACCGGCNRG	GGagGcGGTgGtC-ACCGGCGAG	+	5	1	0	3.30	1.65
Chr2:211201974-211201996	211201979	3	RNA	GGCCGAGGTCGACTACCGGCNRG	GtCCG--GaaGAaTACCGGaAAG	-	5	2	0	3.30	1.65
Chr2:51176098-51176120	51176116	2	RNA	GGCCGAGGTCGACTACCGGCNRG	GGCCGA--TaGcCcgCCcGCCGG	+	5	2	0	3.30	1.65
Chr8:17674943-17674965	17674961	2	RNA	GGCCGAGGTCGACTACCGGCNRG	GGgCGAGGgaGACgA--GGgAGG	+	5	2	0	3.30	1.65
Chr8:78435436-78435458	78435442	4	DNA	GGC-CGAGGTCGACTACCGGCNRG	GcCTCGgGGaCGtCTtCCGGCTGG	-	5	1	0	3.30	1.65
Chr8:28603190-28603212	28603208	2	RNA	GGCCGAGGTCGACTACCGGCNRG	GcaCGAGG--GACTAtgGaCAAG	+	5	2	0	3.30	1.65
Chr5:30767483-30767505	30767487	2	RNA	GGCCGAGGTCGACTACCGGCNRG	GGC--AtcTCGAaTgCCGGtGAG	-	5	2	0	3.30	1.65
Chr2:166609198-166609220	166609204	4	DNA	GGCCGAGGTCGACTACCG--GCNRG	GGCCGgGGTCGgCccaCGGCGCGGG	-	5	2	0	3.30	1.65
Chr9:30580435-30580457	30580451	4	RNA	GGCCGAGGTCGACTACCGGCNRG	GaCaG-GGTCGACgACaaGCGGG	+	5	1	0	3.30	1.65
Chr5:190986159-190986181	190986165	4	RNA	GGCCGAGGTCGACTACCGGCNRG	GaCCaAGGTCttCTACC--gCGG	-	5	2	0	3.30	1.65
Chr1:270926327-270926349	270926342	5	RNA	GGCCGAGGTCGACTACCGGCNRG	GGC--tcGcgGACTACCGGCTGG	+	4	2	0	3.30	1.65
Chr2:12880441-12880463	12880448	5	RNA	GGCCGAGGTCGACTACCGGCNRG	tGgCGAGGTCGAggACC--tTGG	-	5	2	0	3.30	1.65
Chr8:87377392-87377414	87377398	4	RNA	GGCCGAGGTCGACTACCGGCNRG	GGCC-AcGTtGcCgACCGtCCAG	-	5	1	0	3.30	1.65
Chr8:21761405-21761427	21761421	4	RNA	GGCCGAGGTCGACTACCGGCNRG	G-aCGgGGTCGACgACgaGCGGG	+	5	1	0	3.30	1.65
Chr6:121253118-121253140	121253135	3	RNA	GGCCGAGGTCGACTACCGGCNRG	GGC--AtGTCtACTgCCcGgGAG	+	5	2	0	3.30	1.65
Chr10:118840559-118840581	118840575	4	DNA	GGCCGAGGTCGACTA--CCGGCNRG	ctCCtAGGcCGACcAATCCGGCAGG	+	5	2	0	3.30	1.65
Chr2:26369481-26369503	26369486	3	RNA	GGCCGAGGTCGACTACCGGCNRG	GGtCGgGGcC--CgACCGGtCAG	-	5	2	0	3.30	1.65
Chr9:47366433-47366455	47366449	4	RNA	GGCCGAGGTCGACTACCGGCNRG	G-aCGgGGTCGACgACgaGCGGG	+	5	1	0	3.30	1.65
Chr2:46847383-46847405	46847388	3	DNA	GGC--CGAGGTCGACTACCGGCNRG	GGCCTCGgGGgCtACacCCGGCGGG	-	5	2	0	3.30	1.65
Chr1:197247089-197247111	197247104	5	RNA	GGCCGAGGTCGACTACCGGCNRG	GGgC-AGGcCGAgcACCGGCCGG	+	4	1	0	3.30	1.65
Chr4:201985716-201985738	201985732	4	RNA	GGCCGAGGTCGACTACCGGCNRG	GG--GtcGTCGcgcACCGGCGGG	+	5	2	0	3.30	1.65
Chr6:167238488-167238510	167238495	5	DNA	GGCCGAG--GTCGACTACCGGCNRG	GGCCcAGCAGcaGACTAgCGGCCAG	-	4	2	0	3.30	1.65
Chr1:191590798-191590820	191590815	3	RNA	GGCCGAGGTCGACTACCGGCNRG	GGgCGAGtgCG--gACaGGCGGG	+	5	2	0	3.30	1.65
Chr2:149852123-149852145	149852138	5	RNA	GGCCGAGGTCGACTACCGGCNRG	GGCtGAGGcgtcCTAC--GCAGG	+	5	2	0	3.30	1.65
Chr8:23452031-23452053	23452036	3	DNA	GGC--CGAGGTCGACTACCGGCNRG	GGCCTCGgGGgCtACacCCGGCGGG	-	5	2	0	3.30	1.65
Chr8:104466062-104466084	104466066	2	RNA	GGCCGAGGTCGACTACCGGCNRG	GaggGAGG--GAgcACCGGCCAG	-	5	2	0	3.30	1.65
Chr4:204778178-204778200	204778193	5	RNA	GGCCGAGGTCGACTACCGGCNRG	GGgCG-GGcCaACctCCGGCCGG	+	5	1	0	3.30	1.65
Chr7:86848533-86848555	86848550	3	RNA	GGCCGAGGTCGACTACCGGCNRG	GGCCGAGtaCG--TtgCcGCAGG	+	5	2	0	3.30	1.65
Chr8:86712842-86712864	86712849	5	RNA	GGCCGAGGTCGACTACCGGCNRG	aaCCGAGGgCGcCT-CgGGCAGG	-	5	1	0	3.30	1.65
Chr4:45194669-45194691	45194684	5	RNA	GGCCGAGGTCGACTACCGGCNRG	GcCaGtGGaCGAC--CtGGCCAG	+	5	2	0	3.30	1.65
Chr4:224844686-224844708	224844691	3	RNA	GGCCGAGGTCGACTACCGGCNRG	G-aCGgGGTCGACaACgaGCGGG	-	5	1	0	3.30	1.65

Chr2:122879933-122879955	122879937	2	RNA	GGCCGAGGTCGACTACCGGCNRG	cGCC-AGGcCGAgctCCGGCGAG	-	5	1	0	3.30	1.65
Chr5:158999624-158999646	158999640	4	RNA	GGCCGAGGTCGACTACCGGCNRG	GGgaGAGGggGAgT--CGGCCAG	+	5	2	0	3.30	1.65
Chr10:48356902-48356924	48356906	2	DNA	GGCCGAGGTCGACTACCG--GCNRG	GGCgGAGGTCGtCTACaGAAGCCAG	-	3	2	0	3.30	1.65
Chr7:135420232-135420254	135420237	3	DNA	GGC-CGAGGTCGACTACCGGCNRG	aGCTCGAGGgCGAgTgaCGGCGAG	-	5	1	0	3.30	1.65
Chr7:31525853-31525875	31525859	4	DNA	GGC--CGAGGTCGACTACCGGCNRG	GGCGACGAGcTCGACTcCgGtgTGG	-	5	2	0	3.30	1.65
Chr2:24119474-24119496	24119492	2	DNA	GG-CCGAGGTCGACTACCGGCNRG	GGTCCGAGGcCGAggtCgGGCGAG	+	5	1	0	3.30	1.65
Chr5:9102752-9102774	9102758	4	RNA	GGCCGAGGTCGACTACCGGCNRG	GGt-aAGGgCGgCTgCCGGCTGG	-	5	1	0	3.30	1.65
Chr1:249986873-249986895	249986889	4	RNA	GGCCGAGGTCGACTACCGGCNRG	GG--GAGtTCaAaaACCGGaGGG	+	5	2	0	3.30	1.65
Chr2:79513821-79513843	79513838	3	RNA	GGCCGAGGTCGACTACCGGCNRG	GGCCGAGc-CaACgAtCGGgAGG	+	5	1	0	3.30	1.65
Chr9:158828559-158828581	158828563	2	RNA	GGCCGAGGTCGACTACCGGCNRG	tG--GAGGaaGACgACCtGCAAG	-	5	2	0	3.30	1.65
Chr1:304064781-304064803	304064798	3	RNA	GGCCGAGGTCGACTACCGGCNRG	cGgCGAGG--GAaTACgGGaAGG	+	5	2	0	3.30	1.65
Chr9:125811554-125811576	125811569	5	RNA	GGCCGAGGTCGACTACCGGCNRG	GGaCGAcGTgGAt--CtGGCGAG	+	5	2	0	3.30	1.65
Chr5:107175111-107175133	107175128	3	RNA	GGCCGAGGTCGACTACCGGCNRG	GtCCaAGcaCaACTACC--CGAG	+	5	2	0	3.30	1.65
Chr8:38140537-38140559	38140542	3	RNA	GGCCGAGGTCGACTACCGGCNRG	GGCCaAGGgtGcCT-CgGGCTAG	-	5	1	0	3.30	1.65
Chr4:144024909-144024931	144024925	4	RNA	GGCCGAGGTCGACTACCGGCNRG	GGCCcActTCcACTAgCG-CAAG	+	5	1	0	3.30	1.65
Chr7:93519602-93519624	93519607	3	DNA	GGCCGAGGTCGA--CTACCGGCNRG	GGtCGAGGgCGAGTCgAtCGGCAGG	-	4	2	0	3.30	1.65
Chr5:192960636-192960658	192960654	2	DNA	GGCCGA--GGTCGACTACCGGCNRG	GtCaGACTGGcaGACTACaGGCCAG	+	5	2	0	3.30	1.65
Chr7:16047436-16047458	16047443	5	RNA	GGCCGAGGTCGACTACCGGCNRG	GGgCGAG--CGctgACaGGCGGG	-	5	2	0	3.30	1.65
Chr4:143196923-143196945	143196941	2	DNA	GGCCGAGGTCGA--CTACCGGCNRG	GGtCGAcGTCGAGGCcAgtGGCGGG	+	5	2	0	3.30	1.65
Chr9:69062577-69062599	69062595	2	RNA	GGCCGAGGTCGACTACCGGCNRG	GGC-tAGGcCGAggACCtGCAGG	+	5	1	0	3.30	1.65
Chr3:7770701-7770723	7770718	3	DNA	GGC-CGAGGTCGACTACCGGCNRG	GGCTCcAcGcCcACTACtGGCCAG	+	5	1	0	3.30	1.65
Chr10:121024132-121024154	121024136	2	RNA	GGCCGAGGTCGACTACCGGCNRG	GGCCGAGtcCGAgTcCC--tGGG	-	5	2	0	3.30	1.65
Chr1:159291722-159291744	159291727	3	DNA	GGCC-GAGGTCGACTACCGGCNRG	GGCCGGAGGTgGAagACgaGCAGG	-	5	1	0	3.30	1.65
Chr4:54536944-54536966	54536949	3	DNA	GGCCGAGGTCGACT-ACCGGCNRG	GGttGAGGaCGACTGACaGGtGGG	-	5	1	0	3.30	1.65
Chr2:105621831-105621853	105621836	3	RNA	GGCCGAGGTCGACTACCGGCNRG	t-CCGAcGgCGtCTAgCGGCGAG	-	5	1	0	3.30	1.65
Chr7:37988950-37988972	37988966	4	RNA	GGCCGAGGTCGACTACCGGCNRG	aGCCGAGGgtGcCT-CgGGCTGG	+	5	1	0	3.30	1.65
Chr10:1696886-1696908	1696901	5	RNA	GGCCGAGGTCGACTACCGGCNRG	GGCgGcGG--GgCggCCGGCAGG	+	5	2	0	3.30	1.65
Chr8:132268614-132268636	132268620	4	RNA	GGCCGAGGTCGACTACCGGCNRG	GGCtGgGGTaG--cACCGGgAGG	-	5	2	0	3.30	1.65
Chr1:155052334-155052356	155052349	5	RNA	GGCCGAGGTCGACTACCGGCNRG	GGCaGgGG-CGACcACaaGCGGG	+	5	1	0	3.30	1.65
Chr4:10986142-10986164	10986149	5	DNA	GGCCGAGGTCGACTACC--GGCNRG	GGCgGAGGTCGtCTgCaGAGGCCAG	-	4	2	0	3.30	1.65
Chr2:72771318-72771340	72771334	4	RNA	GGCCGAGGTCGACTACCGGCNRG	GGC--tcGcaGACTACCGGCTGG	+	4	2	0	3.30	1.65
Chr10:129636421-129636443	129636437	4	RNA	GGCCGAGGTCGACTACCGGCNRG	GcCCGAGGcCGA--ACtcGgTGG	+	5	2	0	3.30	1.65
Chr3:103618456-103618478	103618474	2	DNA	GGCCGAGGTCG-ACTACCGGCNRG	GGaCGAGGgCGTACaACCacCAAG	+	5	1	0	3.30	1.65
Chr2:185635696-185635718	185635701	3	DNA	GGCCGAGGTCGACT-ACCGGCNRG	cGCaGAGGTtGtCTGACCGGtCGG	-	5	1	0	3.30	1.65
Chr7:86575947-86575969	86575952	3	DNA	GGC-CGAGGTCGACTACCGGCNRG	GcCTCGgGGaCGtCTtCCGGCTGG	-	5	1	0	3.30	1.65
Chr5:97641978-97642000	97641993	5	RNA	GGCCGAGGTCGACTACCGGCNRG	GtCCG--GTgcACcACCGGaCAG	+	5	2	0	3.30	1.65
Chr9:128092340-128092362	128092355	5	RNA	GGCCGAGGTCGACTACCGGCNRG	GGaCcAGGTCG--TcCCaGtTGG	+	5	2	0	3.30	1.65
Chr8:139942205-139942227	139942222	3	DNA	GGCCGAGGTCGACTACC--GGCNRG	GGCgGAGGTCGtCggCaGAGGCCAG	+	5	2	0	3.30	1.65
Chr3:4825390-4825412	4825396	4	RNA	GGCCGAGGTCGACTACCGGCNRG	GcCgGAGccCGAC--CCGtCAAG	-	5	2	0	3.30	1.65
Chr7:62390275-62390297	62390291	4	RNA	GGCCGAGGTCGACTACCGGCNRG	GGgCGAGcTCGgCcAC-GGCAAG	+	4	1	0	3.30	1.65
Chr8:153505433-153505455	153505438	3	RNA	GGCCGAGGTCGACTACCGGCNRG	GGtCGAGcTC--CTcCCcGCCGG	-	4	2	0	3.30	1.65
Chr8:119767152-119767174	119767169	3	RNA	GGCCGAGGTCGACTACCGGCNRG	GGatGAGGTgGACgA--GGCGGG	+	4	2	0	3.30	1.65
Chr1:73387788-73387810	73387795	5	RNA	GGCCGAGGTCGACTACCGGCNRG	G-aCGgGGTCGACaACgaGCGGG	-	5	1	0	3.30	1.65
Chr7:48100604-48100626	48100620	4	RNA	GGCCGAGGTCGACTACCGGCNRG	G-aCGgGGTCGACaACgaGCGGG	+	5	1	0	3.30	1.65
Chr2:44995534-44995556	44995552	2	RNA	GGCCGAGGTCGACTACCGGCNRG	GGCCGA--TaGcCcACCcGCCGG	+	4	2	0	3.30	1.65
Chr8:158801857-158801879	158801872	5	RNA	GGCCGAGGTCGACTACCGGCNRG	GGC--AGGcCGAgcACCGGCCGG	+	3	2	0	3.30	1.65
Chr2:115610922-115610944	115610927	3	RNA	GGCCGAGGTCGACTACCGGCNRG	aGC--AGGTgGACTtCgGGaGAG	-	5	2	0	3.30	1.65
Chr5:159188090-159188112	159188097	5	RNA	GGCCGAGGTCGACTACCGGCNRG	GGCCGAGGcCGg-TgCaGGgAAG	-	5	1	0	3.30	1.65
Chr2:108882451-108882473	108882467	4	DNA	GGCCGAGGTCGACT--ACCGGCNRG	tcCCGAGGcCGACTCCtCCGtCGAG	+	5	2	0	3.30	1.65
Chr4:120350896-120350918	120350914	2	RNA	GGCCGAGGTCGACTACCGGCNRG	GGCCGtGtTC-ACcgCCGtCGGG	+	5	1	0	3.30	1.65
Chr3:39270810-39270832	39270815	3	DNA	GGCCGAGGTCGACT-ACCGGCNRG	GGgtGAGacaGACTGACCGGCGGG	-	5	1	0	3.30	1.65
Chr6:29714832-29714854	29714850	2	RNA	GGCCGAGGTCGACTACCGGCNRG	GGggGAGGgCG--TACaGaCGAG	+	5	2	0	3.30	1.65
Chr5:199787056-199787078	199787060	2	RNA	GGCCGAGGTCGACTACCGGCNRG	GGCCGAGGcCGg-TgCgGGgAAG	-	5	1	0	3.30	1.65
Chr8:139103175-139103197	139103182	5	RNA	GGCCGAGGTCGACTACCGGCNRG	GGCgGAaGTCG--TtCgGcCGAG	-	5	2	0	3.30	1.65
Chr3:154781338-154781360	154781356	2	RNA	GGCCGAGGTCGACTACCGGCNRG	G--CGAGGaCGgCatCCcGCCGG	+	5	2	0	3.30	1.65
Chr1:18438664-18438686	18438670	4	RNA	GGCCGAGGTCGACTACCGGCNRG	GGCgGAGGTCG--TtCgGcaGAG	-	5	2	0	3.30	1.65
Chr8:153051902-153051924	153051918	4	RNA	GGCCGAGGTCGACTACCGGCNRG	GGCCGAGccCGAg--CCGagGGG	+	5	2	0	3.30	1.65
Chr2:9239214-9239236	9239218	2	RNA	GGCCGAGGTCGACTACCGGCNRG	GGgCG-GGcCaACctCCGGCCGG	-	5	1	0	3.30	1.65
Chr7:135879138-135879160	135879144	4	DNA	GGC-CGAGGTCGACTACCGGCNRG	GGCTCGgaGaCGtCTtCCGGCTGG	-	5	1	0	3.30	1.65
Chr9:15295368-15295390	15295374	4	RNA	GGCCGAGGTCGACTACCGGCNRG	GGC--ttGctGACTACCGGCTGG	-	4	2	0	3.30	1.65
Chr5:168752859-168752881	168752865	4	RNA	GGCCGAGGTCGACTACCGGCNRG	tGaCGAGGaCGAagAC-GGCAGG	-	5	1	0	3.30	1.65
Chr4:68993740-68993762	68993755	5	RNA	GGCCGAGGTCGACTACCGGCNRG	aGCaGAGGcCGA--AgCGaCCAG	+	5	2	0	3.30	1.65
Chr2:1392809-1392831	1392825	4	RNA	GGCCGAGGTCGACTACCGGCNRG	G-aCGAGGTCGACgACgaGCGGG	+	4	1	0	3.30	1.65
Chr6:169119644-169119666	169119649	3	RNA	GGCCGAGGTCGACTACCGGCNRG	GcCgGAGccCGAC--CCGtCAGG	-	5	2	0	3.30	1.65
Chr2:90107750-90107772	90107756	4	RNA	GGCCGAGGTCGACTACCGGCNRG	G-aCGgGGTCGACgACgaGCGGG	-	5	1	0	3.30	1.65
Chr6:84118713-84118735	84118717	2	RNA	GGCCGAGGTCGACTACCGGCNRG	GGCCacGGTCGAC-AaCaaCCAG	-	5	1	0	3.30	1.65
Chr1:259601813-259601835	259601829	4	DNA	GGCCGAGG--TCGACTACCGGCNRG	GcCgcAGcCATCGACTcCCGGCCAG	+	5	2	0	3.30	1.65
Chr10:149762722-149762744	149762737	5	DNA	GGCCG--AGGTCGACTACCGGCNRG	GGCCGGTAtGgCGcCTcCCtGCGAG	+	5	2	0	3.30	1.65
Chr1:79911649-79911671	79911667	2	RNA	GGCCGAGGTCGACTACCGGCNRG	GGCgGAGGcCaAaTt--GGCGAG	+	5	2	0	3.30	1.65
Chr4:142083947-142083969	142083963	4	RNA	GGCCGAGGTCGACTACCGGCNRG	GGCgacGcaCGACTACC--CGAG	+	5	2	0	3.30	1.65
Chr2:112183309-112183331	112183315	4	RNA	GGCCGAGGTCGACTACCGGCNRG	GGCCGAtGTgGAgTA--GagAAG	-	5	2	0	3.30	1.65
Chr6:148381872-148381894	148381887	5	RNA	GGCCGAGGTCGACTACCGGCNRG	GtC--tccTtGACTACCGGCCGG	+	5	2	0	3.30	1.65
Chr6:79844790-79844812	79844806	4	DNA	GGCCGAGGTCGA--CTACCGGCNRG	GGCCGAGGTCGAGTCTgagccCTGG	+	5	2	0	3.30	1.65
Chr3:76184669-76184691	76184687	2	RNA	GGCCGAGGTCGACTACCGGCNRG	GGCgGtGGct--CTAaCGGCGAG	+	5	2	0	3.30	1.65
Chr1:69014233-69014255	69014249	4	RNA	GGCCGAGGTCGACTACCGGCNRG	acCCGAGGTtGgC--CCcGCGAG	+	5	2	0	3.30	1.65
Chr6:148966046-148966068	148966062	4	RNA	GGCCGAGGTCGACTACCGGCNRG	GGCCccG--aGACTtCCaGCTAG	+	5	2	0	3.30	1.65
Chr4:34640935-34640957	34640950	5	X	GGCCGAGGTCGACTACCGGCNRG	GGCgGAGGaCGcCTACCtGCTAG	+	4	0	0	3.30	1.65
Chr1:14600836-14600858	14600843	5	DNA	GGCCGAG-GTCGACTACCGGCNRG	GttCGAGCGTCaACgAtCGGCAGG	-	5	1	0	3.30	1.65
Chr5:150381051-150381073	150381057	4	RNA	GGCCGAGGTCGACTACCGGCNRG	GGCCG--GTCGgCaACtGcaCGG	-	5	2	0	3.30	1.65
Chr8:59459813-59459835	59459817	2	RNA	GGCCGAGGTCGACTACCGGCNRG	cGCCG--GTCatCTtCCtGCGGG	-	5	2	0	3.30	1.65
Chr5:81399374-81399396	81399391	3	RNA	GGCCGAGGTCGACTACCGGCNRG	GcCCGAGccCGAgT--CGGtAGG	+	5	2	0	3.30	1.65
Chr7:56199891-56199913	56199895	2	RNA	GGCCGAGGTCGACTACCGGCNRG	GGtCGAGGTgG--gtgCGGCGAG	-	5	2	0	3.30	1.65
Chr8:27680198-27680220	27680215	3	RNA	GGCCGAGGTCGACTACCGGCNRG	GGC--AGGcCagCctCCGGCCGG	+	5	2	0	3.30	1.65
Chr7:60205438-60205460	60205442	2	RNA	GGCCGAGGTCGACTACCGGCNRG	aGCCtcGGgCGACTACt--CTGG	-	5	2	0	3.30	1.65
Chr2:46487787-46487809	46487804	3	RNA	GGCCGAGGTCGACTACCGGCNRG	GGaCcAGGTCG--TcCCaGtTGG	+	5	2	0	3.30	1.65
Chr4:122650852-122650874	122650870	2	RNA	GGCCGAGGTCGACTACCGGCNRG	GGCCGAGGcCtcCT-CgGGCTGG	+	4	1	0	3.30	1.65
Chr9:121338305-121338327	121338320	5	RNA	GGCCGAGGTCGACTACCGGCNRG	GGCtcAGG--GgCTACtGtCGGG	+	5	2	0	3.30	1.65
Chr6:81106471-81106493	81106489	2	RNA	GGCCGAGGTCGACTACCGGCNRG	GcCgGAGccCGAC--CCGtCAGG	+	5	2	0	3.30	1.65
Chr5:34015526-34015548	34015544	2	RNA	GGCCGAGGTCGACTACCGGCNRG	tGCCacGGT--ACTACttGCTAG	+	5	2	0	3.30	1.65
Chr8:113133356-113133378	113133360	2	RNA	GGCCGAGGTCGACTACCGGCNRG	cGCCGgGGTCGACTt--GGaGGG	-	4	2	0	3.30	1.65
Chr7:47143964-47143986	47143970	4	RNA	GGCCGAGGTCGACTACCGGCNRG	GGCtGAGGcCGAgg--CGGgCAG	-	5	2	0	3.30	1.65
Chr10:130917066-130917088	130917072	4	RNA	GGCCGAGGTCGACTACCGGCNRG	cGgCGAGGaCcACTAC--GgAGG	-	5	2	0	3.30	1.65
Chr5:64893484-64893506	64893489	3	RNA	GGCCGAGGTCGACTACCGGCNRG	GGgaGgGGgCGA--ACCGGCCGG	-	4	2	0	3.30	1.65
Chr5:223339829-223339851	223339846	3	RNA	GGCCGAGGTCGACTACCGGCNRG	GGC--tcGcgGACTACCGtCTGG	+	5	2	0	3.30	1.65
Chr6:153101756-153101778	153101761	3	DNA	GGCCGAGGTCGACTACCG-GCNRG	GGCCGgGGcCGACatCgGTGCTGG	-	5	1	0	3.30	1.65
Chr1:286042778-286042800	286042794	4	RNA	GGCCGAGGTCGACTACCGGCNRG	G-CCGcGcTCGACTgtCGGtTGG	+	5	1	0	3.30	1.65
Chr3:116178561-116178583	116178568	5	RNA	GGCCGAGGTCGACTACCGGCNRG	GGCCGAGGgt-ACctCgGGCTAG	-	5	1	0	3.30	1.65
Chr4:213167543-213167565	213167558	5	RNA	GGCCGAGGTCGACTACCGGCNRG	GGCCaAGG--GAgTACaacCAAG	+	5	2	0	3.30	1.65
Chr2:142259580-142259602	142259598	2	RNA	GGCCGAGGTCGACTACCGGCNRG	GGCCtAGGTgGgC--CgaGCTGG	+	5	2	0	3.30	1.65
Chr7:28220809-28220831	28220824	5	RNA	GGCCGAGGTCGACTACCGGCNRG	GGCaGAGG--GAgaACCGagAGG	+	5	2	0	3.30	1.65
Chr8:110954114-110954136	110954119	3	DNA	GGCCGAGGTCGACT--ACCGGCNRG	GGgtGgGGTCaAtTTGACCGGCGGG	-	5	2	1	2.20	1.60
Chr8:178808992-178809014	178809008	4	RNA	GGCCGAGGTCGACTACCGGCNRG	GGCCG-GGTCatCcACtGGCGGG	+	4	1	1	2.20	1.60
Chr2:66024995-66025017	66025010	5	RNA	GGCCGAGGTCGACTACCGGCNRG	GGC--AGGcCGAgcACtaGCCGG	+	5	2	1	2.20	1.60
Chr1:217527608-217527630	217527626	2	RNA	GGCCGAGGTCGACTACCGGCNRG	GGCCGAGcgCGA--AggGGgGAG	+	5	2	1	2.20	1.60
Chr5:57806840-57806862	57806845	3	RNA	GGCCGAGGTCGACTACCGGCNRG	GGC--tcGcgGACTACCGGCTGG	-	4	2	1	2.20	1.60
Chr3:112228848-112228870	112228864	4	RNA	GGCCGAGGTCGACTACCGGCNRG	GaggGAGtTCGAC--CgGGCAAG	+	5	2	1	2.20	1.60
Chr5:11517314-11517336	11517329	5	RNA	GGCCGAGGTCGACTACCGGCNRG	GGCtcgGG--GgCTACtGGCGGG	+	5	2	2	1.10	1.55
Chr2:126822592-126822614	126822597	3	RNA	GGCCGAGGTCGACTACCGGCNRG	G-aCGgGGTCGACgACgaGCGGG	-	5	1	2	1.10	1.55
Chr5:6293703-6293725	6293720	3	DNA	GGC-CGAGGTCGACTACCGGCNRG	GGCTCGgGGaCGtCTtCCGGCTGG	+	4	1	2	1.10	1.55
Chr2:222205784-222205806	222205802	2	DNA	GGC-CGAGGTCGACTACCGGCNRG	GcCTCGgGGaCGtCTtCCGGCTGG	+	5	1	2	1.10	1.55
Chr4:79497029-79497051	79497036	5	DNA	GGCCGAGGT-CGACTACCGGCNRG	GGCCGAGGTGCctCTtCCaGtCAG	-	5	1	3	0.00	1.50
Chr8:100501490-100501512	100501497	5	RNA	GGCCGAGGTCGACTACCGGCNRG	GtCCGcGtTCGACTcaC--CGAG	-	5	2	3	0.00	1.50
Chr9:4458431-4458453	4458448	3	RNA	GGCCGAGGTCGACTACCGGCNRG	GGCgatGGTCaA--AgCGGCAGG	+	5	2	3	0.00	1.50
Chr7:42527345-42527367	42527352	5	DNA	GGCCGAGGTCGACTACCGG--CNRG	GGCCGAGGTtGAggACgaGCCCTGG	-	5	2	3	0.00	1.50
Chr5:29045810-29045832	29045817	5	DNA	GGCCGAGGTCGA--CTACCGGCNRG	cGCCGAGGggGAGGCcACCGGCGGG	-	4	2	3	0.00	1.50
Chr1:123873628-123873650	123873635	5	RNA	GGCCGAGGTCGACTACCGGCNRG	GGC--tcGcgGACTACCGaCTGG	-	5	2	3	0.00	1.50
Chr10:70952836-70952858	70952853	3	RNA	GGCCGAGGTCGACTACCGGCNRG	cGCCGAcGT-GAtcACCGaCAAG	+	5	1	3	0.00	1.50
Chr7:104956963-104956985	104956970	5	RNA	GGCCGAGGTCGACTACCGGCNRG	GGCCccGGTgGcCT--CGaCAGG	-	5	2	3	0.00	1.50
Chr10:102562470-102562492	102562487	3	RNA	GGCCGAGGTCGACTACCGGCNRG	GGC--AGacCGAgctCCGGCCGG	+	5	2	3	0.00	1.50
Chr1:257485659-257485681	257485666	5	RNA	GGCCGAGGTCGACTACCGGCNRG	GGaCcAGGTCG--TcCCaGtTGG	-	5	2	3	0.00	1.50
Chr5:208851535-208851557	208851542	5	RNA	GGCCGAGGTCGACTACCGGCNRG	G-aCGgGGTCGACaACgaGCGGG	-	5	1	3	0.00	1.50
Chr7:126396588-126396610	126396605	3	RNA	GGCCGAGGTCGACTACCGGCNRG	G--tGcGGgCGAgTtCCGGCGGG	+	5	2	3	0.00	1.50
Chr4:116539376-116539398	116539383	5	RNA	GGCCGAGGTCGACTACCGGCNRG	caCCaAGtTCGcCT-CCGGCCAG	-	5	1	3	0.00	1.50
Chr4:118075945-118075967	118075952	5	RNA	GGCCGAGGTCGACTACCGGCNRG	GGaCGc--TCGgCTACttGCAGG	-	5	2	3	0.00	1.50
Chr2:114660869-114660891	114660886	3	RNA	GGCCGAGGTCGACTACCGGCNRG	GGtCGtGGTCG-CgAgtGGCTGG	+	5	1	3	0.00	1.50
Chr5:29752652-29752674	29752659	5	RNA	GGCCGAGGTCGACTACCGGCNRG	GGC--AGGcCGAgcACCGGCCGG	-	3	2	3	0.00	1.50

Chr3:195863035-195863057	195863042	5	DNA	GGCCGAGGTCGACTA--CCGGCNRG	ctCCaAGGcCGACcAATCCGGCAGG	-	5	2	3	0.00	1.50
Chr9:9851479-9851501	9851496	3	RNA	GGCCGAGGTCGACTACCGGCNRG	GGtCaAGGgCGAg--CtGGCCGG	+	5	2	3	0.00	1.50
Chr9:22545982-22546004	22545999	3	RNA	GGCCGAGGTCGACTACCGGCNRG	GGtCGcaGTCGgC--CCGtCGAG	+	5	2	3	0.00	1.50
Chr8:53892169-53892191	53892186	3	RNA	GGCCGAGGTCGACTACCGGCNRG	G-aCGgGGTCGACaACgaGCGGG	+	5	1	3	0.00	1.50
Chr4:45405850-45405872	45405857	5	RNA	GGCCGAGGTCGACTACCGGCNRG	GGaCcAGGTCG--TcCCaGaTGG	-	5	2	3	0.00	1.50
Chr3:66790130-66790152	66790137	5	RNA	GGCCGAGGTCGACTACCGGCNRG	cGtCGAGGgCGACT--CcGgAAG	-	5	2	3	0.00	1.50
Chr2:58887090-58887112	58887107	3	RNA	GGCCGAGGTCGACTACCGGCNRG	GGgCG-GGcCaACctCCGGCCGG	+	5	1	3	0.00	1.50
Chr3:193999605-193999627	193999622	3	RNA	GGCCGAGGTCGACTACCGGCNRG	GGCCGAGGcCGAC--CgGGaCAG	+	3	2	3	0.00	1.50
Chr4:136515459-136515481	136515466	5	RNA	GGCCGAGGTCGACTACCGGCNRG	aGCCGAG--aGAgTcCCtGCGGG	-	5	2	3	0.00	1.50
Chr3:230050058-230050080	230050065	5	RNA	GGCCGAGGTCGACTACCGGCNRG	GtCCG--GTgcACcACCGGaTAG	-	5	2	3	0.00	1.50
Chr6:55177304-55177326	55177321	3	RNA	GGCCGAGGTCGACTACCGGCNRG	GcgCGAGGTCtgtTAC--GCCAG	+	5	2	3	0.00	1.50
Chr10:100294613-100294635	100294630	3	RNA	GGCCGAGGTCGACTACCGGCNRG	t--CGAGGgCGACgtCCtGCAGG	+	5	2	3	0.00	1.50
Chr10:32442091-32442113	32442098	5	RNA	GGCCGAGGTCGACTACCGGCNRG	GGCtGgGcTgGACTA--GGtTGG	-	5	2	3	0.00	1.50
Chr8:64079907-64079929	64079924	3	RNA	GGCCGAGGTCGACTACCGGCNRG	GGaCaAcGaCGACTAC--GaTAG	+	5	2	3	0.00	1.50
Chr9:7723232-7723254	7723239	5	DNA	GGCCGAGGTCGA--CTACCGGCNRG	GGCCGAGGcCGAGGCTgagGcCGAG	-	5	2	3	0.00	1.50
Chr2:155169783-155169805	155169800	3	RNA	GGCCGAGGTCGACTACCGGCNRG	GGtCG--GTCtACTcCtGGgAAG	+	5	2	3	0.00	1.50
Chr4:33876484-33876506	33876501	3	RNA	GGCCGAGGTCGACTACCGGCNRG	tGgCt-cGTgGACTACCGGCTGG	+	5	1	3	0.00	1.50
Chr6:73589961-73589983	73589968	5	DNA	GGCCGAGGTCGACTACCGG--CNRG	GGCtGAGGgCGAtTgCCtGCTCCAG	-	5	2	3	0.00	1.50
Chr8:40668907-40668929	40668914	5	RNA	GGCCGAGGTCGACTACCGGCNRG	GtCCGAGGT-GAgaAaCaGCCGG	-	5	1	3	0.00	1.50
Chr2:128137627-128137649	128137644	3	DNA	GGCCGAGGTCGACTACCGG-CNRG	cGCCGAcGTCtACTcCaGGCCCGG	+	5	1	3	0.00	1.50
Chr4:228599171-228599193	228599178	5	RNA	GGCCGAGGTCGACTACCGGCNRG	aaCCaAGtTCGcCT-CCGGCCAG	-	5	1	3	0.00	1.50
Chr2:213719094-213719116	213719101	5	RNA	GGCCGAGGTCGACTACCGGCNRG	GtCCG--GcCGAtcACCcGCAGG	-	5	2	3	0.00	1.50
Chr2:124931731-124931753	124931748	3	RNA	GGCCGAGGTCGACTACCGGCNRG	tGCCGtGGTCGcCg--CGGCAGG	+	4	2	3	0.00	1.50
Chr5:84770055-84770077	84770062	5	RNA	GGCCGAGGTCGACTACCGGCNRG	GGC--AGGcCGAgcACCGGCCGG	-	3	2	3	0.00	1.50
Chr4:63176355-63176377	63176372	3	DNA	GGCCGAGGTCGA--CTACCGGCNRG	GcgCcAtcTCGATTCTACCGGCGAG	+	5	2	3	0.00	1.50
Chr6:40000900-40000922	40000907	5	RNA	GGCCGAGGTCGACTACCGGCNRG	GGaCGAtGTCGACgACt--gTAG	-	5	2	3	0.00	1.50
Chr4:111008653-111008675	111008660	5	X	GGCCGAGGTCGACTACCGGCNRG	GGaaGAGGTgGcCTAgCGGCTGG	-	5	0	3	0.00	1.50
Chr4:188538030-188538052	188538037	5	RNA	GGCCGAGGTCGACTACCGGCNRG	G-aCGgGGTCGACgACgaGCGGG	-	5	1	3	0.00	1.50
Chr3:77466751-77466773	77466768	3	RNA	GGCCGAGGTCGACTACCGGCNRG	tG--GAtGTCGcCTAgCGGgGGG	+	5	2	3	0.00	1.50
Chr5:157330107-157330129	157330114	5	DNA	GGC-CGAGGTCGACTACCGGCNRG	GcCTCGgGGaCGtCTtCCGGCTGG	-	5	1	3	0.00	1.50
Chr5:11328005-11328027	11328012	5	DNA	GGCCGAG-GTCGACTACCGGCNRG	GttCGAGCGTCaACgAtCGGCGGG	-	5	1	3	0.00	1.50
Chr10:39633639-39633661	39633656	3	DNA	GGCCGA-GGTCGACTACCGGCNRG	GGCCaACGGTCGACTACCacCGGG	+	3	1	3	0.00	1.50
Chr2:219570108-219570130	219570125	3	DNA	GGCCGAGGTCGACTACC--GGCNRG	GGCgGAGGTCGtCggCtGAGGCCAG	+	5	2	3	0.00	1.50
Chr8:77297147-77297169	77297164	3	RNA	GGCCGAGGTCGACTACCGGCNRG	GGCgGAtGTgGACTA--GaCTAG	+	4	2	3	0.00	1.50
Chr1:74967262-74967284	74967279	3	RNA	GGCCGAGGTCGACTACCGGCNRG	GaCaG-GGTCGACgACaaGCGGG	+	5	1	3	0.00	1.50
Chr1:78346621-78346643	78346628	5	RNA	GGCCGAGGTCGACTACCGGCNRG	GtCCG--GTgcACcACCGGaCAG	-	5	2	3	0.00	1.50
Chr4:91865786-91865808	91865793	5	RNA	GGCCGAGGTCGACTACCGGCNRG	GGCacAGaTCaACTtC-GGCGAG	-	5	1	3	0.00	1.50
Chr6:80911482-80911504	80911499	3	RNA	GGCCGAGGTCGACTACCGGCNRG	GGgCGAGG--GtaTggCGGCTGG	+	5	2	3	0.00	1.50
Chr8:85467880-85467902	85467887	5	DNA	GGCCGAGGTCGACT-ACCGGCNRG	cGCCGcGGatGACTCACCGGaTAG	-	5	1	3	0.00	1.50
Chr5:11142263-11142285	11142280	3	DNA	GGCCGAG-GTCGACTACCGGCNRG	GttCGAGCGTCaACgAtCGGCAGG	+	5	1	3	0.00	1.50
Chr4:42889199-42889221	42889206	5	RNA	GGCCGAGGTCGACTACCGGCNRG	GGC--tcGcgGACTACCGGCTGG	-	4	2	3	0.00	1.50
Chr1:85673274-85673296	85673281	5	RNA	GGCCGAGGTCGACTACCGGCNRG	GGC--tcGcgGACTACCGtCTGG	-	5	2	3	0.00	1.50
Chr2:206150669-206150691	206150676	5	RNA	GGCCGAGGTCGACTACCGGCNRG	caCCaAGtTCGcCT-CCGGCCAG	-	5	1	3	0.00	1.50
Chr1:286083702-286083724	286083719	3	RNA	GGCCGAGGTCGACTACCGGCNRG	GGCCGAG--CGAgTgCttGaTGG	+	5	2	3	0.00	1.50
Chr1:81781405-81781427	81781422	3	RNA	GGCCGAGGTCGACTACCGGCNRG	GGgCGAG--CGctgACaGGCGGG	+	5	2	3	0.00	1.50
Chr8:69059183-69059205	69059190	5	RNA	GGCCGAGGTCGACTACCGGCNRG	GGCCGAGGgt-ACctCaGGCTAG	-	5	1	3	0.00	1.50
Chr7:43056476-43056498	43056493	3	RNA	GGCCGAGGTCGACTACCGGCNRG	GGCCcgGaTC--CTcCCaGCGAG	+	5	2	3	0.00	1.50
Chr7:85795292-85795314	85795299	5	RNA	GGCCGAGGTCGACTACCGGCNRG	GGCCatGGcCGAgT--CcGCTAG	-	5	2	3	0.00	1.50
Chr9:107862653-107862675	107862670	3	RNA	GGCCGAGGTCGACTACCGGCNRG	GGC--tcGcgGACTACCGGCTGG	+	4	2	3	0.00	1.50
Chr2:188710859-188710881	188710876	3	DNA	GGCCGAGG-TCGACTACCGGCNRG	GGCCGAGaAgCGcCTcCCGGCGGG	+	4	1	3	0.00	1.50
Chr10:53404957-53404979	53404964	5	RNA	GGCCGAGGTCGACTACCGGCNRG	GGgaGAGGct--CTACtGGCCGG	-	5	2	3	0.00	1.50
Chr3:202112108-202112130	202112125	3	RNA	GGCCGAGGTCGACTACCGGCNRG	GGtCGAtGcCGACaAC--GaGAG	+	5	2	3	0.00	1.50
Chr1:223701304-223701326	223701321	3	RNA	GGCCGAGGTCGACTACCGGCNRG	GGtgGAGaTCaAC-ACCGGgAGG	+	5	1	3	0.00	1.50
Chr1:244285271-244285293	244285288	3	DNA	GGCCGAGGTCGACTACCG-GCNRG	GGCtGAGGaCGACaAggGAGCGGG	+	5	1	3	0.00	1.50
Chr9:47069710-47069732	47069727	3	RNA	GGCCGAGGTCGACTACCGGCNRG	GGCgcAGcTCaACT-CCGGCGAG	+	4	1	3	0.00	1.50
Chr7:145989044-145989066	145989061	3	DNA	GGCCGAGGTCGACTACCGG--CNRG	GGCCGAGtcCGAgccCCGGGTCGGG	+	5	2	3	0.00	1.50
Chr10:96085458-96085480	96085465	5	RNA	GGCCGAGGTCGACTACCGGCNRG	aGCaGAGGcCGA--AgCGaCCAG	-	5	2	3	0.00	1.50
Chr3:71530987-71531009	71531004	3	RNA	GGCCGAGGTCGACTACCGGCNRG	GGCCGgGGcgGgC--CgGGCCGG	+	5	2	3	0.00	1.50
Chr1:134632198-134632220	134632215	3	RNA	GGCCGAGGTCGACTACCGGCNRG	cGCgGgGGTC-ACcAgCGGCGAG	+	5	1	3	0.00	1.50
Chr9:1353433-1353455	1353450	3	RNA	GGCCGAGGTCGACTACCGGCNRG	GG--GccGTCGACgACCGagAAG	+	5	2	3	0.00	1.50
Chr7:8233790-8233812	8233797	5	RNA	GGCCGAGGTCGACTACCGGCNRG	GGCCGAGcgCGA--AggGGgGAG	-	5	2	3	0.00	1.50
Chr4:243915434-243915456	243915441	5	RNA	GGCCGAGGTCGACTACCGGCNRG	G-aCGgGGTCGACaACgaGCGGG	-	5	1	3	0.00	1.50
Chr6:36665946-36665968	36665953	5	RNA	GGCCGAGGTCGACTACCGGCNRG	GGagGcGG-CGAgcACCGGCGAG	-	5	1	3	0.00	1.50
Chr2:106984011-106984033	106984018	5	RNA	GGCCGAGGTCGACTACCGGCNRG	GG--GAGGTCGACaAggGagGAG	-	5	2	3	0.00	1.50
Chr8:63793283-63793305	63793290	5	RNA	GGCCGAGGTCGACTACCGGCNRG	GGC--tcGcaGACTACCGGCTGG	-	4	2	3	0.00	1.50
Chr8:98099744-98099766	98099761	3	RNA	GGCCGAGGTCGACTACCGGCNRG	GGCgGAGGTCtAtTA--GGgAGG	+	4	2	3	0.00	1.50
Chr2:49880304-49880326	49880311	5	DNA	GGCCGAG--GTCGACTACCGGCNRG	cGCCGAaTCGTCGtCcACCtGCCAG	-	5	2	3	0.00	1.50
Chr8:94951551-94951573	94951568	3	DNA	GGCCGAGGTCGAC-TACCGGCNRG	aGCtGAGGaCGACGTAgCGGgCGG	+	5	1	3	0.00	1.50
Chr4:282017-282039	282034	3	DNA	GGCCGAGGTCGA--CTACCGGCNRG	GcgCcAtaTCGATTCTACCGGCGAG	+	5	2	3	0.00	1.50
Chr4:146362552-146362574	146362569	3	RNA	GGCCGAGGTCGACTACCGGCNRG	G-aCGgGGTCGACgACgaGCGGG	+	5	1	3	0.00	1.50
Chr6:150702946-150702968	150702953	5	RNA	GGCCGAGGTCGACTACCGGCNRG	GGaCGAcccCGACT-CCGGtTGG	-	5	1	3	0.00	1.50
Chr6:6554771-6554793	6554788	3	RNA	GGCCGAGGTCGACTACCGGCNRG	GcCCGAGGcCGA--ACtcGgTGG	+	5	2	3	0.00	1.50
Chr8:139110353-139110375	139110360	5	RNA	GGCCGAGGTCGACTACCGGCNRG	cGCCGAaGTCGAtcA--GtCGAG	-	5	2	3	0.00	1.50
Chr4:246679674-246679696	246679681	5	RNA	GGCCGAGGTCGACTACCGGCNRG	GGCCt--GcCaACTtCCGGgCAG	-	5	2	3	0.00	1.50
Chr4:148403328-148403350	148403335	5	RNA	GGCCGAGGTCGACTACCGGCNRG	GGaCtcG--CGACTtCCGcCGGG	-	5	2	3	0.00	1.50
Chr8:44495456-44495478	44495473	3	RNA	GGCCGAGGTCGACTACCGGCNRG	GGCCGAcGgCa--TACCtGgAGG	+	5	2	3	0.00	1.50
Chr7:137697183-137697205	137697190	5	RNA	GGCCGAGGTCGACTACCGGCNRG	GGaCcAGGTCG--TcCCaGtTGG	-	5	2	3	0.00	1.50
Chr6:100859359-100859381	100859366	5	RNA	GGCCGAGGTCGACTACCGGCNRG	GGC--AGGcCaACccCCGGCCGG	-	4	2	3	0.00	1.50
Chr2:134158352-134158374	134158359	5	RNA	GGCCGAGGTCGACTACCGGCNRG	GGC--tcGcaGACTACCGGCTGG	-	4	2	3	0.00	1.50
Chr6:172138342-172138364	172138359	3	RNA	GGCCGAGGTCGACTACCGGCNRG	t--CGgGGaCGtCTtCCGGCTGG	+	5	2	3	0.00	1.50
Chr9:34716853-34716875	34716870	3	RNA	GGCCGAGGTCGACTACCGGCNRG	aGCCtAGGTtG--TACaGaCCAG	+	5	2	3	0.00	1.50
Chr2:58230730-58230752	58230747	3	RNA	GGCCGAGGTCGACTACCGGCNRG	GaCgaAGGTgGACTA--GtCAGG	+	5	2	3	0.00	1.50
Chr7:66577596-66577618	66577603	5	RNA	GGCCGAGGTCGACTACCGGCNRG	GGCCGAGccCGAg--CCGtgGGG	-	5	2	3	0.00	1.50
Chr10:74059386-74059408	74059403	3	RNA	GGCCGAGGTCGACTACCGGCNRG	GGgCG-GGcCaACctCCGGCCGG	+	5	1	3	0.00	1.50
Chr1:258847360-258847382	258847367	5	RNA	GGCCGAGGTCGACTACCGGCNRG	GGC--AGGaCGcCcAgCGGCAGG	-	4	2	3	0.00	1.50
Chr5:87876141-87876163	87876148	5	DNA	GGCCGAGGTCGACTA--CCGGCNRG	tGCaaAGGTCGACcAATCCGGCAGG	-	4	2	3	0.00	1.50
Chr10:22027543-22027565	22027560	3	RNA	GGCCGAGGTCGACTACCGGCNRG	G--aGcGGgaGACgACCGGCTGG	+	5	2	3	0.00	1.50
Chr3:132257332-132257354	132257349	3	DNA	GGC-CGAGGTCGACTACCGGCNRG	GcCTCGgGGaCGtCTtCCGGCTGG	+	5	1	3	0.00	1.50
Chr4:138186651-138186673	138186668	3	RNA	GGCCGAGGTCGACTACCGGCNRG	GGgCGcGG-CGAggtCCGGCCGG	+	5	1	3	0.00	1.50
Chr2:26553003-26553025	26553010	5	RNA	GGCCGAGGTCGACTACCGGCNRG	GGCCGAGGTCGgC--CCaGgGAG	-	3	2	3	0.00	1.50
Chr3:205099959-205099981	205099966	5	RNA	GGCCGAGGTCGACTACCGGCNRG	tGCtGAGGTCG--TcCCtGaTAG	-	5	2	3	0.00	1.50
Chr3:12054105-12054127	12054122	3	RNA	GGCCGAGGTCGACTACCGGCNRG	tGgaGAGGTaGACcAC--GCTGG	+	5	2	3	0.00	1.50
Chr4:9116443-9116465	9116450	5	RNA	GGCCGAGGTCGACTACCGGCNRG	cGaCGAGaTCGACcAC--GCCAG	-	4	2	3	0.00	1.50
Chr7:72561296-72561318	72561313	3	RNA	GGCCGAGGTCGACTACCGGCNRG	GGgCGAGcTCtt-TAgCGGCGAG	+	5	1	3	0.00	1.50
Chr7:45607765-45607787	45607782	3	DNA	GGCCGAGGTCGACTACCGG-CNRG	cGCCGtGGTCGAgcACCGGCgAAG	+	5	1	3	0.00	1.50
Chr9:63648216-63648238	63648233	3	RNA	GGCCGAGGTCGACTACCGGCNRG	GGCCcAGaTtGAaT--CGcCCAG	+	5	2	3	0.00	1.50
Chr8:145308585-145308607	145308592	5	DNA	GGCCGAGGT--CGACTACCGGCNRG	aGaCGAGGTTCCGACcAagGGCAGG	-	5	2	3	0.00	1.50
Chr6:90307141-90307163	90307158	3	DNA	GGCCGAGGTCGAC-TACCGGCNRG	GtCCtcGGaCGACGTgCCGGCCGG	+	5	1	3	0.00	1.50
Chr4:85644323-85644345	85644340	3	RNA	GGCCGAGGTCGACTACCGGCNRG	GGggGgGtTCGAC--CgGGCAGG	+	5	2	3	0.00	1.50
Chr6:65905996-65906018	65906003	5	RNA	GGCCGAGGTCGACTACCGGCNRG	aGCCGcG--tGAaTtCCGGCCAG	-	5	2	3	0.00	1.50
Chr5:62958383-62958405	62958400	3	RNA	GGCCGAGGTCGACTACCGGCNRG	GGgCG-GGcCaACctCCGGCCGG	+	5	1	3	0.00	1.50
Chr3:221386973-221386995	221386990	3	RNA	GGCCGAGGTCGACTACCGGCNRG	GtCCG--GTgcACcACCGGaCAG	+	5	2	3	0.00	1.50
Chr9:107556476-107556498	107556483	5	RNA	GGCCGAGGTCGACTACCGGCNRG	GtCCG--GTgcACcACCGGaCAG	-	5	2	3	0.00	1.50
Chr8:17884352-17884374	17884359	5	RNA	GGCCGAGGTCGACTACCGGCNRG	G-aCGgGGTCGACgACgaGCGGG	-	5	1	3	0.00	1.50
Chr3:200994462-200994484	200994479	3	RNA	GGCCGAGGTCGACTACCGGCNRG	GGC-GcaGTCaACggCCGGCGGG	+	5	1	3	0.00	1.50
Chr2:168093613-168093635	168093630	3	RNA	GGCCGAGGTCGACTACCGGCNRG	GGC--ttGcgGACTACCGGCTGG	+	4	2	3	0.00	1.50
Chr1:104336389-104336411	104336406	3	RNA	GGCCGAGGTCGACTACCGGCNRG	GGCgGAGcTtGACTAC-GGCGAG	+	3	1	3	0.00	1.50
Chr5:204482443-204482465	204482450	5	DNA	GGCCGAGGTCGAC-TACCGGCNRG	aGCtGAGGaCGACGTAgCGGgCGG	-	5	1	3	0.00	1.50
Chr5:10862470-10862492	10862487	3	RNA	GGCCGAGGTCGACTACCGGCNRG	cGCC-AGGTCGcggACCGtCCGG	+	5	1	3	0.00	1.50
Chr6:75926789-75926811	75926806	3	RNA	GGCCGAGGTCGACTACCGGCNRG	G-CtGAGaTCGcCTAgCtGCGGG	+	5	1	3	0.00	1.50
Chr7:38878640-38878662	38878657	3	RNA	GGCCGAGGTCGACTACCGGCNRG	caCCGAGtTgGAgTA--GGCGGG	+	5	2	3	0.00	1.50
Chr3:160758395-160758417	160758412	3	RNA	GGCCGAGGTCGACTACCGGCNRG	cGaCGAGcTaGACT-CCGGtGAG	+	5	1	3	0.00	1.50
Chr10:53898069-53898091	53898086	3	DNA	G--GCCGAGGTCGACTACCGGCNRG	GCTGCCGAGGTCGAaTgtCtcCGGG	+	5	2	3	0.00	1.50
Chr7:97736536-97736558	97736553	3	RNA	GGCCGAGGTCGACTACCGGCNRG	GGtCGgcGgCGAC-AgCGGCAGG	+	5	1	3	0.00	1.50
Chr2:88893807-88893829	88893814	5	RNA	GGCCGAGGTCGACTACCGGCNRG	GG--GAcaTCcgCTACCtGCGGG	-	5	2	3	0.00	1.50
Chr4:132618196-132618218	132618213	3	RNA	GGCCGAGGTCGACTACCGGCNRG	GaCtGcGGcCGACTAC-GGaGGG	+	5	1	3	0.00	1.50
Chr2:121424822-121424844	121424829	5	RNA	GGCCGAGGTCGACTACCGGCNRG	tGCaGAGGTCcACa--CtGCAGG	-	5	2	3	0.00	1.50
Chr1:119438081-119438103	119438098	3	RNA	GGCCGAGGTCGACTACCGGCNRG	GGCaGtGGct--CTAaCGGCGAG	+	5	2	3	0.00	1.50
Chr8:93256144-93256166	93256151	5	RNA	GGCCGAGGTCGACTACCGGCNRG	GGCCt--GcCaACTtCCGGgCAG	-	5	2	3	0.00	1.50
Chr3:68340422-68340444	68340429	5	RNA	GGCCGAGGTCGACTACCGGCNRG	GGC-GAGaTCGACTtggGGaAAG	-	5	1	3	0.00	1.50
Chr2:116257352-116257374	116257359	5	RNA	GGCCGAGGTCGACTACCGGCNRG	GaCgaAGGTgGACTA--GtCAGG	-	5	2	3	0.00	1.50
Chr5:70429653-70429675	70429660	5	RNA	GGCCGAGGTCGACTACCGGCNRG	GGCCGAGGgtGcCT-CgGGCTGG	-	4	1	3	0.00	1.50
Chr1:119107875-119107897	119107892	3	RNA	GGCCGAGGTCGACTACCGGCNRG	G-aCGgGGTCGACgACgaGCGGG	+	5	1	3	0.00	1.50
Chr4:187429626-187429648	187429643	3	RNA	GGCCGAGGTCGACTACCGGCNRG	G--CGAGGTCGACgACgcGaTGG	+	4	2	3	0.00	1.50
Chr7:64653817-64653839	64653834	3	RNA	GGCCGAGGTCGACTACCGGCNRG	GGCgGAGGTgtgCT-CgGGCCGG	+	5	1	3	0.00	1.50
Chr8:64332994-64333016	64333001	5	RNA	GGCCGAGGTCGACTACCGGCNRG	aGCCcAGGaCG--TACaaGCTGG	-	5	2	3	0.00	1.50

Chr10:78654684-78654706	78654691	5	RNA	GGCCGAGGTCGACTACCGGCNRG	GGC-GAGGTCGAggACCttgGGG	-	5	1	3	0.00	1.50
Chr1:267012530-267012552	267012537	5	RNA	GGCCGAGGTCGACTACCGGCNRG	G-aCGgGGTCGACaACgaGCGGG	-	5	1	3	0.00	1.50
Chr10:125820073-125820095	125820080	5	DNA	GGCCGAGGTCGACTACC--GGCNRG	GGCgGAGGTCGtCggCaGAGGCCAG	-	5	2	3	0.00	1.50
Chr4:94800265-94800287	94800272	5	RNA	GGCCGAGGTCGACTACCGGCNRG	tGCgGgGcTCGACTAC-GGaCGG	-	5	1	3	0.00	1.50
Chr4:10013239-10013261	10013256	3	RNA	GGCCGAGGTCGACTACCGGCNRG	ttCCGAtGcCGAtTA--GGCAGG	+	5	2	3	0.00	1.50
Chr9:120983383-120983405	120983400	3	DNA	GGCCGAGGTCGA--CTACCGGCNRG	GGgCGAcGaCGATTCTACtaGCCAG	+	5	2	3	0.00	1.50
Chr4:130009606-130009628	130009613	5	RNA	GGCCGAGGTCGACTACCGGCNRG	GGgCGAGGTC-ACgcgCGGgCAG	-	5	1	3	0.00	1.50
Chr4:6496967-6496989	6496984	3	RNA	GGCCGAGGTCGACTACCGGCNRG	GGCgGcGGgCG--gACaGGCGGG	+	5	2	3	0.00	1.50
Chr6:36624904-36624926	36624911	5	RNA	GGCCGAGGTCGACTACCGGCNRG	tGCCGAGGTCG--TAttGctGGG	-	5	2	3	0.00	1.50
Chr5:101643973-101643995	101643980	5	RNA	GGCCGAGGTCGACTACCGGCNRG	GGC--ttGcgGACTACtGGCTAG	-	5	2	3	0.00	1.50
Chr2:222670902-222670924	222670919	3	RNA	GGCCGAGGTCGACTACCGGCNRG	G--tGcGGgCGAgTtCCGGCGGG	+	5	2	3	0.00	1.50
Chr7:21137739-21137761	21137756	3	DNA	GGCCGAGGTC-GACTACCGGCNRG	GGCtGAGGcCTGACTACCtGaGGG	+	4	1	3	0.00	1.50
Chr8:172012767-172012789	172012784	3	RNA	GGCCGAGGTCGACTACCGGCNRG	caCC-AGGTCGcCTACCtGgAGG	+	5	1	3	0.00	1.50
Chr3:54020223-54020245	54020230	5	RNA	GGCCGAGGTCGACTACCGGCNRG	tGtCG--GaCGAaTACgGGCAGG	-	5	2	3	0.00	1.50
Chr4:121470030-121470052	121470037	5	RNA	GGCCGAGGTCGACTACCGGCNRG	G-CCtcGGgCGAgTACtGGCAGG	-	5	1	3	0.00	1.50
Chr6:168809714-168809736	168809731	3	RNA	GGCCGAGGTCGACTACCGGCNRG	GGCgGAGG-CGgCTAtgGaCTGG	+	5	1	3	0.00	1.50
Chr1:42321946-42321968	42321963	3	RNA	GGCCGAGGTCGACTACCGGCNRG	tGCCGAGGTgG--TggCaGCTGG	+	5	2	3	0.00	1.50
Chr7:26196527-26196549	26196544	3	RNA	GGCCGAGGTCGACTACCGGCNRG	GaCaG-GGTCGACaACgaGCGGG	+	5	1	3	0.00	1.50
Chr3:183744311-183744333	183744318	5	RNA	GGCCGAGGTCGACTACCGGCNRG	GGCCGAGGgCG--cgCgcGCGGG	-	5	2	3	0.00	1.50
Chr1:2335196-2335218	2335213	3	RNA	GGCCGAGGTCGACTACCGGCNRG	G-aCGgGGTCGACgACgaGCGGG	+	5	1	3	0.00	1.50
Chr9:81333146-81333168	81333153	5	RNA	GGCCGAGGTCGACTACCGGCNRG	GGC--tcGcgGACTACCGGCTGG	-	4	2	3	0.00	1.50
Chr1:61110248-61110270	61110265	3	RNA	GGCCGAGGTCGACTACCGGCNRG	GGC--gGGcCaACctCCGGCCGG	+	5	2	3	0.00	1.50
Chr5:11950623-11950645	11950640	3	DNA	GG-CCGAGGTCGACTACCGGCNRG	GGTaCGAGGgCGACacCCaGCAAG	+	5	1	3	0.00	1.50
Chr2:51023454-51023476	51023461	5	RNA	GGCCGAGGTCGACTACCGGCNRG	G-CCtcGGgCGAgTACtGGCAGG	-	5	1	3	0.00	1.50
Chr6:70088222-70088244	70088239	3	RNA	GGCCGAGGTCGACTACCGGCNRG	GGCaGAaG--GACTcCgGGCCGG	+	4	2	3	0.00	1.50
Chr1:43873663-43873685	43873680	3	RNA	GGCCGAGGTCGACTACCGGCNRG	tGgCGAGGTCGAggACC--tTGG	+	5	2	3	0.00	1.50
Chr1:165704390-165704412	165704407	3	RNA	GGCCGAGGTCGACTACCGGCNRG	G-aCGgGGTCGACgACgaGCGGG	+	5	1	3	0.00	1.50
Chr5:122722957-122722979	122722964	5	DNA	GGCCGAGGTCGACTAC--CGGCNRG	GGCgGgtGTtGgCTACTACGGCTAG	-	5	2	3	0.00	1.50
Chr1:150154701-150154723	150154718	3	RNA	GGCCGAGGTCGACTACCGGCNRG	tGCaGgGcTCGACTA--GGtTGG	+	5	2	3	0.00	1.50
Chr4:95396893-95396915	95396900	5	RNA	GGCCGAGGTCGACTACCGGCNRG	GGgCGgGGTCGgCT-CtGGgAGG	-	5	1	3	0.00	1.50
Chr5:115739814-115739836	115739821	5	RNA	GGCCGAGGTCGACTACCGGCNRG	GGCtGgGcTgGACTA--GGtTGG	-	5	2	3	0.00	1.50
Chr2:164461449-164461471	164461456	5	RNA	GGCCGAGGTCGACTACCGGCNRG	GGgCGAGGgCGA--AggGGgGAG	-	5	2	3	0.00	1.50
Chr2:160086853-160086875	160086860	5	RNA	GGCCGAGGTCGACTACCGGCNRG	GGggGAGGTgG--TAgaGGCTGG	-	5	2	3	0.00	1.50
Chr5:22807892-22807914	22807899	5	RNA	GGCCGAGGTCGACTACCGGCNRG	GGCtGAacTCG-CTACCttCGGG	-	5	1	3	0.00	1.50
Chr9:35485798-35485820	35485805	5	RNA	GGCCGAGGTCGACTACCGGCNRG	GGCCGtctTCGAgTA--GtCCGG	-	5	2	3	0.00	1.50
Chr2:17754457-17754479	17754474	3	RNA	GGCCGAGGTCGACTACCGGCNRG	GtCatAGGTC--CTAtCGcCGAG	+	5	2	3	0.00	1.50
Chr9:7548854-7548876	7548861	5	RNA	GGCCGAGGTCGACTACCGGCNRG	GGtCGAGGgtGcCT-CgGGCTGG	-	5	1	3	0.00	1.50
Chr5:85959665-85959687	85959672	5	RNA	GGCCGAGGTCGACTACCGGCNRG	GcCgGAGccCGAC--CCGtCAAG	-	5	2	3	0.00	1.50
Chr1:119836610-119836632	119836617	5	RNA	GGCCGAGGTCGACTACCGGCNRG	GGC-GAGGTCGACgACgaGaTAG	-	4	1	3	0.00	1.50
Chr2:5972866-5972888	5972883	3	RNA	GGCCGAGGTCGACTACCGGCNRG	tGCCGtGGcCt--TcCCGGCCGG	+	5	2	3	0.00	1.50
Chr3:38682391-38682413	38682398	5	RNA	GGCCGAGGTCGACTACCGGCNRG	aGCCacGGTCaACTAgC--CAGG	-	5	2	3	0.00	1.50
Chr6:135776647-135776669	135776654	5	DNA	GGC--CGAGGTCGACTACCGGCNRG	GGCCTCGgGGgCtACacCCGGCGGG	-	5	2	3	0.00	1.50
Chr6:170124276-170124298	170124283	5	RNA	GGCCGAGGTCGACTACCGGCNRG	GGCgGAGcTCGgCTtC-GtCGAG	-	5	1	3	0.00	1.50
Chr1:18355817-18355839	18355834	3	RNA	GGCCGAGGTCGACTACCGGCNRG	cGCCa--GTCGtgTACCGGgAAG	+	5	2	3	0.00	1.50
Chr9:144412821-144412843	144412828	5	RNA	GGCCGAGGTCGACTACCGGCNRG	tGCCGtGGcCt--TcCCGGCCGG	-	5	2	3	0.00	1.50
Chr7:124852216-124852238	124852233	3	RNA	GGCCGAGGTCGACTACCGGCNRG	GGC--ttGcgGACTAtCGGCTGG	+	5	2	3	0.00	1.50
Chr8:149453620-149453642	149453627	5	RNA	GGCCGAGGTCGACTACCGGCNRG	GG--GAGGTCGAggACCaGgAGG	-	4	2	3	0.00	1.50
Chr5:2510066-2510088	2510083	3	RNA	GGCCGAGGTCGACTACCGGCNRG	GGC--ttGcgGACTACCGGCTGG	+	4	2	3	0.00	1.50
Chr3:5740871-5740893	5740888	3	RNA	GGCCGAGGTCGACTACCGGCNRG	caCCaAGtTCGcCT-CCGGCCAG	+	5	1	3	0.00	1.50
Chr9:113641452-113641474	113641459	5	DNA	GGCCGAGG-TCGACTACCGGCNRG	GGCtGtctTTCcACTACCGGCTGG	-	5	1	3	0.00	1.50
Chr4:176923490-176923512	176923497	5	RNA	GGCCGAGGTCGACTACCGGCNRG	GGC--AGGcCaACctCCGGCCGG	-	4	2	3	0.00	1.50
Chr4:211891563-211891585	211891580	3	DNA	GGCCGAGGTCGACTACCG-GCNRG	GGaCGAGGTCtcCTACtGAGaAGG	+	5	1	3	0.00	1.50
Chr2:124461429-124461451	124461446	3	RNA	GGCCGAGGTCGACTACCGGCNRG	GGtCGA--TCtACTcCtGGgAAG	+	5	2	3	0.00	1.50
Chr2:54091487-54091509	54091504	3	RNA	GGCCGAGGTCGACTACCGGCNRG	cGCCGAaGTCGAtcA--GtCGAG	+	5	2	3	0.00	1.50
Chr1:147825700-147825722	147825717	3	RNA	GGCCGAGGTCGACTACCGGCNRG	GGggGgGGgCGA--ACCGGtCGG	+	5	2	3	0.00	1.50
Chr10:128745627-128745649	128745634	5	RNA	GGCCGAGGTCGACTACCGGCNRG	GcCgGAGccCGAC--CCGtCAGG	-	5	2	3	0.00	1.50
Chr4:29567025-29567047	29567042	3	DNA	GGCCGAGGTCGACTACC--GGCNRG	GGCgGAGGTCGtCggCaGAGGCCAG	+	5	2	3	0.00	1.50
Chr6:132897142-132897164	132897159	3	RNA	GGCCGAGGTCGACTACCGGCNRG	GaCaG-GGTCGACgACaaGCGGG	+	5	1	3	0.00	1.50
Chr7:81881669-81881691	81881676	5	DNA	GGCCGAGGTCGACT-ACCGGCNRG	GGgCGAGaTgGtCTGACCtGCCGG	-	5	1	3	0.00	1.50
Chr2:60223890-60223912	60223897	5	RNA	GGCCGAGGTCGACTACCGGCNRG	GGCCG--GTCtACcAgCGagAGG	-	5	2	3	0.00	1.50
Chr10:112183284-112183306	112183291	5	RNA	GGCCGAGGTCGACTACCGGCNRG	aGCaGAG--CGAacgCCGGCGGG	-	5	2	3	0.00	1.50
Chr9:32186804-32186826	32186811	5	DNA	GGCCGAGGTCGACT-ACCGGCNRG	cGCCGtGGaCatCTCACCGGCGGG	-	5	1	3	0.00	1.50
Chr10:88430537-88430559	88430544	5	RNA	GGCCGAGGTCGACTACCGGCNRG	GGC--AGGcCGAgcACCaGCCAG	-	4	2	3	0.00	1.50
Chr5:195505904-195505926	195505911	5	RNA	GGCCGAGGTCGACTACCGGCNRG	GaCtGcGGcCGtCT--CGGCTGG	-	5	2	3	0.00	1.50
Chr4:186335262-186335284	186335269	5	RNA	GGCCGAGGTCGACTACCGGCNRG	GGCCctGGc--ACTACgGGaTGG	-	5	2	3	0.00	1.50
Chr7:177237341-177237363	177237358	3	RNA	GGCCGAGGTCGACTACCGGCNRG	cGCCGAGGcCGgggA--GGCCAG	+	5	2	3	0.00	1.50
Chr5:201524946-201524968	201524953	5	RNA	GGCCGAGGTCGACTACCGGCNRG	atCCtgGGT-GACcACCGGCGGG	-	5	1	3	0.00	1.50
Chr5:27832865-27832887	27832872	5	RNA	GGCCGAGGTCGACTACCGGCNRG	GGC--AGGcCaACctCCGGCCGG	-	4	2	3	0.00	1.50
Chr1:181021666-181021688	181021673	5	RNA	GGCCGAGGTCGACTACCGGCNRG	GGC--ttGcgGACTACCGGCTGG	-	4	2	3	0.00	1.50
Chr8:15550544-15550566	15550561	3	RNA	GGCCGAGGTCGACTACCGGCNRG	G-CCGAGGTCGAggtCgGGCGAG	+	4	1	3	0.00	1.50
Chr4:204248123-204248145	204248140	3	RNA	GGCCGAGGTCGACTACCGGCNRG	cGCCGActgCGACg--CGGCTAG	+	5	2	3	0.00	1.50
Chr1:28789432-28789454	28789439	5	RNA	GGCCGAGGTCGACTACCGGCNRG	GcCCtAGGgC--CcACCGGtCAG	-	5	2	3	0.00	1.50
Chr8:148618540-148618562	148618557	3	RNA	GGCCGAGGTCGACTACCGGCNRG	GtCCG--GTgcACcACCGGaCAG	+	5	2	3	0.00	1.50
Chr1:93501870-93501892	93501877	5	RNA	GGCCGAGGTCGACTACCGGCNRG	GGC--AGGcCGAgcACtGGCCGG	-	4	2	3	0.00	1.50
Chr1:102556097-102556119	102556114	3	RNA	GGCCGAGGTCGACTACCGGCNRG	GtCCG--GTgcACcACCGGaCAG	+	5	2	3	0.00	1.50
Chr6:19490954-19490976	19490961	5	RNA	GGCCGAGGTCGACTACCGGCNRG	GGaCGAGGcgcACaAC--GCTAG	-	5	2	3	0.00	1.50
Chr1:4946356-4946378	4946363	5	RNA	GGCCGAGGTCGACTACCGGCNRG	GaCCGAGG-CGACgACaaGaAGG	-	5	1	3	0.00	1.50
Chr8:135074276-135074298	135074293	3	RNA	GGCCGAGGTCGACTACCGGCNRG	GaCCGAGGT-GACgACaaGaAGG	+	5	1	3	0.00	1.50
Chr6:36494689-36494711	36494696	5	RNA	GGCCGAGGTCGACTACCGGCNRG	GGCaGctGTC--CTtCCGcCAGG	-	5	2	3	0.00	1.50
Chr8:103269032-103269054	103269049	3	RNA	GGCCGAGGTCGACTACCGGCNRG	GcCaGcGGTCGA--AagGGCGGG	+	5	2	3	0.00	1.50
Chr8:53054923-53054945	53054940	3	RNA	GGCCGAGGTCGACTACCGGCNRG	G-aCGgGGTCGACgACgaGCGGG	+	5	1	3	0.00	1.50
Chr5:42636727-42636749	42636744	3	RNA	GGCCGAGGTCGACTACCGGCNRG	ctCCaAGGTCGtC-gCCGGCCAG	+	5	1	3	0.00	1.50
Chr1:190416490-190416512	190416497	5	RNA	GGCCGAGGTCGACTACCGGCNRG	GGC--tcGcgGACTACCGaCTGG	-	5	2	3	0.00	1.50
Chr7:65705760-65705782	65705767	5	RNA	GGCCGAGGTCGACTACCGGCNRG	GGC--tcGctGACTACCGtCTGG	-	5	2	3	0.00	1.50
Chr8:82885005-82885027	82885022	3	RNA	GGCCGAGGTCGACTACCGGCNRG	cGgCGAGGTtG--TggCGGCGAG	+	5	2	3	0.00	1.50
Chr1:114525209-114525231	114525216	5	RNA	GGCCGAGGTCGACTACCGGCNRG	GGCCGAtcTCG--cAtCaGCAAG	-	5	2	3	0.00	1.50
Chr8:84291806-84291828	84291813	5	RNA	GGCCGAGGTCGACTACCGGCNRG	GGCgcAacTCtACT-CCGGCTAG	-	5	1	3	0.00	1.50
Chr1:56666526-56666548	56666533	5	DNA	GGC-CGAGGTCGACTACCGGCNRG	GcCTCGgGGaCGACTtCCGGtTGG	-	5	1	3	0.00	1.50
Chr5:62925596-62925618	62925603	5	X	GGCCGAGGTCGACTACCGGCNRG	GcCCGAGGTCaACctCgGGCGGG	-	5	0	3	0.00	1.50
Chr2:12763024-12763046	12763041	3	RNA	GGCCGAGGTCGACTACCGGCNRG	GGtCcAGGgC--CTtCCtGCGAG	+	5	2	3	0.00	1.50
Chr6:172063662-172063684	172063669	5	DNA	GGC-CGAGGTCGACTACCGGCNRG	GcCTCGgGGaCGtCTtCCGGCTGG	-	5	1	3	0.00	1.50
Chr1:190593432-190593454	190593449	3	RNA	GGCCGAGGTCGACTACCGGCNRG	ttCCGAtGTCGAtTA--tGCCGG	+	5	2	3	0.00	1.50
Chr2:54183719-54183741	54183736	3	DNA	GGC-CGAGGTCGACTACCGGCNRG	GcCTCGgGGaCGtCTtCCGGCTGG	+	5	1	3	0.00	1.50
Chr1:266876252-266876274	266876269	3	RNA	GGCCGAGGTCGACTACCGGCNRG	G-aCGgGGTCGACgACgaGCGGG	+	5	1	3	0.00	1.50
Chr5:162335113-162335135	162335120	5	DNA	GGCCGAGGTCGACTACCGG--CNRG	GGtCGAGGTCGAgTcCgaGCCCCGG	-	5	2	3	0.00	1.50
Chr4:96949999-96950021	96950016	3	RNA	GGCCGAGGTCGACTACCGGCNRG	tGCgcAGGTCGACatCC--CTGG	+	5	2	3	0.00	1.50
Chr2:11257790-11257812	11257807	3	RNA	GGCCGAGGTCGACTACCGGCNRG	GcCCGAGGcCGA--ACttGgTGG	+	5	2	3	0.00	1.50
Chr5:188062070-188062092	188062087	3	RNA	GGCCGAGGTCGACTACCGGCNRG	GGaCtAGGTCG--TcCCaGtTGG	+	5	2	3	0.00	1.50
Chr5:27782968-27782990	27782975	5	RNA	GGCCGAGGTCGACTACCGGCNRG	GGgCG-GGcCaACctCCGGCCGG	-	5	1	3	0.00	1.50
Chr2:124722901-124722923	124722918	3	RNA	GGCCGAGGTCGACTACCGGCNRG	aGCCGAtcTCcAC--CCaGCCAG	+	5	2	3	0.00	1.50
Chr9:105929883-105929905	105929890	5	RNA	GGCCGAGGTCGACTACCGGCNRG	GGaCtAGGTCG--TcCCaGtTGG	-	5	2	3	0.00	1.50
Chr8:42910028-42910050	42910045	3	RNA	GGCCGAGGTCGACTACCGGCNRG	cG--GtGGcCGACTctCGGCTGG	+	5	2	3	0.00	1.50
Chr2:75928676-75928698	75928693	3	RNA	GGCCGAGGTCGACTACCGGCNRG	GtCCGAGGTC--CTtagGaCAAG	+	5	2	3	0.00	1.50
Chr10:26275850-26275872	26275857	5	RNA	GGCCGAGGTCGACTACCGGCNRG	GGCtcAcG--GACTACCGtCTGG	-	4	2	3	0.00	1.50
Chr2:109061746-109061768	109061763	3	RNA	GGCCGAGGTCGACTACCGGCNRG	GGC--AGcctGACgACCGtCTGG	+	5	2	3	0.00	1.50
Chr4:239222933-239222955	239222950	3	RNA	GGCCGAGGTCGACTACCGGCNRG	GGC--tcGcgGACTACCGGCTGG	+	4	2	3	0.00	1.50
Chr5:82742673-82742695	82742690	3	DNA	GGC-CGAGGTCGACTACCGGCNRG	GcCTCGAGGgCtACacCCGGCGGG	+	5	1	3	0.00	1.50
Chr3:52847749-52847771	52847766	3	RNA	GGCCGAGGTCGACTACCGGCNRG	GGCCatGGctG--TAaCGGCCGG	+	5	2	3	0.00	1.50
Chr2:209578199-209578221	209578216	3	RNA	GGCCGAGGTCGACTACCGGCNRG	GGaCcAGGTCG--TcCCaGtTGG	+	5	2	3	0.00	1.50
Chr6:120885104-120885126	120885121	3	RNA	GGCCGAGGTCGACTACCGGCNRG	aGgCGAGcTCGAgTA--GGgAAG	+	5	2	3	0.00	1.50
Chr3:133752817-133752839	133752834	3	RNA	GGCCGAGGTCGACTACCGGCNRG	aGC--AGGcCaACTACCGctTGG	+	5	2	3	0.00	1.50
Chr5:141772940-141772962	141772957	3	RNA	GGCCGAGGTCGACTACCGGCNRG	tGaCcAGGTCGAC--CCaGtCGG	+	5	2	3	0.00	1.50
Chr4:82715624-82715646	82715631	5	DNA	GGC--CGAGGTCGACTACCGGCNRG	GcCAACGcGGTCGAggACtGGCGGG	-	5	2	3	0.00	1.50
Chr7:24225185-24225207	24225202	3	DNA	GGCCGAGGTCGA--CTACCGGCNRG	GcgCcAtcTCGATTCTACCGGCGAG	+	5	2	3	0.00	1.50
Chr4:92024077-92024099	92024084	5	RNA	GGCCGAGGTCGACTACCGGCNRG	GGCgcAcaTCGACT-CtGGCAAG	-	5	1	3	0.00	1.50
Chr5:10078589-10078611	10078596	5	DNA	GGC--CGAGGTCGACTACCGGCNRG	GaCAACGgGGTCGACgACgaGCGGG	-	5	2	3	0.00	1.50
Chr6:66953869-66953891	66953876	5	RNA	GGCCGAGGTCGACTACCGGCNRG	GGCCcAcGaCG--TttCGGCTGG	-	5	2	3	0.00	1.50
Chr3:65067584-65067606	65067591	5	RNA	GGCCGAGGTCGACTACCGGCNRG	G-aCGgGGTCGACaACgaGCGGG	-	5	1	3	0.00	1.50
Chr2:8301729-8301751	8301746	3	DNA	GGCC-GAGGTCGACTACCGGCNRG	GGCCAGAGcgtGAtTAaCGGCTGG	+	5	1	3	0.00	1.50
Chr4:25469952-25469974	25469969	3	RNA	GGCCGAGGTCGACTACCGGCNRG	G-aCGgGGTCGACaACgaGCGGG	+	5	1	3	0.00	1.50
Chr5:116664173-116664195	116664190	3	RNA	GGCCGAGGTCGACTACCGGCNRG	tGCCcAGG--GACaAgCGGgCAG	+	5	2	3	0.00	1.50
Chr1:210041938-210041960	210041945	5	RNA	GGCCGAGGTCGACTACCGGCNRG	GGCgGAGGTaGcCcA--GGCCAG	-	4	2	3	0.00	1.50
Chr3:187183718-187183740	187183725	5	RNA	GGCCGAGGTCGACTACCGGCNRG	GGCCtAcGgC--CTACaGGgCGG	-	5	2	3	0.00	1.50
Chr9:132756515-132756537	132756532	3	RNA	GGCCGAGGTCGACTACCGGCNRG	GGtCGAcGgCGACaACa--CGAG	+	5	2	3	0.00	1.50
Chr2:154133748-154133770	154133765	3	RNA	GGCCGAGGTCGACTACCGGCNRG	GGC--tcGcgGACTACCGtCTGG	+	5	2	3	0.00	1.50
Chr4:61672270-61672292	61672287	3	RNA	GGCCGAGGTCGACTACCGGCNRG	caCCGAGGTCGtCTtCaG-CAGG	+	5	1	3	0.00	1.50

Chr1:101957580-101957602	101957587	5	RNA	GGCCGAGGTCGACTACCGGCNRG	GGgCGAGGcgGACcACC--gTGG	-	5	2	3	0.00	1.50
Chr10:57906985-57907007	57907002	3	RNA	GGCCGAGGTCGACTACCGGCNRG	GGCCtcGGaCGgCgAC--GCAGG	+	5	2	3	0.00	1.50
Chr2:197149932-197149954	197149949	3	RNA	GGCCGAGGTCGACTACCGGCNRG	GGC--tcGcgGACTACCGGCTGG	+	4	2	3	0.00	1.50
Chr1:214293557-214293579	214293564	5	DNA	GG-CCGAGGTCGACTACCGGCNRG	GGTaCGAGGgCGACgtCCtGCAAG	-	5	1	3	0.00	1.50
Chr4:77977161-77977183	77977178	3	RNA	GGCCGAGGTCGACTACCGGCNRG	GGgaGAG--aGAgTAaCGGCTGG	+	5	2	3	0.00	1.50
Chr3:215419943-215419965	215419950	5	RNA	GGCCGAGGTCGACTACCGGCNRG	GGCCG-GtaCGAaTcaCGGCAGG	-	5	1	3	0.00	1.50
Chr1:99480813-99480835	99480820	5	RNA	GGCCGAGGTCGACTACCGGCNRG	GGCCGtGtTCatCTgCC--CAAG	-	5	2	3	0.00	1.50
Chr5:214299556-214299578	214299563	5	RNA	GGCCGAGGTCGACTACCGGCNRG	GaaaGAGGTCGtCT-CCGGaAAG	-	5	1	3	0.00	1.50
Chr7:13713100-13713122	13713107	5	RNA	GGCCGAGGTCGACTACCGGCNRG	aaCCcAGGT--ACcACCaGCCAG	-	5	2	3	0.00	1.50
Chr1:214344093-214344115	214344109	4	RNA	GGCCGAGGTCGACTACCGGCNRG	tG--GcGGcCGACaACCcGCCAG	+	5	2	0	2.75	1.38
Chr3:150252466-150252488	150252471	3	RNA	GGCCGAGGTCGACTACCGGCNRG	tGCCGA--TacACcgCCGGCCGG	-	5	2	0	2.75	1.38
Chr8:151972397-151972419	151972404	5	DNA	GGCCGAGGTCGACTAC-CGGCNRG	GGgCGAGGcCGgCggCGCGGCGGG	-	5	1	0	2.75	1.38
Chr3:102002994-102003016	102003010	4	RNA	GGCCGAGGTCGACTACCGGCNRG	G-aCGAGGTCaACaACgaGCGGG	+	5	1	0	2.75	1.38
Chr10:105202045-105202067	105202063	2	RNA	GGCCGAGGTCGACTACCGGCNRG	GcgCGcGG--GgCTgCCGGCCGG	+	5	2	0	2.75	1.38
Chr1:90735162-90735184	90735169	5	RNA	GGCCGAGGTCGACTACCGGCNRG	GGgCGAGcTCGtC-ggCGGCGAG	-	5	1	0	2.75	1.38
Chr8:38663694-38663716	38663709	5	RNA	GGCCGAGGTCGACTACCGGCNRG	cGCCGtGGTCGcCg--CGGCAGG	+	4	2	0	2.75	1.38
Chr5:143935928-143935950	143935935	5	RNA	GGCCGAGGTCGACTACCGGCNRG	GGC--AGGcCaACcgCCGGCCAG	-	4	2	0	2.75	1.38
Chr6:124161874-124161896	124161892	2	DNA	GG-CCGAGGTCGACTACCGGCNRG	GGTtCGAGGgCGACgtCCtGCAGG	+	5	1	0	2.75	1.38
Chr3:209532291-209532313	209532309	2	X	GGCCGAGGTCGACTACCGGCNRG	GGCCGAGcTCacCTcCCtGCTAG	+	5	0	0	2.75	1.38
Chr9:154838347-154838369	154838353	4	RNA	GGCCGAGGTCGACTACCGGCNRG	GGCCGAGGctGgCg--CGGgTGG	-	5	2	0	2.75	1.38
Chr4:243949172-243949194	243949179	5	RNA	GGCCGAGGTCGACTACCGGCNRG	GGCCG-GGcCatCcACtGGCGGG	-	5	1	0	2.75	1.38
Chr7:70311146-70311168	70311164	2	DNA	GGCCGAGGTCGACTA-CCGGCNRG	GGCCGgGGTaatCaATCCGGCGAG	+	5	1	0	2.75	1.38
Chr2:2866583-2866605	2866587	2	RNA	GGCCGAGGTCGACTACCGGCNRG	GGCCGgaGgtGACTtC-GGCGAG	-	5	1	0	2.75	1.38
Chr1:179744933-179744955	179744938	3	DNA	GGCCGAGGTCGAC--TACCGGCNRG	cGgCGccGTCGACGGTACCGcCCGG	-	5	2	0	2.75	1.38
Chr7:9428967-9428989	9428985	2	DNA	GGCCGAGGTCGACTACCGG--CNRG	GGCtGAGGgCGAtTgCCtGCTCCAG	+	5	2	0	2.75	1.38
Chr1:286042775-286042797	286042791	4	DNA	G--GCCGAGGTCGACTACCGGCNRG	GCCGCCGcGcTCGACTgtCGGtTGG	+	5	2	0	2.75	1.38
Chr5:206571495-206571517	206571501	4	RNA	GGCCGAGGTCGACTACCGGCNRG	GGCCGAGtaCG--TtgCcGCAGG	-	5	2	0	2.75	1.38
Chr5:108783657-108783679	108783673	4	RNA	GGCCGAGGTCGACTACCGGCNRG	GGtCaAttTCGACTtC--GCTGG	+	5	2	0	2.75	1.38
Chr7:133960443-133960465	133960461	2	RNA	GGCCGAGGTCGACTACCGGCNRG	GGCCGgGGcgGgC--CgGGCCGG	+	5	2	0	2.75	1.38
Chr7:117839999-117840021	117840017	2	DNA	GGCCGAGGTCGACTACCGG-CNRG	GGgaGAGGgCGAtcACCGGCCTAG	+	5	1	0	2.75	1.38
Chr5:182293559-182293581	182293577	2	RNA	GGCCGAGGTCGACTACCGGCNRG	G--CGAcGTCGACTACCacgAGG	+	4	2	0	2.75	1.38
Chr4:78527600-78527622	78527618	2	RNA	GGCCGAGGTCGACTACCGGCNRG	cGaCGgaGTC--CcACCGGCGAG	+	5	2	0	2.75	1.38
Chr7:111091542-111091564	111091547	3	RNA	GGCCGAGGTCGACTACCGGCNRG	aaCCGAGGTCGcCT-CaGGCTGG	-	4	1	0	2.75	1.38
Chr7:88825173-88825195	88825179	4	RNA	GGCCGAGGTCGACTACCGGCNRG	GGatGAGGTCGcgTcCC--CTAG	-	5	2	0	2.75	1.38
Chr9:29556821-29556843	29556836	5	RNA	GGCCGAGGTCGACTACCGGCNRG	GGCCG-GtaCGAaTcaCGGCAGG	+	5	1	0	2.75	1.38
Chr4:151727579-151727601	151727597	2	RNA	GGCCGAGGTCGACTACCGGCNRG	cGaCGAGccCGACT-CCGGtTGG	+	5	1	0	2.75	1.38
Chr4:20359371-20359393	20359376	3	RNA	GGCCGAGGTCGACTACCGGCNRG	GGC--gGGcCaACccCCGGCCGG	-	5	2	0	2.75	1.38
Chr5:65989555-65989577	65989560	3	RNA	GGCCGAGGTCGACTACCGGCNRG	GtCCG--GTgcACcACCGGaCAG	-	5	2	0	2.75	1.38
Chr8:56771348-56771370	56771366	2	RNA	GGCCGAGGTCGACTACCGGCNRG	GGCCatGGcCGAgTtC--GCCAG	+	5	2	0	2.75	1.38
Chr8:151877558-151877580	151877576	2	DNA	GGCCGAG--GTCGACTACCGGCNRG	GctCGAGCGGgaGACgACCGGCTGG	+	5	2	0	2.75	1.38
Chr3:29061998-29062020	29062016	2	RNA	GGCCGAGGTCGACTACCGGCNRG	GGCgGActTtGACaAC--GCGGG	+	5	2	0	2.75	1.38
Chr1:41943708-41943730	41943724	4	RNA	GGCCGAGGTCGACTACCGGCNRG	GGCCaAttTCGcCTtC--GCCGG	+	5	2	0	2.75	1.38
Chr8:138785201-138785223	138785219	2	RNA	GGCCGAGGTCGACTACCGGCNRG	GaCaGAGcT-GACaACCGGaGAG	+	5	1	0	2.75	1.38
Chr5:20511163-20511185	20511180	3	RNA	GGCCGAGGTCGACTACCGGCNRG	GGtCGAtGagGACcACC--CCAG	+	5	2	0	2.75	1.38
Chr6:129542768-129542790	129542783	5	RNA	GGCCGAGGTCGACTACCGGCNRG	GGgCGAGGgCGA-gAatGGCAGG	+	5	1	0	2.75	1.38
Chr2:183741875-183741897	183741893	2	RNA	GGCCGAGGTCGACTACCGGCNRG	a--CGgGGTCGACaACgaGCGGG	+	5	2	0	2.75	1.38
Chr6:10901384-10901406	10901399	5	RNA	GGCCGAGGTCGACTACCGGCNRG	GGCtGgGcTgGACTA--GGtTGG	+	5	2	0	2.75	1.38
Chr7:53803649-53803671	53803664	5	RNA	GGCCGAGGTCGACTACCGGCNRG	GGC--ttGcgGAtTACCGGCTGG	+	5	2	0	2.75	1.38
Chr4:127578360-127578382	127578377	3	X	GGCCGAGGTCGACTACCGGCNRG	GGaaGAGGTgGcCTAgCGGCTGG	+	5	0	0	2.75	1.38
Chr6:157417855-157417877	157417871	4	DNA	G--GCCGAGGTCGACTACCGGCNRG	GCCGCCGtGGTCGcCTtCgcGCTGG	+	5	2	0	2.75	1.38
Chr3:43512456-43512478	43512474	2	RNA	GGCCGAGGTCGACTACCGGCNRG	GG--GAaGggGACcAgCGGCTGG	+	5	2	0	2.75	1.38
Chr2:186668118-186668140	186668136	2	RNA	GGCCGAGGTCGACTACCGGCNRG	GGC--tcGcgGACTACtGGCTGG	+	5	2	0	2.75	1.38
Chr1:166206945-166206967	166206950	3	RNA	GGCCGAGGTCGACTACCGGCNRG	G-aCGgGGTCGACaACgaGCGGG	-	5	1	0	2.75	1.38
Chr1:205588764-205588786	205588771	5	RNA	GGCCGAGGTCGACTACCGGCNRG	aGCCGAGGgtGcCT-CgGGCTGG	-	5	1	0	2.75	1.38
Chr3:115593458-115593480	115593463	3	RNA	GGCCGAGGTCGACTACCGGCNRG	cGCCG-GGcCGAgctCCGGCGAG	-	5	1	0	2.75	1.38
Chr8:119783066-119783088	119783071	3	RNA	GGCCGAGGTCGACTACCGGCNRG	GtC--tccTtGACTACCGGCCGG	-	5	2	0	2.75	1.38
Chr6:170578096-170578118	170578112	4	RNA	GGCCGAGGTCGACTACCGGCNRG	G-aCGAGGTCGACaACgaGCGGG	+	4	1	0	2.75	1.38
Chr1:56665462-56665484	56665477	5	RNA	GGCCGAGGTCGACTACCGGCNRG	GGgC-AGGcCGAgcACCaGCCGG	+	5	1	0	2.75	1.38
Chr6:5030952-5030974	5030970	2	RNA	GGCCGAGGTCGACTACCGGCNRG	GaCCGAGGaCaAgg--CGGCAAG	+	5	2	0	2.75	1.38
Chr8:133099564-133099586	133099581	3	DNA	GGCCGAGGTCGACTACC--GGCNRG	GGCgGAGaTCtcCTcCCGAGGCCGG	+	5	2	0	2.75	1.38
Chr5:24782159-24782181	24782174	5	RNA	GGCCGAGGTCGACTACCGGCNRG	GGC--tcGcgGACTACCGtCTGG	+	5	2	0	2.75	1.38
Chr2:152935761-152935783	152935766	3	RNA	GGCCGAGGTCGACTACCGGCNRG	aGC--AGcgCGACTtCCGGgAGG	-	5	2	0	2.75	1.38
Chr4:13285912-13285934	13285917	3	RNA	GGCCGAGGTCGACTACCGGCNRG	G-aCGgGGTCGACaACgaGCGGG	-	5	1	0	2.75	1.38
Chr7:171631366-171631388	171631381	5	RNA	GGCCGAGGTCGACTACCGGCNRG	GcgaGAGGTCGAt--CCGGaTAG	+	5	2	0	2.75	1.38
Chr5:129045322-129045344	129045327	3	RNA	GGCCGAGGTCGACTACCGGCNRG	GGgCGcGGTCGAggACaG-CGGG	-	5	1	0	2.75	1.38
Chr8:99323920-99323942	99323936	4	RNA	GGCCGAGGTCGACTACCGGCNRG	aaCCGAGGaCGgCT-CgGGCTGG	+	5	1	0	2.75	1.38
Chr2:44189994-44190016	44190000	4	RNA	GGCCGAGGTCGACTACCGGCNRG	GGgCGgGGTtGgCT-CCGGgAGG	-	5	1	0	2.75	1.38
Chr8:101358178-101358200	101358195	3	RNA	GGCCGAGGTCGACTACCGGCNRG	GcCtGAGtcCGAC--CCGtCAGG	+	5	2	0	2.75	1.38
Chr6:44061726-44061748	44061743	3	RNA	GGCCGAGGTCGACTACCGGCNRG	GGC-GAGGTCaACcACgGcgTAG	+	5	1	0	2.75	1.38
Chr8:140926581-140926603	140926598	3	RNA	GGCCGAGGTCGACTACCGGCNRG	GGCaGAGGaC--CTAgaGGgGGG	+	5	2	0	2.75	1.38
Chr1:35686356-35686378	35686372	4	RNA	GGCCGAGGTCGACTACCGGCNRG	GGaCGAGG-CGcCcACCGcCCGG	+	4	1	0	2.75	1.38
Chr1:251452902-251452924	251452919	3	DNA	GGCCGAGGTCG-ACTACCGGCNRG	GGCCGAacaCGAACcgCCGGCTGG	+	5	1	0	2.75	1.38
Chr5:153755342-153755364	153755360	2	RNA	GGCCGAGGTCGACTACCGGCNRG	GGCgGAGGTCG--TtCgGcaGAG	+	5	2	0	2.75	1.38
Chr4:71622045-71622067	71622051	4	RNA	GGCCGAGGTCGACTACCGGCNRG	GaCCGAGG-CGACgACaaGaAGG	-	5	1	0	2.75	1.38
Chr5:14166142-14166164	14166160	2	RNA	GGCCGAGGTCGACTACCGGCNRG	GG--GAGGTCGgCgcCaGGaTAG	+	5	2	0	2.75	1.38
Chr10:129625891-129625913	129625897	4	RNA	GGCCGAGGTCGACTACCGGCNRG	GGaCGAcccCGACT-CCGGtTGG	-	5	1	0	2.75	1.38
Chr7:26792873-26792895	26792889	4	RNA	GGCCGAGGTCGACTACCGGCNRG	GGC--AGGgCGAgcACCaGaAGG	+	5	2	0	2.75	1.38
Chr1:138278932-138278954	138278947	5	RNA	GGCCGAGGTCGACTACCGGCNRG	cGCCGccGTCGgCcACCG-CCGG	+	5	1	0	2.75	1.38
Chr2:22260813-22260835	22260828	5	RNA	GGCCGAGGTCGACTACCGGCNRG	GGC--tcGcgGACTACCGGCTGG	+	4	2	0	2.75	1.38
Chr2:182782102-182782124	182782117	5	RNA	GGCCGAGGTCGACTACCGGCNRG	GcCgGAGGcCGACTt--GGaGGG	+	5	2	0	2.75	1.38
Chr2:149090399-149090421	149090417	2	RNA	GGCCGAGGTCGACTACCGGCNRG	GGC--tcGcgGACTACCGaCTGG	+	5	2	0	2.75	1.38
Chr3:810172-810194	810177	3	RNA	GGCCGAGGTCGACTACCGGCNRG	cGtCGA--TCaACTcCaGGCCAG	-	5	2	0	2.75	1.38
Chr1:238778460-238778482	238778466	4	RNA	GGCCGAGGTCGACTACCGGCNRG	cGaCGAGtTCtAtTACC--CGAG	-	5	2	0	2.75	1.38
Chr5:174122720-174122742	174122737	3	RNA	GGCCGAGGTCGACTACCGGCNRG	GGCgGAGGTCG--TtCgGcaGAG	+	5	2	0	2.75	1.38
Chr6:45449881-45449903	45449888	5	RNA	GGCCGAGGTCGACTACCGGCNRG	GGC-GAaaTCGACcgCCGGtGAG	-	5	1	0	2.75	1.38
Chr8:11223615-11223637	11223622	5	RNA	GGCCGAGGTCGACTACCGGCNRG	tGCCGAGGatGcCT-CgGGCTGG	-	5	1	0	2.75	1.38
Chr2:226206648-226206670	226206655	5	RNA	GGCCGAGGTCGACTACCGGCNRG	GtgCGgGGTtGgCT--CGGCCGG	-	5	2	0	2.75	1.38
Chr1:12003580-12003602	12003595	5	RNA	GGCCGAGGTCGACTACCGGCNRG	GGtCG--GTCtACTcCtGGgAAG	+	5	2	0	2.75	1.38
Chr9:124681722-124681744	124681727	3	RNA	GGCCGAGGTCGACTACCGGCNRG	GGCtcAGGTaGAagA--GGCAAG	-	5	2	0	2.75	1.38
Chr4:18429693-18429715	18429697	2	RNA	GGCCGAGGTCGACTACCGGCNRG	G-CaGAGGTCaAaTACaGGaCAG	-	5	1	0	2.75	1.38
Chr1:254308288-254308310	254308303	5	RNA	GGCCGAGGTCGACTACCGGCNRG	GGCCcAtGaCG--TttCGGCCGG	+	5	2	0	2.75	1.38
Chr6:171867766-171867788	171867781	5	RNA	GGCCGAGGTCGACTACCGGCNRG	GGaC-AGGTCGAggAaCGcCAGG	+	5	1	0	2.75	1.38
Chr2:51291025-51291047	51291030	3	RNA	GGCCGAGGTCGACTACCGGCNRG	G-aCGgGGTCGACgACgaGCGGG	-	5	1	0	2.75	1.38
Chr1:173336304-173336326	173336322	2	RNA	GGCCGAGGTCGACTACCGGCNRG	GGCgGAGGcCGAga--CGGtGAG	+	5	2	0	2.75	1.38
Chr8:169947930-169947952	169947946	4	RNA	GGCCGAGGTCGACTACCGGCNRG	GGCCt-GGTgGgCgAgCGGCAGG	+	5	1	0	2.75	1.38
Chr1:234197394-234197416	234197399	3	RNA	GGCCGAGGTCGACTACCGGCNRG	G-aCGgGGTCGACaACgaGCGGG	-	5	1	0	2.75	1.38
Chr4:164613100-164613122	164613106	4	RNA	GGCCGAGGTCGACTACCGGCNRG	GtCCtcGG--GAtaACCGGCAGG	-	5	2	0	2.75	1.38
Chr2:64910493-64910515	64910508	5	RNA	GGCCGAGGTCGACTACCGGCNRG	GGCCG-GGTCGtCcACtGGCGGG	+	3	1	0	2.75	1.38
Chr10:114264179-114264201	114264194	5	RNA	GGCCGAGGTCGACTACCGGCNRG	GGC--tcGcgGACTACCGGCTGG	+	4	2	0	2.75	1.38
Chr10:65497996-65498018	65498003	5	RNA	GGCCGAGGTCGACTACCGGCNRG	GtCCG--GTgcACcACCGGaCAG	-	5	2	0	2.75	1.38
Chr3:80245059-80245081	80245064	3	RNA	GGCCGAGGTCGACTACCGGCNRG	ttCCGAGGTC-ACTAaaGtCAGG	-	5	1	0	2.75	1.38
Chr4:142823194-142823216	142823212	2	RNA	GGCCGAGGTCGACTACCGGCNRG	cG--GAGcTCGAgcACgGGCGGG	+	5	2	0	2.75	1.38
Chr1:182443334-182443356	182443339	3	RNA	GGCCGAGGTCGACTACCGGCNRG	G--CGAGGgCGAgaACgGGaGAG	-	5	2	0	2.75	1.38
Chr7:57779610-57779632	57779628	2	RNA	GGCCGAGGTCGACTACCGGCNRG	GGgCcgGGcCGgCTA--GGCTGG	+	5	2	0	2.75	1.38
Chr5:130328571-130328593	130328586	5	RNA	GGCCGAGGTCGACTACCGGCNRG	GGCCGAGG--GAtggCCcaCGAG	+	5	2	0	2.75	1.38
Chr1:99262878-99262900	99262884	4	RNA	GGCCGAGGTCGACTACCGGCNRG	GcCaGtGGaCGAC--CCGGCCAG	-	4	2	0	2.75	1.38
Chr1:22343569-22343591	22343587	2	DNA	GGC-CGAGGTCGACTACCGGCNRG	GcCTCGgGGaCGtCTtCCGGCTGG	+	5	1	0	2.75	1.38
Chr2:124769964-124769986	124769982	2	DNA	GGCCGAGGTCGACTA-CCGGCNRG	GGgCGAGacgGcCTAACCGGCCGG	+	5	1	0	2.75	1.38
Chr9:107832983-107833005	107832999	4	RNA	GGCCGAGGTCGACTACCGGCNRG	G-CaGgGGgCGAggACCGGCTGG	+	5	1	0	2.75	1.38
Chr2:41740076-41740098	41740081	3	RNA	GGCCGAGGTCGACTACCGGCNRG	GGC--ttGcgGACTACCGGCTGG	-	4	2	0	2.75	1.38
Chr2:177862013-177862035	177862030	3	RNA	GGCCGAGGTCGACTACCGGCNRG	GcCCtAGGTgG--TggCGGCTGG	+	5	2	0	2.75	1.38
Chr1:240236903-240236925	240236919	4	RNA	GGCCGAGGTCGACTACCGGCNRG	GGCCGcGGcCGACat--GtCCAG	+	5	2	0	2.75	1.38
Chr10:87827845-87827867	87827860	5	RNA	GGCCGAGGTCGACTACCGGCNRG	GGC--tcGcaGACTACCGGCTGG	+	4	2	0	2.75	1.38
Chr8:72382689-72382711	72382704	5	RNA	GGCCGAGGTCGACTACCGGCNRG	G-CCtcGGgCGAaTACtGGCAGG	+	5	1	0	2.75	1.38
Chr1:218293233-218293255	218293251	2	RNA	GGCCGAGGTCGACTACCGGCNRG	GGgCGAGcTtGggTA--GGCAGG	+	5	2	0	2.75	1.38
Chr3:196423753-196423775	196423758	3	RNA	GGCCGAGGTCGACTACCGGCNRG	G-aCGgGGTCGACgACgaGCGGG	-	5	1	0	2.75	1.38
Chr3:141716132-141716154	141716148	4	DNA	GGCCGAGGTCGA--CTACCGGCNRG	GGCCGAGGcCGAGGCTtggGGtCGG	+	5	2	0	2.75	1.38
Chr3:205618639-205618661	205618657	2	RNA	GGCCGAGGTCGACTACCGGCNRG	GG--GcGGTCGtggACCGGCGAG	+	4	2	0	2.75	1.38
Chr7:153120997-153121019	153121002	3	RNA	GGCCGAGGTCGACTACCGGCNRG	GtCttgGGT-GACcACCGGCGGG	-	5	1	0	2.75	1.38
Chr9:70838213-70838235	70838218	3	RNA	GGCCGAGGTCGACTACCGGCNRG	GGCCGAGGTCGAggAC-GtCAGG	-	3	1	0	2.75	1.38
Chr4:111121788-111121810	111121793	3	RNA	GGCCGAGGTCGACTACCGGCNRG	GG--GAaGTgGgCTgCCGGgGAG	-	5	2	0	2.75	1.38
Chr9:80534347-80534369	80534353	4	RNA	GGCCGAGGTCGACTACCGGCNRG	GGC--AGGcCGAgctCCGGtCGG	-	5	2	0	2.75	1.38
Chr4:1224057-1224079	1224075	2	RNA	GGCCGAGGTCGACTACCGGCNRG	GGCCGAGtaCG--TtgCcGCAGG	+	5	2	0	2.75	1.38
Chr5:71490334-71490356	71490340	4	RNA	GGCCGAGGTCGACTACCGGCNRG	GtCCG--GTgcACcACCGGaTAG	-	5	2	0	2.75	1.38
Chr7:142493491-142493513	142493496	3	DNA	GGCCGAGGTCGACT-ACCGGCNRG	aaCCtAGGTCcgCTCACCGGCAGG	-	5	1	0	2.75	1.38
Chr2:139404628-139404650	139404644	4	RNA	GGCCGAGGTCGACTACCGGCNRG	GaCCG-GaTCGACaACgaGCGGG	+	5	1	0	2.75	1.38

Chr10:75795316-75795338	75795333	3	RNA	GGCCGAGGTCGACTACCGGCNRG	GcCgGAGccCGAC--CCGtCAAG	+	5	2	0	2.75	1.38
Chr5:133413000-133413022	133413006	4	RNA	GGCCGAGGTCGACTACCGGCNRG	GGtgGAGGgCaACTAtC--CCGG	-	5	2	0	2.75	1.38
Chr4:243565738-243565760	243565742	2	RNA	GGCCGAGGTCGACTACCGGCNRG	GGCCGgaGgtGACTtC-GGCGAG	-	5	1	0	2.75	1.38
Chr8:38783048-38783070	38783064	4	RNA	GGCCGAGGTCGACTACCGGCNRG	GtgCGAtG-CGgCTgCCGGCCGG	+	5	1	0	2.75	1.38
Chr9:133812211-133812233	133812227	4	RNA	GGCCGAGGTCGACTACCGGCNRG	G-aCGgGGTCGACaACgaGCGGG	+	5	1	0	2.75	1.38
Chr2:16595188-16595210	16595203	5	RNA	GGCCGAGGTCGACTACCGGCNRG	GGCtcAcG--GACTAttGGCTGG	+	5	2	0	2.75	1.38
Chr7:64593047-64593069	64593064	3	RNA	GGCCGAGGTCGACTACCGGCNRG	GcCCaAGccCGACTtC--GCCAG	+	5	2	0	2.75	1.38
Chr2:50610977-50610999	50610995	2	RNA	GGCCGAGGTCGACTACCGGCNRG	GGtCGAGGTCG--TggCcGCGGG	+	4	2	0	2.75	1.38
Chr5:171048843-171048865	171048848	3	RNA	GGCCGAGGTCGACTACCGGCNRG	GGCtcAcG--GACTACaGtCTGG	-	5	2	0	2.75	1.38
Chr3:129227775-129227797	129227782	5	RNA	GGCCGAGGTCGACTACCGGCNRG	GcaCGAG--CGAaTACaGGaTAG	-	5	2	0	2.75	1.38
Chr1:245550493-245550515	245550511	2	RNA	GGCCGAGGTCGACTACCGGCNRG	GGC--AGGcCaAgctCCGGCCGG	+	5	2	0	2.75	1.38
Chr2:45460337-45460359	45460355	2	RNA	GGCCGAGGTCGACTACCGGCNRG	GGgCGAGGaC--CgAgaGGCGAG	+	5	2	0	2.75	1.38
Chr5:222727445-222727467	222727450	3	RNA	GGCCGAGGTCGACTACCGGCNRG	GGCaGcGGTCGAC-ggCGGgCGG	-	5	1	0	2.75	1.38
Chr4:188318694-188318716	188318699	3	RNA	GGCCGAGGTCGACTACCGGCNRG	G-aCGgGGTCGACaACgaGCGGG	-	5	1	0	2.75	1.38
Chr3:201853421-201853443	201853427	4	RNA	GGCCGAGGTCGACTACCGGCNRG	aGCCctGcTCGACT--CcGCTGG	-	5	2	0	2.75	1.38
Chr8:78781894-78781916	78781900	4	X	GGCCGAGGTCGACTACCGGCNRG	GGaaGAGGTgGcCTAgCGGCTGG	-	5	0	1	1.65	1.33
Chr5:28634198-28634220	28634205	5	RNA	GGCCGAGGTCGACTACCGGCNRG	GGCtGcGcTaGcCT-CCGGCCAG	-	5	1	1	1.65	1.33
Chr3:198373903-198373925	198373919	4	RNA	GGCCGAGGTCGACTACCGGCNRG	G-aCGgGGaCGgCgACCGGCGGG	+	5	1	1	1.65	1.33
Chr3:211808730-211808752	211808748	2	RNA	GGCCGAGGTCGACTACCGGCNRG	tGgCGAGcTCa--TACCGGCCGG	+	4	2	1	1.65	1.33
Chr1:122460244-122460266	122460248	2	RNA	GGCCGAGGTCGACTACCGGCNRG	GaCCaAGGaatACT-CCGGCGAG	-	5	1	2	0.55	1.28
Chr6:20347024-20347046	20347041	3	DNA	GGC-CGAGGTCGACTACCGGCNRG	GcCTCGgGGaCGtCTtCCGGCTGG	+	5	1	2	0.55	1.28
Chr5:11477335-11477357	11477341	4	RNA	GGCCGAGGTCGACTACCGGCNRG	G-aCGgGGTCGACgACgaGCGGG	-	5	1	2	0.55	1.28
Chr1:91102570-91102592	91102576	4	RNA	GGCCGAGGTCGACTACCGGCNRG	GGCCGgGccgGAC--CgGGCCGG	-	5	2	0	2.20	1.10
Chr4:40308772-40308794	40308778	4	RNA	GGCCGAGGTCGACTACCGGCNRG	GGgCGAaGatGACT--CGGgCGG	-	5	2	0	2.20	1.10
Chr1:141832898-141832920	141832914	4	RNA	GGCCGAGGTCGACTACCGGCNRG	GtCCGgaGTt--CTtCCGGCTGG	+	5	2	0	2.20	1.10
Chr10:25201964-25201986	25201969	3	RNA	GGCCGAGGTCGACTACCGGCNRG	G-aCGgGGTCGACaACgaGCGGG	-	5	1	0	2.20	1.10
Chr7:70569246-70569268	70569251	3	DNA	GGC--CGAGGTCGACTACCGGCNRG	GGCCTCGgGGgCtACacCCGGCGGG	-	5	2	0	2.20	1.10
Chr5:216940342-216940364	216940347	3	RNA	GGCCGAGGTCGACTACCGGCNRG	GGgCG-GGcCaACctCCGGCCGG	-	5	1	0	2.20	1.10
Chr5:84347854-84347876	84347859	3	X	GGCCGAGGTCGACTACCGGCNRG	GGaCGAGcTCGgCgAgCGGCAGG	-	5	0	0	2.20	1.10
Chr1:115548034-115548056	115548050	4	RNA	GGCCGAGGTCGACTACCGGCNRG	GGCgGAGGg-GctTACCGGCGGG	+	4	1	0	2.20	1.10
Chr7:48721400-48721422	48721416	4	RNA	GGCCGAGGTCGACTACCGGCNRG	cG--GAGGgaGAaTACCcGCTGG	+	5	2	0	2.20	1.10
Chr8:23077540-23077562	23077547	5	RNA	GGCCGAGGTCGACTACCGGCNRG	GGaCGAtcTCG--TAgaGGCGAG	-	5	2	0	2.20	1.10
Chr7:114036794-114036816	114036811	3	DNA	GGC-CGAGGTCGACTACCGGCNRG	GcCTCGAGGaCGtCTtCCGGCTGG	+	4	1	0	2.20	1.10
Chr3:20538837-20538859	20538841	2	RNA	GGCCGAGGTCGACTACCGGCNRG	GtCCG--GTgcACcACCGGaCAG	-	5	2	0	2.20	1.10
Chr4:202411281-202411303	202411298	3	RNA	GGCCGAGGTCGACTACCGGCNRG	GtCCG--GTgcACcACCGGaCAG	+	5	2	0	2.20	1.10
Chr8:80758363-80758385	80758381	2	RNA	GGCCGAGGTCGACTACCGGCNRG	GcC--gGGTCGtCcACCGGCGGG	+	4	2	0	2.20	1.10
Chr5:203358148-203358170	203358154	4	RNA	GGCCGAGGTCGACTACCGGCNRG	GGaCcAGGTCG--TcCCaGtTGG	-	5	2	0	2.20	1.10
Chr7:103576040-103576062	103576046	4	RNA	GGCCGAGGTCGACTACCGGCNRG	aGCtGAGGTCttCT--CGGCCAG	-	4	2	0	2.20	1.10
Chr6:139959918-139959940	139959933	5	RNA	GGCCGAGGTCGACTACCGGCNRG	GGC--tcGcgGACTACCGGtTGG	+	5	2	0	2.20	1.10
Chr7:62338175-62338197	62338190	5	RNA	GGCCGAGGTCGACTACCGGCNRG	GcCCGAGaTCGACctCa--CCGG	+	5	2	0	2.20	1.10
Chr1:121232818-121232840	121232825	5	RNA	GGCCGAGGTCGACTACCGGCNRG	GGCtGAG--CGcaTggCGGCGGG	-	5	2	0	2.20	1.10
Chr5:153362641-153362663	153362659	2	RNA	GGCCGAGGTCGACTACCGGCNRG	GtCCGAGGa--ACaACgGcCGAG	+	5	2	0	2.20	1.10
Chr2:105895567-105895589	105895583	4	RNA	GGCCGAGGTCGACTACCGGCNRG	cGgCGAGGa--ACcACCGtCGGG	+	5	2	0	2.20	1.10
Chr3:181915416-181915438	181915433	3	RNA	GGCCGAGGTCGACTACCGGCNRG	GGC--tcGcgGACTACCGGCTGG	+	4	2	0	2.20	1.10
Chr6:160902460-160902482	160902465	3	RNA	GGCCGAGGTCGACTACCGGCNRG	G-CCGAGGcCGcggAgCGGCGGG	-	5	1	0	2.20	1.10
Chr2:82786509-82786531	82786513	2	RNA	GGCCGAGGTCGACTACCGGCNRG	GaCCGAGG-CGACgACaaGaAGG	-	5	1	0	2.20	1.10
Chr10:60737498-60737520	60737515	3	RNA	GGCCGAGGTCGACTACCGGCNRG	caCCaAGG--GAtTACCGtCAAG	+	5	2	0	2.20	1.10
Chr4:83929226-83929248	83929231	3	RNA	GGCCGAGGTCGACTACCGGCNRG	tGCCGAGGTCGgCcgC--GCCGG	-	4	2	0	2.20	1.10
Chr1:15964999-15965021	15965005	4	RNA	GGCCGAGGTCGACTACCGGCNRG	GGCaGAGG-CGcggACCGaCAAG	-	5	1	0	2.20	1.10
Chr4:45643856-45643878	45643871	5	RNA	GGCCGAGGTCGACTACCGGCNRG	GcgCGAGGcCGA--ACgGGCCGG	+	4	2	0	2.20	1.10
Chr3:23439613-23439635	23439617	2	RNA	GGCCGAGGTCGACTACCGGCNRG	GtCCG--GTgcACcACCGGaTAG	-	5	2	0	2.20	1.10
Chr2:146215252-146215274	146215258	4	RNA	GGCCGAGGTCGACTACCGGCNRG	GaCCGActcCGACcACCG-CCAG	-	5	1	0	2.20	1.10
Chr2:151785958-151785980	151785973	5	RNA	GGCCGAGGTCGACTACCGGCNRG	G-CCGgGGTCGgCTcCgGGtCAG	+	5	1	0	2.20	1.10
Chr2:53620626-53620648	53620632	4	DNA	GGCCGA-GGTCGACTACCGGCNRG	GGCgGACGGTCcgCgACCGaCCAG	-	5	1	0	2.20	1.10
Chr2:101555183-101555205	101555187	2	DNA	GGCCGAGGTCGACTACC--GGCNRG	GGCCGAGcTCGcCgAgCTGGGgCGG	-	5	2	0	2.20	1.10
Chr2:162836192-162836214	162836198	4	RNA	GGCCGAGGTCGACTACCGGCNRG	GGC--AGGcCaACctCCGGCCGG	-	4	2	0	2.20	1.10
Chr1:96702091-96702113	96702106	5	DNA	GGC--CGAGGTCGACTACCGGCNRG	cGCACgGAGGTCGgCTACaGcCAGG	+	5	2	0	2.20	1.10
Chr3:206541231-206541253	206541237	4	RNA	GGCCGAGGTCGACTACCGGCNRG	GGC--tcGcgGAtTACCGGCTGG	-	5	2	0	2.20	1.10
Chr6:17339644-17339666	17339648	2	DNA	GGCCGAGGTCGAC--TACCGGCNRG	tGCCGAcGTCGACCGTcCgGGgGAG	-	5	2	0	2.20	1.10
Chr1:116457339-116457361	116457345	4	RNA	GGCCGAGGTCGACTACCGGCNRG	GGCaGcGaTaGcCT--CGGCCGG	-	5	2	0	2.20	1.10
Chr10:110164474-110164496	110164481	5	RNA	GGCCGAGGTCGACTACCGGCNRG	GGCCGAGGgCGAggACC--aTGG	-	4	2	0	2.20	1.10
Chr5:98942464-98942486	98942471	5	RNA	GGCCGAGGTCGACTACCGGCNRG	GcCCGAGGaCGctT--CaGCAGG	-	5	2	0	2.20	1.10
Chr8:64697155-64697177	64697162	5	RNA	GGCCGAGGTCGACTACCGGCNRG	GGgCGgGGTtGgCT-CCGGgAGG	-	5	1	0	2.20	1.10
Chr9:70823798-70823820	70823814	4	RNA	GGCCGAGGTCGACTACCGGCNRG	GGCatAtG-CGgCaACCGGCAAG	+	5	1	0	2.20	1.10
Chr6:42712995-42713017	42712999	2	RNA	GGCCGAGGTCGACTACCGGCNRG	GGCtGAGGaaccCTA--GGCCAG	-	5	2	0	2.20	1.10
Chr4:168633378-168633400	168633394	4	RNA	GGCCGAGGTCGACTACCGGCNRG	GcCgGAGGT-GACgACtGGtGGG	+	5	1	0	2.20	1.10
Chr2:166306364-166306386	166306381	3	RNA	GGCCGAGGTCGACTACCGGCNRG	GGgCGcGGTCGACTgC-GagGAG	+	5	1	0	2.20	1.10
Chr4:36465107-36465129	36465123	4	RNA	GGCCGAGGTCGACTACCGGCNRG	GGCCGccGT-GAgTACaGGtGAG	+	5	1	0	2.20	1.10
Chr3:190137786-190137808	190137804	2	RNA	GGCCGAGGTCGACTACCGGCNRG	GGCCG-GGcCatCcACtGGCGGG	+	5	1	0	2.20	1.10
Chr8:173555957-173555979	173555963	4	RNA	GGCCGAGGTCGACTACCGGCNRG	aGCCGAGGatGtCT--CGGgCAG	-	5	2	0	2.20	1.10
Chr8:40897227-40897249	40897243	4	RNA	GGCCGAGGTCGACTACCGGCNRG	cGCCGActTCG--TAgCGGtGGG	+	5	2	0	2.20	1.10
Chr9:68573271-68573293	68573288	3	RNA	GGCCGAGGTCGACTACCGGCNRG	GGaCcAGGTCG--TcCCaGtTGG	+	5	2	0	2.20	1.10
Chr2:3354111-3354133	3354127	4	RNA	GGCCGAGGTCGACTACCGGCNRG	cGCCGAGGggGAagAC--GCTGG	+	5	2	0	2.20	1.10
Chr3:182620682-182620704	182620699	3	RNA	GGCCGAGGTCGACTACCGGCNRG	GGC--tcGcgGACTACCGGCTGG	+	4	2	0	2.20	1.10
Chr1:13638939-13638961	13638945	4	RNA	GGCCGAGGTCGACTACCGGCNRG	GGCCttGcTacACT--CGGCGAG	-	5	2	0	2.20	1.10
Chr6:133118535-133118557	133118540	3	RNA	GGCCGAGGTCGACTACCGGCNRG	GGC--tcGcgGACTACCGGCTGG	-	4	2	0	2.20	1.10
Chr7:77522969-77522991	77522976	5	RNA	GGCCGAGGTCGACTACCGGCNRG	GGCgcAGcTCcACT-CCaGCGAG	-	5	1	0	2.20	1.10
Chr6:78565157-78565179	78565172	5	RNA	GGCCGAGGTCGACTACCGGCNRG	GGttGAGaT--AaTACCGtCTGG	+	5	2	0	2.20	1.10
Chr2:140635144-140635166	140635150	4	RNA	GGCCGAGGTCGACTACCGGCNRG	GGaCGAcGaCGACTA--tGaTAG	-	5	2	0	2.20	1.10
Chr2:68204360-68204382	68204364	2	RNA	GGCCGAGGTCGACTACCGGCNRG	GaCCGAGGaCGcCT-CgGGaTGG	-	5	1	0	2.20	1.10
Chr9:134814353-134814375	134814369	4	RNA	GGCCGAGGTCGACTACCGGCNRG	GGgtGgGGTCGgCT--CGGgGAG	+	5	2	0	2.20	1.10
Chr7:90520160-90520182	90520178	2	RNA	GGCCGAGGTCGACTACCGGCNRG	acCCGAaGTCGAtTAtC--CCGG	+	5	2	0	2.20	1.10
Chr10:90152598-90152620	90152605	5	RNA	GGCCGAGGTCGACTACCGGCNRG	GGggGAGagCGA--AgCGGCCGG	-	5	2	0	2.20	1.10
Chr2:224205374-224205396	224205389	5	RNA	GGCCGAGGTCGACTACCGGCNRG	GGgCG-GGcCaACccCCGGCCAG	+	5	1	0	2.20	1.10
Chr5:169614276-169614298	169614292	4	RNA	GGCCGAGGTCGACTACCGGCNRG	G-aCGgGGTCGACaACgaGCGGG	+	5	1	0	2.20	1.10
Chr4:59754561-59754583	59754565	2	RNA	GGCCGAGGTCGACTACCGGCNRG	GGaCcAGGTCG--TcCCaGtTGG	-	5	2	0	2.20	1.10
Chr8:36653470-36653492	36653476	4	DNA	GGC-CGAGGTCGACTACCGGCNRG	GcCTCGAGGaCGtCTtCCaGCTGG	-	5	1	0	2.20	1.10
Chr1:118844117-118844139	118844122	3	DNA	GGCCGAGGTCGAC-TACCGGCNRG	GGtCGAGGTCGACGTAgtcGCGGG	-	4	1	0	2.20	1.10
Chr2:84659413-84659435	84659431	2	RNA	GGCCGAGGTCGACTACCGGCNRG	GGCCGAGcgCGA--AggGGgGAG	+	5	2	0	2.20	1.10
Chr6:71113471-71113493	71113489	2	RNA	GGCCGAGGTCGACTACCGGCNRG	GGCCGAcaTgGA--ACtGGaCGG	+	5	2	0	2.20	1.10
Chr4:38243720-38243742	38243738	2	RNA	GGCCGAGGTCGACTACCGGCNRG	GGaCcAGGTCG--TcCCaGtTGG	+	5	2	0	2.20	1.10
Chr1:121532612-121532634	121532618	4	RNA	GGCCGAGGTCGACTACCGGCNRG	GtCCcAGGTCGAacACC--tGGG	-	5	2	0	2.20	1.10
Chr9:81920425-81920447	81920429	2	RNA	GGCCGAGGTCGACTACCGGCNRG	GGttcctGTCGACTAC-GGCGAG	-	5	1	0	2.20	1.10
Chr4:183774110-183774132	183774126	4	RNA	GGCCGAGGTCGACTACCGGCNRG	GGCCGA--TaGcCcgCCcGCCGG	+	5	2	0	2.20	1.10
Chr7:156354200-156354222	156354206	4	RNA	GGCCGAGGTCGACTACCGGCNRG	GGaCGAcGaCGACTAC--GaTAG	-	4	2	0	2.20	1.10
Chr1:42844389-42844411	42844405	4	RNA	GGCCGAGGTCGACTACCGGCNRG	G-aCGAGGTCGACgACgaGCGGG	+	4	1	0	2.20	1.10
Chr5:50647306-50647328	50647324	2	RNA	GGCCGAGGTCGACTACCGGCNRG	GGaCGAGGcgG--cAaCGGCAAG	+	5	2	0	2.20	1.10
Chr4:219504700-219504722	219504706	4	RNA	GGCCGAGGTCGACTACCGGCNRG	taCCG--GTCGACaACCaGCAGG	-	4	2	0	2.20	1.10
Chr9:138160150-138160172	138160165	5	RNA	GGCCGAGGTCGACTACCGGCNRG	GagCGA--TtGACTACtGGCTGG	+	4	2	0	2.20	1.10
Chr2:18023142-18023164	18023147	3	RNA	GGCCGAGGTCGACTACCGGCNRG	GcCCGccGTCGACctCC--CCAG	-	5	2	0	2.20	1.10
Chr2:4196458-4196480	4196475	3	RNA	GGCCGAGGTCGACTACCGGCNRG	GGCgGcGGTCGACggC--GaCGG	+	5	2	0	2.20	1.10
Chr7:111741043-111741065	111741061	2	DNA	GGCCGAGGTCGACTACCGG--CNRG	GGtCGAGGTCGAgTcCgaGCCCCGG	+	5	2	0	2.20	1.10
Chr6:5126873-5126895	5126880	5	RNA	GGCCGAGGTCGACTACCGGCNRG	GGgCGAGGgCagCT--CGaCCGG	-	5	2	0	2.20	1.10
Chr7:41639652-41639674	41639669	3	X	GGCCGAGGTCGACTACCGGCNRG	GGCactGGgCGACTACCaGCGGG	+	5	0	0	2.20	1.10
Chr6:137454491-137454513	137454508	3	RNA	GGCCGAGGTCGACTACCGGCNRG	GGCCGAGcgCGA--ACgGGgGAG	+	4	2	0	2.20	1.10
Chr1:269237381-269237403	269237387	4	RNA	GGCCGAGGTCGACTACCGGCNRG	G-aCGgGGTCGACgACgaGCGGG	-	5	1	0	2.20	1.10
Chr3:73434971-73434993	73434987	4	RNA	GGCCGAGGTCGACTACCGGCNRG	GGgCGAG--CaACaACaGGgGAG	+	5	2	0	2.20	1.10
Chr2:43076225-43076247	43076242	3	RNA	GGCCGAGGTCGACTACCGGCNRG	GcCCGAGccCGAgT--CGGtAGG	+	5	2	0	2.20	1.10
Chr3:190096978-190097000	190096995	3	RNA	GGCCGAGGTCGACTACCGGCNRG	GGCtGAGGcCG-CTgCgGGgAAG	+	5	1	0	2.20	1.10
Chr5:157797674-157797696	157797691	3	DNA	GGC-CGAGGTCGACTACCGGCNRG	GcCTCGgGGaCGtCTtCCGGCTGG	+	5	1	0	2.20	1.10
Chr3:75038537-75038559	75038542	3	RNA	GGCCGAGGTCGACTACCGGCNRG	G-aCGgGGTCGACgACgaGCGGG	-	5	1	0	2.20	1.10
Chr1:149668086-149668108	149668090	2	RNA	GGCCGAGGTCGACTACCGGCNRG	GGCaGcGGc--ACTtCgGGCGGG	-	5	2	0	2.20	1.10
Chr4:191273402-191273424	191273420	2	RNA	GGCCGAGGTCGACTACCGGCNRG	G--CGAcGaCGACaACaGcCGAG	+	5	2	0	2.20	1.10
Chr8:68050886-68050908	68050902	4	RNA	GGCCGAGGTCGACTACCGGCNRG	aGCCGcGGTCG--TACgtGgTGG	+	5	2	0	2.20	1.10
Chr1:293039009-293039031	293039014	3	RNA	GGCCGAGGTCGACTACCGGCNRG	GGaaGAGtTCt--TACCaGCAGG	-	5	2	0	2.20	1.10
Chr8:130549515-130549537	130549533	2	DNA	GGCCGAG--GTCGACTACCGGCNRG	GGCCGAGCCGTCtACTcggGcCGGG	+	5	2	0	2.20	1.10
Chr3:90533195-90533217	90533210	5	RNA	GGCCGAGGTCGACTACCGGCNRG	GGCgGAGGTCG--TtCgGcaGAG	+	5	2	0	2.20	1.10
Chr4:94317152-94317174	94317167	5	RNA	GGCCGAGGTCGACTACCGGCNRG	GGCCGAtcTCG--cAtCaGCAAG	+	5	2	0	2.20	1.10
Chr7:114277476-114277498	114277482	4	RNA	GGCCGAGGTCGACTACCGGCNRG	GGgCcAGGTCGAgTg--GGgGAG	-	5	2	0	2.20	1.10
Chr9:68194936-68194958	68194953	3	RNA	GGCCGAGGTCGACTACCGGCNRG	cGCCGtGGT-GtCTACtGGaGGG	+	5	1	0	2.20	1.10
Chr4:128501752-128501774	128501758	4	DNA	GGCCGAGGTCGACTACCGG--CNRG	GGCCGAGGcCGAgTcCgaGCCCTGG	-	5	2	0	2.20	1.10
Chr6:22010571-22010593	22010588	3	DNA	GGCCGAGGTCGA--CTACCGGCNRG	GcgCcAtcTCGATTCTACCGGCGAG	+	5	2	0	2.20	1.10
Chr3:187613975-187613997	187613992	3	RNA	GGCCGAGGTCGACTACCGGCNRG	GGCCGAGGcCGAgctC--GCCGG	+	4	2	0	2.20	1.10
Chr3:140909124-140909146	140909139	5	RNA	GGCCGAGGTCGACTACCGGCNRG	GGCC-AGaTCtACaACgaGCTGG	+	5	1	0	2.20	1.10
Chr6:85084128-85084150	85084144	4	RNA	GGCCGAGGTCGACTACCGGCNRG	GGaCGAcGcCG-CTgCCtGCAGG	+	5	1	0	2.20	1.10
Chr3:41909868-41909890	41909874	4	RNA	GGCCGAGGTCGACTACCGGCNRG	GGCCGAcGgC--CcgCCcGCCGG	-	5	2	0	2.20	1.10

Chr8:47843164-47843186	47843170	4	RNA	GGCCGAGGTCGACTACCGGCNRG	GGCCGgGcTgGgCTg--GGCTGG	-	5	2	0	2.20	1.10
Chr5:209544940-209544962	209544945	3	DNA	GG--CCGAGGTCGACTACCGGCNRG	GGACCCtAcaTCcACTACCaGCAGG	-	5	2	0	2.20	1.10
Chr5:204417049-204417071	204417056	5	RNA	GGCCGAGGTCGACTACCGGCNRG	G-aCGgGGTCGACaACgaGCGGG	-	5	1	0	2.20	1.10
Chr3:203539718-203539740	203539722	2	RNA	GGCCGAGGTCGACTACCGGCNRG	G--CGAGGTCGcCcAtCtGCGAG	-	4	2	0	2.20	1.10
Chr4:79045468-79045490	79045472	2	RNA	GGCCGAGGTCGACTACCGGCNRG	caCCGAGtTgGAgTA--GGCGGG	-	5	2	0	2.20	1.10
Chr4:73255875-73255897	73255893	2	RNA	GGCCGAGGTCGACTACCGGCNRG	tG--GAGGTtGcCaACCGGaCAG	+	5	2	0	2.20	1.10
Chr4:241491282-241491304	241491300	2	RNA	GGCCGAGGTCGACTACCGGCNRG	GGCgGAGGTCG--TtCgGcaGAG	+	5	2	0	2.20	1.10
Chr10:127161537-127161559	127161555	2	DNA	GG--CCGAGGTCGACTACCGGCNRG	GGTCCCGAGGTCGAtccCCctCGGG	+	5	2	0	2.20	1.10
Chr1:286188610-286188632	286188615	3	RNA	GGCCGAGGTCGACTACCGGCNRG	GGCCGAGtcCGAgccCC--CGGG	-	5	2	0	2.20	1.10
Chr1:204172671-204172693	204172675	2	RNA	GGCCGAGGTCGACTACCGGCNRG	GGtCGAGGcCGAgT--CcGgGGG	-	5	2	0	2.20	1.10
Chr10:43160552-43160574	43160568	4	RNA	GGCCGAGGTCGACTACCGGCNRG	GGC-GAGGTCGAgagCgGGCGGG	+	4	1	0	2.20	1.10
Chr7:17750473-17750495	17750478	3	DNA	GGCCGAGGTCGACTA--CCGGCNRG	ctCCaAGGcCGACcAATCCGGCAAG	-	5	2	0	2.20	1.10
Chr3:99690263-99690285	99690267	2	RNA	GGCCGAGGTCGACTACCGGCNRG	GGCCG-cGTCtACTAtCaGtAGG	-	5	1	0	2.20	1.10
Chr10:120823197-120823219	120823201	2	RNA	GGCCGAGGTCGACTACCGGCNRG	GGCCGAGGcCtcCT-CgGGCTGG	-	4	1	0	2.20	1.10
Chr7:74686790-74686812	74686808	2	DNA	GGCCGAGGTC-GACTACCGGCNRG	GGCCGAGGcCTGAggtCgGGCGAG	+	5	1	0	2.20	1.10
Chr3:56717446-56717468	56717451	3	DNA	GGCCGAGGTCGACTACC--GGCNRG	GcCCGAGagCaACcACCGTGGCCAG	-	5	2	0	2.20	1.10
Chr8:160479712-160479734	160479717	3	RNA	GGCCGAGGTCGACTACCGGCNRG	GGCCG-GGTCatCcACtGGCGGG	-	4	1	0	2.20	1.10
Chr1:120664858-120664880	120664864	4	RNA	GGCCGAGGTCGACTACCGGCNRG	GG--GAGGTCGAgcACCGGCCGG	-	2	2	0	2.20	1.10
Chr7:93432459-93432481	93432476	3	DNA	GGCCGAGGT--CGACTACCGGCNRG	GGtCGtGGTGGCGAactCCGGCGAG	+	5	2	0	2.20	1.10
Chr2:112185830-112185852	112185835	3	DNA	GGC--CGAGGTCGACTACCGGCNRG	GGCCTCGgGGgCtACacCCGGCGGG	-	5	2	0	2.20	1.10
Chr7:19971692-19971714	19971697	3	DNA	GGCCG--AGGTCGACTACCGGCNRG	GGtCGTTAGGTCagCTACCttCCGG	-	5	2	0	2.20	1.10
Chr1:16101231-16101253	16101237	4	RNA	GGCCGAGGTCGACTACCGGCNRG	aGCCGAGGgC-ACctCgGGCAAG	-	5	1	0	2.20	1.10
Chr10:30147091-30147113	30147108	3	RNA	GGCCGAGGTCGACTACCGGCNRG	G-CCGcGGcCGAacACCGaCGAG	+	5	1	0	2.20	1.10
Chr1:122440334-122440356	122440350	4	RNA	GGCCGAGGTCGACTACCGGCNRG	GGCCt--GcCaACTtCCGGgCAG	+	5	2	0	2.20	1.10
Chr4:83007020-83007042	83007036	4	RNA	GGCCGAGGTCGACTACCGGCNRG	GGCCGAGccCGAg--CCaGgGGG	+	5	2	0	2.20	1.10
Chr2:49908336-49908358	49908341	3	RNA	GGCCGAGGTCGACTACCGGCNRG	GtgCaAGcTC--CTcCCGGCGAG	-	5	2	0	2.20	1.10
Chr5:179981708-179981730	179981726	2	RNA	GGCCGAGGTCGACTACCGGCNRG	GGCCGAGGgCG--cgCgcGCGGG	+	5	2	0	2.20	1.10
Chr6:116352215-116352237	116352222	5	RNA	GGCCGAGGTCGACTACCGGCNRG	GGC--ttGcgGACTACCGGCTGG	-	4	2	0	2.20	1.10
Chr1:54618101-54618123	54618106	3	RNA	GGCCGAGGTCGACTACCGGCNRG	GGC--tcGTgGACTACCGtCTGG	-	4	2	0	2.20	1.10
Chr3:142032089-142032111	142032096	5	RNA	GGCCGAGGTCGACTACCGGCNRG	G--CaAGGaCGACTAgCGGgGAG	-	4	2	0	2.20	1.10
Chr1:116354814-116354836	116354831	3	RNA	GGCCGAGGTCGACTACCGGCNRG	GGCCcAcGgCG--TttCGGCCGG	+	5	2	0	2.20	1.10
Chr4:47466419-47466441	47466425	4	RNA	GGCCGAGGTCGACTACCGGCNRG	GtCCG--GTgcACcACCGGaCAG	-	5	2	0	2.20	1.10
Chr10:104950422-104950444	104950437	5	RNA	GGCCGAGGTCGACTACCGGCNRG	GGC--AGGcCaAgctCCGGCCAG	+	5	2	0	2.20	1.10
Chr10:45567296-45567318	45567312	4	DNA	G--GCCGAGGTCGACTACCGGCNRG	GCCGCCGgGtTCGACTcCgGGtCAG	+	5	2	0	2.20	1.10
Chr7:169893467-169893489	169893482	5	RNA	GGCCGAGGTCGACTACCGGCNRG	GGCCGAGc-CGtCcACtaGCGGG	+	5	1	0	2.20	1.10
Chr4:189298535-189298557	189298551	4	RNA	GGCCGAGGTCGACTACCGGCNRG	GGaCGAGccCGACT-CtGGtTAG	+	5	1	0	2.20	1.10
Chr8:42713073-42713095	42713088	5	DNA	GGCCGAGGTCGAC--TACCGGCNRG	GGCCGActTCGACCATcCCcGaCGG	+	5	2	0	2.20	1.10
Chr3:225281898-225281920	225281913	5	RNA	GGCCGAGGTCGACTACCGGCNRG	GcCaGtGGaCGgC--CCGGCCAG	+	5	2	0	2.20	1.10
Chr5:214864117-214864139	214864135	2	RNA	GGCCGAGGTCGACTACCGGCNRG	GGCtGgGGTaG--cACCGGgAGG	+	5	2	0	2.20	1.10
Chr9:102201445-102201467	102201450	3	RNA	GGCCGAGGTCGACTACCGGCNRG	GGggGAGGTgG--TAgaGGCTGG	-	5	2	0	2.20	1.10
Chr6:165165861-165165883	165165866	3	RNA	GGCCGAGGTCGACTACCGGCNRG	GGatGAGGagGACT--CGGaCGG	-	5	2	0	2.20	1.10
Chr3:111276764-111276786	111276768	2	RNA	GGCCGAGGTCGACTACCGGCNRG	GtCCG--GTaGAtcACCcGCAGG	-	5	2	0	2.20	1.10
Chr6:66915847-66915869	66915863	4	RNA	GGCCGAGGTCGACTACCGGCNRG	cGCCGAGcTCGgCcAC--GtCAG	+	5	2	0	2.20	1.10
Chr3:135142620-135142642	135142626	4	RNA	GGCCGAGGTCGACTACCGGCNRG	GGCCGAGGgt-ACctCgGGCTAG	-	5	1	0	2.20	1.10
Chr10:146259682-146259704	146259687	3	DNA	GGCCGAGGTCGAC--TACCGGCNRG	GGgCGAGGaCGACGGcgCCaGCGAG	-	5	2	0	2.20	1.10
Chr8:129664790-129664812	129664796	4	RNA	GGCCGAGGTCGACTACCGGCNRG	G-aCGgGGTCGACgACgaGCGGG	-	5	1	0	2.20	1.10
Chr4:209203159-209203181	209203163	2	RNA	GGCCGAGGTCGACTACCGGCNRG	GG--GAGGaCtAaTAtCGGgGGG	-	5	2	0	2.20	1.10
Chr8:42382106-42382128	42382112	4	DNA	GGCC--GAGGTCGACTACCGGCNRG	cGCCTTGgGGaCGtCTtCCGGCTGG	-	5	2	1	1.10	1.05
Chr2:166531357-166531379	166531361	2	RNA	GGCCGAGGTCGACTACCGGCNRG	GGC--AGGcCGAgTACCGGCCGG	-	2	2	1	1.10	1.05
Chr7:162222442-162222464	162222448	4	DNA	GGC-CGAGGTCGACTACCGGCNRG	GcCTCGAGGaCGtCTtCCGGCTGG	-	4	1	1	1.10	1.05
Chr6:153883486-153883508	153883492	4	RNA	GGCCGAGGTCGACTACCGGCNRG	GGCgGctGTCGA--ACCtGaAAG	-	5	2	1	1.10	1.05
Chr4:157776264-157776286	157776281	3	DNA	GGCCGA--GGTCGACTACCGGCNRG	GGCgcATCGGgCGAgTACtGGCAGG	+	5	2	2	0.00	1.00
Chr5:70912068-70912090	70912085	3	RNA	GGCCGAGGTCGACTACCGGCNRG	GtCCG--GTgcACcACCGGaCAG	+	5	2	2	0.00	1.00
Chr2:192335731-192335753	192335738	5	RNA	GGCCGAGGTCGACTACCGGCNRG	GGgCG-GGcCaACctCCGGCCGG	-	5	1	2	0.00	1.00
Chr7:41494072-41494094	41494089	3	RNA	GGCCGAGGTCGACTACCGGCNRG	GGgCG-GGcCaACctCCGGCCGG	+	5	1	2	0.00	1.00
Chr9:85864022-85864044	85864029	5	RNA	GGCCGAGGTCGACTACCGGCNRG	GGCCGAGcTgGgC--CgGGCCGG	-	4	2	2	0.00	1.00
Chr8:17821069-17821091	17821076	5	RNA	GGCCGAGGTCGACTACCGGCNRG	GGC-GAGGTCGAggACCttgGGG	-	5	1	2	0.00	1.00
Chr5:82156214-82156236	82156221	5	DNA	GGCCGAGGTCGACTA-CCGGCNRG	GtgCGAGGcgGACTAACCGGCCGG	-	4	1	2	0.00	1.00
Chr8:134202546-134202568	134202553	5	DNA	GGC--CGAGGTCGACTACCGGCNRG	GGCCTCGgGGgCtACacCCGGCGGG	-	5	2	2	0.00	1.00
Chr1:29555838-29555860	29555845	5	RNA	GGCCGAGGTCGACTACCGGCNRG	GcCCGAGGcCGA--ACtcGtGGG	-	5	2	2	0.00	1.00
Chr8:70500976-70500998	70500983	5	RNA	GGCCGAGGTCGACTACCGGCNRG	GGCaGAGGgtG--cACCGGaTAG	-	5	2	2	0.00	1.00
Chr7:46564084-46564106	46564101	3	RNA	GGCCGAGGTCGACTACCGGCNRG	G--CGAGcTCGACTgtgGGaGGG	+	5	2	2	0.00	1.00
Chr4:25306659-25306681	25306666	5	RNA	GGCCGAGGTCGACTACCGGCNRG	GGCC-AGGTCGtCcACtGGCGGG	-	3	1	2	0.00	1.00
Chr2:237257-237279	237264	5	RNA	GGCCGAGGTCGACTACCGGCNRG	aGCCcAGGaCG--TACaaGCTGG	-	5	2	2	0.00	1.00
Chr7:147128045-147128067	147128052	5	RNA	GGCCGAGGTCGACTACCGGCNRG	GGC--tcGcgGACTACCGGCTGG	-	4	2	2	0.00	1.00
Chr3:177682486-177682508	177682503	3	RNA	GGCCGAGGTCGACTACCGGCNRG	GGCCGAcGTCGAggAa-GaCGAG	+	5	1	2	0.00	1.00
Chr4:96989885-96989907	96989902	3	DNA	GGCCGAGGTCGACTAC--CGGCNRG	GcCCGAcaTCGcCTACGTCGtCCAG	+	5	2	2	0.00	1.00
Chr5:4621852-4621874	4621859	5	RNA	GGCCGAGGTCGACTACCGGCNRG	cGCCtAGGgCtgCTACC--CTAG	-	5	2	2	0.00	1.00
Chr5:148227169-148227191	148227186	3	RNA	GGCCGAGGTCGACTACCGGCNRG	G--CGAGGTCctCctCCtGCCAG	+	5	2	2	0.00	1.00
Chr9:107839649-107839671	107839656	5	RNA	GGCCGAGGTCGACTACCGGCNRG	cGaCGAGcTCGACT-CtGGtTGG	-	5	1	2	0.00	1.00
Chr1:238446048-238446070	238446055	5	RNA	GGCCGAGGTCGACTACCGGCNRG	GGgCGcG--aGgCTACtGGCGGG	-	5	2	2	0.00	1.00
Chr9:44328395-44328417	44328402	5	RNA	GGCCGAGGTCGACTACCGGCNRG	GaCCGtGcTCcACcACCG-CCAG	-	5	1	2	0.00	1.00
Chr5:134292209-134292231	134292216	5	RNA	GGCCGAGGTCGACTACCGGCNRG	GGtaGAGGTCGgCTgCC--CCAG	-	4	2	2	0.00	1.00
Chr5:108954613-108954635	108954620	5	RNA	GGCCGAGGTCGACTACCGGCNRG	GGC-GAGGTCGACgACgcGaTGG	-	4	1	2	0.00	1.00
Chr3:101905042-101905064	101905059	3	RNA	GGCCGAGGTCGACTACCGGCNRG	GGCCGAGcTgGgCcAC--GCTAG	+	4	2	2	0.00	1.00
Chr4:116631328-116631350	116631335	5	RNA	GGCCGAGGTCGACTACCGGCNRG	GGCgGcGGT-GACTACtcGCTGG	-	4	1	2	0.00	1.00
Chr3:215262195-215262217	215262212	3	RNA	GGCCGAGGTCGACTACCGGCNRG	GtCaGAGacCGAt--CCGGCCAG	+	5	2	2	0.00	1.00
Chr6:25868780-25868802	25868787	5	DNA	GGCCGAGGTCGACTACCGG--CNRG	GGCCGgGGTCGgCcACtGGCAtGAG	-	5	2	2	0.00	1.00
Chr10:60042644-60042666	60042651	5	DNA	GGCCGAGGT--CGACTACCGGCNRG	GGCCtAGaTCTCGcCTcCCGGgAGG	-	5	2	2	0.00	1.00
Chr4:67494805-67494827	67494812	5	RNA	GGCCGAGGTCGACTACCGGCNRG	GG--GAcGTCGACaggCGGgCAG	-	5	2	2	0.00	1.00
Chr6:48736027-48736049	48736044	3	RNA	GGCCGAGGTCGACTACCGGCNRG	GGCCGAG--CGcCagCCacCAGG	+	5	2	2	0.00	1.00
Chr1:7611163-7611185	7611180	3	DNA	G--GCCGAGGTCGACTACCGGCNRG	GATGaCtAGGcgGACcACCGGCGGG	+	5	2	2	0.00	1.00
Chr7:33669792-33669814	33669799	5	DNA	GGCCGAGGT-CGACTACCGGCNRG	GGCgGAGGTGCtgtTACCGtCGAG	-	5	1	2	0.00	1.00
Chr3:181977050-181977072	181977057	5	RNA	GGCCGAGGTCGACTACCGGCNRG	GaCgaAGGTgGACTA--GtCAGG	-	5	2	2	0.00	1.00
Chr2:240883021-240883043	240883038	3	RNA	GGCCGAGGTCGACTACCGGCNRG	G-CCGAGGgCGcCgACCaaCTGG	+	5	1	2	0.00	1.00
Chr2:34587738-34587760	34587745	5	RNA	GGCCGAGGTCGACTACCGGCNRG	aGCCGAGGgtGcCT-CgGGCGGG	-	5	1	2	0.00	1.00
Chr6:171197823-171197845	171197830	5	RNA	GGCCGAGGTCGACTACCGGCNRG	cGgCGAGcTtG--TACCGGCCGG	-	4	2	2	0.00	1.00
Chr5:88124067-88124089	88124074	5	RNA	GGCCGAGGTCGACTACCGGCNRG	GGatGAGGagGACT--CGGaCGG	-	5	2	2	0.00	1.00
Chr4:25873734-25873756	25873751	3	RNA	GGCCGAGGTCGACTACCGGCNRG	GGaCGAtccCGACT-CCGGtTAG	+	5	1	2	0.00	1.00
Chr8:34018605-34018627	34018622	3	RNA	GGCCGAGGTCGACTACCGGCNRG	GGaCcAGtTCGACTA--aGtCAG	+	5	2	2	0.00	1.00
Chr6:36346864-36346886	36346871	5	RNA	GGCCGAGGTCGACTACCGGCNRG	GaCCGgGGaatACT-CCGGCGAG	-	5	1	2	0.00	1.00
Chr2:188034390-188034412	188034407	3	RNA	GGCCGAGGTCGACTACCGGCNRG	GcCCGAGaTCGACctCa--CCGG	+	5	2	2	0.00	1.00
Chr3:64478044-64478066	64478051	5	RNA	GGCCGAGGTCGACTACCGGCNRG	G-aCGgGGTCGACgACgaGCGGG	-	5	1	2	0.00	1.00
Chr7:73682382-73682404	73682389	5	RNA	GGCCGAGGTCGACTACCGGCNRG	tGCCtgGGcCGACTg--GGCTGG	-	5	2	2	0.00	1.00
Chr2:71741824-71741846	71741831	5	RNA	GGCCGAGGTCGACTACCGGCNRG	GaCtaAGGTCaACTACt--CGGG	-	5	2	2	0.00	1.00
Chr4:153428986-153429008	153428993	5	RNA	GGCCGAGGTCGACTACCGGCNRG	GGCCGcGGcCG--cAgCGtCGGG	-	5	2	2	0.00	1.00
Chr9:58337476-58337498	58337493	3	DNA	GGCCGAGGTCGA--CTACCGGCNRG	GcgCcAtcTCGATTCTACCGGCGAG	+	5	2	2	0.00	1.00
Chr9:132678364-132678386	132678381	3	RNA	GGCCGAGGTCGACTACCGGCNRG	GGCaGAG--CaAgcACCGGCCGG	+	4	2	2	0.00	1.00
Chr2:207168615-207168637	207168622	5	RNA	GGCCGAGGTCGACTACCGGCNRG	G-aCGgGGTCGACaACgaGCTGG	-	5	1	2	0.00	1.00
Chr4:179402651-179402673	179402658	5	RNA	GGCCGAGGTCGACTACCGGCNRG	GGCtGAGGgtGcCT-CgGGCTGG	-	5	1	2	0.00	1.00
Chr6:78088383-78088405	78088390	5	RNA	GGCCGAGGTCGACTACCGGCNRG	tGCCGAaGctGA--ACCGaCCGG	-	5	2	2	0.00	1.00
Chr2:240810882-240810904	240810889	5	RNA	GGCCGAGGTCGACTACCGGCNRG	GGC-GtGGgCGACTAgCGGCTGG	-	3	1	2	0.00	1.00
Chr2:72299808-72299830	72299825	3	DNA	GGCCGA--GGTCGACTACCGGCNRG	GGCgGACAGGTgcACgACaGGCGGG	+	5	2	2	0.00	1.00
Chr2:194745311-194745333	194745328	3	RNA	GGCCGAGGTCGACTACCGGCNRG	GGCtGAGccCGgC--CCGGaGAG	+	5	2	2	0.00	1.00
Chr2:112259603-112259625	112259610	5	RNA	GGCCGAGGTCGACTACCGGCNRG	GctCGAGGTgcACTAC-GGgTAG	-	5	1	2	0.00	1.00
Chr10:88229475-88229497	88229492	3	DNA	GGCCGAGGTCGACTACCGG--CNRG	GGtCGAGGTCGAgTcCgaGCCCCGG	+	5	2	2	0.00	1.00
Chr3:235106549-235106571	235106566	3	DNA	GGCCGAGGTCGACTAC--CGGCNRG	cGCCGAtGcCGACgACGACGGCCAG	+	4	2	2	0.00	1.00
Chr5:112932903-112932925	112932910	5	DNA	GGCCGAGGTCG--ACTACCGGCNRG	GGCCGgGGTCGGGcacACCGGCGGG	-	4	2	2	0.00	1.00
Chr7:21230373-21230395	21230380	5	RNA	GGCCGAGGTCGACTACCGGCNRG	GGCCGAGcgCGA--AggGGgGAG	-	5	2	2	0.00	1.00
Chr6:125991478-125991500	125991485	5	RNA	GGCCGAGGTCGACTACCGGCNRG	GGC-GcGGTCGAactCtGGCGAG	-	5	1	2	0.00	1.00
Chr7:159024964-159024986	159024971	5	DNA	GGCCGAGGTCGACTACC--GGCNRG	GGCaGAGacCGtCTtCCGAGGCCGG	-	5	2	2	0.00	1.00
Chr1:255154078-255154100	255154095	3	RNA	GGCCGAGGTCGACTACCGGCNRG	G-aCGgGGTCGACgACgaGCGGG	+	5	1	2	0.00	1.00
Chr7:34149954-34149976	34149971	3	RNA	GGCCGAGGTCGACTACCGGCNRG	tGtCGtGcTCGAC--CCtGCAAG	+	5	2	2	0.00	1.00
Chr2:240043495-240043517	240043502	5	RNA	GGCCGAGGTCGACTACCGGCNRG	GtCCG--GTgcACcACCGGaTAG	-	5	2	2	0.00	1.00
Chr4:137440406-137440428	137440413	5	DNA	GGCCGAGGTCGACT--ACCGGCNRG	GtCgGAGGTgGACTGGAaCtGCAGG	-	5	2	2	0.00	1.00
Chr8:107558521-107558543	107558538	3	RNA	GGCCGAGGTCGACTACCGGCNRG	GcCCGAG--CGcCcACCtGCCGG	+	4	2	2	0.00	1.00
Chr1:304699677-304699699	304699684	5	RNA	GGCCGAGGTCGACTACCGGCNRG	G-aCGgGGTCGACaACgaGCGGG	-	5	1	2	0.00	1.00
Chr4:40328260-40328282	40328277	3	RNA	GGCCGAGGTCGACTACCGGCNRG	GGC--ttGcgGACTACCGGCTGG	+	4	2	2	0.00	1.00
Chr9:127799939-127799961	127799956	3	RNA	GGCCGAGGTCGACTACCGGCNRG	GGaCtAGGTCG--TcCCaGtTGG	+	5	2	2	0.00	1.00
Chr5:112627098-112627120	112627115	3	RNA	GGCCGAGGTCGACTACCGGCNRG	tGCCcAGG--GACaAgCGGgCAG	+	5	2	2	0.00	1.00
Chr5:23639270-23639292	23639277	5	DNA	GGC--CGAGGTCGACTACCGGCNRG	GGCCTCGgGGgCtACacCCGGCGGG	-	5	2	2	0.00	1.00
Chr6:63927539-63927561	63927546	5	RNA	GGCCGAGGTCGACTACCGGCNRG	aaCaGAGGc--ACgACCGGCAGG	-	5	2	2	0.00	1.00
Chr7:146105289-146105311	146105306	3	RNA	GGCCGAGGTCGACTACCGGCNRG	GGgCG-GGcCaACctCCGGCCAG	+	5	1	2	0.00	1.00
Chr2:114464788-114464810	114464795	5	RNA	GGCCGAGGTCGACTACCGGCNRG	GGCCGAGGTCGA-TggaGaCTGG	-	4	1	2	0.00	1.00
Chr1:54429794-54429816	54429811	3	DNA	G--GCCGAGGTCGACTACCGGCNRG	GATGaCGgGGTCGACaACgaGCGGG	+	5	2	2	0.00	1.00
Chr2:16510172-16510194	16510189	3	RNA	GGCCGAGGTCGACTACCGGCNRG	GtCCG--GaaGAaTACCGGaAAG	+	5	2	2	0.00	1.00

Chr2:1074862-1074884	1074879	3	DNA	GGCC-GAGGTCGACTACCGGCNRG	GGCCCGAGGgCGgCctCgGGCAAG	+	5	1	2	0.00	1.00
Chr8:13708258-13708280	13708275	3	RNA	GGCCGAGGTCGACTACCGGCNRG	GGtCGAGGcCGAgT--CtGaGGG	+	5	2	2	0.00	1.00
Chr1:127278942-127278964	127278949	5	RNA	GGCCGAGGTCGACTACCGGCNRG	GGCCcAtGgCG--TttCGGCCGG	-	5	2	2	0.00	1.00
Chr7:182137973-182137995	182137980	5	X	GGCCGAGGTCGACTACCGGCNRG	GGCCGAGGcaGACTgCaGcCTGG	-	5	0	2	0.00	1.00
Chr7:138466026-138466048	138466043	3	RNA	GGCCGAGGTCGACTACCGGCNRG	G-tCGgGGTCGACaACgaGCGGG	+	5	1	2	0.00	1.00
Chr1:23354265-23354287	23354272	5	RNA	GGCCGAGGTCGACTACCGGCNRG	GcCgGAGccCGAC--CCGtCAGG	-	5	2	2	0.00	1.00
Chr6:21058460-21058482	21058467	5	DNA	GGCCGAGGTCGAC-TACCGGCNRG	GGCCGAGacgGACGTAtgGGCTGG	-	5	1	2	0.00	1.00
Chr5:150123206-150123228	150123213	5	RNA	GGCCGAGGTCGACTACCGGCNRG	GGCCGAtGatGgCTA--GGgAGG	-	5	2	2	0.00	1.00
Chr3:93193878-93193900	93193885	5	RNA	GGCCGAGGTCGACTACCGGCNRG	GcCCGAGGaCaACcACC--aTGG	-	5	2	2	0.00	1.00
Chr2:189372054-189372076	189372071	3	RNA	GGCCGAGGTCGACTACCGGCNRG	GGCCGAttggGgCTAC-GGCTGG	+	5	1	2	0.00	1.00
Chr2:153252942-153252964	153252959	3	RNA	GGCCGAGGTCGACTACCGGCNRG	cGaCGAGGTgGA-ggCCGGCAGG	+	5	1	2	0.00	1.00
Chr2:42268940-42268962	42268957	3	RNA	GGCCGAGGTCGACTACCGGCNRG	GGCgGAGGTCG--TtCgGcaGAG	+	5	2	2	0.00	1.00
Chr1:10436165-10436187	10436172	5	RNA	GGCCGAGGTCGACTACCGGCNRG	G-aCGgGGTCGACaACgaGCGGG	-	5	1	2	0.00	1.00
Chr7:135877158-135877180	135877165	5	RNA	GGCCGAGGTCGACTACCGGCNRG	cGtCGAt-TCcACTACCGGCGGG	-	4	1	2	0.00	1.00
Chr5:195365636-195365658	195365643	5	RNA	GGCCGAGGTCGACTACCGGCNRG	GGCaGtGGTaGACgAg-GGCTGG	-	5	1	2	0.00	1.00
Chr3:196723329-196723351	196723336	5	RNA	GGCCGAGGTCGACTACCGGCNRG	GGCCGgaGTCGA--ACgtGaGAG	-	5	2	2	0.00	1.00
Chr5:189152310-189152332	189152317	5	RNA	GGCCGAGGTCGACTACCGGCNRG	GGCCGgcGgCGACaAC--GCCGG	-	4	2	2	0.00	1.00
Chr1:75649191-75649213	75649198	5	DNA	GGCCGAGGTCGACTACCG-GCNRG	GGtCGAGGTCGtCTgCaGTGgTGG	-	5	1	2	0.00	1.00
Chr7:115060114-115060136	115060131	3	RNA	GGCCGAGGTCGACTACCGGCNRG	GcagGAaGTCGAt--CCGGCGAG	+	5	2	2	0.00	1.00
Chr7:9117462-9117484	9117469	5	RNA	GGCCGAGGTCGACTACCGGCNRG	GGC-GcGGaCaACgACCGcCCGG	-	5	1	2	0.00	1.00
Chr1:79935085-79935107	79935092	5	RNA	GGCCGAGGTCGACTACCGGCNRG	GGaCcAGGTCG--TcCCaGtTGG	-	5	2	2	0.00	1.00
Chr7:158525418-158525440	158525435	3	RNA	GGCCGAGGTCGACTACCGGCNRG	GaCCG--GcCaACTcCtGGCCGG	+	5	2	2	0.00	1.00
Chr1:174887175-174887197	174887192	3	RNA	GGCCGAGGTCGACTACCGGCNRG	aG--GAGGTCcACacCaGGCAGG	+	5	2	2	0.00	1.00
Chr8:19981006-19981028	19981023	3	RNA	GGCCGAGGTCGACTACCGGCNRG	GGg-tAGGcCGAgcACCGGCCGG	+	5	1	2	0.00	1.00
Chr5:78952372-78952394	78952389	3	RNA	GGCCGAGGTCGACTACCGGCNRG	GGgCG-GGcCaACctCCGGCCGG	+	5	1	2	0.00	1.00
Chr1:251915702-251915724	251915709	5	RNA	GGCCGAGGTCGACTACCGGCNRG	tGCCGA--TacACcgCCGGCCGG	-	5	2	2	0.00	1.00
Chr5:5491921-5491943	5491928	5	RNA	GGCCGAGGTCGACTACCGGCNRG	GaCCGAGG-CGACgACaaGaAGG	-	5	1	2	0.00	1.00
Chr1:227453116-227453138	227453133	3	RNA	GGCCGAGGTCGACTACCGGCNRG	GGCCtc--TCGtCTctCGGCCAG	+	5	2	2	0.00	1.00
Chr6:130803392-130803414	130803409	3	RNA	GGCCGAGGTCGACTACCGGCNRG	GGCCc--GcCaACTtCCGGgCAG	+	5	2	2	0.00	1.00
Chr6:125785691-125785713	125785708	3	RNA	GGCCGAGGTCGACTACCGGCNRG	GGCC-AtGTCGtCcACtGGCGGG	+	4	1	2	0.00	1.00
Chr7:123720490-123720512	123720507	3	RNA	GGCCGAGGTCGACTACCGGCNRG	GcCtcgGGTCGACgAC-GGCGGG	+	5	1	2	0.00	1.00
Chr5:204222342-204222364	204222349	5	RNA	GGCCGAGGTCGACTACCGGCNRG	GtCCtcGG--GAtaACCGGCAGG	-	5	2	2	0.00	1.00
Chr1:58198497-58198519	58198504	5	DNA	GGCCGAGGTCGACTACCG--GCNRG	GGCgcAGGTCGgCgACCGAAtCGAG	-	5	2	2	0.00	1.00
Chr3:125996440-125996462	125996457	3	RNA	GGCCGAGGTCGACTACCGGCNRG	GGaCcAGGTCG--TcCCaGtTGG	+	5	2	2	0.00	1.00
Chr1:24117810-24117832	24117817	5	RNA	GGCCGAGGTCGACTACCGGCNRG	GGtCGA--TtGAtgcCCGGCCGG	-	5	2	2	0.00	1.00
Chr4:221793263-221793285	221793270	5	RNA	GGCCGAGGTCGACTACCGGCNRG	GtCCG--GTgcACcACCGGaTAG	-	5	2	2	0.00	1.00
Chr4:234088684-234088706	234088701	3	RNA	GGCCGAGGTCGACTACCGGCNRG	cGCCaAcGTCGgC--CCcGCCAG	+	5	2	2	0.00	1.00
Chr10:6006147-6006169	6006154	5	RNA	GGCCGAGGTCGACTACCGGCNRG	GagCGtGGcCGAtT--CGGCGAG	-	5	2	2	0.00	1.00
Chr4:233088295-233088317	233088302	5	RNA	GGCCGAGGTCGACTACCGGCNRG	a-CCGcGGTCGACcACCcGCCGG	-	4	1	2	0.00	1.00
Chr2:161210380-161210402	161210397	3	RNA	GGCCGAGGTCGACTACCGGCNRG	t--CGAGGaCatCTtCCGGCTGG	+	5	2	2	0.00	1.00
Chr10:119832362-119832384	119832379	3	RNA	GGCCGAGGTCGACTACCGGCNRG	GcCgGcGGTCGACT-CCGtCCGG	+	4	1	2	0.00	1.00
Chr10:39633642-39633664	39633659	3	RNA	GGCCGAGGTCGACTACCGGCNRG	caaCG--GTCGACTACCacCGGG	+	5	2	2	0.00	1.00
Chr10:120975499-120975521	120975506	5	RNA	GGCCGAGGTCGACTACCGGCNRG	GtCCtcGG--GAtaACCGGCAGG	-	5	2	2	0.00	1.00
Chr5:137632765-137632787	137632772	5	DNA	GGCCGAGGTCGACTACCGG--CNRG	GGtCGAGGTCGAgTcCgaGCCCCGG	-	5	2	2	0.00	1.00
Chr2:213131578-213131600	213131585	5	RNA	GGCCGAGGTCGACTACCGGCNRG	GGaCGAcGgCGACTt--GGCAAG	-	4	2	2	0.00	1.00
Chr10:150008745-150008767	150008762	3	DNA	GGCCGAGGTCGACT--ACCGGCNRG	GaCtGtGGTCGgCTGCACCGGgGGG	+	5	2	2	0.00	1.00
Chr7:157084213-157084235	157084230	3	DNA	GGC--CGAGGTCGACTACCGGCNRG	GGCCTCGgGGgCtACacCCGGCGGG	+	5	2	2	0.00	1.00
Chr9:129870864-129870886	129870881	3	RNA	GGCCGAGGTCGACTACCGGCNRG	GGC--AGGgCGACcAagGcCAAG	+	5	2	2	0.00	1.00
Chr1:248328296-248328318	248328303	5	RNA	GGCCGAGGTCGACTACCGGCNRG	GGCtGAGGgtGcCTtC-GGCAGG	-	5	1	2	0.00	1.00
Chr1:217174659-217174681	217174676	3	RNA	GGCCGAGGTCGACTACCGGCNRG	GGttaAGaTC-ACTACCGGCTGG	+	4	1	2	0.00	1.00
Chr3:146234967-146234989	146234984	3	RNA	GGCCGAGGTCGACTACCGGCNRG	caCCaAGtTCGcCT-CCGGCCAG	+	5	1	2	0.00	1.00
Chr5:20792377-20792399	20792394	3	RNA	GGCCGAGGTCGACTACCGGCNRG	cG--GAGtTCGACcACCGGtGAG	+	4	2	2	0.00	1.00
Chr10:129515239-129515261	129515246	5	RNA	GGCCGAGGTCGACTACCGGCNRG	GGCgGAG--CcAagACCGGtAAG	-	5	2	2	0.00	1.00
Chr4:134692008-134692030	134692015	5	RNA	GGCCGAGGTCGACTACCGGCNRG	aGggGAGaTgGACTAC-GGCTAG	-	5	1	2	0.00	1.00
Chr3:231653432-231653454	231653439	5	RNA	GGCCGAGGTCGACTACCGGCNRG	GcCgGAGccCGAC--CCGtCAAG	-	5	2	2	0.00	1.00
Chr6:139056990-139057012	139056997	5	RNA	GGCCGAGGTCGACTACCGGCNRG	GaCgGAG-TCGACaACgaGCGGG	-	5	1	2	0.00	1.00
Chr2:36345316-36345338	36345323	5	RNA	GGCCGAGGTCGACTACCGGCNRG	tGCCGAGGTCG--TcCCtGaTAG	-	4	2	2	0.00	1.00
Chr6:159092141-159092163	159092148	5	DNA	GGCCGA-GGTCGACTACCGGCNRG	GGCgGACGGTCcgCgACCGaCCAG	-	5	1	2	0.00	1.00
Chr6:48756286-48756308	48756303	3	RNA	GGCCGAGGTCGACTACCGGCNRG	G-aCGgGGTCGACaACgaGCGGG	+	5	1	2	0.00	1.00
Chr1:203648489-203648511	203648496	5	RNA	GGCCGAGGTCGACTACCGGCNRG	GGCCG-GGTCGtCcACtGGCGGG	-	3	1	2	0.00	1.00
Chr6:67437711-67437733	67437718	5	RNA	GGCCGAGGTCGACTACCGGCNRG	GG--GAaGTCGACTgtCtcCAAG	-	5	2	2	0.00	1.00
Chr3:120021351-120021373	120021358	5	RNA	GGCCGAGGTCGACTACCGGCNRG	GtCCG--GTgcACcACCGGaTGG	-	5	2	2	0.00	1.00
Chr8:135492607-135492629	135492624	3	RNA	GGCCGAGGTCGACTACCGGCNRG	GGCCaAGGcC--CTgCgtGCGAG	+	5	2	2	0.00	1.00
Chr6:70495846-70495868	70495863	3	RNA	GGCCGAGGTCGACTACCGGCNRG	GcCCGAGGcCGA--ACtcGgTGG	+	5	2	2	0.00	1.00
Chr7:77638660-77638682	77638677	3	RNA	GGCCGAGGTCGACTACCGGCNRG	GGCtGAtGTCaAgTACt--CAGG	+	5	2	2	0.00	1.00
Chr1:301206840-301206862	301206847	5	RNA	GGCCGAGGTCGACTACCGGCNRG	GtttGAGGT--ACgACCGGaGAG	-	5	2	2	0.00	1.00
Chr10:130123197-130123219	130123214	3	RNA	GGCCGAGGTCGACTACCGGCNRG	GGCgGtG--CGcCcACCcGCAGG	+	5	2	2	0.00	1.00
Chr9:119632212-119632234	119632229	3	RNA	GGCCGAGGTCGACTACCGGCNRG	GGgCG-GGcCGACctCCGGCCGG	+	4	1	2	0.00	1.00
Chr7:169088060-169088082	169088077	3	RNA	GGCCGAGGTCGACTACCGGCNRG	G-aCGgGGTCGACgACgaGCGGG	+	5	1	2	0.00	1.00
Chr5:158274315-158274337	158274322	5	RNA	GGCCGAGGTCGACTACCGGCNRG	ccCCGgGGTCGA--AaCGcCGAG	-	5	2	2	0.00	1.00
Chr8:164180904-164180926	164180921	3	RNA	GGCCGAGGTCGACTACCGGCNRG	atCCcAtGTCGA--ACgGGCAAG	+	5	2	2	0.00	1.00
Chr5:12129976-12129998	12129983	5	RNA	GGCCGAGGTCGACTACCGGCNRG	t--CGAGaTCGACcACgcGCGGG	-	5	2	2	0.00	1.00
Chr1:27877816-27877838	27877833	3	RNA	GGCCGAGGTCGACTACCGGCNRG	a--CGgGGTCGACgACgaGCGGG	+	5	2	2	0.00	1.00
Chr7:148639167-148639189	148639184	3	RNA	GGCCGAGGTCGACTACCGGCNRG	GGC--tcGcgGACTACCGtCTGG	+	5	2	2	0.00	1.00
Chr8:26367980-26368002	26367997	3	RNA	GGCCGAGGTCGACTACCGGCNRG	ttCCGAtGTCGAtTA--tGCTGG	+	5	2	2	0.00	1.00
Chr8:152819325-152819347	152819342	3	RNA	GGCCGAGGTCGACTACCGGCNRG	GGCtGAGcgCGgCTg--GGCTGG	+	5	2	2	0.00	1.00
Chr1:102734820-102734842	102734837	3	RNA	GGCCGAGGTCGACTACCGGCNRG	GtCCG--GTgcACcACCGGaCAG	+	5	2	2	0.00	1.00
Chr9:148787260-148787282	148787267	5	DNA	GGCCGAGGTCGACTACCG-GCNRG	GGCCGgcGTCGAgcACCtCGCTGG	-	5	1	2	0.00	1.00
Chr9:136164941-136164963	136164948	5	RNA	GGCCGAGGTCGACTACCGGCNRG	GGCCatGG-CGcCgtCCGGCCGG	-	5	1	2	0.00	1.00
Chr6:46578926-46578948	46578933	5	RNA	GGCCGAGGTCGACTACCGGCNRG	tGgCtAGGTCGtCgA--GGCAGG	-	5	2	2	0.00	1.00
Chr2:166012334-166012356	166012351	3	RNA	GGCCGAGGTCGACTACCGGCNRG	GG--GAGGagGtaTACgGGCAAG	+	5	2	2	0.00	1.00
Chr2:61178642-61178664	61178649	5	RNA	GGCCGAGGTCGACTACCGGCNRG	aGCCGgGGaCGAg--CCGaCGAG	-	5	2	2	0.00	1.00
Chr6:156689741-156689763	156689748	5	RNA	GGCCGAGGTCGACTACCGGCNRG	GGCCc--GTtGAggtCCGGCTGG	-	5	2	2	0.00	1.00
Chr6:157449386-157449408	157449393	5	RNA	GGCCGAGGTCGACTACCGGCNRG	GtCCG-GGTCGACcACCGGCAGG	-	2	1	2	0.00	1.00
Chr2:119055300-119055322	119055307	5	RNA	GGCCGAGGTCGACTACCGGCNRG	aGCCGAGGgCGcCT--CGGgCAG	-	4	2	2	0.00	1.00
Chr6:2318479-2318501	2318486	5	RNA	GGCCGAGGTCGACTACCGGCNRG	GGC--ttGcgGACTACCGGCTGG	-	4	2	2	0.00	1.00
Chr1:212138712-212138734	212138719	5	RNA	GGCCGAGGTCGACTACCGGCNRG	GGCCG-GGTCGtCcACtGGCGGG	-	3	1	2	0.00	1.00
Chr4:65547281-65547303	65547288	5	RNA	GGCCGAGGTCGACTACCGGCNRG	GGaCcAGGTCG--TcCCaGtTGG	-	5	2	2	0.00	1.00
Chr8:120328231-120328253	120328248	3	DNA	G-GCCGAGGTCGACTACCGGCNRG	GCGCCGgcGTCGACTcCCGcCGGG	+	4	1	2	0.00	1.00
Chr8:172420228-172420250	172420245	3	RNA	GGCCGAGGTCGACTACCGGCNRG	GGCCGgaGcC--CTACCcGtCAG	+	5	2	2	0.00	1.00
Chr2:103165038-103165060	103165045	5	RNA	GGCCGAGGTCGACTACCGGCNRG	GGagGAGGTgG--TAgaGGCTAG	-	5	2	2	0.00	1.00
Chr2:104196829-104196851	104196846	3	RNA	GGCCGAGGTCGACTACCGGCNRG	GGaCGAGGcgcACaAC--GCTAG	+	5	2	2	0.00	1.00
Chr9:138983243-138983265	138983250	5	RNA	GGCCGAGGTCGACTACCGGCNRG	GGaCGAcccCGACT-CCGGtTGG	-	5	1	2	0.00	1.00
Chr1:148312942-148312964	148312959	3	RNA	GGCCGAGGTCGACTACCGGCNRG	GGCaGAGG--GgCTcCtcGCCGG	+	5	2	2	0.00	1.00
Chr7:177801759-177801781	177801766	5	RNA	GGCCGAGGTCGACTACCGGCNRG	GatCGAGcaCG--TACCaGCTGG	-	5	2	2	0.00	1.00
Chr4:146949056-146949078	146949073	3	RNA	GGCCGAGGTCGACTACCGGCNRG	GcaCGccGTCG-CTgCCGGCCGG	+	5	1	2	0.00	1.00
Chr2:58318422-58318444	58318429	5	RNA	GGCCGAGGTCGACTACCGGCNRG	GGa-GAGGTtGtCTgCtGGCCAG	-	5	1	2	0.00	1.00
Chr8:91881872-91881894	91881879	5	RNA	GGCCGAGGTCGACTACCGGCNRG	GtCCGAcGaCGACT-CCaaCGGG	-	5	1	2	0.00	1.00
Chr5:29957185-29957207	29957192	5	RNA	GGCCGAGGTCGACTACCGGCNRG	GtCCG--GTgcACcACCGGaTAG	-	5	2	2	0.00	1.00
Chr10:103294100-103294122	103294117	3	RNA	GGCCGAGGTCGACTACCGGCNRG	GGtCGtcGTCG--TACCaaCCGG	+	5	2	2	0.00	1.00
Chr5:123241243-123241265	123241260	3	RNA	GGCCGAGGTCGACTACCGGCNRG	GGCCGAG--CcAaTgCtGGtGAG	+	5	2	2	0.00	1.00
Chr5:116131541-116131563	116131558	3	DNA	GGCCGAGGTCG-ACTACCGGCNRG	GGCgGcGGTgGCtCTAaCGGCGAG	+	5	1	2	0.00	1.00
Chr1:165772361-165772383	165772378	3	DNA	GGC-CGAGGTCGACTACCGGCNRG	GcCTCGgGGaCGtCTtCCGGCTGG	+	5	1	2	0.00	1.00
Chr5:167857191-167857213	167857198	5	RNA	GGCCGAGGTCGACTACCGGCNRG	GGCCGtGGTC--CgggCGGgAGG	-	5	2	2	0.00	1.00
Chr3:174368949-174368971	174368956	5	DNA	GGCCGAGGTCGAC-TACCGGCNRG	GGCCGAGcTCGgCATgggGGCAGG	-	5	1	2	0.00	1.00
Chr7:41034208-41034230	41034215	5	RNA	GGCCGAGGTCGACTACCGGCNRG	GGCCGAGGatGcCT-CgGGCTAG	-	4	1	2	0.00	1.00
Chr4:171838956-171838978	171838963	5	X	GGCCGAGGTCGACTACCGGCNRG	GGCCGtGGcCGAgTAgaGGCGGG	-	5	0	2	0.00	1.00
Chr4:142088464-142088486	142088481	3	RNA	GGCCGAGGTCGACTACCGGCNRG	GGCgacGcaCGACTACC--CGAG	+	5	2	2	0.00	1.00
Chr8:81559583-81559605	81559590	5	RNA	GGCCGAGGTCGACTACCGGCNRG	GGtCGgaaTCGtCT--CGGCAGG	-	5	2	2	0.00	1.00
Chr10:149625639-149625661	149625646	5	RNA	GGCCGAGGTCGACTACCGGCNRG	cGCtGAGGTCGAgcA--GGaGAG	-	5	2	2	0.00	1.00
Chr10:123319772-123319794	123319779	5	RNA	GGCCGAGGTCGACTACCGGCNRG	GGCaGAGGgCG--cACCGGaCAG	-	4	2	2	0.00	1.00
Chr4:13670781-13670803	13670798	3	RNA	GGCCGAGGTCGACTACCGGCNRG	GGC--AGGcCGAgcACCGGCCAG	+	3	2	2	0.00	1.00
Chr10:78120118-78120140	78120135	3	RNA	GGCCGAGGTCGACTACCGGCNRG	G-aCGgGGTCGACgACgaGCGGG	+	5	1	2	0.00	1.00
Chr7:4021755-4021777	4021762	5	X	GGCCGAGGTCGACTACCGGCNRG	GGCCGAGcTCGAgTtatGGCCGG	-	5	0	2	0.00	1.00
Chr7:93049525-93049547	93049532	5	RNA	GGCCGAGGTCGACTACCGGCNRG	G-aCGgGGTCGACaACgaGCGGG	-	5	1	2	0.00	1.00
Chr1:24667508-24667530	24667525	3	RNA	GGCCGAGGTCGACTACCGGCNRG	GGCCGAGG-CGggTAgCtcCTGG	+	5	1	2	0.00	1.00
Chr3:80034544-80034566	80034551	5	RNA	GGCCGAGGTCGACTACCGGCNRG	GGCaGgGcgCGACT-CCGGtGAG	-	5	1	2	0.00	1.00
Chr7:46462110-46462132	46462127	3	X	GGCCGAGGTCGACTACCGGCNRG	GcCCGAGcTgGACTtCgGGCGAG	+	5	0	2	0.00	1.00
Chr8:130883778-130883800	130883795	3	RNA	GGCCGAGGTCGACTACCGGCNRG	a--CGgGGTCGACgACgaGCGGG	+	5	2	2	0.00	1.00
Chr1:94436136-94436158	94436153	3	RNA	GGCCGAGGTCGACTACCGGCNRG	GGCCGAaGcCGA--AgCtaCCGG	+	5	2	2	0.00	1.00
Chr4:76727200-76727222	76727217	3	RNA	GGCCGAGGTCGACTACCGGCNRG	tGCtGAGGTgGAtTACC--tGAG	+	5	2	2	0.00	1.00
Chr6:121585436-121585458	121585453	3	RNA	GGCCGAGGTCGACTACCGGCNRG	tGCgGAGc-CGACgACCaGCCGG	+	5	1	2	0.00	1.00
Chr1:288551640-288551662	288551657	3	RNA	GGCCGAGGTCGACTACCGGCNRG	GGt-GtGGTCGAggACtGGCGGG	+	5	1	2	0.00	1.00
Chr7:173363549-173363571	173363556	5	RNA	GGCCGAGGTCGACTACCGGCNRG	GGtgGcGG-CGgCTACCGGaGGG	-	5	1	2	0.00	1.00
Chr4:147342829-147342851	147342846	3	RNA	GGCCGAGGTCGACTACCGGCNRG	aGCCGAGtT-GAacAgCGGCTGG	+	5	1	2	0.00	1.00
Chr5:131149495-131149517	131149502	5	RNA	GGCCGAGGTCGACTACCGGCNRG	GGCgGcGGgCGAC-ggCGGCGGG	-	5	1	2	0.00	1.00

Chr10:129019630-129019652	129019647	3	RNA	GGCCGAGGTCGACTACCGGCNRG	G-aCGgGGTCGACaACgaGCGGG	+	5	1	2	0.00	1.00
Chr10:123327514-123327536	123327531	3	RNA	GGCCGAGGTCGACTACCGGCNRG	GGCCGAGGTaGACgAa-GtaGAG	+	5	1	2	0.00	1.00
Chr10:57278910-57278932	57278917	5	RNA	GGCCGAGGTCGACTACCGGCNRG	GGCCGAGcgCGA--AggGGgGGG	-	5	2	2	0.00	1.00
Chr2:160691875-160691897	160691892	3	RNA	GGCCGAGGTCGACTACCGGCNRG	GG--GgGaTCGACccCCaGCCGG	+	5	2	2	0.00	1.00
Chr4:139741790-139741812	139741807	3	RNA	GGCCGAGGTCGACTACCGGCNRG	aGaCGAGcTCGACT-CtGGtTGG	+	5	1	2	0.00	1.00
Chr1:141335412-141335434	141335419	5	RNA	GGCCGAGGTCGACTACCGGCNRG	GGCCGAGG--GAtggCCcaCGAG	-	5	2	2	0.00	1.00
Chr5:19999870-19999892	19999887	3	RNA	GGCCGAGGTCGACTACCGGCNRG	G-aCGgGGTCGACaACgaGCGGG	+	5	1	2	0.00	1.00
Chr5:154323098-154323120	154323105	5	RNA	GGCCGAGGTCGACTACCGGCNRG	GGC--ttGcgGACTACaGGCTGG	-	5	2	2	0.00	1.00
Chr2:115844734-115844756	115844741	5	RNA	GGCCGAGGTCGACTACCGGCNRG	GGCCaAGcgCG--gACCGtCCGG	-	5	2	2	0.00	1.00
Chr4:165533463-165533485	165533470	5	RNA	GGCCGAGGTCGACTACCGGCNRG	GGCgGAGG--GgCagCCGGCCGG	-	4	2	2	0.00	1.00
Chr2:45333383-45333405	45333400	3	RNA	GGCCGAGGTCGACTACCGGCNRG	G-aCGAGGTCGACaACgaGCGGG	+	4	1	2	0.00	1.00
Chr1:265313976-265313998	265313993	3	RNA	GGCCGAGGTCGACTACCGGCNRG	aGCCGAGtTCcACTAC-GGaGAG	+	4	1	2	0.00	1.00
Chr9:26400028-26400050	26400045	3	RNA	GGCCGAGGTCGACTACCGGCNRG	GtCCG--GTgcACcACCGGaTAG	+	5	2	2	0.00	1.00
Chr7:84852488-84852510	84852505	3	RNA	GGCCGAGGTCGACTACCGGCNRG	GGCCGAGtaCG--TtgCcGCAGG	+	5	2	2	0.00	1.00
Chr10:94000556-94000578	94000573	3	RNA	GGCCGAGGTCGACTACCGGCNRG	GaCgaAGGTgGACTA--GtCAGG	+	5	2	2	0.00	1.00
Chr10:66150444-66150466	66150451	5	RNA	GGCCGAGGTCGACTACCGGCNRG	tcCCtAGGTC--CgAtCGGCCAG	-	5	2	2	0.00	1.00
Chr1:117161254-117161276	117161261	5	RNA	GGCCGAGGTCGACTACCGGCNRG	GGCCGgG--CcACcAgCGGaAGG	-	5	2	2	0.00	1.00
Chr2:86752278-86752300	86752295	3	RNA	GGCCGAGGTCGACTACCGGCNRG	GG--GcGGTCGtggACCGtCGAG	+	5	2	2	0.00	1.00
Chr1:229450438-229450460	229450455	3	DNA	GGCCGAGGT--CGACTACCGGCNRG	GGaCGAGGTTCCGACcAagGGCGGG	+	4	2	2	0.00	1.00
Chr3:93391071-93391093	93391078	5	RNA	GGCCGAGGTCGACTACCGGCNRG	GaCaG-GGTCGACgACaaGCGGG	-	5	1	2	0.00	1.00
Chr2:138645561-138645583	138645578	3	RNA	GGCCGAGGTCGACTACCGGCNRG	GGCgGtGG--GAtcgCCGGCGAG	+	5	2	2	0.00	1.00
Chr2:101562640-101562662	101562657	3	RNA	GGCCGAGGTCGACTACCGGCNRG	GGC-GAaGTtGgCTACCtGgAGG	+	5	1	2	0.00	1.00
Chr8:15152296-15152318	15152303	5	RNA	GGCCGAGGTCGACTACCGGCNRG	GGaCcAGGTCG--TcCCaGtTGG	-	5	2	2	0.00	1.00
Chr3:178007838-178007860	178007855	3	RNA	GGCCGAGGTCGACTACCGGCNRG	GGC-GAGcaCGtagACCGGCCAG	+	5	1	2	0.00	1.00
Chr5:204965973-204965995	204965990	3	RNA	GGCCGAGGTCGACTACCGGCNRG	c--CGAGaTaGACTAtCaGCTAG	+	5	2	2	0.00	1.00
Chr2:225889857-225889879	225889864	5	RNA	GGCCGAGGTCGACTACCGGCNRG	aGCCatGGcCGACT-CaGGCGGG	-	5	1	2	0.00	1.00
Chr9:142788515-142788537	142788522	5	RNA	GGCCGAGGTCGACTACCGGCNRG	GG--GAGaTCGAtctCCGtCGGG	-	5	2	2	0.00	1.00
Chr5:4062455-4062477	4062462	5	RNA	GGCCGAGGTCGACTACCGGCNRG	GGCgtA--TCtgCTgCCGGCGGG	-	5	2	2	0.00	1.00
Chr1:55835919-55835941	55835926	5	DNA	GGCCGAGG-TCGACTACCGGCNRG	cGCCGAGaCTCGcCTgtCGGCTAG	-	5	1	2	0.00	1.00
Chr2:14263116-14263138	14263133	3	RNA	GGCCGAGGTCGACTACCGGCNRG	GtgCcAGGTCG-CTgCCaGCAGG	+	5	1	2	0.00	1.00
Chr5:199934854-199934876	199934861	5	RNA	GGCCGAGGTCGACTACCGGCNRG	GtCCG--GTgcACcACCGGaCAG	-	5	2	2	0.00	1.00
Chr1:197047925-197047947	197047942	3	RNA	GGCCGAGGTCGACTACCGGCNRG	G-aCGAcGaCGACgACCtGCTGG	+	5	1	2	0.00	1.00
Chr1:32192321-32192343	32192338	3	RNA	GGCCGAGGTCGACTACCGGCNRG	GaCCGgGGggGA--ACCGGtCGG	+	5	2	2	0.00	1.00
Chr5:93937940-93937962	93937947	5	RNA	GGCCGAGGTCGACTACCGGCNRG	GGCgttGGaCGACTAC--GCCGG	-	4	2	2	0.00	1.00
Chr6:107757665-107757687	107757682	3	RNA	GGCCGAGGTCGACTACCGGCNRG	GGCCtAGGcCGA--AattGCTAG	+	5	2	2	0.00	1.00
Chr1:112271048-112271070	112271065	3	RNA	GGCCGAGGTCGACTACCGGCNRG	GG--GAGGaCGAgcACCcGtAGG	+	5	2	2	0.00	1.00
Chr2:127387923-127387945	127387940	3	RNA	GGCCGAGGTCGACTACCGGCNRG	GGgCGgGGTtGgCT-CCGGgAGG	+	5	1	2	0.00	1.00
Chr9:7940908-7940930	7940925	3	RNA	GGCCGAGGTCGACTACCGGCNRG	GGgCGAGcTCGA--ACgGGCTAG	+	3	2	2	0.00	1.00
Chr1:38747461-38747483	38747468	5	RNA	GGCCGAGGTCGACTACCGGCNRG	GGCgGAGGTCGAgTAg-GGgCAG	-	4	1	2	0.00	1.00
Chr9:152449405-152449427	152449422	3	RNA	GGCCGAGGTCGACTACCGGCNRG	GGCgGAGG--GACagCtGtCGGG	+	5	2	2	0.00	1.00
Chr1:22495432-22495454	22495449	3	RNA	GGCCGAGGTCGACTACCGGCNRG	GcCCGAGGgCaACcACC--gTGG	+	5	2	2	0.00	1.00
Chr6:54009023-54009045	54009040	3	RNA	GGCCGAGGTCGACTACCGGCNRG	GGCCGgGGTgGA-TctCGcCGAG	+	5	1	2	0.00	1.00
Chr7:137588483-137588505	137588490	5	DNA	GGC--CGAGGTCGACTACCGGCNRG	GGCACCGccGTCtAgTAgCGGCTGG	-	5	2	2	0.00	1.00
Chr2:124122825-124122847	124122842	3	RNA	GGCCGAGGTCGACTACCGGCNRG	GGt-GAGGTCGACgACatGaTGG	+	5	1	2	0.00	1.00
Chr2:163194446-163194468	163194463	3	RNA	GGCCGAGGTCGACTACCGGCNRG	GGCCGAGtaCG--TtgCcGCAAG	+	5	2	2	0.00	1.00
Chr5:13561317-13561339	13561334	3	RNA	GGCCGAGGTCGACTACCGGCNRG	G-aCGgGGTCGACgACgaGCGGG	+	5	1	2	0.00	1.00
Chr4:208437914-208437936	208437931	3	RNA	GGCCGAGGTCGACTACCGGCNRG	GGatGAGGagGACT--CGGaCGG	+	5	2	2	0.00	1.00
Chr3:232570199-232570221	232570216	3	RNA	GGCCGAGGTCGACTACCGGCNRG	GGa-GAGGgaGACgAtCGGCTGG	+	5	1	2	0.00	1.00
Chr4:173354999-173355021	173355016	3	RNA	GGCCGAGGTCGACTACCGGCNRG	GtCCG--GTgcACcACCGGaCAG	+	5	2	2	0.00	1.00
Chr5:2760202-2760224	2760209	5	RNA	GGCCGAGGTCGACTACCGGCNRG	GGCCGAcagC--CcACCcGCCGG	-	5	2	2	0.00	1.00
Chr3:214825383-214825405	214825400	3	RNA	GGCCGAGGTCGACTACCGGCNRG	GGtCG-GGTgcACTcCCGcCAGG	+	5	1	2	0.00	1.00
Chr9:77697514-77697536	77697531	3	RNA	GGCCGAGGTCGACTACCGGCNRG	GGgC-AGGcCGAgcACCGGCCGG	+	4	1	2	0.00	1.00
Chr8:95710211-95710233	95710218	5	RNA	GGCCGAGGTCGACTACCGGCNRG	G-aCGgGGTCGACaACgaGCGGG	-	5	1	2	0.00	1.00
Chr10:120745055-120745077	120745062	5	RNA	GGCCGAGGTCGACTACCGGCNRG	GGC--AGacCGAgctCCGGCCGG	-	5	2	2	0.00	1.00
Chr9:53478402-53478424	53478409	5	RNA	GGCCGAGGTCGACTACCGGCNRG	GctCGAG--tGACTcCaGGCGAG	-	5	2	2	0.00	1.00
Chr1:86156470-86156492	86156477	5	RNA	GGCCGAGGTCGACTACCGGCNRG	GG--GAGGgCGcCgACgGGaGGG	-	5	2	2	0.00	1.00
Chr7:159036223-159036245	159036240	3	RNA	GGCCGAGGTCGACTACCGGCNRG	cGCCaAacTCtAC--CCGGCCGG	+	5	2	2	0.00	1.00
Chr7:72618723-72618745	72618730	5	DNA	GGC-CGAGGTCGACTACCGGCNRG	GcCTCGAGGaCGtCTtCCGGCTGG	-	4	1	2	0.00	1.00
Chr1:306256510-306256532	306256517	5	DNA	GGCCGAGG-TCGACTACCGGCNRG	GGCtGAaGATCGACTACatGtTGG	-	5	1	2	0.00	1.00
Chr3:195835568-195835590	195835585	3	RNA	GGCCGAGGTCGACTACCGGCNRG	GGCgGAaGTCG--TtCgGcCGAG	+	5	2	2	0.00	1.00
Chr7:181053709-181053731	181053726	3	RNA	GGCCGAGGTCGACTACCGGCNRG	GGagGAGGTCGACgAC-GagCGG	+	5	1	2	0.00	1.00
Chr1:200694720-200694742	200694737	3	RNA	GGCCGAGGTCGACTACCGGCNRG	GtCCG--GaaGAaTACCGGaAAG	+	5	2	2	0.00	1.00
Chr1:10504032-10504054	10504049	3	X	GGCCGAGGTCGACTACCGGCNRG	GGCCGAGaTCccCTggCGGCGGG	+	5	0	2	0.00	1.00
Chr5:172702656-172702678	172702663	5	RNA	GGCCGAGGTCGACTACCGGCNRG	caCCaAGtTCGcCT-CCGGCCAG	-	5	1	2	0.00	1.00
Chr2:64885770-64885792	64885777	5	RNA	GGCCGAGGTCGACTACCGGCNRG	cGCCGAcGT-GACcgCCGaCAAG	-	5	1	2	0.00	1.00
Chr7:68005428-68005450	68005445	3	RNA	GGCCGAGGTCGACTACCGGCNRG	GcCCGAGGgCaACcACC--aTGG	+	5	2	2	0.00	1.00
Chr1:266544125-266544147	266544132	5	RNA	GGCCGAGGTCGACTACCGGCNRG	GGC-aAGGTgGcaTACCGGCTGG	-	4	1	2	0.00	1.00
Chr4:228259976-228259998	228259993	3	RNA	GGCCGAGGTCGACTACCGGCNRG	tGgCGAGGTCGAggACC--tTGG	+	5	2	2	0.00	1.00
Chr8:106375413-106375435	106375430	3	RNA	GGCCGAGGTCGACTACCGGCNRG	c--CGcGGcCGAacACCGGCAAG	+	5	2	2	0.00	1.00
Chr1:151542562-151542584	151542579	3	RNA	GGCCGAGGTCGACTACCGGCNRG	GGCCt--GcCaACTtCCGGgCAG	+	5	2	2	0.00	1.00
Chr2:17800002-17800024	17800009	5	RNA	GGCCGAGGTCGACTACCGGCNRG	GtCCG--GTgcACcACCGGaCAG	-	5	2	2	0.00	1.00
Chr5:93820288-93820310	93820305	3	DNA	G--GCCGAGGTCGACTACCGGCNRG	GTAGCCGAGGcCGACctCaGGCGAG	+	4	2	2	0.00	1.00
Chr8:146627299-146627321	146627306	5	DNA	GGC--CGAGGTCGACTACCGGCNRG	cGCTTCGgGGaCGtCTtCCGGCTGG	-	5	2	2	0.00	1.00
Chr4:228197456-228197478	228197463	5	RNA	GGCCGAGGTCGACTACCGGCNRG	GGCtcAcG--GACTACCtGCTGG	-	4	2	2	0.00	1.00
Chr2:152700927-152700949	152700944	3	RNA	GGCCGAGGTCGACTACCGGCNRG	GaCCGAGG-CGACgACaaGaAGG	+	5	1	2	0.00	1.00
Chr1:251528727-251528749	251528734	5	RNA	GGCCGAGGTCGACTACCGGCNRG	GaCCG-GGTCGACaACgaGCGGG	-	4	1	2	0.00	1.00
Chr3:86293443-86293465	86293450	5	RNA	GGCCGAGGTCGACTACCGGCNRG	GGaCGAGGTCG--ggatGGCCGG	-	5	2	2	0.00	1.00
Chr4:82435430-82435452	82435447	3	RNA	GGCCGAGGTCGACTACCGGCNRG	tGCaGgGcTCGgCTA--GGCTGG	+	5	2	2	0.00	1.00
Chr9:88565741-88565763	88565748	5	X	GGCCGAGGTCGACTACCGGCNRG	GaCCGAGGggGAaTACCcGCTGG	-	5	0	2	0.00	1.00
Chr2:193040511-193040533	193040518	5	RNA	GGCCGAGGTCGACTACCGGCNRG	GGCCGAGGcCGAgccCC--tGGG	-	5	2	2	0.00	1.00
Chr8:62989458-62989480	62989475	3	RNA	GGCCGAGGTCGACTACCGGCNRG	cGgtGAGcTtG--TACCGGCCGG	+	5	2	2	0.00	1.00
Chr6:18764727-18764749	18764734	5	RNA	GGCCGAGGTCGACTACCGGCNRG	GtCCG--GTgGcCcACCGGaTAG	-	5	2	2	0.00	1.00
Chr8:4558797-4558819	4558804	5	RNA	GGCCGAGGTCGACTACCGGCNRG	GaCC-AGGTCttCTcCCaGCAGG	-	5	1	2	0.00	1.00
Chr1:82134215-82134237	82134222	5	RNA	GGCCGAGGTCGACTACCGGCNRG	GGC--tcGcgGACTACCGGCTGG	-	4	2	2	0.00	1.00
Chr7:125319594-125319616	125319601	5	RNA	GGCCGAGGTCGACTACCGGCNRG	GcaCGAGGgCGACTt--GGCAAG	-	4	2	2	0.00	1.00
Chr4:99787853-99787875	99787870	3	RNA	GGCCGAGGTCGACTACCGGCNRG	GGCCGAGGgC-ACctCgGGCTGG	+	4	1	2	0.00	1.00
Chr1:276024506-276024528	276024523	3	RNA	GGCCGAGGTCGACTACCGGCNRG	tGgCtgcG--GACTACCGGCTGG	+	5	2	2	0.00	1.00
Chr3:30024630-30024652	30024637	5	X	GGCCGAGGTCGACTACCGGCNRG	GGCCGAGGcCGAgctCgGGCGAG	-	5	0	2	0.00	1.00
Chr4:127812308-127812330	127812315	5	RNA	GGCCGAGGTCGACTACCGGCNRG	GGCaGAGGgtG--cACCGGaCAG	-	5	2	2	0.00	1.00
Chr3:84602825-84602847	84602842	3	RNA	GGCCGAGGTCGACTACCGGCNRG	GGgCGAGcTCGtC-ggCGGCGAG	+	5	1	2	0.00	1.00
Chr8:5994399-5994421	5994416	3	RNA	GGCCGAGGTCGACTACCGGCNRG	G-aCGgGGTCGACaACgaGCGGG	+	5	1	2	0.00	1.00
Chr4:144602546-144602568	144602563	3	X	GGCCGAGGTCGACTACCGGCNRG	GGCtGAGacCGACTtCgGGCGAG	+	5	0	2	0.00	1.00
Chr2:59678565-59678587	59678582	3	RNA	GGCCGAGGTCGACTACCGGCNRG	GGC--ttGcgGACTACCGGCTGG	+	4	2	2	0.00	1.00
Chr8:63187388-63187410	63187405	3	DNA	GGCCGAGGTCGACTA--CCGGCNRG	GGgCGAGtcgGACaACTCCGGCGAG	+	5	2	2	0.00	1.00
Chr7:33714278-33714300	33714285	5	DNA	GGC-CGAGGTCGACTACCGGCNRG	aGCTCGAatTCtACcACCGGCTGG	-	5	1	2	0.00	1.00
Chr3:185874988-185875010	185874995	5	RNA	GGCCGAGGTCGACTACCGGCNRG	GtCCG--GTgcACcACCGGaCAG	-	5	2	2	0.00	1.00
Chr5:124273353-124273375	124273370	3	DNA	GGCCGAGGTCGACTACC--GGCNRG	cGaCGAGGgCGACgACaGTGGCAAG	+	5	2	2	0.00	1.00
Chr2:242848948-242848970	242848965	3	RNA	GGCCGAGGTCGACTACCGGCNRG	GGC--ttGTgGAtTACCGGCTGG	+	4	2	2	0.00	1.00
Chr2:89511218-89511240	89511235	3	RNA	GGCCGAGGTCGACTACCGGCNRG	tGCCGAtGgCGctT--CGGCCAG	+	5	2	2	0.00	1.00
Chr4:167507325-167507347	167507342	3	RNA	GGCCGAGGTCGACTACCGGCNRG	GGgaGAGaTCaACT-CgGGCAAG	+	5	1	2	0.00	1.00
Chr9:153190486-153190508	153190503	3	DNA	GGCCGAGGTCGACTACCGG--CNRG	GGCCGAGGcCGAgTcCgaGCCCTGG	+	5	2	2	0.00	1.00
Chr4:123629510-123629532	123629527	3	DNA	GGCCGAGGTCGACTACC--GGCNRG	GcCCGAGGgCaACcACCGTGGCCAG	+	4	2	2	0.00	1.00
Chr1:216585878-216585900	216585885	5	RNA	GGCCGAGGTCGACTACCGGCNRG	GGC--AGGctGAgcACCGGCCGG	-	4	2	2	0.00	1.00
Chr1:192014236-192014258	192014253	3	RNA	GGCCGAGGTCGACTACCGGCNRG	GGaCG--GTCGgCgACgtGCGGG	+	5	2	2	0.00	1.00
Chr4:52822882-52822904	52822889	5	RNA	GGCCGAGGTCGACTACCGGCNRG	G--CGAGGgCGcggAgCGGCTGG	-	5	2	2	0.00	1.00
Chr5:15475695-15475717	15475712	3	RNA	GGCCGAGGTCGACTACCGGCNRG	aGCCGAGGgtGcCT-CgGGCCGG	+	5	1	2	0.00	1.00
Chr9:113739620-113739642	113739627	5	RNA	GGCCGAGGTCGACTACCGGCNRG	GtCCG--GaaGAgTACCGGaAAG	-	5	2	2	0.00	1.00
Chr10:58567884-58567906	58567891	5	RNA	GGCCGAGGTCGACTACCGGCNRG	GGC--AGGcCGAgcgCCGGCCGG	-	4	2	2	0.00	1.00
Chr7:21748109-21748131	21748116	5	RNA	GGCCGAGGTCGACTACCGGCNRG	GGCCaAGGTCa--TcggGGCAGG	-	5	2	2	0.00	1.00
Chr1:160337997-160338019	160338004	5	RNA	GGCCGAGGTCGACTACCGGCNRG	acCCaAGGTCGA--AaCGGaCGG	-	5	2	2	0.00	1.00
Chr3:151701046-151701068	151701053	5	X	GGCCGAGGTCGACTACCGGCNRG	GGCCGAGGTCGAgctCgaGCGAG	-	5	0	2	0.00	1.00
Chr8:162144561-162144583	162144578	3	RNA	GGCCGAGGTCGACTACCGGCNRG	aGaCGAtGTCGACa--CaGCAAG	+	5	2	2	0.00	1.00
Chr7:106843587-106843609	106843594	5	RNA	GGCCGAGGTCGACTACCGGCNRG	GGCgaActTCGgCTAC--GCGGG	-	5	2	2	0.00	1.00
Chr4:139754924-139754946	139754941	3	RNA	GGCCGAGGTCGACTACCGGCNRG	GGCCaAcG--GgCTACtGGgTGG	+	5	2	2	0.00	1.00
Chr3:4244332-4244354	4244339	5	RNA	GGCCGAGGTCGACTACCGGCNRG	G-CCtcGGgCGAgTACCaGCAAG	-	5	1	2	0.00	1.00
Chr5:35326229-35326251	35326236	5	DNA	GGCCGAGGTCGACTA--CCGGCNRG	ctCCtAGGcCGACcAATCCGGCAGG	-	5	2	2	0.00	1.00
Chr4:165114006-165114028	165114013	5	RNA	GGCCGAGGTCGACTACCGGCNRG	GGCgaAGcTCatCT--CGGCAAG	-	5	2	2	0.00	1.00
Chr1:266477269-266477291	266477286	3	DNA	GGCCGAGGTCGACTACCGG--CNRG	GGCCGAGGTCGAgTcCgaGCCCTGG	+	4	2	2	0.00	1.00
Chr10:120789913-120789935	120789920	5	RNA	GGCCGAGGTCGACTACCGGCNRG	GGCgGAGGTCG--TtCgGcaGAG	-	5	2	2	0.00	1.00
Chr5:117396773-117396795	117396790	3	RNA	GGCCGAGGTCGACTACCGGCNRG	GGC--AGGcCaAgctCCGGCCGG	+	5	2	2	0.00	1.00
Chr8:162200504-162200526	162200511	5	RNA	GGCCGAGGTCGACTACCGGCNRG	GGC--tcGcgGACTACCGcCTAG	-	5	2	2	0.00	1.00
Chr8:81361636-81361658	81361653	3	RNA	GGCCGAGGTCGACTACCGGCNRG	GGC--tcGcaGACTACCGcCTGG	+	5	2	2	0.00	1.00
Chr4:100294488-100294510	100294505	3	RNA	GGCCGAGGTCGACTACCGGCNRG	aG--GAGGTCGACTtggcGCTGG	+	5	2	2	0.00	1.00
Chr2:113204267-113204289	113204284	3	RNA	GGCCGAGGTCGACTACCGGCNRG	GGgCGAGGgCGgC-gCCGGtCGG	+	5	1	2	0.00	1.00
Chr5:148949111-148949133	148949118	5	RNA	GGCCGAGGTCGACTACCGGCNRG	GGa--AGGcCGAtgACaGGCGGG	-	5	2	2	0.00	1.00
Chr5:170139479-170139501	170139486	5	DNA	GGCCGAGGTCGACTA--CCGGCNRG	ctCCtAGGcCGACcAATCCGGCAGG	-	5	2	2	0.00	1.00

Chr1:121846086-121846108	121846093	5	RNA	GGCCGAGGTCGACTACCGGCNRG	GGCtGAGaTt-AgaACCGGCGGG	-	5	1	2	0.00	1.00
Chr7:156838989-156839011	156839006	3	RNA	GGCCGAGGTCGACTACCGGCNRG	caCCaAGtTCGcCT-CCGGCCAG	+	5	1	2	0.00	1.00
Chr8:15436603-15436625	15436620	3	RNA	GGCCGAGGTCGACTACCGGCNRG	GGgCG-GGcCtACctCCGGCCGG	+	5	1	2	0.00	1.00
Chr7:88111977-88111999	88111994	3	DNA	GGCCGAGGTCGA-CTACCGGCNRG	aGCCGAGGcCGATCgACCGatGAG	+	5	1	2	0.00	1.00
Chr1:106347042-106347064	106347049	5	RNA	GGCCGAGGTCGACTACCGGCNRG	GGC--AGGaCGAaTAaaGaCCGG	-	5	2	2	0.00	1.00
Chr7:165223996-165224018	165224013	3	RNA	GGCCGAGGTCGACTACCGGCNRG	G-CCGAGGcCGAgctCgGGCGAG	+	5	1	2	0.00	1.00
Chr3:220370964-220370986	220370981	3	RNA	GGCCGAGGTCGACTACCGGCNRG	GGC--ttGcgGAtTACCGGCTGG	+	5	2	2	0.00	1.00
Chr8:4996049-4996071	4996056	5	RNA	GGCCGAGGTCGACTACCGGCNRG	ccCCGgGGTCGA--AaCGcCGAG	-	5	2	2	0.00	1.00
Chr2:3969609-3969631	3969616	5	RNA	GGCCGAGGTCGACTACCGGCNRG	GGCCGAGGgt-ACctCgGGCTGG	-	5	1	2	0.00	1.00
Chr4:176776870-176776892	176776877	5	DNA	GGCCGAGGTCGACTACCGG--CNRG	GGtCGAGGTCGAgTcCgaGCCCTGG	-	5	2	2	0.00	1.00
Chr8:79847512-79847534	79847519	5	RNA	GGCCGAGGTCGACTACCGGCNRG	GGagGgGaTCGACTA--GGgGAG	-	5	2	2	0.00	1.00
Chr6:137270185-137270207	137270202	3	RNA	GGCCGAGGTCGACTACCGGCNRG	aGCCGAGGgtGcCT-CgGGCAGG	+	5	1	2	0.00	1.00
Chr1:65383212-65383234	65383229	3	RNA	GGCCGAGGTCGACTACCGGCNRG	cGCCGcGGTCGcCat--GGCAGG	+	5	2	2	0.00	1.00
Chr1:302484041-302484063	302484048	5	RNA	GGCCGAGGTCGACTACCGGCNRG	GcCtcAGGTCGAC-ggCGGCGGG	-	5	1	2	0.00	1.00
Chr1:158686361-158686383	158686368	5	RNA	GGCCGAGGTCGACTACCGGCNRG	GGgCGgGGTtGgCT-CCGGgAGG	-	5	1	2	0.00	1.00
Chr4:146127556-146127578	146127573	3	RNA	GGCCGAGGTCGACTACCGGCNRG	GGCgaAcGT-GACaACCGGtAAG	+	5	1	2	0.00	1.00
Chr8:95686187-95686209	95686194	5	RNA	GGCCGAGGTCGACTACCGGCNRG	GGC--tcGcgGACTACCGtCTGG	-	5	2	2	0.00	1.00
Chr6:19132057-19132079	19132074	3	RNA	GGCCGAGGTCGACTACCGGCNRG	GcCgGgGGTCGACTt--GGaGGG	+	5	2	2	0.00	1.00
Chr5:59842963-59842985	59842980	3	RNA	GGCCGAGGTCGACTACCGGCNRG	G--CGAGGaCaACaACCaGaAGG	+	5	2	2	0.00	1.00
Chr4:109179406-109179428	109179423	3	RNA	GGCCGAGGTCGACTACCGGCNRG	GtCCGAGGaC--CTAaCtGCGAG	+	4	2	2	0.00	1.00
Chr7:68498628-68498650	68498645	3	DNA	GGCCGAGGTCGACTACC--GGCNRG	GcCaGAGcTCGgCcACCATGGCCAG	+	5	2	2	0.00	1.00
Chr3:157868846-157868868	157868863	3	RNA	GGCCGAGGTCGACTACCGGCNRG	aGCCcAGGaCG--TACaaGCTGG	+	5	2	2	0.00	1.00
Chr3:43610460-43610482	43610467	5	RNA	GGCCGAGGTCGACTACCGGCNRG	GGC--tcGcaGACTACCGGCTGG	-	4	2	2	0.00	1.00
Chr3:880380-880402	880387	5	RNA	GGCCGAGGTCGACTACCGGCNRG	GtCaGAG-TCGACctCgGGCGAG	-	5	1	2	0.00	1.00
Chr7:12856794-12856816	12856801	5	RNA	GGCCGAGGTCGACTACCGGCNRG	aGCCGtGGT-GgCatCCGGCTGG	-	5	1	2	0.00	1.00
Chr5:18497644-18497666	18497661	3	RNA	GGCCGAGGTCGACTACCGGCNRG	GGC--AGGcCGAgcACCaaCCGG	+	5	2	2	0.00	1.00
Chr8:96805245-96805267	96805262	3	RNA	GGCCGAGGTCGACTACCGGCNRG	GaCCGAGGT-GACgACaaGaAGG	+	5	1	2	0.00	1.00
Chr3:207124871-207124893	207124888	3	RNA	GGCCGAGGTCGACTACCGGCNRG	GGaCcAGGTCG--TcCCaGtTGG	+	5	2	2	0.00	1.00
Chr2:204542876-204542898	204542893	3	RNA	GGCCGAGGTCGACTACCGGCNRG	GGC--tcGcgGACTACCGGCTGG	+	4	2	2	0.00	1.00
Chr4:117792293-117792315	117792300	5	RNA	GGCCGAGGTCGACTACCGGCNRG	G-atGgGGTCGACaACgGGCGGG	-	5	1	2	0.00	1.00

Supplementary Table 5. CLEAVE-seq data and a shortlist of computationally predicted high ranked M2 sites with low number of mismatches and bulges. On-target site is shown on the top. RNA Bulges are shown as boxes, respectively. Mismatches are shown in color boxes.

Site	Computational prediction		CLEAVE-Seq reads	Sequence																							Genomic Location
	Mismatch	*Bulge*	*100nM*	20	19	18	17	16	15	14	13	12	11	10	9	8	7	6	5	4	3	2	1	0	-1	-2	
1	0	0	227	G	G	C	C	G	A	G	G	T	C	G	A	C	T	A	C	C	G	G	C	C	G	G	Chr9:143631773-143631795
2	1	1	0											■			C							C			Chr3:214572069-214572090
3	2	1	0						■						T					T				G			Chr3:216792332-216792353
4	2	1	0		■							G			G									A			Chr5:196093089-196093111
5	2	1	0			A						■				T								G			Chr5:219494224-219494245
6	2	2	0		T				■	■										T				T			Chr7:116040382-116040402
7	3	0	0		C							G							G					A			Chr4:173200547-173200569

Supplementary Table 6 . CLEAVE-seq data and a shortlist of M2 sites validated using MIPS. On-target site is shown on the top. DNA and RNA Bulges are shown as gray and black boxes, respectively. Mismatches are shown in color boxes.

Site	Computational prediction		Sequence																							Genomic Location	Predicted by Cas-OFFinder	Identified in CLEAVE-Seq	Avg Normalized CLEAVE-Seq Read Count
	Mismatch	*Bulge*	20	19	18	17	16	15	14	13	12	11	10	9	8	7	6	5	4	3	2	1	0	-1	-2				
1	0	0	G	G	C	C	G	A	G	G	T	C	G	A	C	T	A	C	C	G	G	C	C	G	G	Chr9:143631773-143631795	Yes	Yes	227.16
2	1	1		■							G												A			Chr3:200475375-200475397	Yes	Yes	48.43
3	1	1				A									■								G			Chr6:166308906-166308927	Yes	Yes	12.21
4	1	1											■										C			Chr3:214572069-214572090	Yes	No	0.00
5	2	1		■				C			G												A			Chr10:5286571-5286593	Yes	Yes	111.10
6	2	1		T				■								C							A			Chr6:157449386-157449407	Yes	Yes	1.00
7	2	1						■						T					T				G			Chr3:216792332-216792353	Yes	No	0.00
8	2	1		■							G			G									A			Chr5:196093089-196093111	Yes	No	0.00
9	2	1			A						■				T								G			Chr5:219494224-219494245	Yes	No	0.00
10	2	2	T		■	■		G															A			Chr5:37860732-37860754	Yes	Yes	529.17
11	2	2		T				■	■										T				T			Chr7:116040382-116040402	Yes	No	0.00
12	3	0		C							G							G					A			Chr4:173200547-173200569	Yes	No	0.00
13	3	1		■					C		G			G									A			Chr1:5942859-5942881	Yes	Yes	4.41
14	3	1					A				g									A	C		G			Chr10:39633639-39633661	Yes	Yes	1.00
15	3	1				A					A		■						A				A			Chr2:103727824-103727845	Yes	No	0.00
16	3	1						c					A		T					C			G			Chr3:54133373-54133396	Yes	No	0.00
17	3	1		■								A		G							A		A			Chr6:28373069-28373091	Yes	No	0.00
18	3	2		A		g	T			A													G			Chr7:173232468-173232490	Yes	Yes	11.29
19	4	0		C							G		A			C							G			Chr3:54313532-54313554	Yes	No	0.00
20	4	1		■	A											A			G	A			G			Chr1:168558804-168558826	Yes	Yes	3.31
21	4	1		■	A											A			A	A			G			Chr10:84440789-84440811	Yes	Yes	16.42
22	4	1	A	■				C								C				C			C			Chr4:233088295-233088317	Yes	Yes	1.00
23	4	1				t		G			A			T			T						T			Chr5:6293703-6293726	Yes	Yes	1.55
24	4	1		■	T						C		C								A		G			Chr5:82350877-82350899	Yes	Yes	2.93
25	4	1	C		G								A	g				A					C			Chr1:141609726-141609748	Yes	No	0.00
26	4	1	T		G								A	g				A					C			Chr1:141619342-141619364	Yes	No	0.00
27	4	1		■	A											A			G	A			G			Chr1:89052551-89052573	Yes	No	0.00
28	4	1	A	■								A			G					C			T			Chr10:51974012-51974034	Yes	No	0.00
29	4	1		■									A			C				A	C		A			Chr2:19426157-19426179	Yes	No	0.00
30	4	1	A	■				C				T								C			C			Chr2:36174928-36174950	Yes	No	0.00
31	4	1		■	A											A			G	A			G			Chr2:81561460-81561482	Yes	No	0.00
32	4	1		■	A											A			G	A			G			Chr3:199671651-199671673	Yes	No	0.00
33	4	1		■	A			G											G	A			G			Chr3:222638047-222638069	Yes	No	0.00
34	4	1		■		G				C			A				G						G			Chr5:40664611-40664633	Yes	No	0.00
35	4	1	A						A	a									A	T			T			Chr6:114459472-114459495	Yes	No	0.00
36	4	1	C	■							A			C						T			A			Chr7:15742994-15743016	Yes	No	0.00
37	4	1	A		G						a					A				A			A			Chr7:159564951-159564973	Yes	No	0.00
38	4	2		A		g		G											G	A			G			Chr3:168788394-168788416	Yes	Yes	2.48
39	4	2	T				tc				A						T						T			Chr7:79326098-79326121	Yes	No	0.00
40	5	0		C	G					C	G									T			G			Chr2:80845032-80845054	Yes	Yes	10.71
41	5	0	T		A					A						A				A			G			Chr2:197687616-197687638	Yes	Yes	8.58
42	5	0			A	A						G		C				G					T			Chr1:124111388-124111410	Yes	Yes	2.48
43	5	0			A	A						G		C				G					T			Chr0:4325171-4325193	Yes	Yes	2.48
44	5	0		C			A							G		A	T						G			Chr1:101163004-101163026	Yes	No	0.00
45	5	0			A	A						G		C				G					T			Chr1:104009978-104010000	Yes	No	0.00
46	5	0	T		A											A			G	A			T			Chr1:116346261-116346283	Yes	No	0.00
47	5	0			A	A						G		C				G					T			Chr1:203782886-203782908	Yes	No	0.00
48	5	0			A	A						G		C				G					T			Chr1:92500265-92500287	Yes	No	0.00
49	5	0		A							G	G			A					C			T			Chr10:4860335-4860357	Yes	No	0.00
50	5	0			A	A						G		C				G					T			Chr10:88386224-88386246	Yes	No	0.00
51	5	0			A	A						G		C				G					T			Chr2:239879667-239879689	Yes	No	0.00
52	5	0			A	A						G		C				G					T			Chr2:38715655-38715677	Yes	No	0.00
53	5	0			A	A						G		C				G					T			Chr2:75127397-75127419	Yes	No	0.00
54	5	0			A	A						G		C				G					T			Chr3:107792653-107792675	Yes	No	0.00
55	5	0			A	A						G		C				G					T			Chr3:111193547-111193569	Yes	No	0.00
56	5	0		C			A				A					A	T						G			Chr4:131687629-131687651	Yes	No	0.00
57	5	0		C			A	G								A	T						G			Chr4:96932584-96932606	Yes	No	0.00
58	5	0			A	A						G		C				G					T			Chr5:29439883-29439905	Yes	No	0.00
59	5	0			A	A						G		C				G					T			Chr5:39014962-39014984	Yes	No	0.00
60	5	0	C		A			G											G	A			A			Chr5:47203908-47203930	Yes	No	0.00
61	5	0			A	A						G		C				G					T			Chr5:60919606-60919628	Yes	No	0.00
62	5	0			A	A						G		C				G					T			Chr5:75784576-75784598	Yes	No	0.00
63	5	0			A	A						G		C				G					T			Chr7:34966766-34966788	Yes	No	0.00
64	5	0			A	A						G		C				G					T			Chr7:8090624-8090646	Yes	No	0.00
65	5	0			A	A						G		C				G					T			Chr8:160311153-160311175	Yes	No	0.00
66	5	0			A	A						G		C				G					T			Chr8:25573743-25573765	Yes	No	0.00
67	5	0			A	A						G		C				G					T			Chr8:56845265-56845287	Yes	No	0.00
68	5	1	T		A											■		A			A	G	C			Chr2:45333382-45333404	Yes	Yes	0.00
69	5	1	A	■				T			A									T	T		C			Chr1:247722181-247722203	Yes	Yes	4.68
70	5	1		■	A	A						G		C				G					T			Chr8:78781894-78781916	Yes	Yes	1.33
71	5	1	C		G	T					g	A						A					C			Chr1:124001644-124001671	Yes	No	0.00
72	5	1		■	A	A						G		C				G					T			Chr1:175146350-175146372	Yes	No	0.00
73	5	1	T		A			G								G			■			G	C			Chr1:180655201-180655223	Yes	No	0.00
74	5	1		■	A	A						G		C				G					T			Chr1:21083695-21083717	Yes	No	0.00
75	5	1	T		A											■		A			A	G	C			Chr1:5273647-5273669	Yes	No	0.00
76	5	1	C	■			A							G		A	T						G			Chr1:73592041-73592063	Yes	No	0.00
77	5	1				G	A	G				A						A				c	G			Chr10:37395498-37395520	Yes	No	0.00
78	5	1		■	A	A						G		C				G					T			Chr10:75750456-75750478	Yes	No	0.00
79	5	1	A	■			T											T		A	A		C			Chr2:81171011-81171033	Yes	No	0.00
80	5	1		■	A	A						G		C				G					T			Chr2:82344809-82344831	Yes	No	0.00
81	5	1		■	A	A						G		C				G					T			Chr2:84402863-84402885	Yes	No	0.00
82	5	1		■	A	A						G		C				G					T			Chr3:16052720-16052742	Yes	No	0.00
83	5	1	T		A											■		A			A	G	C			Chr4:26448021-26448043	Yes	No	0.00
84	5	1		C							C		A			C			■			G	C			Chr4:35475536-35475558	Yes	No	0.00
85	5	1		■	A	A						G		C				G					T			Chr4:72754228-72754250	Yes	No	0.00
86	5	1				G	A	G				A						A					lcG			Chr5:112910962-112910984	Yes	No	0.00
87	5	1		■	A	A						G		C				G					T			Chr5:141546929-141546951	Yes	No	0.00
88	5	1		■	A	A						G		C				G					T			Chr5:189221660-189221682	Yes	No	0.00
89	5	1	T		A											■		A			A	G	C			Chr5:205487171-205487193	Yes	No	0.00
90	5	1	A	■					A	A	A									A			A			Chr5:41228760-41228782	Yes	No	0.00
91	5	1	C	■							C		A				C		A				T			Chr6:167702031-167702053	Yes	No	0.00
92	5	1		■	A	A						G		C				G					T			Chr6:82650049-82650071	Yes	No	0.00
93	5	1		■	A	A						G		C				G					T			Chr8:146121378-146121400	Yes	No	0.00
94	5	1		■		A	A				G		A								T		T			Chr8:157442665-157442687	Yes	No	0.00
95	5	1		■	A	A						G		C				G					T			Chr8:79394412-79394434	Yes	No	0.00
96	5	2		C		■	■							C	G	G					T		C			Chr7:124810037-124810059	Yes	No	0.00

Supplementary Table 7. MIPS genotyping of 390 T_0 maize plants generated from three different target sites and delivery methods. Any allele, mutant or WT represent >5% of total reads to be called an allele. Percent mutant alleles indicate total mutant alleles/total alleles from plants analyzed. Mosaic plants contain >2 alleles. Homozygous mutant plants contain single mutant allele with >90% reads

Target Site	Transformation Method	Number of Plants Analyzed	Percent Mutant Alleles	Mosaic Plants	Homozygous WT Plants	Homozygous Mutant Plants	Heterozygous Mut/Mut Plants	Heterozygous WT/Mut Plants
M1	Agro	26	61.30%	0.00%	46.20%	34.60%	19.20%	0.00%
M2	DNA-particle gun	112	95.90%	8.10%	5.20%	34.90%	45.90%	5.80%
	Agro	117	99.00%	9.30%	0.00%	49.20%	39.80%	1.70%
	RNP-particle gun	94	33.30%	0.00%	61.50%	15.40%	15.40%	7.70%
M3	Agro	41	94.30%	5.60%	8.30%	55.60%	30.60%	0.00%
